# Ionic Remote α-C–H Allenylation of Silyl Ethers Involving a [1,5]-Hydride Shift Promoted by Silylium-Ion Regeneration

**DOI:** 10.1021/jacs.4c18137

**Published:** 2025-01-31

**Authors:** Honghua Zuo, Sebastian Kemper, Hendrik F. T. Klare, Martin Oestreich

**Affiliations:** Institut für Chemie, Technische Universität Berlin, 10623 Berlin, Germany

## Abstract

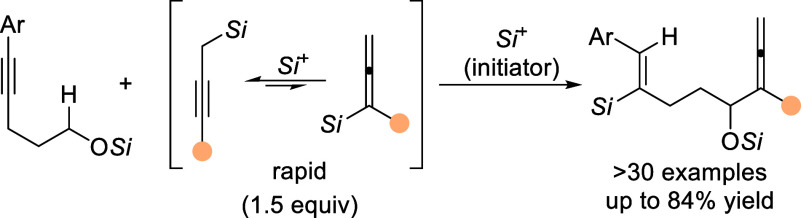

A silylium-ion-promoted α-C–H allenylation of silyl ethers tethered to an internal alkyne is described. The actual intermolecular allenylation event occurs after the *trans*-selective hydrosilylation of the alkyne, where an in situ-generated β-silicon-stabilized vinyl cation engages in an intramolecular [1,5]-hydride shift. This process transforms the silyl ether into a silylcarboxonium ion, which reacts with propargylsilanes as nucleophiles, formed by the rapid silylium-ion-catalyzed isomerization of allenylsilanes. As part of the allenylation step, the propagating silylium-ion electrophile is regenerated, thereby closing the catalytic cycle. An allylsilane is also applicable in this transformation, producing the corresponding α-C–H allylation product in high yield.

## Introduction

**Scheme 1:**
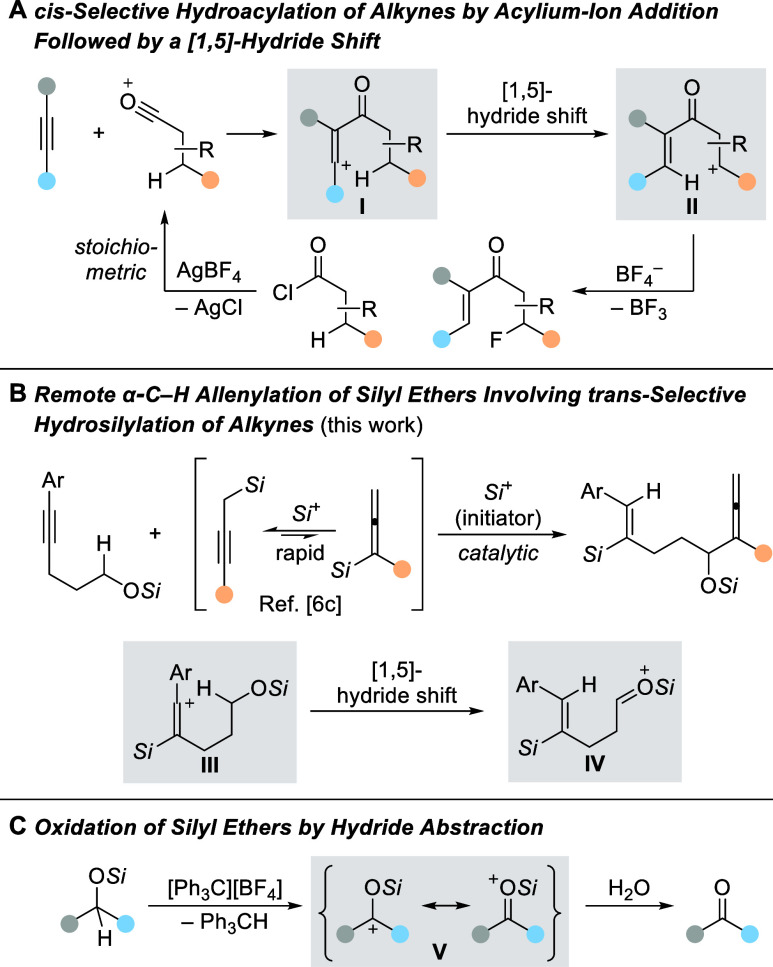
Hydride-Shift Processes in the Hydroacylation of Alkynes, α-C–H Allenylation of Silyl Ethers, and Oxidation of Silyl Ethers R = aryl and/or alkyl, Ar = aryl, *Si* = triorganosilyl. Counteranions BF_4_^–^ and [HCB_11_H_5_Br_6_]^−^ have been omitted for clarity.

Hydride transfer reactions can be exploited as a redox-neutral tool to activate unreactive C(sp^3^)–H bonds by an ionic mechanism.^1^ Especially [1,5]-hydride shift reactions from a C(sp^3^)–H to an unsaturated carbon atom followed by a ring closure onto the newly formed electrophilic site have been utilized for that purpose.^1a,1c^ Except for a few isolated reports by Shchegolev, Smit, and coworkers, examples that involve the intermolecular capture of such carbocation-like intermediates arising from a vinyl cation-initiated [1,5]-hydride shift by an external nucleophile are quite rare (Scheme 1A).^2^ In their seminal work, acyl chlorides are primed for their addition across C–C triple bonds with a stoichiometric amount of AgBF_4_ to eventually form vinyl cation intermediate **I** (gray box). Subsequent to this acylium-ion addition, a [1,5]-hydride shift leads to another carbocation intermediate **II** that is then intercepted by fluoride from the tetrafluoroborate counteranion. Aside from C(sp^3^)–F bond formation, that work also includes two examples of C(sp^3^)–C(sp^2^) bond formation where carbocation intermediate **II** engages in a Friedel–Crafts reaction with the arene solvent (not shown).^2f^

The Shchegolev–Smit system raises several key questions: (1) What other electrophile or carbophilic Lewis acid can turn a C–C triple bond into a hydride acceptor?^3^ (2) How can this stoichiometric reaction be translated into a catalytic process? (3) Would there be a terminating carbon nucleophile that would release the above electrophile to maintain catalytic turnover? Silylium ions or silylium-ion-like cations^4^ do fulfill objective 1 by forming β-silicon-stabilized vinyl cations with alkynes.^5,6^ With a cationic silicon electrophile as an initiator in place, the proper choice of a silicon-containing nucleophile could then secure the self-regeneration of the silylium-ion initiator^4,7^ and at the same time close the catalytic cycle, thereby achieving objectives 2 and 3. Based on these considerations, we designed the reaction cascade outlined in Scheme 1B: Electrophilic alkyne activation by silylation gives the crucial vinyl cation intermediate **III** (gray box), which is supposed to abstract a hydride from the methylene group α to a silyl ether by a [1,5]-hydride shift to yield silylcarboxonium ion **IV**. While their use in this context is surprisingly uncommon, alkyl and aryl ethers have been employed more frequently.^1^ However, Jung had shown that the oxidation of silyl ethers involving intermediate **V** by trityl salt-mediated hydride abstraction is a facile transformation (Scheme 1C).^8^ Our idea was to react silylcarboxonium ion **IV** with propargyl- and allenylsilanes, respectively, with the former being rapidly generated in situ from the corresponding allenylsilane.^6c^ We report here the realization of this strategy that enables an ionic remote α-C–H allenylation of silyl ethers.

## Results and Discussion

We began our investigation with triisopropylsilyl-protected 5-phenylpent-4-yn-1-ol (**1a**) and α-methyl-substituted allenylsilane **2a** as model substrates (Table 1). Using 1.0 mol % of silylium carborate [Me_3_Si(HCB_11_H_5_Br_6_)] as the initiator in benzene, the reaction proceeded smoothly at ambient temperature to deliver the desired product **3aa** in 51% yield (entry 1) but both starting materials were not fully converted. We therefore raised the reaction temperature to 50 °C, and it turned out to be beneficial as the yield increased to 74% (entry 2). A screening of arene solvents showed that the reaction is largely dependent on their polarity (entries 3–5), and toluene gave essentially the same result as benzene (entry 6). As expected, Reed’s benzenium ion^9^ (entry 7) and the trityl salt [Ph_3_C][HCB_11_H_5_Br_6_] (entry 8) worked equally well to initiate this reaction, but both were slightly inferior. Conversely, no desired product was detected with Me_3_SiOTf (entry 9) and Me_3_SiNTf_2_ (entry 10) as the initiators. Given the inherent volatility of allenylsilane, the amount of allenylsilane **2a** was gradually increased from 1.2 to 2.0 equiv to improve the reaction efficiency (entries 11 and 12). At 1.5 equiv of **2a**, the yield of **3aa** reached a maximum, furnishing product **3aa** in a 78% isolated yield (entry 11). In all cases, the *Z* configuration of the alkene was observed exclusively, thus corresponding to a *trans* hydrosilylation of the alkyne.

**Table 1:**
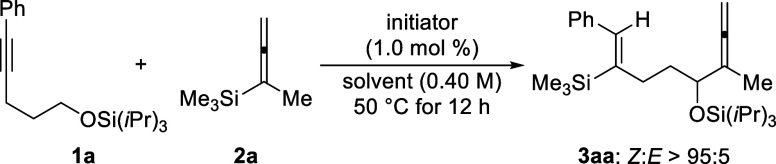
Optimization of the Reaction Conditions^a^^,^^b^

entry	initiator	solvent	**2a** (equiv)	yield (%)^c^
1^d^	[Me_3_Si(HCB_11_H_5_Br_6_)]	PhH	1.2	51
2	[Me_3_Si(HCB_11_H_5_Br_6_)]	PhH	1.2	74
3	[Me_3_Si(HCB_11_H_5_Br_6_)]	PhF	1.2	54
4	[Me_3_Si(HCB_11_H_5_Br_6_)]	PhCl	1.2	21
5	[Me_3_Si(HCB_11_H_5_Br_6_)]	*o*-C_6_H_4_Cl_2_	1.2	<10
6	[Me_3_Si(HCB_11_H_5_Br_6_)]	PhMe	1.2	71
7	[H(C_6_H_6_)][HCB_11_H_5_Br_6_]	PhH	1.2	70
8	[Ph_3_C][HCB_11_H_5_Br_6_]	PhH	1.2	69
9	Me_3_SiOTf	PhH	1.2	n.d.
10	Me_3_SiNTf_2_	PhH	1.2	n.d.
11	[Me_3_Si(HCB_11_H_5_Br_6_)]	PhH	1.5	82^e^
12	[Me_3_Si(HCB_11_H_5_Br_6_)]	PhH	2.0	73

a All reactions were performed on a 0.20 mmol scale under an argon atmosphere in 0.5 mL of the indicated solvent.

b *Z*:*E* > 95:5 in all cases, as verified by ^1^H NMR spectroscopy of the crude reaction mixtures.

c Yields were determined by ^1^H NMR spectroscopy using CH_2_Br_2_ as an internal standard.

d Reaction was performed at room temperature.

e 78% isolated yield after flash chromatography on silica gel. n.d. = not detected.

**Scheme 2:**
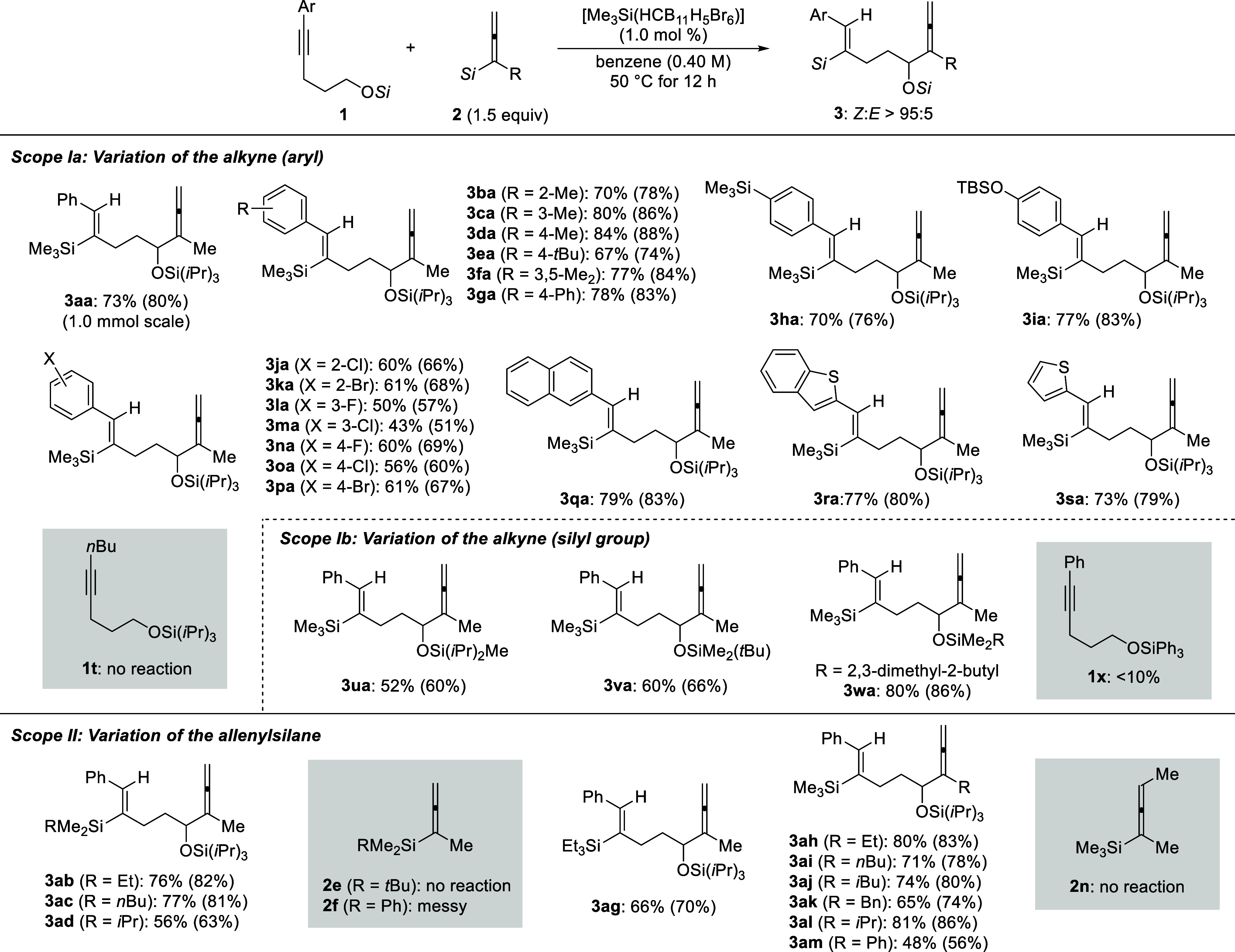
Substrate Scope of the Remote α-C–H Allenylation of Silyl Ethers^,^^,^ All reactions were performed with silyl ether **1** (0.20 mmol), allenylsilane **2** (0.30 mmol, 1.5 equiv), and initiator [Me_3_Si(HCB_11_H_5_Br_6_)] (2.0 μmol, 1.0 mol %) under an argon atmosphere in benzene (0.5 mL) at 50 °C for 12 h. *Z*:*E* > 95:5 in all cases, as verified by ^1^H NMR spectroscopy of the crude reaction mixtures. Isolated yields refer to analytically pure material after flash chromatography on silica gel; yields in parentheses were determined by ^1^H NMR spectroscopy using CH_2_Br_2_ as an internal standard. TBS = *tert*-butyldimethylsilyl.

With the optimized reaction conditions in hand, we assessed the generality of this α-C–H allenylation reaction (Scheme 2). The model reaction was run on a 1.0 mmol scale, resulting in a slightly diminished yield of 73% for **3aa**. A range of silyl ethers containing a methyl (**1b**–**d**), *t*-butyl (**1e**), and two methyl groups (**1f**) at the aryl ring were well tolerated, forming the corresponding products **3ba**–**fa** in good to high yields. Apart from these alkyl-substituted cases, a representative example of a 4-phenyl-substituted substrate (**1g**) was also tested, affording the desired product **3ga** in a high yield of 78%. Additionally, no desilylation was observed for **1h** bearing a trimethylsilyl group. Notably, a decent yield was also recorded for reactant **1i** with a silyl ether functional group installed at the *para*-position of the aryl ring. Not surprisingly, a series of halogen-substituted silyl ethers proceeded smoothly in this reaction, albeit the yields of **3ja**–**pa** were relatively lower than those of non-halogen-substituted substrates. We attribute this to a weak deactivation effect of the halogen atoms. In addition, using analogous β-naphthyl-substituted silyl ether **1q** as a substrate gave an equally high yield. It is noteworthy that applying a benzo[*b*]thien-2-yl- and a thien-2-yl-substituted substrate was feasible as well, giving rise to products **3ra** and **3sa** in 77% and 73% yield, respectively. Surprisingly, potentially competing Lewis adduct formation between the sulfur donor and the silylium ion had no significant effect on this reaction.^6b^ Unfortunately, substrate **1t,** as an example with an alkyl substituent at the alkyne terminus, showed no conversion, presumably because the corresponding alkyl-substituted vinyl cation is energetically not accessible. In turn, the neighboring group effect of the aryl group facilitates vinyl cation formation.^10^ Next, we examined a number of other silyl protecting groups. Both (*i*Pr)_2_MeSi (**1u**) and *t*BuMe_2_Si (**1v**) groups displayed a similar result, but **1w** with a bulkier 2,3-dimethyl-2-butyl group was greatly superior. However, a Ph_3_Si group as in **1x** was reluctant to react, and other aryl-substituted silyl ethers with Me_2_PhSi and MePh_2_Si protections were not even chemically stable (not shown).

Variation of allenylsilane **2** was first probed for different substitution patterns at the silicon atom while keeping the R part as a methyl group. Replacing one of the methyl groups in Me_3_Si with an ethyl (**2b**), *n*-butyl (**2c**), or isopropyl (**2d**) group led to the formation of products **3ab**–**ad** in moderate to good yields. Increasing the steric hindrance is detrimental to this transformation, and the *t*BuMe_2_Si group (**2e**) was too bulky for this reaction to occur. The messy reaction outcome with a Me_2_PhSi group (**2f**) could result from known substituent redistribution processes.^4a,11^ In addition, product **3ag** bearing an Et_3_Si group was generated in a synthetically useful yield. We then turned to the variation of the R group attached to the allenyl moiety, while not changing the Me_3_Si part. A broad array of linear or branched alkyl substituents (**2h**–**l**) were well tolerated, allowing for the formation of products **3ah**–**al** in the usual yield range. Particularly, an aryl substituent as in **2m** was compatible, while the internal allenylsilane **2n** was not.

**Scheme 3:**
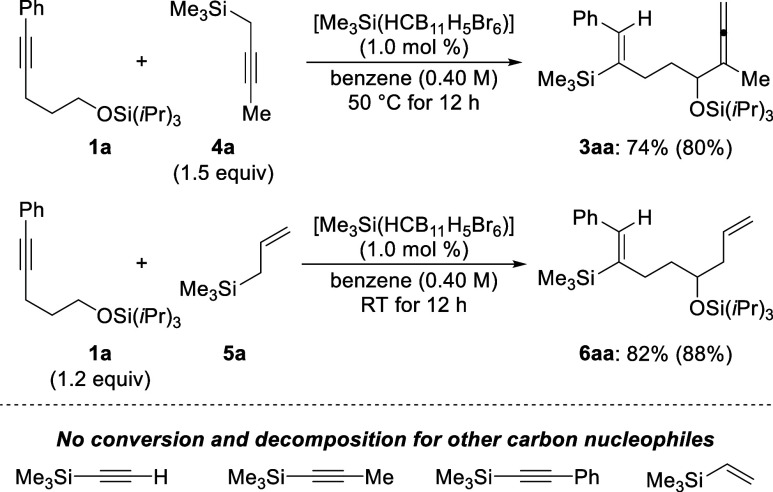
Remote α-C–H Allenylation and Allylation of the Silyl Ether^,^^,^ Reactions were performed with **1a** (0.2 mmol), propargylsilane **4a** (0.3 mmol, 1.5 equiv), or **1a** (0.24 mmol, 1.2 equiv), allylsilane **5a** (0.20 mmol), and initiator [Me_3_Si(HCB_11_H_5_Br_6_)] (2.0 μmol, 1.0 mol %) under an argon atmosphere in benzene (0.5 mL) at 50 °C or room temperature for 12 h. *Z*:*E* > 95:5, as verified by ^1^H NMR spectroscopy of the crude reaction mixtures. Isolated yields refer to analytically pure material after flash chromatography on silica gel; yields in parentheses were determined by ^1^H NMR spectroscopy using CH_2_Br_2_ as an internal standard.

In an attempt to expand this protocol to other carbon nucleophiles (Scheme 3, top), we found that directly employing propargylsilane **4a** as the substrate resulted in product formation in 74% yield. This observation strongly supports a mechanism where **4a** is indeed the external nucleophile in the catalytic cycle, as silylium-ion-promoted allenyl-to-propargylsilane isomerization is known to be a fast process.^6c^ Except for allylsilane **5a** that underwent a similar process to achieve the remote α-C–H allylation of silyl ethers, the other nucleophiles shown at the bottom of Scheme 3 were reluctant to give the desired products. No conversion or decomposition was observed for these carbon nucleophiles.

**Scheme 4:**
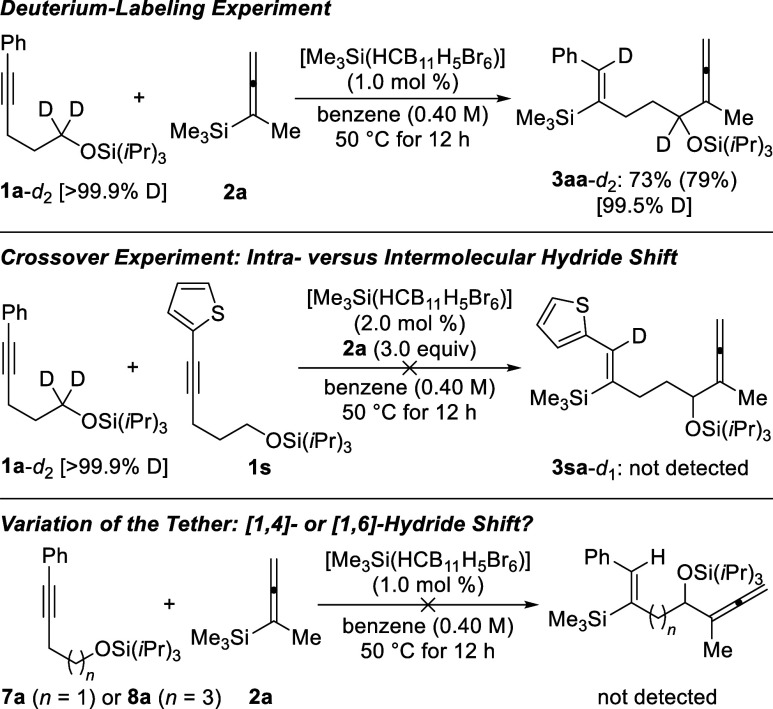
Mechanistic Control Experiments^,^^,^ Isolated yields refer to analytically pure material after flash chromatography on silica gel. Yields in parentheses were determined by ^1^H NMR spectroscopy using CH_2_Br_2_ as an internal standard. The overall deuteration grades of **1a**-*d*_2_ and **3aa**-*d*_2_ were calculated by HRMS analysis; the exact deuteration grades for each relevant position were ≥95%, as estimated by ^1^H NMR spectroscopy.

To gain insight into the reaction mechanism, three control experiments were performed (Scheme 4). We had already speculated that a [1,5]-hydride shift is the key step of this cascade. We therefore conducted a deuterium-labeling experiment, and the incorporation of deuterium into the vinylic position of product **3aa**-*d*_2_ supported our hypothesis (top). To exclude any potential intermolecular hydride shift, a crossover experiment was conducted (middle), and only products **3aa**-*d*_2_ and **3sa** were obtained; no incorporation of deuterium into the vinylic position of **3sa** ruled out this alternative pathway. We then explored other, yet less likely, [1,4]- or [1,6]-hydride transfers by treating substrate **7a** or **8a** with allenylsilane **2a** under the standard reaction conditions (bottom). However, no conversion was seen for either starting material, which is again in line with the intramolecular nature of the hydride transfer.

**Scheme 5:**
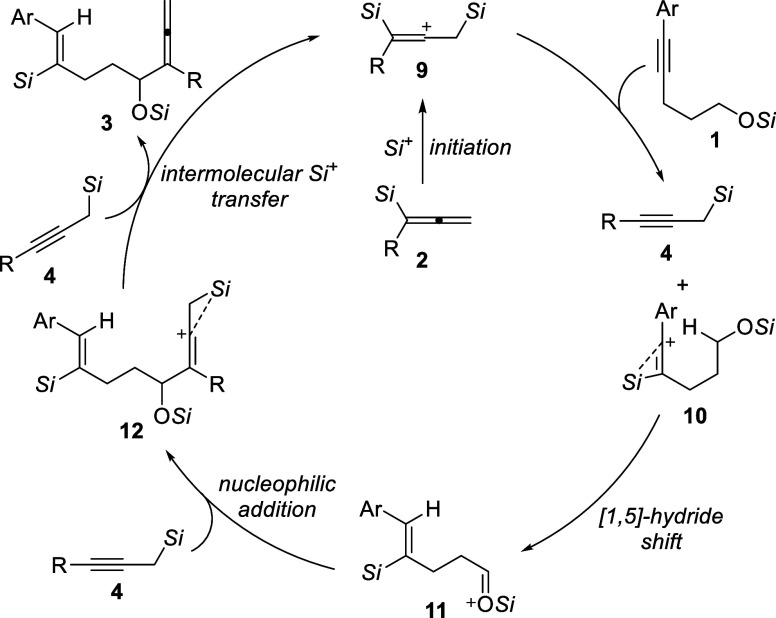
Proposed Catalytic Cycle The counteranion [HCB_11_H_5_Br_6_]^−^ is omitted for clarity.

Based on our previous (3 + 2) annulation of internal alkynes with allenylsilanes^6c^ and the outcome of the above control experiments, we propose the catalytic cycle outlined in Scheme 5. The reaction starts with the formation of the β,β’-bis(silicon)-stabilized vinyl cation intermediate **9** from allenylsilane **2**. Site-selective silylium-ion transfer from **9** to the alkyne moiety of silyl ether **1** generates propargylsilane **4** along with the β-silicon-stabilized vinyl cation **10**, which subsequently undergoes the anticipated [1,5]-hydride shift to yield the silylcarboxonium ion **11**. Nucleophilic attack of the C–C triple bond in **4** leads to another β-silicon-stabilized vinyl cation **12**. From this, the desired product **3** is released by β-elimination of the silylium ion, which, in turn, is captured by propargylsilane **4** to regenerate carbocation **9** as the propagating silylium-ion species.

**Scheme 6:**
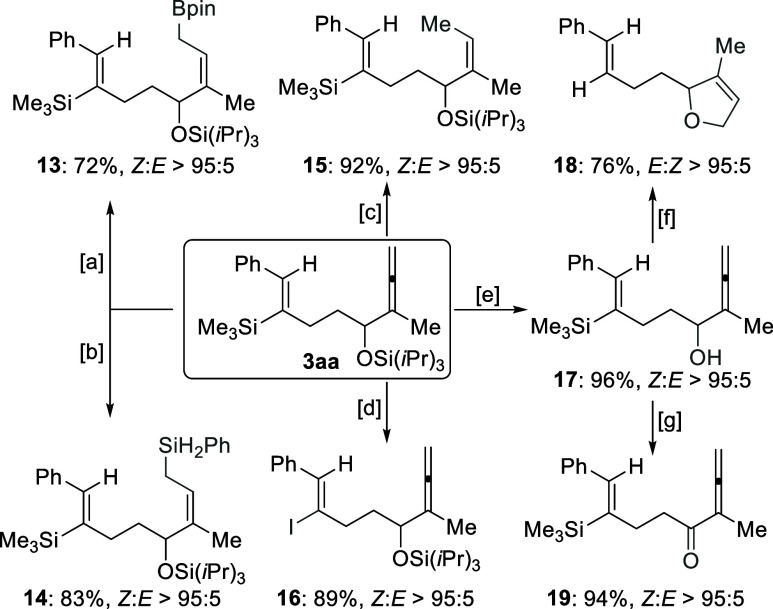
Synthetic Transformations^,^ Isolated yields refer to analytically pure material after flash chromatography on silica gel. Diastereomeric ratios were verified by ^1^H NMR spectroscopy of the crude reaction mixtures. Reagents and reactions conditions: [a] [Ir(cod)Cl]_2_ (1.0 mol %), dppm (2.0 mol %), HBpin (1.2 equiv), CH_2_Cl_2_, RT for 12 h; [b] Co(acac)_2_ (1.0 mol %), Xantphos (1.0 mol %), PhSiH_3_ (1.1 equiv), THF, RT for 2 h; [c] Lindlar’s catalyst (10 wt %), H_2_ (balloon), EtOH/EtOAc (1:1), RT for 2 h; [d] NIS (1.2 equiv), MeCN, RT for 2 h; [e] TBAF (1.2 equiv), THF, RT for 2 h; [f] AgNO_3_ (1.2 equiv), acetone/H_2_O (1:1), 40 °C for 12 h; [g] DMP (1.1 equiv), CH_2_Cl_2_, RT for 1.5 h. dppm = 1,1-bis(diphenylphosphino)methane, NIS = *N*-iodosuccinimide, TBAF = tetra-*n*-butylammonium fluoride, DMP = Dess-Martin periodinane.

To illustrate the synthetic value of the densely functionalized products, we investigated a series of synthetic transformations of **3aa** (Scheme 6). An iridium-catalyzed regio- and stereoselective hydroboration of the allene unit with pinacolborane preferentially occurred at the less hindered terminal double bond, giving the allyl boronic ester **13** in good yield.^12^ Hydrosilylation of this terminal double bond was readily achieved by applying the method developed by Ge and coworkers,^13^ yielding the allylsilane product **14** in 83% yield with superb regio- and stereoselectivity. Remarkably, the allene moiety can undergo a chemoselective semihydrogenation at the terminal double bond with Lindlar’s catalyst^14^ to form product **15** as a single stereoisomer in excellent yield. Moreover, the vinylic silyl group in **3aa** can be easily converted into a vinyl iodide **16** through iododesilylation with NIS.^15^ Upon deprotection with TBAF, α-allenol **17** was obtained in almost quantitative yield, which is a versatile linchpin that allows for further derivatization.^16^ Treatment of **17** with AgNO_3_ in aqueous acetone provided access to a 2,5-dihydrofuran derivative **18** in good yield,^17^ along with a protodesilylation of the vinylsilane moiety. Dess-Martin oxidation cleanly converted the alcohol **17** into ketone product **19** in 94% yield.^18^

## Conclusions

In summary, we disclose here a new approach to C–C bond formation in the α position of silyl ethers by an ionic mechanism. This has been achieved by a silylium-ion-promoted hydrosilylation of an internal C–C triple bond, where the hydride is intramolecularly delivered from a remote methylene group α to a tethered silyl ether. That vinyl cation-initiated [1,5]-hydride shift yields a silylcarboxonium ion, which is intermolecularly captured by propargyl- and allylsilanes as nucleophiles. The reaction displays broad scope with the functional-group tolerance typically seen in silylium-ion catalysis.

## Data Availability

The data underlying this study are available in the published article and its Supporting Information.

## Supporting Information Available

Experimental procedures, including the synthesis of starting materials and characterization data, and NMR spectral data for all new compounds (PDF)
