# Supplementary material for: Ionic Remote α-C–H Allenylation of Silyl Ethers Involving a [1,5]-Hydride Shift Promoted by Silylium-Ion Regeneration
Source: J Am Chem Soc. 2025 Jan 31;147(6):5426–31. doi: 10.1021/jacs.4c18137 (PMC11826908; doi:10.1021/jacs.4c18137)
Supplement: Supplementary file 1 — ja4c18137_si_001.pdf [file ja4c18137_si_001.pdf]

Ionic Remote α-C–H Allenylation of Silyl Ethers Involving a [1,5]-Hydride Shift Promoted by Silylium-Ion Regeneration

Honghua Zuo, Sebastian Kemper, Hendrik F. T. Klare, and Martin Oestreich*

Institut für Chemie, Technische Universität Berlin
Straße des 17. Juni 115, 10623 Berlin, Germany

martin.oestreich@tu-berlin.de

Supporting Information

Table of Contents

1 General Information S3

2 Optimization of the Reaction Conditions S4

3 Experimental Details for the Substrate Synthesis S5

3.1 Synthesis of Silyl Ethers **1**: Variation of the Alkyne Aryl Group (GP 1) S5

3.2 Synthesis of Silyl Ethers **1**: Variation of the Silyl Group (GP 2) S5

3.3 Characterization Data for Silyl Ethers **1** S6

3.4 Synthesis of Allenylsilanes **2** (GP 3) S16

3.5 Characterization Data for Allenylsilanes **2** S16

4 Experimental Details for the Silylium-Ion-Promoted Ionic Remote *α*-C–H Allenylation of Silyl Ethers S18

4.1 General Procedure for the *α*-C–H Allenylation of Silyl Ethers (GP 4) S18

4.2 Characterization Data for *α*-C–H Allenylation Products **3** S18

5 Experimental Details for the Silylium-Ion-Promoted Ionic Remote *α*-C–H Allenylation and Allylation of Silyl Ether 1a S37

5.1 Procedure for the *α*-C–H Allenylation of Silyl Ether **1a** with Propargylsilane **4a** S37

5.2 Procedure for the *α*-C–H Allylation of Silyl Ether **1a** with Allylsilane **5a** S37

6 Mechanistic Control Experiments S39

7 Experimental Details for the Synthetic Transformations of 3aa S45

8 Determination of the Alkene Configuration S52

9 NMR Spectra S54

10 References S263

1 General Information

All reactions were performed in flame-dried glassware using an MBraun glovebox (O_2 < 0.5 ppm, H_2O < 1.0 ppm) or conventional Schlenk techniques under a static pressure of argon (glovebox) or nitrogen (fume hood) unless otherwise stated. All given elevated temperatures refer to external oil bath temperatures. Standard solvents and reagents were obtained from commercial suppliers and used as received unless otherwise stated. Glassware was dried overnight at 150 °C or flame dried using a heat gun. All plastic syringes and needles used in the glovebox were dried overnight at 60 °C. Liquids and solutions were transferred with syringes. Technical grade solvents for extraction and chromatography were distilled prior to use. Tetrahydrofuran (THF) was dried over sodium and freshly distilled prior to use. Dry benzene (C_6H_6) and *n*-pentane were obtained from an MBraun solvent purification system (SPS-800), degassed by three freeze-pump-thaw cycles, and stored in a glovebox over thermally activated 4 Å molecular sieves. Dichloromethane (CH_2Cl_2), toluene (C_7H_8), fluorobenzene (C_6H_5F), chlorobenzene (C_6H_5Cl), bromobenzene (C_6H_5Br), and 1,2-dichlorobenzene (1,2-$C_6H_4Cl_2$) were dried over CaH_2, distilled, degassed by three freeze-pump-thaw cycles, and stored in a glovebox over thermally activated 4 Å molecular sieves. Silylium carborates [$R_3Si(HCB_{11}H_5Br_6)$] (R = Me,[S1] Et,[S2] and *i*Pr[S3]) and Reed's acid [$H(C_6H_6)$][$HCB_{11}H_5Br_6$][S4] were synthesized according to reported procedures. Thin-layer chromatography (TLC) was performed on Macherey-Nagel Alugram® Xtra SIL G/UV254 silica gel 60 pre-coated aluminum-backed plates (200 µm layer thickness). Product spots were visualized under UV light (λ_{max} = 254 nm) and with a ceric ammonium molybdate stain. Column chromatography was performed on Grace 60 (40–63 µm, 230–400 mesh, ASTM) silica gel. ^1H, ^{13}C, ^{19}F, and ^{29}Si NMR spectra were recorded in $CDCl_3$ on a Bruker AV400, AV500, and AV700 instrument, respectively. Chemical shifts are reported in parts per million (ppm) and are referenced to the residual solvent resonance as the internal standard ($CHCl_3$: δ = 7.26 ppm for ^1H NMR and $CDCl_3$: δ = 77.16 ppm for ^{13}C NMR). ^{19}F and ^{29}Si NMR spectra are referenced in compliance with the unified scale for NMR chemical shifts as recommended by the IUPAC stating the chemical shift relative to CCl_3F and TMS, respectively.[S5] Data are reported as follows: chemical shift, multiplicity (s = singlet, d = doublet, t = triplet, q = quartet, quint = quintet, sept = septet, m = multiplet, br = broad), coupling constants (Hz), and integration. Infrared (IR) spectra were recorded on an Agilent Technologies Cary 630, and the signals are reported in wavenumbers (cm^{-1}). High resolution mass spectra (HRMS) were obtained from the Center for Mass Spectrometry at the Institut für Chemie, Technische Universität Berlin on a Thermo Fisher Scientific LTQ Orbitrap XL apparatus using APCI or ESI technique with a linear ion trap analyzer.

2 Optimization of the Reaction Conditions[a,b]

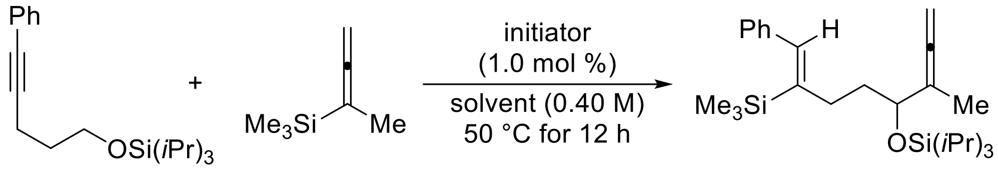

1a **2a** **3aa**: *Z*:*E* > 95:5

Entry	Initiator	Solvent	**2a** (Equiv)	Yield (%)[c]
1[d]	$[Me_3Si(HCB_{11}H_5Br_6)]$	PhH	1.2	51
2	$[Me_3Si(HCB_{11}H_5Br_6)]$	PhH	1.2	74
3	$[Me_3Si(HCB_{11}H_5Br_6)]$	PhF	1.2	54
4	$[Me_3Si(HCB_{11}H_5Br_6)]$	PhCl	1.2	21
5	$[Me_3Si(HCB_{11}H_5Br_6)]$	PhBr	1.2	25
6	$[Me_3Si(HCB_{11}H_5Br_6)]$	1,2-$C_6H_4Cl_2$	1.2	<10
7	$[Me_3Si(HCB_{11}H_5Br_6)]$	PhMe	1.2	71
8	$[Me_3Si(HCB_{11}H_5Br_6)]$	*p*-xylene	1.2	52
9	$[Me_3Si(HCB_{11}H_5Br_6)]$	$PhCF_3$	1.2	18
10	$[Me_3Si(HCB_{11}H_5Br_6)]$	$CHCl_3$	1.2	16
11	$[Me_3Si(HCB_{11}H_5Br_6)]$	*n*-hexane	1.2	trace
12	$[Et_3Si(HCB_{11}H_5Br_6)]$	PhH	1.2	72
13	$[iPr_3Si(HCB_{11}H_5Br_6)]$	PhH	1.2	70
14	$[Et_3Si(toluene)][B(C_6F_5)_4]$	PhH	1.2	65
15	$[H(C_6H_6)][HCB_{11}H_5Br_6]$	PhH	1.2	70
16	$[Ph_3C][HCB_{11}H_5Br_6]$	PhH	1.2	69
17	$AlCl_3$	PhH	1.2	n.d.
18	TfOH	PhH	1.2	n.d.
19	Me_3SiOTf	PhH	1.2	n.d.
20	Me_3SiNTf_2	PhH	1.2	n.d.
21	$[Me_3Si(HCB_{11}H_5Br_6)]$	PhH	1.5	82 (78)[e]
22	$[Me_3Si(HCB_{11}H_5Br_6)]$	PhH	2.0	73

[a]All reactions were performed on a 0.20 mmol scale under argon atmosphere in 0.5 mL of indicated solvent. [b]*Z*:*E* > 95:5 in all cases as verified by ^{1}H NMR spectroscopy of the crude reaction mixtures. [c]Yields were determined by ^{1}H NMR spectroscopy using CH_2Br_2 as an internal standard. [d]Reaction was performed at room temperature. [e]Isolated yield after flash chromatography on silica gel is given in parentheses. n.d. = not detected.

3 Experimental Details for the Substrate Synthesis

3.1 Synthesis of Silyl Ethers 1: Variation of the Alkyne Aryl Group (GP 1)

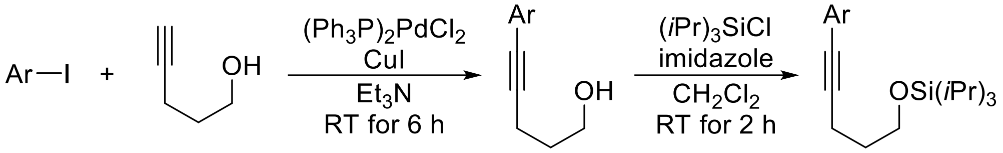

S1 **S2** **S3** **1**

Step 1: According to a reported procedure,[S6] a solution of iodoarene **S1** (5.0 mmol, 1.0 equiv) and pent-4-yn-1-ol (**S2**, 6.0 mmol, 504.7 mg 1.2 equiv) in Et_3N (5.0 mL) is added dropwise to a suspension of $(Ph_3P)_2PdCl_2$ (70.2 mg, 0.10 mmol, 2.0 mol %) and CuI (28.6 mg, 0.15 mmol, 3.0 mol %) in Et_3N (25 mL). The reaction mixture is stirred at room temperature for additional 6 h. Upon completion (monitored by TLC), the reaction is quenched by the addition of saturated aqueous NH_4Cl solution (15 mL), and the resulting mixture is extracted with CH_2Cl_2 (3 × 20 mL). The combined organic phases are dried over Na_2SO_4 and concentrated under reduced pressure. Purification of the residue by flash column chromatography on silica gel using *n*-pentane and EtOAc as the eluent (20:1→10:1→5:1) affords 5-aryl-substituted pent-4-yn-1-ol **S3** as a light yellow oil.

Step 2: According to a reported procedure,[S7] 5-aryl-substituted pent-4-yn-1-ol **S3** (4.0 mmol, 1.0 equiv) is dissolved in CH_2Cl_2 (40 mL), and imidazole (544.6 mg, 8.0 mmol, 2.0 equiv) is added in one portion. After stirring for 5 min, $(i\text{Pr})_3SiCl$ (925.5 mg, 4.8 mmol, 1.2 equiv) is added dropwise to the solution. The reaction mixture is stirred at room temperature for additional 2 h. Upon completion (monitored by TLC), the reaction mixture is poured into water (100 mL), and the organic layer is separated. The aqueous phase is extracted with CH_2Cl_2 (3 × 20 mL). The combined organic phases are dried over Na_2SO_4 and concentrated under reduced pressure. Purification of the residue by flash column chromatography on silica gel using *n*-pentane as the eluent affords the corresponding silyl ether **1** as a colorless oil in analytically pure form.

3.2 Synthesis of Silyl Ethers 1: Variation of the Silyl Group (GP 2)

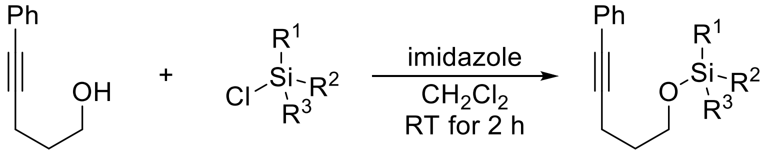

S4 **S5** **1**

5-Phenylpent-4-yn-1-ol (**S4**, 4 mmol, 640.9 mg, 1.0 equiv), prepared according to **GP 1**, is dissolved in CH_2Cl_2 (40 mL), and imidazole (544.6 mg, 8.0 mmol, 2.0 equiv) is added in one portion. After stirring for 5 min, chlorosilane **S5** (4.8 mmol, 1.2 equiv) is added dropwise to the solution, and the reaction mixture is stirred at room temperature for additional 2 h.

Upon completion (monitored by TLC), the reaction mixture is poured into water (100 mL), and the organic layer is separated. The aqueous phase is extracted with CH_2Cl_2 (3 × 20 mL). The combined organic phases are dried over Na_2SO_4 and concentrated under reduced pressure. Purification of the residue by flash column chromatography on silica gel using *n*-pentane as the eluent affords the corresponding silyl ether **1** as a colorless oil in analytically pure form.

3.3 Characterization Data for Silyl Ethers 1

Triisopropyl((5-phenylpent-4-yn-1-yl)oxy)silane (1a)

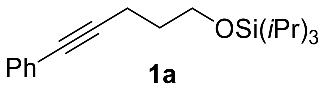

1a

$C_{20}H_{32}OSi$

M = 316.56 g/mol

Prepared according to **GP 1**. Flash column chromatography on silica gel using *n*-pentane as the eluent afforded substrate **1a** as a colorless oil (1.16 g, 92% yield).
R*f* = 0.45 (cyclohexane). **IR** (ATR): ṽ = 2941, 2863, 2314, 2186, 1874, 1747, 1598, 1462, 1384, 1244, 1104, 1067, 880, 753, 685 cm^{-1}. **^{1}H NMR** (400 MHz, $CDCl_3$, 298 K): δ = 7.44–7.37 (m, 2H), 7.34–7.25 (m, 3H), 3.87 (t, J = 6.1 Hz, 2H), 2.56 (t, J = 7.1 Hz, 2H), 1.86 (quint, J = 6.5 Hz, 2H), 1.14–1.06 (m, 21H) ppm. **^{13}C{^{1}H} NMR** (101 MHz, $CDCl_3$, 298 K): δ = 131.7, 128.3, 127.6, 124.2, 90.2, 80.8, 62.0, 32.1, 18.2, 16.0, 12.1 ppm. **^{29}Si DEPT NMR** (79 MHz, $CDCl_3$, 298 K, optimized for J = 15.0 Hz): δ = 12.6 ppm. **HRMS** (APCI): calculated for $C_{20}H_{33}OSi^+$ $[M+H]^+$: 317.2295; Found 317.2295.

Triisopropyl((5-(*o*-tolyl)pent-4-yn-1-yl)oxy)silane (1b)

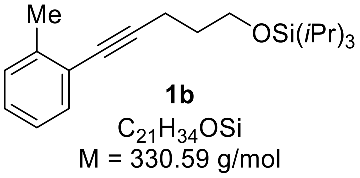

1b

$C_{21}H_{34}OSi$

M = 330.59 g/mol

Prepared according to **GP 1**. Flash column chromatography on silica gel using *n*-pentane as the eluent afforded substrate **1b** as a colorless oil (1.26 g, 95% yield).
R*f* = 0.40 (cyclohexane). **IR** (ATR): ṽ = 3061, 2941, 2863, 2558, 2111, 1797, 1599, 1460, 1382, 1246, 1104, 1066, 983, 753, 678 cm^{-1}. **^{1}H NMR** (500 MHz, $CDCl_3$, 298 K): δ = 7.39–7.33 (m, 1H), 7.20–7.15 (m, 2H), 7.15–7.07 (m, 1H), 3.86 (t, J = 6.1 Hz, 2H), 2.58 (t, J = 7.0 Hz, 2H), 2.42 (s, 3H), 1.86 (quint, J = 6.6 Hz, 2H), 1.14–1.03 (m, 21H) ppm. **^{13}C{^{1}H} NMR** (126 MHz, $CDCl_3$, 298 K): δ = 140.0, 131.9, 129.4, 127.6, 125.5, 124.0, 94.1, 79.6, 62.1, 32.4, 20.9, 18.2, 16.1, 12.2 ppm. **^{29}Si DEPT NMR** (79 MHz, $CDCl_3$, 298 K, optimized for J = 15.0 Hz): δ = 12.6 ppm. **HRMS** (APCI): calculated for $C_{21}H_{35}OSi^+$ $[M+H]^+$: 331.2452; Found 331.2452.

Triisopropyl((5-(*m*-tolyl)pent-4-yn-1-yl)oxy)silane (1c)

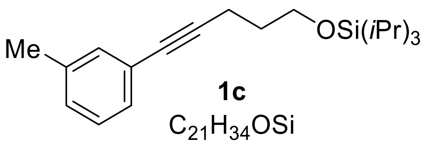

1c
$C_{21}H_{34}OSi$
M = 330.59 g/mol

Prepared according to **GP 1**. Flash column chromatography on silica gel using *n*-pentane as the eluent afforded substrate **1c** as a colorless oil (1.19 g, 90% yield).

R*f* = 0.50 (cyclohexane). **IR** (ATR): ṽ = 3036, 2941, 2863, 2341, 2230, 1870, 1602, 1461, 1383, 1245, 1104, 880, 781, 684 cm^{-1}. **^{1}H NMR** (400 MHz, $CDCl_3$, 298 K): *δ* = 7.24–7.14 (m, 3H), 7.11–7.05 (m, 1H), 3.84 (t, *J* = 6.1 Hz, 2H), 2.53 (t, *J* = 7.0 Hz, 2H), 2.32 (s, 3H), 1.84 (quint, *J* = 6.5 Hz, 2H), 1.15–1.04 (m, 21H) ppm. **^{13}C{^{1}H} NMR** (101 MHz, $CDCl_3$, 298 K): *δ* = 137.9, 132.3, 128.7, 128.5, 128.2, 124.0, 89.8, 80.9, 62.1, 32.2, 21.3, 18.2, 16.0, 12.2 ppm. **^{29}Si DEPT NMR** (79 MHz, $CDCl_3$, 298 K, optimized for *J* = 15.0 Hz): *δ* = 12.6 ppm. **HRMS** (APCI): calculated for $C_{21}H_{35}OSi^{+}$ $[M+H]^{+}$: 331.2452; Found 331.2449.

Triisopropyl((5-(*p*-tolyl)pent-4-yn-1-yl)oxy)silane (1d)

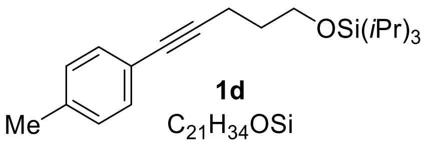

1d
$C_{21}H_{34}OSi$
M = 330.59 g/mol

Prepared according to **GP 1**. Flash column chromatography on silica gel using *n*-pentane as the eluent afforded substrate **1d** as a colorless oil (1.27 g, 96% yield).

R*f* = 0.36 (cyclohexane). **IR** (ATR): ṽ = 3026, 2940, 2863, 2727, 2296, 2020, 1898, 1509, 1461, 1383, 1245, 1104, 1067, 881, 814, 679 cm^{-1}. **^{1}H NMR** (400 MHz, $CDCl_3$, 298 K): *δ* = 7.30–7.26 (m, 2H), 7.11–7.06 (m, 2H), 3.84 (t, *J* = 6.1 Hz, 2H), 2.52 (t, *J* = 7.0 Hz, 2H), 2.33 (s, 3H), 1.87–1.78 (m, 2H), 1.12–1.02 (m, 21H) ppm. **^{13}C{^{1}H} NMR** (101 MHz, $CDCl_3$, 298 K): *δ* = 137.6, 131.5, 129.1, 121.1, 89.3, 80.8, 62.1, 32.2, 21.5, 18.2, 16.0, 12.2 ppm. **^{29}Si DEPT NMR** (79 MHz, $CDCl_3$, 298 K, optimized for *J* = 15.0 Hz): *δ* = 12.6 ppm. **HRMS** (APCI): calculated for $C_{21}H_{35}OSi^{+}$ $[M+H]^{+}$: 331.2452; Found 331.2453.

((5-(4-(*tert*-Butyl)phenyl)pent-4-yn-1-yl)oxy)triisopropylsilane (1e)

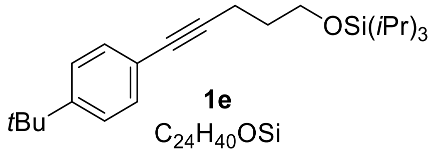

1e
$C_{24}H_{40}OSi$
M = 372.67 g/mol

Prepared according to **GP 1**. Flash column chromatography on silica gel using *n*-pentane as the eluent afforded substrate **1e** as a colorless oil (1.28 g, 86% yield).

R$_f$ = 0.45 (cyclohexane). **IR** (ATR): ṽ = 3034, 2942, 2864, 2722, 2267, 1953, 1657, 1502, 1461, 1363, 1267, 1105, 1067, 985, 881, 832, 721, 679 cm^{-1}. **^{1}H NMR** (400 MHz, $CDCl_3$, 298 K): δ = 7.36–7.27 (m, 4H), 3.84 (t, J = 6.0 Hz, 2H), 2.53 (t, J = 7.0 Hz, 2H), 1.83 (quint, J = 6.6 Hz, 2H), 1.31 (s, 9H), 1.15–1.04 (m, 21H) ppm. **^{13}C{^{1}H} NMR** (101 MHz, $CDCl_3$, 298 K): δ = 150.7, 131.3, 125.3, 121.2, 89.3, 80.8, 62.1, 34.8, 32.2, 31.3, 18.2, 16.0, 12.2 ppm. **^{29}Si DEPT NMR** (79 MHz, $CDCl_3$, 298 K, optimized for J = 15.0 Hz): δ = 12.6 ppm. **HRMS** (APCI): calculated for $C_{24}H_{41}OSi^+$ [M+H]$^+$: 373.2921; Found 373.2920.

((5-(3,5-Dimethylphenyl)pent-4-yn-1-yl)oxy)triisopropylsilane (1f)

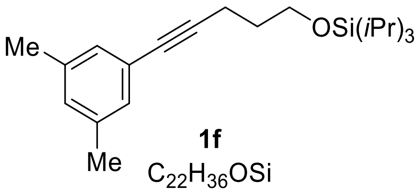

1f
$C_{22}H_{36}OSi$
M = 344.61 g/mol

Prepared according to **GP 1**. Flash column chromatography on silica gel using *n*-pentane as the eluent afforded substrate **1f** as a colorless oil (1.23 g, 89% yield).
R$_f$ = 0.49 (cyclohexane). **IR** (ATR): ṽ = 2940, 2863, 2728, 2478, 2084, 1892, 1737, 1598, 1461, 1382, 1246, 1104, 1065, 881, 847, 793, 684 cm^{-1}. **^{1}H NMR** (500 MHz, $CDCl_3$, 298 K): δ = 7.05–7.00 (m, 2H), 6.94–6.88 (m, 1H), 3.84 (t, J = 6.1 Hz, 2H), 2.52 (t, J = 7.1 Hz, 2H), 2.28 (s, 6H), 1.88–1.78 (m, 2H), 1.13–1.06 (m, 21H) ppm. **^{13}C{^{1}H} NMR** (126 MHz, $CDCl_3$, 298 K): δ = 137.8, 129.5, 129.4, 123.8, 89.4, 81.0, 62.1, 32.2, 21.2, 18.2, 16.0, 12.2 ppm. **^{29}Si DEPT NMR** (99 MHz, $CDCl_3$, 298 K, optimized for J = 15.0 Hz): δ = 12.6 ppm. **HRMS** (APCI): calculated for $C_{22}H_{37}OSi^+$ [M+H]$^+$: 345.2608; Found 345.2608.

((5-([1,1'-Biphenyl]-4-yl)pent-4-yn-1-yl)oxy)triisopropylsilane (1g)

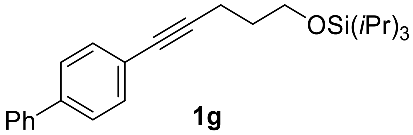

1g
$C_{26}H_{36}OSi$
M = 392.66 g/mol

Prepared according to **GP 1**. Flash column chromatography on silica gel using *n*-pentane as the eluent afforded substrate **1g** as a colorless oil (1.26 g, 80% yield).
R$_f$ = 0.38 (cyclohexane). **IR** (ATR): ṽ = 3030, 2940, 2863, 2725, 2371, 2283, 2079, 1799, 1670, 1599, 1485, 1461, 1383, 1104, 1067, 881, 838, 761, 678 cm^{-1}. **^{1}H NMR** (400 MHz, $CDCl_3$, 298 K): δ = 7.62–7.57 (m, 2H), 7.55–7.51 (m, 2H), 7.49–7.41 (m, 4H), 7.38–7.32 (m, 1H), 3.87 (t, J = 6.0 Hz, 2H), 2.57 (t, J = 7.0 Hz, 2H), 2.01–1.77 (m, 2H), 1.15–1.04 (m, 21H) ppm. **^{13}C{^{1}H} NMR** (101 MHz, $CDCl_3$, 298 K): δ = 140.7, 140.3, 132.1, 128.9, 127.6, 127.1, 127.0, 123.2, 90.9, 80.6, 62.0, 32.2, 18.2, 16.1, 12.2 ppm. **^{29}Si DEPT NMR** (79 MHz, $CDCl_3$, 298 K, optimized for J = 15.0 Hz): δ = 12.6 ppm. **HRMS** (APCI): calculated for

$C_{26}H_{37}OSi^+$ [M+H]$^+$: 393.2608; Found 393.2609.

Triisopropyl((5-(4-(trimethylsilyl)phenyl)pent-4-yn-1-yl)oxy)silane (1h)

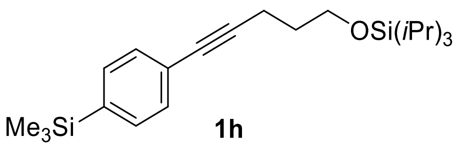

1h
$C_{23}H_{40}OSi_2$
M = 388.74 g/mol

Prepared according to **GP 1**. Flash column chromatography on silica gel using *n*-pentane as the eluent afforded substrate **1h** as a colorless oil (1.26 g, 81% yield).
R*f* = 0.50 (cyclohexane). **IR** (ATR): ṽ = 3064, 2942, 2864, 2562, 2280, 1939, 1595, 1462, 1248, 1106, 1067, 881, 838, 754, 679 cm^{-1}. **^{1}H NMR** (400 MHz, $CDCl_3$, 298 K): δ = 7.46–7.41 (m, 2H), 7.38–7.34 (m, 2H), 3.84 (t, *J* = 6.0 Hz, 2H), 2.54 (t, *J* = 7.0 Hz, 2H), 1.83 (quint, *J* = 6.5 Hz, 2H), 1.12–1.03 (m, 21H), 0.26 (s, 9H) ppm. **^{13}C{^{1}H} NMR** (101 MHz, $CDCl_3$, 298 K): δ = 140.1, 133.2, 130.8, 124.5, 90.6, 80.9, 62.0, 32.1, 18.2, 16.1, 12.1, −1.1 ppm. **^{29}Si DEPT NMR** (79 MHz, $CDCl_3$, 298 K, optimized for *J* = 15.0 Hz): δ = 12.6, −3.9 ppm. **HRMS** (APCI): calculated for $C_{23}H_{41}OSi_2^+$ [M+H]$^+$: 389.2690; Found 389.2688.

***tert*-Butyldimethyl(4-(5-((triisopropylsilyl)oxy)pent-1-yn-1-yl)phenoxy)silane (1i)**

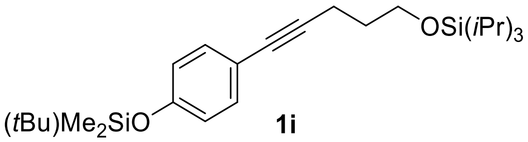

1i
$C_{26}H_{46}O_2Si_2$
M = 446.82 g/mol

Prepared according to **GP 1**. Flash column chromatography on silica gel using *n*-pentane as the eluent afforded substrate **1i** as a colorless oil (1.39 g, 78% yield).
R*f* = 0.52 (cyclohexane). **IR** (ATR): ṽ = 3039, 2930, 2862, 2303, 2171, 1887, 1602, 1505, 1462, 1252, 1104, 1067, 908, 836, 779, 676 cm^{-1}. **^{1}H NMR** (400 MHz, $CDCl_3$, 298 K): δ = 7.29–7.22 (m, 2H), 6.78–6.70 (m, 2H), 3.82 (t, *J* = 6.1 Hz, 2H), 2.50 (t, *J* = 7.0 Hz, 2H), 1.86–1.77 (m, 2H), 1.14–1.03 (m, 21H), 0.97 (s, 9H), 0.18 (s, 6H) ppm. **^{13}C{^{1}H} NMR** (101 MHz, $CDCl_3$, 298 K): δ = 155.4, 132.9, 120.2, 117.0, 88.7, 80.5, 62.1, 32.2, 25.8, 18.3, 18.2, 16.0, 12.1, −4.3 ppm. **^{29}Si DEPT NMR** (79 MHz, $CDCl_3$, 298 K, optimized for *J* = 15.0 Hz): δ = 21.4, 12.6 ppm. **HRMS** (APCI): calculated for $C_{26}H_{45}O_2Si_2^+$ [M−H]$^+$: 445.2953; Found 445.2950.

((5-(2-Chlorophenyl)pent-4-yn-1-yl)oxy)triisopropylsilane (1j)

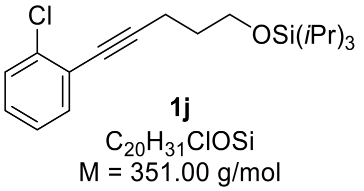

1j
$C_{20}H_{31}ClOSi$
M = 351.00 g/mol

Prepared according to **GP 1**. Flash column chromatography on silica gel using *n*-pentane as the eluent afforded substrate **1j** as a colorless oil (1.28 g, 91% yield).

R*f* = 0.37 (cyclohexane). **IR** (ATR): ṽ = 3068, 2941, 2863, 2235, 2115, 1799, 1471, 1245, 1104, 1065, 984, 881, 750, 678 cm^{-1}. **^{1}H NMR** (400 MHz, $CDCl_3$, 298 K): δ = 7.44–7.39 (m, 1H), 7.39–7.34 (m, 1H), 7.22–7.14 (m, 2H), 3.87 (t, J = 6.1 Hz, 2H), 2.60 (t, J = 7.0 Hz, 2H), 1.87 (quint, J = 6.5 Hz, 2H), 1.13–1.03 (m, 21H) ppm. **^{13}C{^{1}H} NMR** (101 MHz, $CDCl_3$, 298 K): δ = 135.9, 133.4, 129.2, 128.6, 126.4, 124.0, 95.9, 77.7, 62.0, 32.0, 18.2, 16.2, 12.2 ppm. **^{29}Si DEPT NMR** (79 MHz, $CDCl_3$, 298 K, optimized for J = 15.0 Hz): δ = 12.6 ppm. **HRMS** (APCI): calculated for $C_{20}H_{32}ClOSi^+$ $[M+H]^+$: 351.1905; Found 351.1906.

((5-(2-Bromophenyl)pent-4-yn-1-yl)oxy)triisopropylsilane (1k)

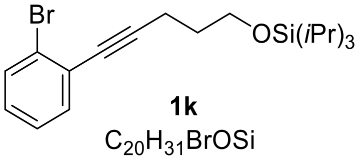

1k
$C_{20}H_{31}BrOSi$
M = 395.46 g/mol

Prepared according to **GP 1**. Flash column chromatography on silica gel using *n*-pentane as the eluent afforded substrate **1k** as a colorless oil (1.31 g, 83% yield).

R*f* = 0.41 (cyclohexane). **IR** (ATR): ṽ = 3065, 2940, 2863, 2233, 2122, 1913, 1800, 1467, 1246, 1103, 1065, 984, 880, 749, 678 cm^{-1}. **^{1}H NMR** (400 MHz, $CDCl_3$, 298 K): δ = 7.57–7.53 (m, 1H), 7.42 (dd, J = 7.7, 1.7 Hz, 1H), 7.22 (td, J = 7.6, 1.2 Hz, 1H), 7.11 (td, J = 7.8, 1.8 Hz, 1H), 3.88 (t, J = 6.1 Hz, 2H), 2.59 (t, J = 7.0 Hz, 2H), 1.87 (quint, J = 6.5 Hz, 2H), 1.11–1.04 (m, 21H) ppm. **^{13}C{^{1}H} NMR** (101 MHz, $CDCl_3$, 298 K): δ = 133.4, 132.4, 128.8, 127.0, 126.2, 125.6, 95.4, 79.5, 62.0, 32.0, 18.2, 16.2, 12.1 ppm. **^{29}Si DEPT NMR** (79 MHz, $CDCl_3$, 298 K, optimized for J = 15.0 Hz): δ = 12.6 ppm. **HRMS** (APCI): calculated for $C_{20}H_{32}BrOSi^+$ $[M+H]^+$: 395.1400; Found 395.1398.

((5-(3-Fluorophenyl)pent-4-yn-1-yl)oxy)triisopropylsilane (1l)

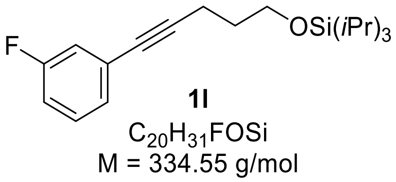

1l
$C_{20}H_{31}FOSi$
M = 334.55 g/mol

Prepared according to **GP 1**. Flash column chromatography on silica gel using *n*-pentane

as the eluent afforded substrate **1l** as a colorless oil (1.28 g, 96% yield).

R*f* = 0.50 (cyclohexane). **IR** (ATR): ṽ = 2941, 2864, 2231, 2103, 1929, 1699, 1609, 1463, 1278, 1104, 1067, 995, 880, 781, 678 cm^{-1}. **^{1}H NMR** (400 MHz, $CDCl_3$, 298 K): *δ* = 7.32–7.27 (m, 1H), 7.23–7.18 (m, 1H), 7.15–7.10 (m, 1H), 7.05–6.99 (m, 1H), 3.88 (t, *J* = 6.0 Hz, 2H), 2.58 (t, *J* = 7.0 Hz, 2H), 1.88 (quint, *J* = 6.5 Hz, 2H), 1.16–1.09 (m, 21H) ppm. **^{13}C{^{1}H} NMR** (101 MHz, $CDCl_3$, 298 K): *δ* = 162.5 (d, $J_{C,F}$ = 245.8 Hz), 129.8 (d, $J_{C,F}$ = 8.8 Hz), 127.5 (d, $J_{C,F}$ = 2.9 Hz), 126.1 (d, $J_{C,F}$ = 9.5 Hz), 118.5 (d, $J_{C,F}$ = 22.6 Hz), 114.9 (d, $J_{C,F}$ = 21.2 Hz), 91.4, 79.7 (d, $J_{C,F}$ = 3.4 Hz), 61.9, 32.0, 18.2, 16.0, 12.2 ppm. **^{19}F NMR** (471 MHz, $CDCl_3$, 298 K): *δ* = −113.5 ppm. **^{29}Si DEPT NMR** (79 MHz, $CDCl_3$, 298 K, optimized for *J* = 15.0 Hz): *δ* = 12.7 ppm. **HRMS** (APCI): calculated for $C_{20}H_{32}FOSi^+$ [M+H]$^+$: 335.2201; Found 335.2200.

((5-(3-Chlorophenyl)pent-4-yn-1-yl)oxy)triisopropylsilane (1m)

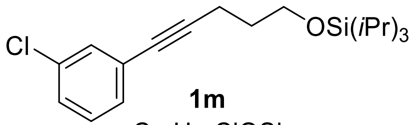

1m
$C_{20}H_{31}ClOSi$
M = 351.00 g/mol

Prepared according to **GP 1**. Flash column chromatography on silica gel using *n*-pentane as the eluent afforded substrate **1m** as a colorless oil (1.12 g, 80% yield).

R*f* = 0.41 (cyclohexane). **IR** (ATR): ṽ = 3067, 2941, 2864, 2237, 2103, 1931, 1679, 1591, 1461, 1244, 1105, 1067, 991, 879, 779, 679 cm^{-1}. **^{1}H NMR** (400 MHz, $CDCl_3$, 298 K): *δ* = 7.46–7.44 (m, 1H), 7.36–7.27 (m, 3H), 3.91 (t, *J* = 6.0 Hz, 2H), 2.61 (t, *J* = 7.0 Hz, 2H), 1.95–1.86 (m, 2H), 1.19–1.12 (m, 21H) ppm. **^{13}C{^{1}H} NMR** (101 MHz, $CDCl_3$, 298 K): *δ* = 134.1, 131.6, 129.8, 129.5, 127.9, 125.9, 91.7, 79.5, 61.9, 32.0, 18.2, 16.0, 12.2 ppm. **^{29}Si DEPT NMR** (79 MHz, $CDCl_3$, 298 K, optimized for *J* = 15.0 Hz): *δ* = 12.7 ppm. **HRMS** (APCI): calculated for $C_{20}H_{32}ClOSi^+$ [M+H]$^+$: 351.1905; Found 351.1902.

((5-(4-Fluorophenyl)pent-4-yn-1-yl)oxy)triisopropylsilane (1n)

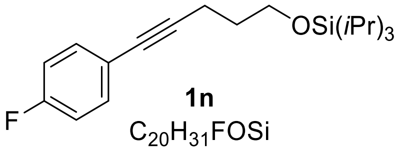

1n
$C_{20}H_{31}FOSi$
M = 334.55 g/mol

Prepared according to **GP 1**. Flash column chromatography on silica gel using *n*-pentane as the eluent afforded substrate **1n** as a colorless oil (1.22 g, 91% yield).

R*f* = 0.54 (cyclohexane). **IR** (ATR): ṽ = 3050, 2941, 2864, 2248, 2090, 1887, 1601, 1505, 1462, 1230, 1104, 1067, 984, 881, 833, 679 cm^{-1}. **^{1}H NMR** (400 MHz, $CDCl_3$, 298 K): *δ* = 7.38–7.31 (m, 2H), 7.02–6.91 (m, 2H), 3.83 (t, *J* = 6.0 Hz, 2H), 2.51 (t, *J* = 7.0 Hz, 2H),

1.82 (quint, J = 6.6 Hz, 2H), 1.12–1.04 (m, 21H) ppm. **^{13}C{^{1}H} NMR** (101 MHz, $CDCl_3$, 298 K): δ = 162.2 (d, $J_{C,F}$ = 248.1 Hz), 133.4 (d, $J_{C,F}$ = 8.3 Hz), 120.3 (d, $J_{C,F}$ = 3.6 Hz), 115.5 (d, $J_{C,F}$ = 21.8 Hz), 89.8, 79.7, 62.0, 32.1, 18.2, 15.9, 12.2 ppm. **^{19}F NMR** (471 MHz, $CDCl_3$, 298 K): δ = −112.5 ppm. **^{29}Si DEPT NMR** (79 MHz, $CDCl_3$, 298 K, optimized for J = 15.0 Hz): δ = 12.6 ppm. **HRMS** (APCI): calculated for $C_{20}H_{32}FOSi^+$ [M+H]$^+$: 335.2201; Found 335.2200.

((5-(4-Chlorophenyl)pent-4-yn-1-yl)oxy)triisopropylsilane (1o)

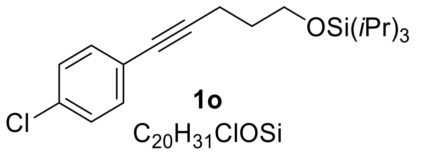

1o
$C_{20}H_{31}ClOSi$
M = 351.00 g/mol

Prepared according to **GP 1**. Flash column chromatography on silica gel using *n*-pentane as the eluent afforded substrate **1o** as a colorless oil (1.29 g, 92% yield).
R*f* = 0.39 (cyclohexane). **IR** (ATR): ṽ = 2940, 2863, 2229, 2012, 1895, 1591, 1488, 1462, 1384, 1092, 983, 880, 825, 678 cm^{-1}. **^{1}H NMR** (400 MHz, $CDCl_3$, 298 K): δ = 7.35–7.30 (m, 2H), 7.29–7.24 (m, 2H), 3.85 (t, J = 6.0 Hz, 2H), 2.55 (t, J = 7.0 Hz, 2H), 1.85 (quint, J = 6.5 Hz, 2H), 1.14–1.06 (m, 21H) ppm. **^{13}C{^{1}H} NMR** (101 MHz, $CDCl_3$, 298 K): δ = 133.5, 132.9, 128.6, 122.7, 91.3, 79.7, 62.0, 32.0, 18.2, 16.0, 12.2 ppm. **^{29}Si DEPT NMR** (79 MHz, $CDCl_3$, 298 K, optimized for J = 15.0 Hz): δ = 12.6 ppm. **HRMS** (APCI): calculated for $C_{20}H_{32}ClOSi^+$ [M+H]$^+$: 351.1905; Found 351.1903.

((5-(4-Bromophenyl)pent-4-yn-1-yl)oxy)triisopropylsilane (1p)

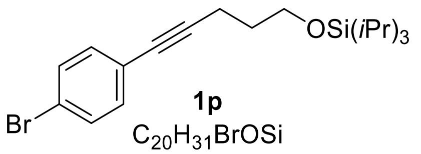

1p
$C_{20}H_{31}BrOSi$
M = 395.46 g/mol

Prepared according to **GP 1**. Flash column chromatography on silica gel using *n*-pentane as the eluent afforded substrate **1p** as a colorless oil (1.39 g, 88% yield).
R*f* = 0.44 (cyclohexane). **IR** (ATR): ṽ = 2940, 2863, 2229, 2010, 1895, 1586, 1484, 1461, 1389, 1104, 1068, 983, 880, 821, 678 cm^{-1}. **^{1}H NMR** (400 MHz, $CDCl_3$, 298 K): δ = 7.43–7.38 (m, 2H), 7.25–7.21 (m, 2H), 3.82 (t, J = 6.0 Hz, 2H), 2.51 (t, J = 7.0 Hz, 2H), 1.82 (quint, J = 6.6 Hz, 2H), 1.13–1.02 (m, 21H) ppm. **^{13}C{^{1}H} NMR** (101 MHz, $CDCl_3$, 298 K): δ = 133.1, 131.5, 123.2, 121.7, 91.5, 79.8, 62.0, 32.0, 18.2, 16.0, 12.2 ppm. **^{29}Si DEPT NMR** (79 MHz, $CDCl_3$, 298 K, optimized for J = 15.0 Hz): δ = 12.7 ppm. **HRMS** (APCI): calculated for $C_{20}H_{32}BrOSi^+$ [M+H]$^+$: 395.1400; Found 351.1396.

Triisopropyl((5-(naphthalen-2-yl)pent-4-yn-1-yl)oxy)silane (1q)

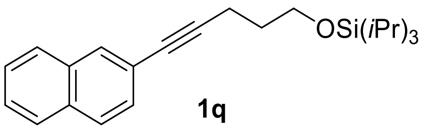

1q
$C_{24}H_{34}OSi$
M = 366.62 g/mol

Prepared according to **GP 1**. Flash column chromatography on silica gel using *n*-pentane as the eluent afforded substrate **1q** as a colorless oil (1.39 g, 95% yield).

R*f* = 0.52 (cyclohexane). **IR** (ATR): ṽ = 3056, 2940, 2863, 2228, 2078, 1904, 1596, 1461, 1245, 1103, 1066, 992, 881, 742, 678 cm^{-1}. **^{1}H NMR** (400 MHz, $CDCl_3$, 298 K): δ = 7.93–7.89 (m, 1H), 7.83–7.73 (m, 3H), 7.52–7.42 (m, 3H), 3.89 (t, *J* = 6.0 Hz, 2H), 2.60 (t, *J* = 7.0 Hz, 2H), 1.89 (quint, *J* = 6.5 Hz, 2H), 1.15–1.07 (m, 21H) ppm. **^{13}C{^{1}H} NMR** (101 MHz, $CDCl_3$, 298 K): δ = 133.2, 132.6, 131.2, 128.9, 127.9, 127.8, 127.7, 126.5, 126.4, 121.6, 90.6, 81.1, 62.1, 32.2, 18.2, 16.1, 12.2 ppm. **^{29}Si DEPT NMR** (79 MHz, $CDCl_3$, 298 K, optimized for *J* = 15.0 Hz): δ = 12.6 ppm. **HRMS** (APCI): calculated for $C_{24}H_{35}OSi^{+}$ $[M+H]^{+}$: 367.2452; Found 367.2453.

((5-(Benzo[*b*]thiophen-2-yl)pent-4-yn-1-yl)oxy)triisopropylsilane (1r)

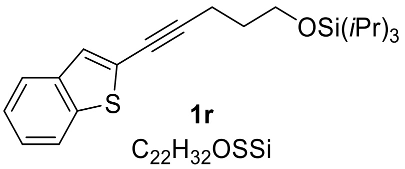

1r
$C_{22}H_{32}OSSi$
M = 372.64 g/mol

Prepared according to **GP 1**. Flash column chromatography on silica gel using *n*-pentane as the eluent afforded substrate **1r** as a colorless oil (1.36 g, 91% yield).

R*f* = 0.50 (cyclohexane). **IR** (ATR): ṽ = 3057, 2940, 2863, 2223, 2070, 1933, 1559, 1459, 1433, 1103, 1067, 974, 880, 742, 678 cm^{-1}. **^{1}H NMR** (400 MHz, $CDCl_3$, 298 K): δ = 7.76–7.67 (m, 2H), 7.38–7.30 (m, 3H), 3.84 (t, *J* = 6.0 Hz, 2H), 2.60 (t, *J* = 7.0 Hz, 2H), 1.86 (quint, *J* = 6.6 Hz, 2H), 1.12–1.04 (m, 21H) ppm. **^{13}C{^{1}H} NMR** (101 MHz, $CDCl_3$, 298 K): δ = 139.9, 139.3, 127.7, 125.1, 124.7, 124.3, 123.6, 122.0, 96.5, 74.4, 61.9, 31.8, 18.2, 16.4, 12.1 ppm. **^{29}Si DEPT NMR** (79 MHz, $CDCl_3$, 298 K, optimized for *J* = 15.0 Hz): δ = 12.8 ppm. **HRMS** (APCI): calculated for $C_{22}H_{33}OSSi^{+}$ $[M+H]^{+}$: 373.2016; Found 373.2015.

Triisopropyl((5-(thiophen-2-yl)pent-4-yn-1-yl)oxy)silane (1s)

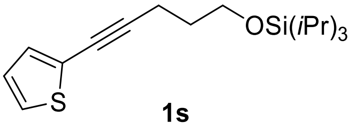

1s
$C_{18}H_{30}OSSi$
M = 322.58 g/mol

Prepared according to **GP 1**. Flash column chromatography on silica gel using *n*-pentane

as the eluent afforded substrate **1s** as a colorless oil (1.19 g, 92% yield).

R*f* = 0.50 (cyclohexane). **IR** (ATR): ṽ = 2940, 2863, 2303, 2106, 2017, 1787, 1518, 1461, 1384, 1188, 1104, 1066, 976, 881, 723, 679 cm^{-1}. **^{1}H NMR** (400 MHz, $CDCl_3$, 298 K): *δ* = 7.19–7.14 (m, 1H), 7.13–7.08 (m, 1H), 6.95–6.90 (m, 1H), 3.83 (t, *J* = 6.0 Hz, 2H), 2.55 (t, *J* = 7.0 Hz, 2H), 1.83 (quint, *J* = 6.5 Hz, 2H), 1.12–1.04 (m, 21H) ppm. **^{13}C{^{1}H} NMR** (101 MHz, $CDCl_3$, 298 K): *δ* = 131.0, 126.9, 126.0, 124.4, 94.3, 73.9, 61.9, 31.9, 18.2, 16.3, 12.1 ppm. **^{29}Si DEPT NMR** (79 MHz, $CDCl_3$, 298 K, optimized for *J* = 15.0 Hz): *δ* = 12.7 ppm. **HRMS** (APCI): calculated for $C_{18}H_{31}OSSi^+$ $[M+H]^+$: 323.1859; Found 323.1858.

Diisopropyl(methyl)((5-phenylpent-4-yn-1-yl)oxy)silane (1u)

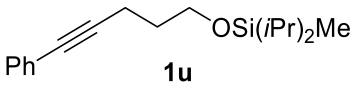

1u

$C_{18}H_{28}OSi$

M = 288.51 g/mol

Prepared according to **GP 2**. Flash column chromatography on silica gel using *n*-pentane as the eluent afforded substrate **1u** as a colorless oil (957.6 mg, 83% yield).

R*f* = 0.56 (cyclohexane). **IR** (ATR): ṽ = 3057, 2940, 2863, 2561, 2241, 1943, 1873, 1665, 1598, 1461, 1251, 1102, 1068, 993, 880, 752, 689 cm^{-1}. **^{1}H NMR** (400 MHz, $CDCl_3$, 298 K): *δ* = 7.42–7.37 (m, 2H), 7.32–7.24 (m, 3H), 3.79 (t, *J* = 6.1 Hz, 2H), 2.51 (t, *J* = 7.0 Hz, 2H), 1.82 (quint, *J* = 6.6 Hz, 2H), 1.07–0.88 (m, 14H), 0.05 (s, 3H) ppm. **^{13}C{^{1}H} NMR** (101 MHz, $CDCl_3$, 298 K): *δ* = 131.7, 128.3, 127.6, 124.2, 90.0, 80.8, 61.9, 32.0, 17.6, 17.5, 16.0, 13.1, −8.6 ppm. **^{29}Si DEPT NMR** (79 MHz, $CDCl_3$, 298 K, optimized for *J* = 15.0 Hz): *δ* = 18.3 ppm. **HRMS** (APCI): calculated for $C_{18}H_{29}OSi^+$ $[M+H]^+$: 289.1982; Found 289.1981.

***tert*-Butyldimethyl((5-phenylpent-4-yn-1-yl)oxy)silane (1v)**

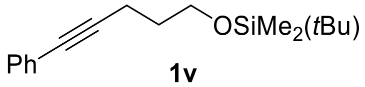

1v

$C_{17}H_{26}OSi$

M = 274.48 g/mol

Prepared according to **GP 2**. Flash column chromatography on silica gel using *n*-pentane as the eluent afforded substrate **1v** as a colorless oil (933.2 mg, 85% yield).

R*f* = 0.52 (cyclohexane). **IR** (ATR): ṽ = 2951, 2928, 2855, 2333, 2112, 1942, 1800, 1598, 1469, 1252, 1101, 1068, 981, 832, 774, 753, 689 cm^{-1}. **^{1}H NMR** (400 MHz, $CDCl_3$, 298 K): *δ* = 7.38–7.31 (m, 2H), 7.26–7.19 (m, 3H), 3.72 (t, *J* = 6.0 Hz, 2H), 2.45 (t, *J* = 7.0 Hz, 2H), 1.76 (quint, *J* = 6.5 Hz, 2H), 0.87 (s, 9H), 0.04 (s, 6H) ppm. **^{13}C{^{1}H} NMR** (101 MHz, $CDCl_3$, 298 K): *δ* = 131.7, 128.3, 127.6, 124.2, 90.0, 80.8, 61.8, 31.9, 26.1, 18.5, 16.0, −5.2 ppm. **^{29}Si DEPT NMR** (79 MHz, $CDCl_3$, 298 K, optimized for *J* = 15.0 Hz): *δ* = 19.1 ppm. **HRMS** (APCI): calculated for $C_{17}H_{27}OSi^+$ $[M+H]^+$: 275.1826; Found 275.1826.

(2,3-Dimethylbutan-2-yl)dimethyl((5-phenylpent-4-yn-1-yl)oxy)silane (1w)

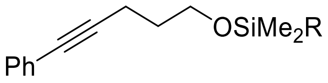

R = 2,3-dimethyl-2-butyl
1w
$C_{19}H_{30}OSi$
M = 302.53 g/mol

Prepared according to **GP 2**. Flash column chromatography on silica gel using *n*-pentane as the eluent afforded substrate **1w** as a colorless oil (980.2 mg, 81% yield).

R*f* = 0.42 (cyclohexane). **IR** (ATR): ṽ = 2952, 2865, 2601, 2248, 1946, 1747, 1598, 1464, 1250, 1100, 980, 825, 774, 753, 689 cm^{-1}. **^{1}H NMR** (400 MHz, $CDCl_3$, 298 K): δ = 7.38–7.31 (m, 2H), 7.27–7.19 (m, 3H), 3.69 (t, *J* = 6.0 Hz, 2H), 2.45 (t, *J* = 7.0 Hz, 2H), 1.75 (quint, *J* = 6.5 Hz, 2H), 1.59 (hept, *J* = 6.8 Hz, 1H), 0.85 (d, *J* = 6.8 Hz, 6H), 0.82 (s, 6H), 0.07 (s, 6H) ppm. **^{13}C{^{1}H} NMR** (101 MHz, $CDCl_3$, 298 K): δ = 131.7, 128.3, 127.6, 124.2, 90.1, 80.8, 61.5, 34.4, 31.9, 25.3, 20.5, 18.7, 16.1, −3.3 ppm. **^{29}Si DEPT NMR** (79 MHz, $CDCl_3$, 298 K, optimized for *J* = 15.0 Hz): δ = 19.4 ppm. **HRMS** (APCI): calculated for $C_{19}H_{31}OSi^+$ $[M+H]^+$: 303.2139; Found 303.2139.

3.4 Synthesis of Allenylsilanes 2 (GP 3)

Allenylsilanes **2a**–**2d**, **2g**–**2i**, and **2m** were already prepared and characterized in our previous work on the (3+2) annulation of allenylsilanes with internal alkynes.[S8] The additional allenylsilanes were synthesized according to the following procedure.

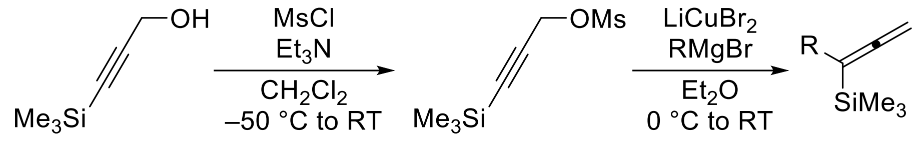

S6 **S7** **2**

Step 1: According to a reported procedure,[S9] 3-(trimethylsilyl)prop-2-yn-1-ol (**S6**, 1.28 g, 10.0 mmol, 1.0 equiv) and Et_3N (15.0 mmol, 1.52 g, 1.5 equiv) are dissolved in CH_2Cl_2 (60 mL) and cooled to −50 °C. Then, mesyl chloride (12.0 mmol, 1.37 g, 1.2 equiv) is added dropwise to this solution. After stirring for 30 min, the resulting mixture is gradually warmed to room temperature and stirred for additional 2 h. Upon completion, the reaction mixture is poured into water (50 mL), and the organic layer is separated. The aqueous phase is extracted with CH_2Cl_2 (3 × 20 mL). The combined organic phases are dried over Na_2SO_4 and concentrated under reduced pressure to afford mesylate **S7** as a light yellow oil, which is directly used in the next step without further purification.

Step 2: A solution of CuBr (12.0 mmol, 1.72 g, 1.2 equiv) and LiBr (12.0 mmol, 1.04 g, 1.2 equiv) in Et_2O (20 mL) is cooled to 0 °C. Then, freshly prepared RMgBr (12.0 mmol, 1.2 equiv) is slowly added to this mixture. After stirring at 0 °C for 30 min, a solution of mesylate **S7** in Et_2O (5.0 mL) is added, and the resulting mixture is gradually warmed to room temperature and stirred for additional 12 h. Upon completion, the reaction mixture is quenched by the addition of saturated NH_4Cl solution (20 mL), and the aqueous phase is extracted with Et_2O (3 × 15 mL). The combined organic phases are dried over Na_2SO_4 and carefully concentrated under reduced pressure (40 °C, 750 mbar). Purification of the residue by flash column chromatography on silica gel using *n*-pentane as the eluent affords the corresponding allenylsilane **2** as a colorless oil in analytically pure form.

3.5 Characterization Data for Allenylsilanes 2

Trimethyl(5-methylhexa-1,2-dien-3-yl)silane (2j)

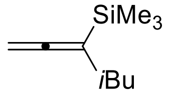

2j
$C_{10}H_{20}Si$
M = 168.36 g/mol

Prepared from *i*BuMgBr according to **GP 3**. Flash column chromatography on silica gel using *n*-pentane as the eluent afforded substrate **2j** as a colorless oil (1.24 g, 74% yield).

R*f* = 0.80 (cyclohexane, stained with $KMnO_4$). **IR** (ATR): ṽ = 2953, 2869, 2371, 2159, 1924, 1625, 1464, 1247, 1010, 834, 806, 752, 691 cm^{-1}. **^{1}H NMR** (500 MHz, $CDCl_3$, 298 K): δ = 4.30 (t, *J* = 2.9 Hz, 2H), 1.85–1.81 (m, 2H), 1.80–1.72 (m, 1H), 0.91 (d, *J* = 6.4 Hz, 6H), 0.09 (s, 9H) ppm. **^{13}C{^{1}H} NMR** (126 MHz, $CDCl_3$, 298 K): δ = 208.8, 93.5, 68.3, 38.6, 28.1, 22.7, −1.5 ppm. **^{29}Si DEPT NMR** (99 MHz, $CDCl_3$, 298 K, optimized for *J* = 7.0 Hz): δ = −9.9 ppm.

Trimethyl(1-phenylbuta-2,3-dien-2-yl)silane (2k)

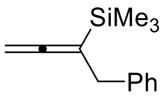

2k
$C_{13}H_{18}Si$
M = 202.37 g/mol

Prepared from $PhCH_2MgBr$ according to **GP 3**. Flash column chromatography on silica gel using *n*-pentane as the eluent afforded substrate **2k** as a colorless oil (1.42 g, 70% yield). **R*f*** = 0.78 (cyclohexane, stained with $KMnO_4$). **^{1}H NMR** (500 MHz, $CDCl_3$, 298 K): δ = 7.30–7.26 (m, 2H), 7.22–7.16 (m, 3H), 4.33 (t, *J* = 2.7 Hz, 2H), 3.35 (t, *J* = 2.7 Hz, 2H), 0.03 (s, 9H) ppm. **^{13}C{^{1}H} NMR** (126 MHz, $CDCl_3$, 298 K): δ = 210.0, 140.7, 129.1, 128.2, 126.1, 94.6, 69.0, 36.4, −1.4 ppm. **^{29}Si DEPT NMR** (99 MHz, $CDCl_3$, 298 K, optimized for *J* = 7.0 Hz): δ = −4.0 ppm. The NMR spectroscopic data are in accordance with those reported.[S10]

Trimethyl(4-methylpenta-1,2-dien-3-yl)silane (2l)

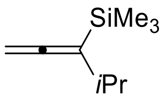

2l
$C_9H_{18}Si$
M = 154.33 g/mol

Prepared from *i*PrMgBr according to **GP 3**. Flash column chromatography on silica gel using *n*-pentane as the eluent afforded substrate **2l** as a colorless oil (1.17 g, 76% yield). **R*f*** = 0.82 (cyclohexane, stained with $KMnO_4$). **IR** (ATR): ṽ = 2952, 2866, 2485, 2098, 1922, 1617, 1475, 1247, 1001, 834, 803, 756, 690 cm^{-1}. **^{1}H NMR** (500 MHz, $CDCl_3$, 298 K): δ = 4.37 (d, *J* = 2.1 Hz, 2H), 2.25–2.14 (m, 1H), 1.05 (d, *J* = 6.7 Hz, 6H), 0.11 (s, 9H) ppm. **^{13}C{^{1}H} NMR** (101 MHz, $CDCl_3$, 298 K): δ = 207.8, 101.7, 70.2, 28.3, 23.5, −1.0 ppm. **^{29}Si DEPT NMR** (79 MHz, $CDCl_3$, 298 K, optimized for *J* = 7.0 Hz): δ = −5.3 ppm.

4 Experimental Details for the Silylium-Ion-Promoted Ionic Remote *α*-C–H Allenylation of Silyl Ethers

4.1 General Procedure for the *α*-C–H Allenylation of Silyl Ethers (GP 4)

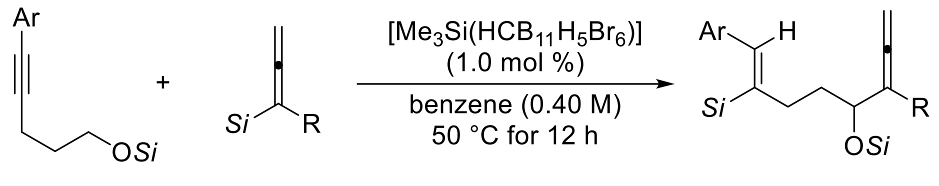

In an argon-filled glovebox, silyl ether **1** (0.20 mmol, 1.0 equiv) and allenylsilane **2** (0.30 mmol, 1.5 equiv) are dissolved in benzene (0.5 mL). After stirring for 1 min, silylium carborate [$Me_3Si(HCB_{11}H_5Br_6)$] (1.4 mg, 2.0 µmol, 1.0 mol %) is added, and the resulting reaction mixture is stirred for additional 12 h at 50 °C. Upon completion of the reaction, the reaction mixture is removed from the glovebox, and all volatiles are evaporated under reduced pressure. CH_2Br_2 (34.8 mg, 0.20 mmol, 1.0 equiv) is subsequently added as an internal standard, and C_6D_6 (0.5 mL) is used as the NMR solvent to determine the yield by ^{1}H NMR spectroscopy. Purification by flash column chromatography on silica gel using *n*-pentane as the eluent affords the *α*-C–H allenylation product **3** in analytically pure form.

4.2 Characterization Data for *α*-C–H Allenylation Products 3

(*Z*)-Triisopropyl((3-methyl-8-phenyl-7-(trimethylsilyl)octa-1,2,7-trien-4-yl)oxy)silane (3aa)

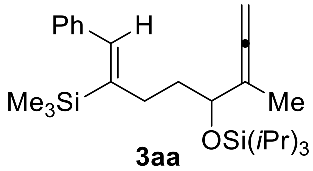

3aa

$C_{27}H_{46}OSi_2$
M = 442.83 g/mol

Prepared from **1a** (63.3 mg, 0.20 mmol) and **2a** (37.9 mg, 0.30 mmol) according to **GP 4**. Flash column chromatography on silica gel using *n*-pentane as the eluent afforded product **3aa** as a colorless oil (69.1 mg, 78% yield).

R*f* = 0.66 (cyclohexane). **IR** (ATR): ṽ = 3053, 2941, 2864, 2285, 2105, 1958, 1804, 1591, 1461, 1247, 1059, 834, 747, 680 cm^{-1}. **^{1}H NMR** (500 MHz, $CDCl_3$, 298 K): δ = 7.36–7.30 (m, 2H), 7.30–7.25 (m, 1H), 7.25–7.20 (m, 3H), 4.74–4.61 (m, 2H), 4.43–4.36 (m, 1H), 2.34–2.20 (m, 2H), 1.89–1.78 (m, 2H), 1.76 (t, *J* = 3.2 Hz, 3H), 1.20–1.08 (m, 21H), 0.01 (s, 9H) ppm. **^{13}C{^{1}H} NMR** (101 MHz, $CDCl_3$, 298 K): δ = 206.6, 144.5, 142.0, 140.8, 128.7, 127.9, 126.8, 100.4, 74.7, 74.1, 37.2, 35.1, 18.3, 18.2, 12.5, 12.2, 0.6 ppm. **^{29}Si DEPT**

NMR (79 MHz, $CDCl_3$, 298 K, optimized for J = 15.0 Hz): δ = 12.5, −6.6 ppm. **HRMS** (ESI): calculated for $C_{27}H_{47}OSi_2^+$ [M+H]$^+$: 443.3160; Found 443.3158.

(*Z*)-Triisopropyl((3-methyl-8-(*o*-tolyl)-7-(trimethylsilyl)octa-1,2,7-trien-4-yl)oxy)silane (3ba)

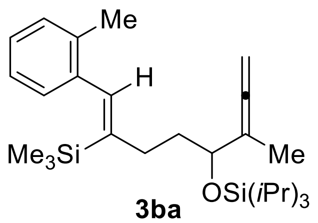

3ba
$C_{28}H_{48}OSi_2$
M = 456.86 g/mol

Prepared from **1b** (66.1 mg, 0.20 mmol) and **2a** (37.9 mg, 0.30 mmol) according to **GP 4**. Flash column chromatography on silica gel using *n*-pentane as the eluent afforded product **3ba** as a colorless oil (64.1 mg, 70% yield).

R*f* = 0.61 (cyclohexane). **IR** (ATR): ṽ = 3059, 2942, 2864, 2284, 2086, 1958, 1694, 1596, 1460, 1246, 1059, 835, 745, 679 cm^{-1}. **^{1}H NMR** (500 MHz, $CDCl_3$, 298 K): δ = 7.19–7.13 (m, 1H), 7.13–7.06 (m, 4H), 4.69–4.56 (m, 2H), 4.39–4.32 (m, 1H), 2.26–2.17 (m, 5H), 1.86–1.73 (m, 2H), 1.71 (t, J = 3.1 Hz, 3H), 1.14–1.04 (m, 21H), −0.13 (s, 9H) ppm. **^{13}C{^{1}H} NMR** (126 MHz, $CDCl_3$, 298 K): δ = 206.6, 143.9, 141.5, 140.3, 136.1, 129.6, 129.3, 127.2, 125.2, 100.5, 74.7, 74.1, 37.4, 34.5, 20.1, 18.3, 18.2, 12.5, 12.3, 0.3 ppm. **^{29}Si DEPT NMR** (99 MHz, $CDCl_3$, 298 K, optimized for J = 15.0 Hz): δ = 12.5, −6.4 ppm. **HRMS** (ESI): calculated for $C_{28}H_{49}OSi_2^+$ [M+H]$^+$: 457.3316; Found 457.3313.

(*Z*)-Triisopropyl((3-methyl-8-(*m*-tolyl)-7-(trimethylsilyl)octa-1,2,7-trien-4-yl)oxy)-silane (3ca)

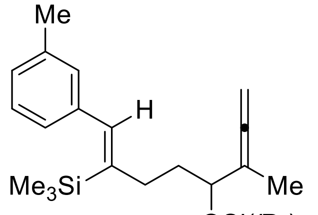

3ca
$C_{28}H_{48}OSi_2$
M = 456.86 g/mol

Prepared from **1c** (66.1 mg, 0.20 mmol) and **2a** (37.9 mg, 0.30 mmol) according to **GP 4**. Flash column chromatography on silica gel using *n*-pentane as the eluent afforded product **3ca** as a colorless oil (73.4 mg, 80% yield).

R*f* = 0.60 (cyclohexane). **IR** (ATR): ṽ = 2941, 2864, 2482, 2280, 2087, 1958, 1694, 1594, 1461, 1246, 1059, 881, 834, 755, 679 cm^{-1}. **^{1}H NMR** (400 MHz, $CDCl_3$, 298 K): δ = 7.20–

7.13 (m, 2H), 7.07–7.02 (m, 1H), 7.02–6.95 (m, 2H), 4.68–4.56 (m, 2H), 4.37–4.30 (m, 1H), 2.34 (s, 3H), 2.26–2.14 (m, 2H), 1.83–1.72 (m, 2H), 1.70 (t, *J* = 3.1 Hz, 3H) 1.14–1.03 (m, 21H), −0.03 (s, 9H) ppm. **^{13}C{^{1}H} NMR** (101 MHz, $CDCl_3$, 298 K): *δ* = 206.7, 144.2, 142.2, 140.7, 137.3, 129.6, 127.7, 127.5, 125.7, 100.5, 74.7, 74.0, 37.3, 35.1, 21.5, 18.3, 18.2, 12.6, 12.3, 0.7 ppm. **^{29}Si DEPT NMR** (79 MHz, $CDCl_3$, 298 K, optimized for *J* = 15.0 Hz): *δ* = 12.4, −6.7 ppm. **HRMS** (ESI): calculated for $C_{28}H_{49}OSi_2^+$ [M+H]$^+$: 457.3316; Found 457.3313.

(*Z*)-Triisopropyl((3-methyl-8-(*p*-tolyl)-7-(trimethylsilyl)octa-1,2,7-trien-4-yl)oxy)silane (3da)

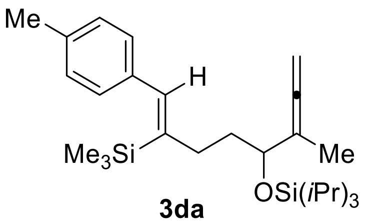

3da
$C_{28}H_{48}OSi_2$
M = 456.86 g/mol

Prepared from **1d** (66.1 mg, 0.20 mmol) and **2a** (37.9 mg, 0.30 mmol) according to **GP 4**. Flash column chromatography on silica gel using *n*-pentane as the eluent afforded product **3da** as a colorless oil (76.9 mg, 84% yield).

R*f* = 0.50 (cyclohexane). **IR** (ATR): ṽ = 2941, 2864, 2580, 2300, 1958, 1507, 1461, 1369, 1247, 1059, 881, 836, 679 cm^{-1}. **^{1}H NMR** (400 MHz, $CDCl_3$, 298 K): *δ* = 7.15 (s, 1H), 7.11–7.05 (m, 4H), 4.68–4.57 (m, 2H), 4.37–4.31 (m, 1H), 2.35 (s, 3H), 2.26–2.15 (m, 2H), 1.83–1.72 (m, 2H), 1.71 (t, *J* = 3.1 Hz, 3H), 1.15–1.01 (m, 21H), −0.03 (s, 9H) ppm. **^{13}C{^{1}H} NMR** (101 MHz, $CDCl_3$, 298 K): *δ* = 206.7, 143.8, 142.1, 137.9, 136.4, 128.6, 128.5, 100.5, 74.7, 74.0, 37.3, 35.2, 21.3, 18.3, 18.2, 12.6, 12.2, 0.7 ppm. **^{29}Si DEPT NMR** (79 MHz, $CDCl_3$, 298 K, optimized for *J* = 15.0 Hz): *δ* = 12.4, −6.7 ppm. **HRMS** (ESI): calculated for $C_{28}H_{49}OSi_2^+$ [M+H]$^+$: 457.3316; Found 457.3314.

(*Z*)-((8-(4-(*tert*-Butyl)phenyl)-3-methyl-7-(trimethylsilyl)octa-1,2,7-trien-4-yl)oxy)-triisopropylsilane (3ea)

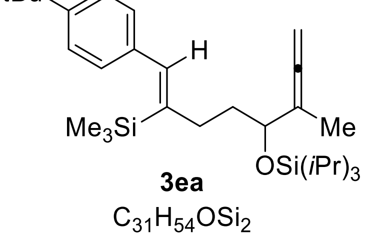

3ea
$C_{31}H_{54}OSi_2$
M = 498.94 g/mol

Prepared from **1e** (74.5 mg, 0.20 mmol) and **2a** (37.9 mg, 0.30 mmol) according to **GP 4**.

Flash column chromatography on silica gel using *n*-pentane as the eluent afforded product **3ea** as a colorless oil (67.0 mg, 67% yield).

R$_f$ = 0.58 (cyclohexane). **IR** (ATR): ṽ = 2943, 2864, 2308, 2085, 1958, 1506, 1461, 1364, 1247, 1059, 881, 835, 680 cm^{-1}. **^{1}H NMR** (400 MHz, $CDCl_3$, 298 K): δ = 7.33–7.27 (m, 2H), 7.15 (s, 1H), 7.14–7.08 (m, 2H), 4.68–4.56 (m, 2H), 4.37–4.30 (m, 1H), 2.26–2.14 (m, 2H), 1.83–1.72 (m, 2H), 1.70 (t, *J* = 3.1 Hz, 3H), 1.33 (s, 9H), 1.14–1.02 (m, 21H), −0.03 (s, 9H) ppm. **^{13}C{^{1}H} NMR** (101 MHz, $CDCl_3$, 298 K): δ = 206.6, 149.8, 143.8, 142.1, 137.8, 128.4, 124.7, 100.5, 74.7, 74.0, 37.3, 35.2, 34.6, 31.5, 18.3, 18.2, 12.5, 12.2, 0.6 ppm. **^{29}Si DEPT NMR** (79 MHz, $CDCl_3$, 298 K, optimized for *J* = 15.0 Hz): δ = 12.5, −6.7 ppm. **HRMS** (ESI): calculated for $C_{31}H_{55}OSi_2^+$ [M+H]$^+$: 499.3786; Found 499.3788.

(*Z*)-((8-(3,5-Dimethylphenyl)-3-methyl-7-(trimethylsilyl)octa-1,2,7-trien-4-yl)oxy)-triisopropylsilane (3fa)

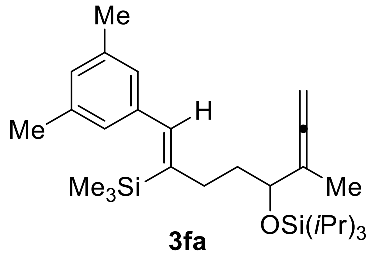

3fa
$C_{29}H_{50}OSi_2$
M = 470.89 g/mol

Prepared from **1f** (68.9 mg, 0.20 mmol) and **2a** (37.9 mg, 0.30 mmol) according to **GP 4**. Flash column chromatography on silica gel using *n*-pentane as the eluent afforded product **3fa** as a colorless oil (72.9 mg, 77% yield).

R$_f$ = 0.65 (cyclohexane). **IR** (ATR): ṽ = 2941, 2864, 2277, 2093, 1958, 1598, 1461, 1370, 1246, 1059, 881, 834, 757, 679 cm^{-1}. **^{1}H NMR** (400 MHz, $CDCl_3$, 298 K): δ = 7.13 (s, 1H), 6.87 (s, 1H), 6.81 (s, 2H), 4.69–4.57 (m, 2H), 4.37–4.31 (m, 1H), 2.30 (s, 6H), 2.24–2.16 (m, 2H), 1.82–1.72 (m, 2H), 1.71 (t, *J* = 3.1 Hz, 3H), 1.14–1.04 (m, 21H), −0.02 (s, 9H) ppm. **^{13}C{^{1}H} NMR** (101 MHz, $CDCl_3$, 298 K): δ = 206.7, 143.8, 142.3, 140.6, 137.2, 128.3, 126.6, 100.5, 74.7, 74.0, 37.3, 35.1, 21.4, 18.3, 18.2, 12.6, 12.2, 0.7 ppm. **^{29}Si DEPT NMR** (79 MHz, $CDCl_3$, 298 K, optimized for *J* = 15.0 Hz): δ = 12.4, −6.8 ppm. **HRMS** (ESI): calculated for $C_{29}H_{51}OSi_2^+$ [M+H]$^+$: 471.3473; Found 471.3467.

(*Z*)-((8-([1,1'-Biphenyl]-4-yl)-3-methyl-7-(trimethylsilyl)octa-1,2,7-trien-4-yl)oxy)-triisopropylsilane (3ga)

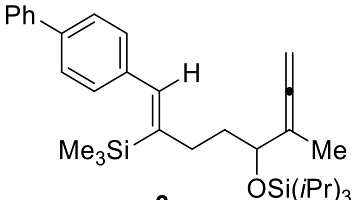

3ga
$C_{33}H_{50}OSi_2$
M = 518.93 g/mol

Prepared from **1g** (78.5 mg, 0.20 mmol) and **2a** (37.9 mg, 0.30 mmol) according to **GP 4**. Flash column chromatography on silica gel using *n*-pentane as the eluent afforded product **3ga** as a colorless oil (80.9 mg, 78% yield).

R*f* = 0.32 (cyclohexane). **IR** (ATR): ṽ = 3027, 2941, 2864, 2285, 2090, 1957, 1599, 1485, 1369, 1247, 1059, 881, 836, 759, 679 cm^{-1}. **^{1}H NMR** (400 MHz, $CDCl_3$, 298 K): *δ* = 7.66–7.61 (m, 2H), 7.57–7.52 (m, 2H), 7.49–7.42 (m, 2H), 7.38–7.32 (m, 1H), 7.28–7.25 (m, 2H), 7.21 (s, 1H), 4.70–4.58 (m, 2H), 4.39–4.33 (m, 1H), 2.30–2.19 (m, 2H), 1.86–1.74 (m, 2H), 1.72 (t, *J* = 3.1 Hz, 3H), 1.14–1.06 (m, 21H), 0.02 (s, 9H) ppm. **^{13}C{^{1}H} NMR** (101 MHz, $CDCl_3$, 298 K): *δ* = 206.7, 144.8, 141.6, 141.0, 139.8, 139.5, 129.2, 128.9, 127.3, 127.1, 126.5, 100.5, 74.7, 74.1, 37.3, 35.3, 18.3, 18.2, 12.6, 12.3, 0.7 ppm. **^{29}Si DEPT NMR** (79 MHz, $CDCl_3$, 298 K, optimized for *J* = 15.0 Hz): *δ* = 12.5, −6.6 ppm. **HRMS** (ESI): calculated for $C_{33}H_{51}OSi_2^+$ [M+H]$^+$: 519.3473; Found 519.3474.

(*Z*)-Triisopropyl((3-methyl-7-(trimethylsilyl)-8-(4-(trimethylsilyl)phenyl)octa-1,2,7-trien-4-yl)oxy)silane (3ha)

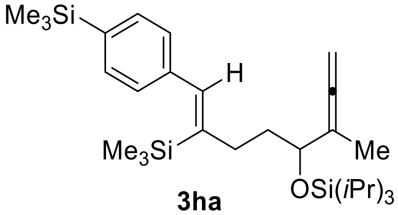

3ha
$C_{30}H_{54}OSi_3$
M = 515.02 g/mol

Prepared from **1h** (77.7 mg, 0.20 mmol) and **2a** (37.9 mg, 0.30 mmol) according to **GP 4**. Flash column chromatography on silica gel using *n*-pentane as the eluent afforded product **3ha** as a colorless oil (72.4 mg, 70% yield).

R*f* = 0.62 (cyclohexane). **IR** (ATR): ṽ = 2943, 2865, 2277, 2088, 1958, 1590, 1461, 1384, 1247, 1060, 881, 832, 755, 679 cm^{-1}. **^{1}H NMR** (500 MHz, $CDCl_3$, 298 K): *δ* = 7.47–7.41 (m, 2H), 7.20–7.14 (m, 3H), 4.69–4.56 (m, 2H), 4.38–4.30 (m, 1H), 2.27–2.16 (m, 2H), 1.84–1.73 (m, 2H), 1.71 (t, *J* = 3.1 Hz, 3H), 1.16–1.03 (m, 21H), 0.28 (s, 9H), −0.02 (s, 9H) ppm. **^{13}C{^{1}H} NMR** (126 MHz, $CDCl_3$, 298 K): *δ* = 206.7, 144.5, 142.1, 141.1, 138.7, 132.9,

128.1, 100.5, 74.7, 74.1, 37.3, 35.2, 18.3, 18.2, 12.6, 12.3, 0.7, −0.9 ppm. **^{29}Si DEPT NMR** (99 MHz, $CDCl_3$, 298 K, optimized for *J* = 15.0 Hz): *δ* = 12.5, −4.2, −6.6 ppm. **HRMS** (ESI): calculated for $C_{30}H_{55}OSi_3^+$ [M+H]$^+$: 515.3555; Found 515.3556.

(*Z*)-*tert*-Butyldimethyl(4-(6-methyl-5-((triisopropylsilyl)oxy)-2-(trimethylsilyl)octa-1,6,7-trien-1-yl)phenoxy)silane (3ia)

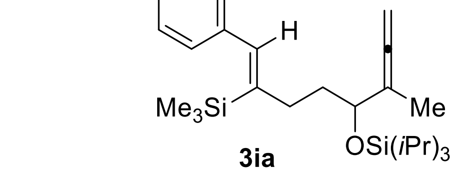

3ia
$C_{33}H_{60}O_2Si_3$
M = 573.10 g/mol

Prepared from **1i** (89.4 mg, 0.20 mmol) and **2a** (37.9 mg, 0.30 mmol) according to **GP 4**. Flash column chromatography on silica gel using *n*-pentane as the eluent afforded product **3ia** as a colorless oil (88.5 mg, 77% yield).

R*f* = 0.57 (cyclohexane). **IR** (ATR): ṽ = 3029, 2940, 2862, 2241, 1959, 1606, 1502, 1462, 1250, 1060, 911, 834, 779, 679 cm^{-1}. **^{1}H NMR** (400 MHz, $CDCl_3$, 298 K): *δ* = 7.11 (s, 1H), 7.06–7.00 (m, 2H), 6.78–6.73 (m, 2H), 4.67–4.55 (m, 2H), 4.35–4.28 (m, 1H), 2.24–2.11 (m, 2H), 1.81–1.70 (m, 2H), 1.69 (t, *J* = 3.1 Hz, 3H), 1.11–1.05 (m, 21H), 0.98 (s, 9H), 0.19 (s, 6H), −0.04 (s, 9H) ppm. **^{13}C{^{1}H} NMR** (101 MHz, $CDCl_3$, 298 K): *δ* = 206.6, 154.7, 143.5, 141.8, 134.1, 129.8, 119.6, 100.4, 74.7, 74.0, 37.3, 35.1, 25.9, 18.4, 18.3, 18.2, 12.5, 12.2, 0.7, −4.3 ppm. **^{29}Si DEPT NMR** (79 MHz, $CDCl_3$, 298 K, optimized for *J* = 15.0 Hz): *δ* = 20.9, 12.5, −6.8 ppm. **HRMS** (APCI): calculated for $C_{33}H_{61}O_2Si_3^+$ [M+H]$^+$: 573.3974; Found 573.3974.

(*Z*)-((8-(2-Chlorophenyl)-3-methyl-7-(trimethylsilyl)octa-1,2,7-trien-4-yl)oxy)-triisopropylsilane (3ja)

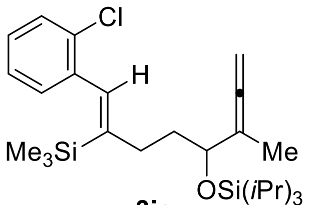

3ja
$C_{27}H_{45}ClOSi_2$
M = 477.28 g/mol

Prepared from **1j** (70.2 mg, 0.20 mmol) and **2a** (37.9 mg, 0.30 mmol) according to **GP 4**. Flash column chromatography on silica gel using *n*-pentane as the eluent afforded product **3ja** as a colorless oil (56.9 mg, 60% yield).

R*f* = 0.51 (cyclohexane). **IR** (ATR): ṽ = 3054, 2941, 2864, 2292, 2085, 1958, 1703, 1585,

1463, 1247, 1054, 881, 835, 750, 679 cm^{-1}. **^{1}H NMR** (500 MHz, $CDCl_3$, 298 K): δ = 7.35–7.29 (m, 1H), 7.22–7.15 (m, 3H), 7.05–7.02 (m, 1H), 4.68–4.57 (m, 2H), 4.38–4.31 (m, 1H), 2.28–2.20 (m, 2H), 1.84–1.73 (m, 2H), 1.70 (t, J = 3.1 Hz, 3H), 1.11–1.04 (m, 21H), −0.09 (s, 9H) ppm. **^{13}C{^{1}H} NMR** (126 MHz, $CDCl_3$, 298 K): δ = 206.6, 145.9, 139.3, 139.1, 133.7, 131.2, 129.0, 128.5, 126.1, 100.4, 74.7, 74.1, 37.1, 34.6, 18.3, 18.2, 12.5, 12.3, 0.3 ppm. **^{29}Si DEPT NMR** (99 MHz, $CDCl_3$, 298 K, optimized for J = 15.0 Hz): δ = 12.5, −6.0 ppm. **HRMS** (ESI): calculated for $C_{27}H_{46}ClOSi_2^+$ [M+H]$^+$: 477.2770; Found 477.2767.

(*Z*)-((8-(2-Bromophenyl)-3-methyl-7-(trimethylsilyl)octa-1,2,7-trien-4-yl)oxy)-triisopropylsilane (3ka)

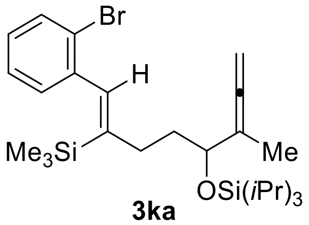

3ka

$C_{27}H_{45}BrOSi_2$

M = 521.73 g/mol

Prepared from **1k** (79.1 mg, 0.20 mmol) and **2a** (37.9 mg, 0.30 mmol) according to **GP 4**. Flash column chromatography on silica gel using *n*-pentane as the eluent afforded product **3ka** as a colorless oil (63.6 mg, 61% yield).

R*f* = 0.50 (cyclohexane). **IR** (ATR): ṽ = 3053, 2941, 2864, 2557, 2275, 2086, 1958, 1699, 1582, 1461, 1247, 1060, 881, 835, 749, 677 cm^{-1}. **^{1}H NMR** (400 MHz, $CDCl_3$, 298 K): δ = 7.54–7.50 (m, 1H), 7.26–7.17 (m, 2H), 7.14–7.07 (m, 1H), 6.98 (s, 1H), 4.68–4.57 (m, 2H), 4.38–4.32 (m, 1H), 2.28–2.17 (m, 2H), 1.84–1.73 (m, 2H), 1.70 (t, J = 3.1 Hz, 3H), 1.13–1.02 (m, 21H), −0.09 (s, 9H) ppm. **^{13}C{^{1}H} NMR** (101 MHz, $CDCl_3$, 298 K): δ = 206.6, 145.5, 141.2, 132.1, 131.2, 128.6, 126.8, 124.0, 100.4, 74.7, 74.1, 37.0, 34.4, 18.3, 18.2, 12.5, 12.3, 0.3 ppm. **^{29}Si DEPT NMR** (79 MHz, $CDCl_3$, 298 K, optimized for J = 15.0 Hz): δ = 12.5, −6.0 ppm. **HRMS** (ESI): calculated for $C_{27}H_{46}BrOSi_2^+$ [M+H]$^+$: 521.2265; Found 521.2266.

(*Z*)-((8-(3-Fluorophenyl)-3-methyl-7-(trimethylsilyl)octa-1,2,7-trien-4-yl)oxy)-triisopropylsilane (3la)

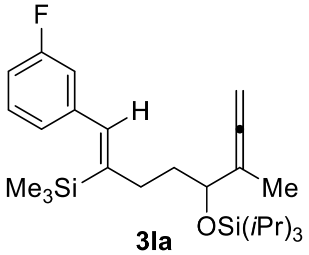

3la

$C_{27}H_{45}FOSi_2$

M = 460.82 g/mol

Prepared from **1l** (66.9 mg, 0.20 mmol) and **2a** (37.9 mg, 0.30 mmol) according to **GP 4**. Flash column chromatography on silica gel using *n*-pentane as the eluent afforded product **3la** as a colorless oil (45.9 mg, 50% yield).

R*f* = 0.63 (cyclohexane). **IR** (ATR): ṽ = 2942, 2865, 2567, 2240, 2080, 1958, 1578, 1461, 1369, 1249, 1060, 880, 834, 785, 757, 680 cm^{-1}. **^{1}H NMR** (500 MHz, $CDCl_3$, 298 K): δ = 7.32–7.27 (m, 1H), 7.16 (s, 1H), 7.03–6.89 (m, 3H), 4.74–4.61 (m, 2H), 4.41–4.35 (m, 1H), 2.31–2.20 (m, 2H), 1.88–1.77 (m, 2H), 1.75 (t, *J* = 3.2 Hz, 3H), 1.20–1.07 (m, 21H), 0.02 (s, 9H) ppm. **^{13}C{^{1}H} NMR** (126 MHz, $CDCl_3$, 298 K): δ = 206.6, 162.5 (d, $J_{C,F}$ = 245.8 Hz), 145.9, 143.1 (d, $J_{C,F}$ = 7.4 Hz), 140.5, 129.3 (d, $J_{C,F}$ = 8.4 Hz), 124.5 (d, $J_{C,F}$ = 2.9 Hz), 115.6 (d, $J_{C,F}$ = 21.0 Hz), 113.6 (d, $J_{C,F}$ = 21.1 Hz), 100.4, 74.6, 74.1, 37.1, 35.0, 18.3, 18.2, 12.5, 12.3, 0.5 ppm. **^{19}F NMR** (471 MHz, $CDCl_3$, 298 K): δ = −114.2 ppm. **^{29}Si DEPT NMR** (99 MHz, $CDCl_3$, 298 K, optimized for *J* = 15.0 Hz): δ = 12.5, −6.4 ppm. **HRMS** (ESI): calculated for $C_{27}H_{46}FOSi_2^+$ $[M+H]^+$: 461.3066; Found 461.3066.

(*Z*)-((8-(3-Chlorophenyl)-3-methyl-7-(trimethylsilyl)octa-1,2,7-trien-4-yl)oxy)-triisopropylsilane (3ma)

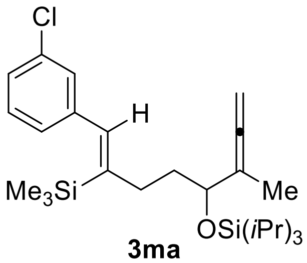

3ma
$C_{27}H_{45}ClOSi_2$
M = 477.28 g/mol

Prepared from **1m** (70.2 mg, 0.20 mmol) and **2a** (37.9 mg, 0.30 mmol) according to **GP 4**. Flash column chromatography on silica gel using *n*-pentane as the eluent afforded product **3ma** as a colorless oil (41.2 mg, 43% yield).

R*f* = 0.49 (cyclohexane). **IR** (ATR): ṽ = 3055, 2942, 2864, 2279, 2094, 1958, 1703, 1588, 1463, 1248, 1060, 880, 834, 780, 682 cm^{-1}. **^{1}H NMR** (500 MHz, $CDCl_3$, 298 K): δ = 7.23–7.16 (m, 3H), 7.09 (s, 1H), 7.07–7.02 (m, 1H), 4.69–4.56 (m, 2H), 4.37–4.29 (m, 1H), 2.26–2.17 (m, 2H), 1.82–1.72 (m, 2H), 1.70 (t, *J* = 3.1 Hz, 3H), 1.13–1.03 (m, 21H), −0.03 (s, 9H) ppm. **^{13}C{^{1}H} NMR** (126 MHz, $CDCl_3$, 298 K): δ = 206.6, 146.2, 142.6, 140.3, 133.7, 129.1, 128.9, 126.9, 100.4, 74.6, 74.1, 37.1, 35.0, 18.3, 18.2, 12.5, 12.3, 0.6 ppm. **^{29}Si DEPT NMR** (99 MHz, $CDCl_3$, 298 K, optimized for *J* = 15.0 Hz): δ = 12.6, −6.4 ppm. **HRMS** (ESI): calculated for $C_{27}H_{46}ClOSi_2^+$ $[M+H]^+$: 477.2770; Found 477.2766.

(*Z*)-((8-(4-Fluorophenyl)-3-methyl-7-(trimethylsilyl)octa-1,2,7-trien-4-yl)oxy)-triisopropylsilane (3na)

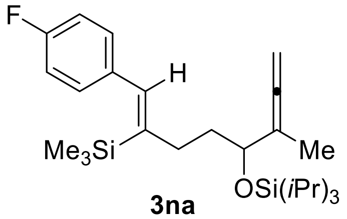

3na
$C_{27}H_{45}FOSi_2$
M = 460.82 g/mol

Prepared from **1n** (66.9 mg, 0.20 mmol) and **2a** (37.9 mg, 0.30 mmol) according to **GP 4**. Flash column chromatography on silica gel using *n*-pentane as the eluent afforded product **3na** as a colorless oil (55.7 mg, 60% yield).

R*f* = 0.68 (cyclohexane). **IR** (ATR): ṽ = 3047, 2942, 2865, 2154, 1959, 1600, 1503, 1369, 1224, 1060, 881, 834, 756, 680 cm^{-1}. **^{1}H NMR** (400 MHz, $CDCl_3$, 298 K): δ = 7.15–7.08 (m, 3H), 7.00–6.93 (m, 2H), 4.68–4.55 (m, 2H), 4.36–4.29 (m, 1H), 2.26–2.13 (m, 2H), 1.82–1.71 (m, 2H), 1.70 (t, J = 3.1 Hz, 3H), 1.13–1.02 (m, 21H), −0.05 (s, 9H) ppm. **^{13}C{^{1}H} NMR** (101 MHz, $CDCl_3$, 298 K): δ = 206.6, 162.0 (d, $J_{C,F}$ = 245.3 Hz), 145.0, 140.8, 136.8 (d, $J_{C,F}$ = 3.3 Hz), 130.2 (d, $J_{C,F}$ = 7.9 Hz), 114.7 (d, $J_{C,F}$ = 21.2 Hz), 100.5, 74.7, 74.1, 37.2, 35.0, 18.3, 18.2, 12.6, 12.3, 0.6 ppm. **^{19}F NMR** (471 MHz, $CDCl_3$, 298 K): δ = −116.0 ppm. **^{29}Si DEPT NMR** (79 MHz, $CDCl_3$, 298 K, optimized for J = 15.0 Hz): δ = 12.5, −6.6 ppm. **HRMS** (APCI): calculated for $C_{27}H_{46}FOSi_2^+$ $[M+H]^+$: 461.3066; Found 461.3063.

(*Z*)-((8-(4-Chlorophenyl)-3-methyl-7-(trimethylsilyl)octa-1,2,7-trien-4-yl)oxy)-triisopropylsilane (3oa)

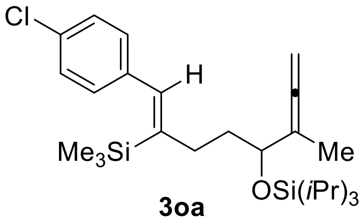

3oa
$C_{27}H_{45}ClOSi_2$
M = 477.28 g/mol

Prepared from **1o** (70.2 mg, 0.20 mmol) and **2a** (37.9 mg, 0.30 mmol) according to **GP 4**. Flash column chromatography on silica gel using *n*-pentane as the eluent afforded product **3oa** as a colorless oil (53.8 mg, 56% yield).

R*f* = 0.66 (cyclohexane). **IR** (ATR): ṽ = 2942, 2864, 2369, 2206, 2161, 1959, 1702, 1485, 1461, 1248, 1086, 1059, 881, 836, 757, 679 cm^{-1}. **^{1}H NMR** (400 MHz, $CDCl_3$, 298 K): δ = 7.28–7.23 (m, 2H), 7.13–7.07 (m, 3H), 4.69–4.56 (m, 2H), 4.36–4.30 (m, 1H), 2.26–2.15 (m, 2H), 1.81–1.72 (m, 2H), 1.70 (t, J = 3.1 Hz, 3H), 1.12–1.04 (m, 21H), −0.03 (s, 9H) ppm. **^{13}C{^{1}H} NMR** (101 MHz, $CDCl_3$, 298 K): δ = 206.6, 145.6, 140.6, 139.3, 132.7, 130.1,

128.0, 100.4, 74.6, 74.1, 37.1, 35.1, 18.3, 18.2, 12.5, 12.3, 0.6 ppm. **^{29}Si DEPT NMR** (79 MHz, $CDCl_3$, 298 K, optimized for J = 15.0 Hz): δ = 12.5, −6.5 ppm. **HRMS** (ESI): calculated for $C_{27}H_{46}ClOSi_2^+$ [M+H]$^+$: 477.2770; Found 477.2769.

(*Z*)-((8-(4-Bromophenyl)-3-methyl-7-(trimethylsilyl)octa-1,2,7-trien-4-yl)oxy)-triisopropylsilane (3pa)

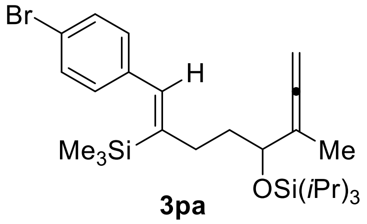

3pa
$C_{27}H_{45}BrOSi_2$
M = 521.73 g/mol

Prepared from **1p** (79.1 mg, 0.20 mmol) and **2a** (37.9 mg, 0.30 mmol) according to **GP 4**. Flash column chromatography on silica gel using *n*-pentane as the eluent afforded product **3pa** as a colorless oil (63.7 mg, 61% yield).

R*f* = 0.57 (cyclohexane). **IR** (ATR): ṽ = 2941, 2864, 2274, 2093, 1957, 1711, 1583, 1482, 1385, 1248, 1062, 881, 836, 757, 678 cm^{-1}. **^{1}H NMR** (400 MHz, $CDCl_3$, 298 K): δ = 7.43–7.36 (m, 2H), 7.07–7.00 (m, 3H), 4.68–4.54 (m, 2H), 4.34–4.28 (m, 1H), 2.25–2.13 (m, 2H), 1.82–1.70 (m, 2H), 1.69 (t, J = 3.1 Hz, 3H), 1.13–1.01 (m, 21H), −0.04 (s, 9H) ppm. **^{13}C{^{1}H} NMR** (101 MHz, $CDCl_3$, 298 K): δ = 206.6, 145.7, 140.6, 139.7, 131.0, 130.4, 120.7, 100.4, 74.6, 74.1, 37.1, 35.1, 18.3, 18.2, 12.5, 12.3, 0.6 ppm. **^{29}Si DEPT NMR** (79 MHz, $CDCl_3$, 298 K, optimized for J = 15.0 Hz): δ = 12.5, −6.5 ppm. **HRMS** (ESI): calculated for $C_{27}H_{46}BrOSi_2^+$ [M+H]$^+$: 521.2265; Found 521.2268.

(*Z*)-Triisopropyl((3-methyl-8-(naphthalen-2-yl)-7-(trimethylsilyl)octa-1,2,7-trien-4-yl)oxy)silane (3qa)

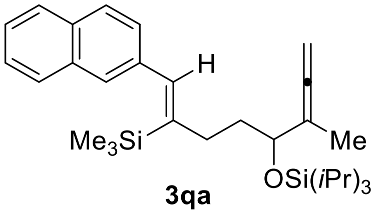

3qa
$C_{31}H_{48}OSi_2$
M = 492.89 g/mol

Prepared from **1q** (73.3 mg, 0.20 mmol) and **2a** (37.9 mg, 0.30 mmol) according to **GP 4**. Flash column chromatography on silica gel using *n*-pentane as the eluent afforded product **3qa** as a colorless oil (77.7 mg, 79% yield).

R*f* = 0.49 (cyclohexane). **IR** (ATR): ṽ = 3053, 2941, 2864, 2370, 2161, 1958, 1700, 1590, 1502, 1461, 1247, 1059, 881, 834, 736, 679 cm^{-1}. **^{1}H NMR** (400 MHz, $CDCl_3$, 298 K): δ = 7.86–7.73 (m, 3H), 7.65 (s, 1H), 7.51–7.42 (m, 2H), 7.36–7.29 (m, 2H), 4.71–4.58 (m, 2H),

4.41–4.34 (m, 1H), 2.34–2.22 (m, 2H), 1.90–1.76 (m, 2H), 1.73 (t, J = 3.2 Hz, 3H), 1.18–1.03 (m, 21H), −0.02 (s, 9H) ppm. **^{13}C{^{1}H} NMR** (101 MHz, CDCl$_3$, 298 K): δ = 206.7, 145.1, 142.0, 138.3, 133.2, 132.5, 128.0, 127.8, 127.4, 127.35, 127.30, 126.2, 125.7, 100.5, 74.7, 74.1, 37.3, 35.3, 18.3, 18.2, 12.6, 12.3, 0.7 ppm. **^{29}Si DEPT NMR** (79 MHz, CDCl$_3$, 298 K, optimized for J = 15.0 Hz): δ = 12.5, −6.5 ppm. **HRMS** (ESI): calculated for $C_{31}H_{49}OSi_2^+$ [M+H]$^+$: 493.3316; Found 493.3314.

(*Z*)-((8-(Benzo[*b*]thiophen-2-yl)-3-methyl-7-(trimethylsilyl)octa-1,2,7-trien-4-yl)oxy)-triisopropylsilane (3ra)

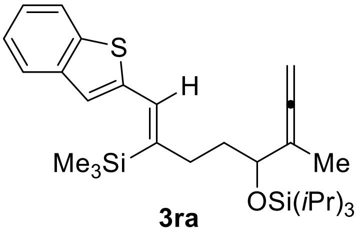

3ra
$C_{29}H_{46}OSSi_2$
M = 498.92 g/mol

Prepared from **1r** (74.5 mg, 0.20 mmol) and **2a** (37.9 mg, 0.30 mmol) according to **GP 4**. Flash column chromatography on silica gel using *n*-pentane as the eluent afforded product **3ra** as a colorless oil (77.2 mg, 77% yield).

R*f* = 0.52 (cyclohexane). **IR** (ATR): ṽ = 3056, 2941, 2863, 2286, 2086, 1958, 1594, 1458, 1247, 1060, 880, 836, 741, 679 cm^{-1}. **^{1}H NMR** (400 MHz, CDCl$_3$, 298 K): δ = 7.80–7.75 (m, 1H), 7.74–7.69 (m, 1H), 7.36–7.26 (m, 2H), 7.11–7.07 (m, 1H), 7.05 (s, 1H), 4.70–4.57 (m, 2H), 4.38–4.30 (m, 1H), 2.32–2.19 (m, 2H), 1.84–1.72 (m, 2H), 1.71 (t, J = 3.1 Hz, 3H), 1.14–1.02 (m, 21H), 0.10 (s, 9H) ppm. **^{13}C{^{1}H} NMR** (101 MHz, CDCl$_3$, 298 K): δ = 206.6, 149.4, 143.3, 140.3, 140.0, 133.3, 124.3, 124.1, 123.4, 123.0, 122.2, 100.4, 74.6, 74.2, 36.9, 35.4, 18.3, 18.2, 12.5, 12.3, 0.4 ppm. **^{29}Si DEPT NMR** (79 MHz, CDCl$_3$, 298 K, optimized for J = 15.0 Hz): δ = 12.7, −6.1 ppm. **HRMS** (ESI): calculated for $C_{29}H_{47}OSSi_2^+$ [M+H]$^+$: 499.2881; Found 499.2882.

(*Z*)-Triisopropyl((3-methyl-8-(thiophen-2-yl)-7-(trimethylsilyl)octa-1,2,7-trien-4-yl)oxy)silane (3sa)

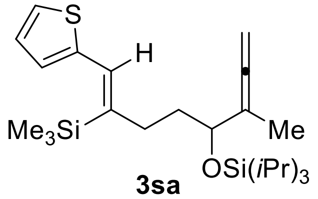

3sa
$C_{25}H_{44}OSSi_2$
M = 448.86 g/mol

Prepared from **1s** (64.5 mg, 0.20 mmol) and **2a** (37.9 mg, 0.30 mmol) according to **GP 4**. Flash column chromatography on silica gel using *n*-pentane as the eluent afforded product

3sa as a colorless oil (65.8 mg, 73% yield).

R*f* = 0.60 (cyclohexane). **IR** (ATR): ṽ = 2941, 2864, 2487, 2156, 2024, 1959, 1461, 1248, 1059, 881, 835, 755, 683 cm^{-1}. **^{1}H NMR** (400 MHz, $CDCl_3$, 298 K): *δ* = 7.20 (dd, *J* = 5.1, 1.2 Hz, 1H), 7.06–7.03 (m, 1H), 6.94 (dd, *J* = 5.1, 3.4 Hz, 1H), 6.84 (dt, *J* = 3.5, 1.2 Hz, 1H), 4.69–4.55 (m, 2H), 4.36–4.28 (m, 1H), 2.27–2.15 (m, 2H), 1.80–1.70 (m, 2H), 1.69 (t, *J* = 3.1 Hz, 3H), 1.11–1.05 (m, 21H), 0.06 (s, 9H) ppm. **^{13}C{^{1}H} NMR** (101 MHz, $CDCl_3$, 298 K): *δ* = 206.6, 147.6, 142.9, 133.2, 126.8, 126.5, 124.9, 100.4, 74.6, 74.1, 37.1, 35.3, 18.3, 18.2, 12.5, 12.2, 0.3 ppm. **^{29}Si DEPT NMR** (79 MHz, $CDCl_3$, 298 K, optimized for *J* = 15.0 Hz): *δ* = 12.5, −6.4 ppm. **HRMS** (ESI): calculated for $C_{25}H_{45}OSSi_2^+$ [M+H]$^+$: 449.2724; Found 449.2722.

(*Z*)-(5-((Diisopropyl(methyl)silyl)oxy)-6-methyl-1-phenylocta-1,6,7-trien-2-yl)-trimethylsilane (3ua)

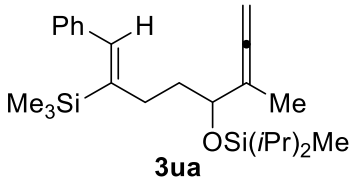

3ua
$C_{25}H_{42}OSi_2$
M = 414.78 g/mol

Prepared from **1u** (57.7 mg, 0.20 mmol) and **2a** (37.9 mg, 0.30 mmol) according to **GP 4**. Flash column chromatography on silica gel using *n*-pentane as the eluent afforded product **3ua** as a colorless oil (43.5 mg, 52% yield).

R*f* = 0.67 (cyclohexane). **IR** (ATR): ṽ = 3055, 2941, 2863, 2284, 2078, 1958, 1802, 1591, 1461, 1248, 1073, 833, 747, 697 cm^{-1}. **^{1}H NMR** (400 MHz, $CDCl_3$, 298 K): *δ* = 7.33–7.27 (m, 2H), 7.27–7.22 (m, 1H), 7.22–7.17 (m, 3H), 4.71–4.57 (m, 2H), 4.27–4.21 (m, 1H), 2.37–2.15 (m, 2H), 1.83–1.72 (m, 2H), 1.71 (t, *J* = 3.2 Hz, 3H), 1.09–0.88 (m, 14H), 0.07 (s, 3H), −0.03 (s, 9H) ppm. **^{13}C{^{1}H} NMR** (101 MHz, $CDCl_3$, 298 K): *δ* = 206.4, 144.4, 141.9, 140.8, 128.7, 127.9, 126.8, 100.7, 74.7, 74.1, 37.1, 35.3, 17.8, 17.7, 17.60, 17.57, 13.5, 13.4, 12.4, 0.6, −7.7 ppm. **^{29}Si DEPT NMR** (79 MHz, $CDCl_3$, 298 K, optimized for *J* = 15.0 Hz): *δ* = 17.3, −6.6 ppm. **HRMS** (ESI): calculated for $C_{25}H_{43}OSi_2^+$ [M+H]$^+$: 415.2847; Found 415.2845.

(*Z*)-*tert*-Butyldimethyl((3-methyl-8-phenyl-7-(trimethylsilyl)octa-1,2,7-trien-4-yl)oxy)-silane (3va)

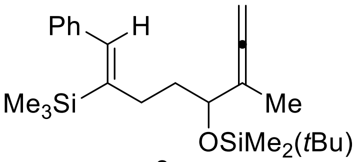

3va
$C_{24}H_{40}OSi_2$
M = 400.75 g/mol

Prepared from **1v** (54.9 mg, 0.20 mmol) and **2a** (37.9 mg, 0.30 mmol) according to **GP 4**. Flash column chromatography on silica gel using *n*-pentane as the eluent afforded product **3va** as a colorless oil (48.3 mg, 60% yield).

R*f* = 0.69 (cyclohexane). **IR** (ATR): ṽ = 2951, 2855, 2346, 2088, 1957, 1803, 1591, 1469, 1248, 1071, 831, 747, 697 cm^{-1}. **^{1}H NMR** (500 MHz, $CDCl_3$, 298 K): δ = 7.33–7.27 (m, 2H), 7.27–7.22 (m, 1H), 7.21–7.17 (m, 3H), 4.72–4.57 (m, 2H), 4.24–4.16 (m, 1H), 2.43–2.32 (m, 1H), 2.28–2.16 (m, 1H), 1.85–1.62 (m, 5H), 0.94 (s, 9H), 0.10 (s, 3H), 0.08 (s, 3H), −0.03 (s, 9H) ppm. **^{13}C{^{1}H} NMR** (126 MHz, $CDCl_3$, 298 K): δ = 206.2, 144.5, 141.9, 140.9, 128.7, 127.9, 126.8, 101.0, 74.5, 74.2, 37.0, 35.5, 26.0, 18.4, 12.6, 0.6, −4.5, −4.9 ppm. **^{29}Si DEPT NMR** (99 MHz, $CDCl_3$, 298 K, optimized for J = 15.0 Hz): δ = 18.1, −6.6 ppm. **HRMS** (ESI): calculated for $C_{24}H_{41}OSi_2^+$ [M+H]$^+$: 401.2690; Found 401.2688.

(*Z*)-(2,3-Dimethylbutan-2-yl)dimethyl((3-methyl-8-phenyl-7-(trimethylsilyl)octa-1,2,7-trien-4-yl)oxy)silane (3wa)

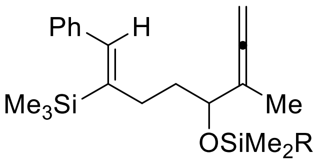

R = 2,3-dimethyl-2-butyl
3wa
$C_{26}H_{44}OSi_2$
M = 428.81 g/mol

Prepared from **1w** (60.5 mg, 0.20 mmol) and **2a** (37.9 mg, 0.30 mmol) according to **GP 4**. Flash column chromatography on silica gel using *n*-pentane as the eluent afforded product **3wa** as a colorless oil (68.7 mg, 80% yield).

R*f* = 0.60 (cyclohexane). **IR** (ATR): ṽ = 2952, 2865, 2289, 2084, 1957, 1705, 1592, 1462, 1249, 1070, 828, 747, 697 cm^{-1}. **^{1}H NMR** (500 MHz, $CDCl_3$, 298 K): δ = 7.33–7.27 (m, 2H), 7.27–7.22 (m, 1H), 7.22–7.17 (m, 3H), 4.71–4.56 (m, 2H), 4.24–4.17 (m, 1H), 2.42–2.32 (m, 1H), 2.27–2.16 (m, 1H), 1.83–1.73 (m, 1H), 1.73–1.62 (m, 5H), 0.94 (d, J = 1.4 Hz, 3H), 0.92 (d, J = 1.4 Hz, 3H), 0.89 (d, J = 2.9 Hz, 6H), 0.14 (s, 3H), 0.12 (s, 3H), −0.03 (s, 9H) ppm. **^{13}C{^{1}H} NMR** (126 MHz, $CDCl_3$, 298 K): δ = 206.3, 144.5, 141.9, 140.9, 128.7, 127.9, 126.8, 100.9, 74.5, 74.1, 37.0, 35.5, 34.3, 25.2, 20.55, 20.47, 18.82, 18.76, 12.6, 0.6, −2.4,

−2.9 ppm. **^{29}Si DEPT NMR** (99 MHz, $CDCl_3$, 298 K, optimized for *J* = 15.0 Hz): *δ* = 18.4, −6.6 ppm. **HRMS** (ESI): calculated for $C_{26}H_{45}OSi_2^+$ [M+H]$^+$: 429.3003; Found 429.3000.

(*Z*)-Ethyldimethyl(6-methyl-1-phenyl-5-((triisopropylsilyl)oxy)octa-1,6,7-trien-2-yl)-silane (3ab)

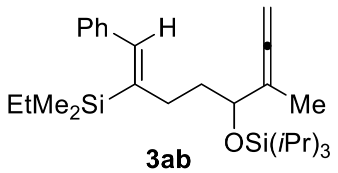

3ab
$C_{28}H_{48}OSi_2$
M = 456.86 g/mol

Prepared from **1a** (63.3 mg, 0.20 mmol) and **2b** (42.1 mg, 0.30 mmol) according to **GP 4**. Flash column chromatography on silica gel using *n*-pentane as the eluent afforded product **3ab** as a colorless oil (69.5 mg, 76% yield).

R*f* = 0.68 (cyclohexane). **IR** (ATR): ṽ = 2942, 2865, 2351, 2119, 1958, 1591, 1461, 1249, 1059, 1009, 881, 813, 747, 679 cm^{-1}. **^{1}H NMR** (400 MHz, $CDCl_3$, 298 K): *δ* = 7.34–7.29 (m, 2H), 7.28–7.23 (m, 2H), 7.23–7.19 (m, 2H), 4.72–4.61 (m, 2H), 4.41–4.35 (m, 1H), 2.30–2.19 (m, 2H), 1.88–1.76 (m, 2H), 1.75 (t, *J* = 3.1 Hz, 3H), 1.16–1.09 (m, 21H), 0.86 (t, *J* = 7.9 Hz, 3H), 0.46 (q, *J* = 7.9 Hz, 2H), −0.03 (s, 3H), −0.04 (s, 3H) ppm. **^{13}C{^{1}H} NMR** (101 MHz, $CDCl_3$, 298 K): *δ* = 206.7, 143.7, 142.3, 140.9, 128.7, 127.8, 126.8, 100.5, 74.7, 74.1, 37.3, 35.0, 18.3, 18.2, 12.6, 12.3, 8.4, 7.6, −1.8 ppm. **^{29}Si DEPT NMR** (79 MHz, $CDCl_3$, 298 K, optimized for *J* = 15.0 Hz): *δ* = 12.5, −3.7 ppm. **HRMS** (APCI): calculated for $C_{28}H_{49}OSi_2^+$ [M+H]$^+$: 457.3316; Found 457.3312.

(*Z*)-Butyldimethyl(6-methyl-1-phenyl-5-((triisopropylsilyl)oxy)octa-1,6,7-trien-2-yl)-silane (3ac)

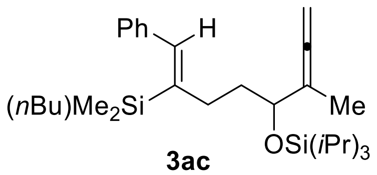

3ac
$C_{30}H_{52}OSi_2$
M = 484.92 g/mol

Prepared from **1a** (63.3 mg, 0.20 mmol) and **2c** (50.5 mg, 0.30 mmol) according to **GP 4**. Flash column chromatography on silica gel using *n*-pentane as the eluent afforded product **3ac** as a colorless oil (74.5 mg, 77% yield).

R*f* = 0.72 (cyclohexane). **IR** (ATR): ṽ = 2942, 2864, 2368, 2161, 1959, 1591, 1461, 1370, 1248, 1060, 880, 813, 747, 679 cm^{-1}. **^{1}H NMR** (400 MHz, $CDCl_3$, 298 K): *δ* = 7.34–7.29 (m, 2H), 7.28–7.25 (m, 1H), 7.24–7.19 (m, 3H), 4.72–4.60 (m, 2H), 4.41–4.35 (m, 1H), 2.29–2.19 (m, 2H), 1.88–1.77 (m, 2H), 1.75 (t, *J* = 3.1 Hz, 3H), 1.28–1.17 (m, 4H), 1.16–

1.09 (m, 21H), 0.85 (t, *J* = 7.0 Hz, 3H), 0.50–0.43 (m, 2H), −0.03 (s, 3H), −0.03 (s, 3H) ppm. **^{13}C{^{1}H} NMR** (101 MHz, $CDCl_3$, 298 K): *δ* = 206.6, 143.9, 142.2, 140.9, 128.7, 127.8, 126.8, 100.5, 74.7, 74.0, 37.3, 35.0, 26.7, 26.3, 18.3, 18.2, 16.4, 13.9, 12.6, 12.3, −1.2 ppm. **^{29}Si DEPT NMR** (79 MHz, $CDCl_3$, 298 K, optimized for *J* = 15.0 Hz): *δ* = 12.4, −5.1 ppm. **HRMS** (APCI): calculated for $C_{30}H_{53}OSi_2^+$ [M+H]$^+$: 485.3629; Found 485.3625.

(*Z*)-Triisopropyl((7-(*iso*propyldimethylsilyl)-3-methyl-8-phenylocta-1,2,7-trien-4-yl)oxy)silane (3ad)

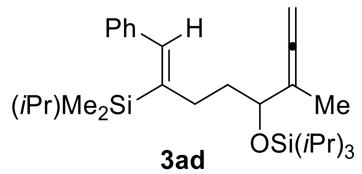

3ad

$C_{29}H_{50}OSi_2$

M = 470.89 g/mol

Prepared from **1a** (63.3 mg, 0.20 mmol) and **2d** (46.3 mg, 0.30 mmol) according to **GP 4**. Flash column chromatography on silica gel using *n*-pentane as the eluent afforded product **3ad** as a colorless oil (52.8 mg, 56% yield).

R*f* = 0.71 (cyclohexane). **IR** (ATR): ṽ = 3055, 2941, 2863, 2465, 2258, 1959, 1714, 1591, 1461, 1249, 1060, 880, 810, 747, 679 cm^{-1}. **^{1}H NMR** (400 MHz, $CDCl_3$, 298 K): *δ* = 7.39–7.33 (m, 2H), 7.33–7.28 (m, 2H), 7.28–7.23 (m, 2H), 4.77–4.65 (m, 2H), 4.46–4.39 (m, 1H), 2.34–2.22 (m, 2H), 1.94–1.82 (m, 2H), 1.79 (t, *J* = 3.1 Hz, 3H), 1.22–1.14 (m, 21H), 0.95 (d, *J* = 1.7 Hz, 3H), 0.93 (d, *J* = 1.7 Hz, 3H), 0.83–0.71 (m, 1H), −0.03 (s, 6H) ppm. **^{13}C{^{1}H} NMR** (101 MHz, $CDCl_3$, 298 K): *δ* = 206.6, 143.2, 142.4, 140.9, 128.7, 127.8, 126.8, 100.5, 74.7, 74.1, 37.3, 34.7, 18.3, 18.2, 17.8, 13.6, 12.6, 12.3, −3.6, −3.7 ppm. **^{29}Si DEPT NMR** (79 MHz, $CDCl_3$, 298 K, optimized for *J* = 15.0 Hz): *δ* = 12.4, −1.1 ppm. **HRMS** (APCI): calculated for $C_{29}H_{51}OSi_2^+$ [M+H]$^+$: 471.3473; Found 471.3469.

(*Z*)-Triethyl(6-methyl-1-phenyl-5-((triisopropylsilyl)oxy)octa-1,6,7-trien-2-yl)silane (3ag)

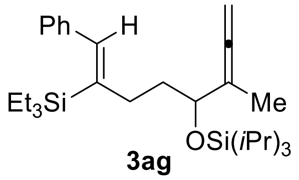

3ag

$C_{30}H_{52}OSi_2$

M = 484.92 g/mol

Prepared from **1a** (63.3 mg, 0.20 mmol) and **2g** (50.5 mg, 0.30 mmol) according to **GP 4**. Flash column chromatography on silica gel using *n*-pentane as the eluent afforded product **3ag** as a colorless oil (64.1 mg, 66% yield).

R$_f$ = 0.64 (cyclohexane). **IR** (ATR): ṽ = 3055, 2944, 2867, 2245, 1958, 1590, 1460, 1416, 1238, 1060, 999, 881, 729, 677 cm^{-1}. **^{1}H NMR** (400 MHz, CDCl$_3$, 298 K): δ = 7.34–7.26 (m, 4H), 7.23–7.18 (m, 2H), 4.72–4.60 (m, 2H), 4.40–4.33 (m, 1H), 2.26–2.13 (m, 2H), 1.89–1.75 (m, 2H), 1.74 (t, J = 3.1 Hz, 3H), 1.16–1.08 (m, 21H), 0.87 (t, J = 7.9 Hz, 9H), 0.47 (q, J = 7.9 Hz, 6H) ppm. **^{13}C{^{1}H} NMR** (101 MHz, CDCl$_3$, 298 K): δ = 206.6, 142.8, 142.0, 140.9, 128.6, 127.7, 126.8, 100.6, 74.8, 74.1, 37.4, 34.4, 18.3, 18.2, 12.6, 12.3, 7.7, 4.4 ppm. **^{29}Si DEPT NMR** (79 MHz, CDCl$_3$, 298 K, optimized for J = 15.0 Hz): δ = 12.4, 1.3 ppm. **HRMS** (APCI): calculated for $C_{30}H_{53}OSi_2^+$ [M+H]$^+$: 485.3629; Found 485.3625.

(*Z*)-(6-Ethyl-1-phenyl-5-((triisopropylsilyl)oxy)octa-1,6,7-trien-2-yl)trimethylsilane (3ah)

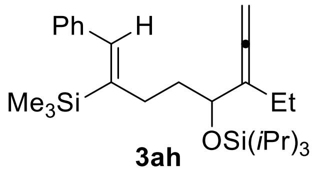

3ah
$C_{28}H_{48}OSi_2$
M = 456.86 g/mol

Prepared from **1a** (63.3 mg, 0.20 mmol) and **2h** (42.1 mg, 0.30 mmol) according to **GP 4**. Flash column chromatography on silica gel using *n*-pentane as the eluent afforded product **3ah** as a colorless oil (73.4 mg, 80% yield).
R$_f$ = 0.70 (cyclohexane). **IR** (ATR): ṽ = 2941, 2864, 2437, 2162, 2037, 1591, 1460, 1381, 1247, 1056, 881, 834, 747, 680 cm^{-1}. **^{1}H NMR** (400 MHz, CDCl$_3$, 298 K): δ = 7.36–7.30 (m, 2H), 7.29–7.24 (m, 1H), 7.23–7.18 (m, 3H), 4.85–4.71 (m, 2H), 4.41 (t, J = 6.8 Hz, 1H), 2.34–2.20 (m, 2H), 2.23–2.09 (m, 1H), 2.08–1.95 (m, 1H), 1.90–1.74 (m, 2H), 1.17–1.05 (m, 21H), 1.09 (t, J = 7.4 Hz, 3H), −0.01 (s, 9H) ppm. **^{13}C{^{1}H} NMR** (101 MHz, CDCl$_3$, 298 K): δ = 206.2, 144.5, 142.0, 140.9, 128.7, 127.9, 126.8, 107.7, 76.6, 74.9, 37.7, 35.1, 18.3, 18.2, 18.0, 12.6, 12.1, 0.6 ppm. **^{29}Si DEPT NMR** (79 MHz, CDCl$_3$, 298 K, optimized for J = 15.0 Hz): δ = 12.3, −6.6 ppm. **HRMS** (APCI): calculated for $C_{28}H_{49}OSi_2^+$ [M+H]$^+$: 457.3316; Found 457.3314.

(*Z*)-Triisopropyl((1-phenyl-2-(trimethylsilyl)-6-vinylidenedec-1-en-5-yl)oxy)silane (3ai)

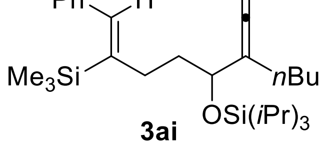

3ai
$C_{30}H_{52}OSi_2$
M = 484.92 g/mol

Prepared from **1a** (63.3 mg, 0.20 mmol) and **2i** (50.5 mg, 0.30 mmol) according to **GP 4**.

Flash column chromatography on silica gel using *n*-pentane as the eluent afforded product **3ai** as a colorless oil (69.0 mg, 71% yield).

R*f* = 0.62 (cyclohexane). **IR** (ATR): ṽ = 3055, 2940, 2864, 2396, 2118, 1955, 1591, 1461, 1247, 1059, 881, 834, 747, 680 cm^{-1}. **^{1}H NMR** (400 MHz, $CDCl_3$, 298 K): *δ* = 7.35–7.30 (m, 2H), 7.28–7.24 (m, 1H), 7.23–7.18 (m, 3H), 4.80–4.68 (m, 2H), 4.39 (t, *J* = 6.7 Hz, 1H), 2.29–2.21 (m, 2H), 2.17–2.07 (m, 1H), 2.02–1.92 (m, 1H), 1.86–1.76 (m, 2H), 1.52–1.39 (m, 4H), 1.15–1.08 (m, 21H), 0.96 (t, *J* = 7.0 Hz, 3H), −0.01 (s, 9H) ppm. **^{13}C{^{1}H} NMR** (101 MHz, $CDCl_3$, 298 K): *δ* = 206.3, 144.6, 141.9, 140.9, 128.7, 127.9, 126.8, 106.1, 76.2, 75.0, 37.6, 35.1, 29.9, 24.7, 22.9, 18.3, 18.2, 14.2, 12.6, 0.6 ppm. **^{29}Si DEPT NMR** (79 MHz, $CDCl_3$, 298 K, optimized for *J* = 15.0 Hz): *δ* = 12.3, −6.6 ppm. **HRMS** (APCI): calculated for $C_{30}H_{53}OSi_2^+$ [M+H]$^+$: 485.3629; Found 485.3628.

(*Z*)-Triisopropyl((8-methyl-1-phenyl-2-(trimethylsilyl)-6-vinylidenenon-1-en-5-yl)oxy)silane (3aj)

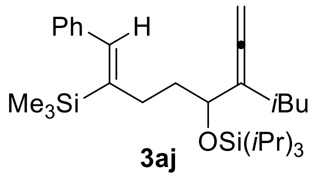

3aj
$C_{30}H_{52}OSi_2$
M = 484.92 g/mol

Prepared from **1a** (63.3 mg, 0.20 mmol) and **2j** (50.5 mg, 0.30 mmol) according to **GP 4**. Flash column chromatography on silica gel using *n*-pentane as the eluent afforded product **3aj** as a colorless oil (72.1 mg, 74% yield).

R*f* = 0.61 (cyclohexane). **IR** (ATR): ṽ = 3055, 2944, 2865, 2330, 2122, 1955, 1591, 1462, 1247, 1059, 881, 834, 747, 680 cm^{-1}. **^{1}H NMR** (400 MHz, $CDCl_3$, 298 K): *δ* = 7.35–7.29 (m, 2H), 7.28–7.24 (m, 1H), 7.23–7.19 (m, 3H), 4.80–4.69 (m, 2H), 4.38 (t, *J* = 6.7 Hz, 1H), 2.30–2.22 (m, 2H), 2.06–1.95 (m, 1H), 1.91–1.77 (m, 4H), 1.16–1.09 (m, 21H), 1.01 (d, *J* = 1.6 Hz, 3H), 0.99 (d, *J* = 1.7 Hz, 3H), −0.01 (s, 9H) ppm. **^{13}C{^{1}H} NMR** (101 MHz, $CDCl_3$, 298 K): *δ* = 206.5, 144.6, 141.9, 140.9, 128.7, 127.9, 126.8, 104.8, 76.0, 75.0, 37.6, 34.89, 34.91, 26.5, 23.2, 23.1, 18.3, 18.2, 12.6, 0.6 ppm. **^{29}Si DEPT NMR** (79 MHz, $CDCl_3$, 298 K, optimized for *J* = 15.0 Hz): *δ* = 12.2, −6.6 ppm. **HRMS** (APCI): calculated for $C_{30}H_{53}OSi_2^+$ [M+H]$^+$: 485.3629; Found 485.3626.

(*Z*)-(6-Benzyl-1-phenyl-5-((triisopropylsilyl)oxy)octa-1,6,7-trien-2-yl)trimethylsilane (3ak)

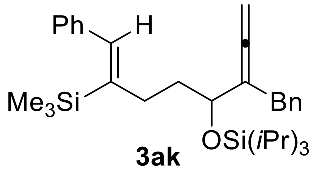

3ak

$C_{33}H_{50}OSi_2$
M = 518.93 g/mol

Prepared from **1a** (63.3 mg, 0.20 mmol) and **2k** (60.7 mg, 0.30 mmol) according to **GP 4**. Flash column chromatography on silica gel using *n*-pentane as the eluent afforded product **3ak** as a colorless oil (67.9 mg, 65% yield).

R*f* = 0.40 (cyclohexane). **IR** (ATR): ṽ = 3026, 2941, 2864, 2653, 2247, 1956, 1593, 1492, 1455, 1247, 1059, 834, 748, 695 cm^{-1}. **^{1}H NMR** (400 MHz, $CDCl_3$, 298 K): δ = 7.39–7.34 (m, 4H), 7.33–7.27 (m, 4H), 7.25–7.22 (m, 2H), 7.13 (s, 1H), 4.74–4.64 (m, 2H), 4.52–4.47 (m, 1H), 3.56–3.48 (m, 1H), 3.44–3.36 (m, 1H), 2.32–2.22 (m, 2H), 1.94–1.80 (m, 2H), 1.23–1.10 (m, 21H), 0.02 (s, 9H) ppm. **^{13}C{^{1}H} NMR** (101 MHz, $CDCl_3$, 298 K): δ = 207.2, 144.3, 142.0, 140.8, 139.9, 129.5, 128.7, 128.2, 127.8, 126.8, 126.1, 106.0, 76.5, 74.6, 37.7, 34.9, 32.9, 18.3, 18.2, 12.6, 0.6 ppm. **^{29}Si DEPT NMR** (79 MHz, $CDCl_3$, 298 K, optimized for J = 15.0 Hz): δ = 12.7, −6.6 ppm. **HRMS** (APCI): calculated for $C_{33}H_{51}OSi_2^+$ $[M+H]^+$: 519.3473; Found 519.3470.

(*Z*)-Triisopropyl((3-isopropyl-8-phenyl-7-(trimethylsilyl)octa-1,2,7-trien-4-yl)oxy)-silane (3al)

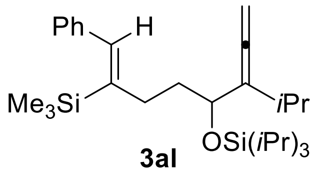

3al

$C_{29}H_{50}OSi_2$
M = 470.89 g/mol

Prepared from **1a** (63.3 mg, 0.20 mmol) and **2l** (46.3 mg, 0.30 mmol) according to **GP 4**. Flash column chromatography on silica gel using *n*-pentane as the eluent afforded product **3al** as a colorless oil (76.6 mg, 81% yield).

R*f* = 0.75 (cyclohexane). **IR** (ATR): ṽ = 3055, 2942, 2865, 2321, 2087, 1951, 1591, 1461, 1247, 1055, 881, 834, 747, 679 cm^{-1}. **^{1}H NMR** (400 MHz, $CDCl_3$, 298 K): δ = 7.35–7.29 (m, 2H), 7.28–7.24 (m, 1H), 7.23–7.19 (m, 3H), 4.86–4.75 (m, 2H), 4.45–4.39 (m, 1H), 2.39–2.32 (m, 1H), 2.32–2.23 (m, 2H), 1.91–1.81 (m, 2H), 1.18–1.09 (m, 27H), −0.01 (s, 9H) ppm. **^{13}C{^{1}H} NMR** (101 MHz, $CDCl_3$, 298 K): δ = 206.1, 144.7, 141.9, 140.9, 128.7, 127.9, 126.8, 112.4, 77.5, 74.4, 38.1, 34.7, 25.7, 23.5, 23.4, 18.32, 18.29, 12.7, 0.6 ppm.

^{29}Si DEPT NMR (79 MHz, $CDCl_3$, 298 K, optimized for J = 15.0 Hz): δ = 11.9, −6.6 ppm. **HRMS** (APCI): calculated for $C_{29}H_{50}OSi_2^+$ $[M]^+$: 470.3395; Found 470.3393.

(*Z*)-(1,6-Diphenyl-5-((triisopropylsilyl)oxy)octa-1,6,7-trien-2-yl)trimethylsilane (3am)

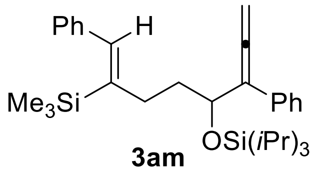

3am

$C_{32}H_{48}OSi_2$

M = 504.91 g/mol

Prepared from **1a** (63.3 mg, 0.20 mmol) and **2m** (56.5 mg, 0.30 mmol) according to **GP 4**. Flash column chromatography on silica gel using *n*-pentane as the eluent afforded product **3am** as a colorless oil (48.3 mg, 48% yield).

R_f = 0.38 (cyclohexane). **IR** (ATR): ṽ = 3023, 2942, 2864, 2312, 2087, 1939, 1704, 1492, 1460, 1247, 1061, 881, 834, 759, 694 cm^{-1}. **^{1}H NMR** (400 MHz, $CDCl_3$, 298 K): δ = 7.62–7.57 (m, 2H), 7.34–7.28 (m, 2H), 7.26–7.17 (m, 4H), 7.14–7.10 (m, 2H), 7.08 (s, 1H), 5.11–5.02 (m, 2H), 4.84–4.77 (m, 1H), 2.32–2.18 (m, 2H), 2.02–1.84 (m, 2H), 1.18–1.04 (m, 21H), −0.16 (s, 9H) ppm. **^{13}C{^{1}H} NMR** (101 MHz, $CDCl_3$, 298 K): δ = 209.5, 144.4, 142.0, 140.8, 134.6, 128.7, 128.4, 128.0, 127.8, 126.9, 126.7, 108.5, 77.8, 74.2, 38.1, 34.8, 18.33, 18.26, 12.6, 0.5 ppm. **^{29}Si DEPT NMR** (79 MHz, $CDCl_3$, 298 K, optimized for J = 15.0 Hz): δ = 13.2, −6.6 ppm. **HRMS** (APCI): calculated for $C_{32}H_{49}OSi_2^+$ $[M+H]^+$: 505.3316; Found 505.3312.

5 Experimental Details for the Silylium-Ion-Promoted Ionic Remote α-C–H Allenylation and Allylation of Silyl Ether 1a

5.1 Procedure for the α-C–H Allenylation of Silyl Ether 1a with Propargylsilane 4a

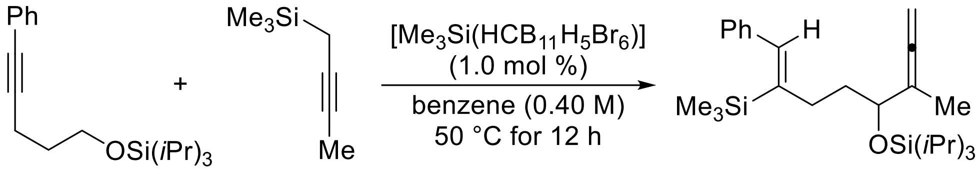

1a **4a** (1.5 equiv) **3aa**: 74% (80%)

In an argon-filled glovebox, silyl ether **1a** (63.1 mg, 0.20 mmol, 1.0 equiv) and propargylsilane **4a** (37.9 mg, 0.30 mmol, 1.5 equiv) were dissolved in benzene (0.5 mL), and the solution was stirred for 1 min. Then, $[Me_3Si(HCB_{11}H_5Br_6)]$ (1.4 mg, 2.0 µmol, 1.0 mol %) was added, and the resulting mixture was stirred at 50 °C for 12 h. Upon completion, the reaction mixture was removed from the glovebox, and all volatiles were evaporated under reduced pressure. CH_2Br_2 (34.8 mg, 0.20 mmol, 1.0 equiv) was subsequently added as an internal standard, and C_6D_6 (0.5 mL) was used as the NMR solvent for NMR spectroscopic analysis, revealing the formation of **3aa** in 80% NMR yield. Purification by flash column chromatography on silica gel using *n*-pentane as the eluent afforded product **3aa** as a colorless oil (65.5 mg, 74% yield) in analytically pure form.
For the characterization data of **3aa**, see **Section 4.2**.

5.2 Procedure for the α-C–H Allylation of Silyl Ether 1a with Allylsilane 5a

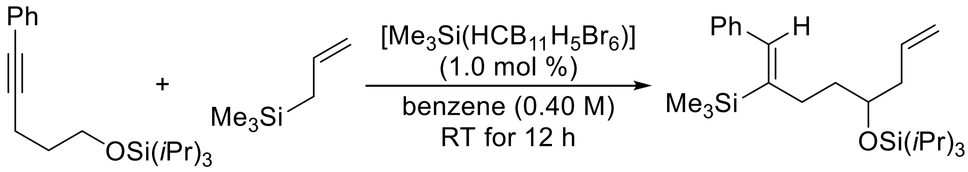

1a (1.2 equiv) **5a** **6aa**: 82% (88%)

In an argon-filled glovebox, silyl ether **1a** (76.0 mg, 0.24 mmol, 1.2 equiv) and allylsilane **5a** (22.9 mg, 0.20 mmol, 1.0 equiv) were dissolved in benzene (0.5 mL), and the solution was stirred for 1 min. Then, $[Me_3Si(HCB_{11}H_5Br_6)]$ (1.4 mg, 2.0 µmol, 1.0 mol %) was added, and the resulting mixture was stirred at room temperature for 12 h. Upon completion, the reaction mixture was removed from the glovebox, and all volatiles were evaporated under reduced pressure. CH_2Br_2 (34.8 mg, 0.20 mmol, 1.0 equiv) was subsequently added as an internal standard, and C_6D_6 (0.5 mL) was used as the NMR solvent for NMR spectroscopic analysis, revealing the formation of **6aa** in 88% NMR yield. Purification by flash column chromatography on silica gel using *n*-pentane as the eluent afforded product **6aa** as a colorless oil (70.7 mg, 82% yield) in analytically pure form.

(*Z*)-Triisopropyl((8-phenyl-7-(trimethylsilyl)octa-1,7-dien-4-yl)oxy)silane (6aa)

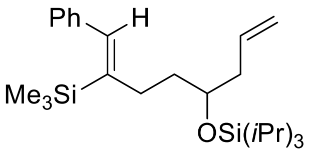

6aa
$C_{26}H_{46}OSi_2$
M = 430.82 g/mol

R*f* = 0.69 (cyclohexane). **IR** (ATR): ṽ = 3076, 2941, 2864, 2565, 2126, 1938, 1639, 1461, 1247, 1099, 1059, 881, 833, 747, 676 cm^{-1}. **^{1}H NMR** (400 MHz, $CDCl_3$, 298 K): *δ* = 7.33–7.27 (m, 2H), 7.27–7.22 (m, 1H), 7.21–7.17 (m, 3H), 5.97–5.83 (m, 1H), 5.17–5.06 (m, 2H), 4.01–3.92 (m, 1H), 2.45–2.23 (m, 4H), 1.77–1.61 (m, 2H), 1.17–1.05 (m, 21H), −0.03 (s, 9H) ppm. **^{13}C{^{1}H} NMR** (101 MHz, $CDCl_3$, 298 K): *δ* = 144.9, 141.7, 140.9, 135.1, 128.7, 127.8, 126.8, 117.1, 72.2, 41.6, 37.7, 34.5, 18.4, 12.8, 0.6 ppm. **^{29}Si DEPT NMR** (79 MHz, $CDCl_3$, 298 K, optimized for *J* = 15.0 Hz): *δ* = 10.5, −6.6 ppm. **HRMS** (APCI): calculated for $C_{26}H_{47}OSi_2^+$ [M+H]$^+$: 431.3160; Found 431.3159.

6 Mechanistic Control Experiments

Synthesis of Deuterated Silyl Ether 1a-*d*₂

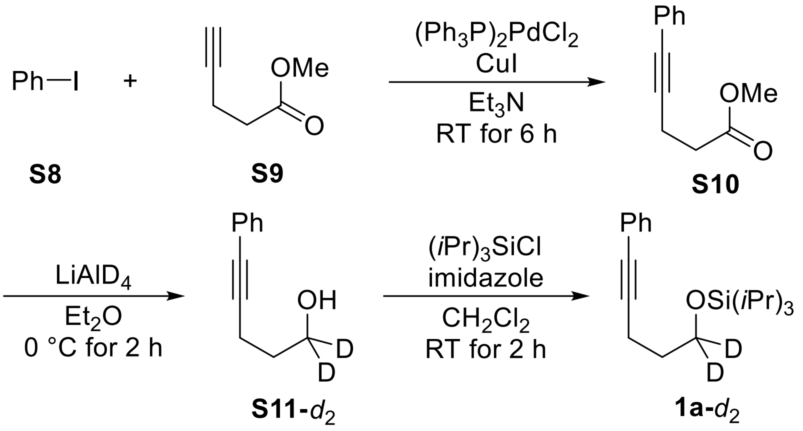

Step 1: According to a reported procedure,[S11] a solution of iodobenzene (**S8**, 1.02 g, 5.0 mmol, 1.0 equiv) and methyl pent-4-ynoate (**S9**, 6.0 mmol, 672.8 mg 1.2 equiv) in Et_3N (5.0 mL) was added dropwise to a suspension of $(Ph_3P)_2PdCl_2$ (70.2 mg, 0.10 mmol, 2.0 mol %) and CuI (28.6 mg, 0.15 mmol, 3.0 mol %) in Et_3N (25 mL). The reaction mixture was stirred at room temperature for additional 6 h. Upon completion (monitored by TLC), the reaction was quenched by the addition of saturated aqueous NH_4Cl solution (15 mL), and the resulting mixture was extracted with CH_2Cl_2 (3 × 20 mL). The combined organic phases were dried over Na_2SO_4 and concentrated under reduced pressure. Purification of the residue by flash column chromatography on silica gel using *n*-pentane and EtOAc as the eluent (50:1→30:1→20:1) afforded methyl 5-phenylpent-4-ynoate (**S10**) as a colorless oil in analytically pure form.

Step 2: According to a reported procedure,[S12] methyl 5-phenylpent-4-ynoate (**S10**, 752.9 mg, 4.0 mmol, 1.0 equiv) was dissolved in Et_2O (40 mL), and the solution was cooled to 0 °C. Then, $LiAlD_4$ (335.8 mg, 8.0 mmol, 2.0 equiv) was added in small portions. The reaction mixture was stirred at 0 °C for additional 2 h. Upon completion (monitored by TLC), the reaction was quenched by the addition of saturated NaOH solution (15 mL), and the resulting mixture was extracted with Et_2O (3 × 20 mL). The combined organic phases were dried over Na_2SO_4 and concentrated under reduced pressure to afford deuterated 5-phenylpent-4-yn-1-ol (**S11**-*d*₂) as a colorless oil, which was directly used in the next step without further purification.

Step 3: Deuterated 5-phenylpent-4-yn-1-ol (**S11**-*d*₂, 648.9 mg, 4.0 mmol, 1.0 equiv) was dissolved in CH_2Cl_2 (40 mL), and imidazole (544.6 mg, 8.0 mmol, 2.0 equiv) was added in one portion. After stirring for 5 min, $(iPr)_3SiCl$ (925.5 mg, 4.8 mmol, 1.2 equiv) was added dropwise to the solution. The reaction mixture was stirred at room temperature for additional 2 h. Upon completion (monitored by TLC), the reaction mixture was poured into water (100 mL), and the organic layer was separated. The aqueous layer was extracted

with CH_2Cl_2 (3 × 20 mL). The combined organic phases were dried over Na_2SO_4 and concentrated under reduced pressure. Purification of the residue by flash column chromatography on silica gel using *n*-pentane as the eluent afforded the deuterated silyl ether **1a**-*d*2 as a colorless oil (1.17 g, 92% yield) in analytically pure form.

Triisopropyl((5-phenylpent-4-yn-1-yl-1,1-*d*2)oxy)silane (1a-*d*2)

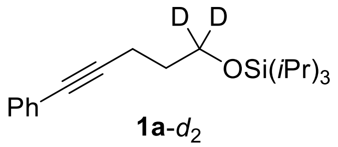

1a-*d*2
$C_{20}H_{30}D_2OSi$
M = 318.57 g/mol

R*f* = 0.54 (cyclohexane). **IR** (ATR): ṽ = 2940, 2864, 2378, 2079, 1872, 1598, 1462, 1150, 1100, 1049, 881, 753, 685 cm^{-1}. **^{1}H NMR** (400 MHz, $CDCl_3$, 298 K): δ = 7.44–7.38 (m, 2H), 7.33–7.27 (m, 3H), 2.56 (t, J = 7.0 Hz, 2H), 1.85 (t, J = 7.0 Hz, 2H), 1.14–1.04 (m, 21H) ppm. **^{13}C{^{1}H} NMR** (101 MHz, $CDCl_3$, 298 K): δ = 131.6, 128.3, 127.6, 124.2, 90.2, 80.7, 31.9, 18.2, 15.9, 12.1 ppm. **^{29}Si DEPT NMR** (99 MHz, $CDCl_3$, 298 K, optimized for J = 15.0 Hz): δ = 12.6 ppm. **HRMS** (APCI): calculated for $C_{20}H_{31}D_2OSi^+$ $[M+H]^+$: 319.2421; Found 319.2420.

***α*-C–H Allenylation of Deuterated Silyl Ether 1a-*d*2 with Allenylsilane 2a**

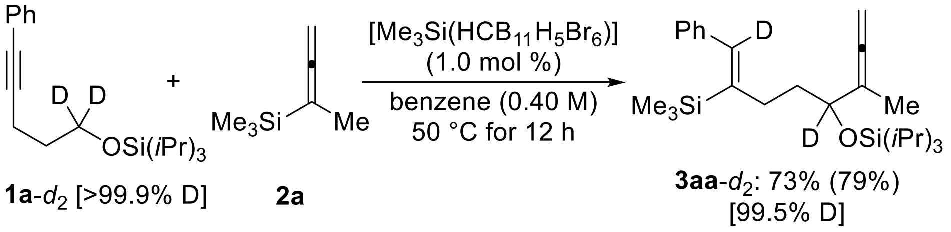

In an argon-filled glovebox, deuterated silyl ether **1a**-*d*2 (63.7 mg, 0.20 mmol, 1.0 equiv) and allenylsilane **2a** (37.9 mg, 0.30 mmol, 1.5 equiv) were dissolved in benzene (0.5 mL), and the solution was stirred for 1 min. Then, $[Me_3Si(HCB_{11}H_5Br_6)]$ (1.4 mg, 2.0 μmol, 1.0 mol %) was added, and the resulting mixture was stirred at 50 °C for 12 h. Upon completion, the reaction mixture was removed from the glovebox, and all volatiles were evaporated under reduced pressure. CH_2Br_2 (34.8 mg, 0.20 mmol, 1.0 equiv) was subsequently added as an internal standard, and C_6D_6 (0.5 mL) was used as the NMR solvent for NMR spectroscopic analysis, revealing the formation of **3aa**-*d*2 in 79% NMR yield. Purification by flash column chromatography on silica gel using *n*-pentane as the eluent afforded product **3aa**-*d*2 as a colorless oil (65.1 mg, 73% yield) in analytically pure form.

(*Z*)-Triisopropyl((3-methyl-8-phenyl-7-(trimethylsilyl)octa-1,2,7-trien-4-yl-4,8-*d*2)oxy)silane (3aa-*d*2)

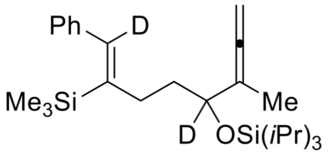

3aa-d_2
$C_{27}H_{44}D_2OSi_2$
M = 444.85 g/mol

R*f* = 0.62 (cyclohexane). **IR** (ATR): ṽ = 3054, 2941, 2864, 2289, 2177, 1958, 1696, 1461, 1248, 1105, 1012, 834, 753, 679 cm^{-1}. **^{1}H NMR** (400 MHz, $CDCl_3$, 298 K): δ = 7.33–7.27 (m, 2H), 7.26–7.23 (m, 1H), 7.22–7.18 (m, 2H), 4.71–4.58 (m, 2H), 2.29–2.17 (m, 2H), 1.85–1.74 (m, 2H), 1.72 (t, J = 3.1 Hz, 3H), 1.15–1.05 (m, 21H), −0.02 (s, 9H) ppm. **^{13}C{^{1}H} NMR** (101 MHz, $CDCl_3$, 298 K): δ = 206.6, 144.3, 141.7 (t, $J_{C,D}$ = 22.4 Hz), 140.7, 128.7, 127.9, 126.8, 100.4, 74.4 (d, $J_{C,D}$ = 21.9 Hz), 74.1, 37.1, 35.0, 18.3, 18.2, 12.5, 12.2, 0.6 ppm. **^{29}Si DEPT NMR** (79 MHz, $CDCl_3$, 298 K, optimized for J = 15.0 Hz): δ = 12.5, −6.6 ppm. **HRMS** (ESI): calculated for $C_{27}H_{45}D_2OSi_2^+$ [M+H]$^+$: 445.3285; Found 445.3285.

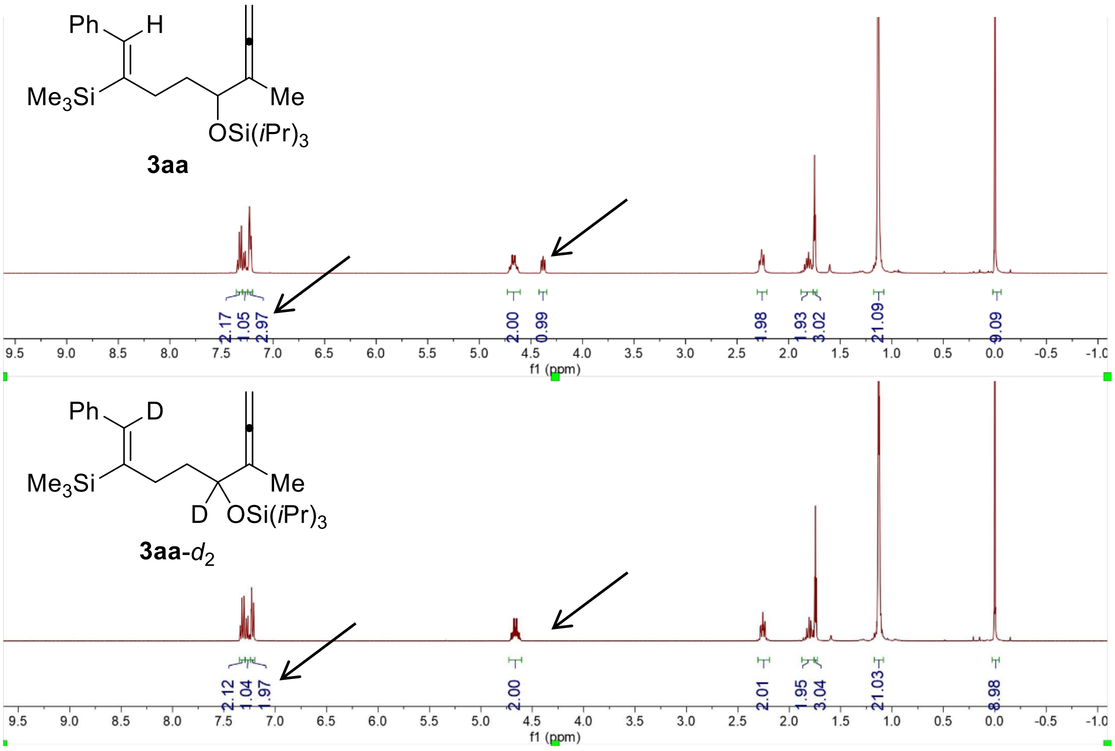

Figure S1. ^{1}H NMR spectra of non-deuterated product **3aa** (top) and deuterated product **3aa**-d_2 (bottom)

Crossover Experiment of Deuterated Silyl Ether 1a-*d*2 and 1s with Allenylsilane 2a

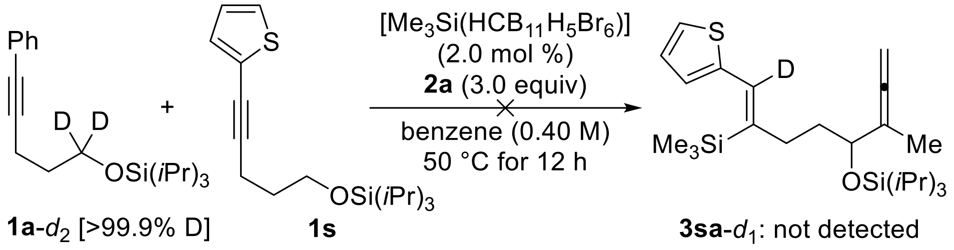

In an argon-filled glovebox, deuterated silyl ether **1a**-*d*2 (63.7 mg, 0.20 mmol, 1.0 equiv), **1s** (64.5 mg, 0.20 mmol, 1.0 equiv) and allenylsilane **2a** (75.8 mg, 0.60 mmol, 3.0 equiv) were dissolved in benzene (1.0 mL), and the solution was stirred for 1 min. Then, [$Me_3Si(HCB_{11}H_5Br_6)$] (2.8 mg, 4.0 μmol, 2.0 mol %) was added, and the resulting mixture was stirred at 50 °C for 12 h. Upon completion, the reaction mixture was removed from the glovebox, and all volatiles were evaporated under reduced pressure. The crude reaction mixture was then dissolved in $CDCl_3$ (0.5 mL) for NMR spectroscopic analysis, and only products **3aa**-*d*2 and **3sa** were obtained (see the bottom spectra). No incorporation of deuterium into the vinylic position of **3sa** was detected.

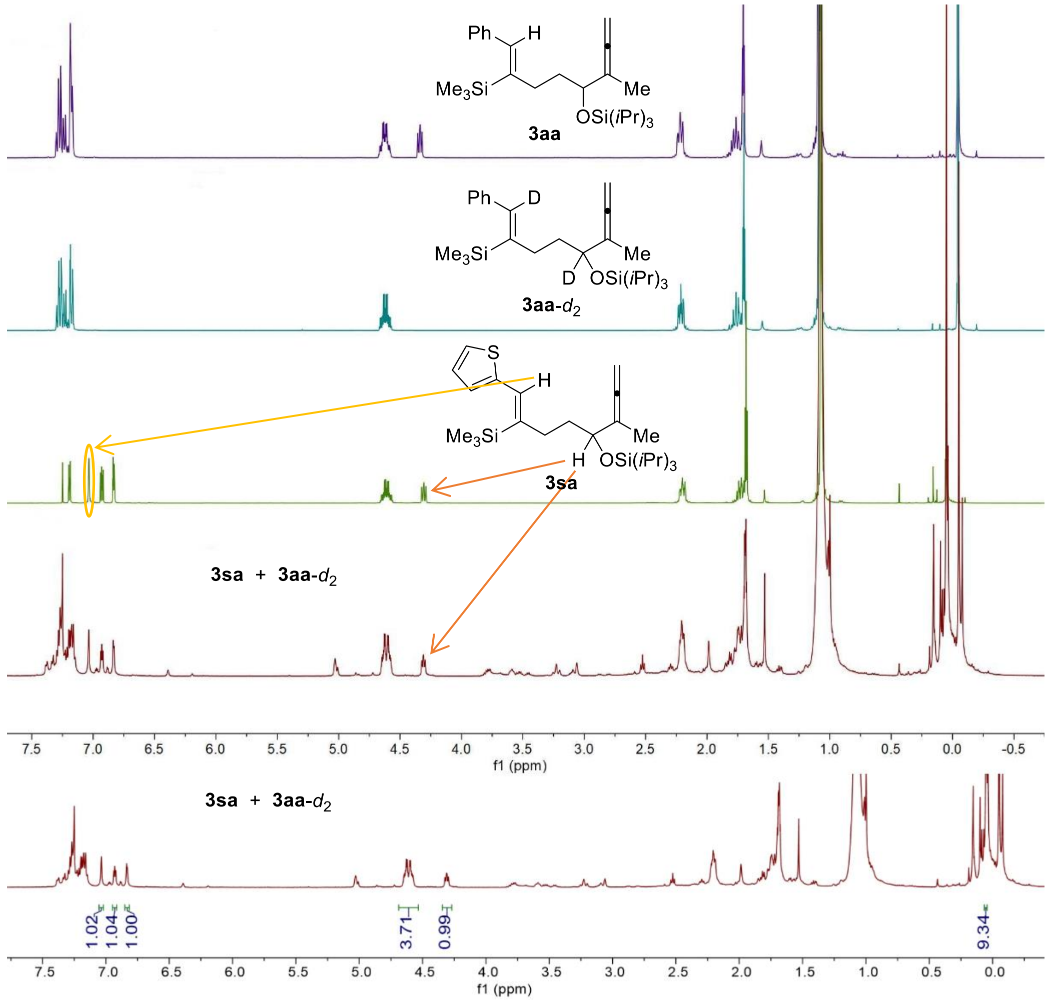

Figure S2. Crude ^{1}H NMR spectra of the crossover experiment reaction mixture (bottom)

Synthesis of Silyl Ethers 7a and 8a

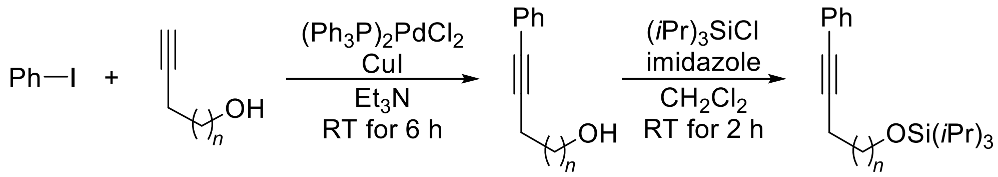

S8 **S12** (n = 1) or **S13** (n = 3) **S14** (n = 1) or **S15** (n = 3) **7a** (n = 1) or **8a** (n = 3)

Step 1: According to a reported procedure,[S6] a solution of iodobenzene (**S8**, 1.02 g, 5.0 mmol, 1.0 equiv) and but-3-yn-1-ol (**S12**) or hex-5-yn-1-ol (**S13**) (6.0 mmol, 1.2 equiv) in Et_3N (5.0 mL) was added dropwise to a suspension of $(Ph_3P)_2PdCl_2$ (70.2 mg, 0.10 mmol, 2.0 mol %) and CuI (28.6 mg, 0.15 mmol, 3.0 mol %) in Et_3N (25 mL). The reaction mixture was stirred at room temperature for additional 6 h. Upon completion (monitored by TLC), the reaction was quenched by the addition of saturated aqueous NH_4Cl solution (15 mL), and the resulting mixture was extracted with CH_2Cl_2 (3 × 20 mL). The combined organic phases were dried over Na_2SO_4 and concentrated under reduced pressure. Purification of the residue by flash column chromatography on silica gel using *n*-pentane and EtOAc as the eluent (20:1→10:1→5:1) afforded 4-phenylbut-3-yn-1-ol (**S14**) or 6-phenylhex-5-yn-1-ol (**S15**) as a colorless oil in analytically pure form.

Step 2: 4-Phenylbut-3-yn-1-ol (**S14**) or 6-phenylhex-5-yn-1-ol (**S15**) (4.0 mmol, 1.0 equiv) was dissolved in CH_2Cl_2 (40 mL), and imidazole (544.6 mg, 8.0 mmol, 2.0 equiv) was added in one portion. After stirring for 5 min, $(i\text{Pr})_3SiCl$ (925.5 mg, 4.8 mmol, 1.2 equiv) was added dropwise to the solution. The reaction mixture was stirred at room temperature for additional 2 h. Upon completion (monitored by TLC), the reaction mixture was poured into water (100 mL), and the organic layer was separated. The aqueous layer was extracted with CH_2Cl_2 (3 × 20 mL). The combined organic phases were dried over Na_2SO_4 and concentrated under reduced pressure. Purification of the residue by flash column chromatography on silica gel using *n*-pentane as the eluent afforded silyl ether **7a** (1.08 g, 89% yield) or **8a** (1.22 g, 92% yield) as a colorless oil in analytically pure form.

Triisopropyl((4-phenylbut-3-yn-1-yl)oxy)silane (7a)

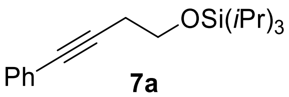

7a

$C_{19}H_{30}OSi$
M = 302.53 g/mol

R*f* = 0.47 (cyclohexane). **IR** (ATR): ṽ = 2940, 2864, 2339, 2008, 1798, 1598, 1462, 1383, 1104, 1069, 880, 753, 685 cm^{-1}. **^{1}H NMR** (400 MHz, $CDCl_3$, 298 K): δ = 7.43–7.37 (m, 2H), 7.32–7.25 (m, 3H), 3.90 (t, J = 7.2 Hz, 2H), 2.67 (t, J = 7.2 Hz, 2H), 1.16–1.04 (m, 21H) ppm. **^{13}C{^{1}H} NMR** (101 MHz, $CDCl_3$, 298 K): δ = 131.7, 128.3, 127.8, 123.9, 87.3, 81.7,

62.3, 24.1, 18.1, 12.1 ppm. **^{29}Si DEPT NMR** (79 MHz, $CDCl_3$, 298 K, optimized for J = 15.0 Hz): δ = 13.4 ppm. **HRMS** (APCI): calculated for $C_{19}H_{31}OSi^+$ $[M+H]^+$: 303.2139; Found 303.2142.

Triisopropyl((6-phenylhex-5-yn-1-yl)oxy)silane (8a)

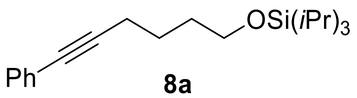

8a
$C_{21}H_{34}OSi$
M = 330.59 g/mol

R$_f$ = 0.41 (cyclohexane). **IR** (ATR): ṽ = 3055, 2940, 2863, 2336, 2087, 1799, 1598, 1461, 1384, 1105, 1067, 881, 753, 686 cm^{-1}. **^{1}H NMR** (400 MHz, $CDCl_3$, 298 K): δ = 7.41–7.35 (m, 2H), 7.31–7.22 (m, 3H), 3.74 (t, J = 6.5 Hz, 2H), 2.44 (t, J = 6.5 Hz, 2H), 1.75–1.64 (m, 4H), 1.11–1.01 (m, 21H) ppm. **^{13}C{^{1}H} NMR** (101 MHz, $CDCl_3$, 298 K): δ = 131.7, 128.3, 127.6, 124.2, 90.4, 80.9, 63.1, 32.3, 25.4, 19.4, 18.2, 12.1 ppm. **^{29}Si DEPT NMR** (79 MHz, $CDCl_3$, 298 K, optimized for J = 15.0 Hz): δ = 12.4 ppm. **HRMS** (APCI): calculated for $C_{21}H_{35}OSi^+$ $[M+H]^+$: 331.2452; Found 331.2451.

Unsuccessful *α*-C–H Allenylation of Silyl Ethers 7a and 8a

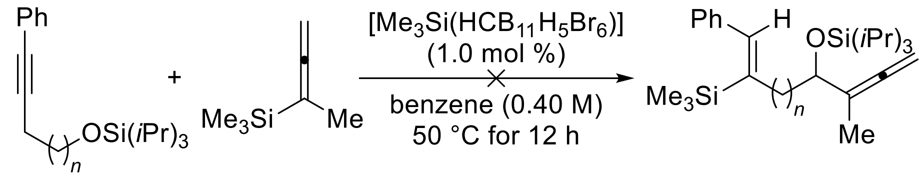

7a (n = 1) or **8a** (n = 3) **2a** Not detected

In an argon-filled glovebox, deuterated silyl ether **7a** (60.5 mg, 0.20 mmol, 1.0 equiv) or **8a** (66.1 mg, 0.20 mmol, 1.0 equiv) and allenylsilane **2a** (37.9 mg, 0.30 mmol, 1.5 equiv) were dissolved in benzene (0.5 mL), and the solution was stirred for 1 min. Then, $[Me_3Si(HCB_{11}H_5Br_6)]$ (1.4 mg, 2.0 µmol, 1.0 mol %) was added, and the resulting mixture was stirred at 50 °C for 12 h. Upon completion, the reaction mixture was removed from the glovebox, and all volatiles were evaporated under reduced pressure. CH_2Br_2 (34.8 mg, 0.20 mmol, 1.0 equiv) was subsequently added as an internal standard, and C_6D_6 (0.5 mL) was used as the NMR solvent for NMR spectroscopic analysis. The desired *α*-C–H allenylation products were not detected for both starting materials.

7 Experimental Details for the Synthetic Transformations of 3aa

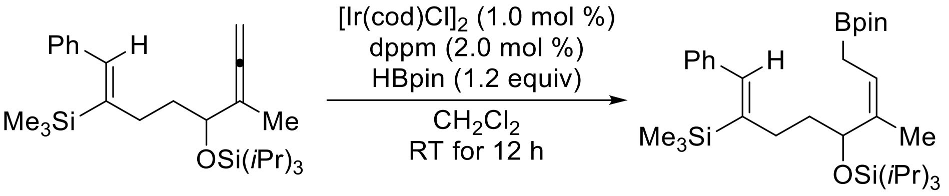

3aa **13**: 72%

$[Ir(cod)Cl]_2$ (1.4 mg, 2.0 μmol, 1.0 mol %) and dppm (1.5 mg, 4.0 μmol, 2.0 mol %) were weighed into an oven-dried Schlenk tube. The tube was sealed, evacuated and backfilled with N_2 for three times before adding CH_2Cl_2 (1.0 mL). Then, HBpin (30.7 mg, 0.24 mmol, 1.2 equiv) was added via a syringe in one portion, and the resulting mixture was stirred for 1 min before adding compound **3aa** (88.6 mg, 0.20 mmol). The reaction was stirred at room temperature for additional 12 h. Upon completion of the reaction (monitored by TLC), all volatiles were evaporated under reduced pressure. Purification of the residue by flash column chromatography on silica gel using *n*-pentane and EtOAc (50:1→30:1) as the eluent afforded hydroboration product **13** as a colorless oil (82.2 mg, 72% yield) in analytically pure form.

Triisopropyl(((2*Z*,7*Z*)-3-methyl-8-phenyl-1-(4,4,5,5-tetramethyl-1,3,2-dioxaborolan-2-yl)-7-(trimethylsilyl)octa-2,7-dien-4-yl)oxy)silane (13)

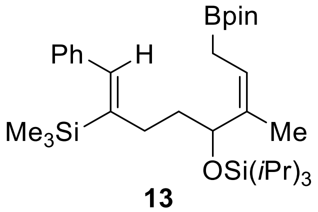

13

$C_{33}H_{59}BO_3Si_2$
M = 570.81 g/mol

R*f* = 0.65 (cyclohexane/EtOAc = 6:1). **IR** (ATR): ṽ = 2941, 2865, 2383, 2002, 1711, 1443, 1343, 1248, 1144, 1060, 835, 675 cm^{-1}. **^{1}H NMR** (400 MHz, $CDCl_3$, 298 K): δ = 7.31–7.26 (m, 2H), 7.25–7.21 (m, 1H), 7.19–7.14 (m, 3H), 5.34 (t, *J* = 7.9 Hz, 1H), 4.69 (dd, *J* = 8.2, 5.7 Hz, 1H), 2.25–2.06 (m, 2H), 1.80–1.64 (m, 7H), 1.25 (s, 12H), 1.11–1.04 (m, 21H), −0.05 (s, 9H) ppm. **^{13}C{^{1}H} NMR** (101 MHz, $CDCl_3$, 298 K): δ = 144.9, 141.7, 140.9, 136.9, 128.7, 127.8, 126.7, 120.5, 83.3, 70.3, 37.9, 35.4, 25.0, 18.3, 18.2, 17.9, 12.6, 0.6 ppm (*Note: the carbon attached to boron was not observed due to the quadrupole broadening caused by the ^{11}B nucleus*). **^{29}Si DEPT NMR** (79 MHz, $CDCl_3$, 298 K, optimized for *J* = 15.0 Hz): δ = 11.5, −6.7 ppm. **^{11}B NMR** (128 MHz, $CDCl_3$, 298 K): δ = 32.9 ppm. **HRMS** (APCI): calculated for $C_{33}H_{59}BO_3Si_2^+$ $[M]^+$: 570.4090; Found 570.4091.

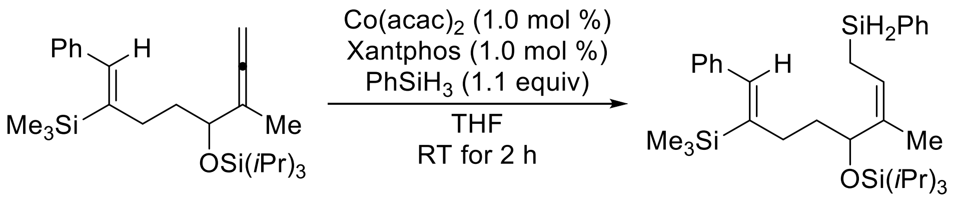

$Co(acac)_2$ (0.5 mg, 2.0 μmol, 1.0 mol %) and Xantphos (1.2 mg, 2.0 μmol, 1.0 mol %) were weighed into an oven-dried Schlenk tube. The tube was sealed, evacuated and backfilled with N_2 for three times before adding THF (1.0 mL). Then, $PhSiH_3$ (23.8 mg, 0.22 mmol, 1.1 equiv) and compound **3aa** (88.6 mg, 0.20 mmol) were added via a syringe in one portion, and the resulting mixture was stirred at room temperature for additional 2 h. Upon completion of the reaction (monitored by TLC), all volatiles were evaporated under reduced pressure. Purification of the residue by flash column chromatography on silica gel using *n*-pentane as the eluent afforded hydrosilylation product **14** as a colorless oil (91.5 mg, 83% yield) in analytically pure form.

Triisopropyl(((2*Z*,7*Z*)-3-methyl-8-phenyl-1-(phenylsilyl)-7-(trimethylsilyl)octa-2,7-dien-4-yl)oxy)silane (14)

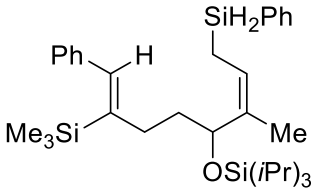

14
$C_{33}H_{54}OSi_3$
M = 551.05 g/mol

R*f* = 0.71 (cyclohexane). **IR** (ATR): ṽ = 3019, 2941, 2863, 2313, 2135, 1591, 1461, 1247, 1060, 832, 697, 679 cm^{-1}. **^{1}H NMR** (400 MHz, $CDCl_3$, 298 K): δ = 7.64–7.59 (m, 2H), 7.48–7.38 (m, 3H), 7.35–7.25 (m, 3H), 7.24–7.19 (m, 2H), 7.17 (s, 1H), 5.31 (t, J = 8.4 Hz, 1H), 4.75 (t, J = 6.7 Hz, 1H), 4.37 (t, J = 3.8 Hz, 2H), 2.34–2.22 (m, 1H), 2.20–2.08 (m, 1H), 2.02–1.92 (m, 1H), 1.91–1.80 (m, 2H), 1.78 (s, 3H), 1.72–1.60 (m, 1H), 1.17–1.05 (m, 21H), −0.01 (s, 9H) ppm. **^{13}C{^{1}H} NMR** (101 MHz, $CDCl_3$, 298 K): δ = 144.7, 141.7, 140.8, 137.6, 135.4, 132.1, 129.9, 128.7, 128.2, 127.8, 126.8, 120.3, 70.4, 38.0, 35.4, 18.35, 18.28, 18.2, 12.6, 11.9, 0.6 ppm. **^{29}Si DEPT NMR** (79 MHz, $CDCl_3$, 298 K, optimized for J = 15.0 Hz): δ = 11.5, −6.6, −32.4 ppm. **HRMS** (APCI): calculated for $C_{33}H_{54}OSi_3^+$ $[M]^+$: 550.3477; Found 550.3477.

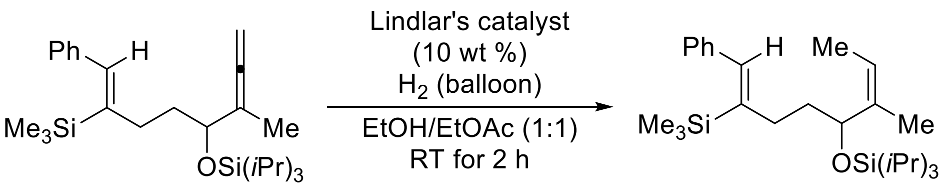

3aa **15**: 92%

Compound **3aa** (44.3 mg, 0.10 mmol) and Lindlar's catalyst (4.4 mg, 10 wt %) were weighed into an oven-dried Schlenk tube. The tube was sealed, evacuated and backfilled with H_2 for three times before adding EtOH (0.5 mL) and EtOAc (0.5 mL mL). The resulting mixture was stirred at room temperature for 2 h. Upon completion of the reaction (monitored by TLC), all volatiles were evaporated under reduced pressure. Purification of the residue by flash column chromatography on silica gel using *n*-pentane as the eluent afforded hydrogenation product **15** as a colorless oil (40.9 mg, 92% yield) in analytically pure form. *(Note: Overreduction of the vinylsilane moiety was observed with a longer reaction time)*

Triisopropyl(((2*Z*,7*Z*)-3-methyl-8-phenyl-7-(trimethylsilyl)octa-2,7-dien-4-yl)oxy)-silane (15)

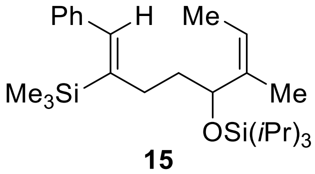

15

$C_{27}H_{48}OSi_2$

M = 444.85 g/mol

R*f* = 0.70 (cyclohexane). **IR** (ATR): ṽ = 2941, 2864, 2560, 2244, 2091, 1707, 1591, 1460, 1378, 1247, 1062, 833, 747, 680 cm^{-1}. **^{1}H NMR** (400 MHz, $CDCl_3$, 298 K): δ = 7.34–7.29 (m, 2H), 7.28–7.24 (m, 1H), 7.23–7.18 (m, 3H), 5.33 (q, *J* = 6.7 Hz, 1H), 4.76 (dd, *J* = 8.4, 5.5 Hz, 1H), 2.29–2.09 (m, 2H), 1.89–1.69 (m, 5H), 1.66 (dd, *J* = 6.8, 1.6 Hz, 3H), 1.15–1.07 (m, 21H), −0.01 (s, 9H) ppm. **^{13}C{^{1}H} NMR** (101 MHz, $CDCl_3$, 298 K): δ = 144.7, 141.6, 140.8, 138.3, 128.7, 127.9, 126.8, 120.1, 70.1, 37.6, 35.1, 18.3, 18.2, 17.8, 13.5, 12.6, 0.6 ppm. **^{29}Si DEPT NMR** (79 MHz, $CDCl_3$, 298 K, optimized for *J* = 15.0 Hz): δ = 11.5, −6.6 ppm. **HRMS** (APCI): calculated for $C_{27}H_{48}OSi_2^+$ [M]$^+$: 444.3238; Found 444.3238.

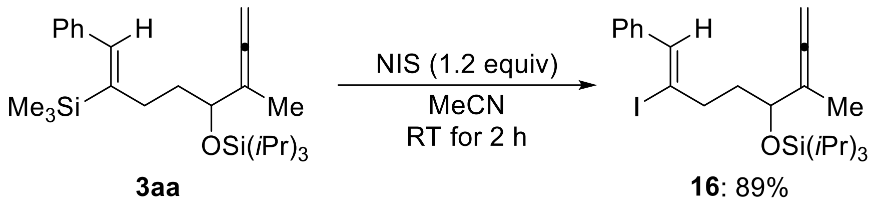

NIS (27.0 mg, 0.12 mmol, 1.2 equiv) was weighed into a Schlenk tube. The tube was sealed, evacuated and backfilled with N_2 for three times before adding compound **3aa** (44.3 mg, 0.10 mmol) and MeCN (1.0 mL). The reaction mixture was stirred at room temperature for 2 h, and then quenched by the addition of saturated $Na_2S_2O_3$ solution (0.5 mL). The organic layer was separated, and the aqueous phase was extracted with CH_2Cl_2 (3 × 5 mL). The combined organic phases were dried over Na_2SO_4, and all volatiles were evaporated under reduced pressure. Purification of the residue by flash column chromatography on silica gel using *n*-pentane as the eluent afforded iododesilylation product **16** as a pale yellow oil (44.2 mg, 89% yield) in analytically pure form.

(*Z*)-((7-Iodo-3-methyl-8-phenylocta-1,2,7-trien-4-yl)oxy)triisopropylsilane (16)

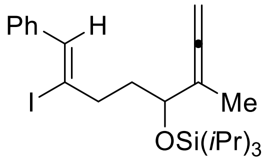

16

$C_{24}H_{37}IOSi$

M = 496.55 g/mol

R*f* = 0.75 (cyclohexane/EtOAc = 10/1). **IR** (ATR): ṽ = 3023, 2940, 2863, 2369, 2074, 1957, 1700, 1461, 1369, 1247, 1058, 881, 843, 745, 679 cm^{-1}. **^{1}H NMR** (500 MHz, $CDCl_3$, 298 K): *δ* = 7.46–7.40 (m, 2H), 7.38–7.32 (m, 2H), 7.31–7.27 (m, 1H), 6.72 (s, 1H), 4.70–4.56 (m, 2H), 4.40–4.33 (m, 1H), 2.70–2.61 (m, 2H), 2.04–1.95 (m, 1H), 1.94–1.84 (m, 1H), 1.71 (t, *J* = 3.1 Hz, 3H) 1.15–1.01 (m, 21H) ppm. **^{13}C{^{1}H} NMR** (126 MHz, $CDCl_3$, 298 K): *δ* = 206.6, 138.7, 134.5, 128.7, 128.2, 127.7, 108.2, 100.2, 74.4, 73.3, 43.1, 35.8, 18.3, 18.2, 12.5, 12.4 ppm. **^{29}Si DEPT NMR** (99 MHz, $CDCl_3$, 298 K, optimized for *J* = 15.0 Hz): *δ* = 13.0 ppm. **HRMS** (APCI): calculated for $C_{24}H_{38}IOSi^+$ [M+H]$^+$: 497.1731; Found 497.1730.

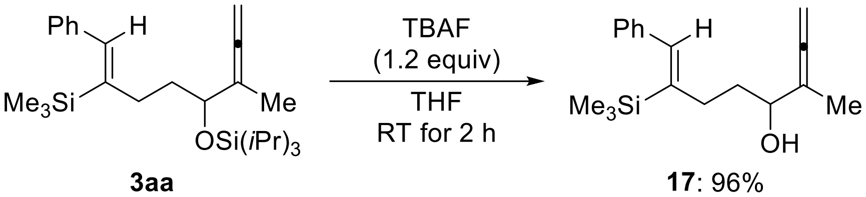

Compound **3aa** (88.6 mg, 0.20 mmol) was weighed into an oven-dried Schlenk tube. The tube was sealed, evacuated and backfilled with N_2 for three times before adding THF (1.0 mL). Next, TBAF (0.24 mL,1.0 M in THF, 0.24 mmol, 1.2 equiv) was added via a syringe in one portion, and the resulting mixture was stirred at room temperature for additional 2 h. Upon completion of the reaction (monitored by TLC), all volatiles were evaporated under reduced pressure. Purification of the residue by flash column chromatography on silica gel using *n*-pentane and EtOAc (50:1→40:1→30:1) as the eluent afforded *α*-allenol **17** as a colorless oil (55.0 mg, 96% yield) in analytically pure form.

(*Z*)-3-Methyl-8-phenyl-7-(trimethylsilyl)octa-1,2,7-trien-4-ol (17)

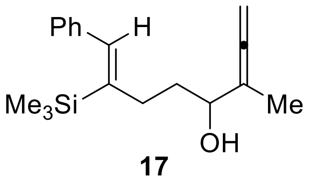

17
$C_{18}H_{26}OSi$
M = 286.49 g/mol

R*f* = 0.69 (cyclohexane/EtOAc = 5/1). **IR** (ATR): ṽ = 3021, 2943, 2863, 2247, 2116, 1957, 1701, 1591, 1440, 1247, 1069, 917, 831, 746, 696 cm^{-1}. **^{1}H NMR** (400 MHz, $CDCl_3$, 298 K): δ = 7.33–7.27 (m, 2H), 7.27–7.22 (m, 2H), 7.21–7.17 (m, 2H), 4.85–4.78 (m, 2H), 4.17–4.10 (m, 1H), 2.49–2.28 (m, 2H), 1.88–1.70 (m, 5H), 1.68 (s, 1H), −0.02 (s, 9H) ppm. **^{13}C{^{1}H} NMR** (101 MHz, $CDCl_3$, 298 K): δ = 205.1, 144.2, 142.4, 140.7, 128.7, 127.9, 126.9, 102.0, 76.9, 72.3, 35.9, 35.0, 14.5, 0.6 ppm. **^{29}Si DEPT NMR** (99 MHz, $CDCl_3$, 298 K, optimized for J = 15.0 Hz): δ = −6.5 ppm. **HRMS** (APCI): calculated for $C_{18}H_{27}OSi^+$ $[M+H]^+$: 287.1826; Found 287.1824.

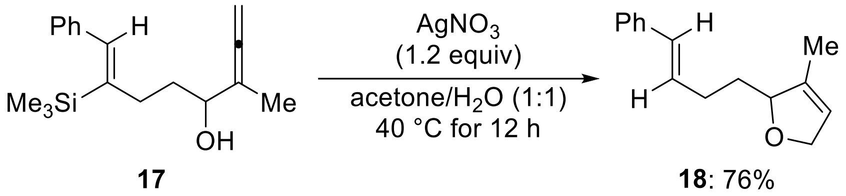

AgNO3 (20.4 mg, 0.12 mmol, 1.2 equiv) was weighed into a Schlenk tube. The tube was sealed, evacuated and backfilled with N_2 for three times before adding acetone (0.5 mL) and H_2O (0.5 mL). Next, *α*-allenol **17** (28.6 mg, 0.10 mmol) was added via a syringe in one portion, and the resulting mixture was stirred at 40 °C for additional 12 h. Upon completion of the reaction (monitored by TLC), the aqueous phase was extracted with CH_2Cl_2 (3 × 5 mL). The combined organic phases were dried over Na_2SO_4, and all volatiles were evaporated under reduced pressure. Purification of the residue by flash column chromatography on silica gel using *n*-pentane and EtOAc (80:1→60:1→50:1) as the eluent afforded 2,5-dihydrofuran **18** as a colorless oil (16.3 mg, 76% yield) in analytically pure form.

(*E*)-3-Methyl-2-(4-phenylbut-3-en-1-yl)-2,5-dihydrofuran (18)

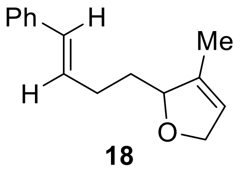

18

$C_{15}H_{18}O$

M = 214.31 g/mol

R*f* = 0.66 (cyclohexane/EtOAc = 8/1). **IR** (ATR): ṽ = 3024, 2919, 2852, 2412, 2121, 1999, 1757, 1663, 1493, 1051, 963, 744, 695 cm^{-1}. **^{1}H NMR** (400 MHz, $CDCl_3$, 298 K): *δ* = 7.36–7.32 (m, 2H), 7.31–7.26 (m, 2H), 7.22–7.16 (m, 1H), 6.45–6.38 (m, 1H), 6.32–6.19 (m, 1H), 5.50 (quint, *J* = 1.7 Hz, 1H), 4.74–4.65 (m, 1H), 4.64–4.52 (m, 2H), 2.35–2.25 (m, 2H), 1.90–1.79 (m, 1H), 1.72 (dt, *J* = 3.1, 1.9 Hz, 3H), 1.65–1.55 (m, 1H) ppm. **^{13}C{^{1}H} NMR** (101 MHz, $CDCl_3$, 298 K): *δ* = 138.0, 137.9, 130.8, 130.1, 128.6, 127.0, 126.1, 120.9, 87.1, 74.7, 33.8, 28.4, 12.6 ppm. **HRMS** (APCI): calculated for $C_{15}H_{19}O^+$ $[M+H]^+$: 215.1430; Found 215.1429.

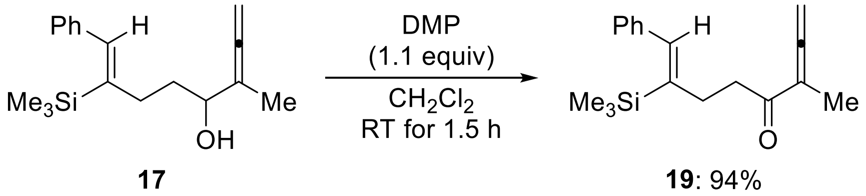

α-Allenol **17** (28.6 mg, 0.10 mmol) was weighed into an oven-dried Schlenk tube. The tube was sealed, evacuated and backfilled with N_2 for three times before adding CH_2Cl_2 (1.0 mL). Next, Dess-Martin periodinane (46.7 mg, 0.11 mmol, 1.1 equiv) was added in one portion, and the resulting mixture was stirred at room temperature for additional 1.5 h. Upon completion of the reaction (monitored by TLC), all volatiles were evaporated under reduced pressure. Purification of the residue by flash column chromatography on silica gel using *n*-pentane and EtOAc (60:1→50:1→40:1) as the eluent afforded ketone product **19** as a colorless oil (26.7 mg, 94% yield) in analytically pure form.

(*Z*)-3-Methyl-8-phenyl-7-(trimethylsilyl)octa-1,2,7-trien-4-one (19)

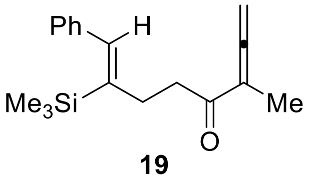

19

$C_{18}H_{24}OSi$

M = 284.47 g/mol

R*f* = 0.71 (cyclohexane/EtOAc = 6/1). **IR** (ATR): ṽ = 3056, 2952, 2895, 2313, 2104, 1934, 1674, 1591, 1441, 1247, 1067, 917, 832, 747, 697 cm^{-1}. **^{1}H NMR** (400 MHz, $CDCl_3$, 298 K): *δ* = 7.32–7.27 (m, 2H), 7.26–7.22 (m, 1H), 7.20–7.15 (m, 3H), 5.19 (q, *J* = 3.0 Hz, 2H), 2.89–2.82 (m, 2H), 2.61–2.51 (m, 2H), 1.83 (t, *J* = 3.0 Hz, 3H), −0.03 (s, 9H) ppm. **^{13}C{^{1}H} NMR** (101 MHz, $CDCl_3$, 298 K): *δ* = 216.5, 201.0, 143.4, 142.2, 140.5, 128.7, 127.9, 126.9, 103.8, 78.8, 39.6, 33.7, 13.3, 0.5 ppm. **^{29}Si DEPT NMR** (99 MHz, $CDCl_3$, 298 K, optimized for *J* = 15.0 Hz): *δ* = −6.3 ppm. **HRMS** (APCI): calculated for $C_{18}H_{25}OSi^+$ [M+H]$^+$: 285.1669; Found 285.1668.

8 Determination of the Alkene Configuration

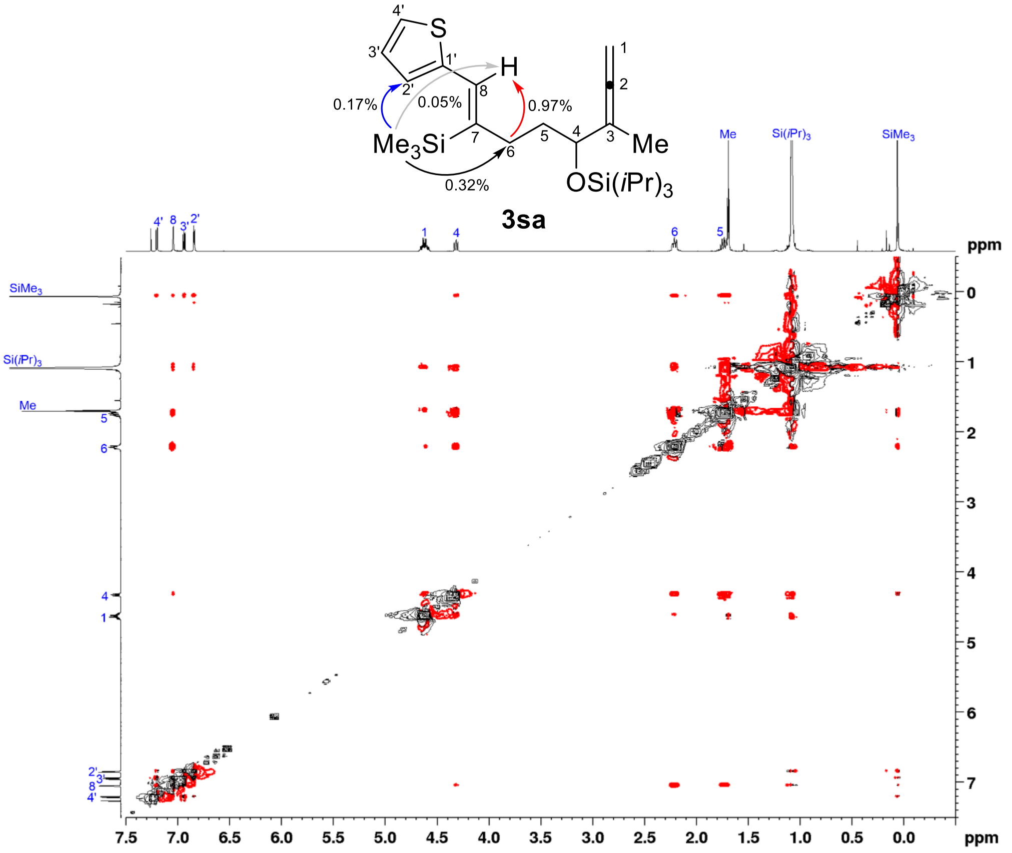

Figure S3. Enhancement factors of selected NOEs taken from a NOESY measurement

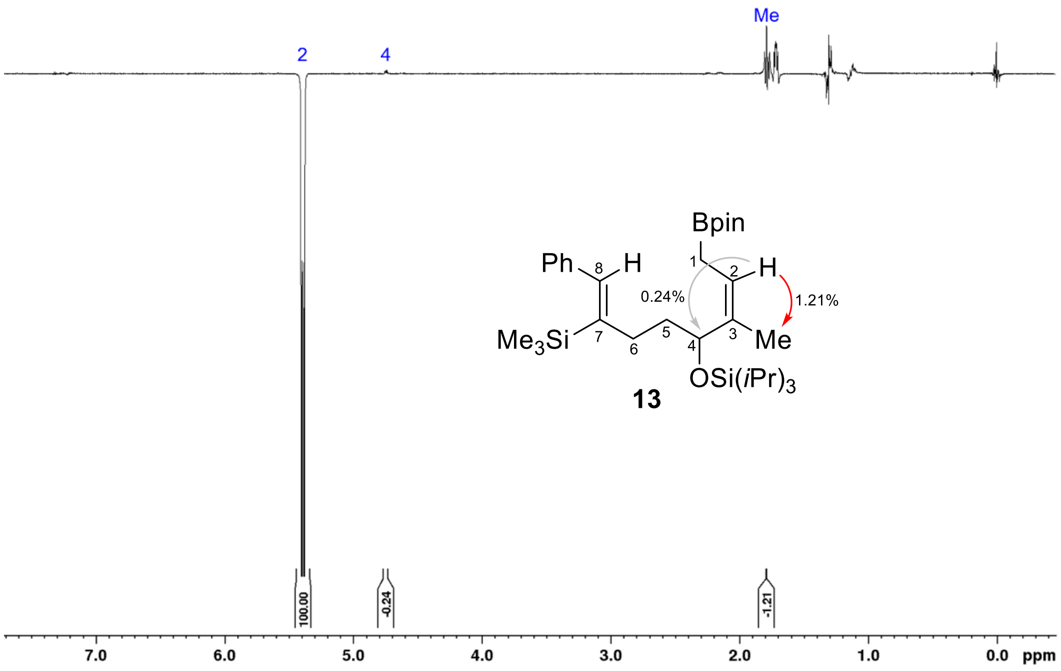

Figure S4. Enhancement factors of selected NOEs taken from a 1D selective NOE measurement with irradiation on H-2 at 5.4 ppm

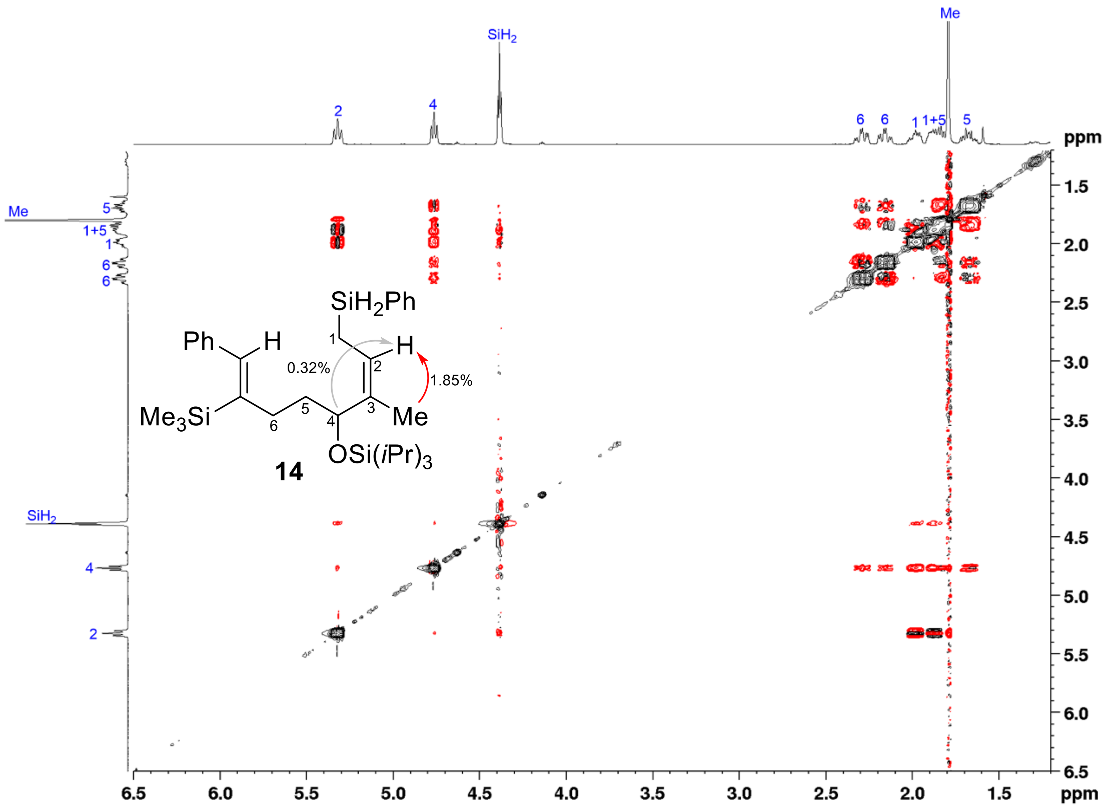

Figure S5. Enhancement factors of selected NOEs taken from a NOESY measurement

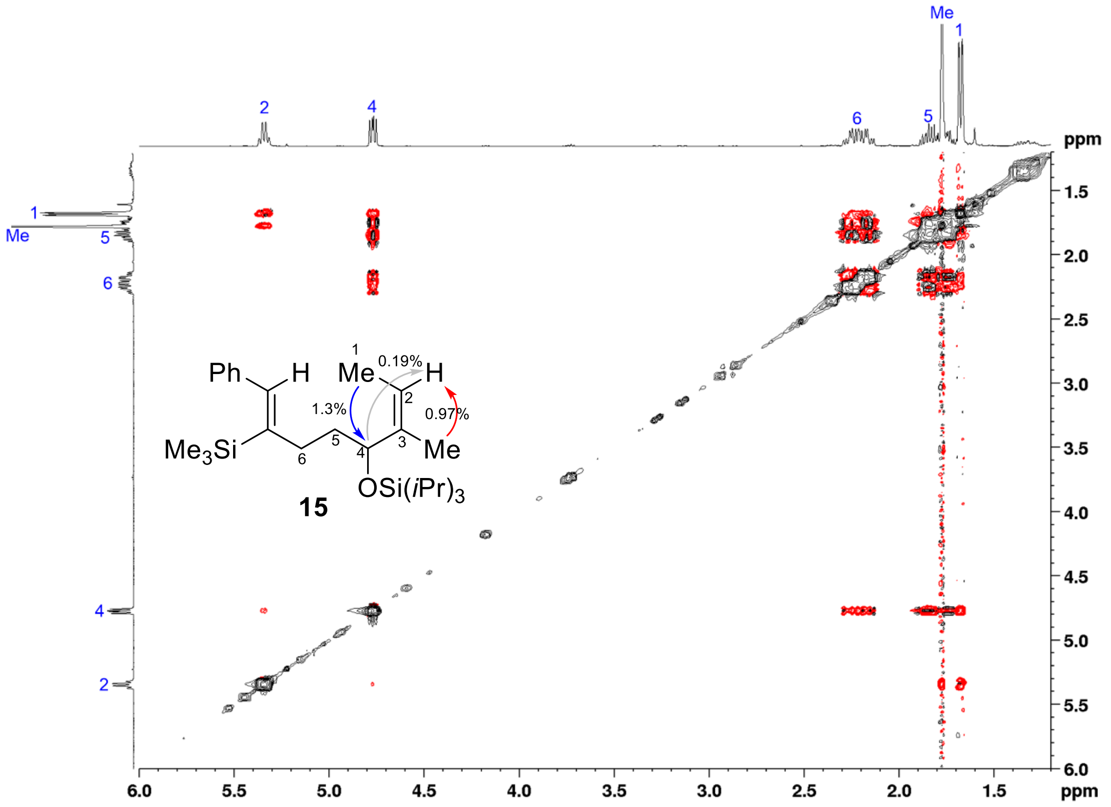

Figure S6. Enhancement factors of selected NOEs taken from a NOESY measurement

9 NMR Spectra

Figure S7. ^{1}H NMR spectrum (400 MHz, $CDCl_3$, 298 K) of triisopropyl((5-phenylpent-4-yn-1-yl)oxy)silane (**1a**)

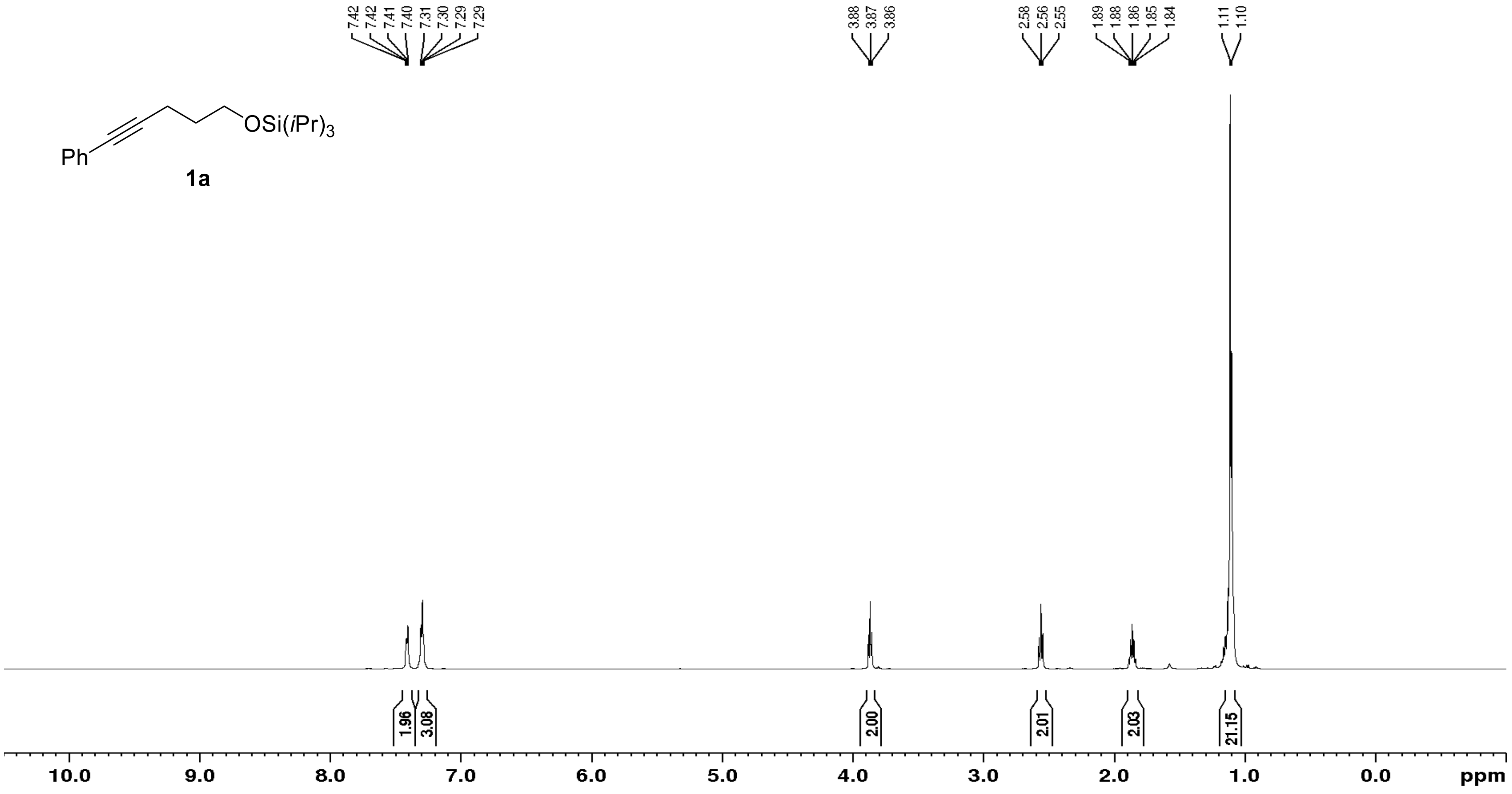

Figure S8. $^{13}C\{^{1}H\}$ NMR spectrum (101 MHz, $CDCl_3$, 298 K) of triisopropyl((5-phenylpent-4-yn-1-yl)oxy)silane (**1a**)

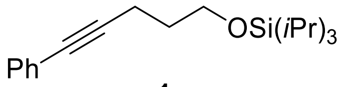

1a

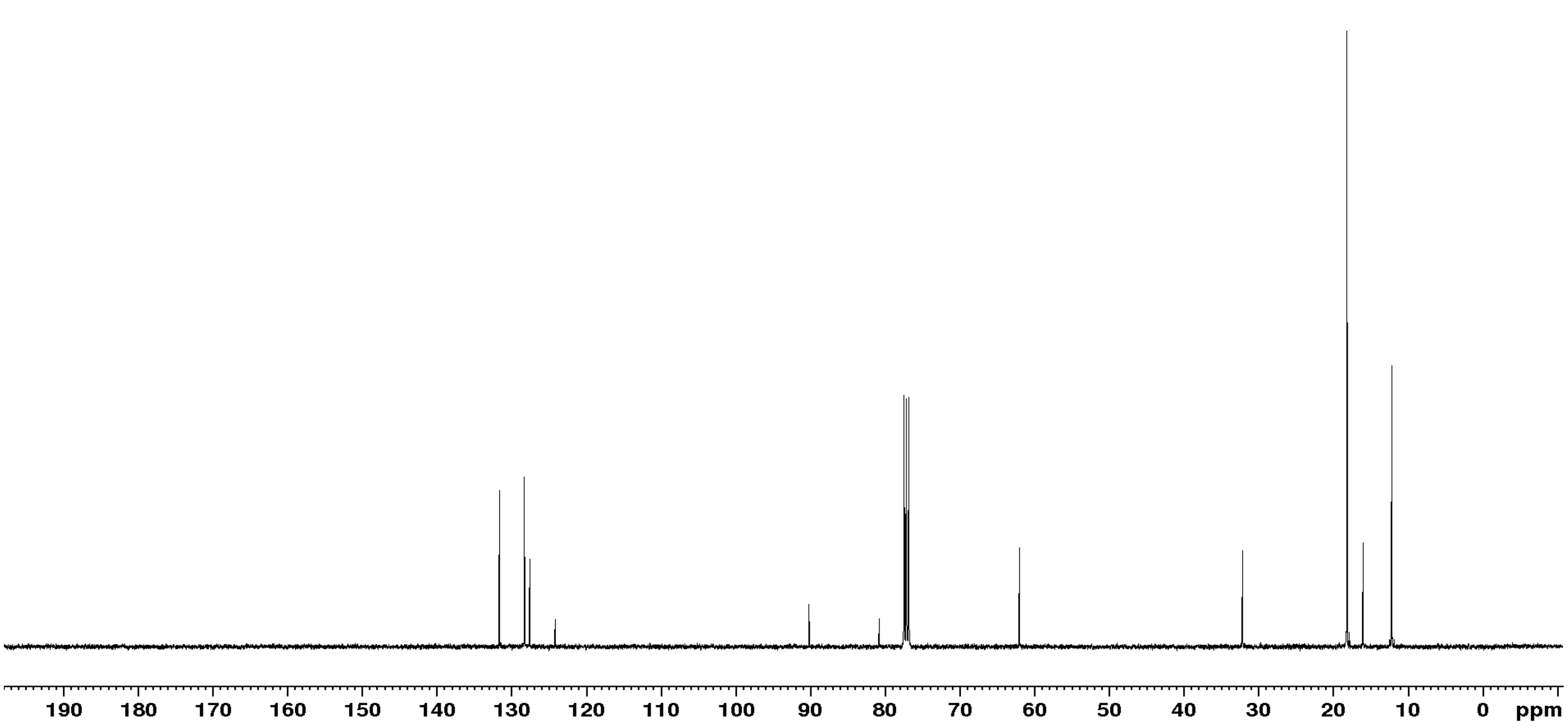

Figure S9. ^{29}Si DEPT NMR spectrum (79 MHz, $CDCl_3$, 298 K, optimized for J = 15.0 Hz) of triisopropyl((5-phenylpent-4-yn-1-yl)oxy)silane (**1a**)

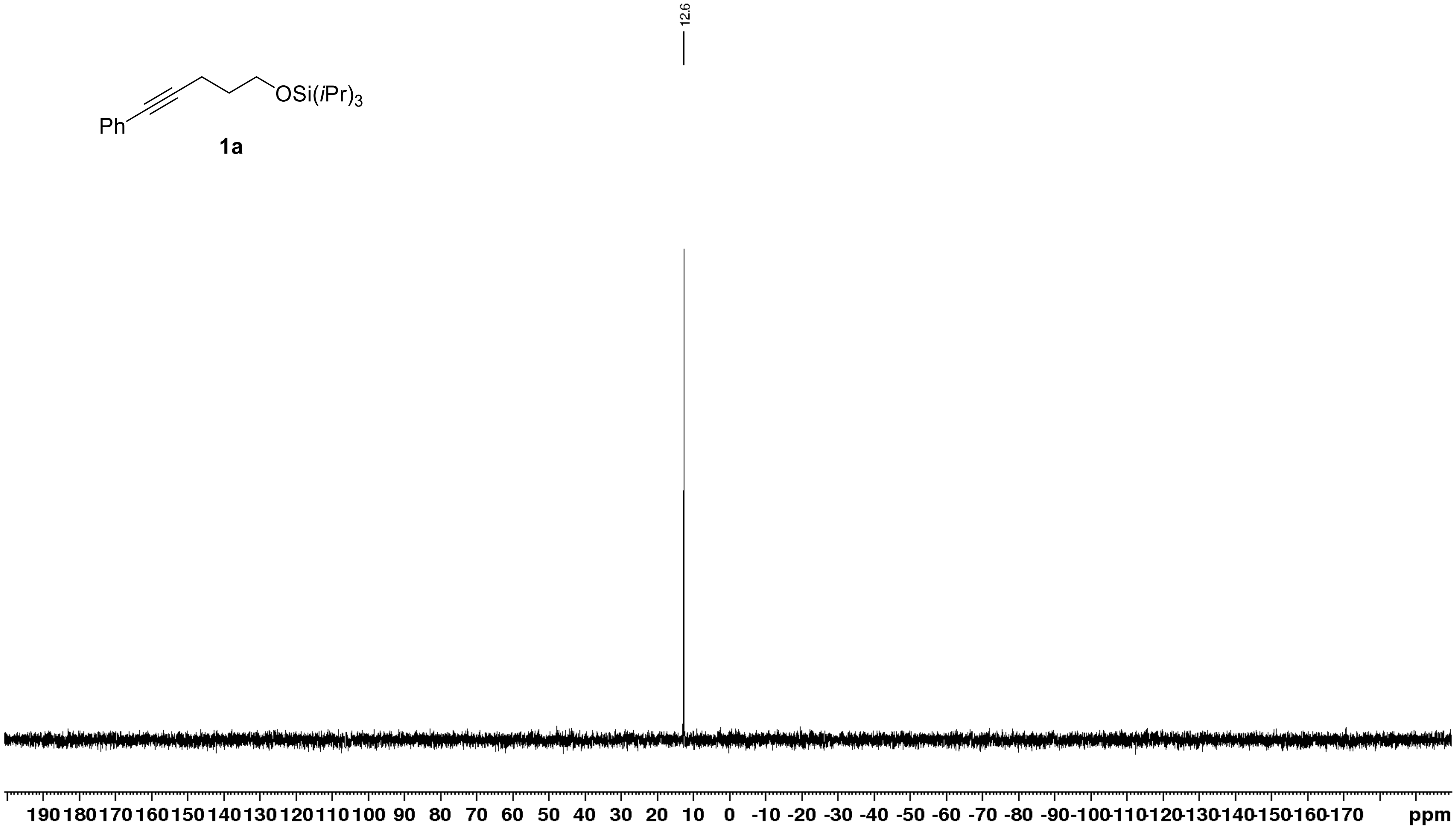

1a

Figure S10. ^{1}H NMR spectrum (500 MHz, $CDCl_3$, 298 K) of triisopropyl((5-(*o*-tolyl)pent-4-yn-1-yl)oxy)silane (**1b**)

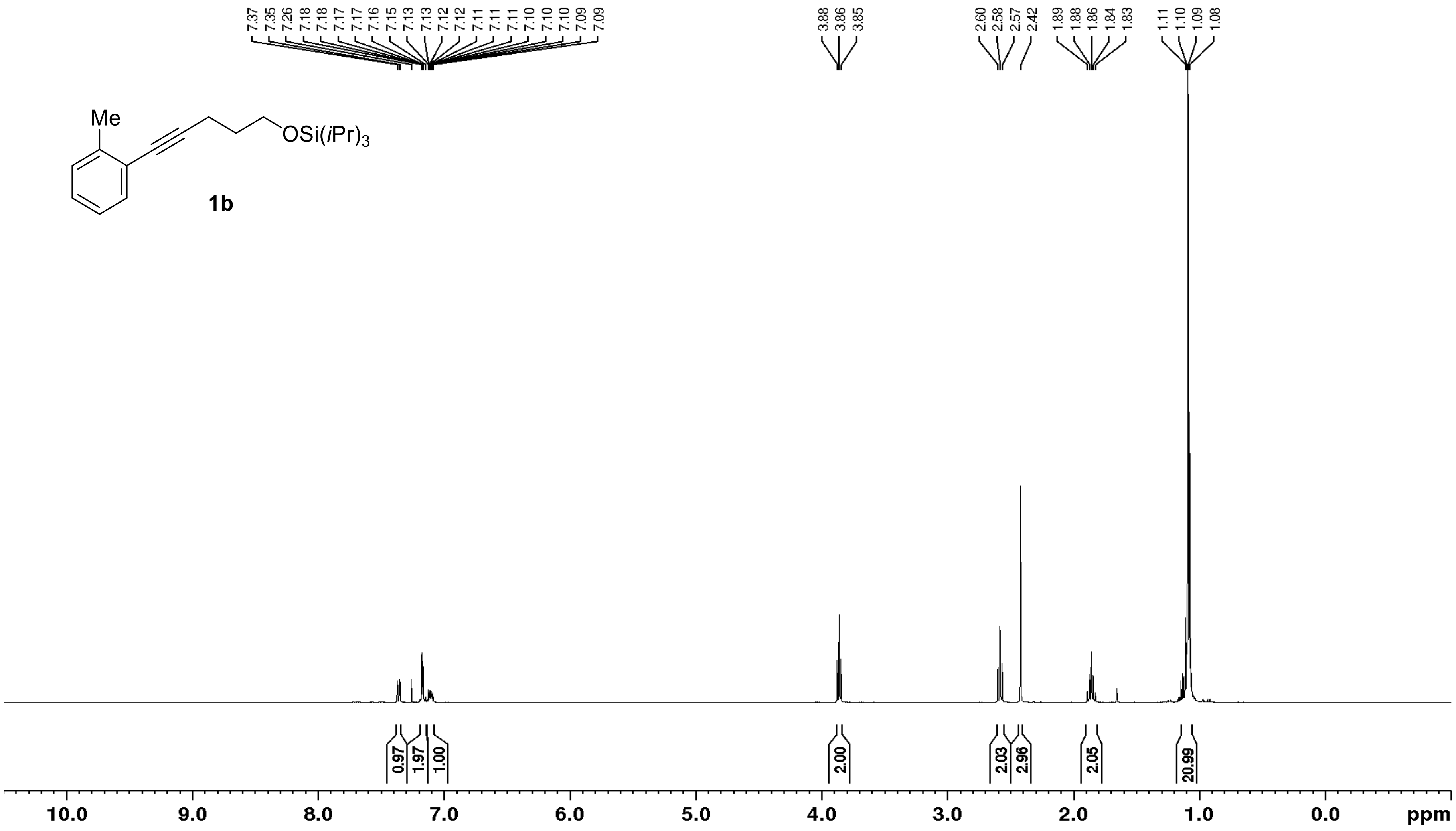

Figure S11. $^{13}C\{^1H\}$ NMR spectrum (126 MHz, $CDCl_3$, 298 K) of triisopropyl((5-(*o*-tolyl)pent-4-yn-1-yl)oxy)silane (**1b**)

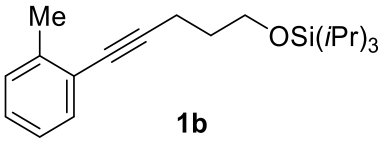

1b

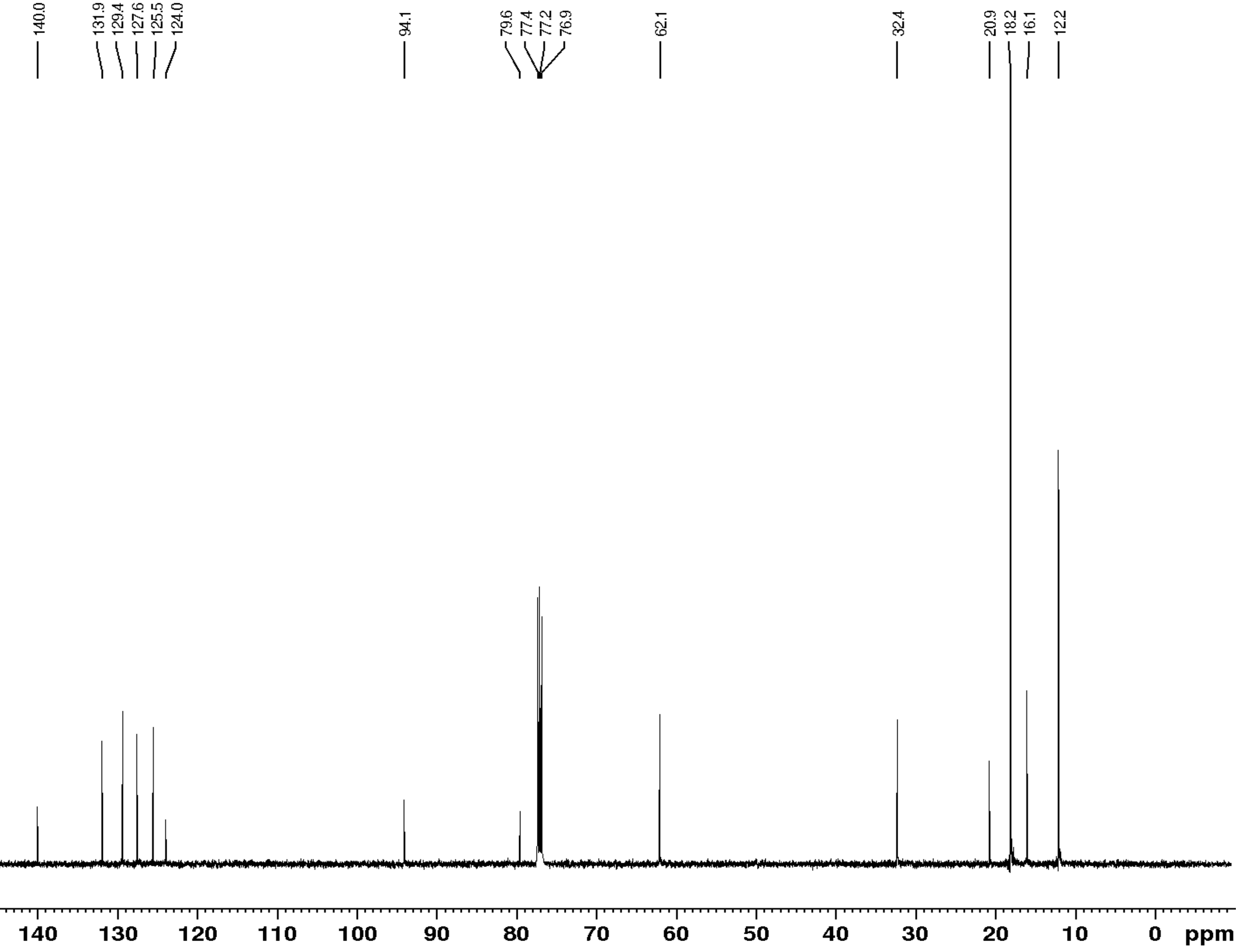

Figure S12. ^{29}Si DEPT NMR spectrum (99 MHz, $CDCl_3$, 298 K, optimized for *J* = 15.0 Hz) of triisopropyl((5-(*o*-tolyl)pent-4-yn-1-yl)oxy)silane (**1b**)

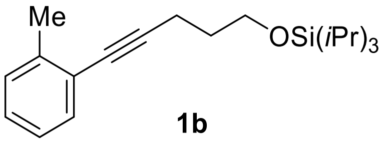

1b

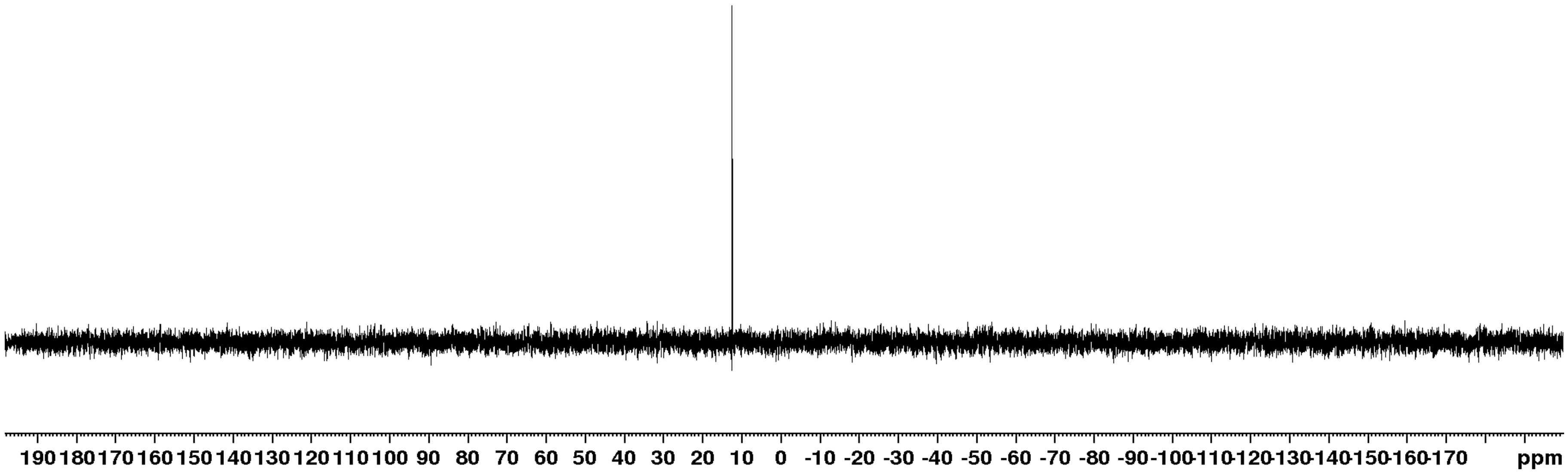

Figure S13. ^{1}H NMR spectrum (400 MHz, $CDCl_3$, 298 K) of triisopropyl((5-(*m*-tolyl)pent-4-yn-1-yl)oxy)silane (**1c**)

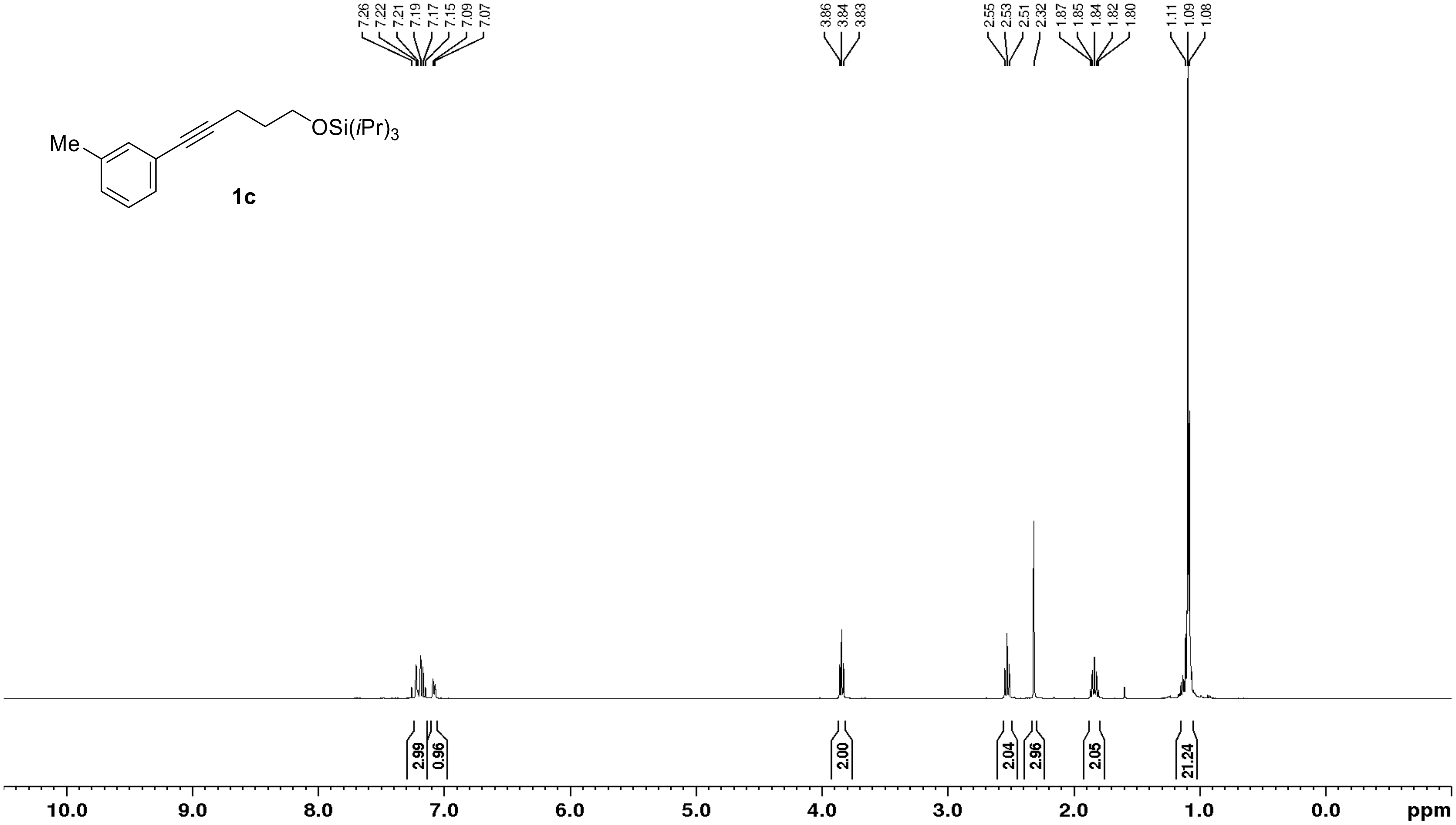

Figure S14. $^{13}C\{^1H\}$ NMR spectrum (101 MHz, $CDCl_3$, 298 K) of triisopropyl((5-(*m*-tolyl)pent-4-yn-1-yl)oxy)silane (**1c**)

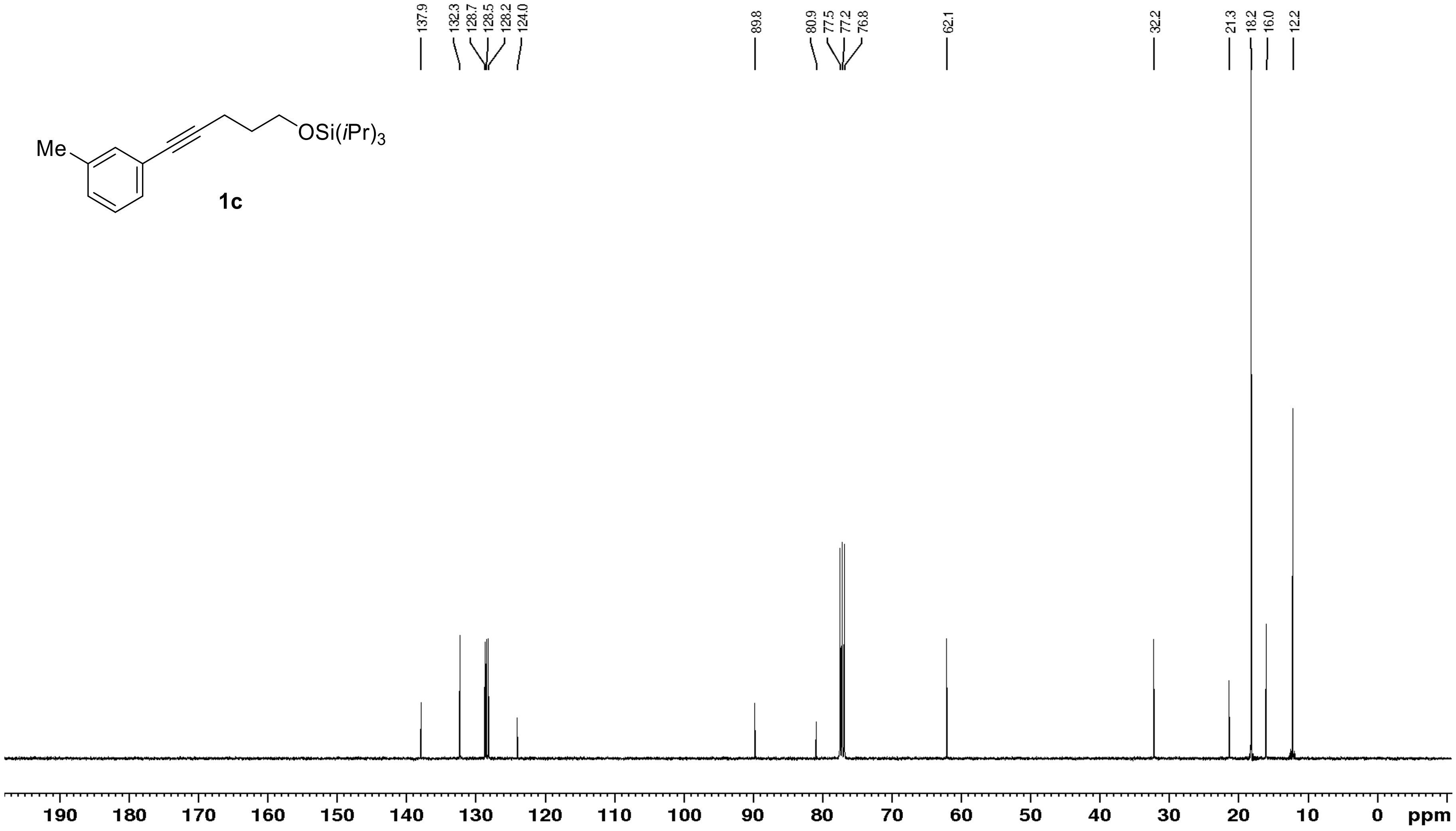

Figure S15. ^{29}Si DEPT NMR spectrum (79 MHz, $CDCl_3$, 298 K, optimized for J = 15.0 Hz) of triisopropyl((5-(*m*-tolyl)pent-4-yn-1-yl)oxy)silane (**1c**)

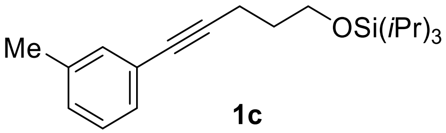

1c

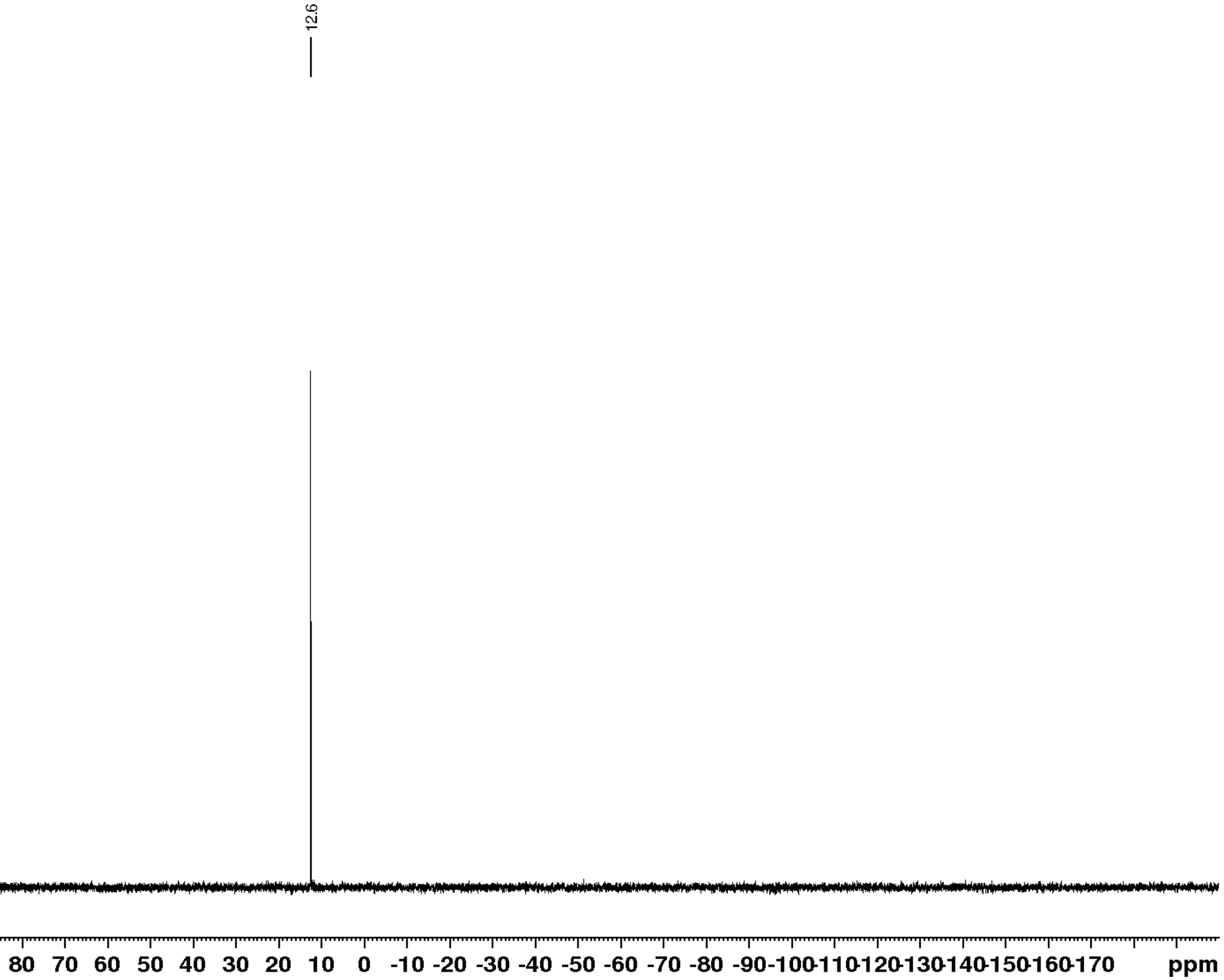

Figure S16. ^{1}H NMR spectrum (400 MHz, $CDCl_3$, 298 K) of triisopropyl((5-(*p*-tolyl)pent-4-yn-1-yl)oxy)silane (**1d**)

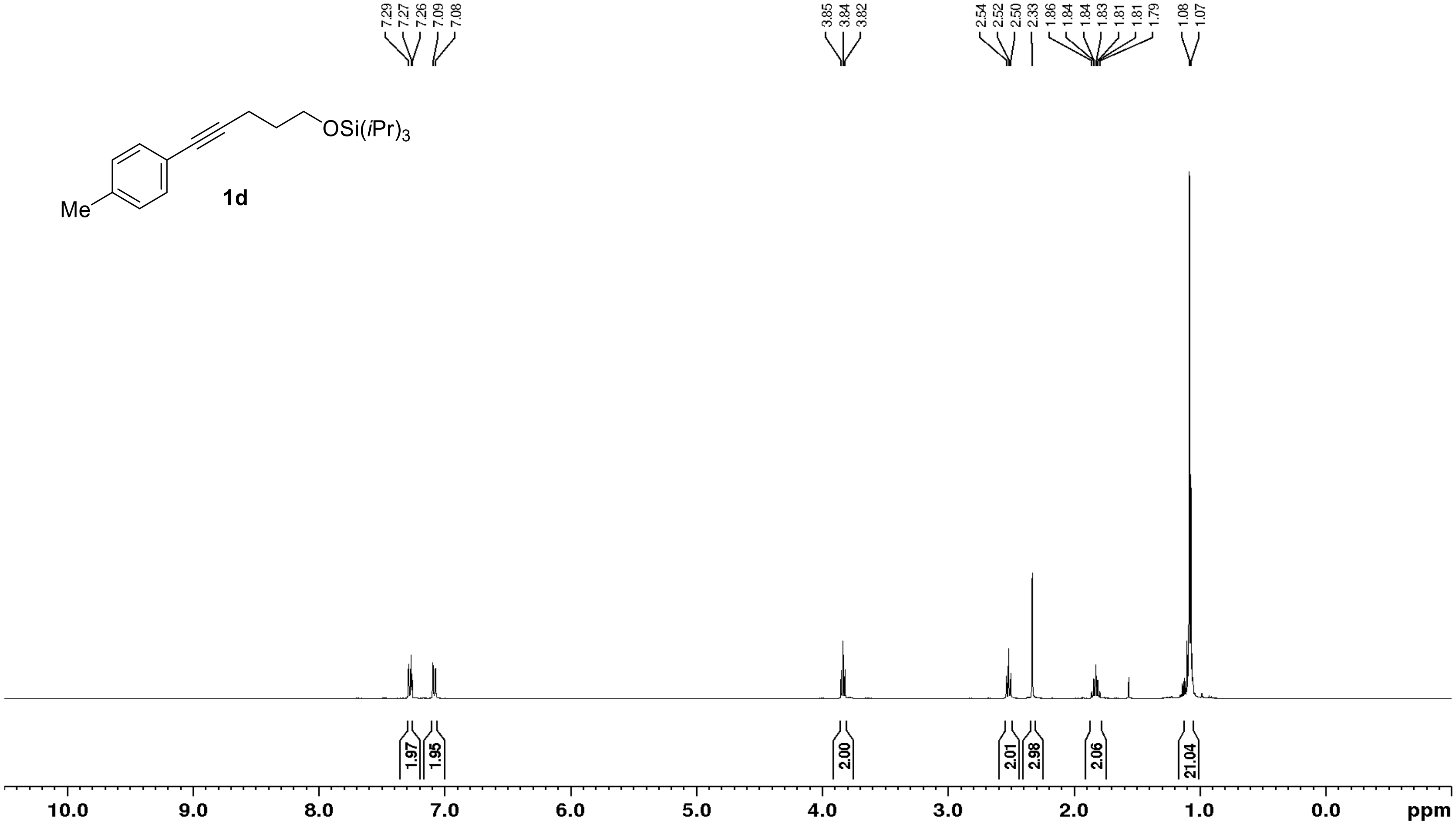

Figure S17. $^{13}C\{^{1}H\}$ NMR spectrum (101 MHz, $CDCl_3$, 298 K) of triisopropyl((5-(*p*-tolyl)pent-4-yn-1-yl)oxy)silane (**1d**)

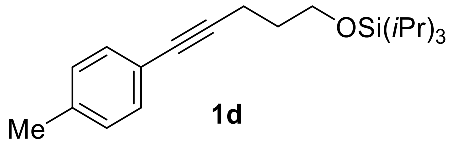

1d

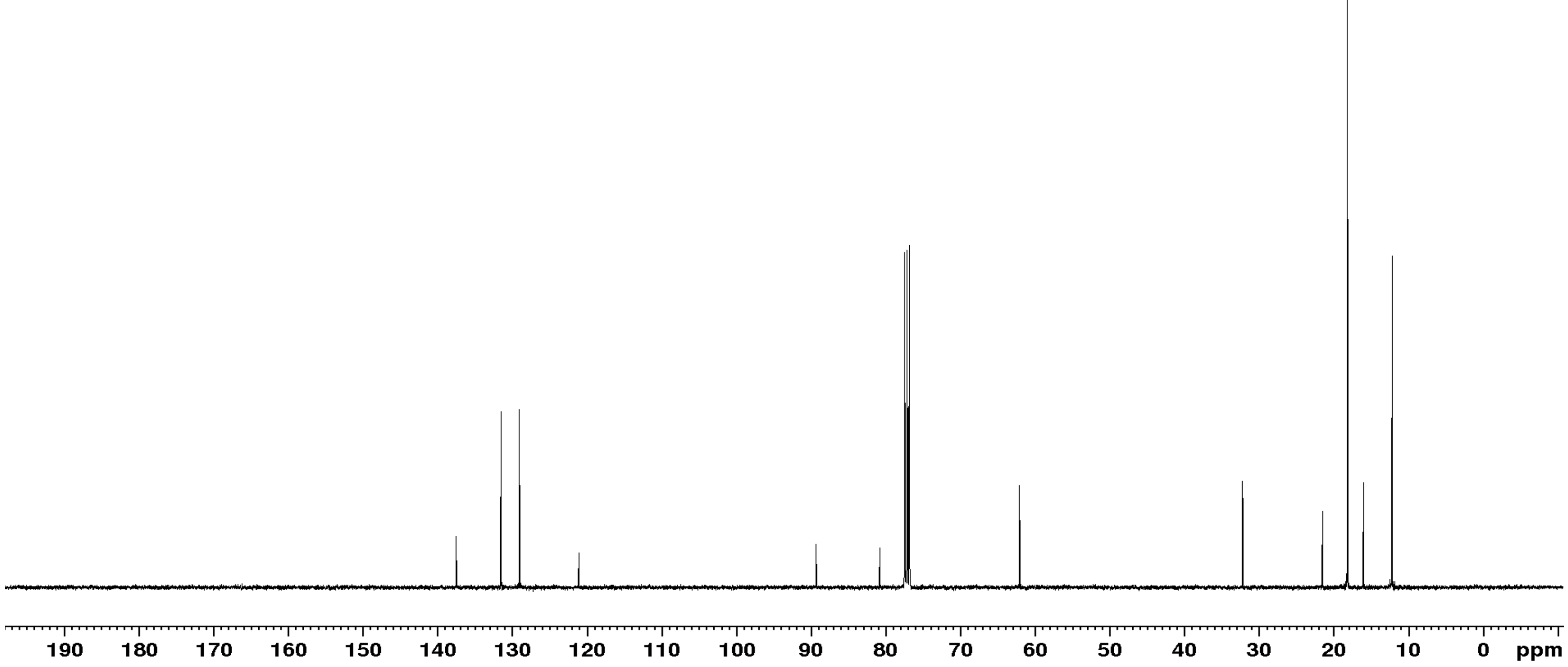

Figure S18. ^{29}Si DEPT NMR spectrum (79 MHz, $CDCl_3$, 298 K, optimized for *J* = 15.0 Hz) of triisopropyl((5-(*p*-tolyl)pent-4-yn-1-yl)oxy)silane (**1d**)

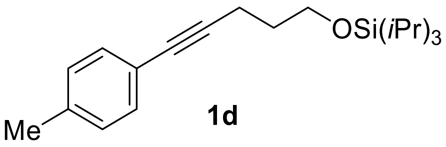

1d

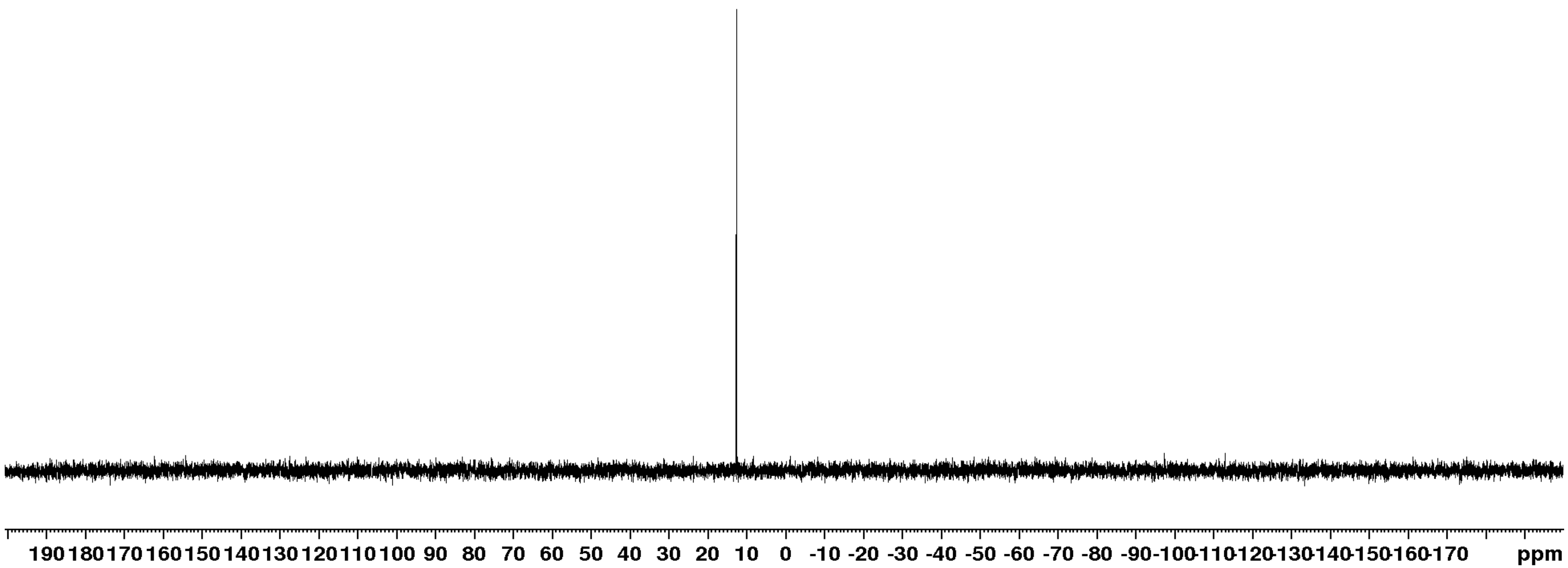

Figure S19. ^{1}H NMR spectrum (400 MHz, $CDCl_3$, 298 K) of ((5-(4-(*tert*-butyl)phenyl)pent-4-yn-1-yl)oxy)triisopropylsilane (**1e**)

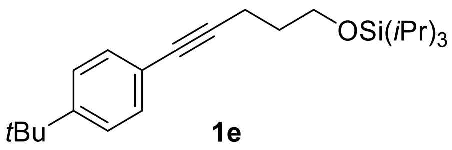

1e

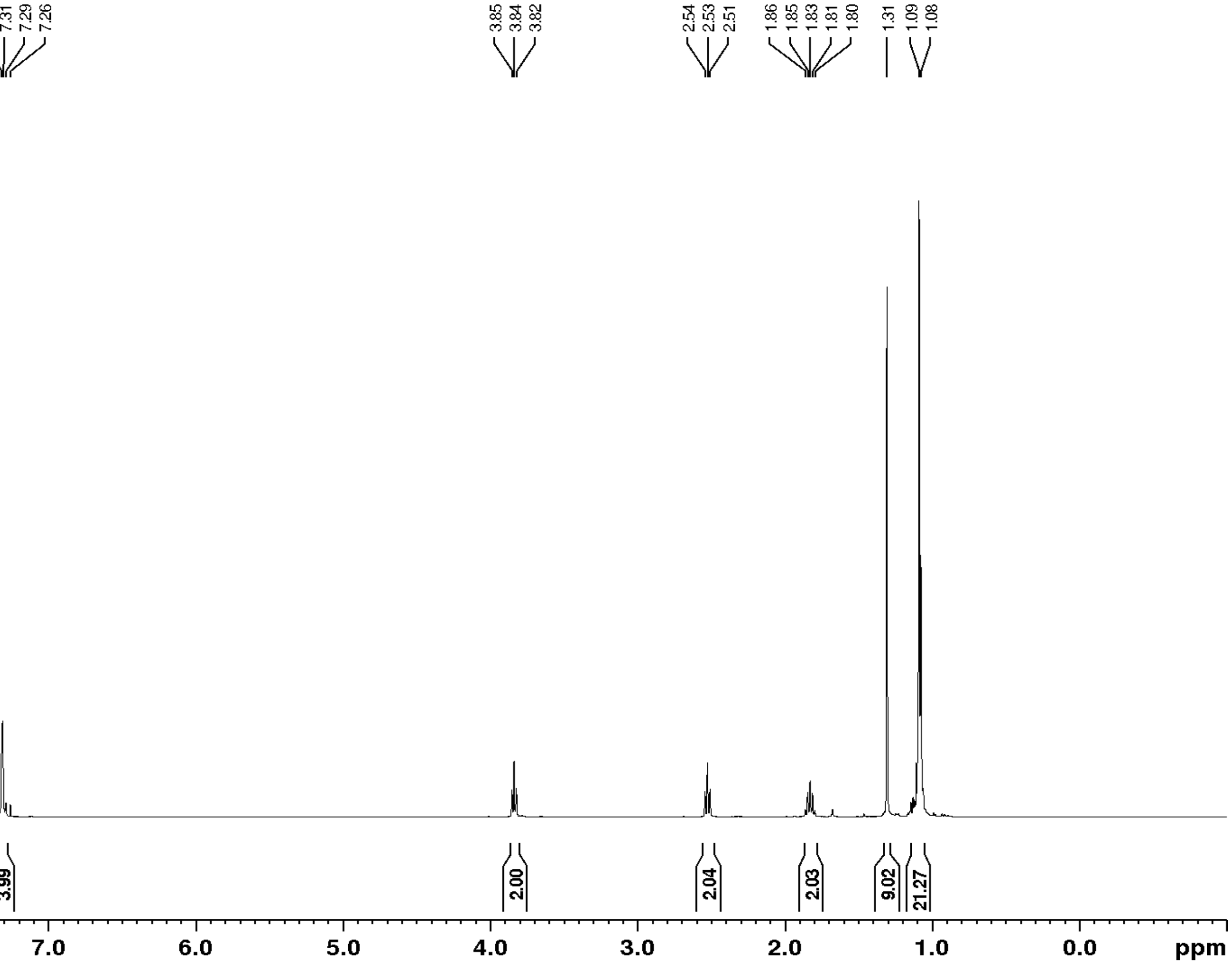

Figure S20. $^{13}C\{^1H\}$ NMR spectrum (101 MHz, $CDCl_3$, 298 K) of ((5-(4-(*tert*-butyl)phenyl)pent-4-yn-1-yl)oxy)triisopropylsilane (**1e**)

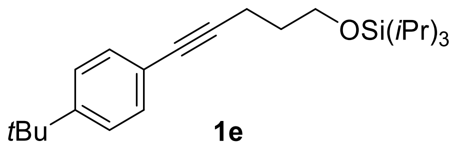

1e

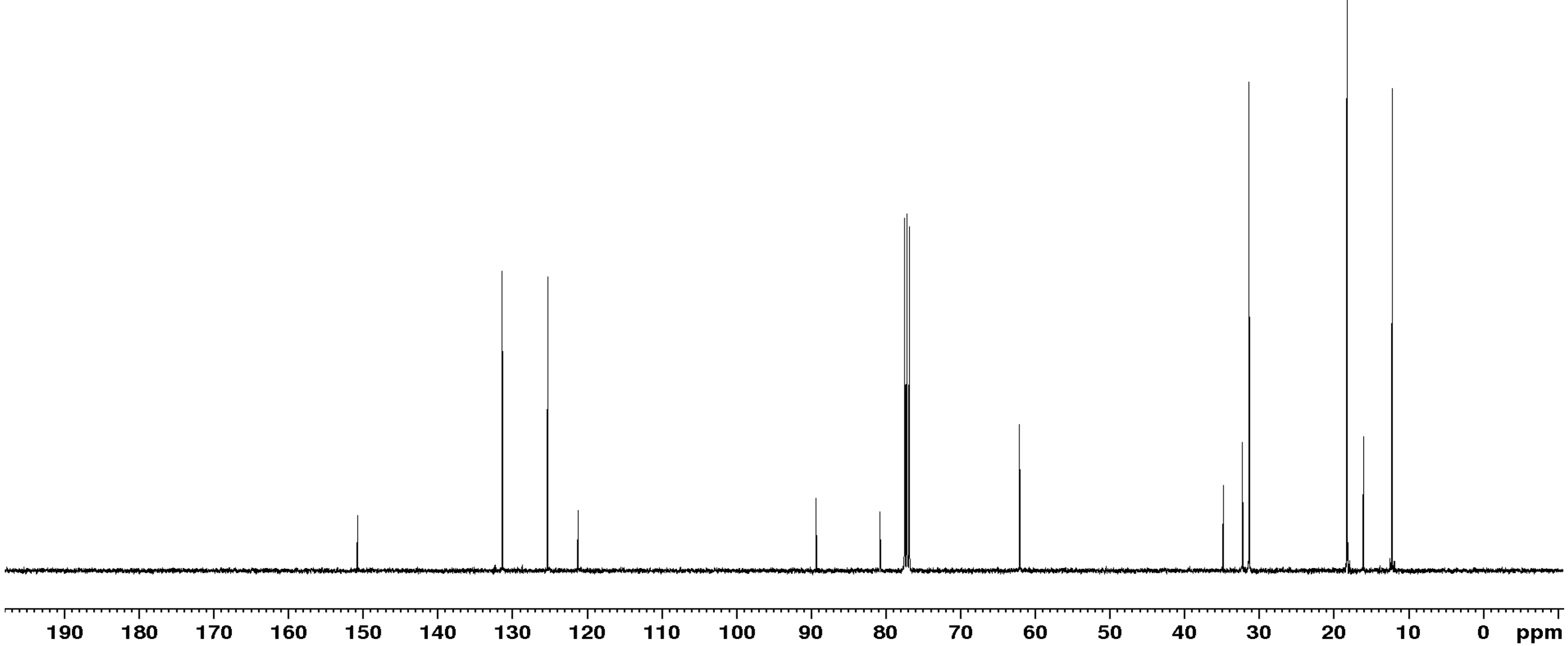

Figure S21. ^{29}Si DEPT NMR spectrum (79 MHz, $CDCl_3$, 298 K, optimized for *J* = 15.0 Hz) of ((5-(4-(*tert*-butyl)phenyl)pent-4-yn-1-yl)oxy)triisopropylsilane (**1e**)

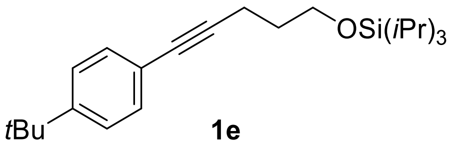

1e

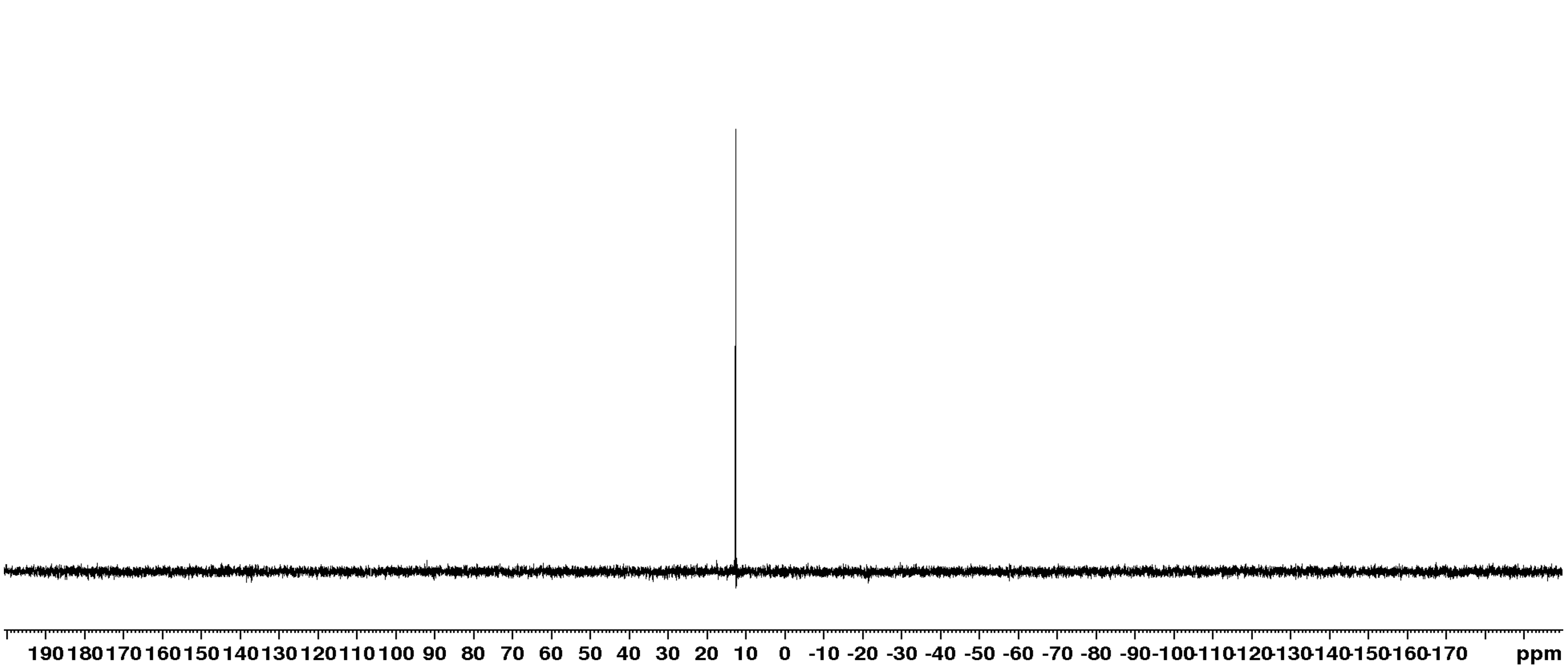

Figure S22. ^{1}H NMR spectrum (500 MHz, $CDCl_3$, 298 K) of ((5-(3,5-dimethylphenyl)pent-4-yn-1-yl)oxy)triisopropylsilane (**1f**)

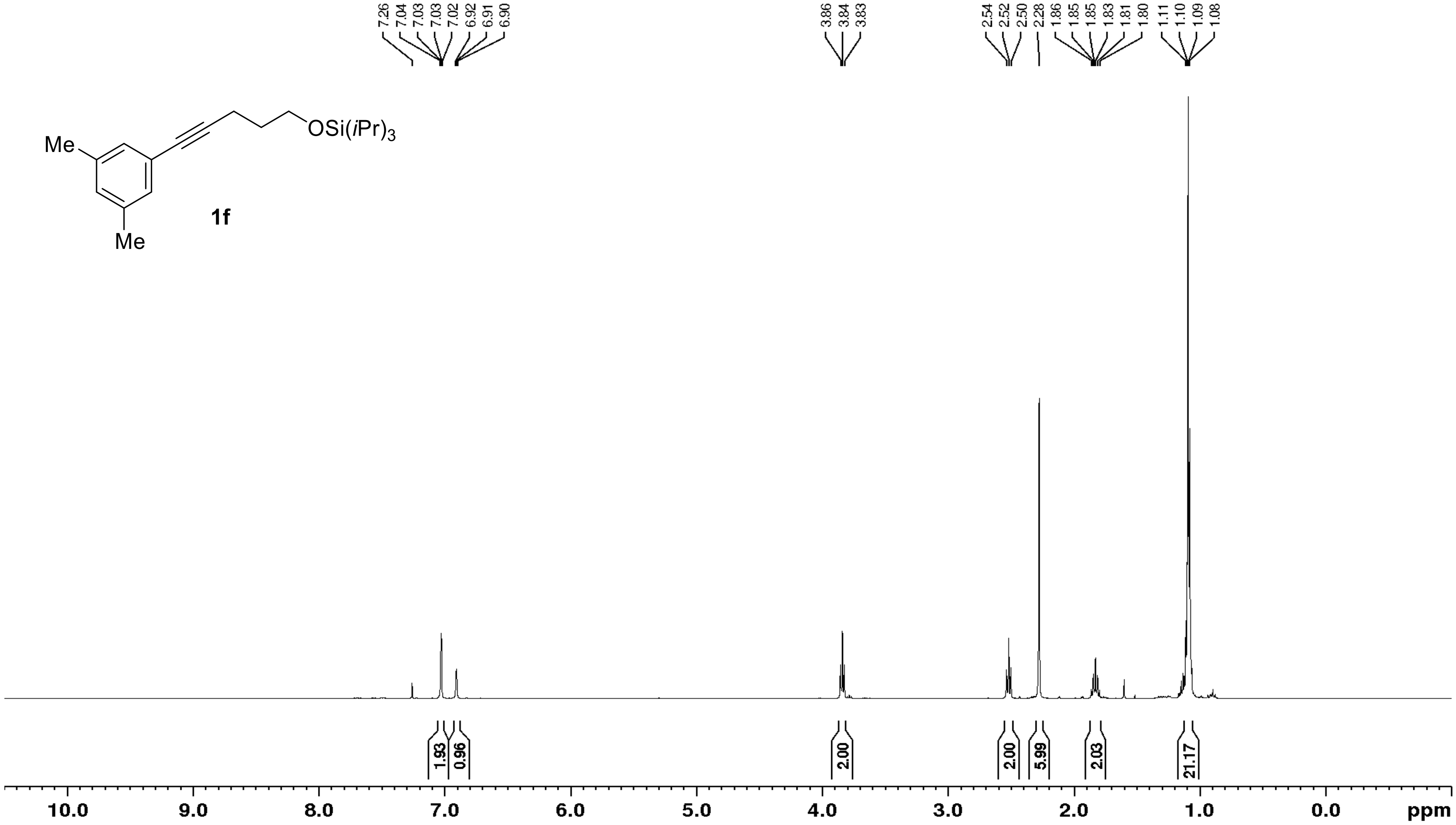

Figure S23. $^{13}C\{^{1}H\}$ NMR spectrum (126 MHz, $CDCl_3$, 298 K) of ((5-(3,5-dimethylphenyl)pent-4-yn-1-yl)oxy)triisopropylsilane (**1f**)

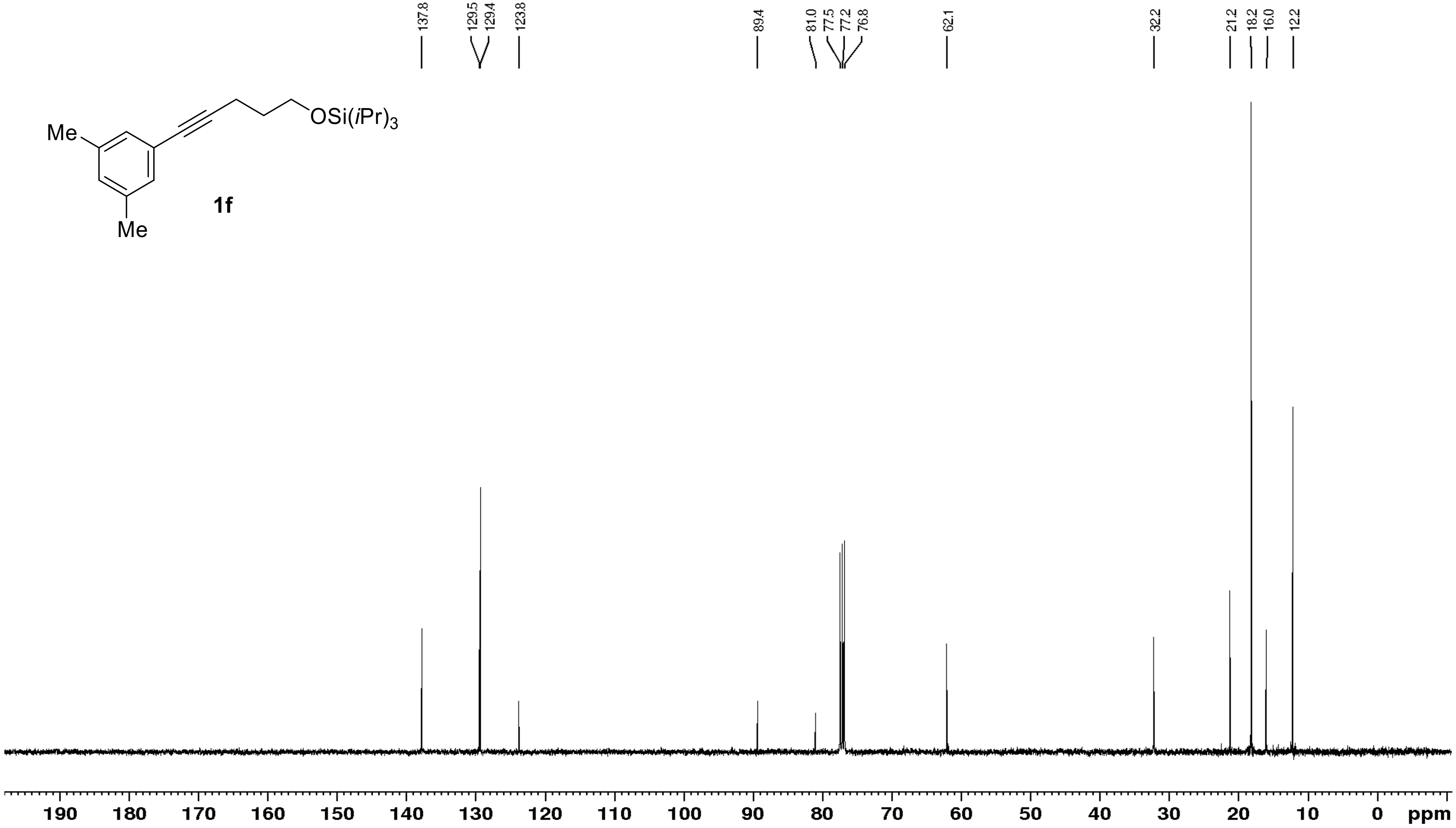

Figure S24. ^{29}Si DEPT NMR spectrum (99 MHz, $CDCl_3$, 298 K, optimized for J = 15.0 Hz) of ((5-(3,5-dimethylphenyl)pent-4-yn-1-yl)oxy)triisopropylsilane (**1f**)

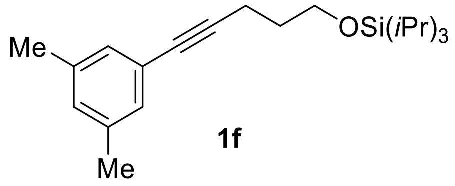

1f

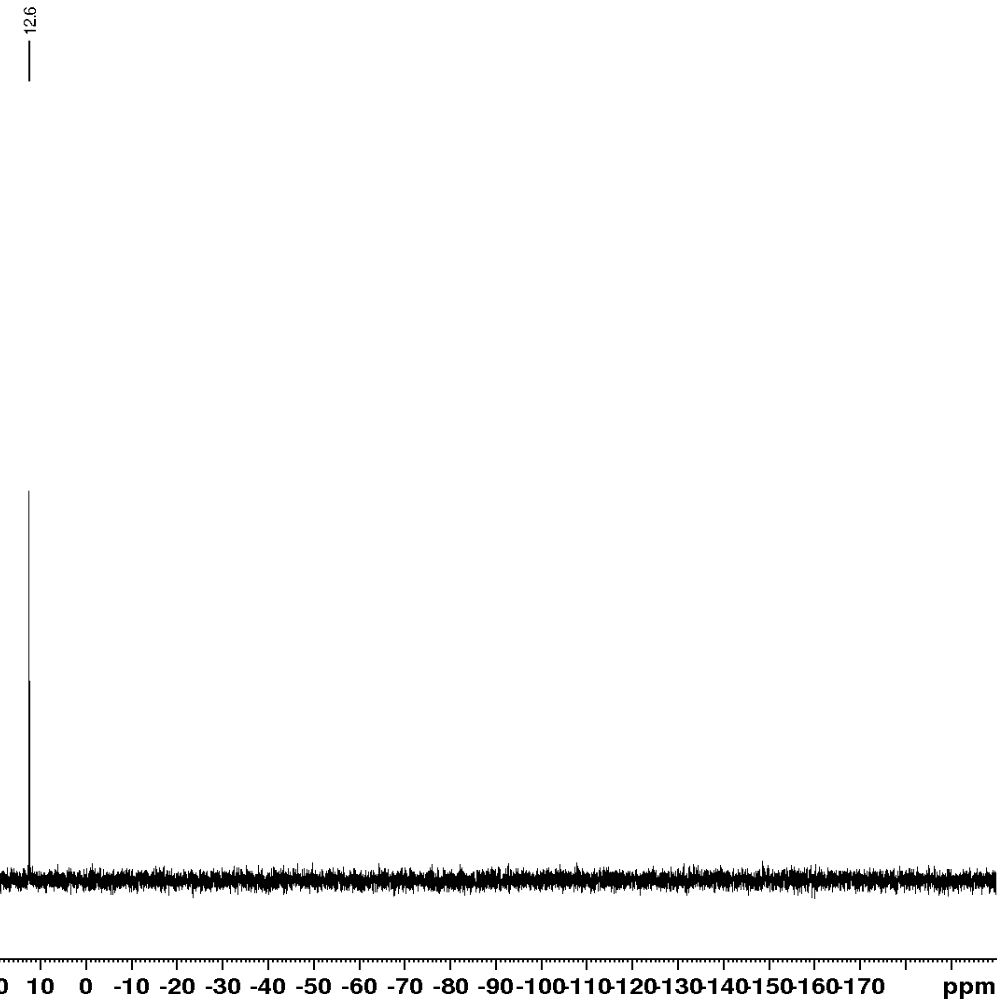

Figure S25. ^{1}H NMR spectrum (400 MHz, $CDCl_3$, 298 K) of ((5-([1,1'-biphenyl]-4-yl)pent-4-yn-1-yl)oxy)triisopropylsilane (**1g**)

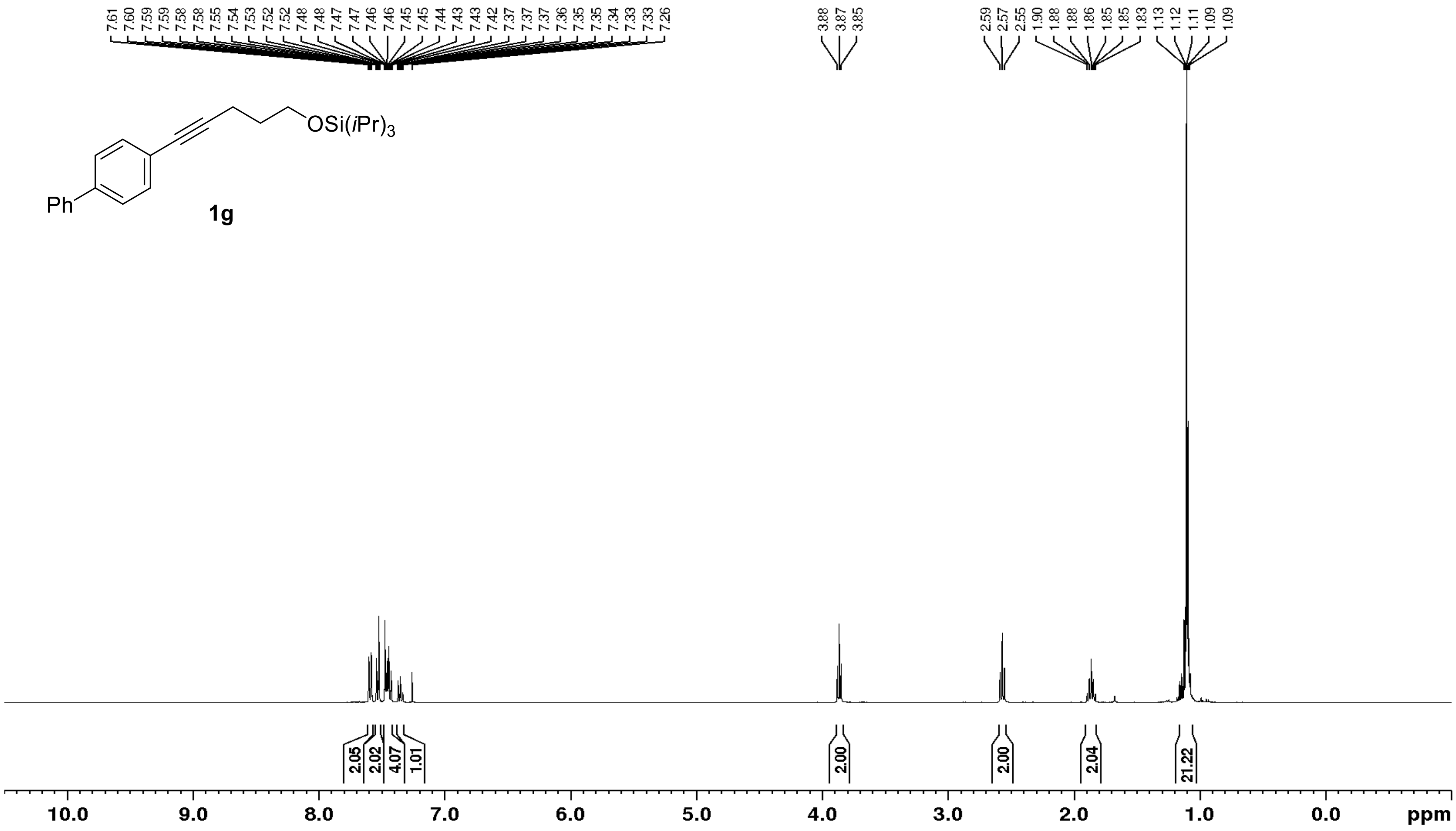

Figure S26. $^{13}C\{^1H\}$ NMR spectrum (101 MHz, $CDCl_3$, 298 K) of ((5-([1,1'-biphenyl]-4-yl)pent-4-yn-1-yl)oxy)triisopropylsilane (**1g**)

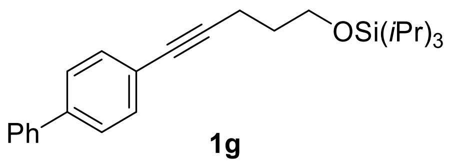

1g

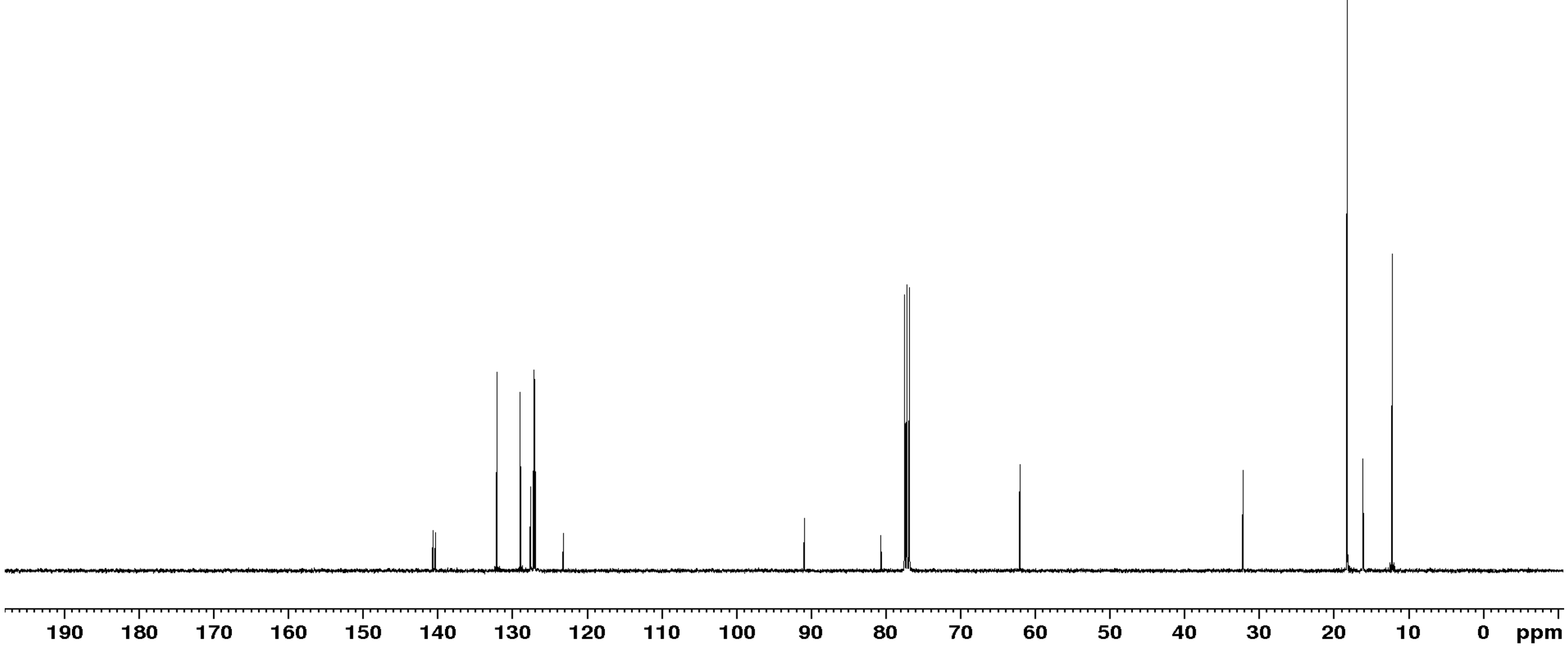

Figure S27. ^{29}Si DEPT NMR spectrum (79 MHz, $CDCl_3$, 298 K, optimized for J = 15.0 Hz) of ((5-([1,1'-biphenyl]-4-yl)pent-4-yn-1-yl)oxy)triisopropylsilane (**1g**)

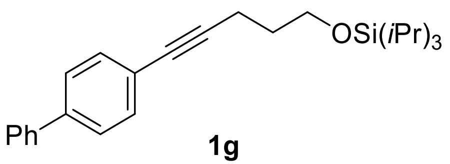

1g

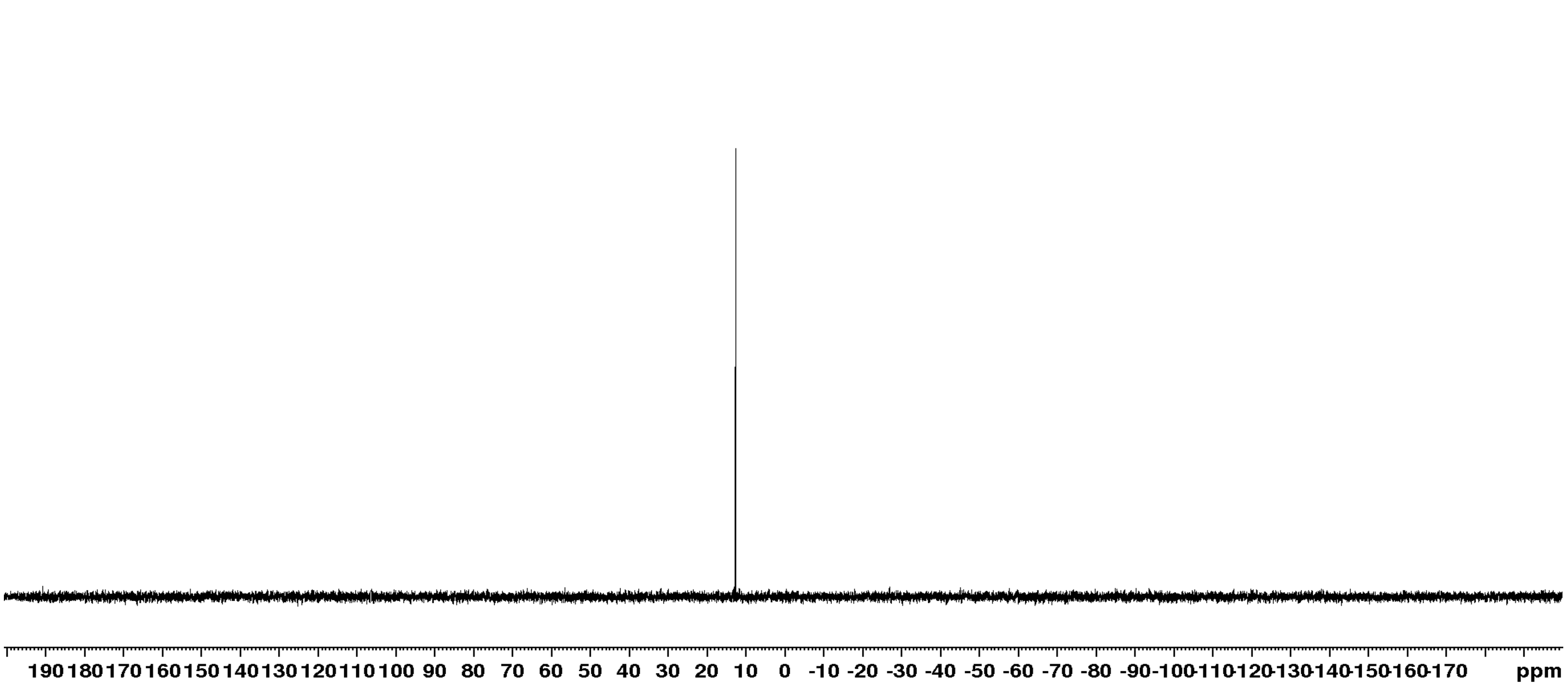

Figure 28. ^{1}H NMR spectrum (400 MHz, $CDCl_3$, 298 K) of triisopropyl((5-(4-(trimethylsilyl)phenyl)pent-4-yn-1-yl)oxy)silane (**1h**)

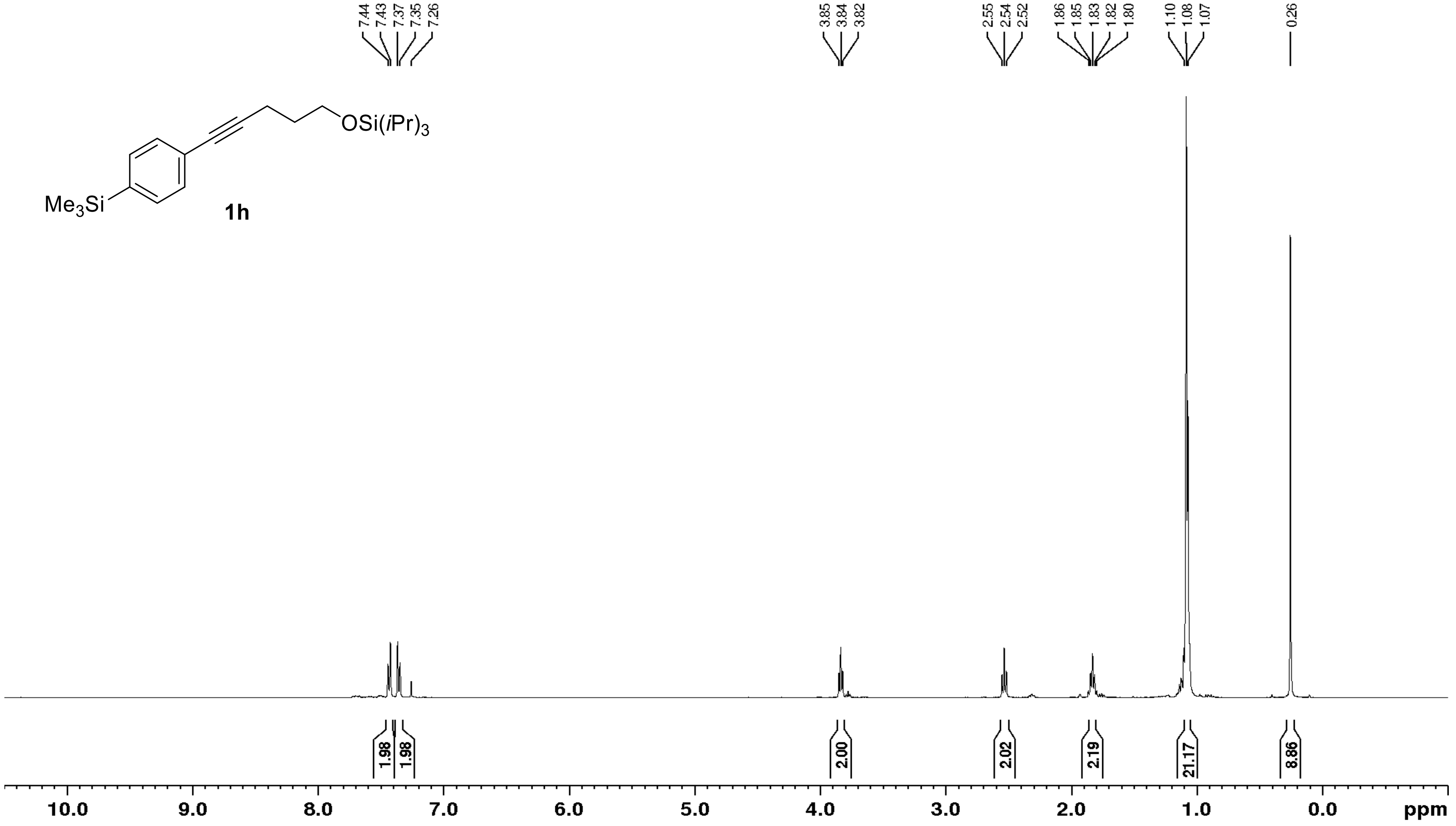

Figure S29. $^{13}C\{^{1}H\}$ NMR spectrum (101 MHz, $CDCl_3$, 298 K) of triisopropyl((5-(4-(trimethylsilyl)phenyl)pent-4-yn-1-yl)oxy)silane (**1h**)

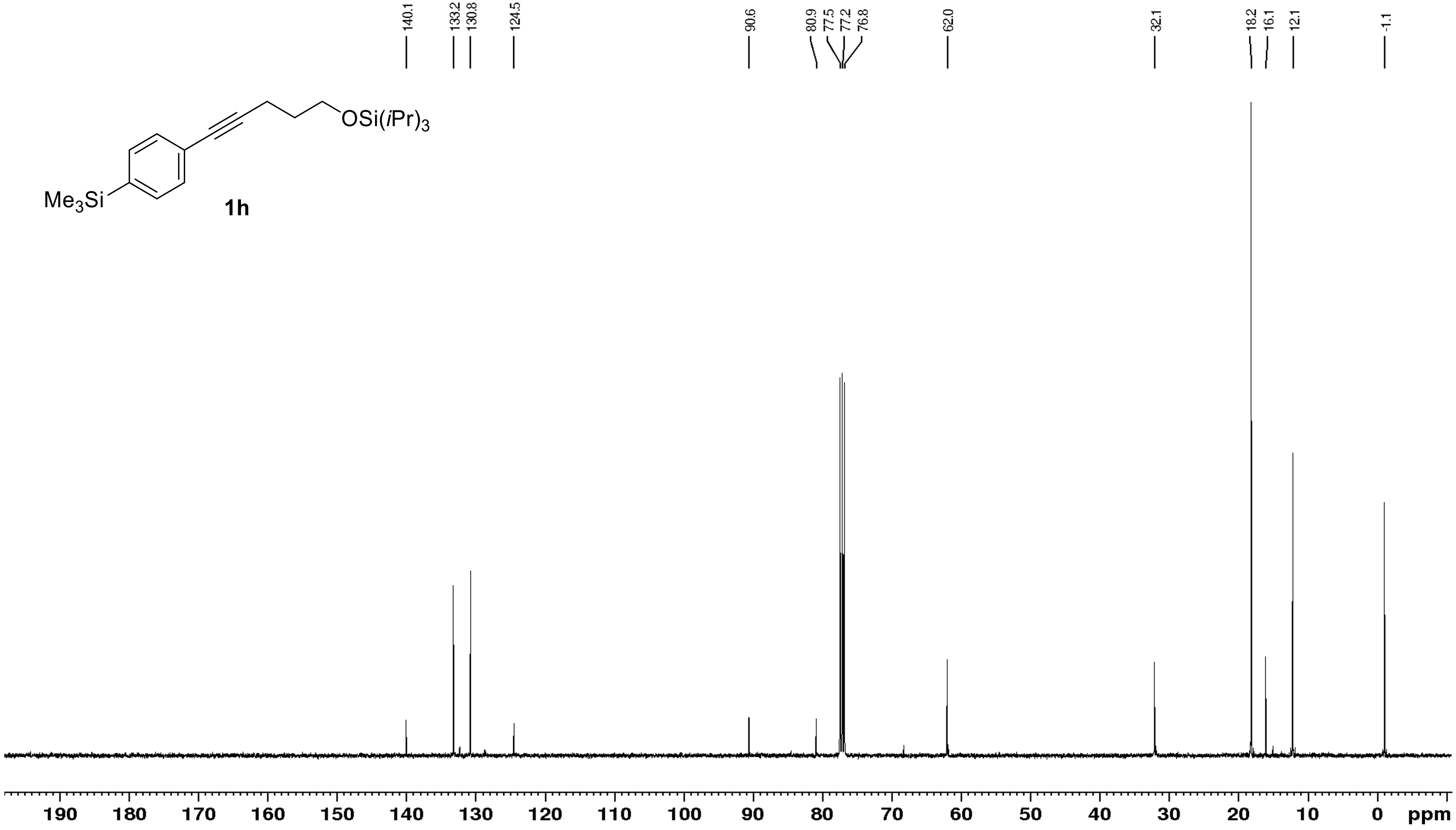

Figure S30. ^{29}Si DEPT NMR spectrum (79 MHz, $CDCl_3$, 298 K, optimized for J = 15.0 Hz) of triisopropyl((5-(4-(trimethylsilyl)phenyl)pent-4-yn-1-yl)oxy)silane (**1h**)

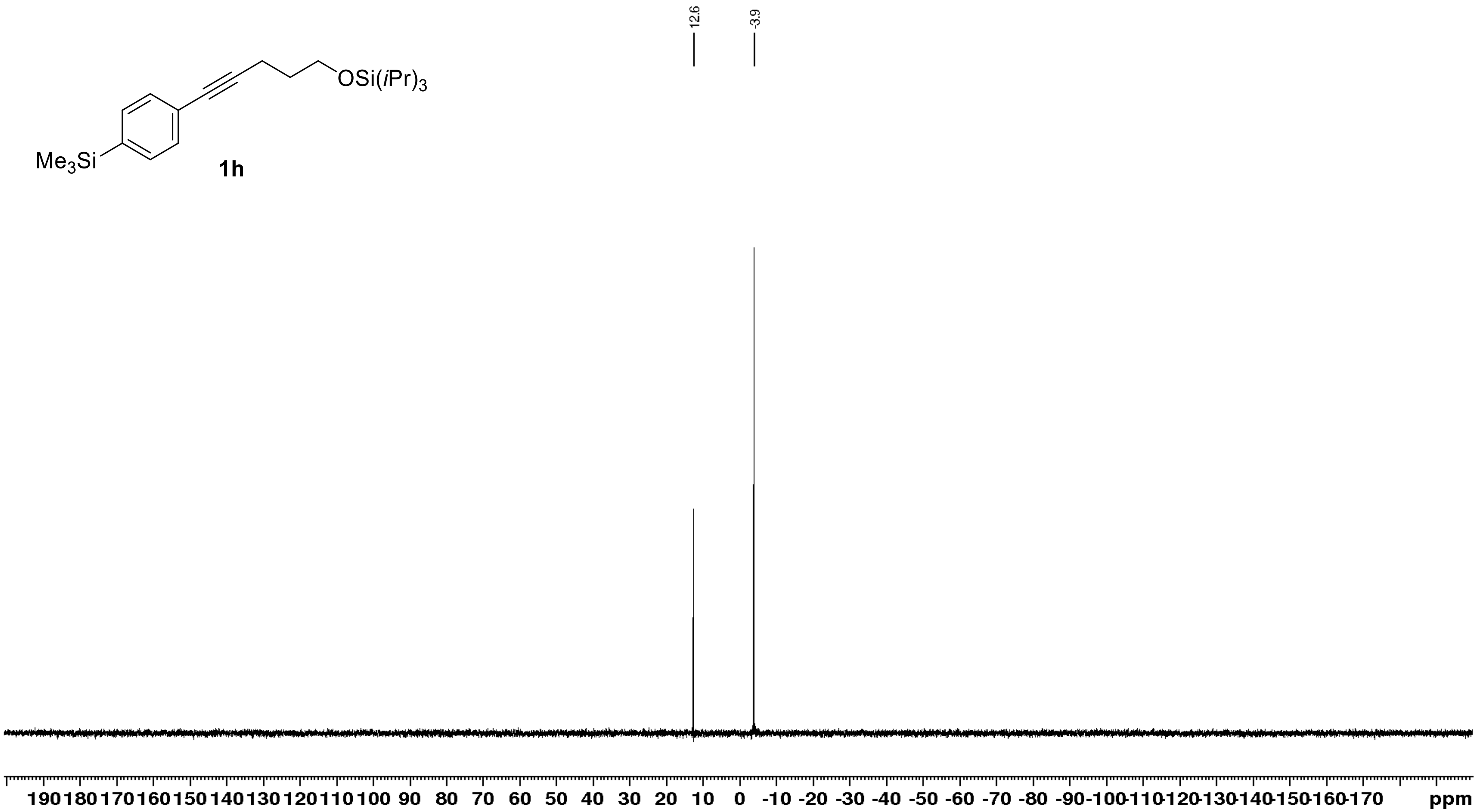

Figure S31. ^{1}H NMR spectrum (400 MHz, $CDCl_3$, 298 K) of *tert*-butyldimethyl(4-(5-((triisopropylsilyl)oxy)pent-1-yn-1-yl)phenoxy)silane (**1i**)

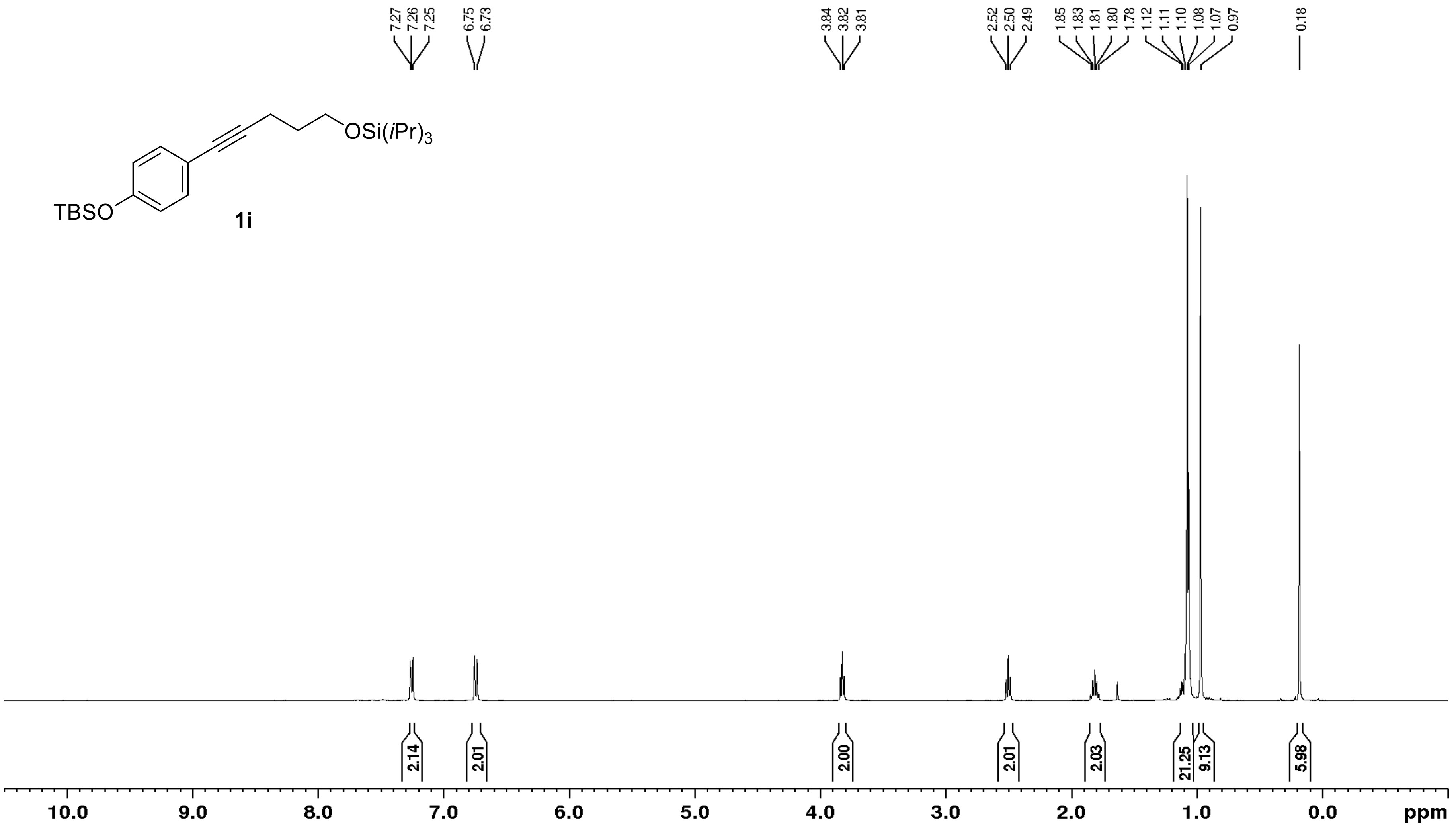

Figure S32. $^{13}C\{^1H\}$ NMR spectrum (101 MHz, $CDCl_3$, 298 K) of *tert*-butyldimethyl(4-(5-((triisopropylsilyl)oxy)pent-1-yn-1-yl)phenoxy)silane (**1i**)

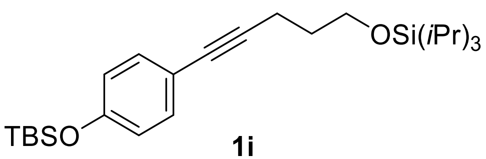

1i

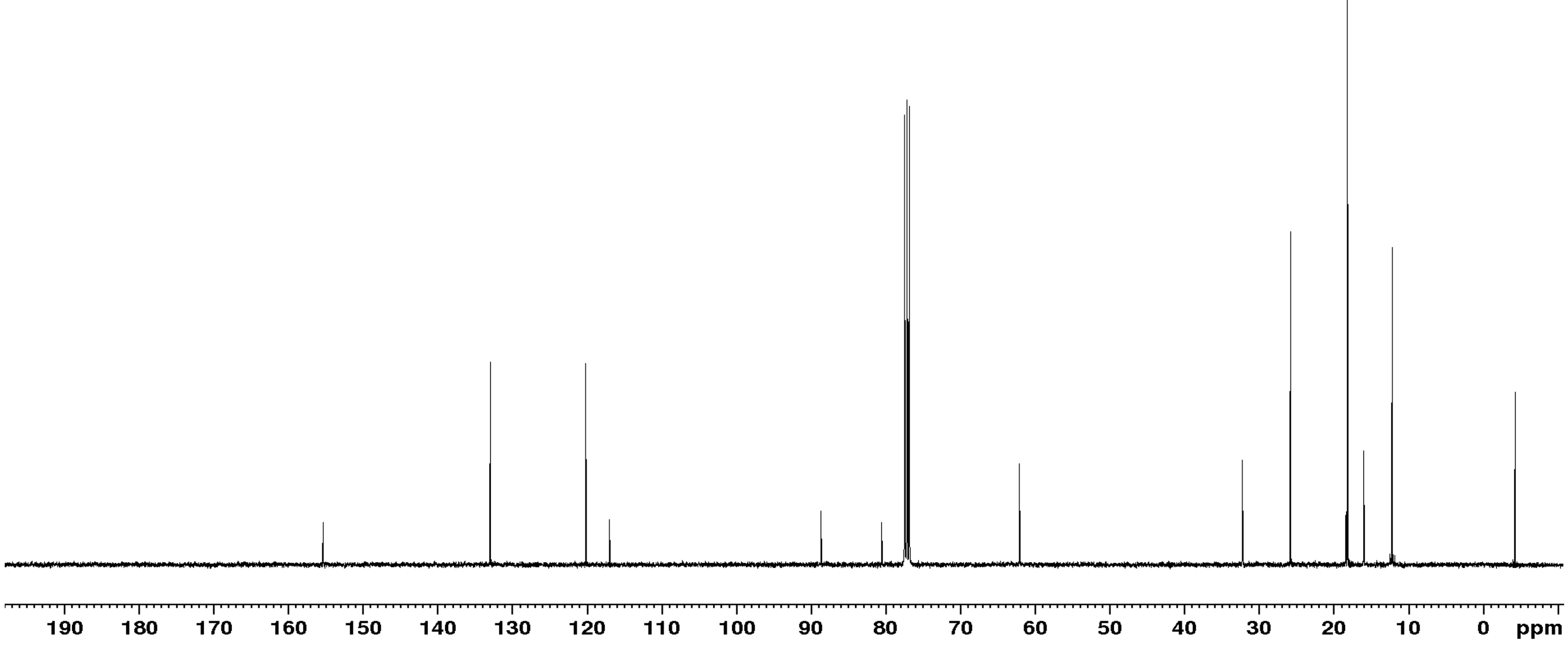

Figure S33. ^{29}Si DEPT NMR spectrum (79 MHz, $CDCl_3$, 298 K, optimized for J = 15.0 Hz) of *tert*-butyldimethyl(4-(5-((triisopropylsilyl)oxy)pent-1-yn-1-yl)phenoxy)silane (**1i**)

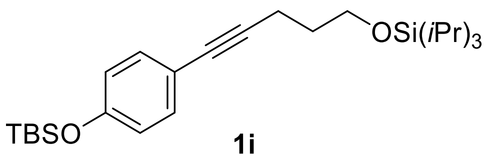

1i

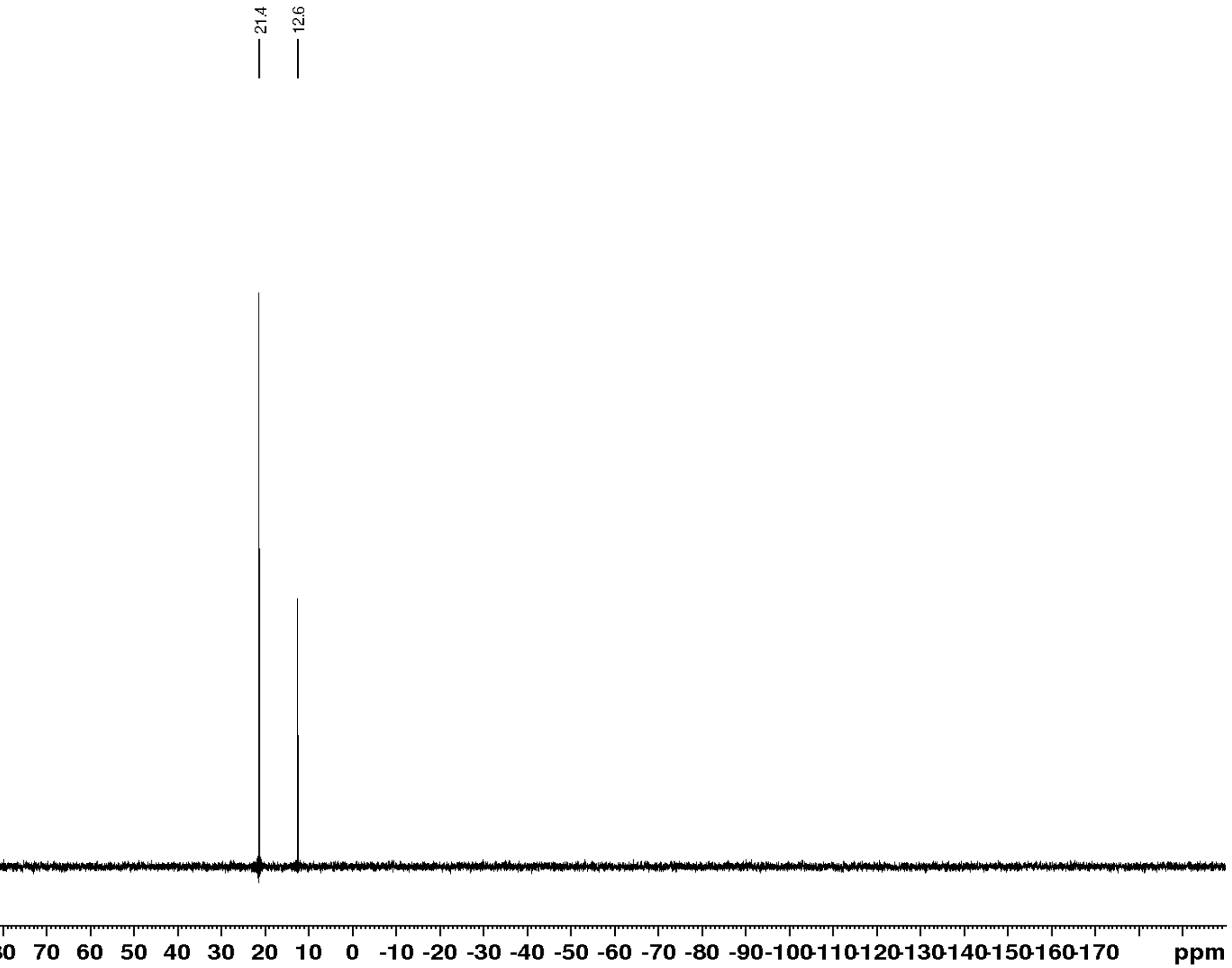

Figure S34. ^{1}H NMR spectrum (400 MHz, $CDCl_3$, 298 K) of ((5-(2-chlorophenyl)pent-4-yn-1-yl)oxy)triisopropylsilane (**1j**)

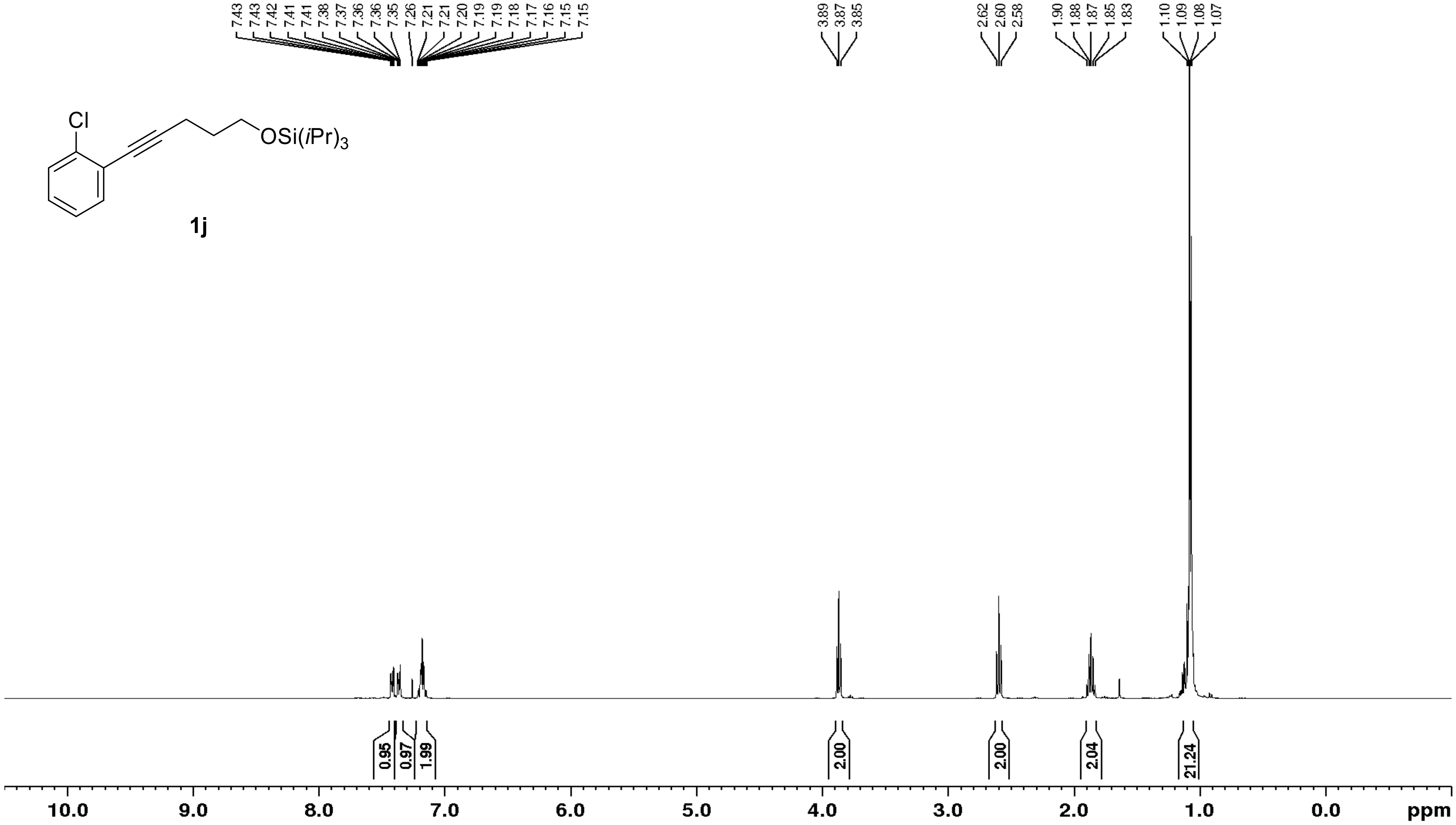

Figure S35. $^{13}C\{^1H\}$ NMR spectrum (101 MHz, $CDCl_3$, 298 K) of ((5-(2-chlorophenyl)pent-4-yn-1-yl)oxy)triisopropylsilane (**1j**)

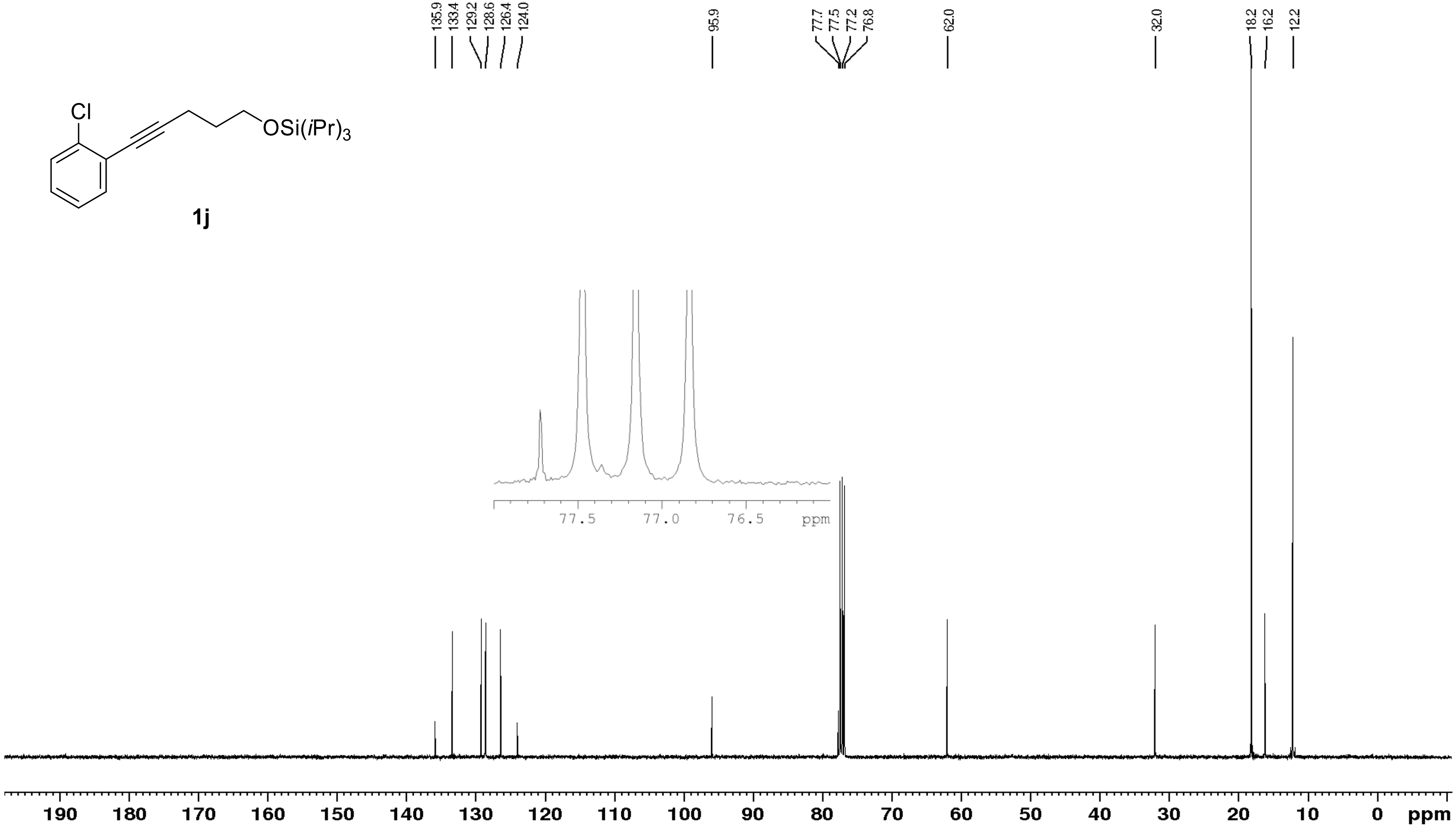

Figure S36. ^{29}Si DEPT NMR spectrum (79 MHz, $CDCl_3$, 298 K, optimized for *J* = 15.0 Hz) of ((5-(2-chlorophenyl)pent-4-yn-1-yl)oxy)triisopropylsilane (**1j**)

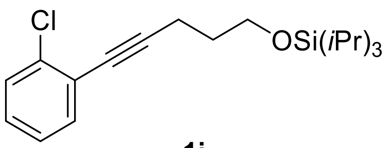

1j

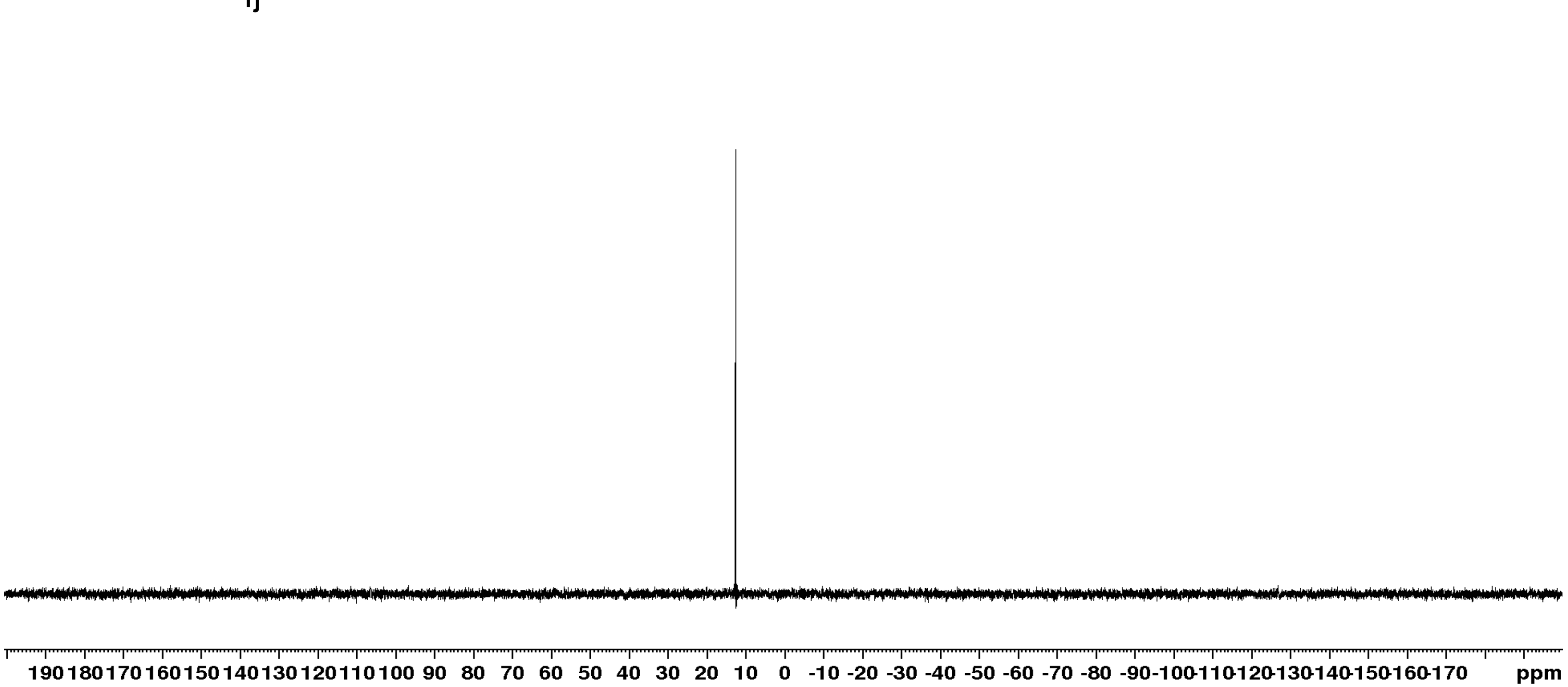

Figure S37. ^{1}H NMR spectrum (400 MHz, $CDCl_3$, 298 K) of ((5-(2-bromophenyl)pent-4-yn-1-yl)oxy)triisopropylsilane (**1k**)

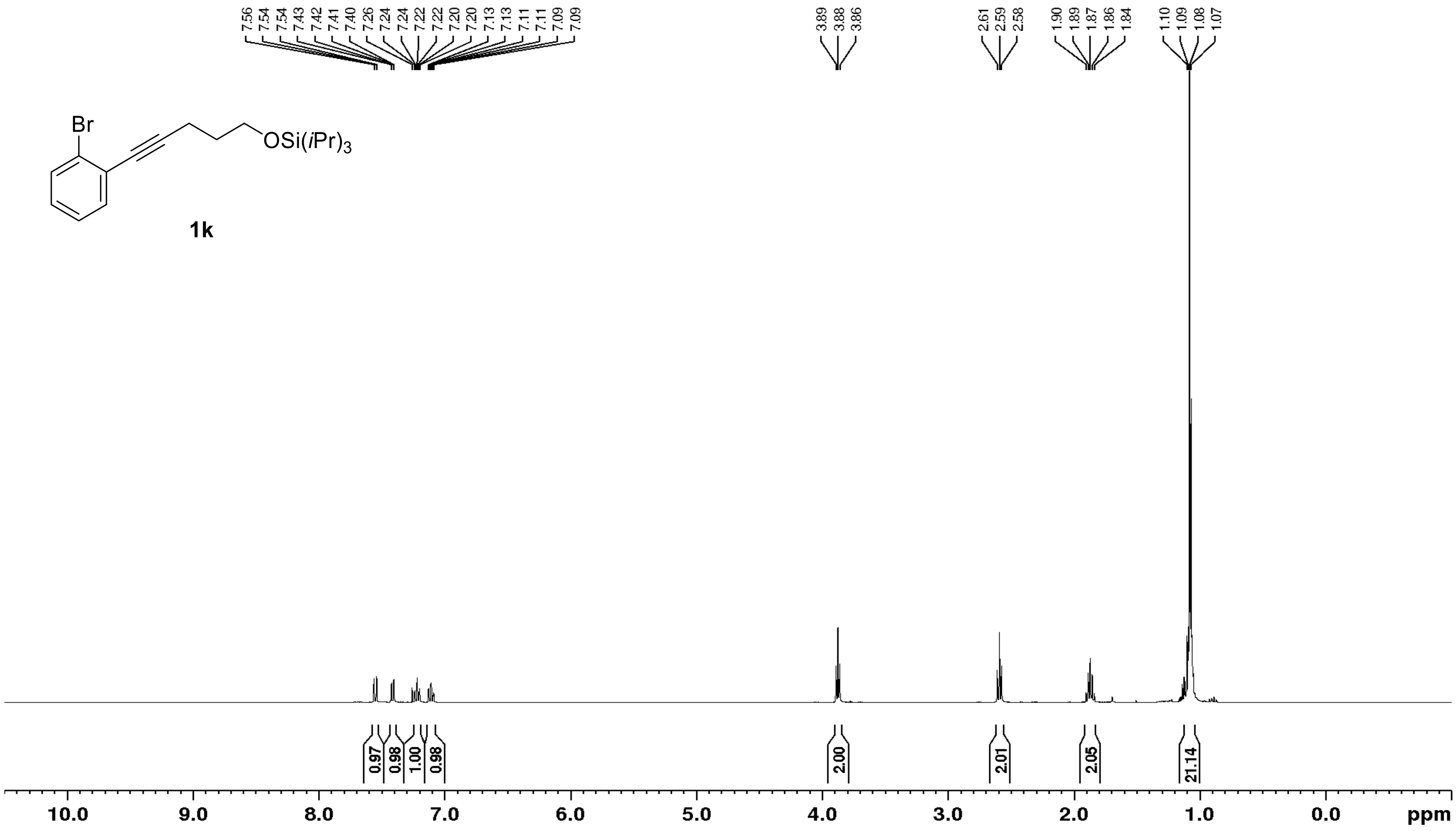

Figure S38. $^{13}C\{^{1}H\}$ NMR spectrum (101 MHz, $CDCl_3$, 298 K) of ((5-(2-bromophenyl)pent-4-yn-1-yl)oxy)triisopropylsilane (**1k**)

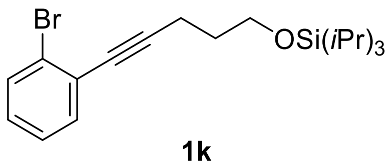

1k

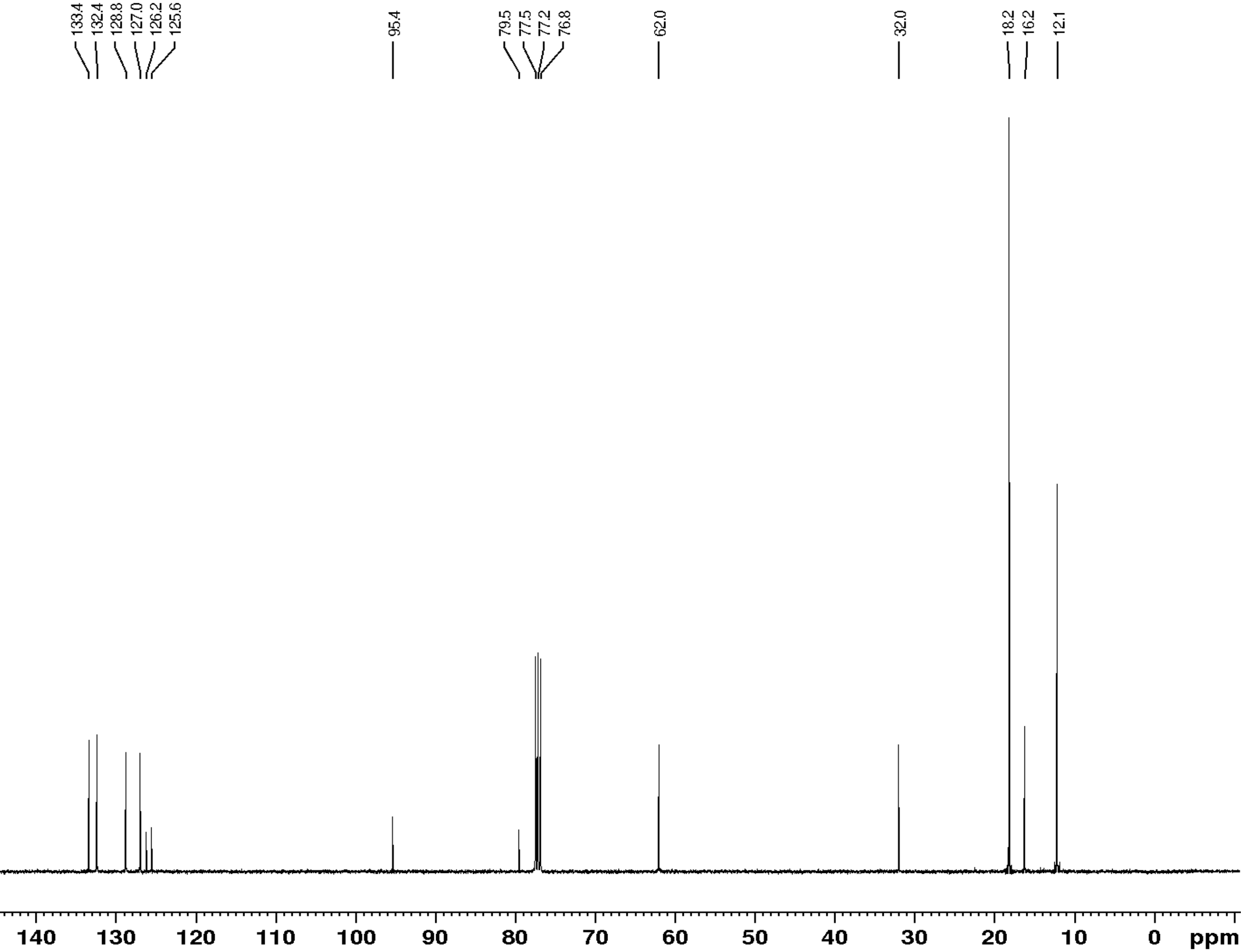

Figure S39. ^{29}Si DEPT NMR spectrum (79 MHz, $CDCl_3$, 298 K, optimized for *J* = 15.0 Hz) of ((5-(2-bromophenyl)pent-4-yn-1-yl)oxy)triisopropylsilane (**1k**)

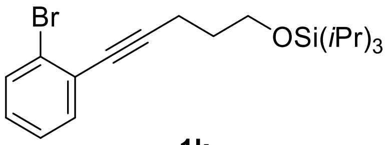

1k

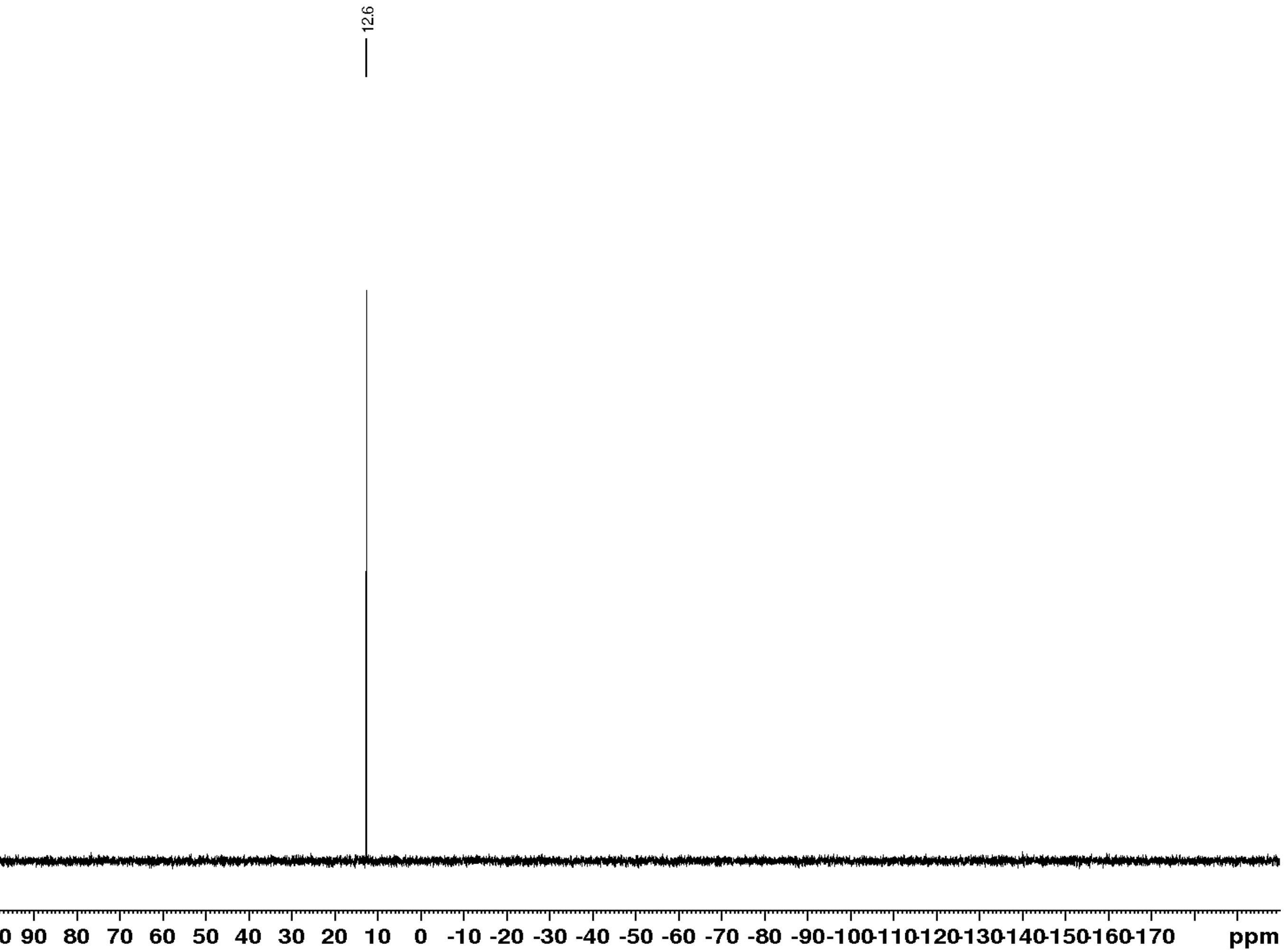

Figure S40. ^{1}H NMR spectrum (400 MHz, $CDCl_3$, 298 K) of ((5-(3-fluorophenyl)pent-4-yn-1-yl)oxy)triisopropylsilane (**1l**)

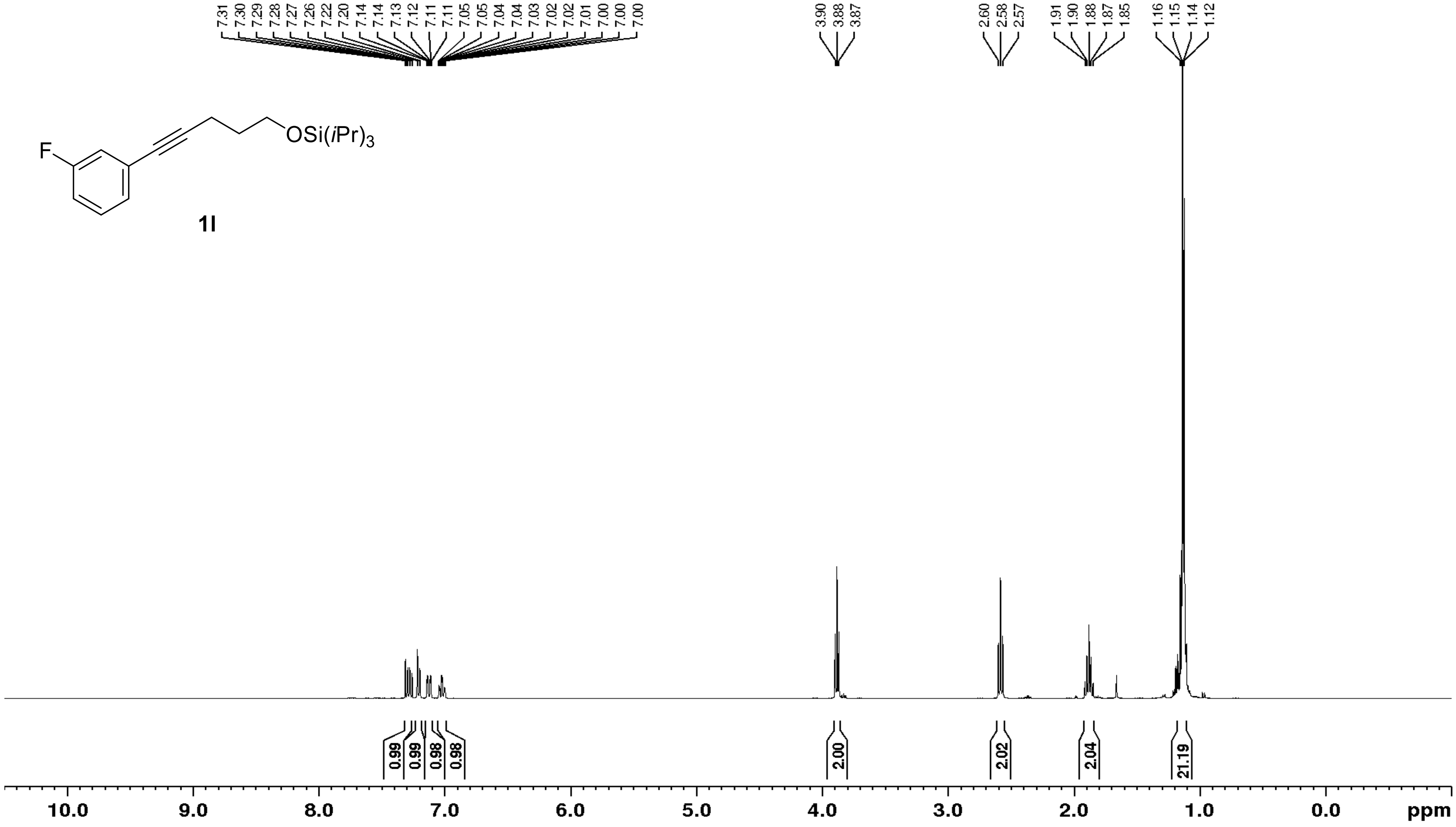

Figure S41. $^{13}C\{^1H\}$ NMR spectrum (101 MHz, $CDCl_3$, 298 K) of ((5-(3-fluorophenyl)pent-4-yn-1-yl)oxy)triisopropylsilane (**1l**)

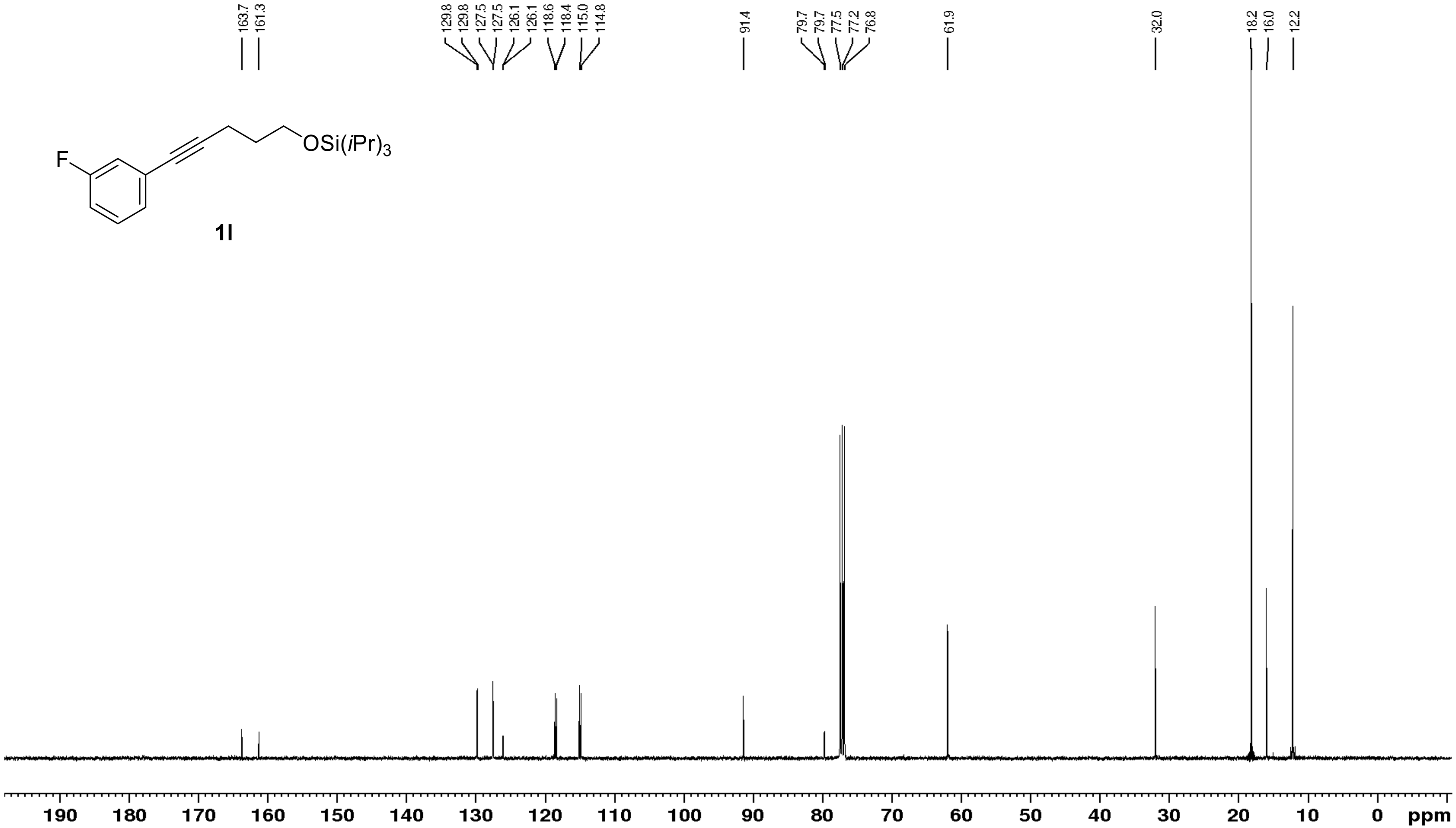

Figure S42. ^{19}F NMR spectrum (471 MHz, $CDCl_3$, 298 K) of ((5-(3-fluorophenyl)pent-4-yn-1-yl)oxy)triisopropylsilane (**1l**)

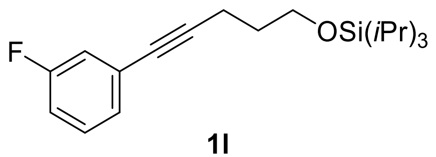

1l

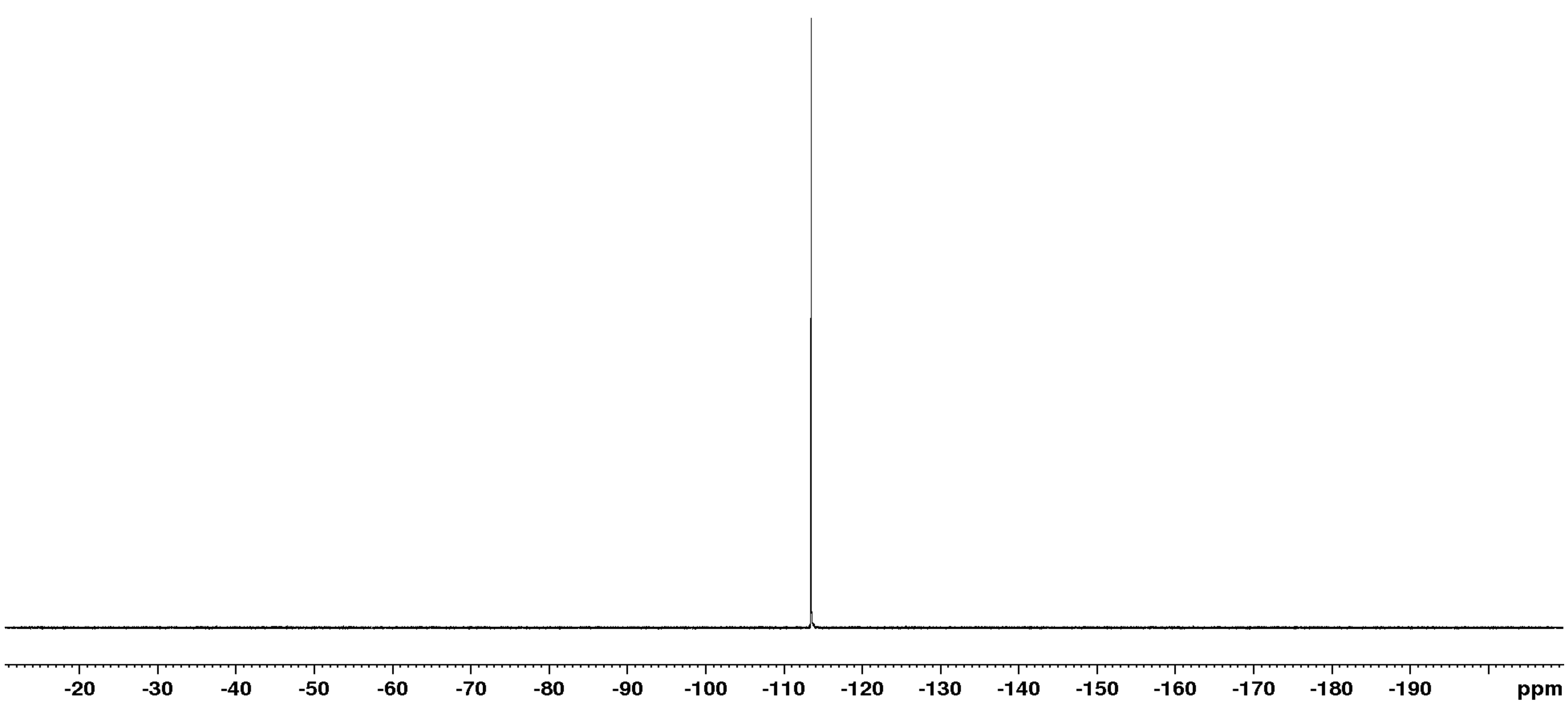

Figure S43. ^{29}Si DEPT NMR spectrum (79 MHz, $CDCl_3$, 298 K, optimized for J = 15.0 Hz) of ((5-(3-fluorophenyl)pent-4-yn-1-yl)oxy)triisopropylsilane (**1l**)

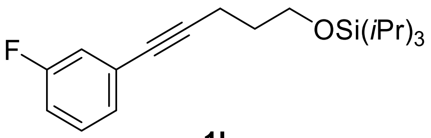

1l

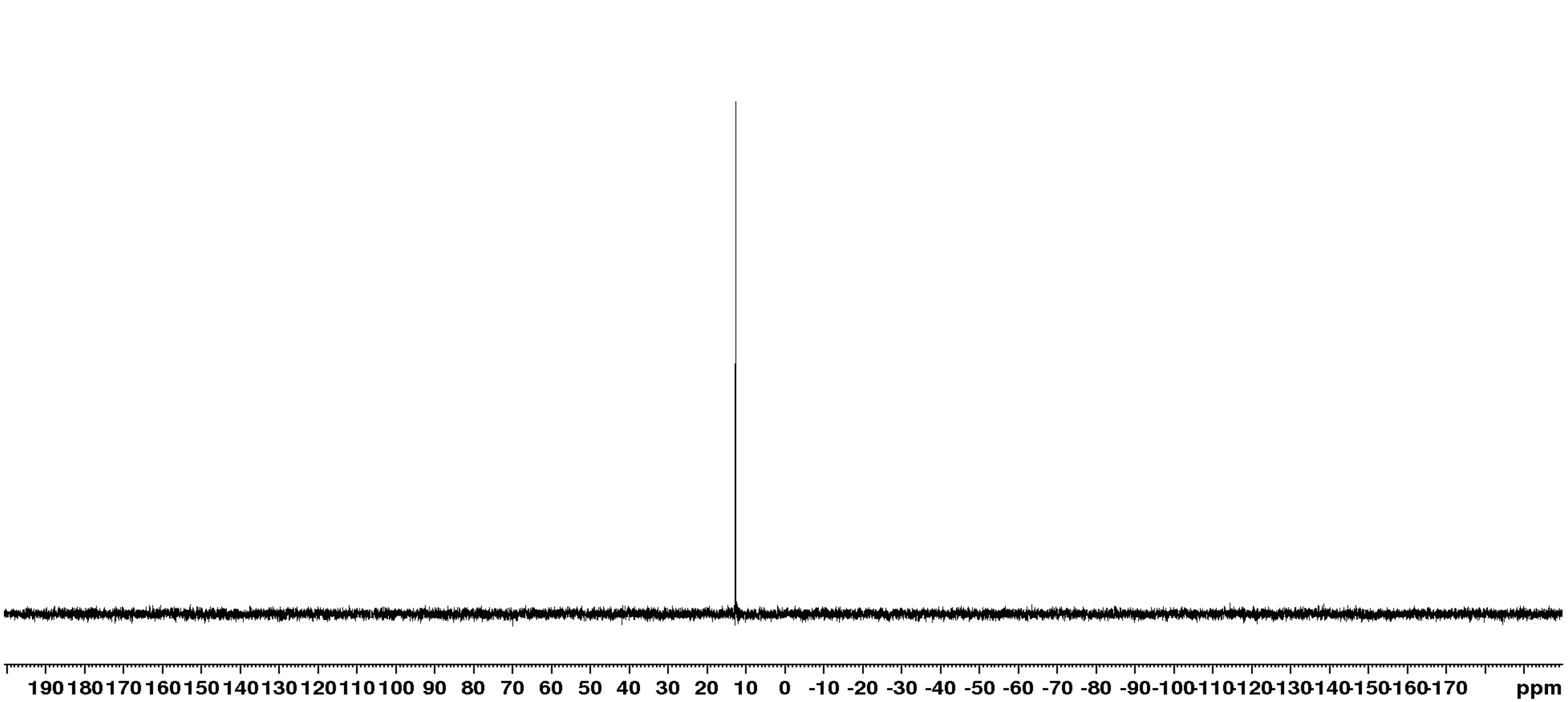

Figure S44. ^{1}H NMR spectrum (400 MHz, $CDCl_3$, 298 K) of ((5-(3-chlorophenyl)pent-4-yn-1-yl)oxy)triisopropylsilane (**1m**)

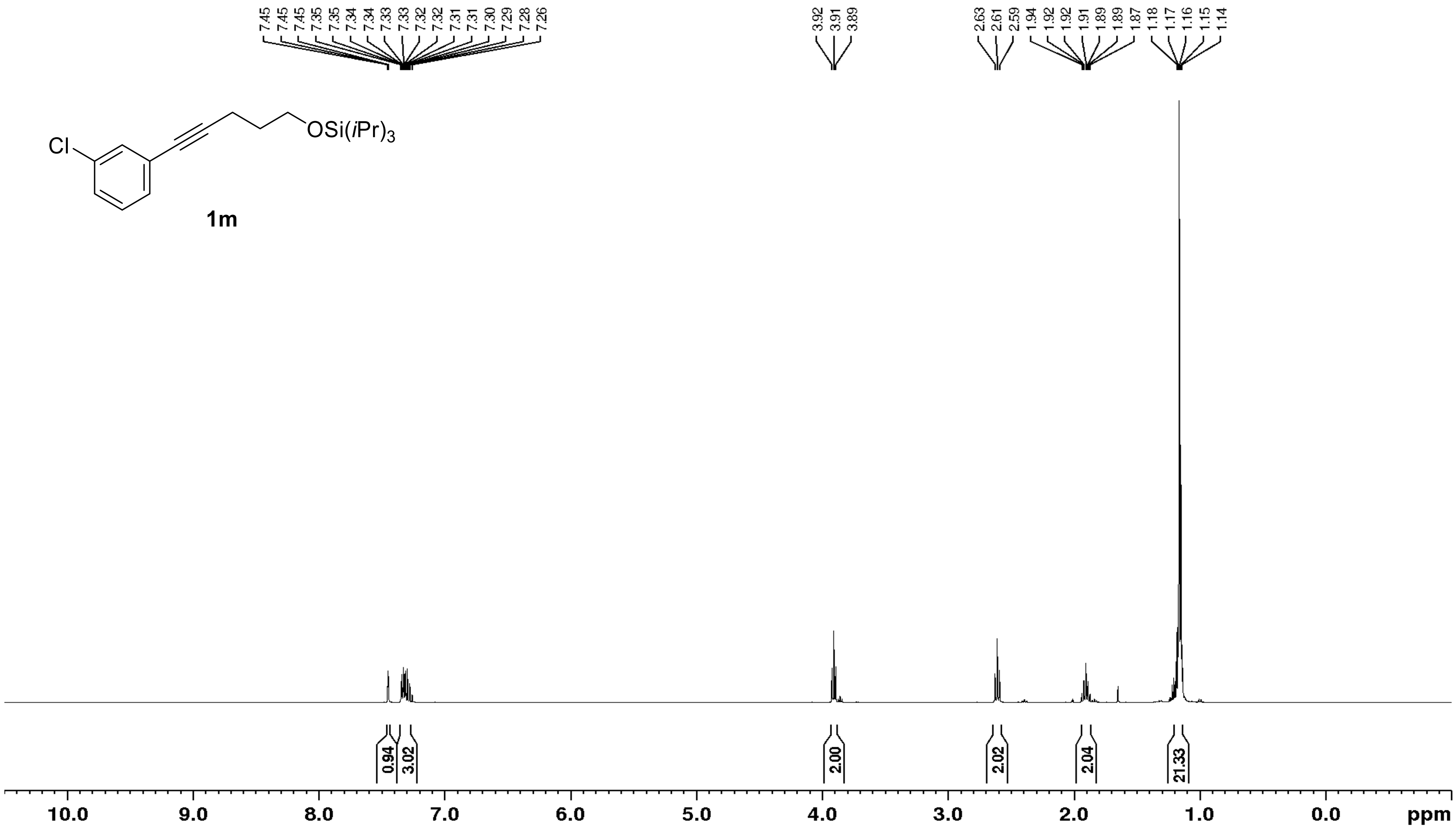

Figure S45. $^{13}C\{^1H\}$ NMR spectrum (101 MHz, $CDCl_3$, 298 K) of ((5-(3-chlorophenyl)pent-4-yn-1-yl)oxy)triisopropylsilane (**1m**)

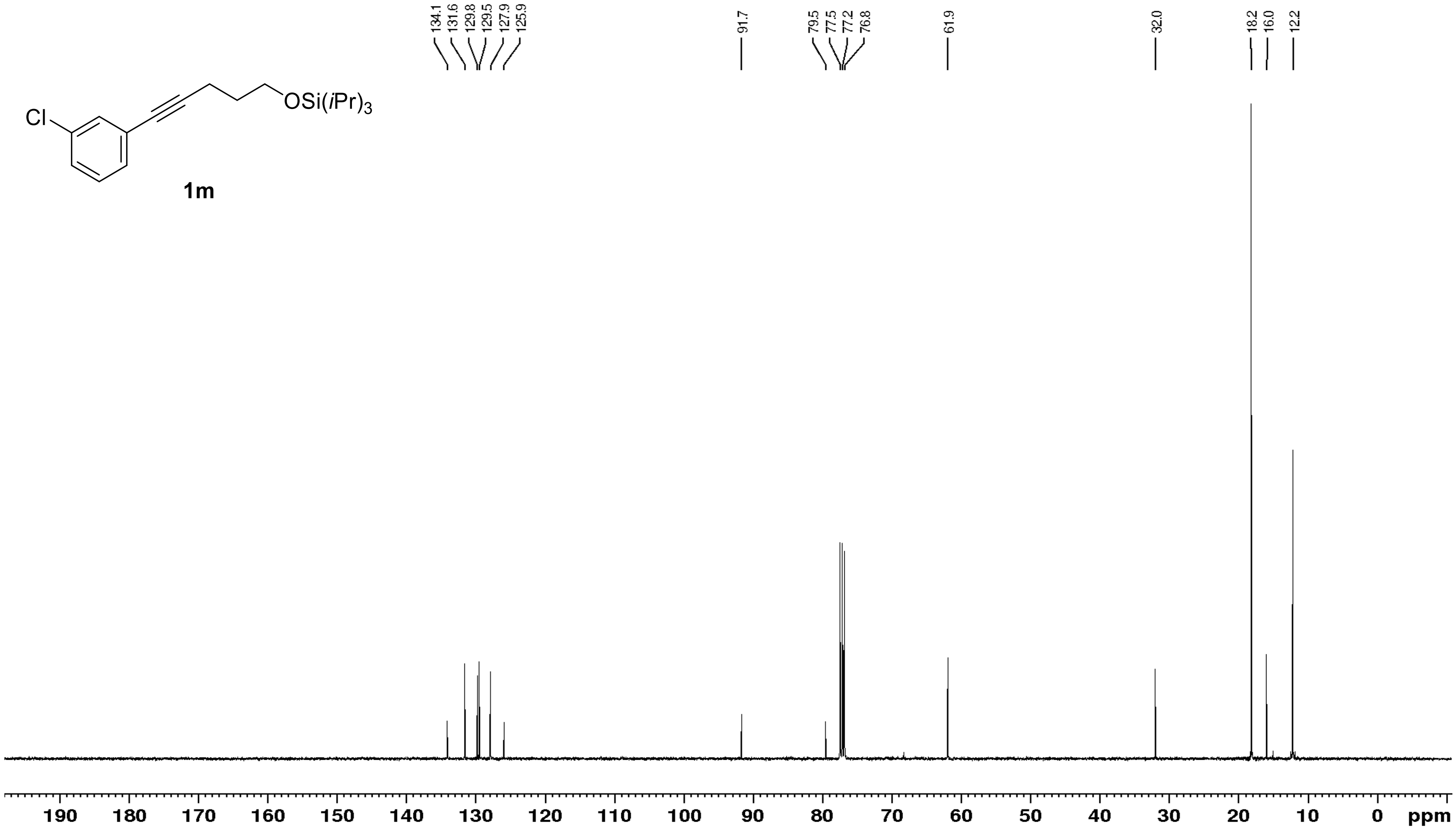

Figure S46. ^{29}Si DEPT NMR spectrum (79 MHz, $CDCl_3$, 298 K, optimized for *J* = 15.0 Hz) of ((5-(3-chlorophenyl)pent-4-yn-1-yl)oxy)triisopropylsilane (**1m**)

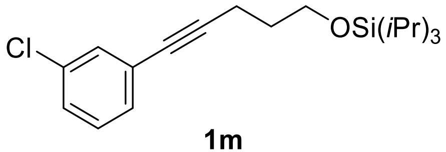

1m

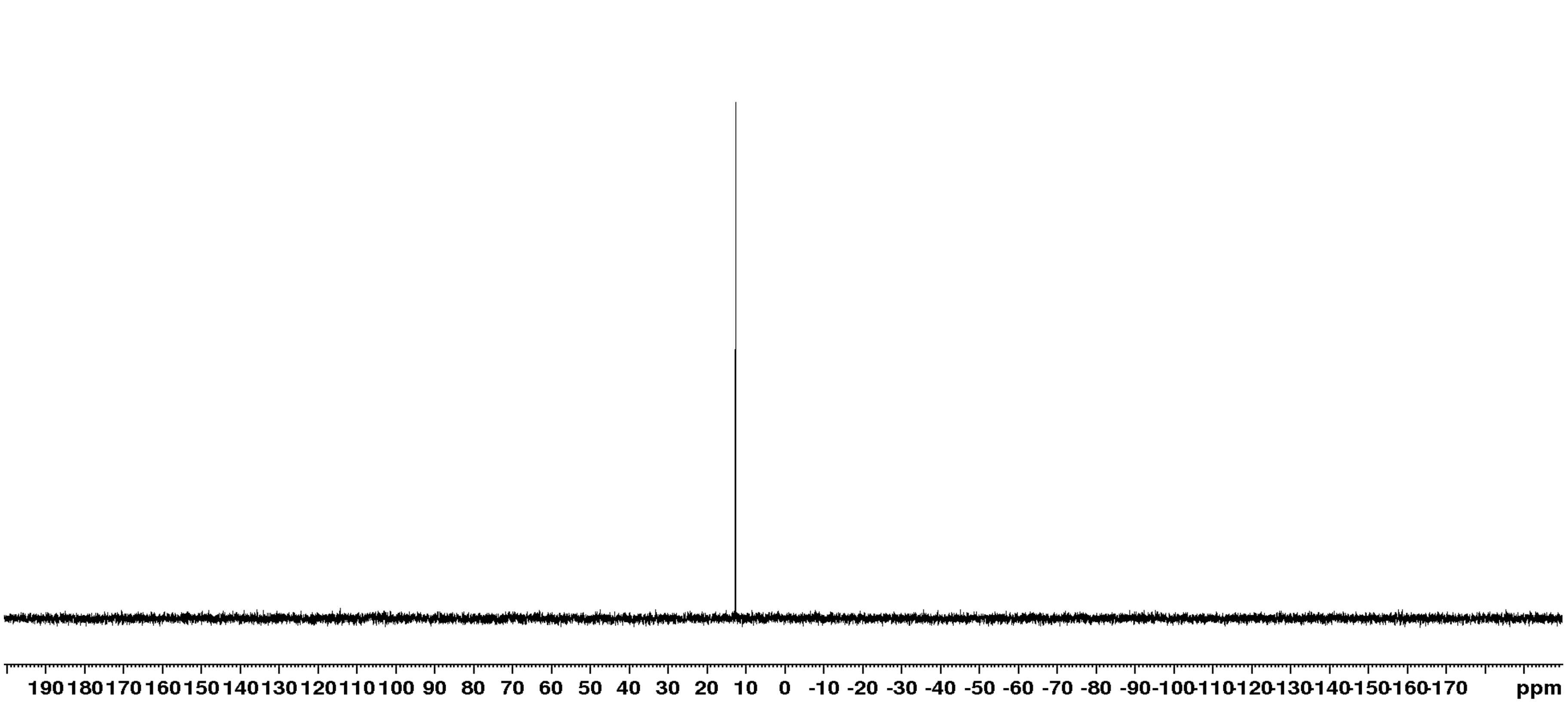

Figure S47. ^{1}H NMR spectrum (400 MHz, $CDCl_3$, 298 K) of ((5-(4-fluorophenyl)pent-4-yn-1-yl)oxy)triisopropylsilane (**1n**)

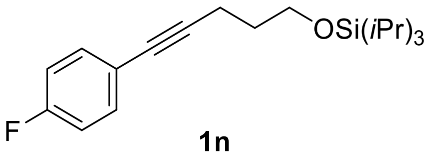

1n

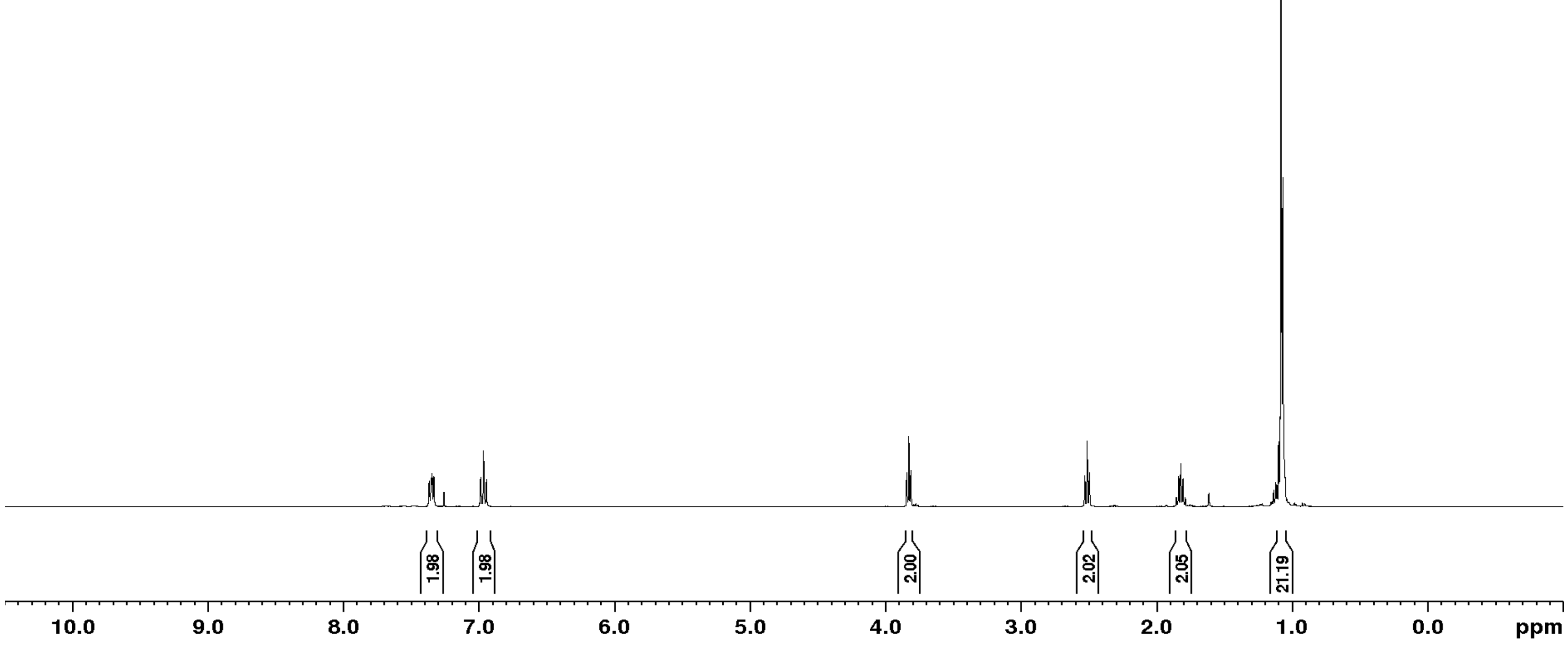

Figure S48. $^{13}C\{^1H\}$ NMR spectrum (101 MHz, $CDCl_3$, 298 K) of ((5-(4-fluorophenyl)pent-4-yn-1-yl)oxy)triisopropylsilane (**1n**)

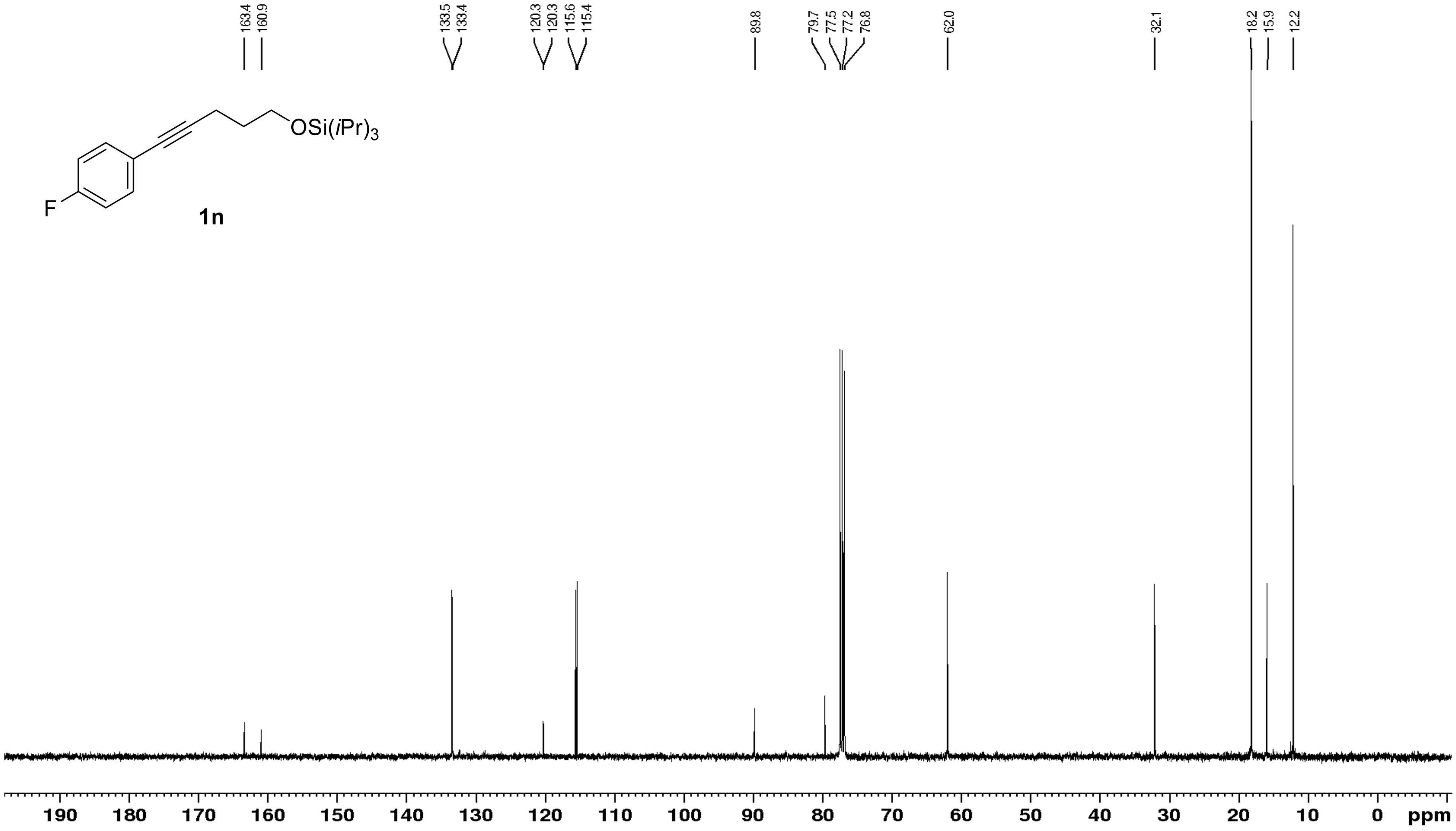

Figure S49. ^{19}F NMR spectrum (471 MHz, $CDCl_3$, 298 K) of ((5-(4-fluorophenyl)pent-4-yn-1-yl)oxy)triisopropylsilane (**1n**)

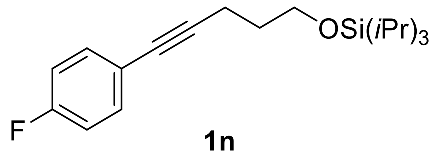

1n

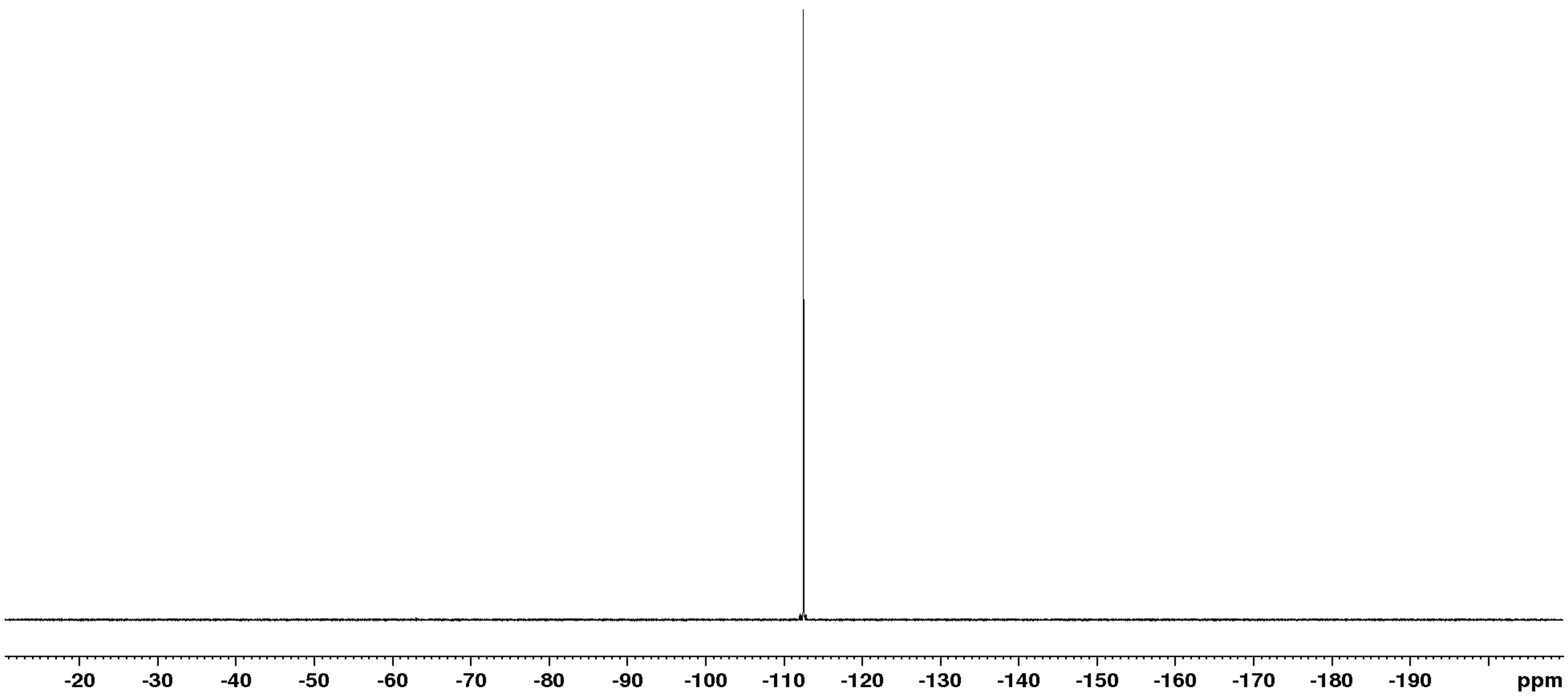

Figure S50. ^{29}Si DEPT NMR spectrum (79 MHz, $CDCl_3$, 298 K, optimized for *J* = 15.0 Hz) of ((5-(4-fluorophenyl)pent-4-yn-1-yl)oxy)triisopropylsilane (**1n**)

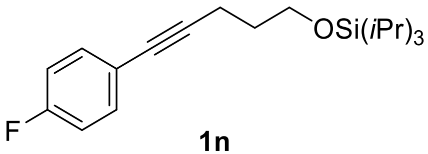

1n

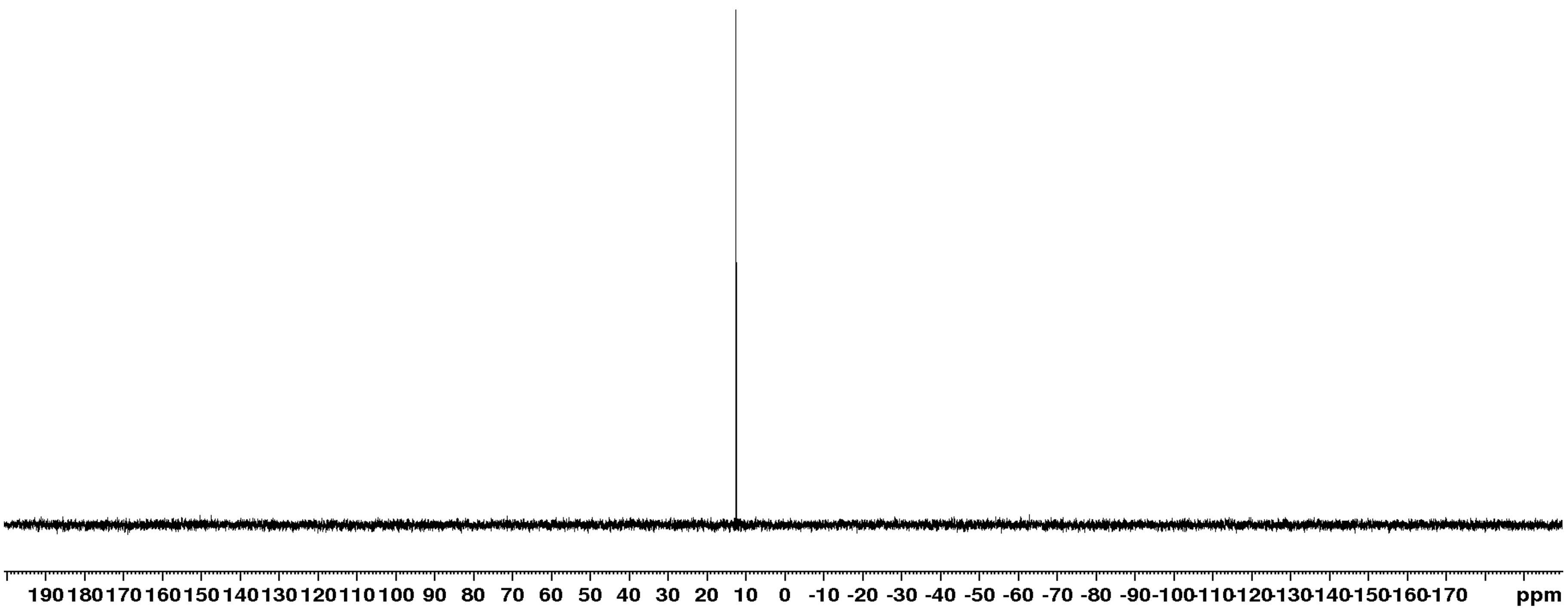

Figure S51. ^{1}H NMR spectrum (400 MHz, $CDCl_3$, 298 K) of ((5-(4-chlorophenyl)pent-4-yn-1-yl)oxy)triisopropylsilane (**1o**)

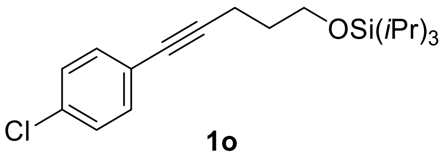

1o

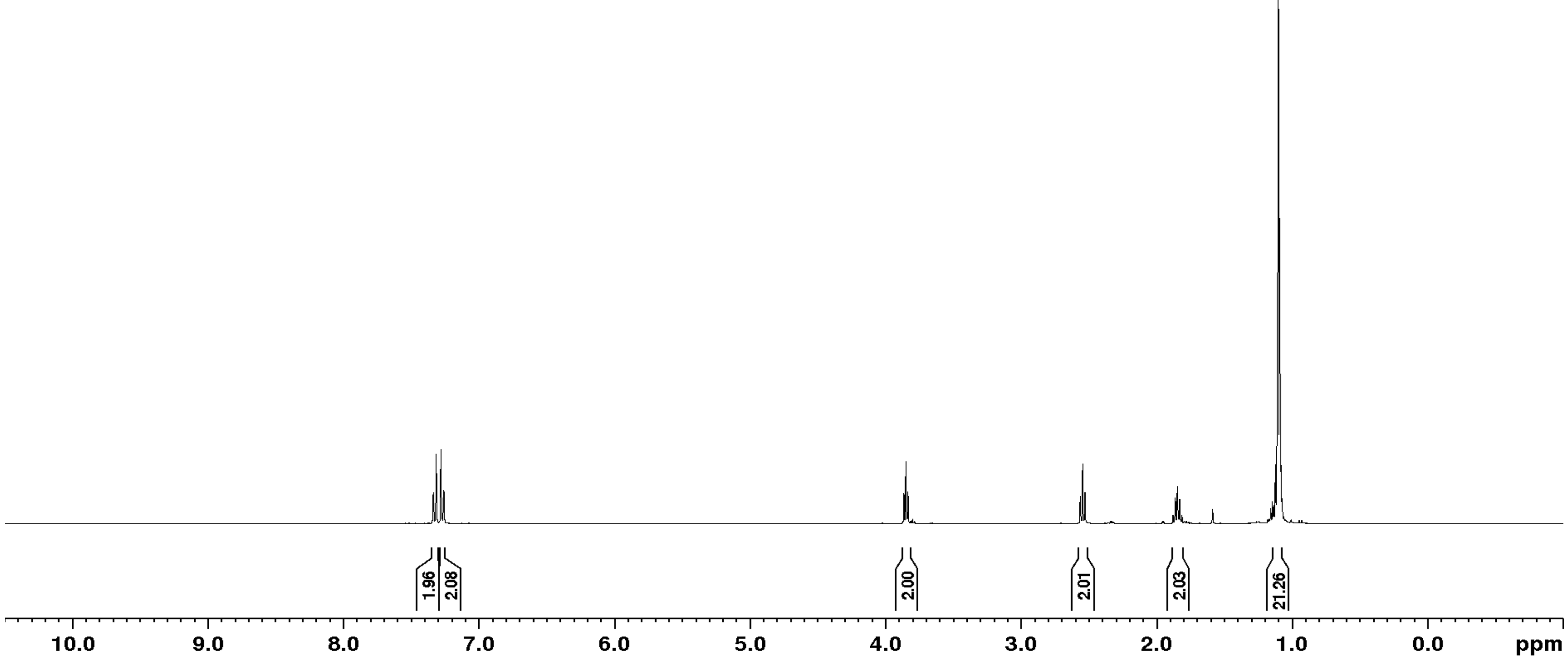

Figure S52. $^{13}C\{^1H\}$ NMR spectrum (101 MHz, $CDCl_3$, 298 K) of ((5-(4-chlorophenyl)pent-4-yn-1-yl)oxy)triisopropylsilane (**1o**)

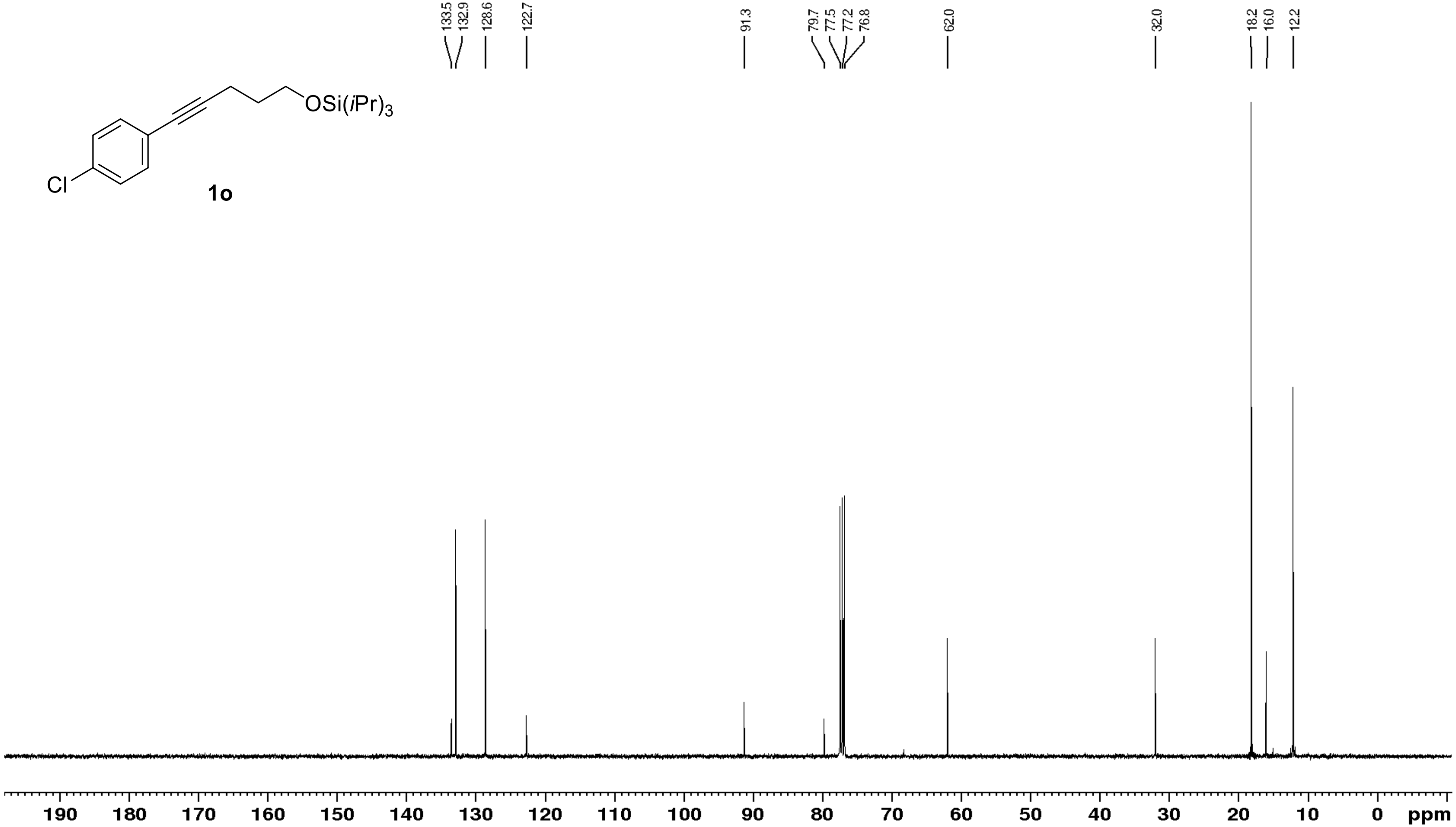

Figure S53. ^{29}Si DEPT NMR spectrum (79 MHz, $CDCl_3$, 298 K, optimized for J = 15.0 Hz) of ((5-(4-chlorophenyl)pent-4-yn-1-yl)oxy)triisopropylsilane (**1o**)

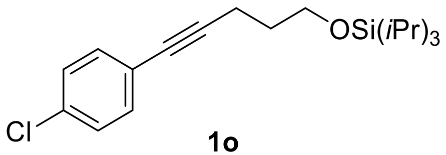

1o

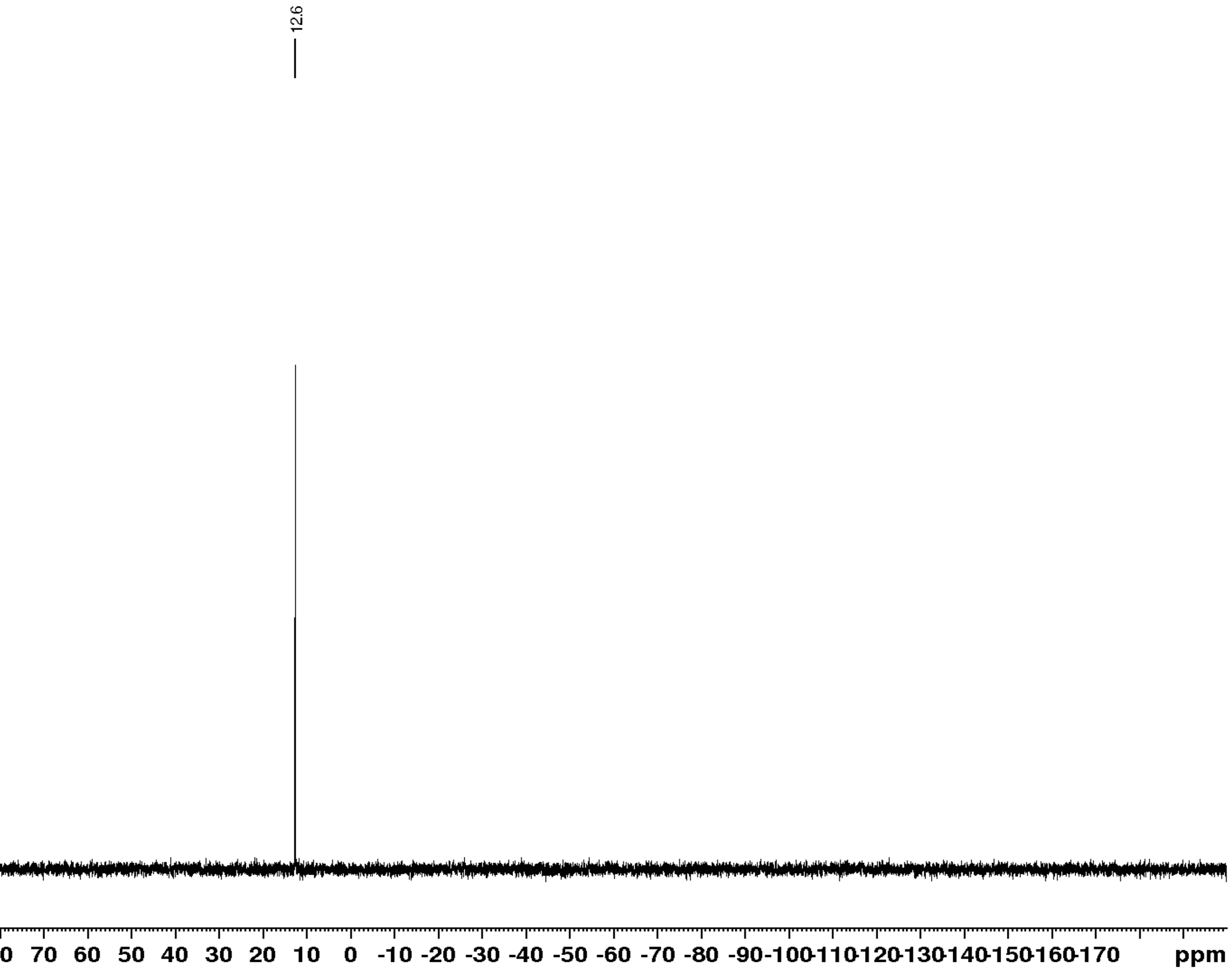

Figure S54. ^{1}H NMR spectrum (400 MHz, $CDCl_3$, 298 K) of ((5-(4-bromophenyl)pent-4-yn-1-yl)oxy)triisopropylsilane (**1p**)

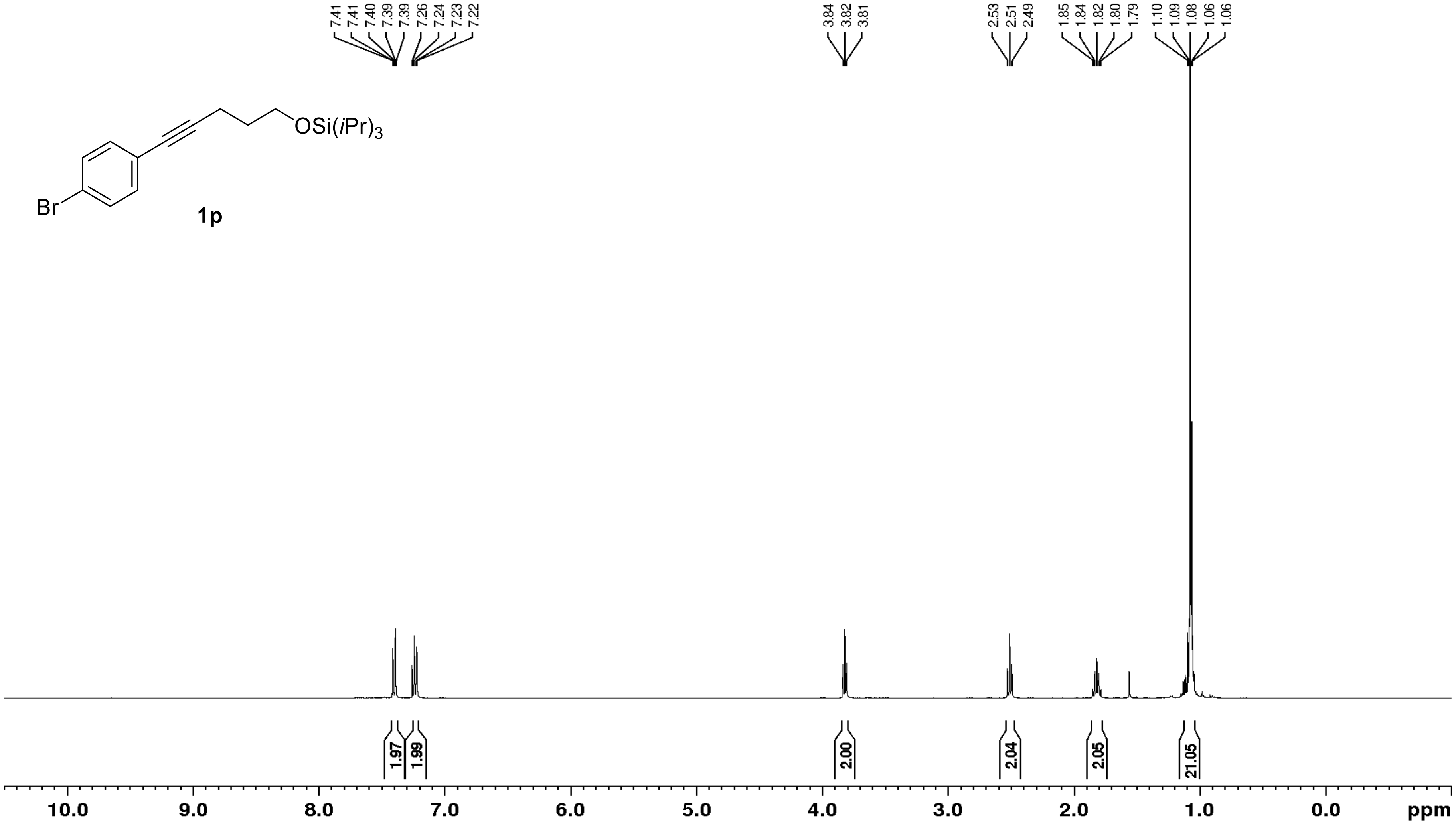

Figure S55. $^{13}C\{^1H\}$ NMR spectrum (101 MHz, $CDCl_3$, 298 K) of ((5-(4-bromophenyl)pent-4-yn-1-yl)oxy)triisopropylsilane (**1p**)

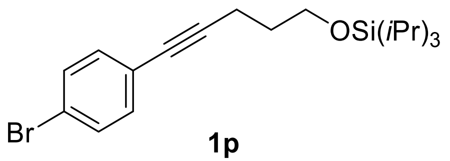

1p

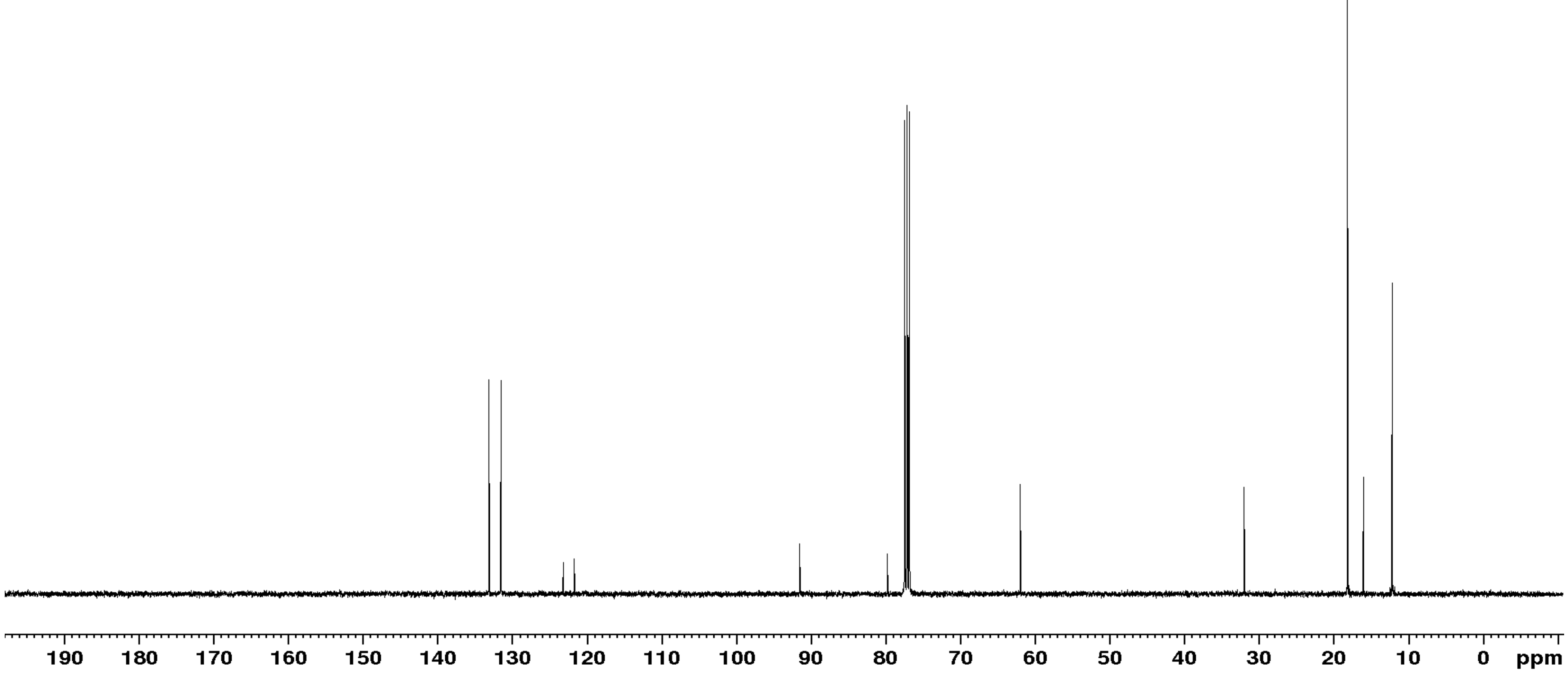

Figure S56. ^{29}Si DEPT NMR spectrum (79 MHz, $CDCl_3$, 298 K, optimized for J = 15.0 Hz) of ((5-(4-bromophenyl)pent-4-yn-1-yl)oxy)triisopropylsilane (**1p**)

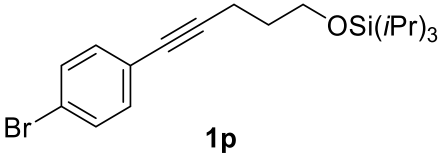

1p

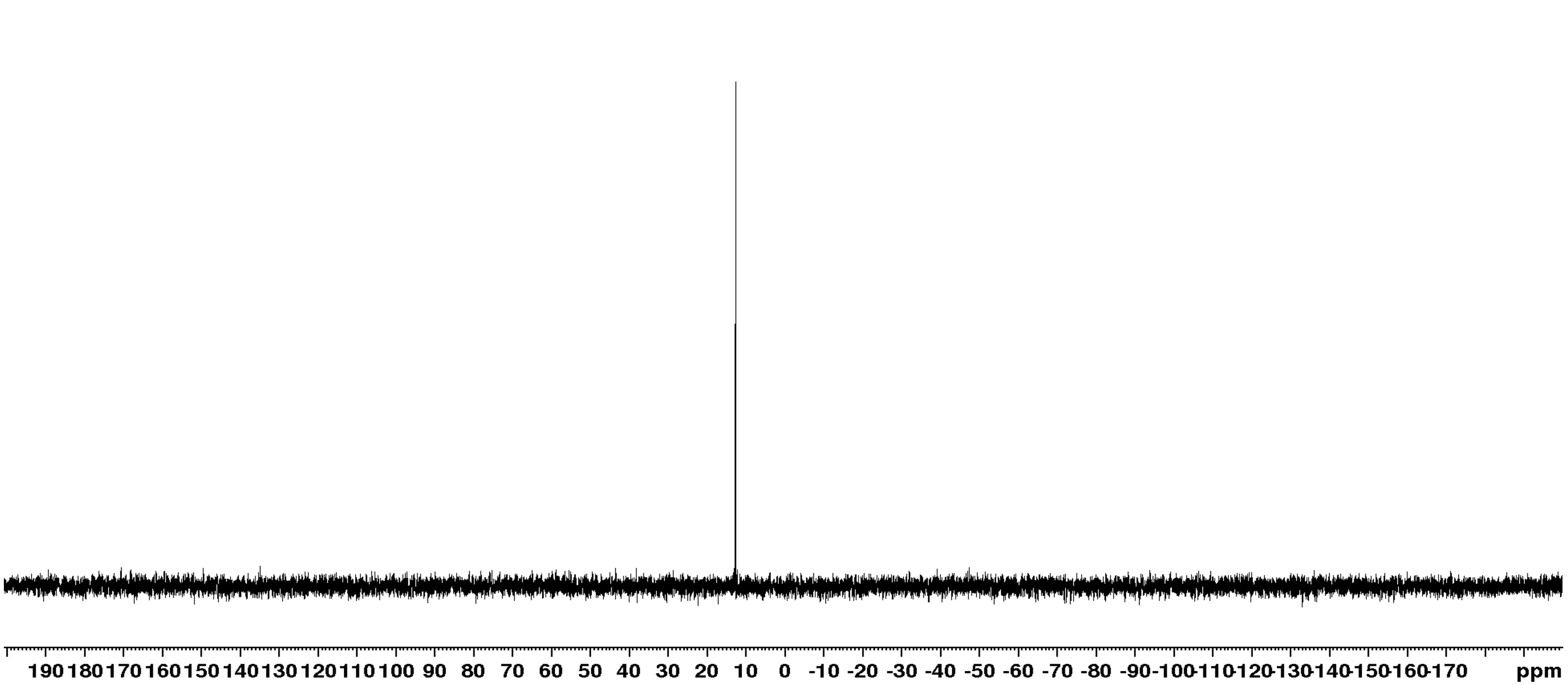

Figure S57. ^{1}H NMR spectrum (400 MHz, $CDCl_3$, 298 K) of triisopropyl((5-(naphthalen-2-yl)pent-4-yn-1-yl)oxy)silane (**1q**)

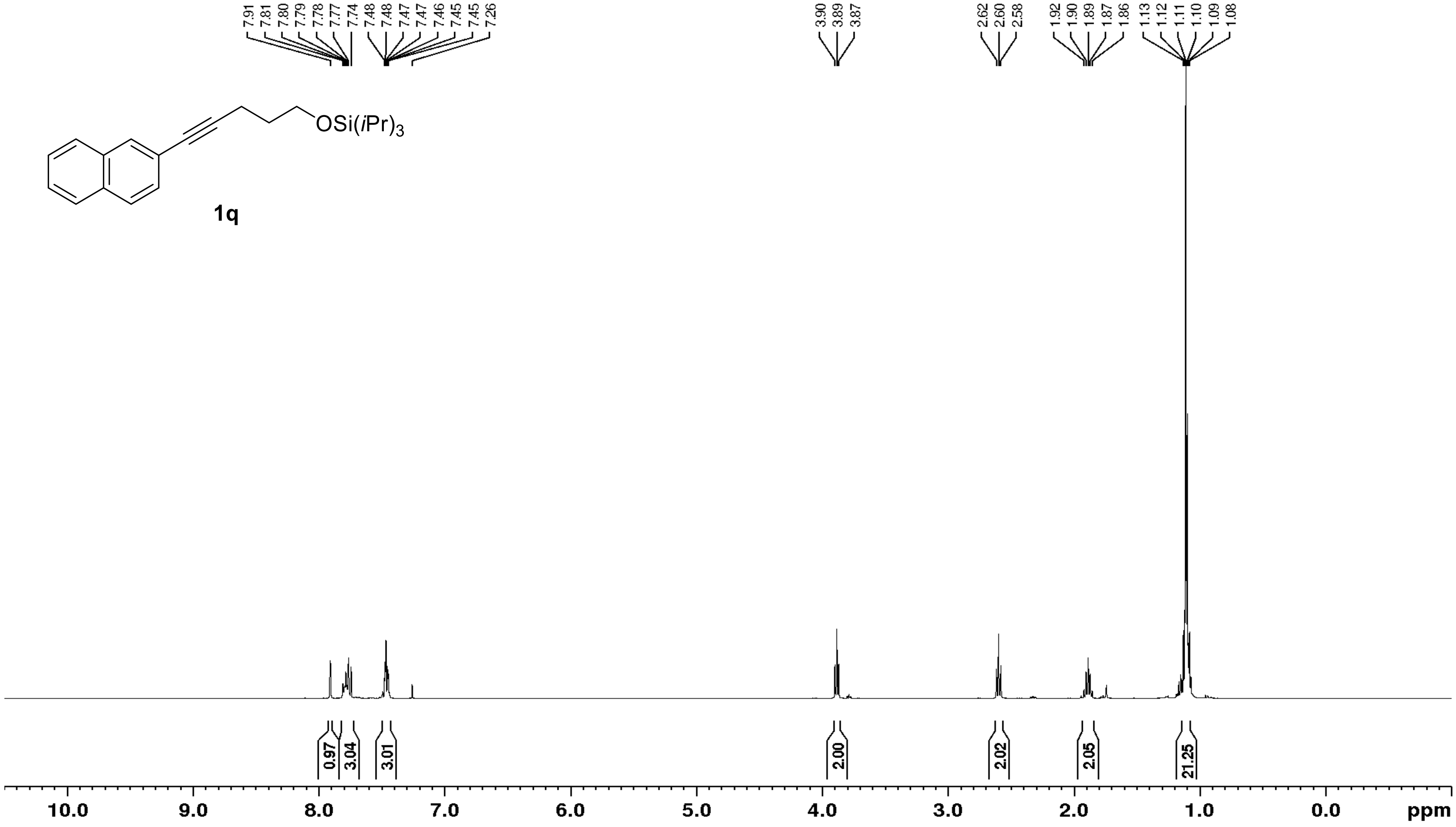

Figure S58. $^{13}C\{^1H\}$ NMR spectrum (101 MHz, $CDCl_3$, 298 K) of triisopropyl((5-(naphthalen-2-yl)pent-4-yn-1-yl)oxy)silane (**1q**)

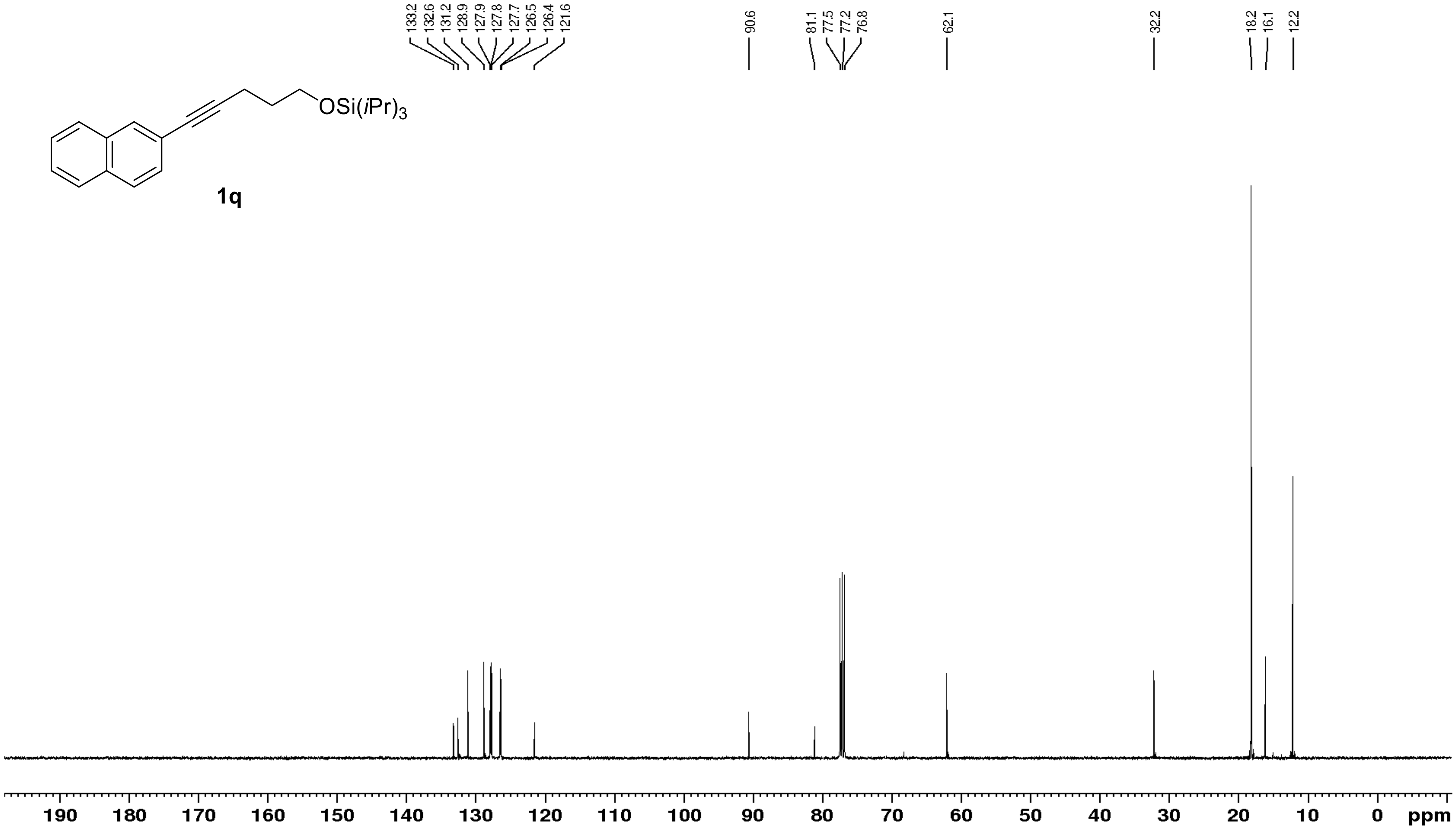

Figure S59. ^{29}Si DEPT NMR spectrum (79 MHz, $CDCl_3$, 298 K, optimized for J = 15.0 Hz) of triisopropyl((5-(naphthalen-2-yl)pent-4-yn-1-yl)oxy)silane (**1q**)

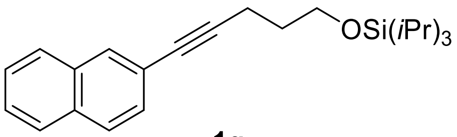

1q

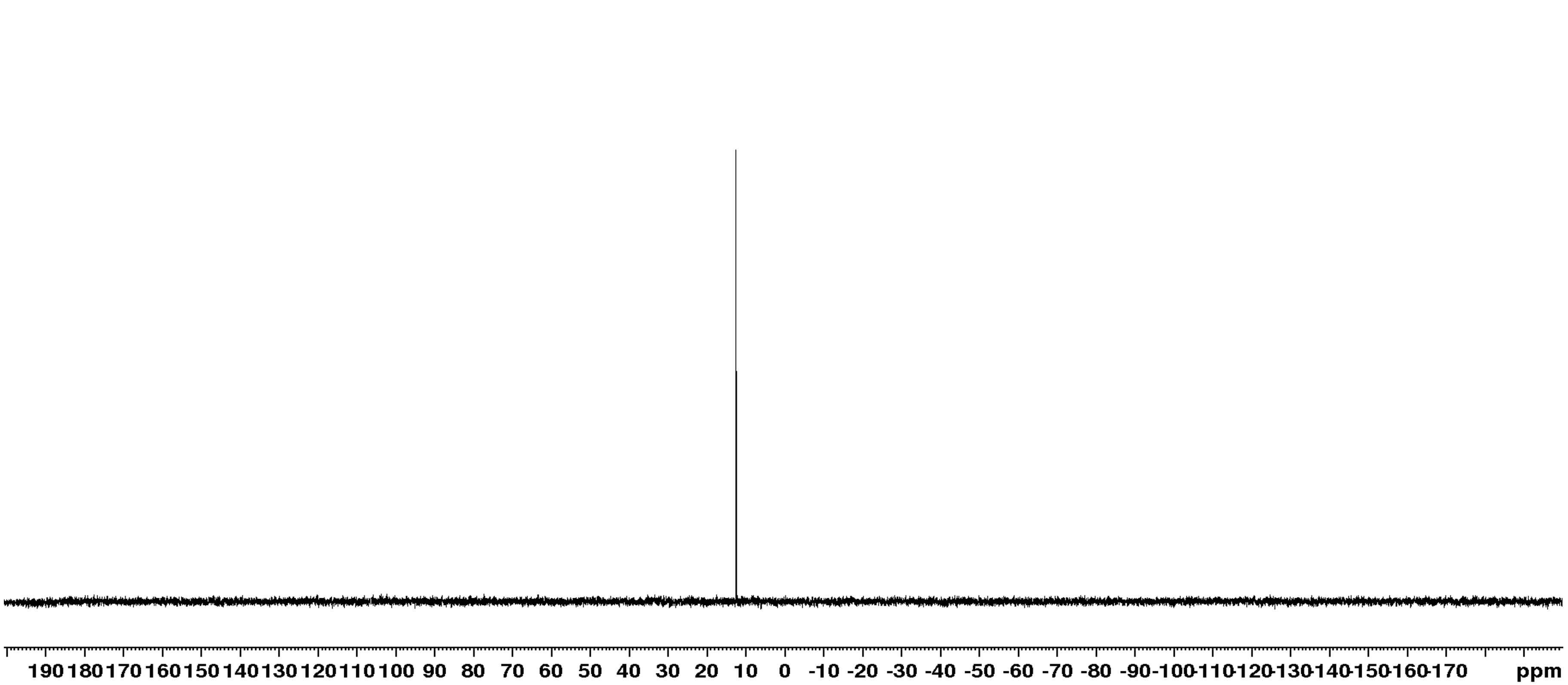

Figure S60. ^{1}H NMR spectrum (400 MHz, $CDCl_3$, 298 K) of ((5-(benzo[*b*]thiophen-2-yl)pent-4-yn-1-yl)oxy)triisopropylsilane (**1r**)

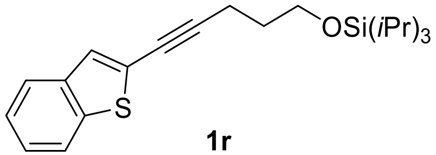

1r

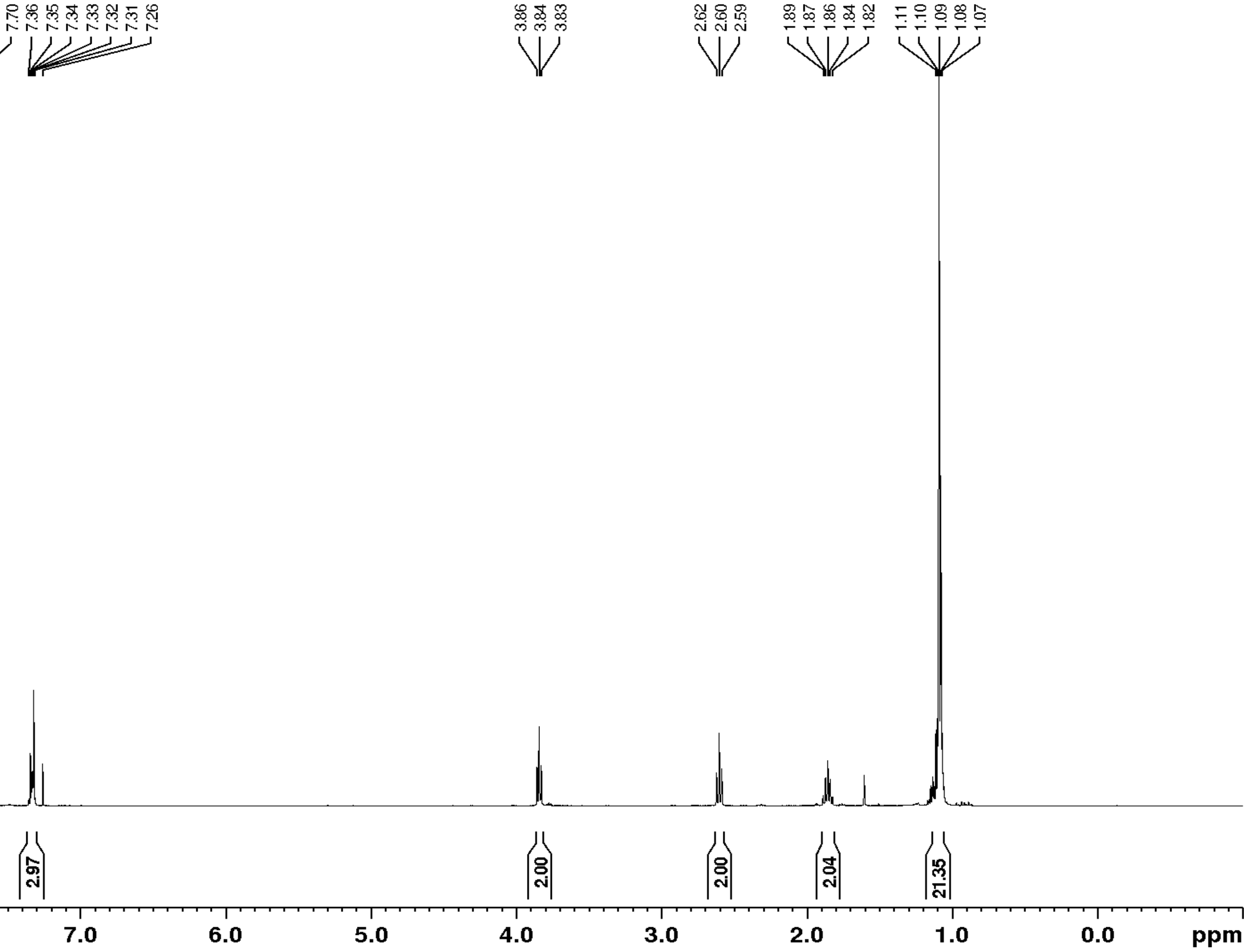

Figure S61. $^{13}C\{^{1}H\}$ NMR spectrum (101 MHz, $CDCl_3$, 298 K) of ((5-(benzo[*b*]thiophen-2-yl)pent-4-yn-1-yl)oxy)triisopropylsilane (**1r**)

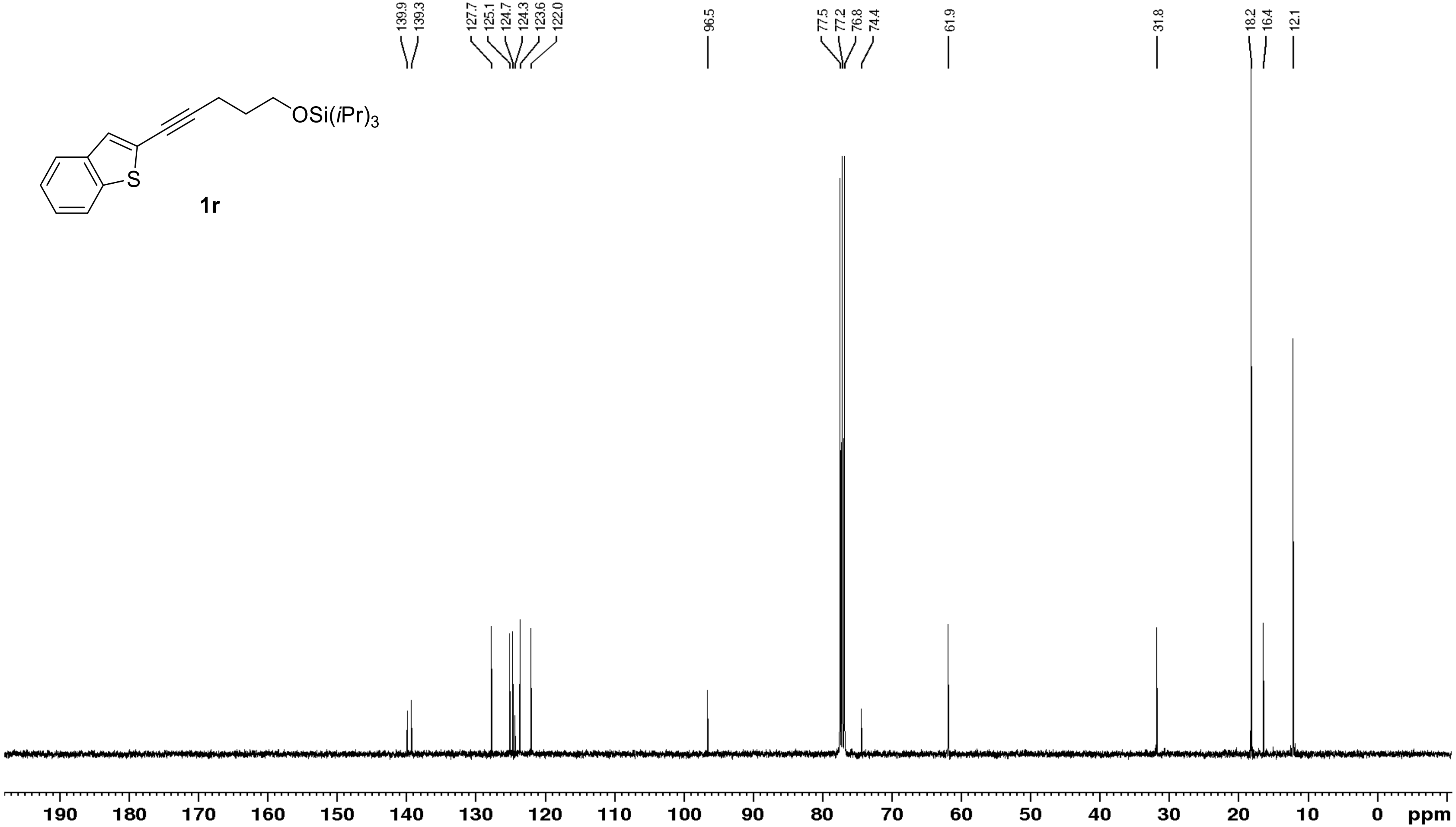

Figure S62. ^{29}Si DEPT NMR spectrum (79 MHz, $CDCl_3$, 298 K, optimized for J = 15.0 Hz) of ((5-(benzo[*b*]thiophen-2-yl)pent-4-yn-1-yl)oxy)triisopropylsilane (**1r**)

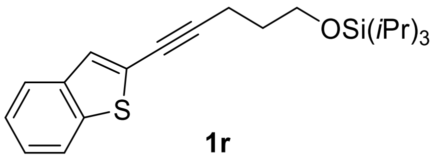

1r

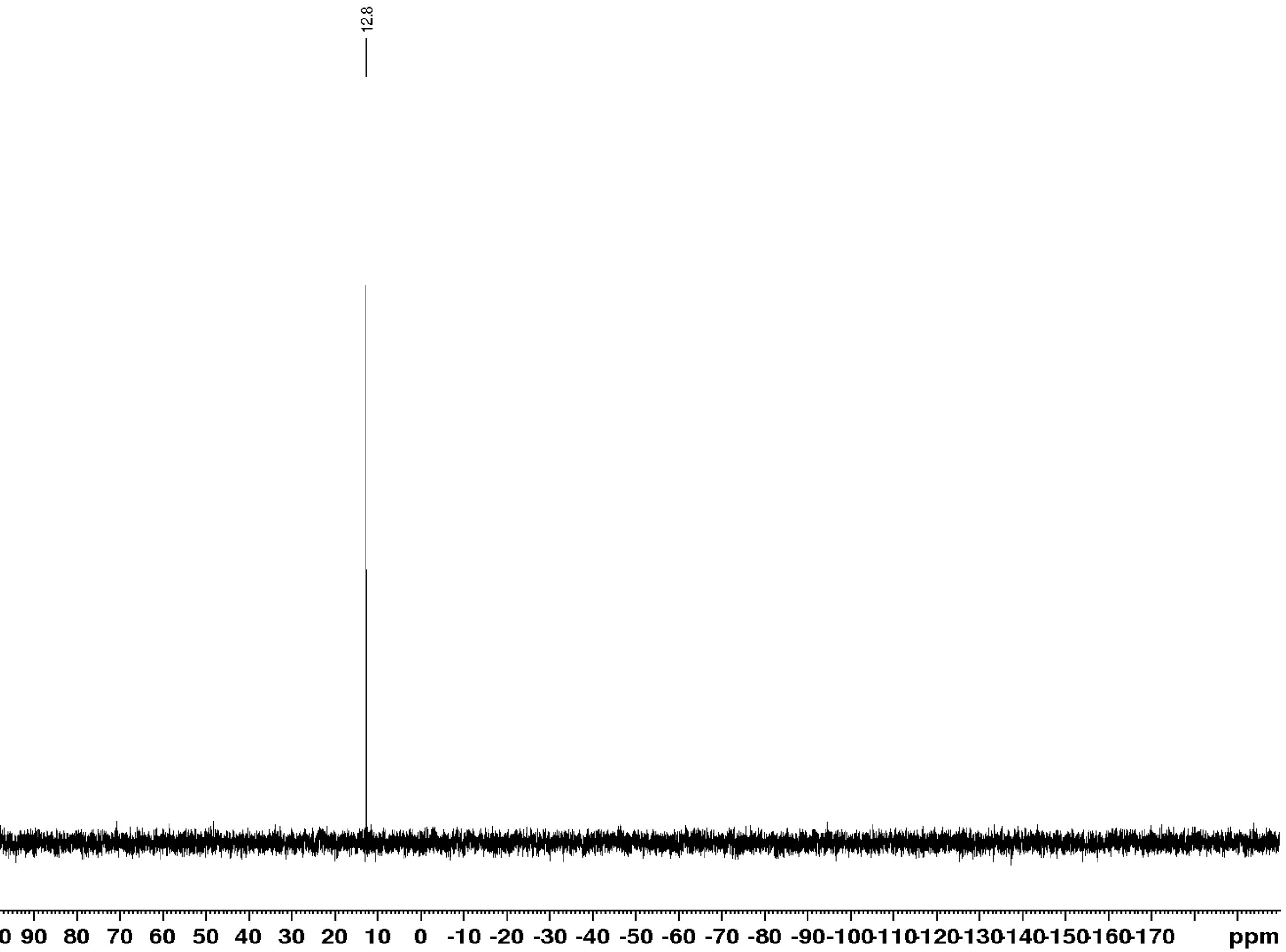

Figure S63. ^{1}H NMR spectrum (400 MHz, $CDCl_3$, 298 K) of triisopropyl((5-(thiophen-2-yl)pent-4-yn-1-yl)oxy)silane (**1s**)

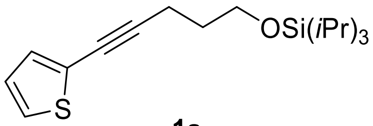

1s

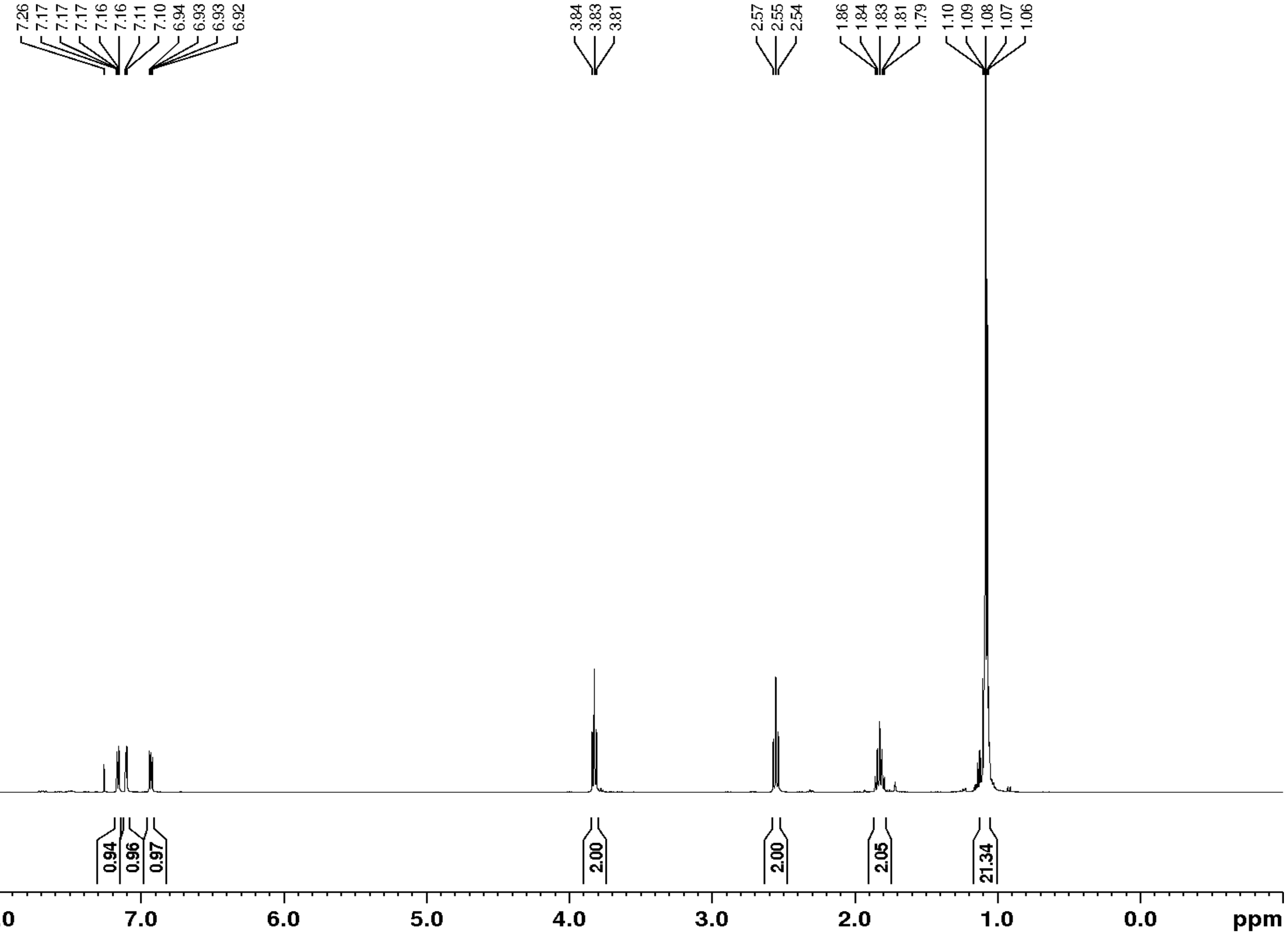

Figure S64. $^{13}C\{^1H\}$ NMR spectrum (101 MHz, $CDCl_3$, 298 K) of triisopropyl((5-(thiophen-2-yl)pent-4-yn-1-yl)oxy)silane (**1s**)

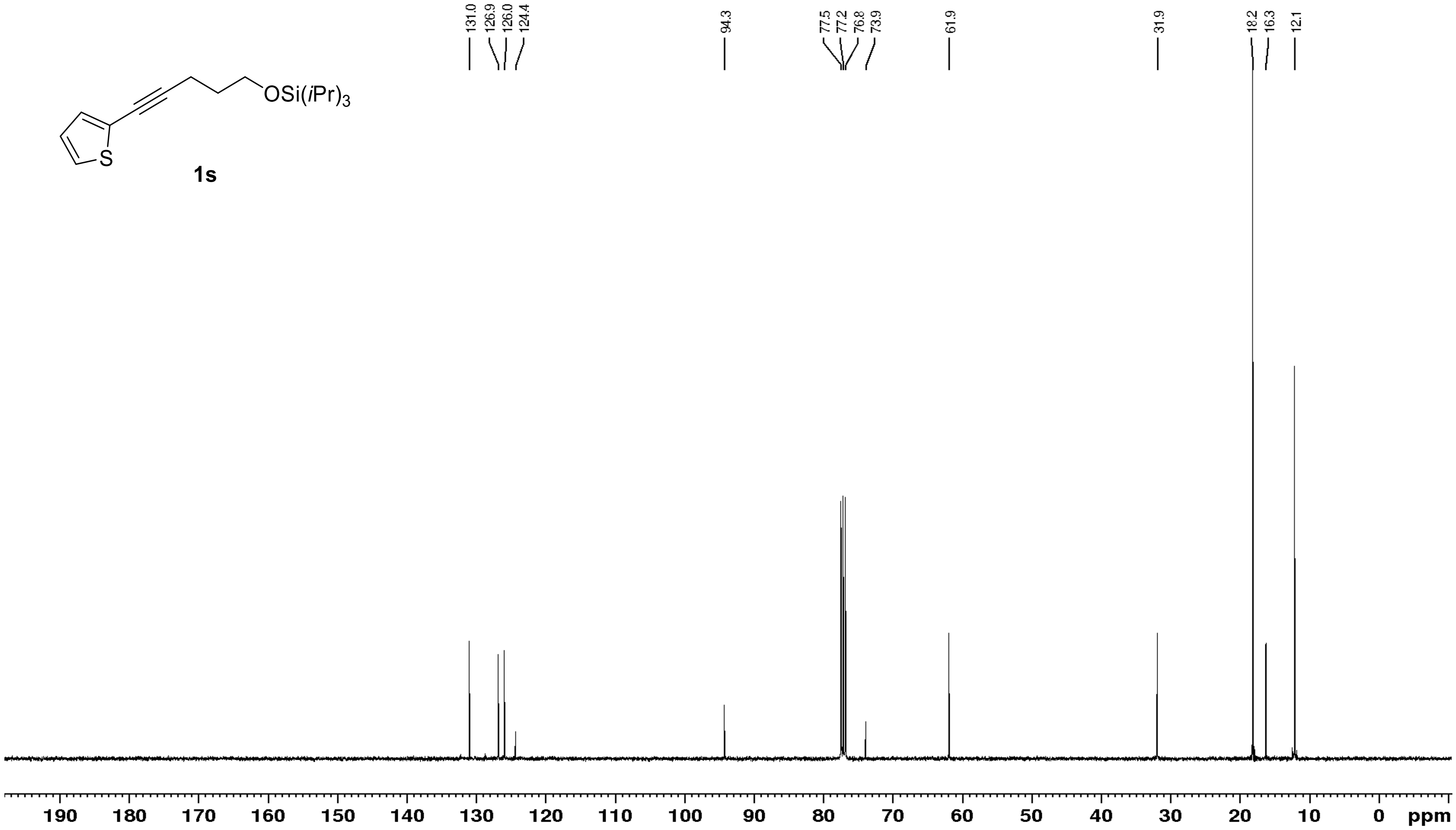

Figure S65. ^{29}Si DEPT NMR spectrum (79 MHz, $CDCl_3$, 298 K, optimized for *J* = 15.0 Hz) of triisopropyl((5-(thiophen-2-yl)pent-4-yn-1-yl)oxy)silane (**1s**)

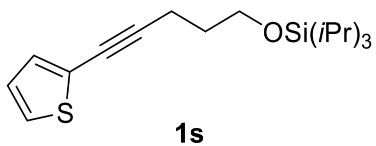

1s

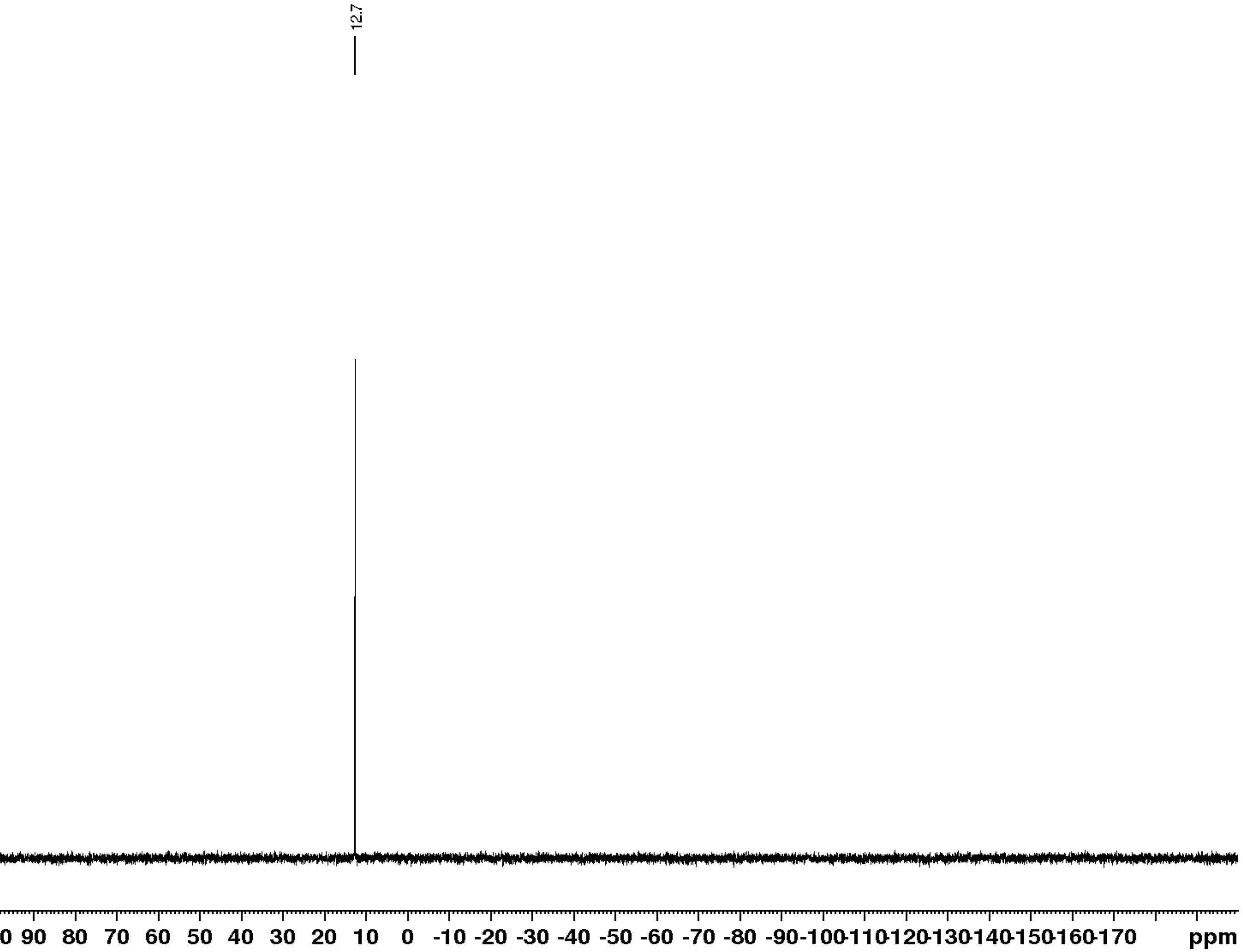

Figure S66. ^{1}H NMR spectrum (400 MHz, $CDCl_3$, 298 K) of diisopropyl(methyl)((5-phenylpent-4-yn-1-yl)oxy)silane (**1u**)

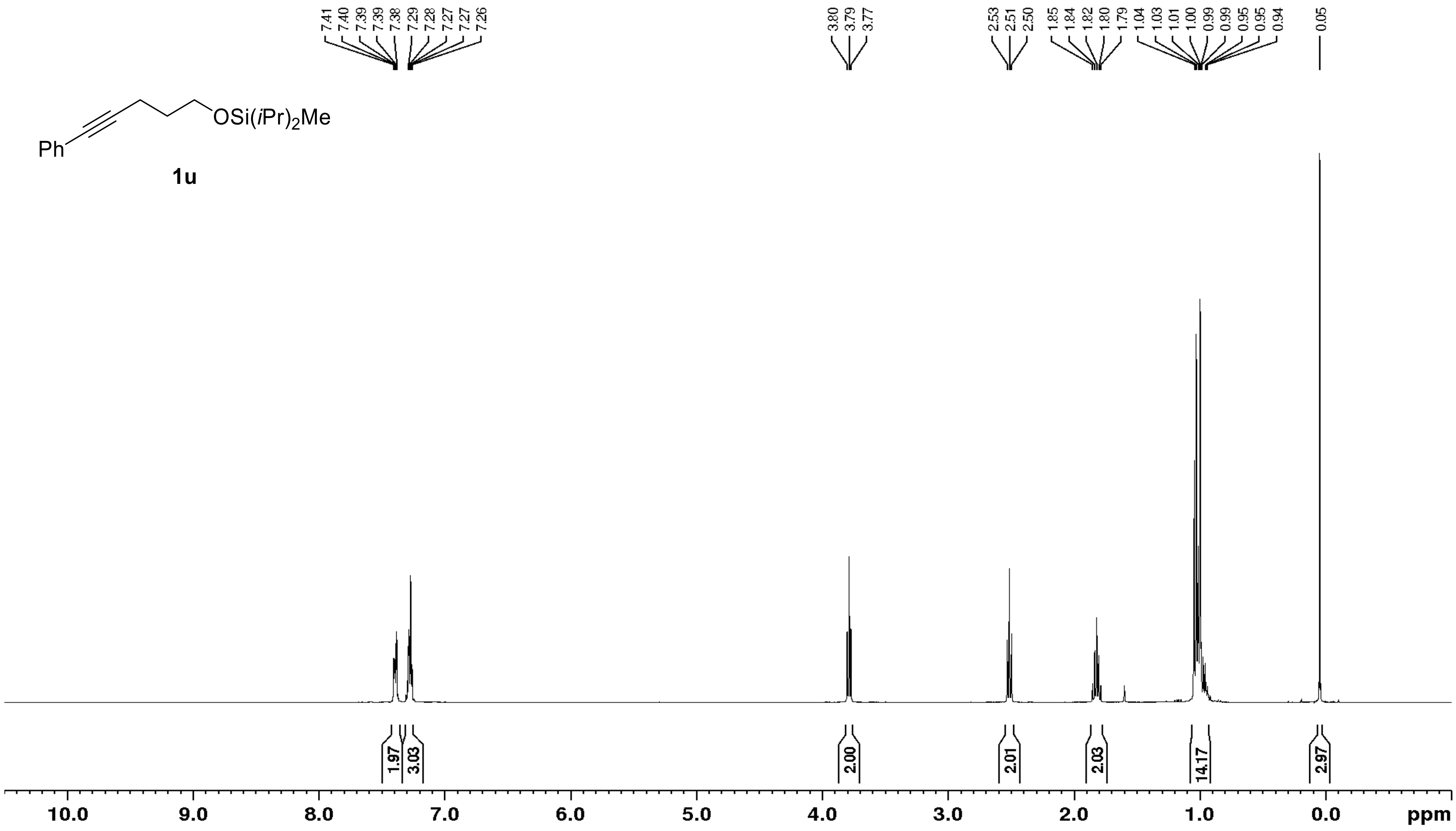

Figure S67. $^{13}C\{^{1}H\}$ NMR spectrum (101 MHz, $CDCl_3$, 298 K) of diisopropyl(methyl)((5-phenylpent-4-yn-1-yl)oxy)silane (**1u**)

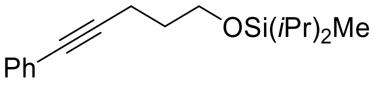

1u

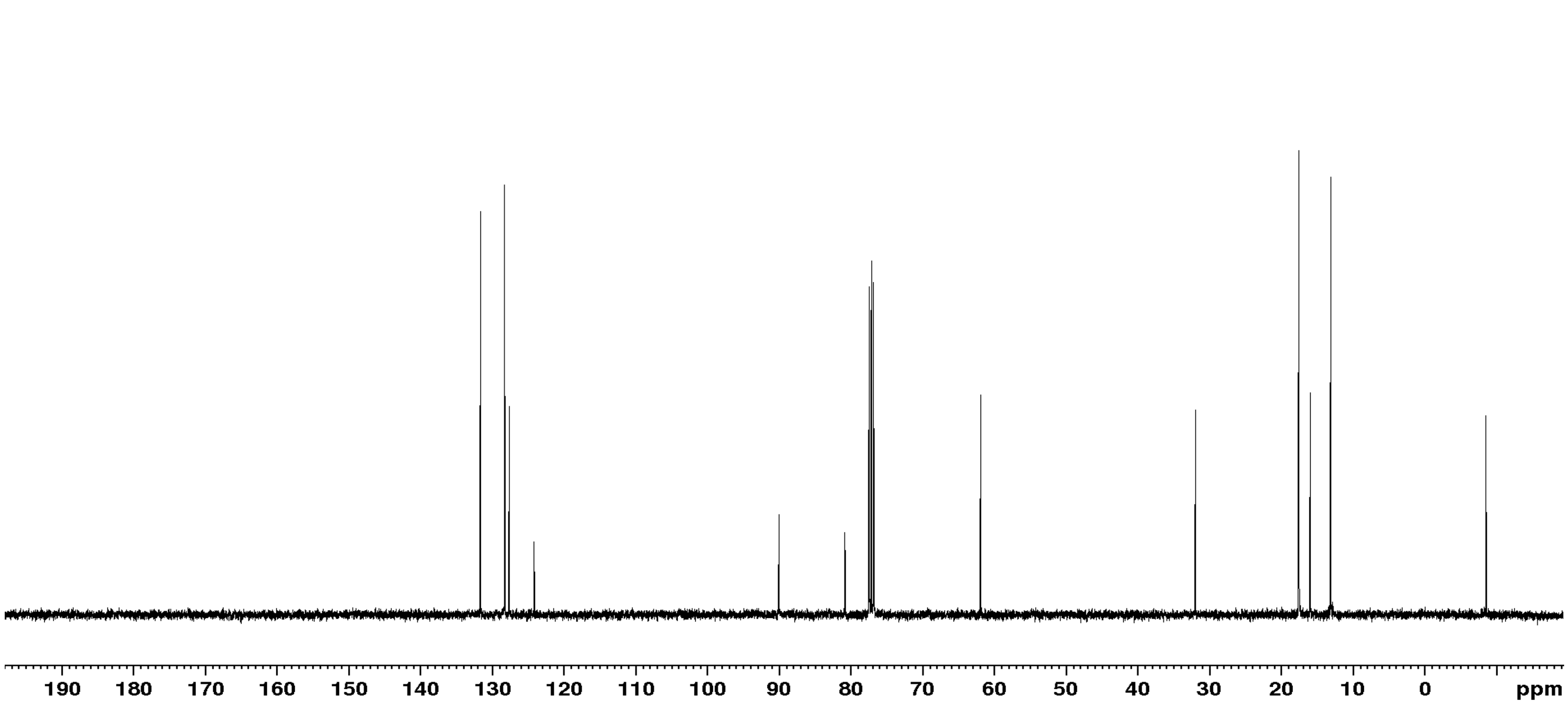

Figure S68. ^{29}Si DEPT NMR spectrum (79 MHz, $CDCl_3$, 298 K, optimized for J = 15.0 Hz) of diisopropyl(methyl)((5-phenylpent-4-yn-1-yl)oxy)silane (**1u**)

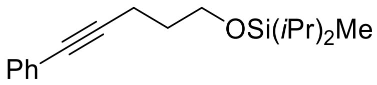

1u

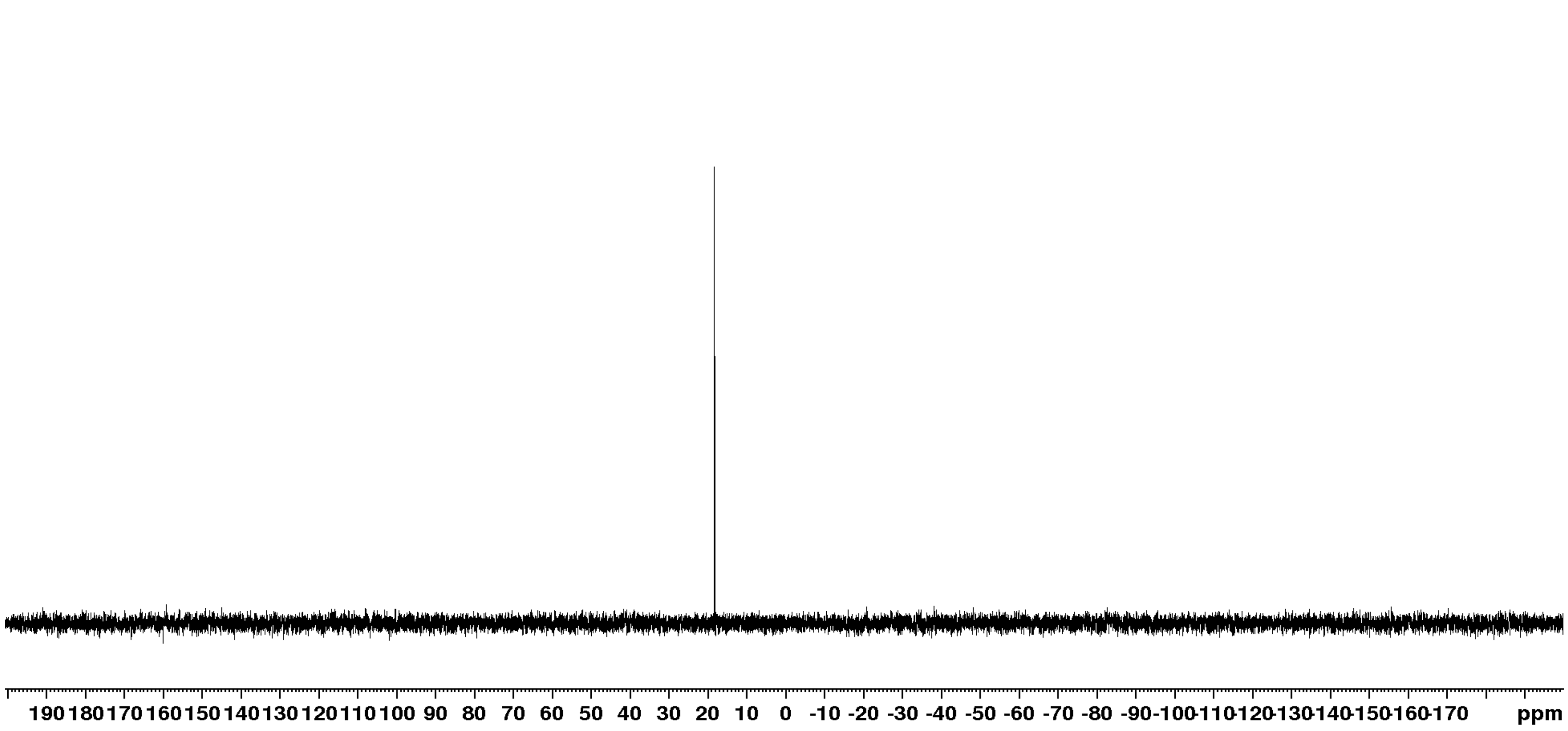

Figure S69. ^{1}H NMR spectrum (400 MHz, $CDCl_3$, 298 K) of *tert*-butyldimethyl((5-phenylpent-4-yn-1-yl)oxy)silane (**1v**)

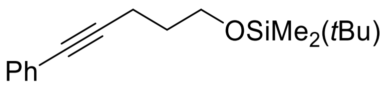

1v

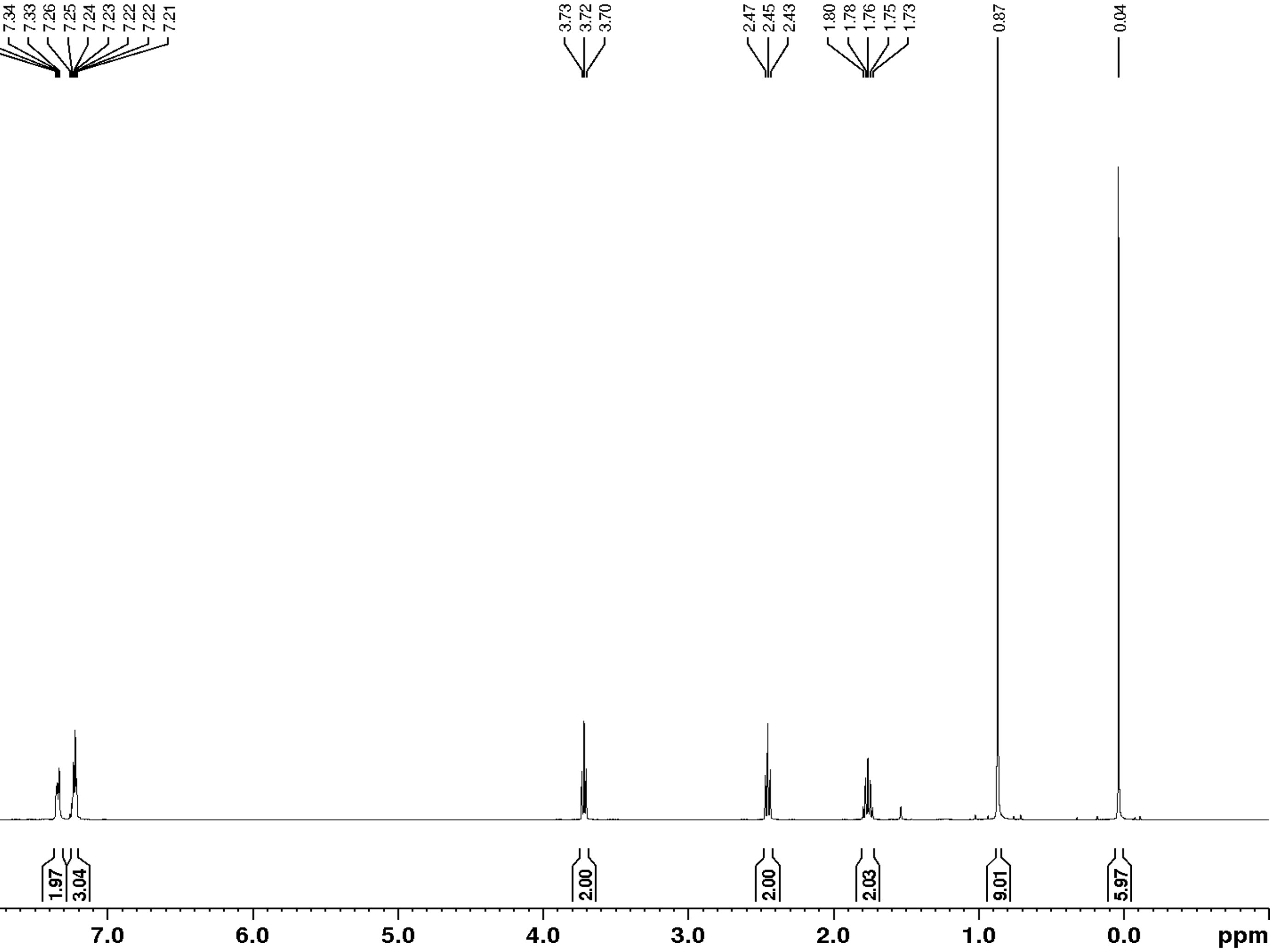

Figure S70. $^{13}C\{^{1}H\}$ NMR spectrum (101 MHz, $CDCl_3$, 298 K) of *tert*-butyldimethyl((5-phenylpent-4-yn-1-yl)oxy)silane (**1v**)

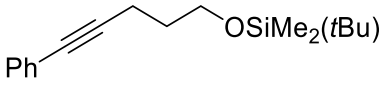

1v

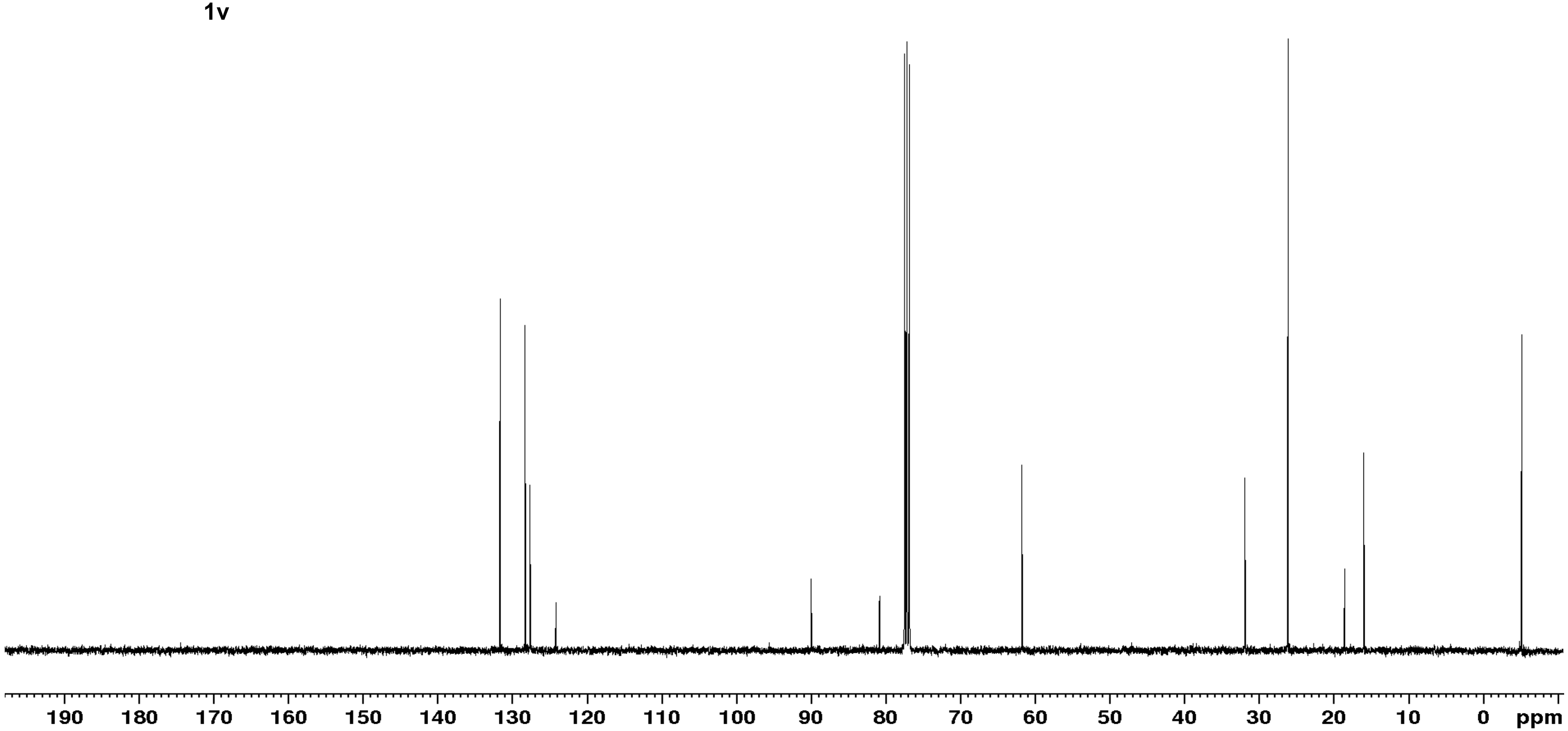

Figure S71. ^{29}Si DEPT NMR spectrum (79 MHz, $CDCl_3$, 298 K, optimized for J = 15.0 Hz) of *tert*-butyldimethyl((5-phenylpent-4-yn-1-yl)oxy)silane (**1v**)

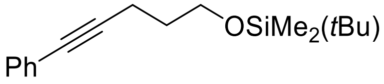

1v

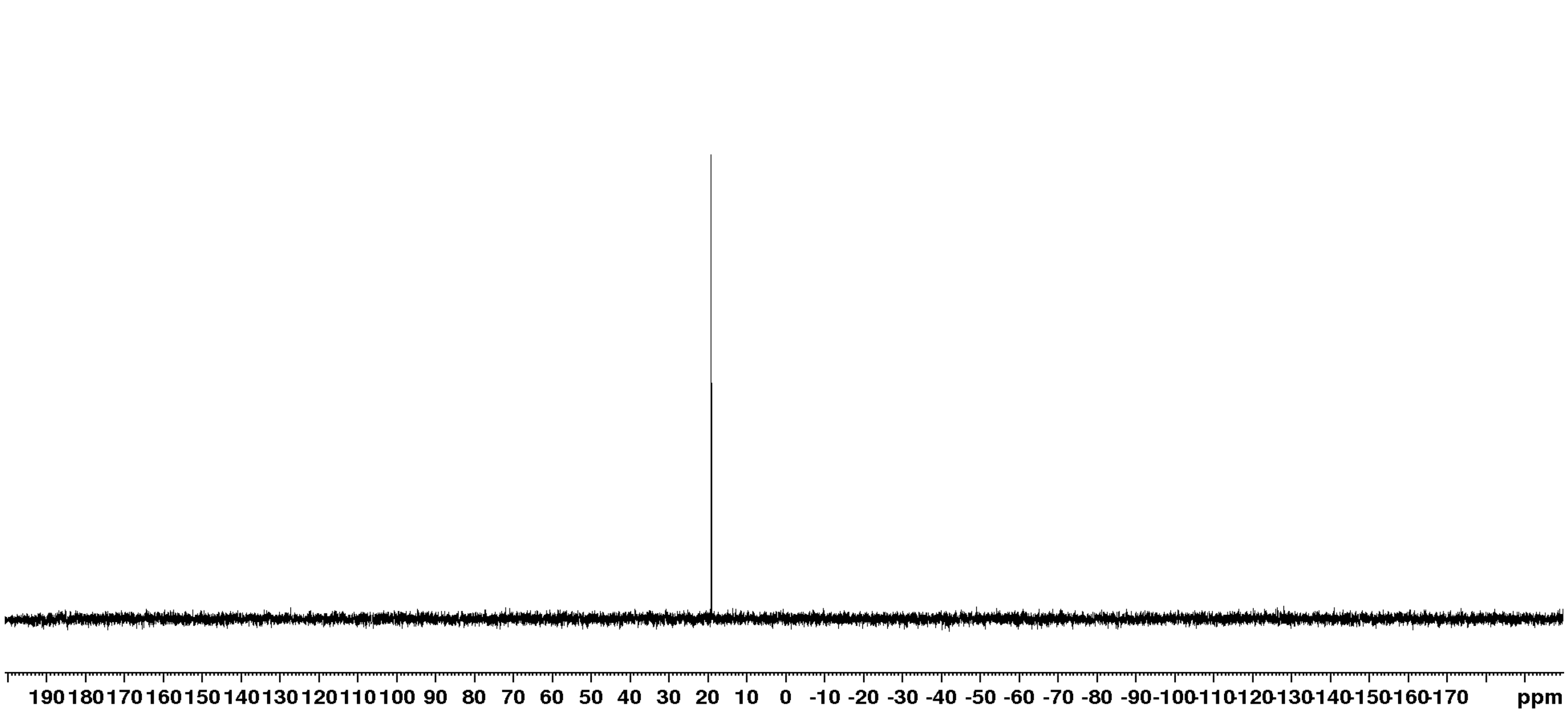

Figure S72. ^{1}H NMR spectrum (400 MHz, $CDCl_3$, 298 K) of (2,3-dimethylbutan-2-yl)dimethyl((5-phenylpent-4-yn-1-yl)oxy)silane (**1w**)

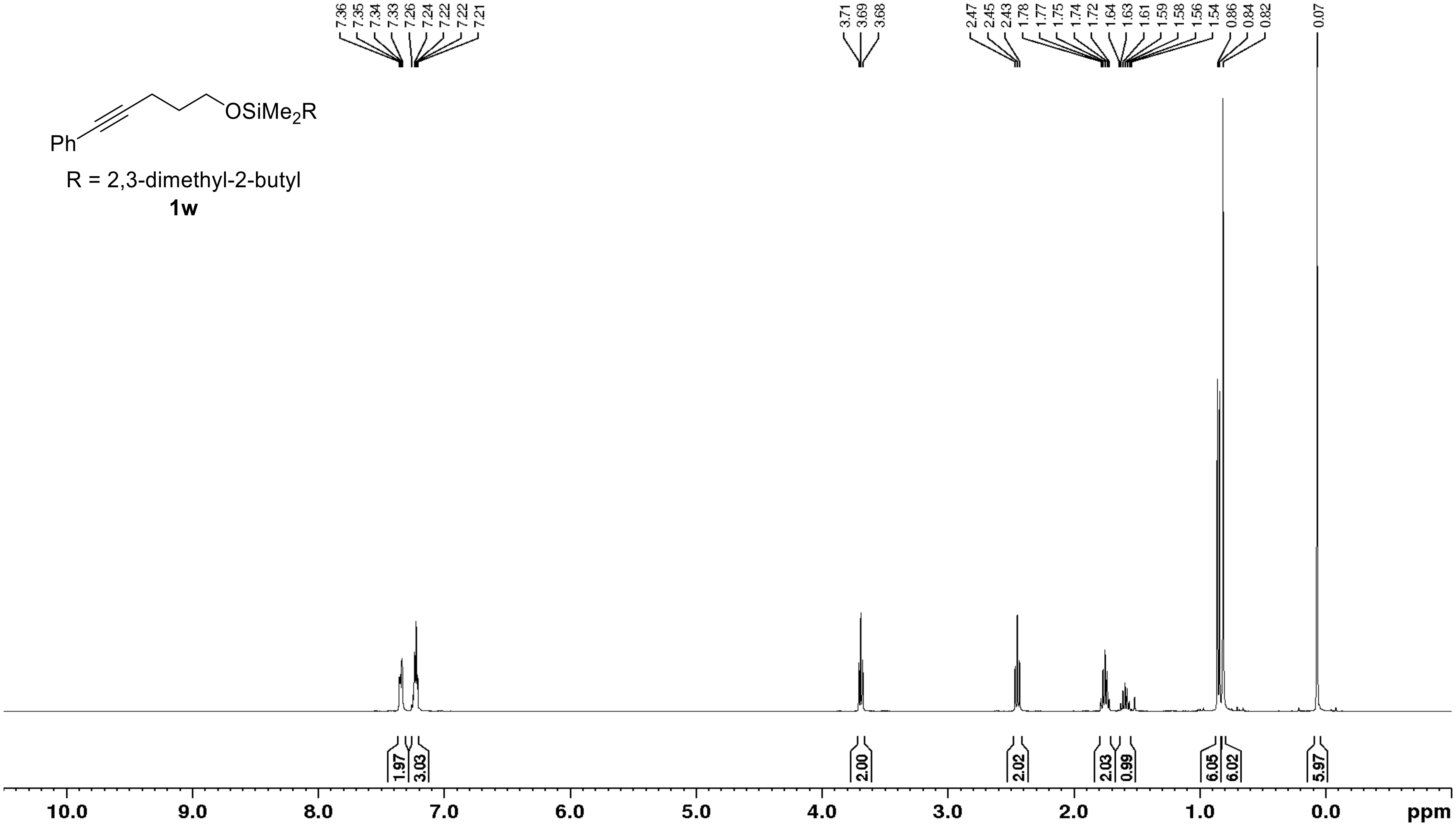

Figure S73. $^{13}C\{^1H\}$ NMR spectrum (101 MHz, $CDCl_3$, 298 K) of (2,3-dimethylbutan-2-yl)dimethyl((5-phenylpent-4-yn-1-yl)oxy)silane (**1w**)

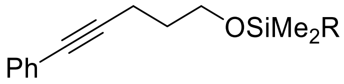

R = 2,3-dimethyl-2-butyl
1w

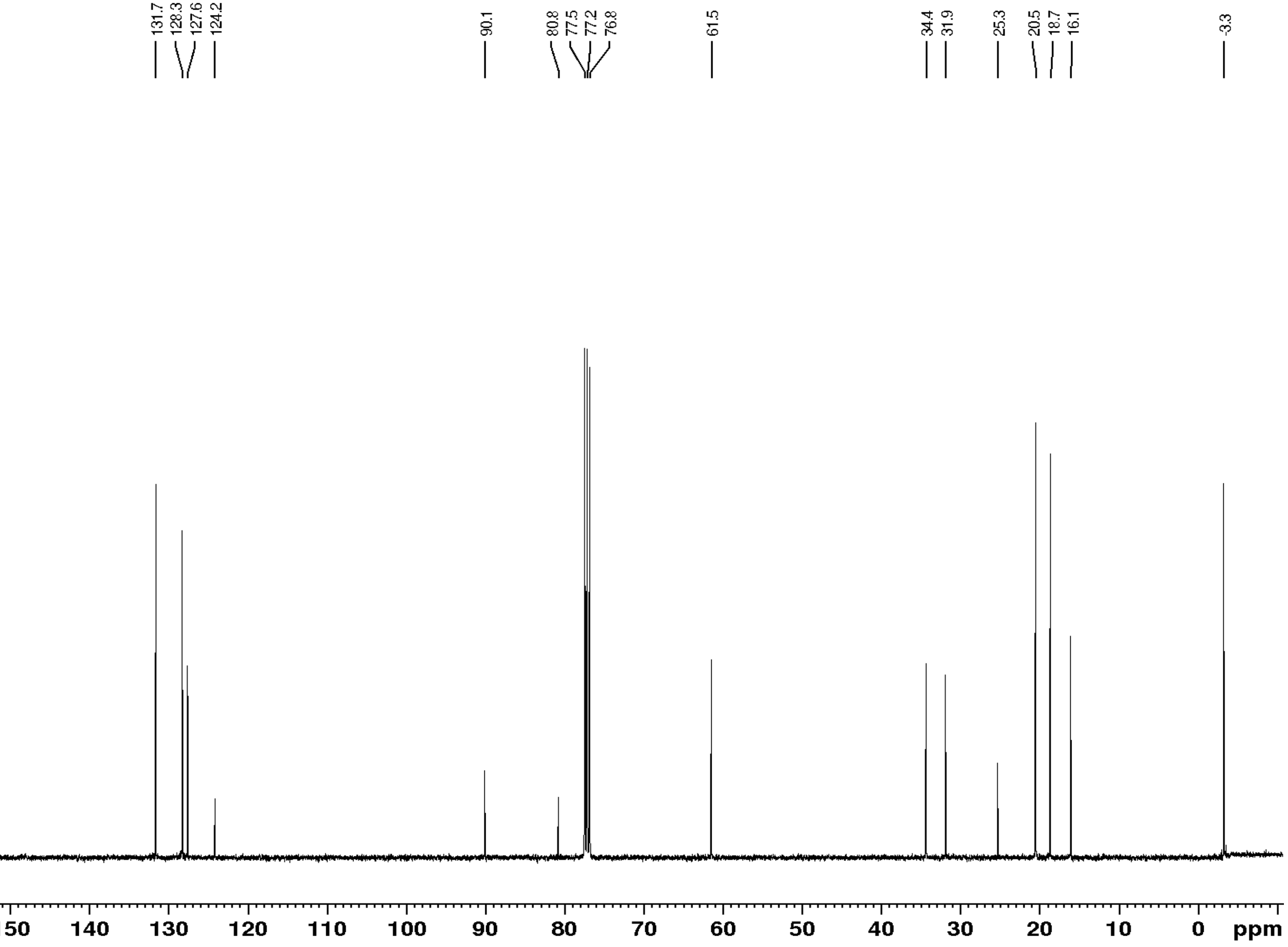

Figure S74. ^{29}Si DEPT NMR spectrum (79 MHz, $CDCl_3$, 298 K, optimized for *J* = 15.0 Hz) of (2,3-dimethylbutan-2-yl)dimethyl((5-phenylpent-4-yn-1-yl)oxy)silane (**1r**)

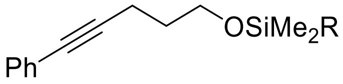

R = 2,3-dimethyl-2-butyl
1w

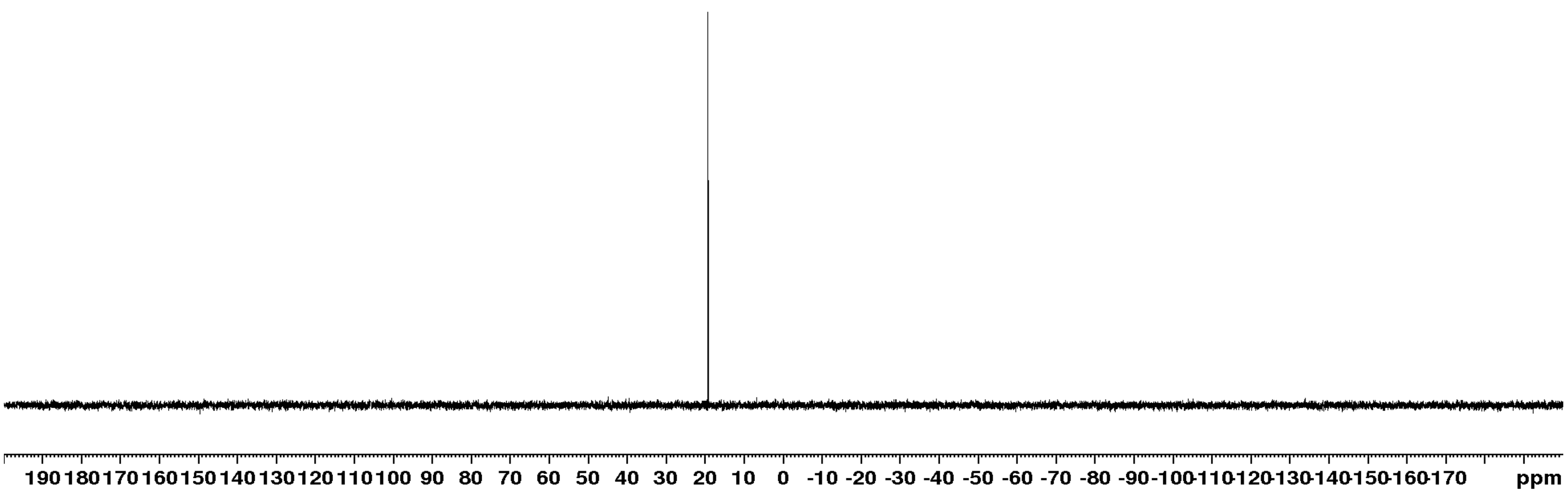

Figure S75. ^{1}H NMR spectrum (500 MHz, $CDCl_3$, 298 K) of trimethyl(5-methylhexa-1,2-dien-3-yl)silane (**2j**)

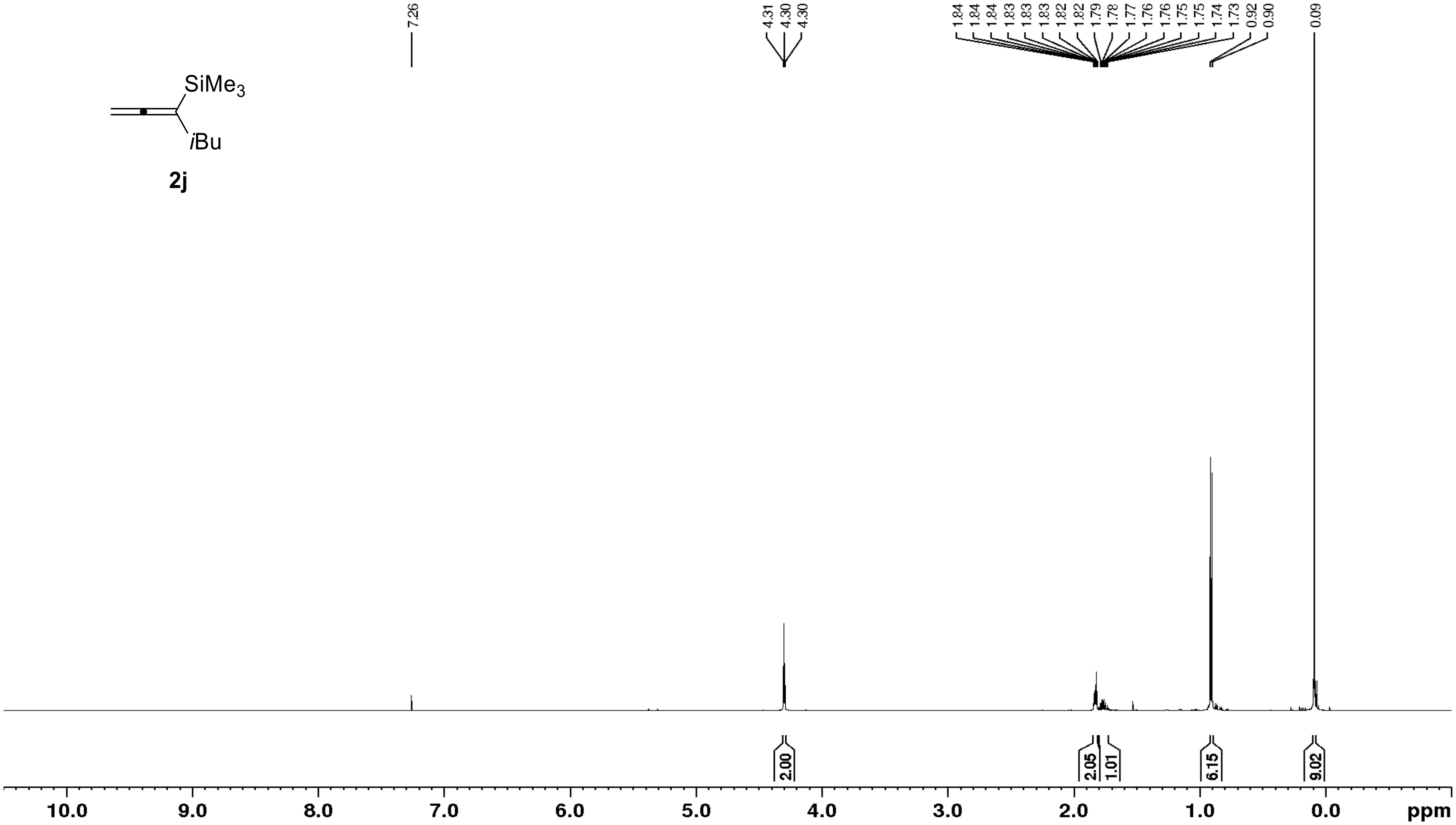

Figure S76. $^{13}C\{^{1}H\}$ NMR spectrum (126 MHz, $CDCl_3$, 298 K) of trimethyl(5-methylhexa-1,2-dien-3-yl)silane (**2j**)

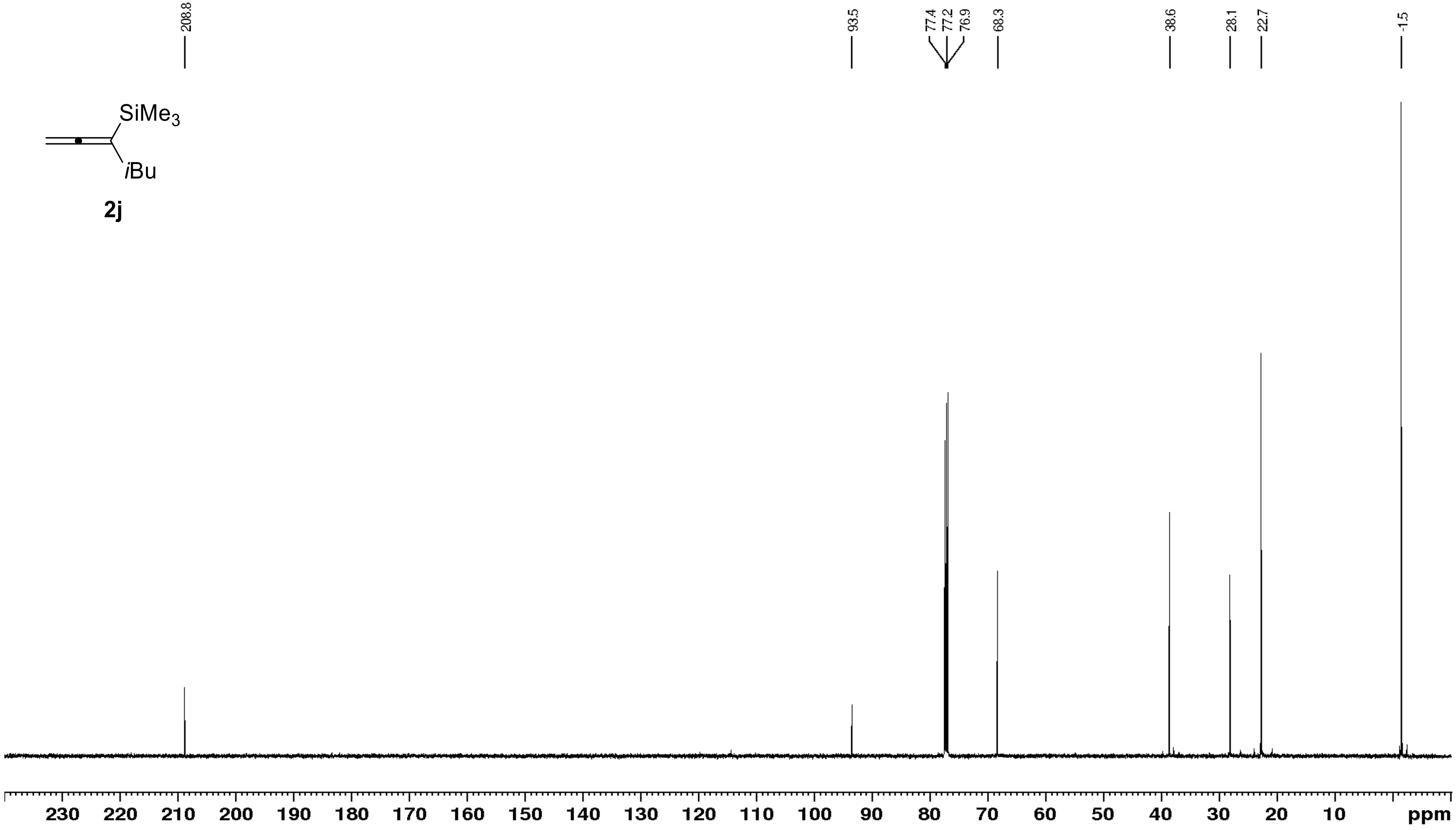

Figure S77. ^{29}Si DEPT NMR spectrum (99 MHz, $CDCl_3$, 298 K, optimized for *J* = 7.0 Hz) of trimethyl(5-methylhexa-1,2-dien-3-yl)silane (**2j**)

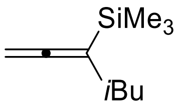

2j

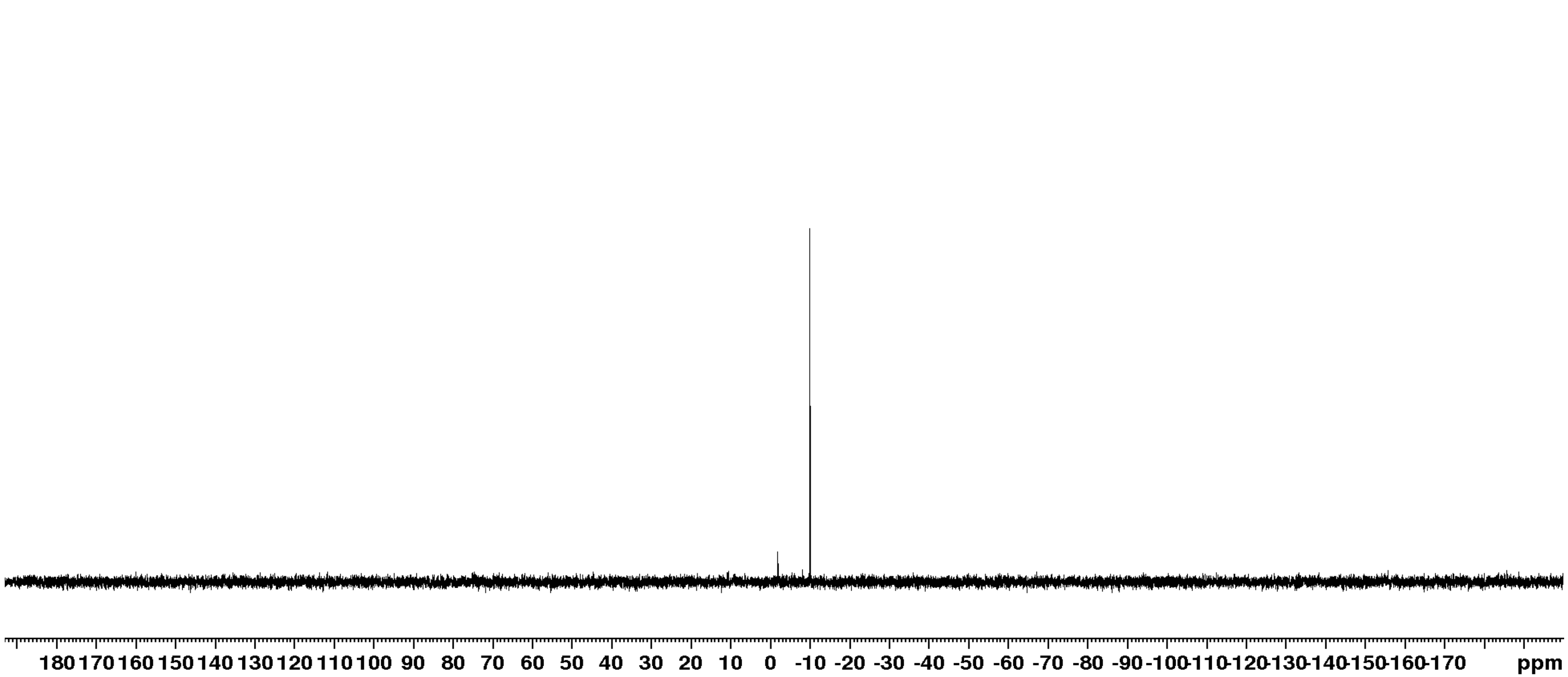

Figure S78. ^{1}H NMR spectrum (500 MHz, $CDCl_3$, 298 K) of trimethyl(4-methylpenta-1,2-dien-3-yl)silane (**2l**)

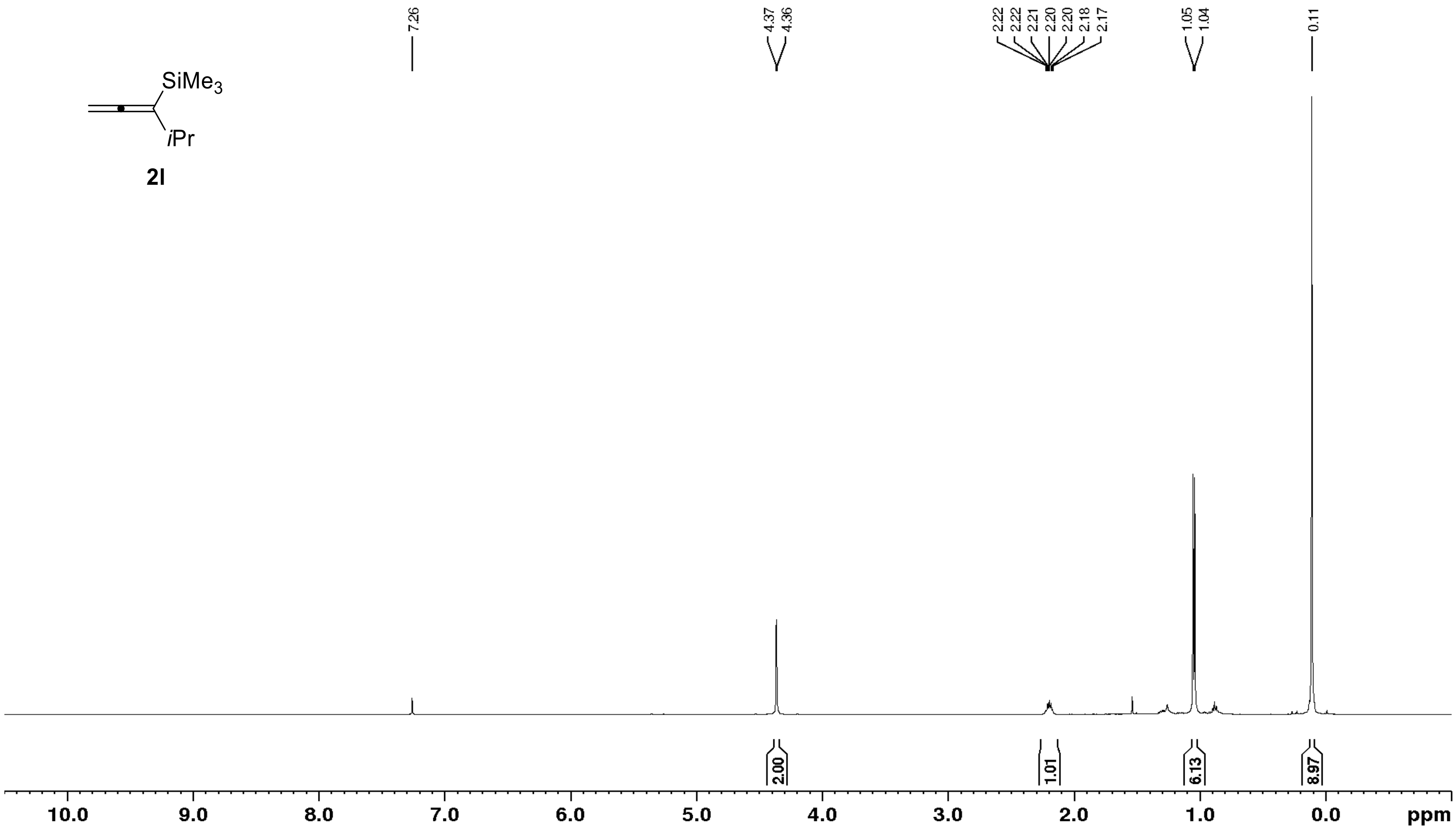

Figure S79. $^{13}C\{^{1}H\}$ NMR spectrum (101 MHz, $CDCl_3$, 298 K) of trimethyl(4-methylpenta-1,2-dien-3-yl)silane (**2l**)

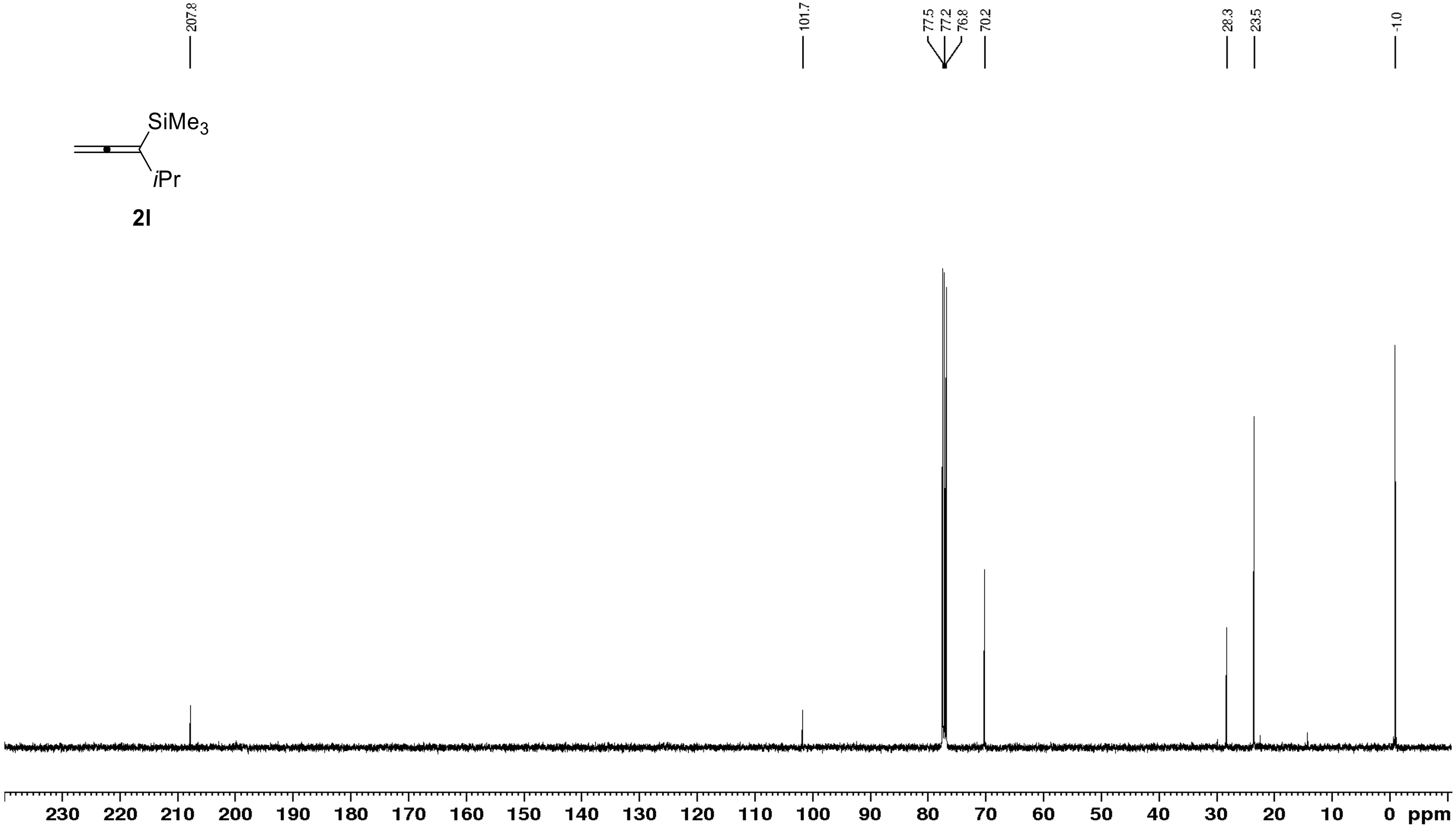

Figure S80. ^{29}Si DEPT NMR spectrum (79 MHz, $CDCl_3$, 298 K, optimized for *J* = 7.0 Hz) of trimethyl(4-methylpenta-1,2-dien-3-yl)silane (**2l**)

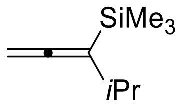

2l

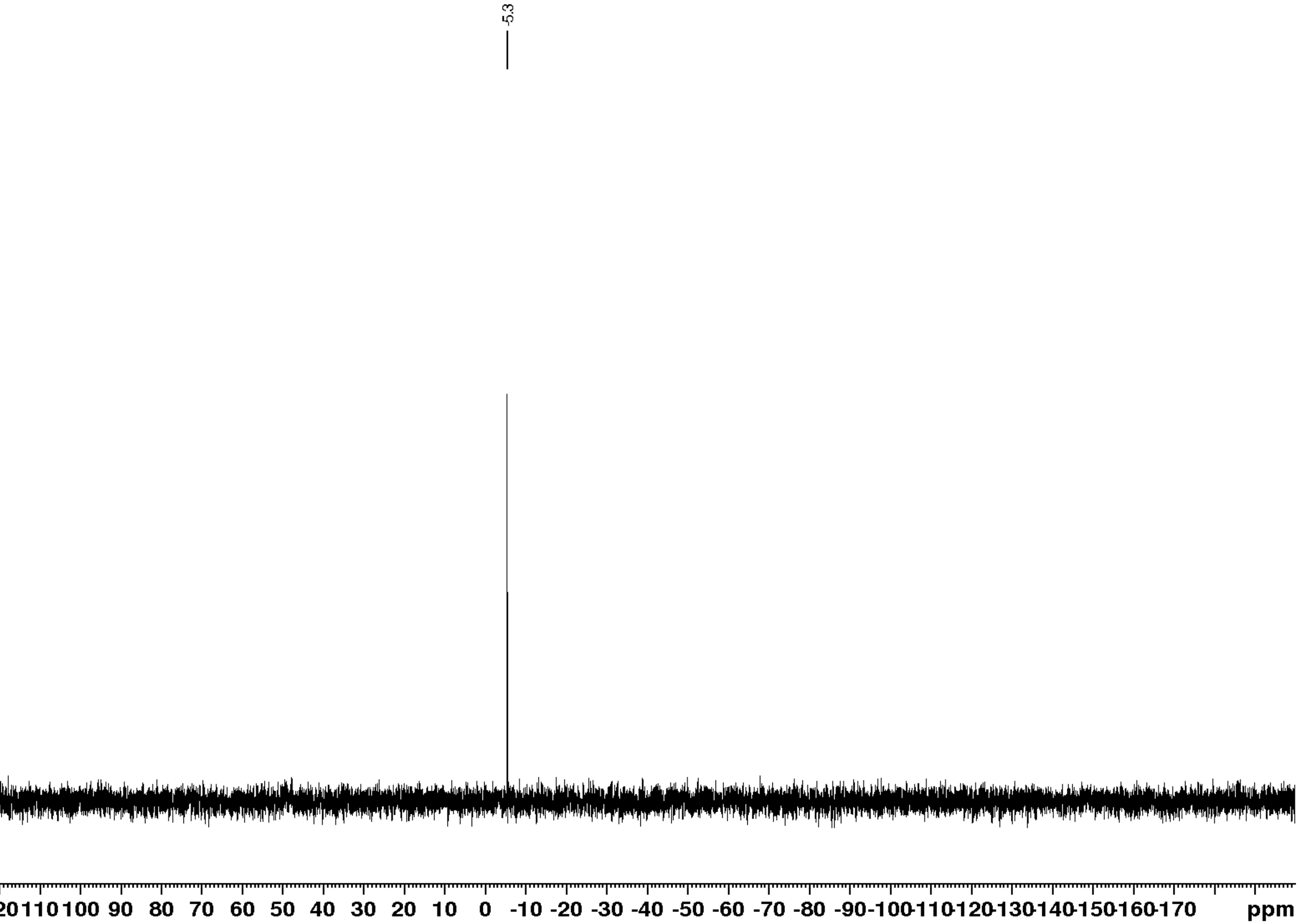

Figure S81. ^{1}H NMR spectrum (500 MHz, $CDCl_3$, 298 K) of **3aa** obtained from the reaction of silyl ether **1a** and allenylsilane **2a**

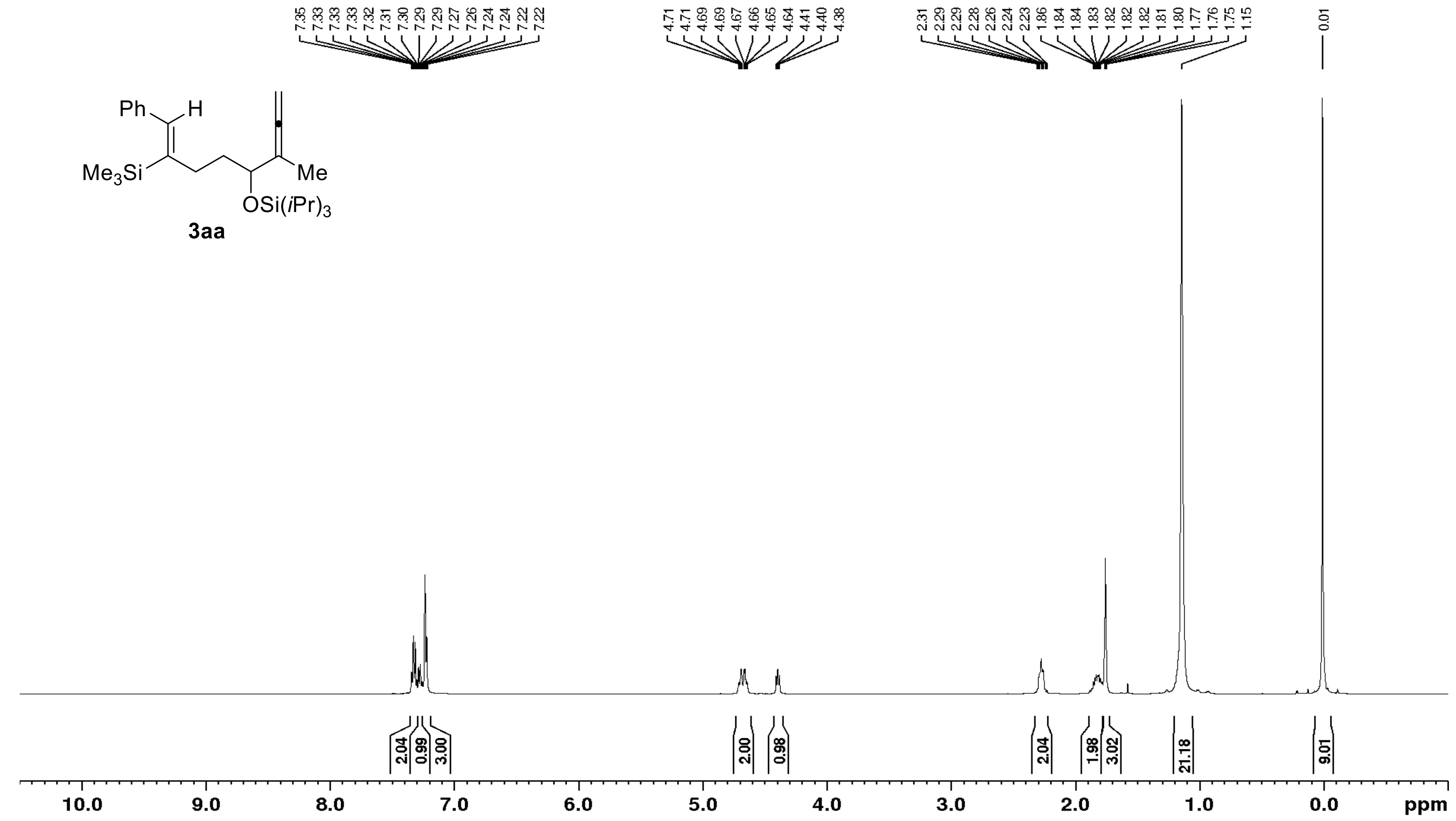

Figure S82. ^{1}H NMR spectrum (500 MHz, $CDCl_3$, 298 K) of **3aa** obtained from the reaction of silyl ether **1a** and propargylsilane **4a**

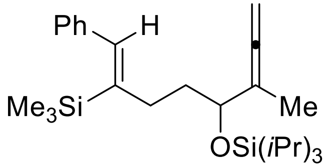

3aa

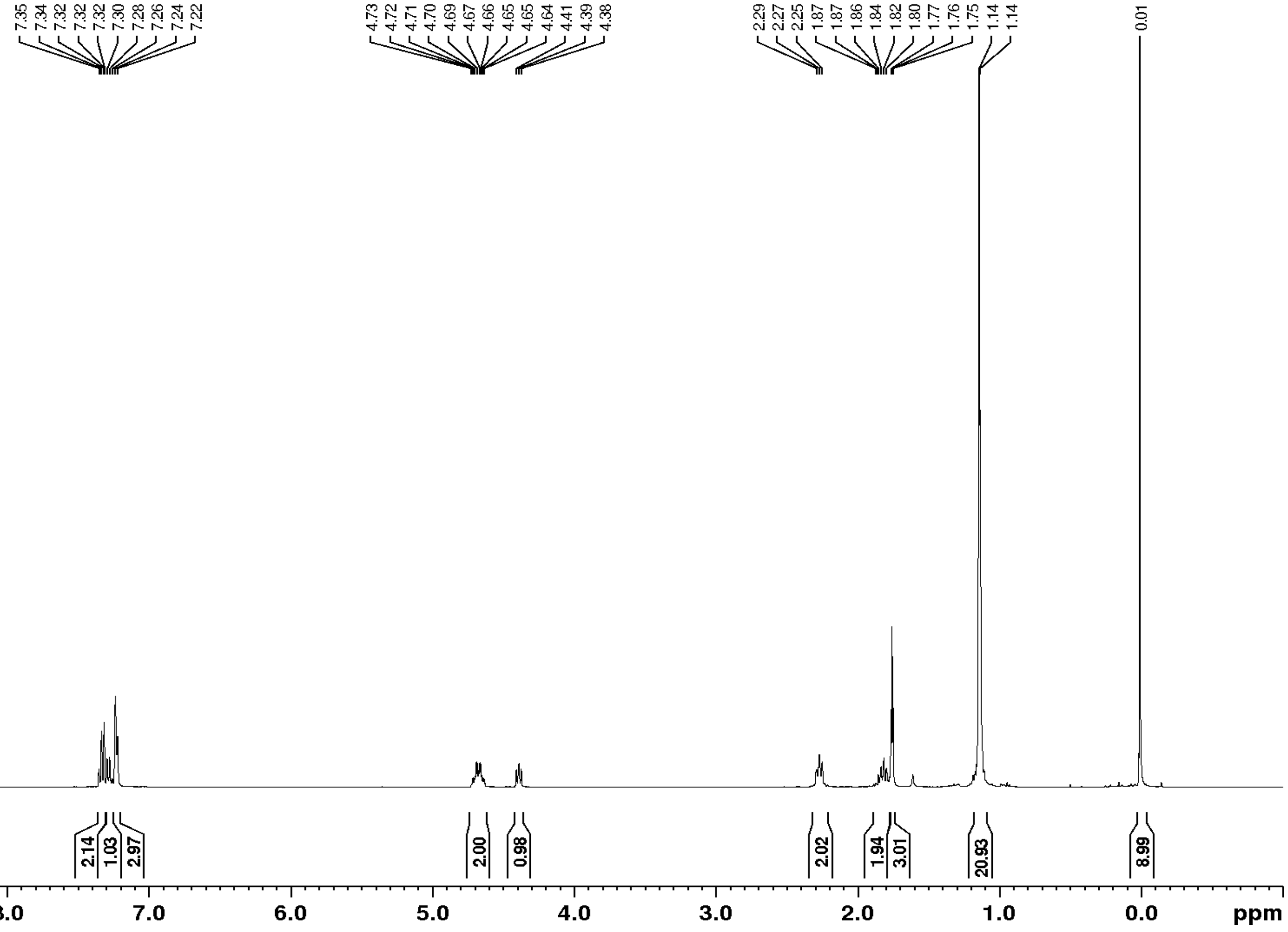

Figure S83. $^{13}C\{^{1}H\}$ NMR spectrum (101 MHz, $CDCl_3$, 298 K) of **3aa**

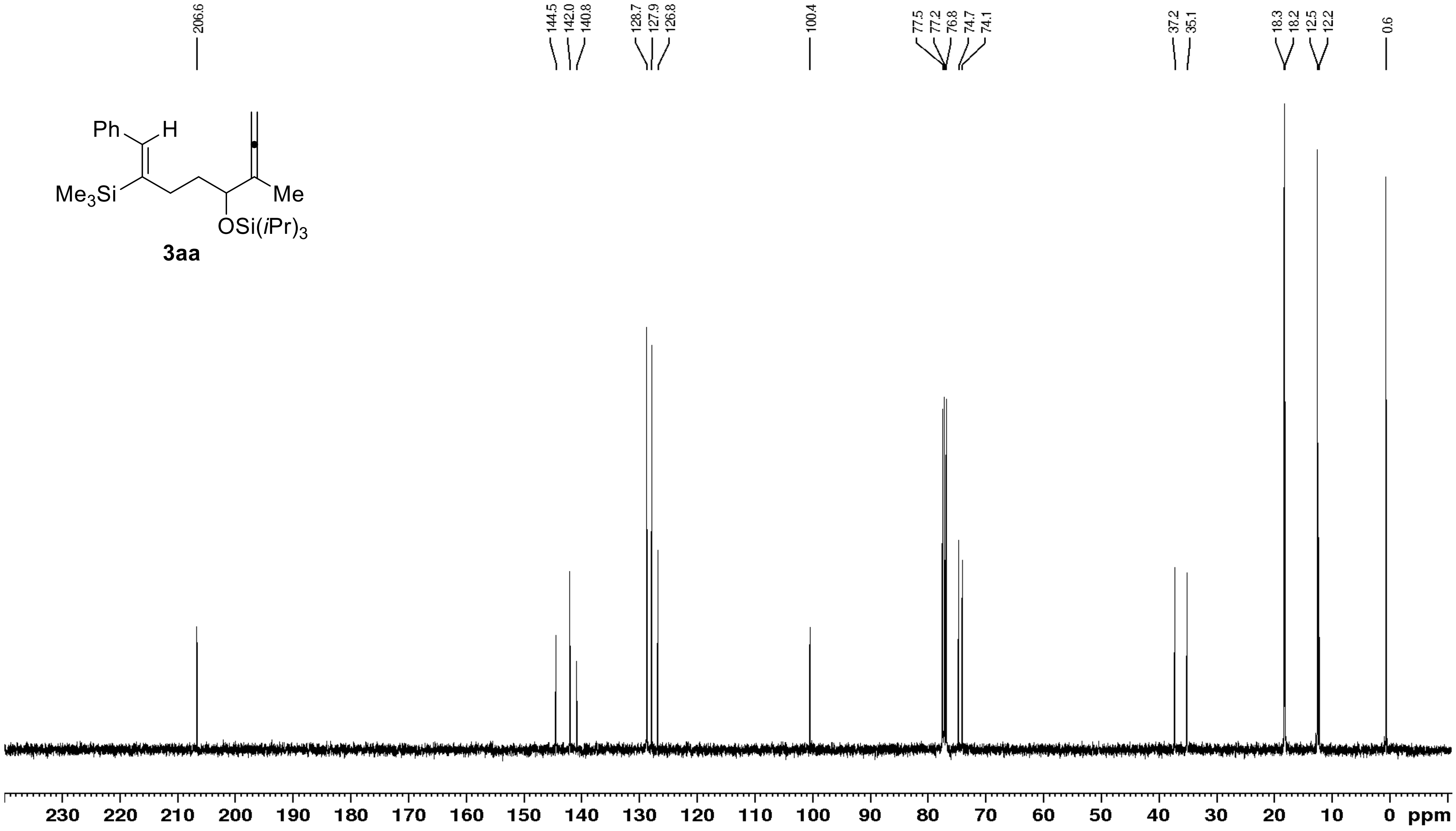

Figure S84. ^{29}Si DEPT NMR spectrum (79 MHz, $CDCl_3$, 298 K, optimized for J = 15.0 Hz) of **3aa**

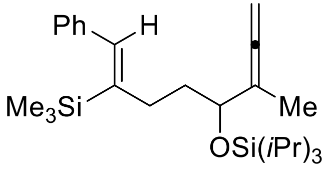

3aa

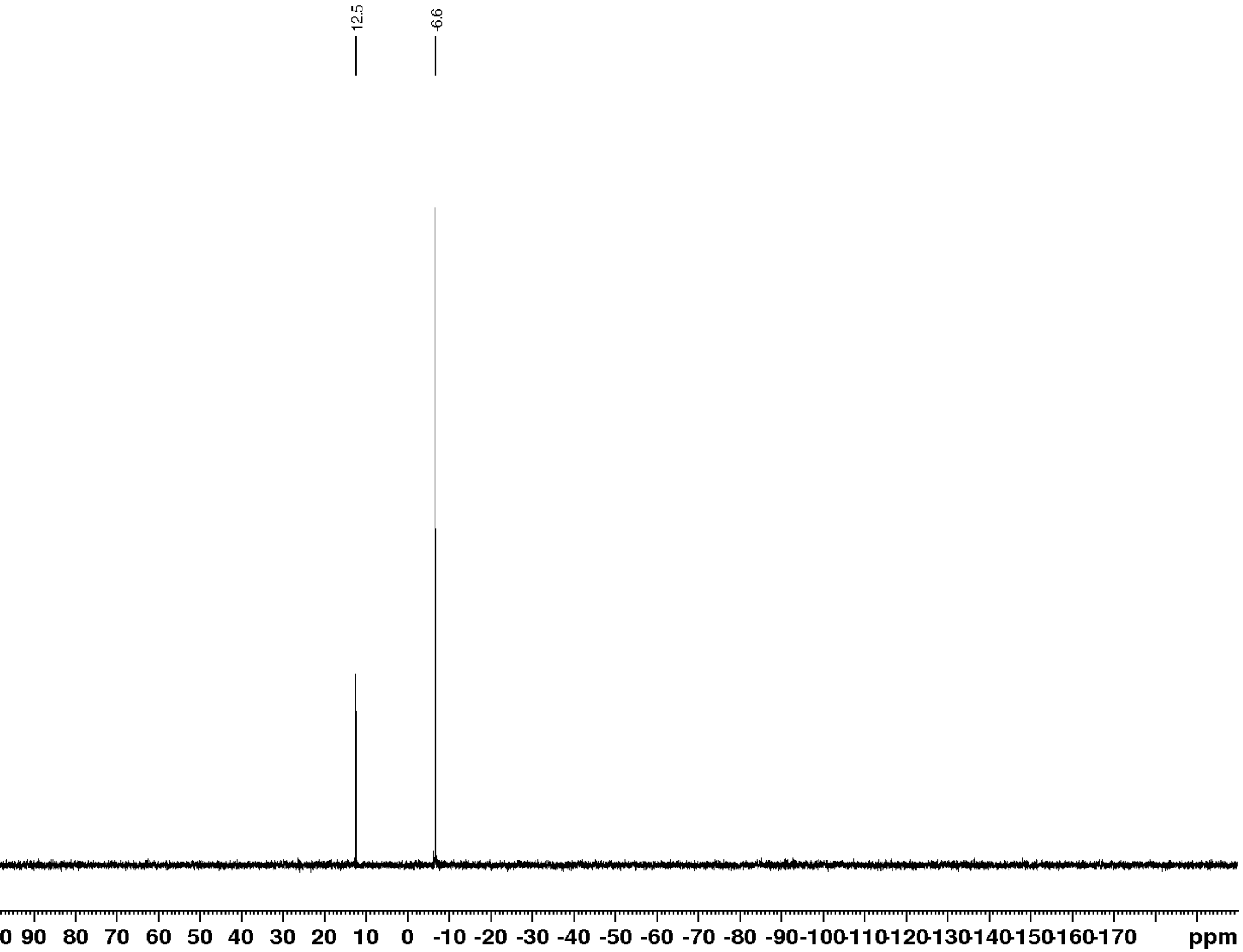

Figure S85. ^{1}H NMR spectrum (500 MHz, $CDCl_3$, 298 K) of **3ba**

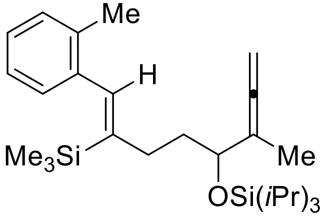

3ba

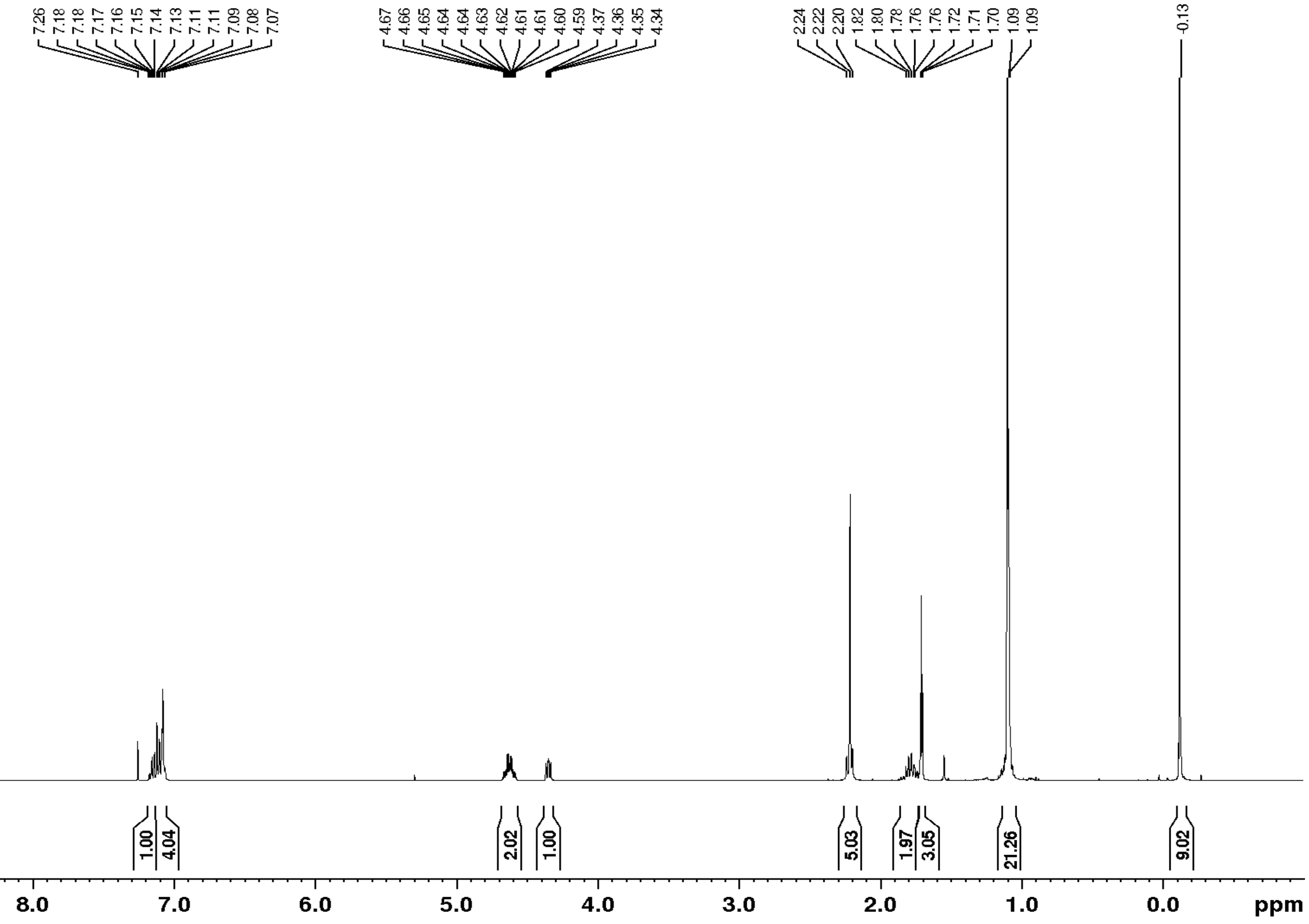

Figure S86. $^{13}C\{^1H\}$ NMR spectrum (126 MHz, $CDCl_3$, 298 K) of **3ba**

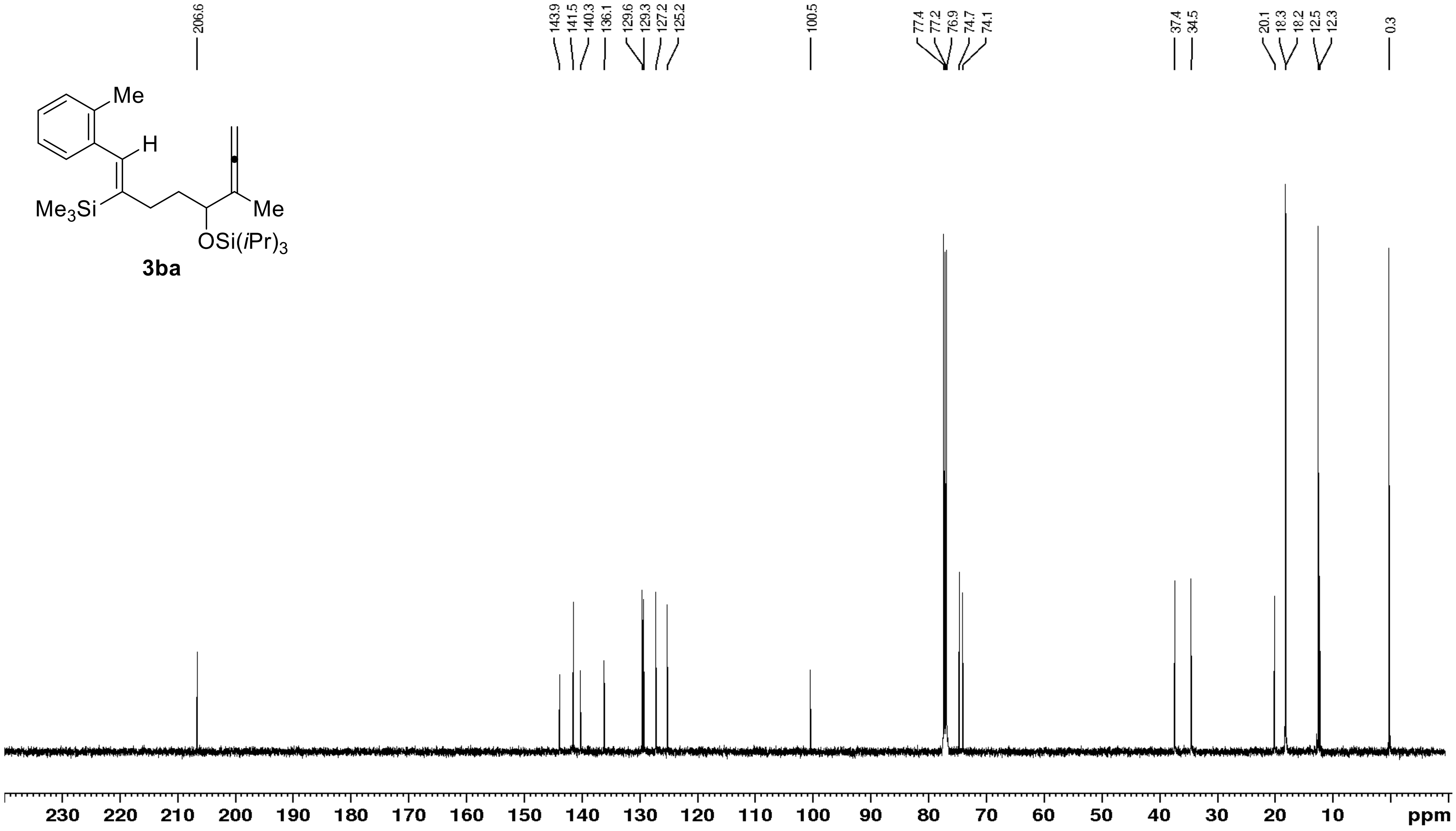

Figure S87. ^{29}Si DEPT NMR spectrum (99 MHz, $CDCl_3$, 298 K, optimized for J = 15.0 Hz) of **3ba**

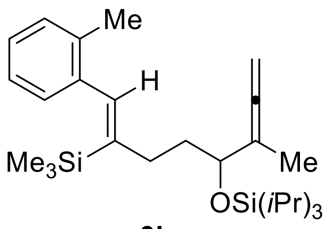

3ba

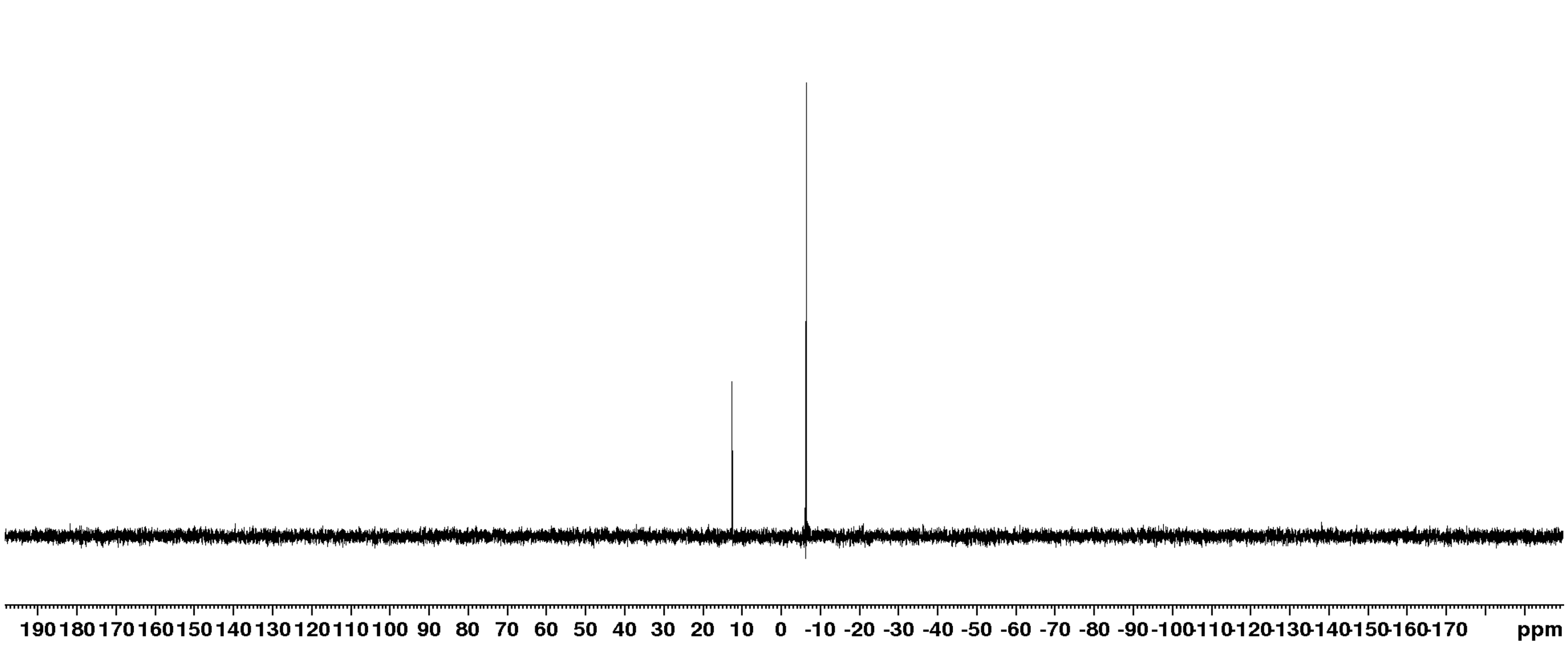

Figure S88. ^{1}H NMR spectrum (400 MHz, $CDCl_3$, 298 K) of **3ca**

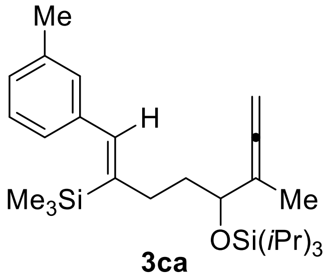

3ca

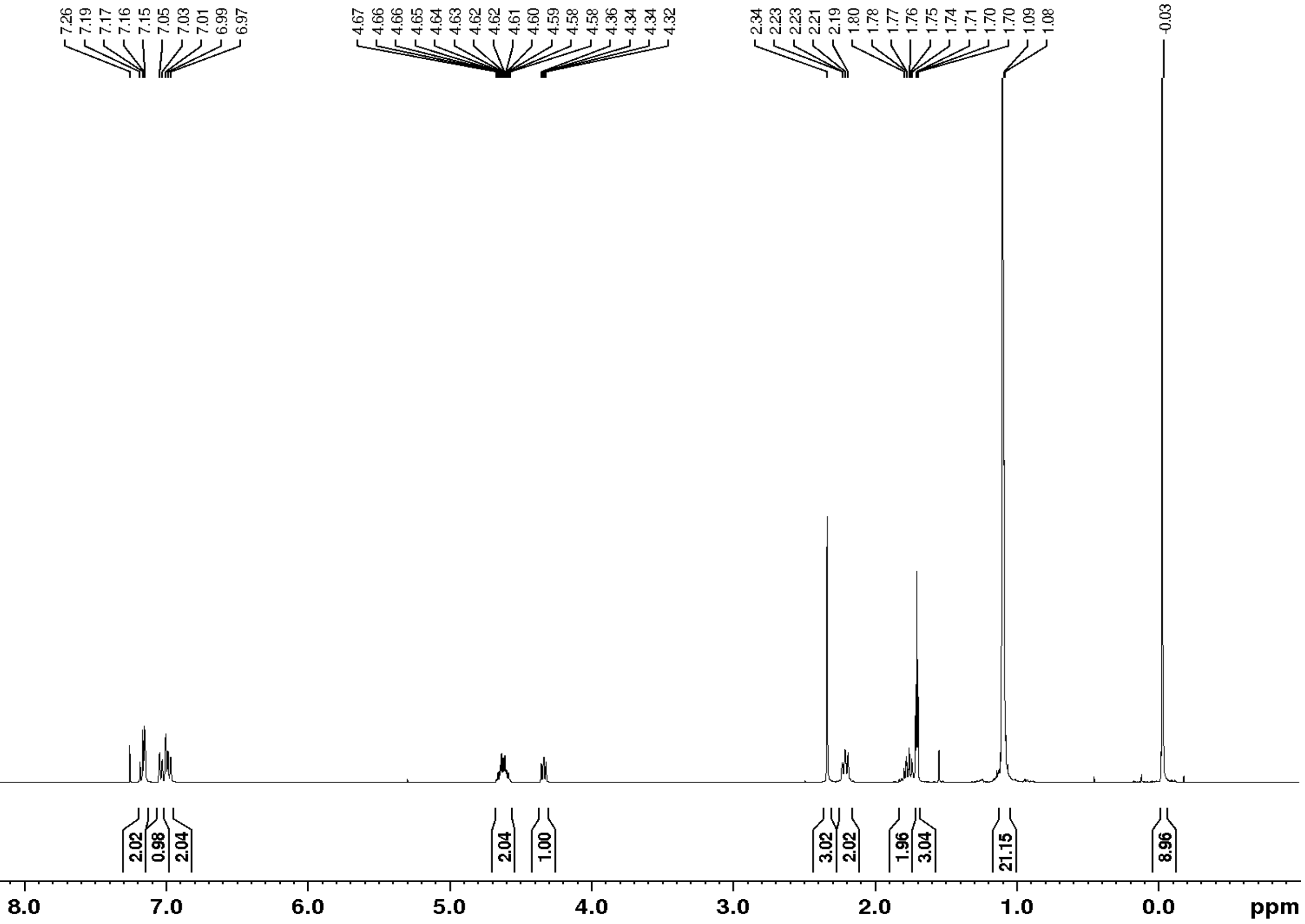

Figure S89. $^{13}C\{^{1}H\}$ NMR spectrum (101 MHz, $CDCl_3$, 298 K) of **3ca**

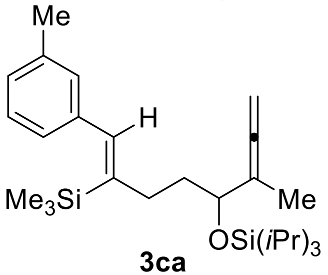

3ca

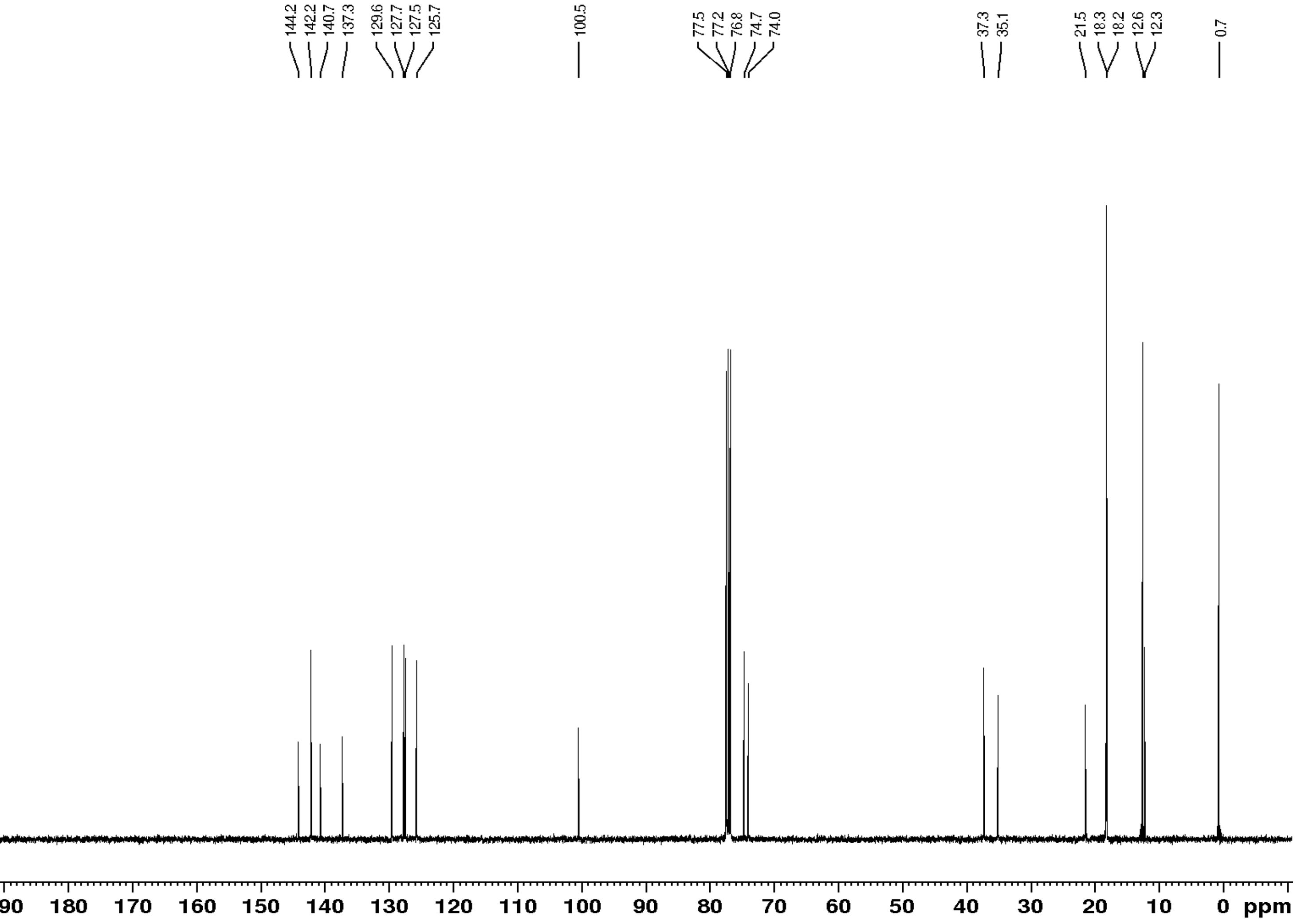

Figure S90. ^{29}Si DEPT NMR spectrum (79 MHz, $CDCl_3$, 298 K, optimized for J = 15.0 Hz) of **3ca**

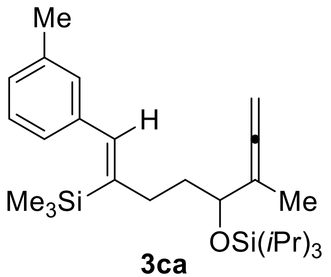

3ca

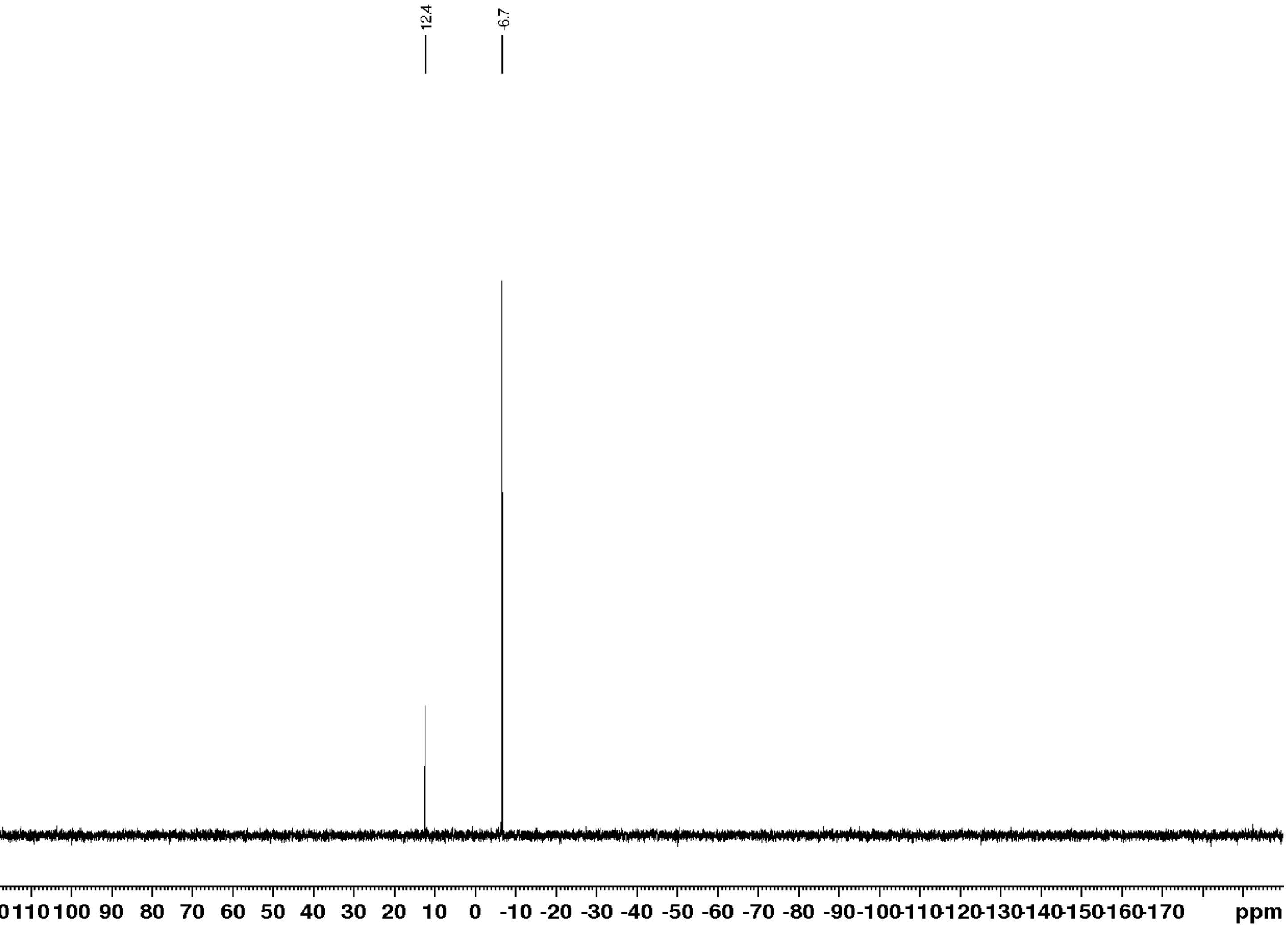

Figure S91. ^{1}H NMR spectrum (400 MHz, $CDCl_3$, 298 K) of **3da**

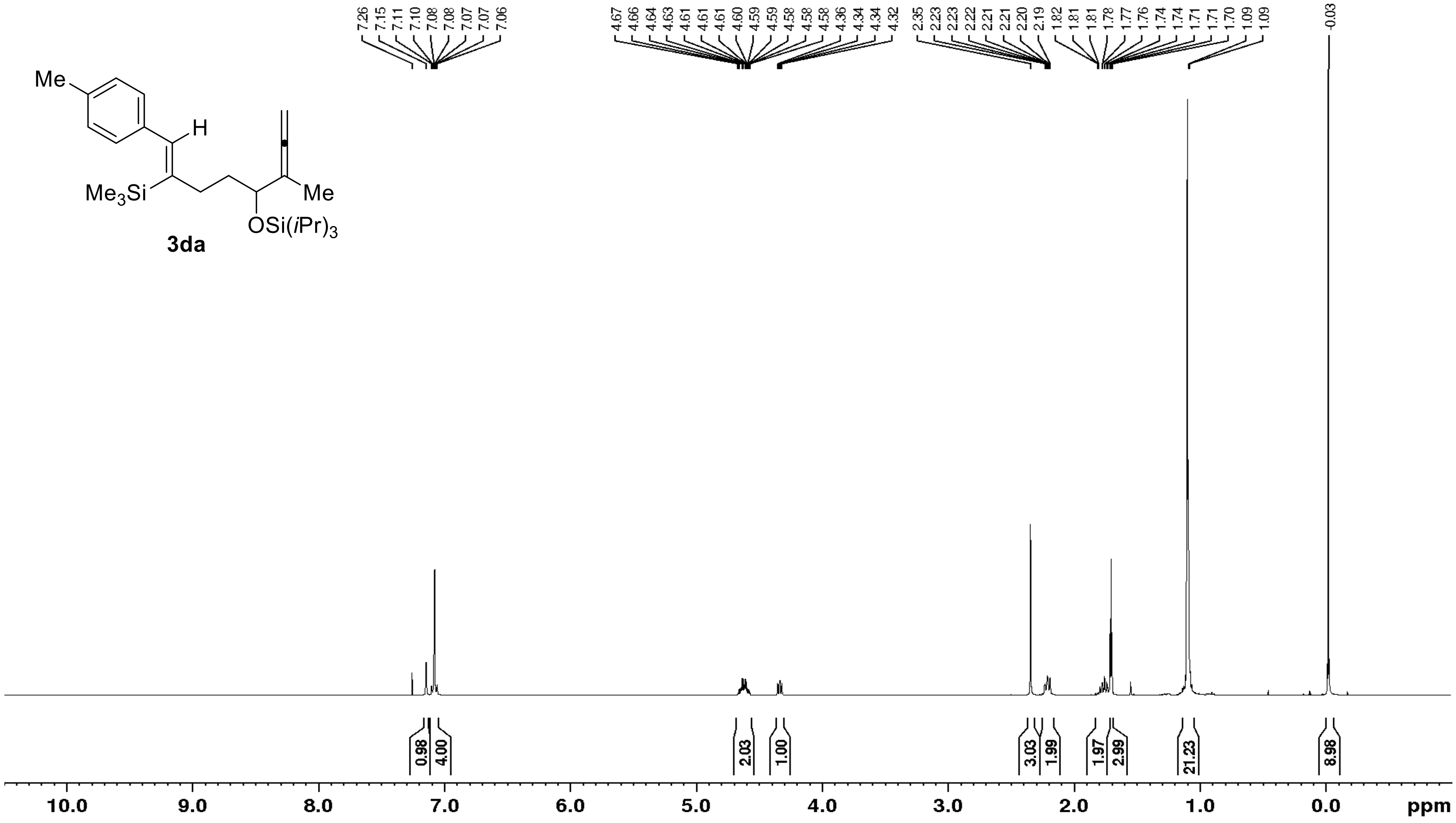

Figure S92. $^{13}C\{^1H\}$ NMR spectrum (101 MHz, $CDCl_3$, 298 K) of **3da**

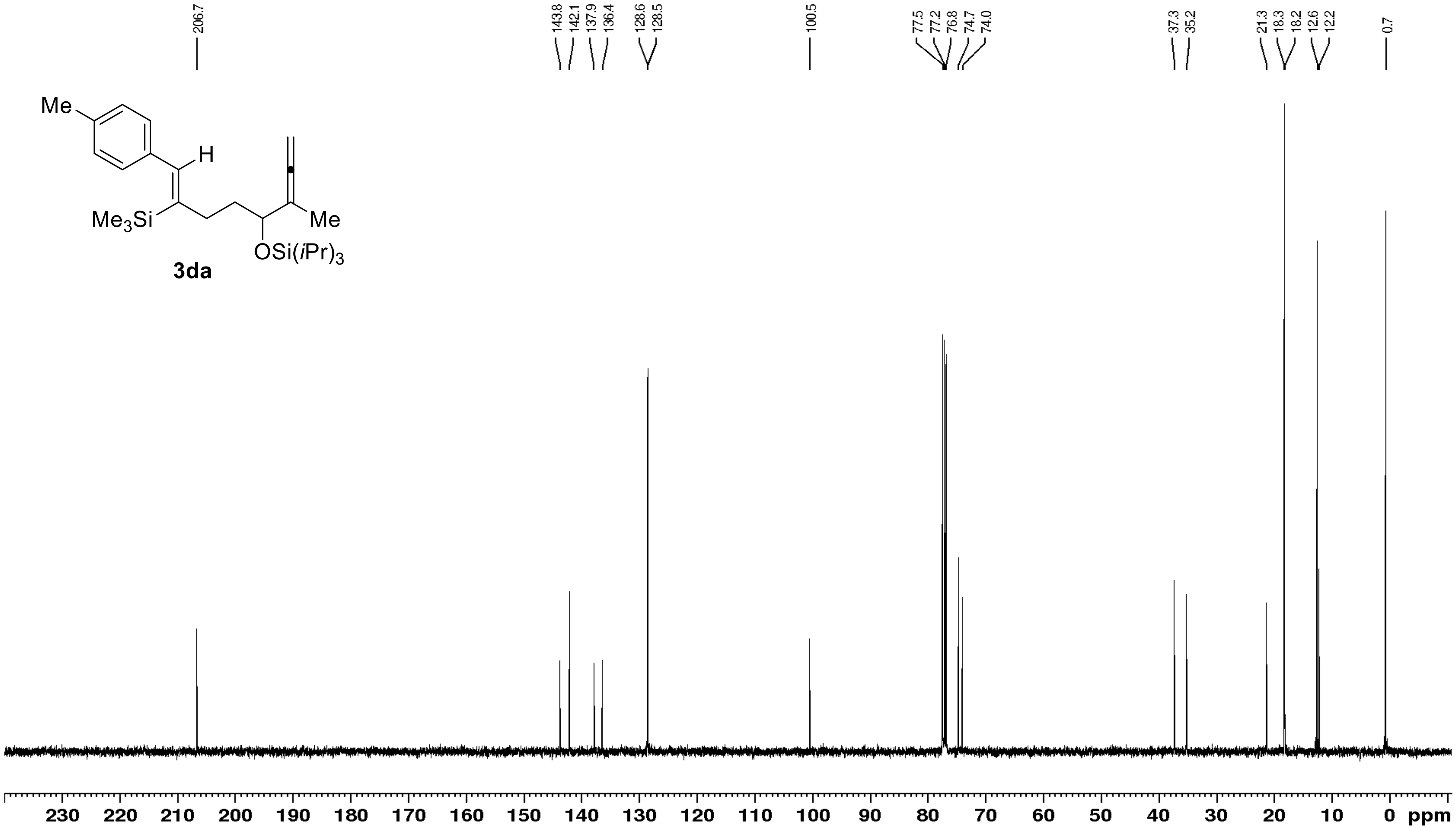

Figure S93. ^{29}Si DEPT NMR spectrum (79 MHz, $CDCl_3$, 298 K, optimized for J = 15.0 Hz) of **3da**

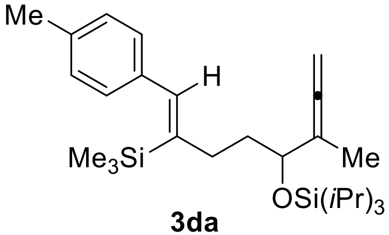

3da

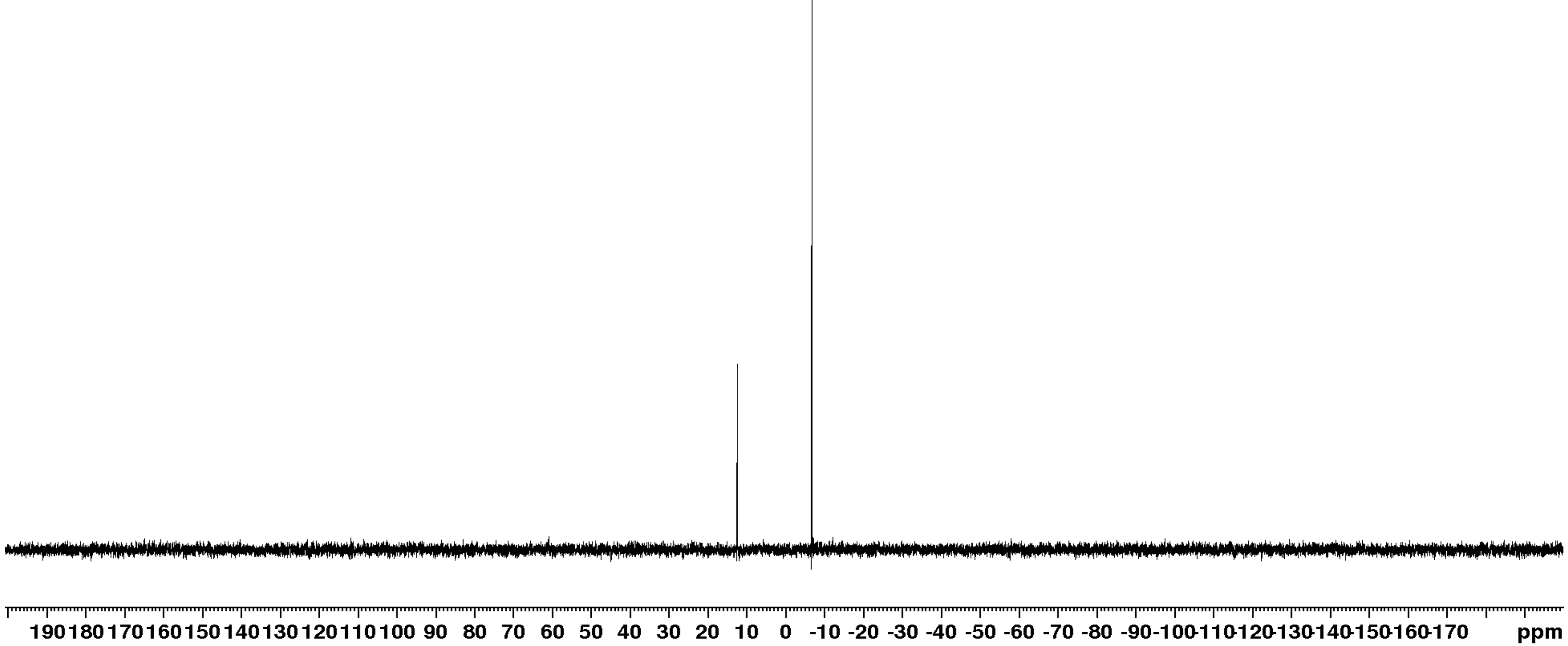

Figure S94. ^{1}H NMR spectrum (400 MHz, $CDCl_3$, 298 K) of **3ea**

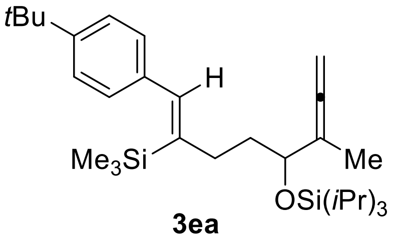

3ea

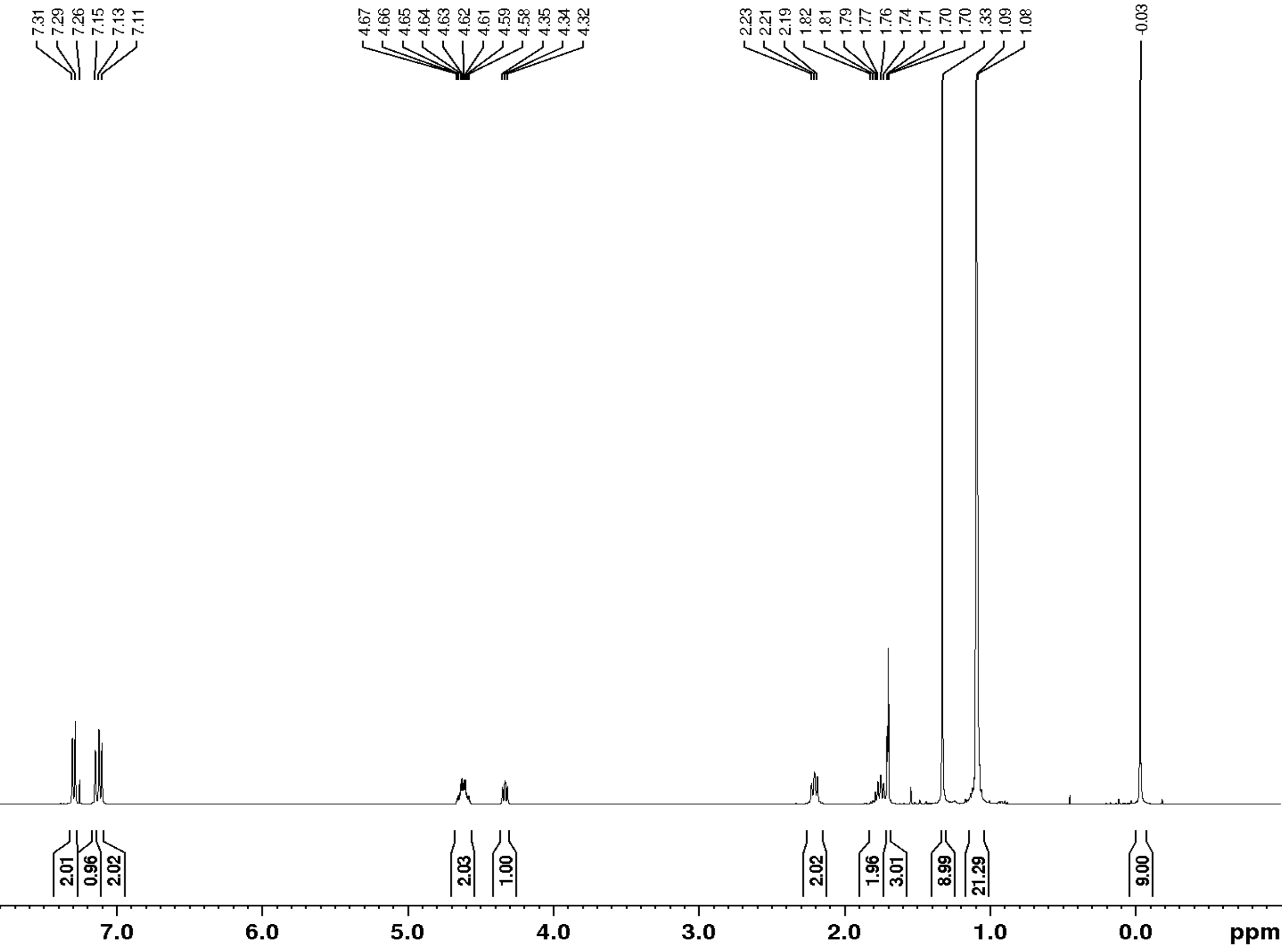

Figure S95. $^{13}C\{^1H\}$ NMR spectrum (101 MHz, $CDCl_3$, 298 K) of **3ea**

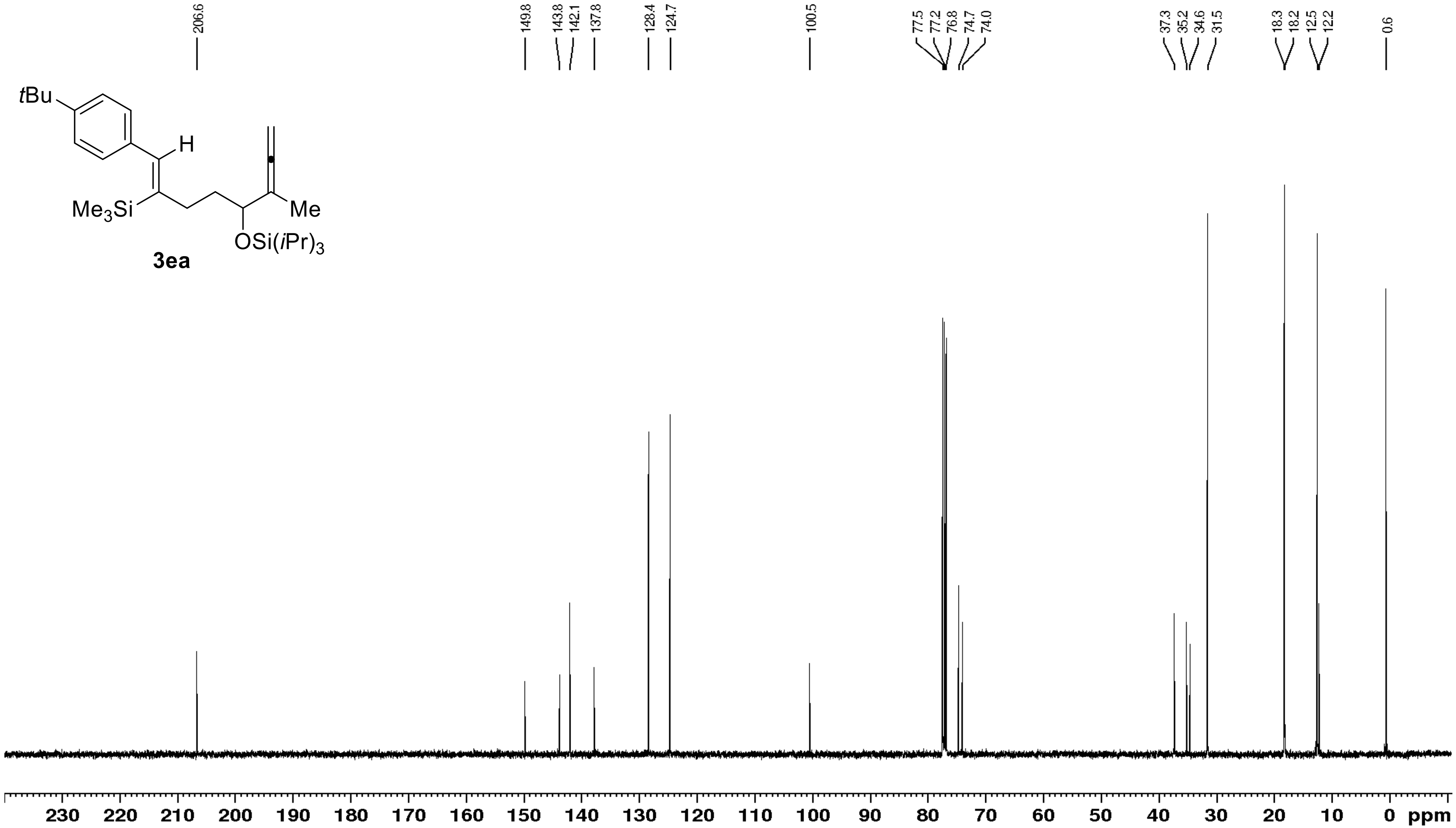

Figure S96. ^{29}Si DEPT NMR spectrum (79 MHz, $CDCl_3$, 298 K, optimized for J = 15.0 Hz) of **3ea**

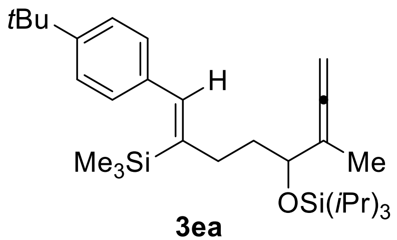

3ea

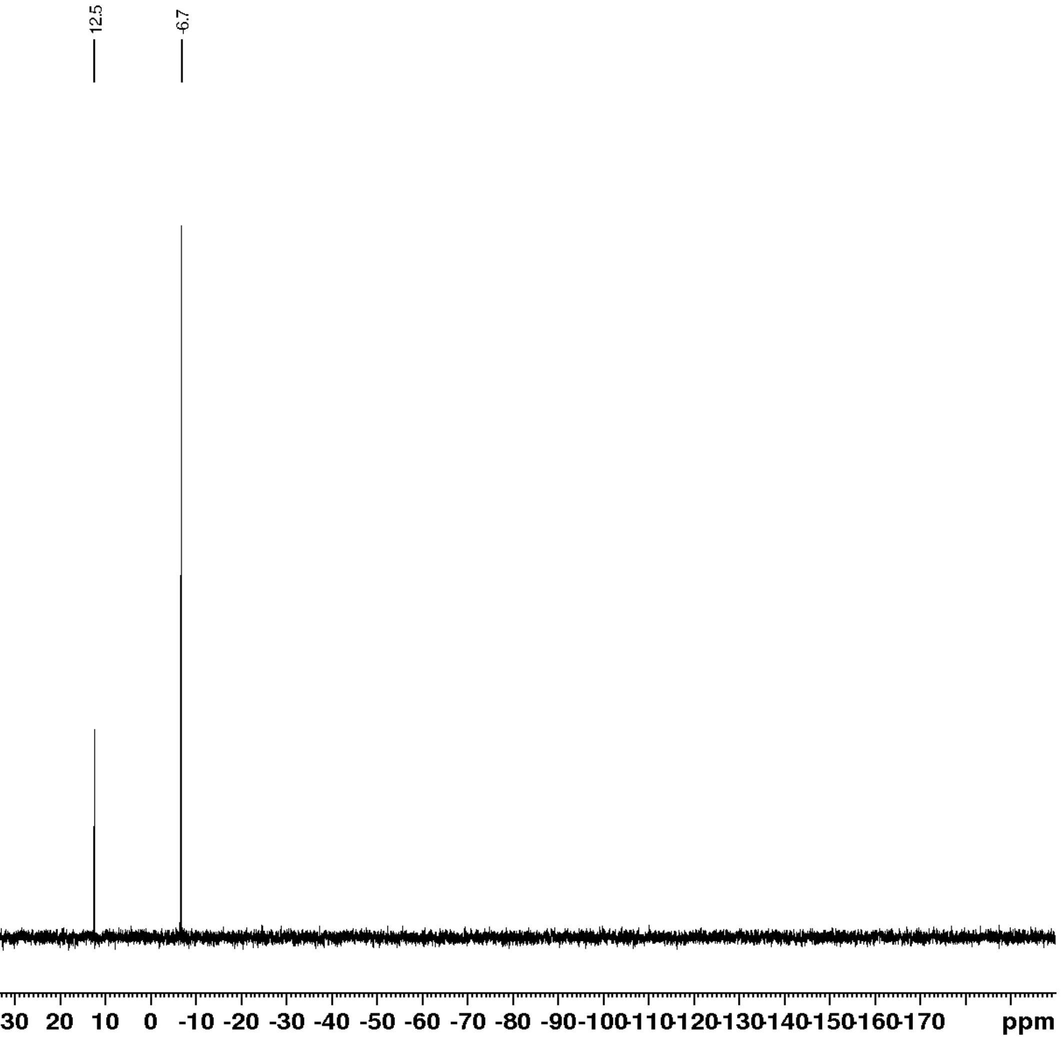

Figure S97. ^{1}H NMR spectrum (400 MHz, $CDCl_3$, 298 K) of **3fa**

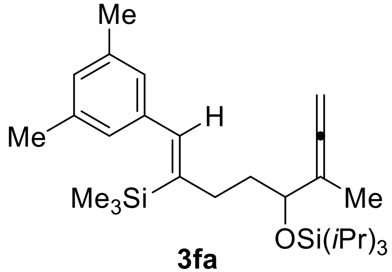

3fa

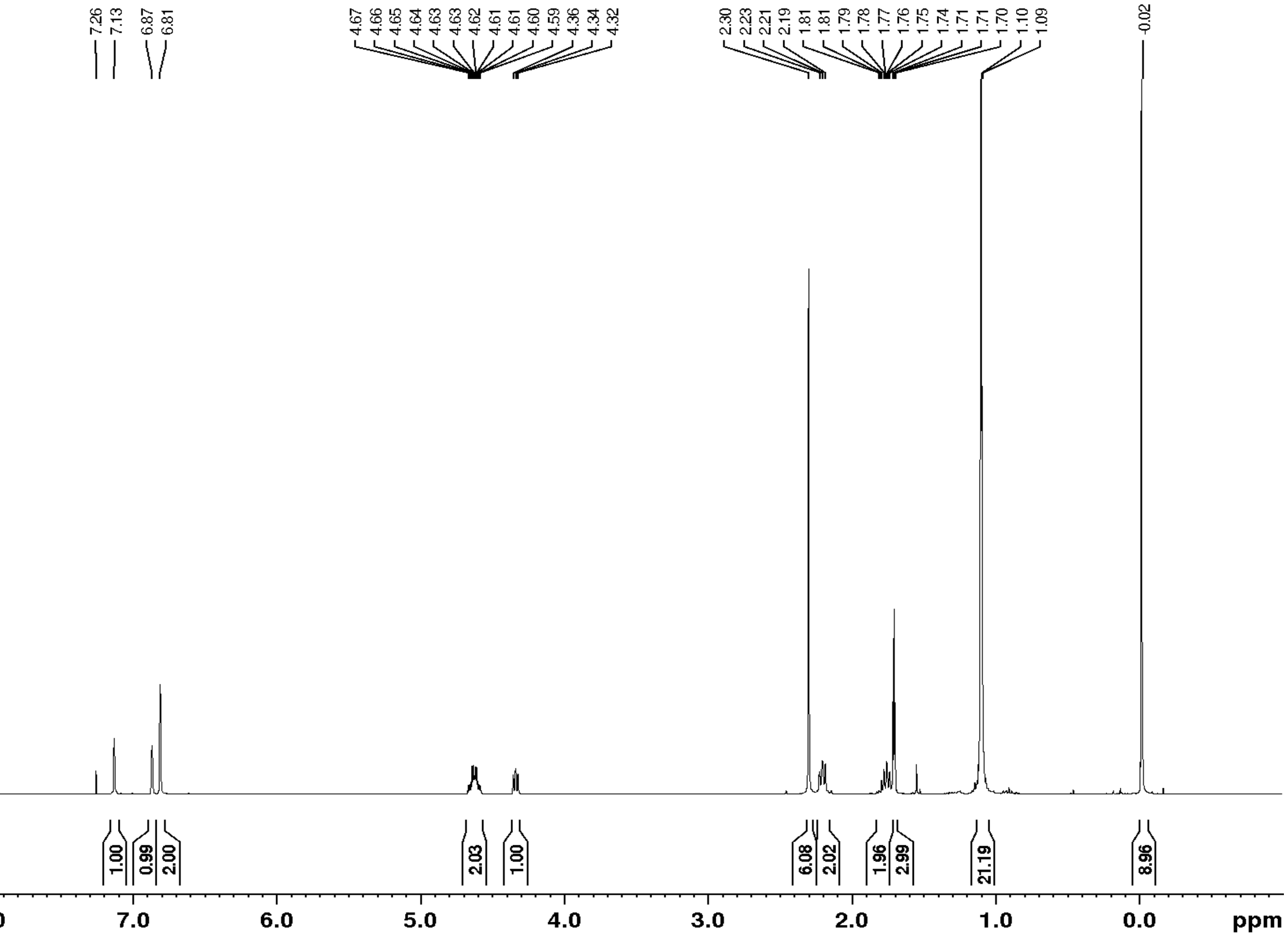

Figure S98. $^{13}C\{^{1}H\}$ NMR spectrum (101 MHz, $CDCl_3$, 298 K) of **3fa**

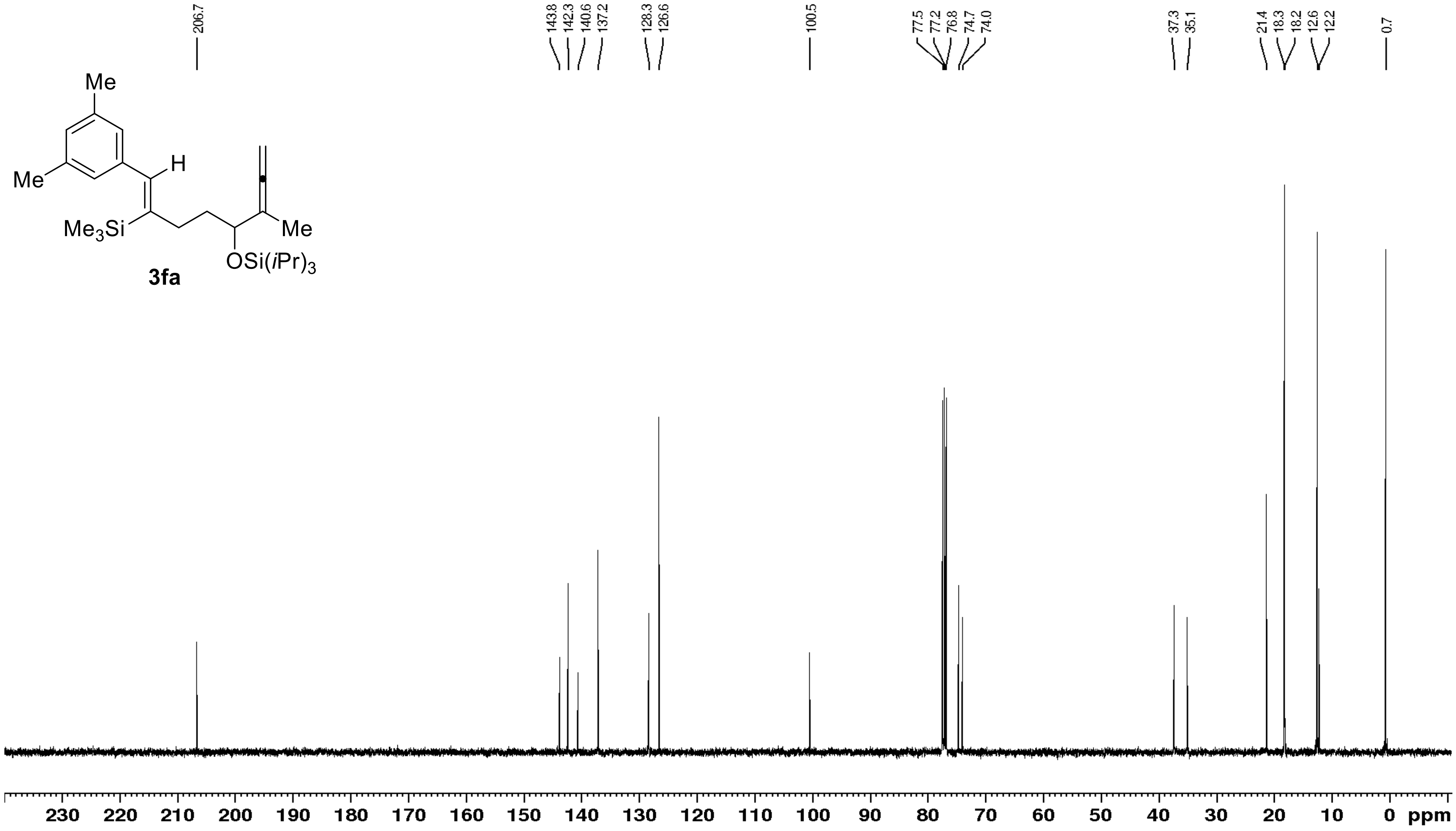

Figure S99. ^{29}Si DEPT NMR spectrum (79 MHz, $CDCl_3$, 298 K, optimized for J = 15.0 Hz) of **3fa**

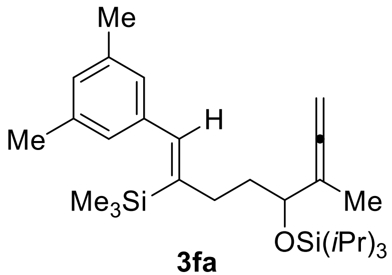

3fa

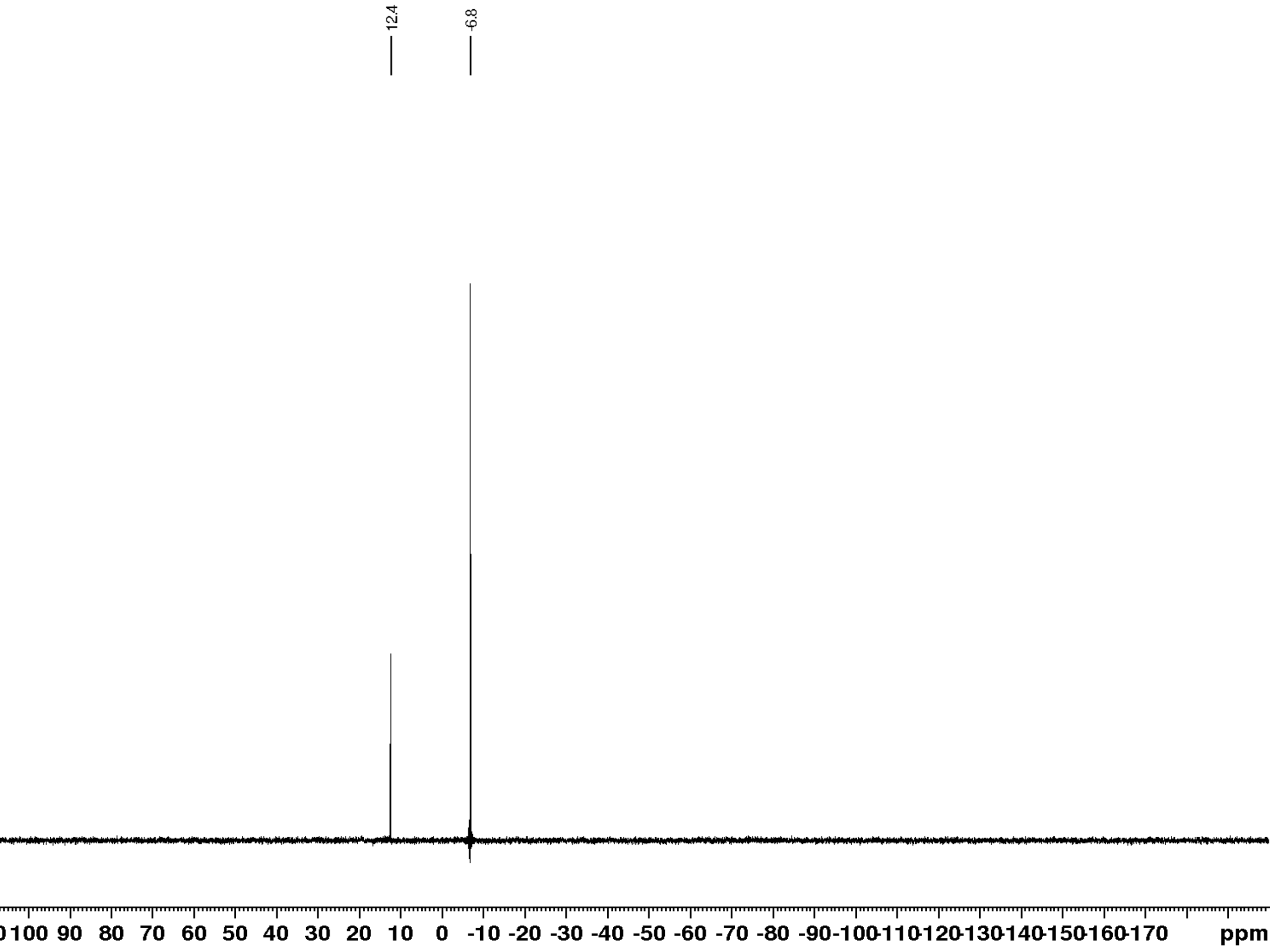

Figure S100. ^{1}H NMR spectrum (400 MHz, $CDCl_3$, 298 K) of **3ga**

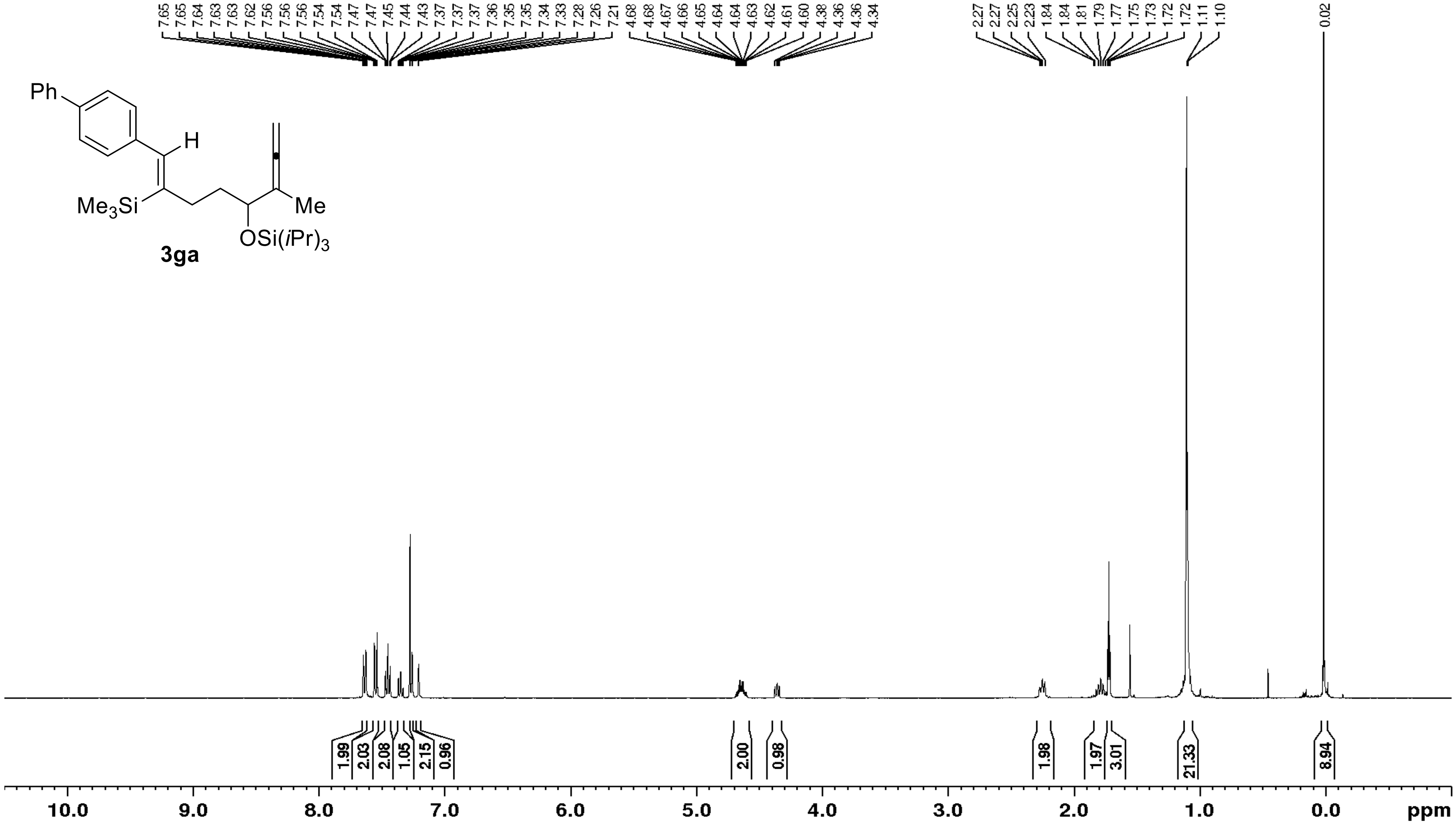

Figure S101. $^{13}C\{^1H\}$ NMR spectrum (101 MHz, $CDCl_3$, 298 K) of **3ga**

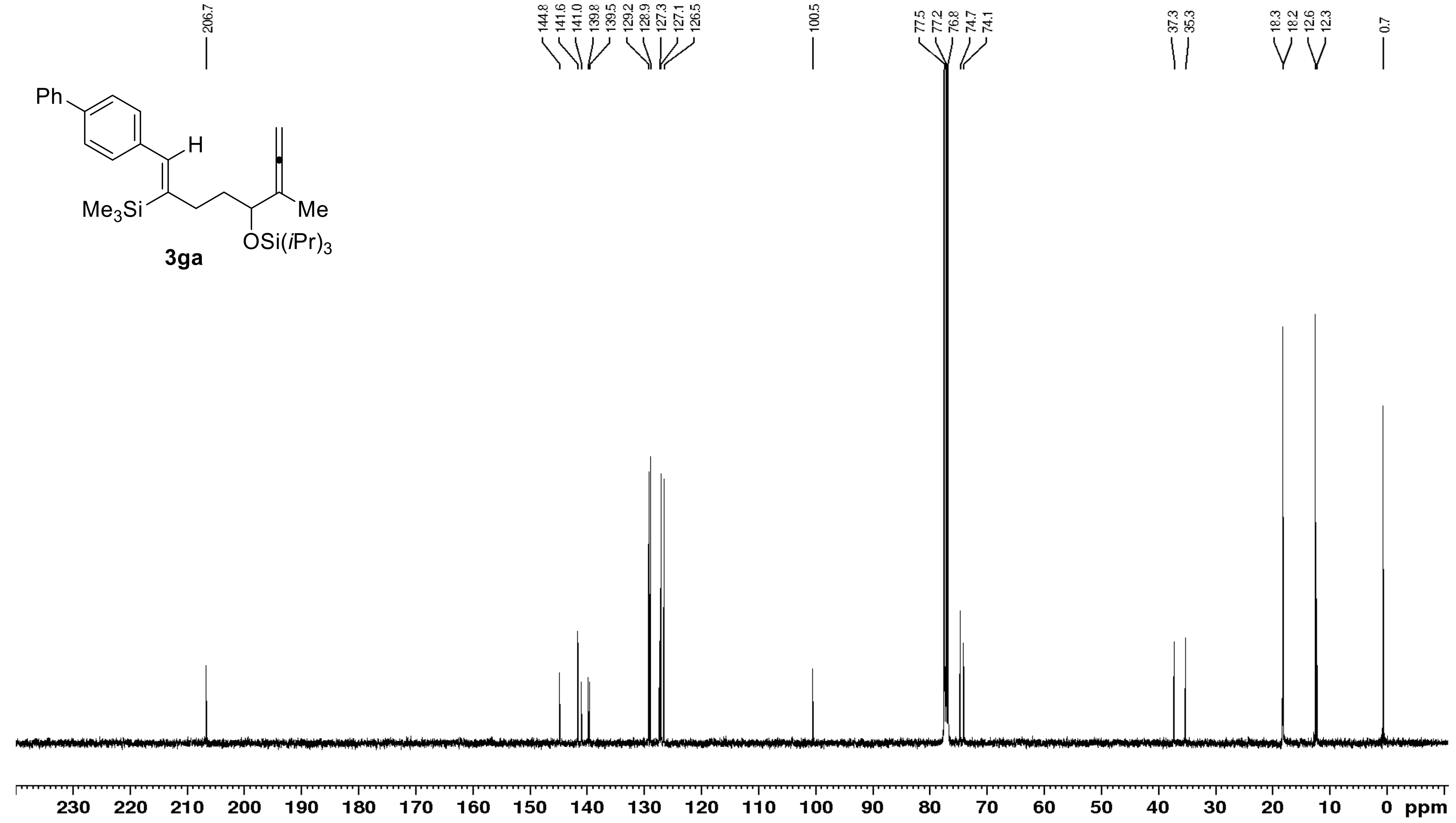

Figure S102. ^{29}Si DEPT NMR spectrum (79 MHz, $CDCl_3$, 298 K, optimized for J = 15.0 Hz) of **3ga**

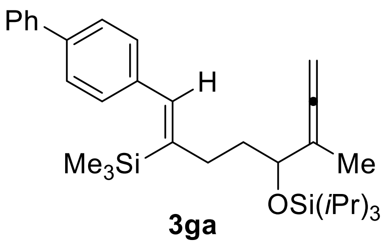

3ga

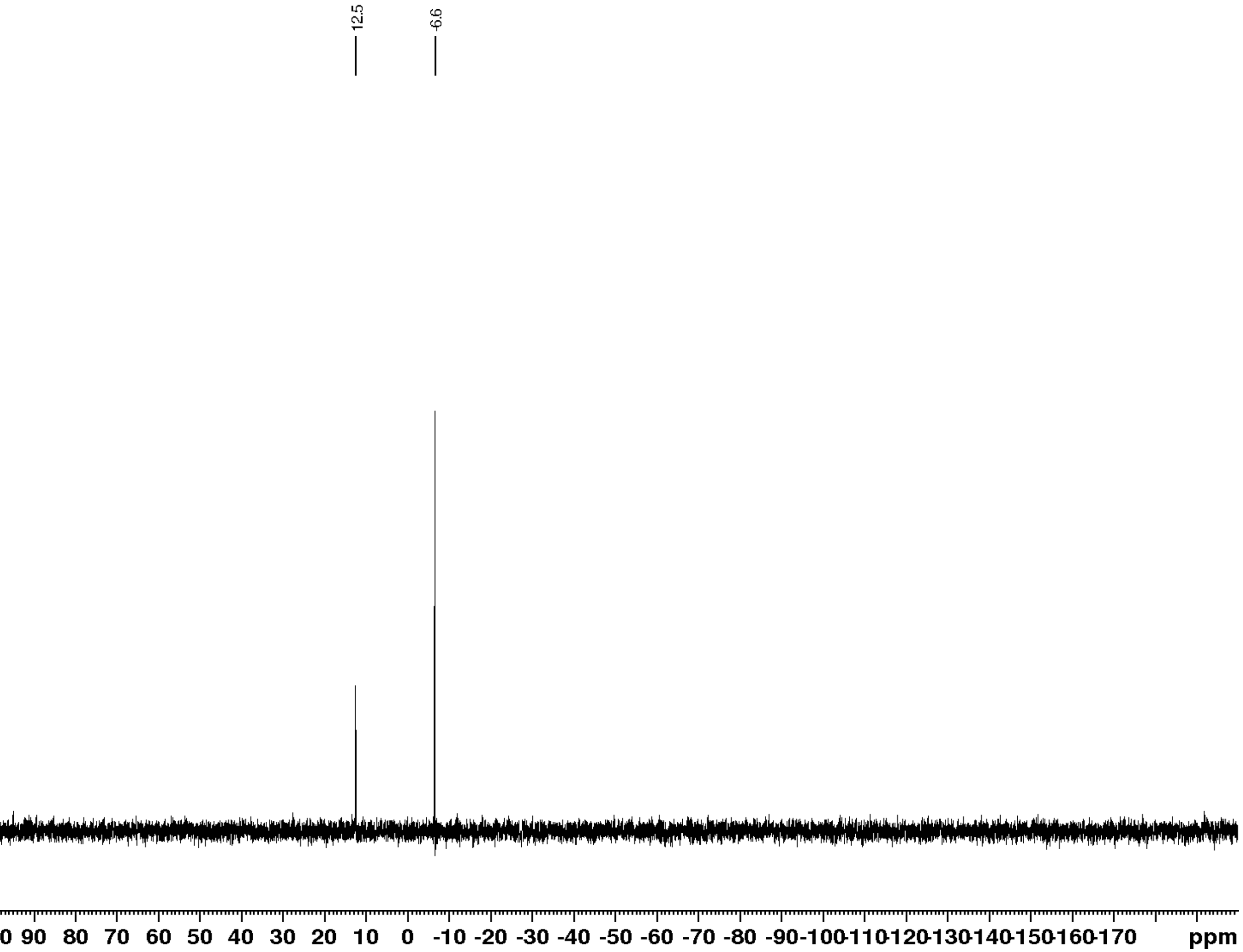

Figure S103. ^{1}H NMR spectrum (500 MHz, $CDCl_3$, 298 K) of **3ha**

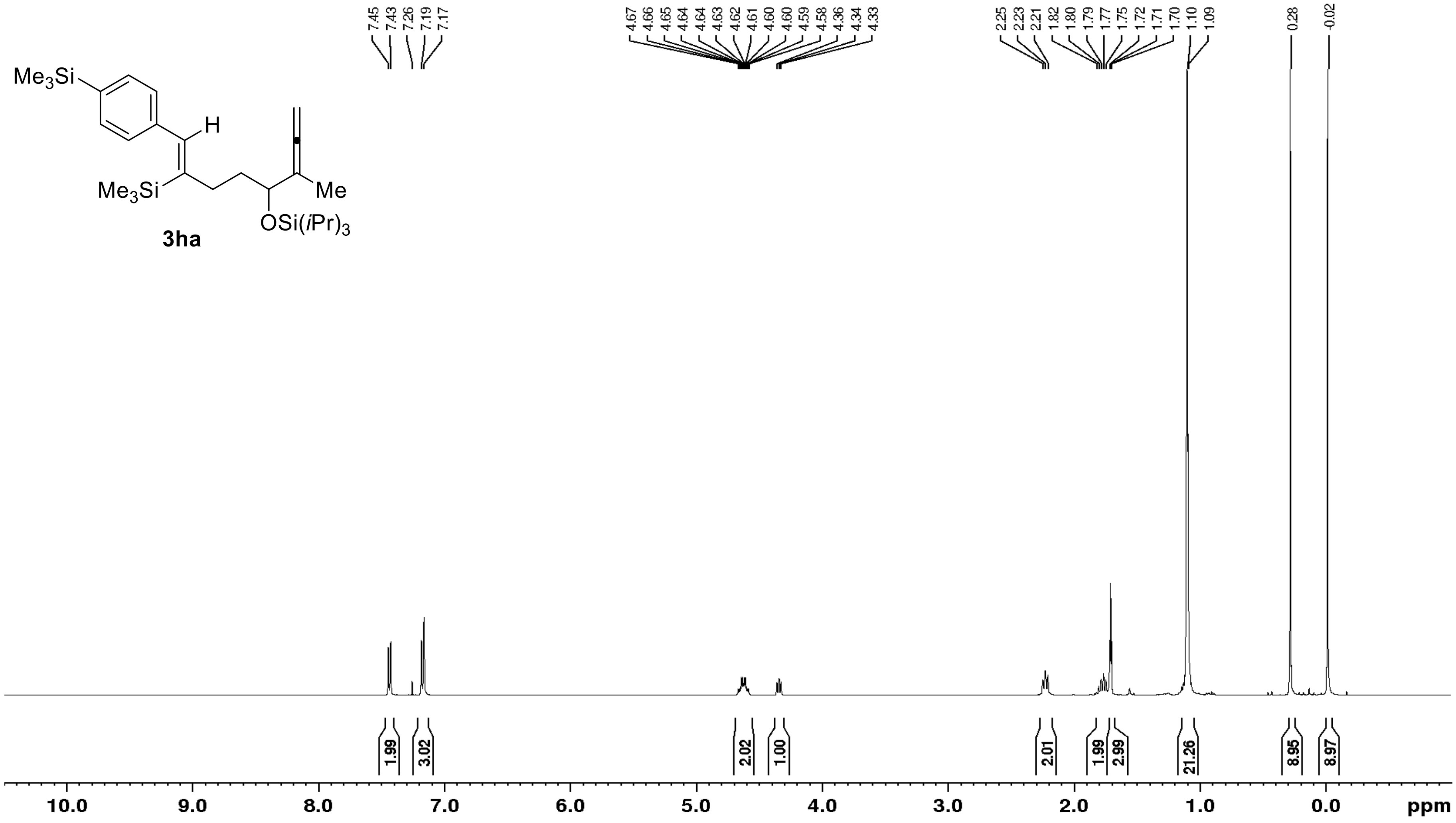

Figure S104. $^{13}C\{^1H\}$ NMR spectrum (126 MHz, $CDCl_3$, 298 K) of **3ha**

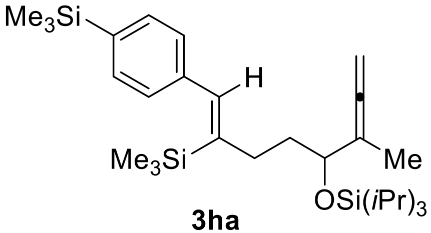

3ha

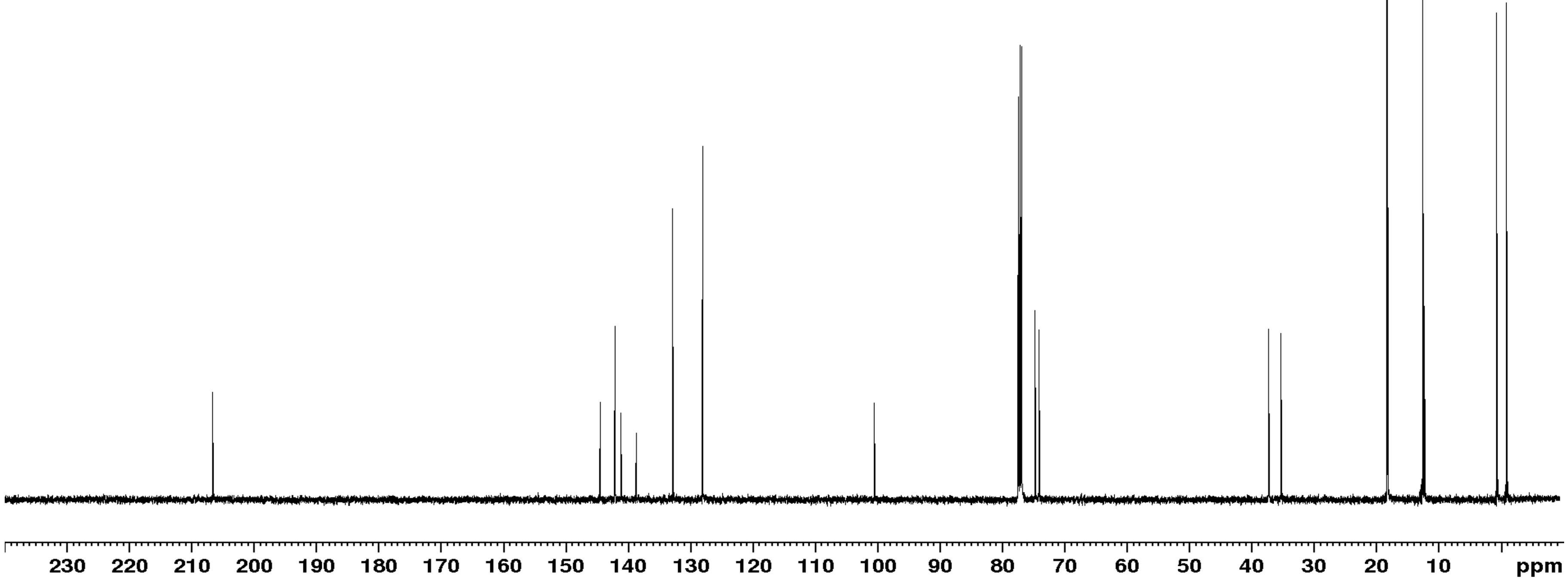

Figure S105. ^{29}Si DEPT NMR spectrum (99 MHz, $CDCl_3$, 298 K, optimized for J = 15.0 Hz) of **3ha**

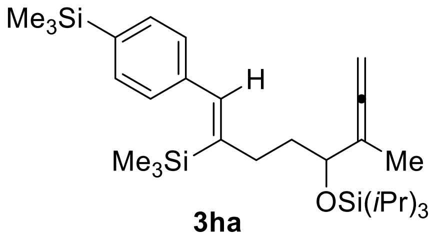

3ha

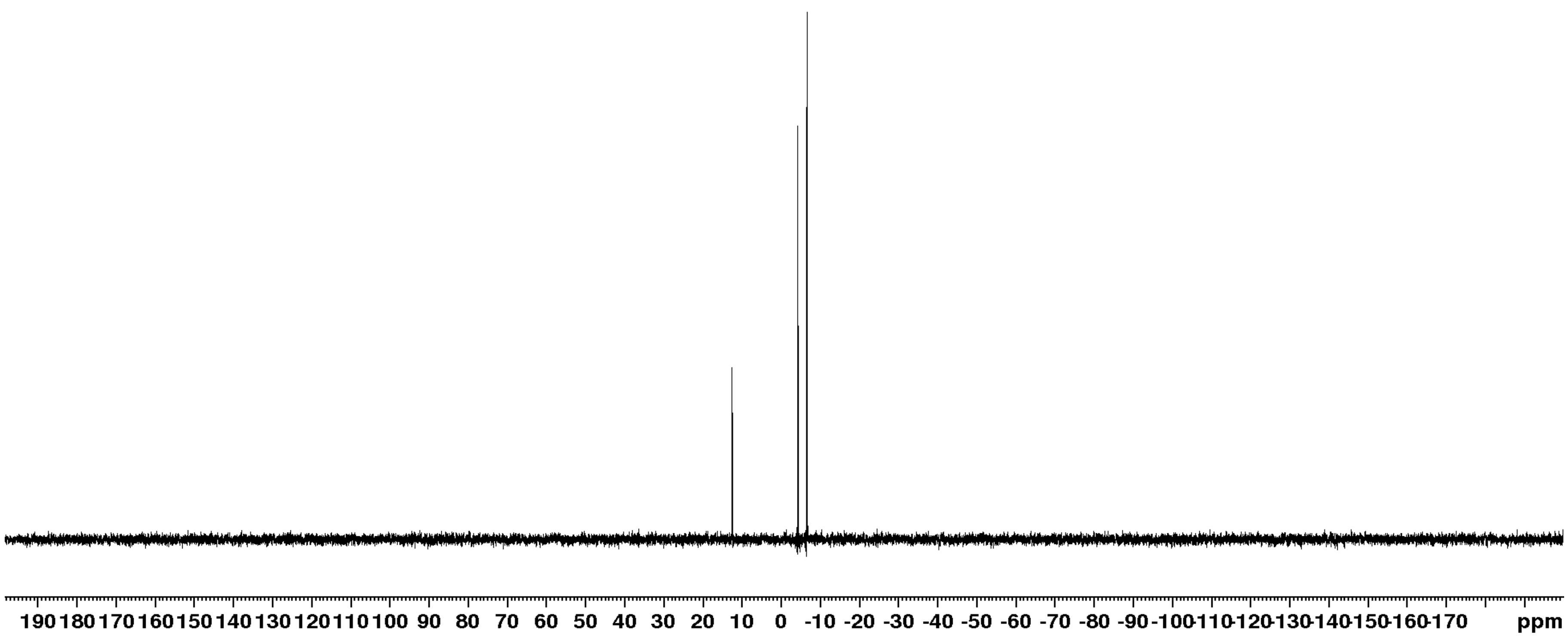

Figure S106. ^{1}H NMR spectrum (400 MHz, $CDCl_3$, 298 K) of **3ia**

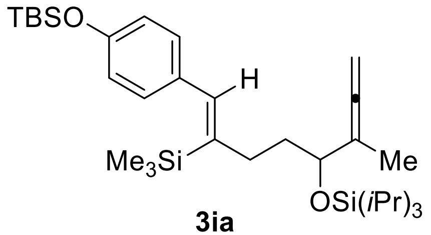

3ia

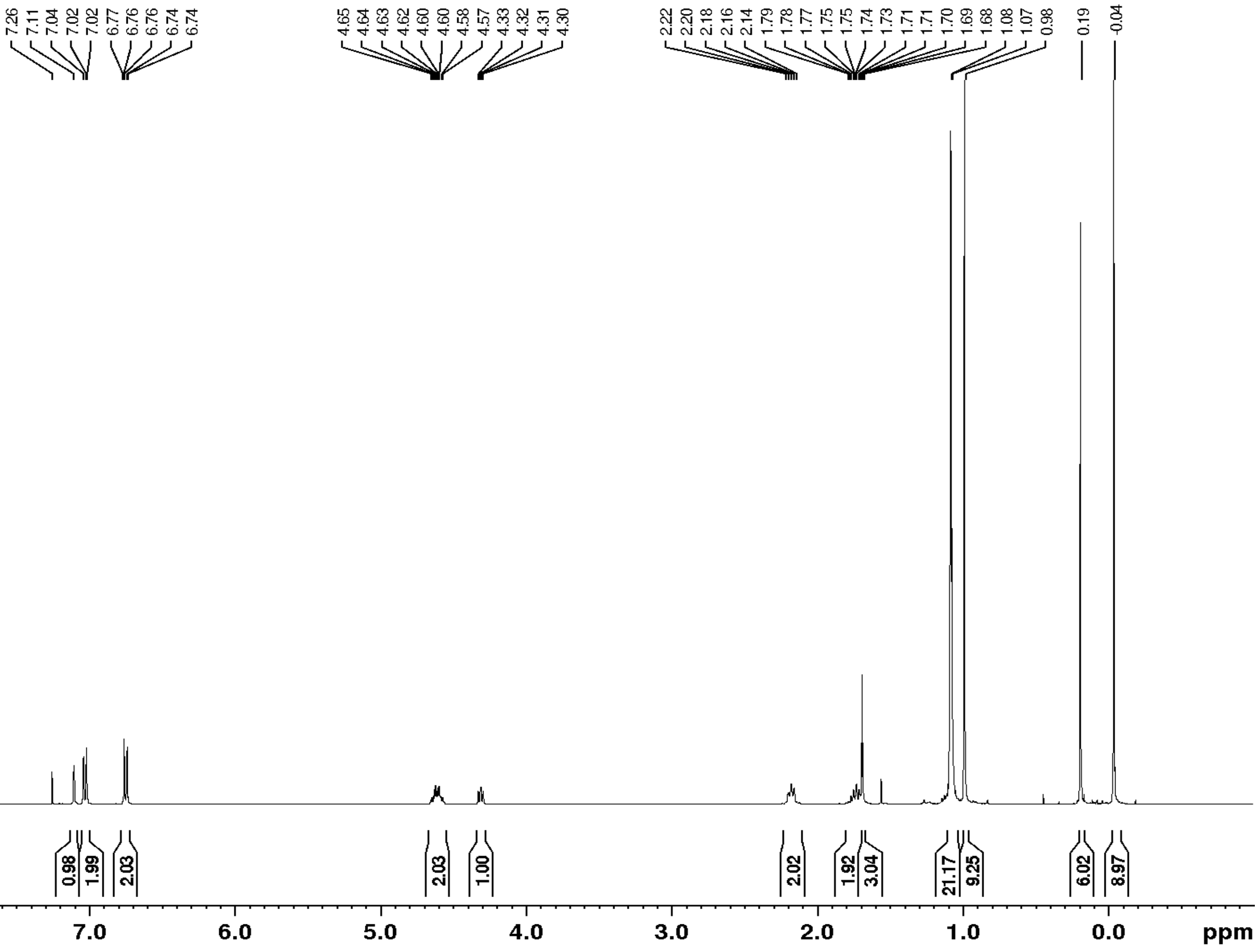

Figure S107. $^{13}C\{^{1}H\}$ NMR spectrum (101 MHz, $CDCl_3$, 298 K) of **3ia**

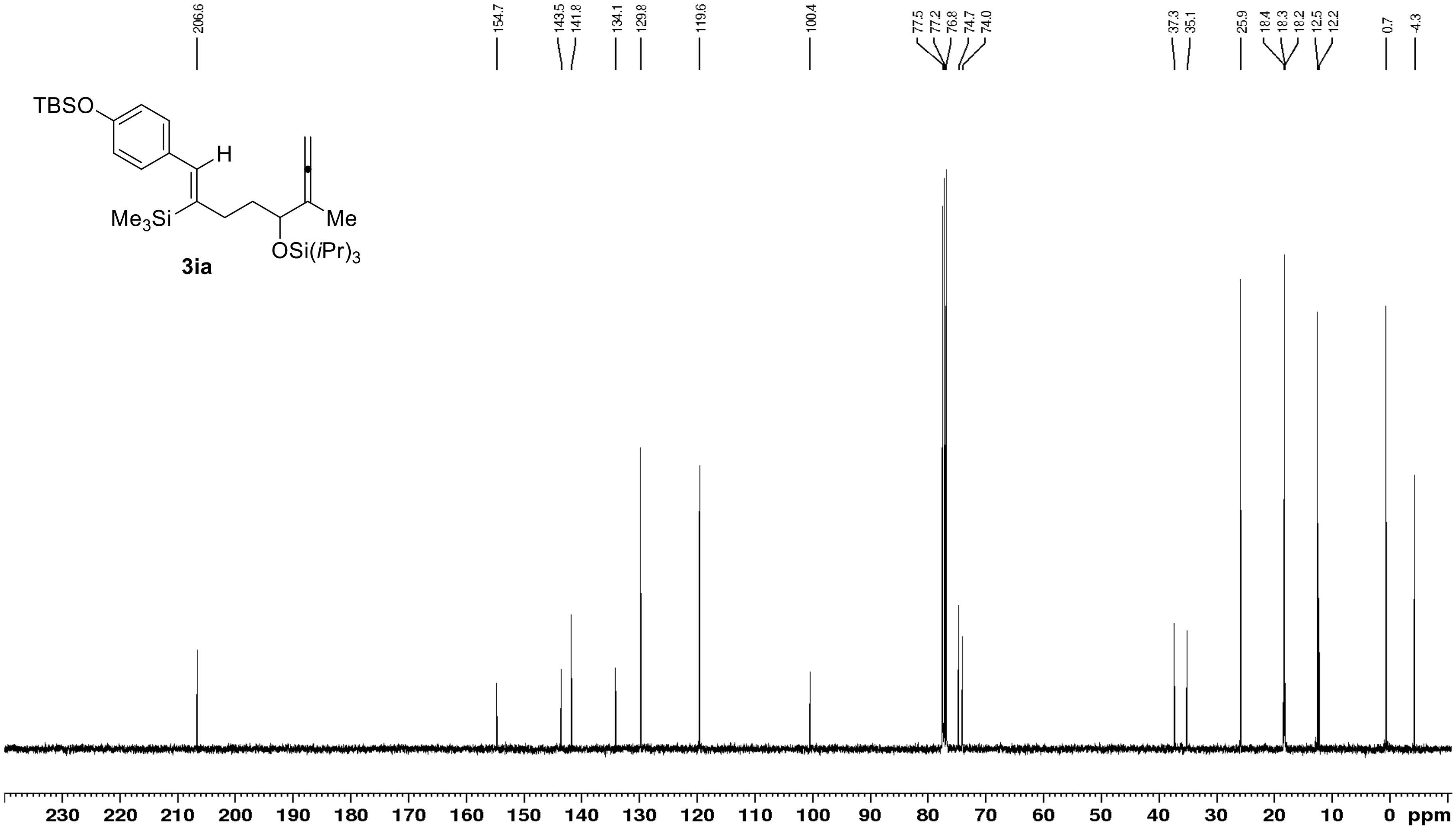

Figure S108. ^{29}Si DEPT NMR spectrum (79 MHz, $CDCl_3$, 298 K, optimized for J = 15.0 Hz) of **3ia**

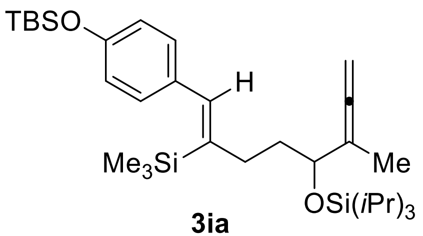

3ia

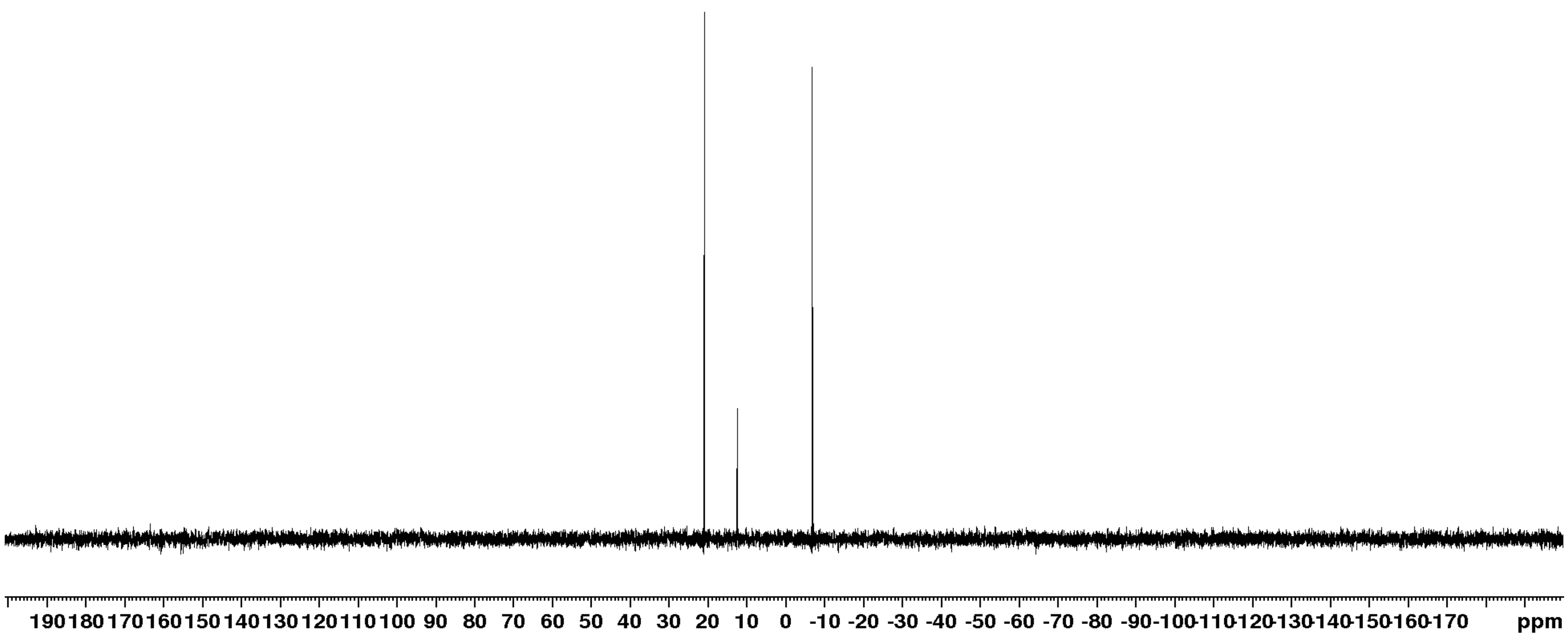

Figure S109. ^{1}H NMR spectrum (500 MHz, $CDCl_3$, 298 K) of **3ja**

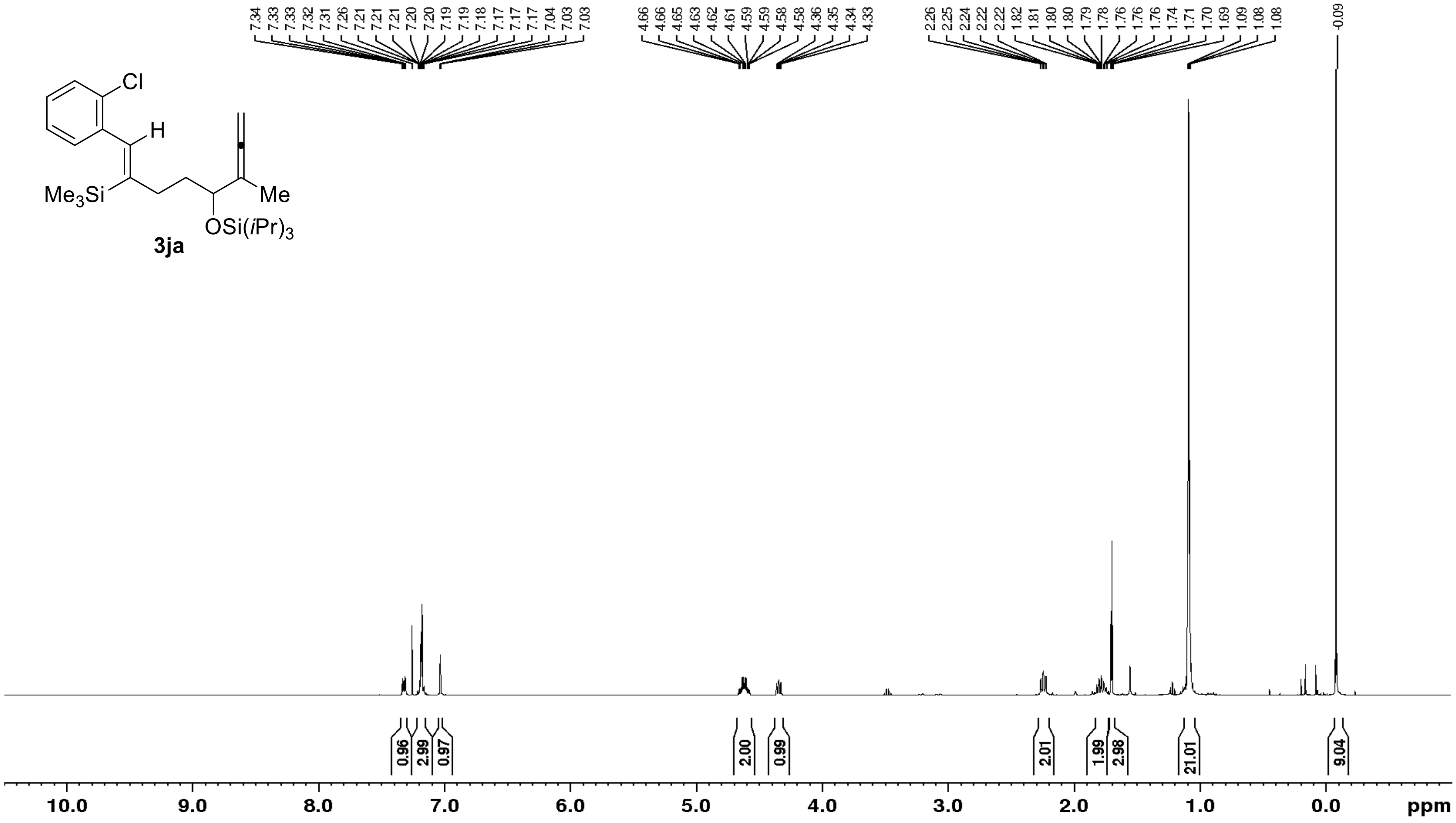

Figure S110. $^{13}C\{^{1}H\}$ NMR spectrum (126 MHz, $CDCl_3$, 298 K) of **3ja**

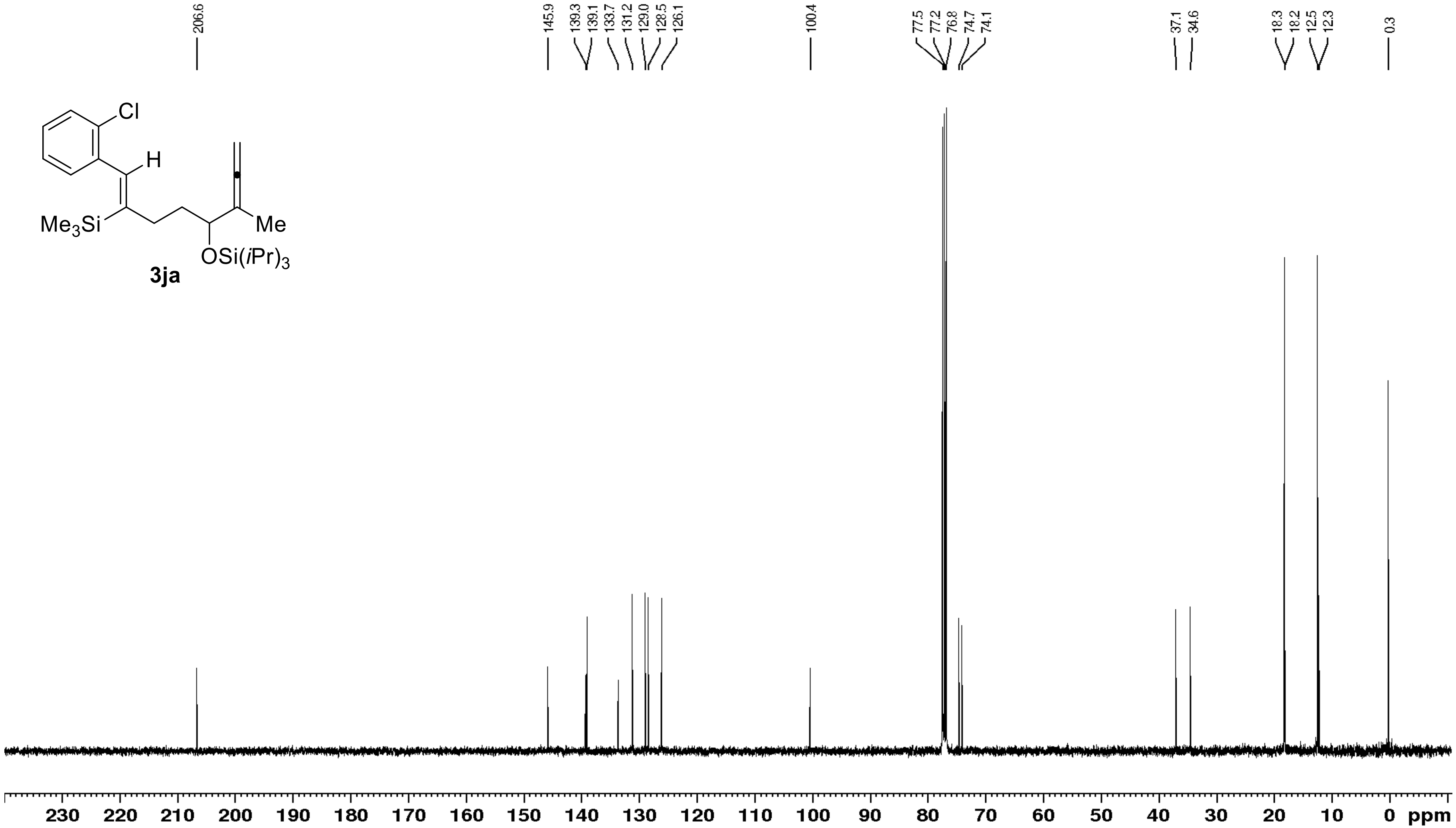

Figure S111. ^{29}Si DEPT NMR spectrum (99 MHz, $CDCl_3$, 298 K, optimized for J = 15.0 Hz) of **3ja**

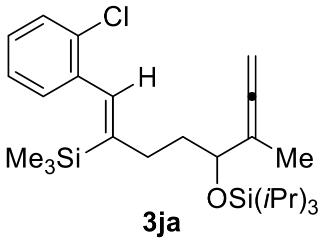

3ja

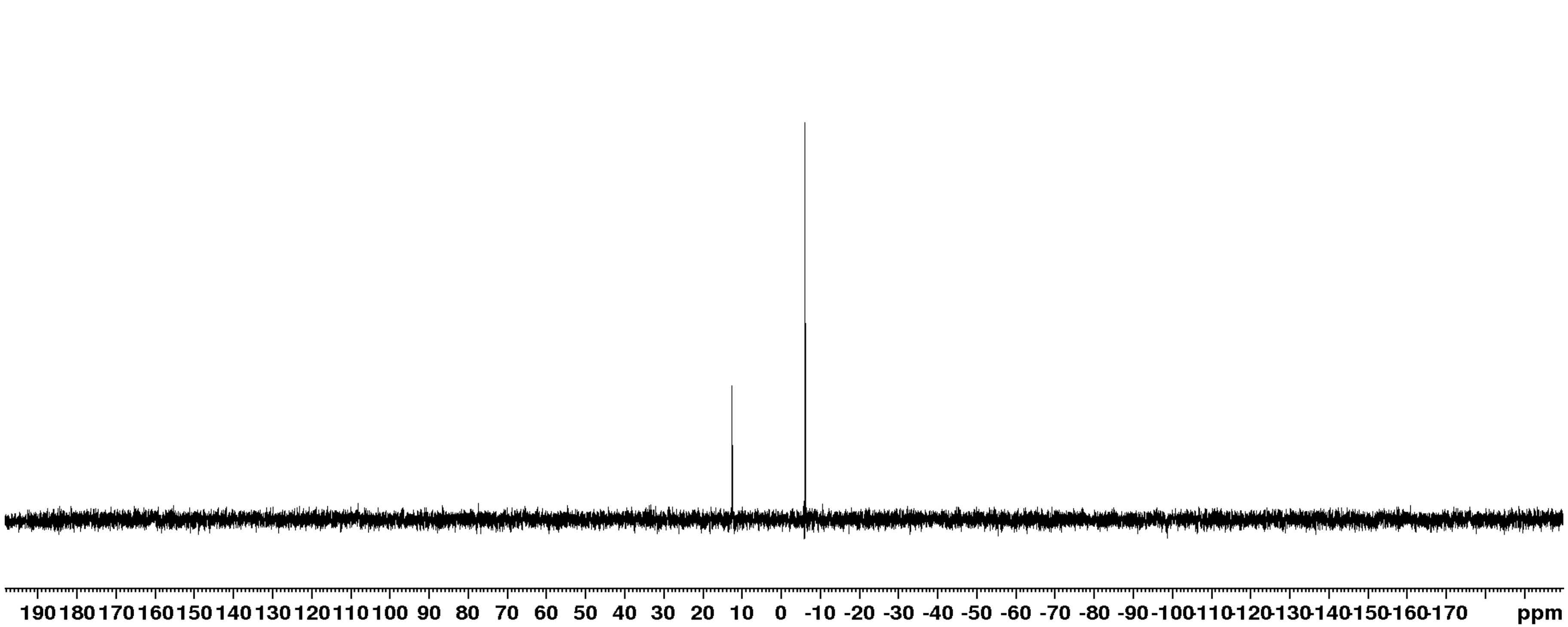

Figure S112. ^{1}H NMR spectrum (400 MHz, $CDCl_3$, 298 K) of **3ka**

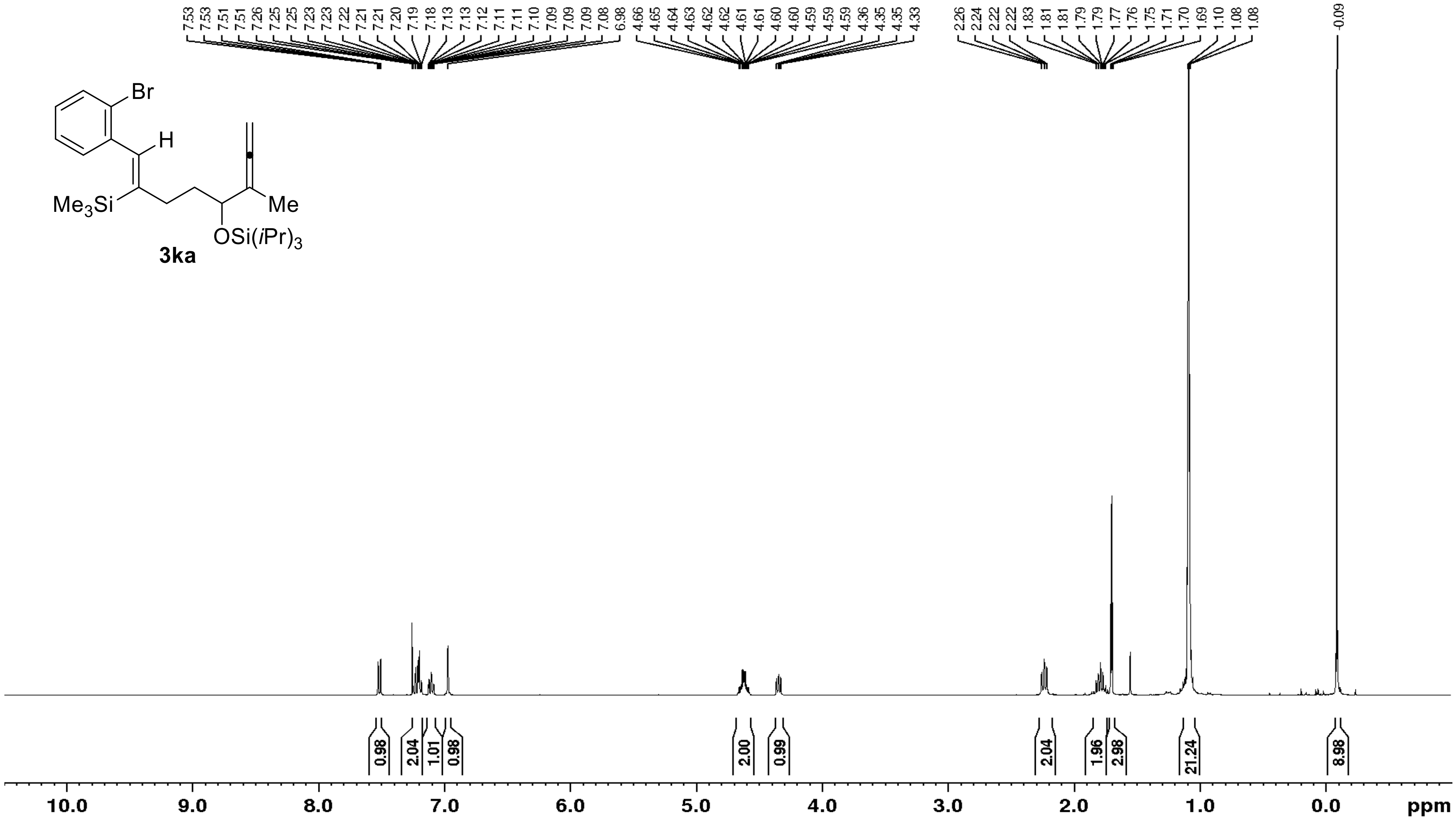

Figure S113. $^{13}C\{^{1}H\}$ NMR spectrum (101 MHz, CDCl₃, 298 K) of **3ka**

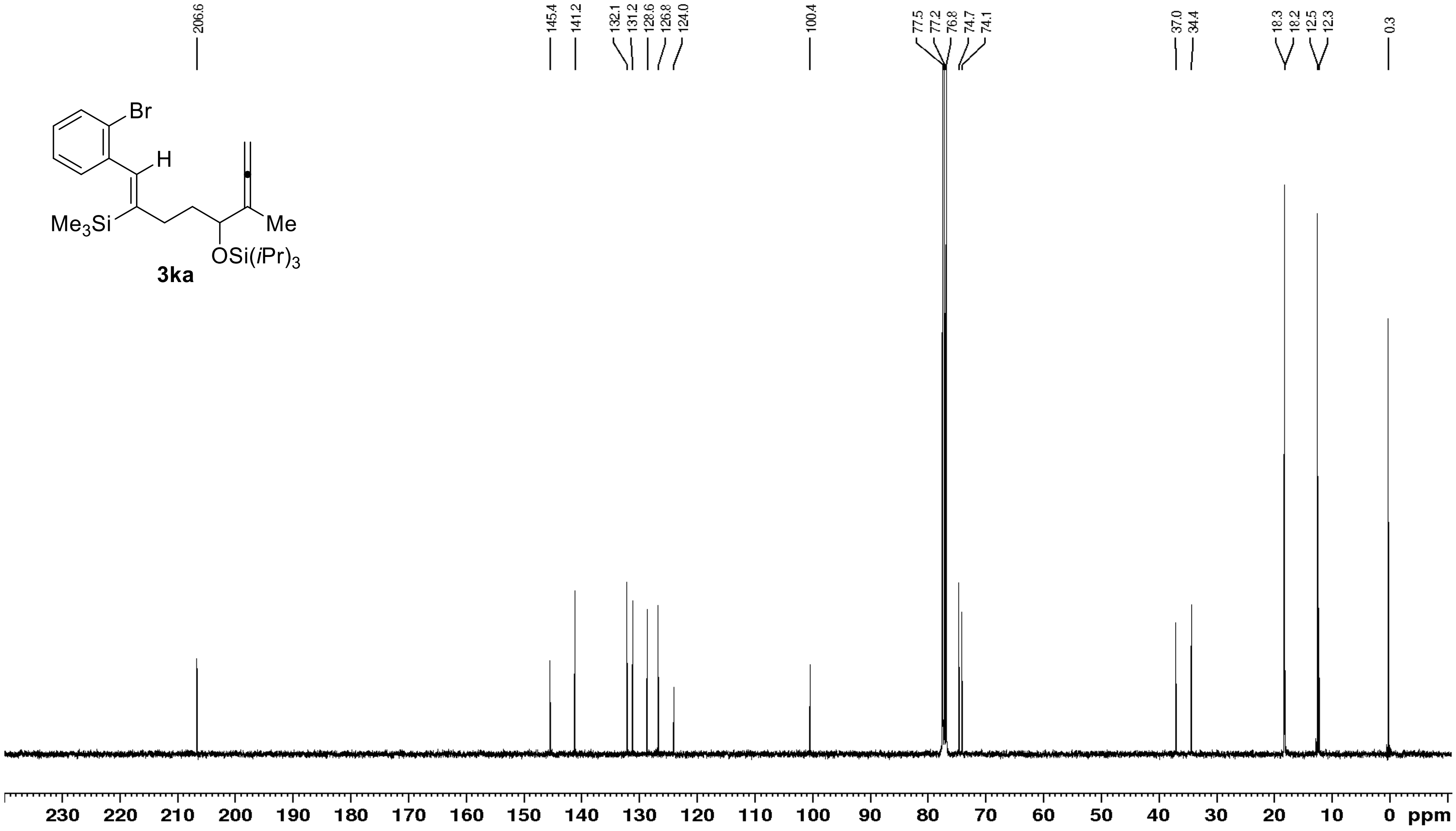

Figure S114. ^{29}Si DEPT NMR spectrum (79 MHz, $CDCl_3$, 298 K, optimized for *J* = 15.0 Hz) of **3ka**

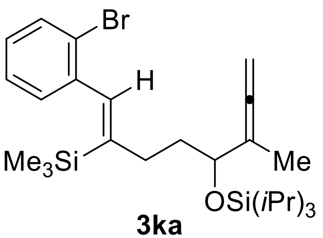

3ka

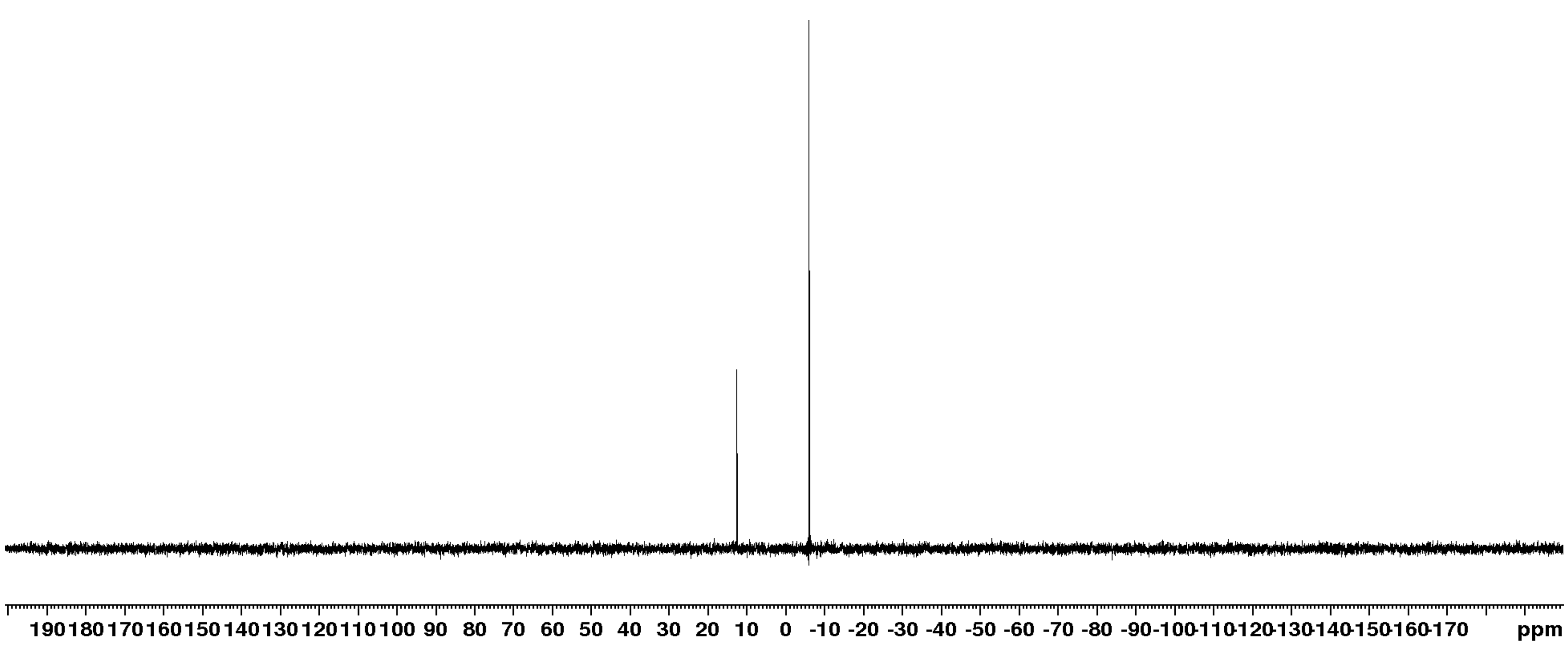

Figure S115. ^{1}H NMR spectrum (500 MHz, $CDCl_3$, 298 K) of **3la**

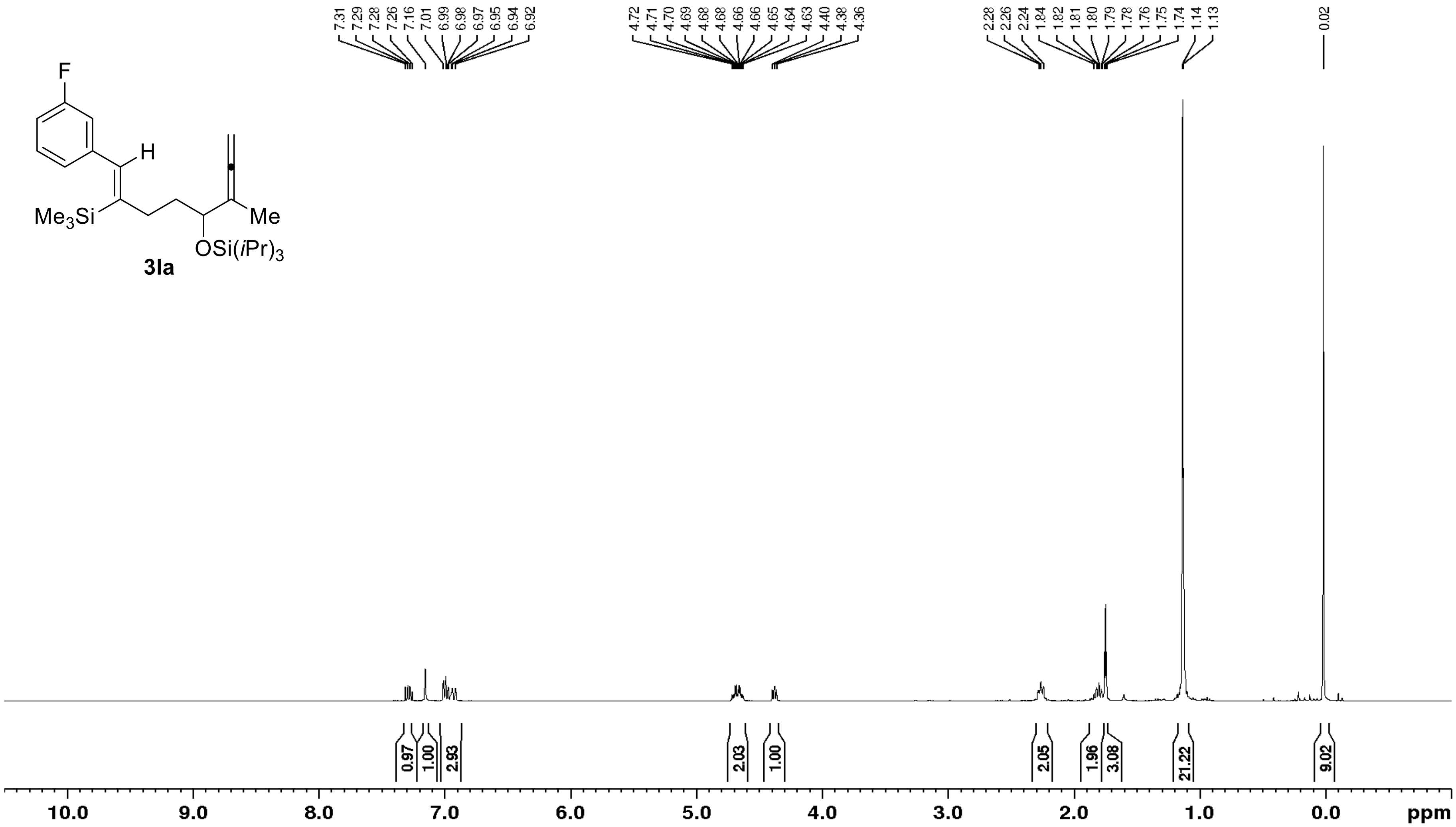

Figure S116. $^{13}C\{^1H\}$ NMR spectrum (126 MHz, $CDCl_3$, 298 K) of **3la**

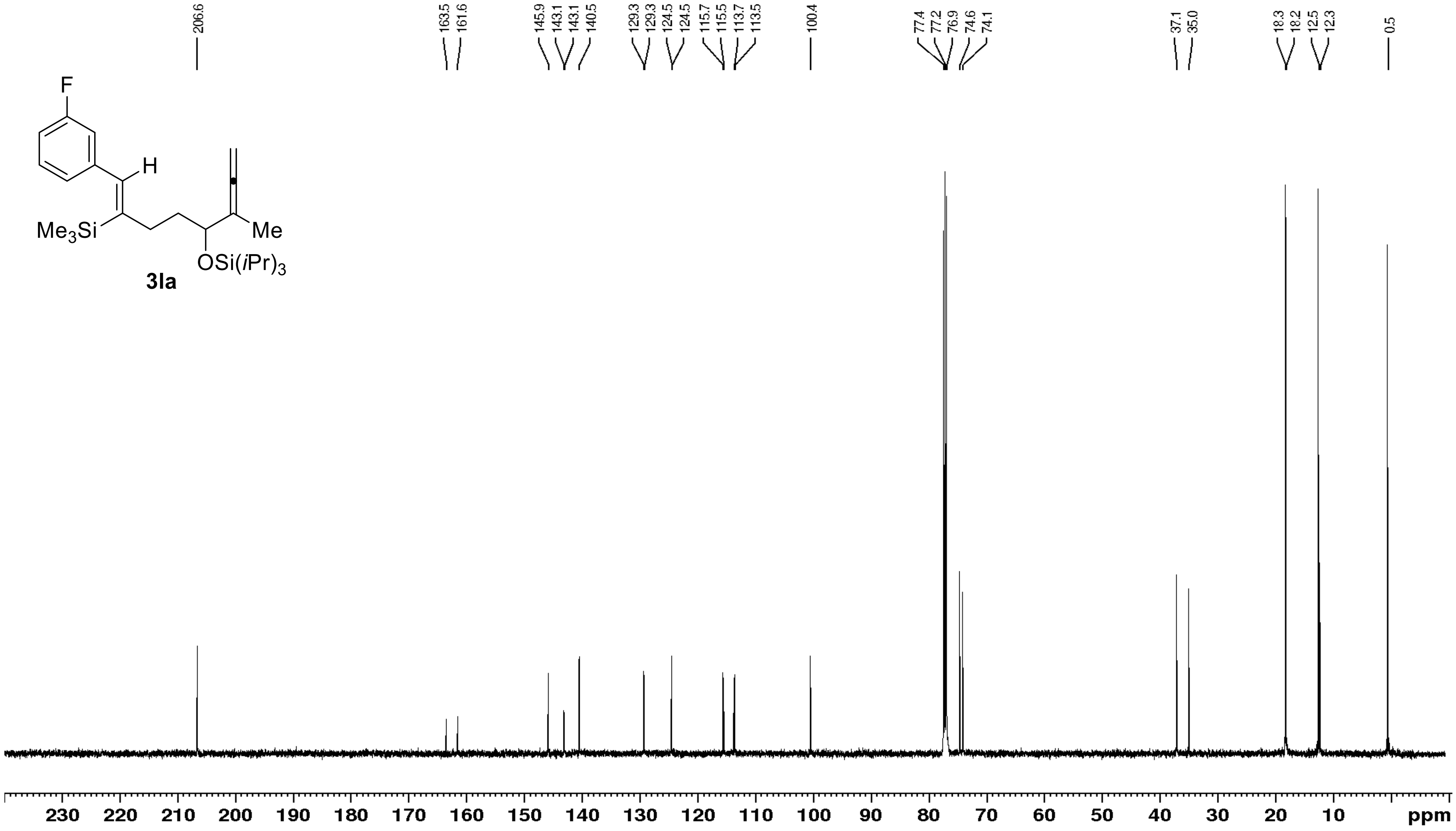

Figure S117. ^{19}F NMR spectrum (471 MHz, $CDCl_3$, 298 K) of **3la**

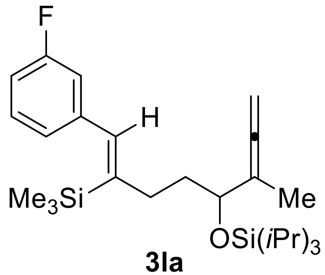

3la

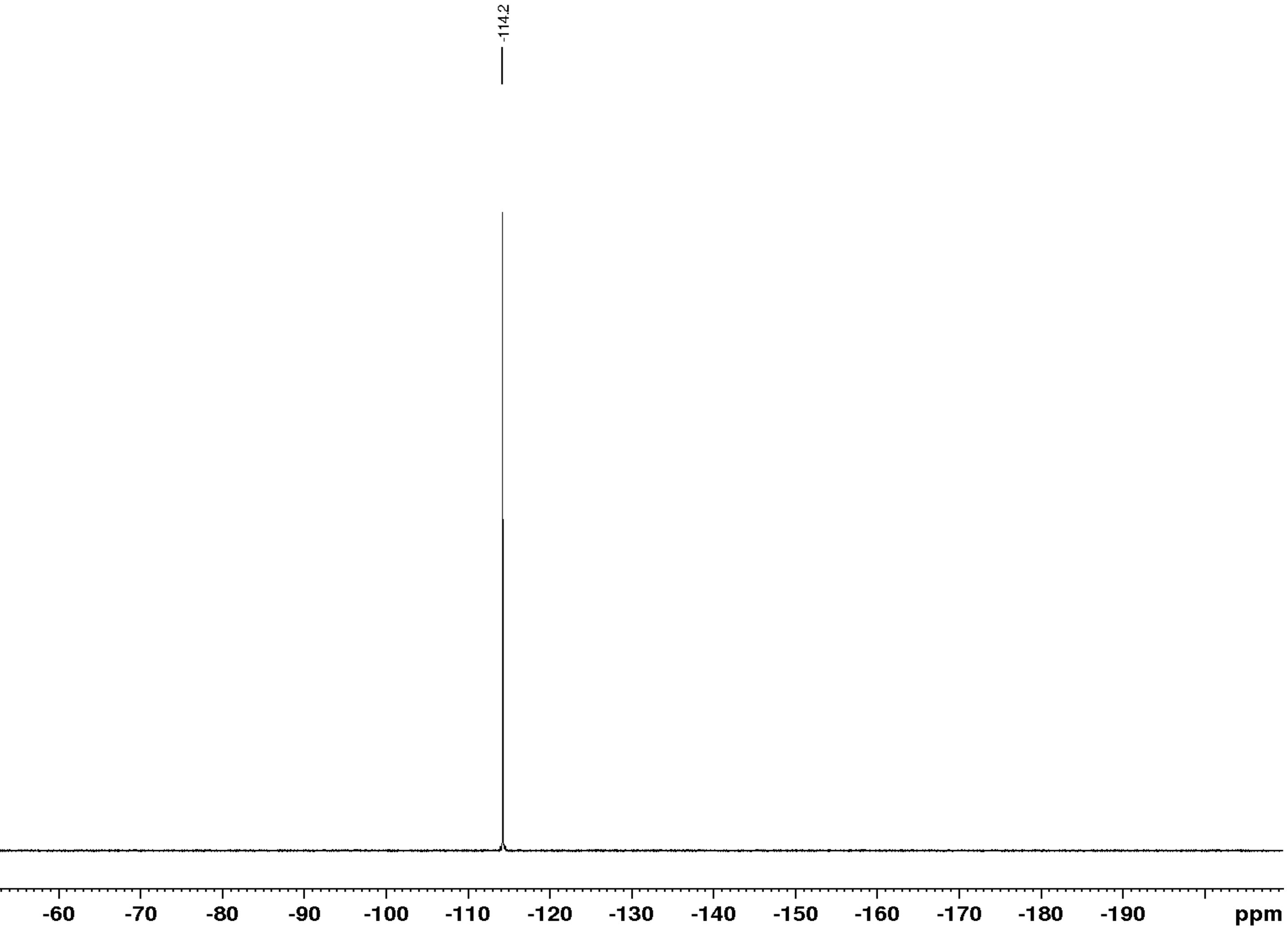

Figure S118. ^{29}Si DEPT NMR spectrum (99 MHz, $CDCl_3$, 298 K, optimized for *J* = 15.0 Hz) of **3la**

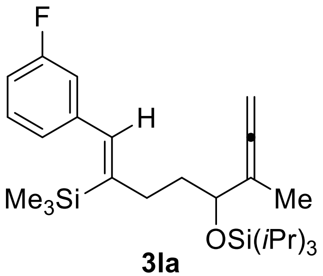

3la

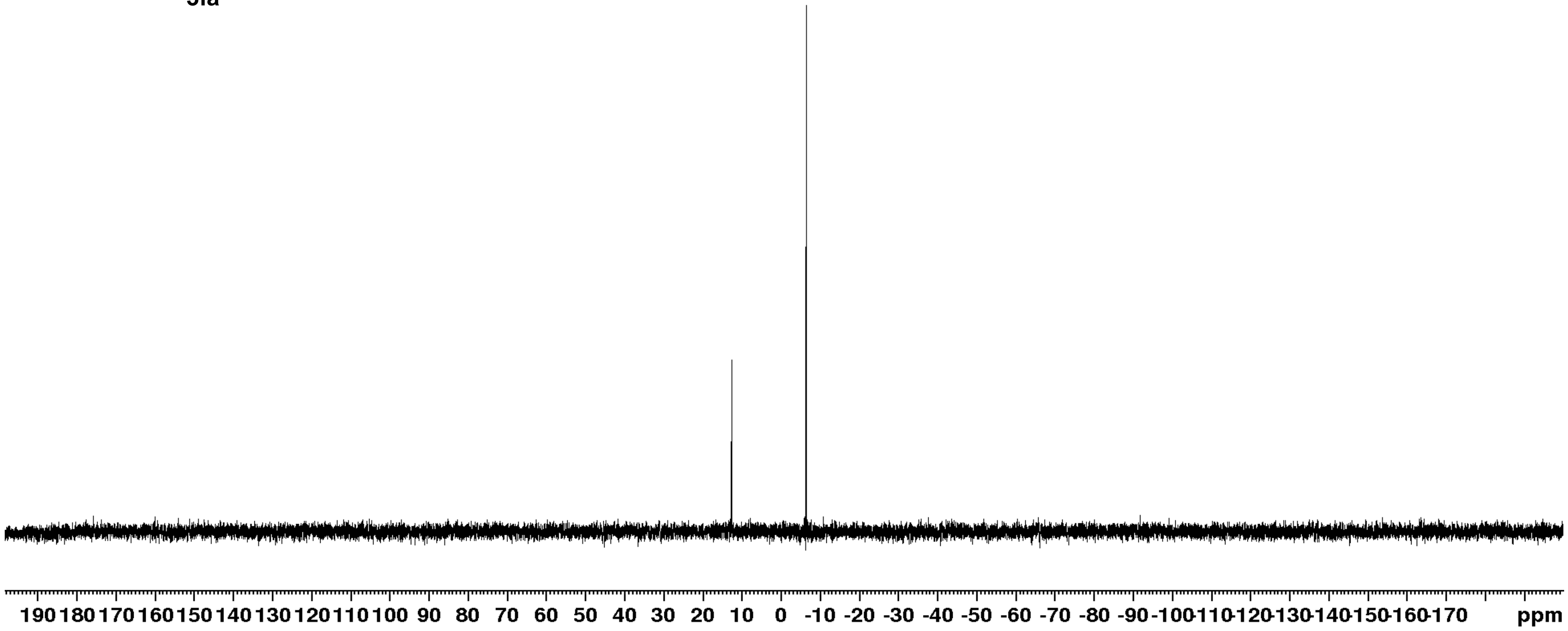

Figure S119. ^{1}H NMR spectrum (500 MHz, $CDCl_3$, 298 K) of **3ma**

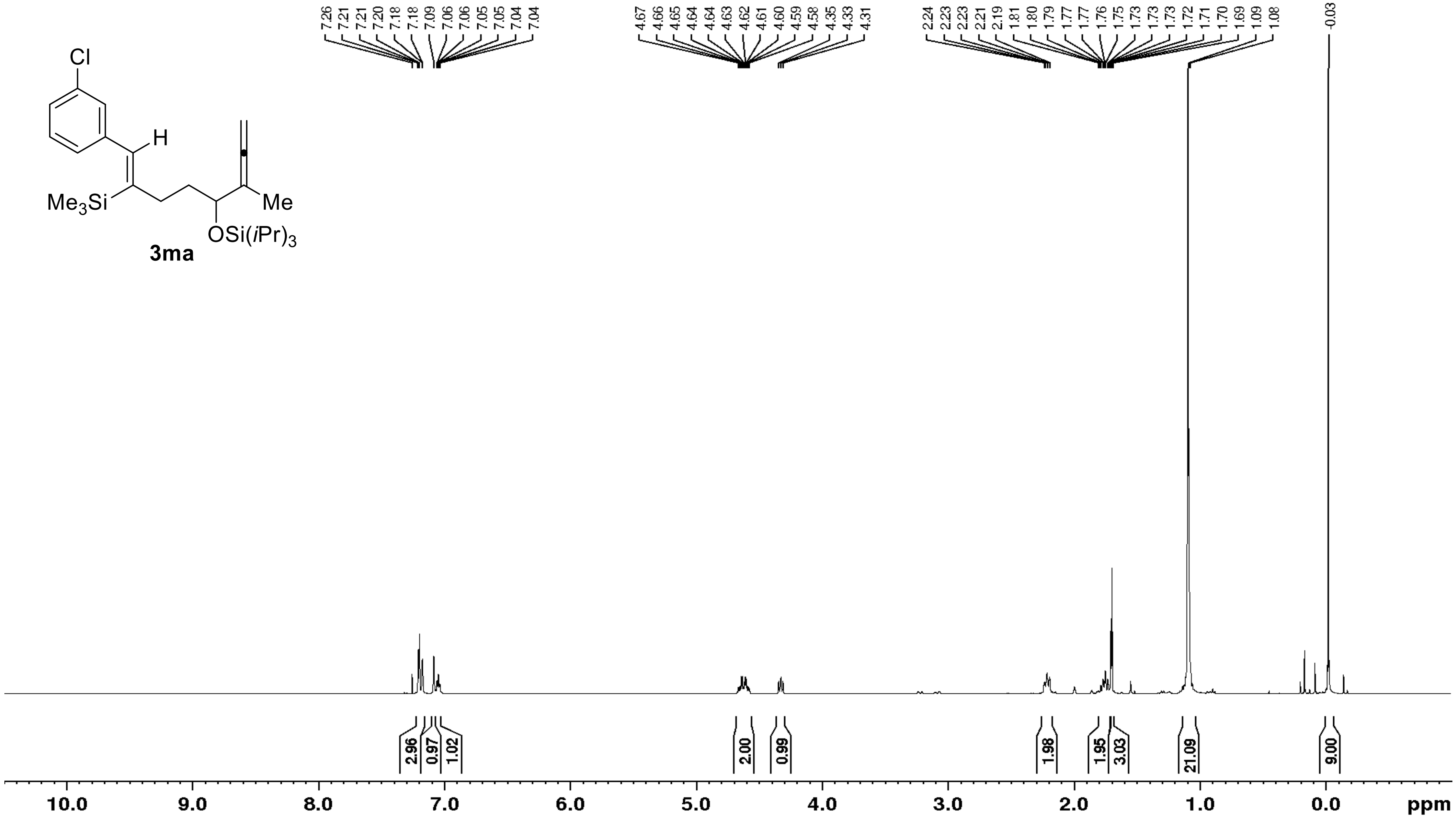

Figure S120. $^{13}C\{^1H\}$ NMR spectrum (126 MHz, $CDCl_3$, 298 K) of **3ma**

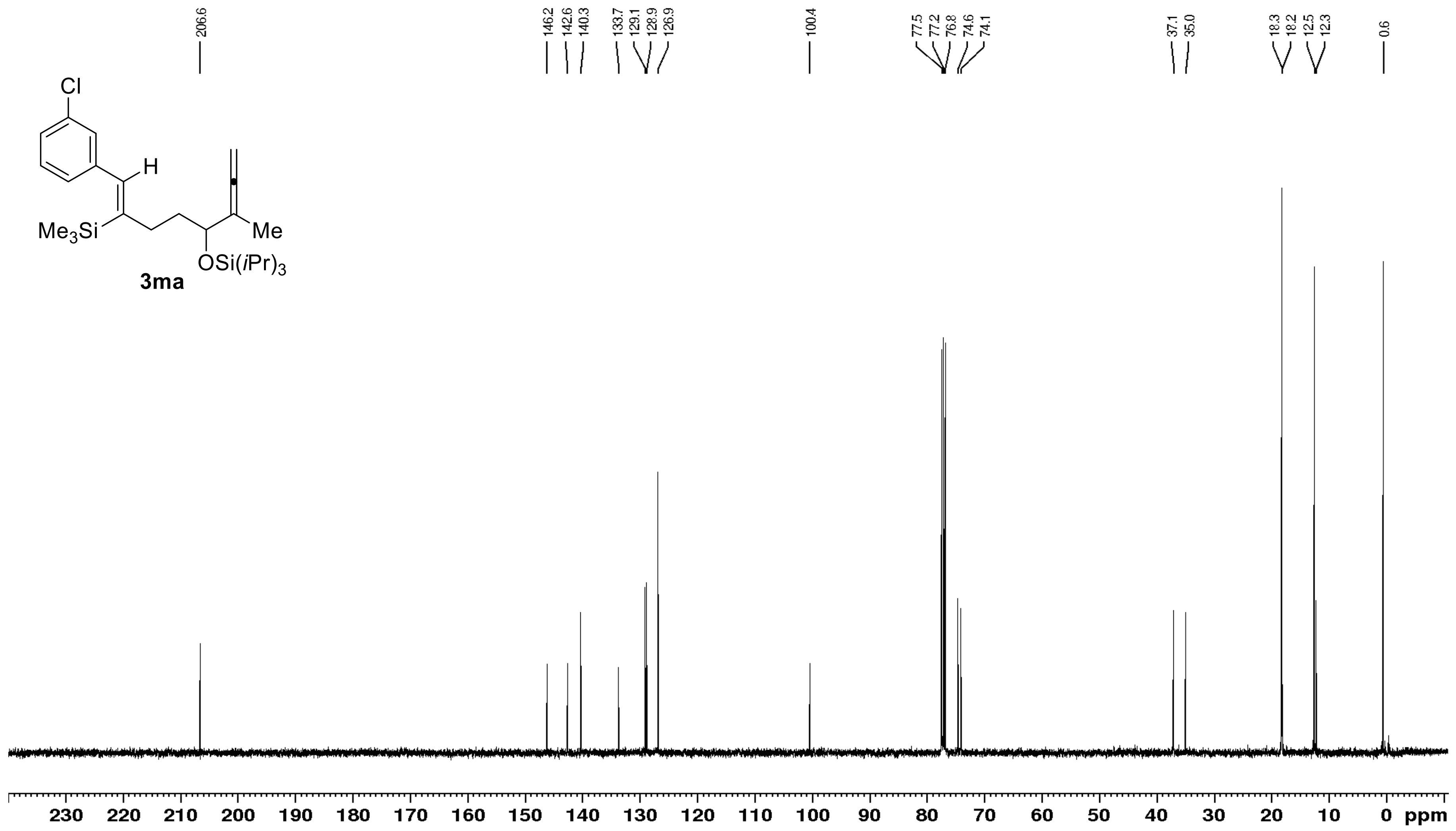

Figure S121. ^{29}Si DEPT NMR spectrum (99 MHz, $CDCl_3$, 298 K, optimized for J = 15.0 Hz) of **3ma**

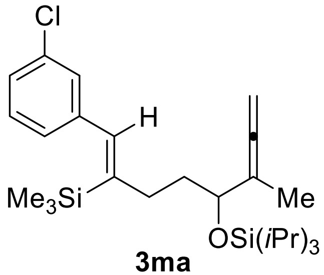

3ma

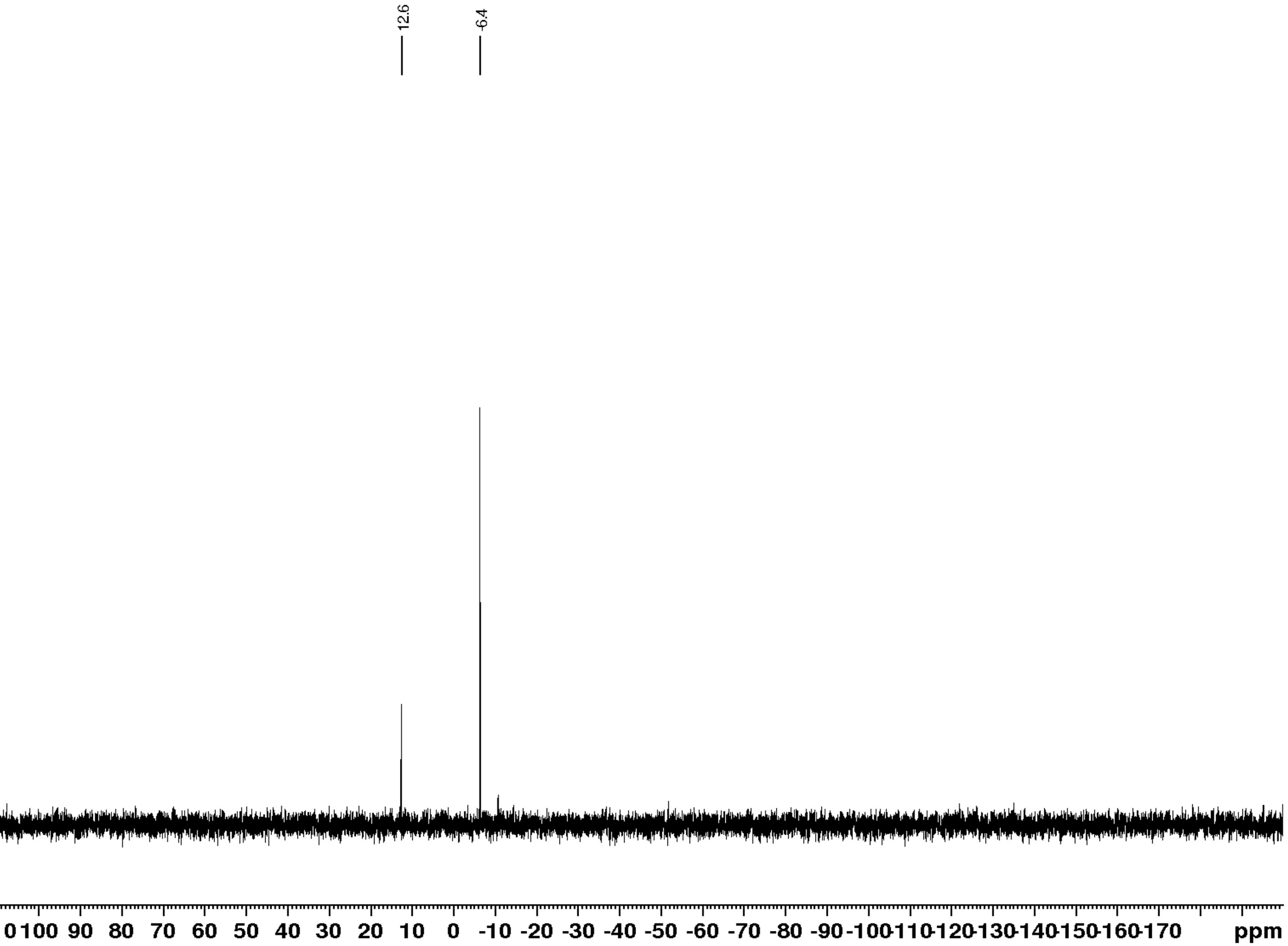

Figure S122. ^{1}H NMR spectrum (400 MHz, $CDCl_3$, 298 K) of **3na**

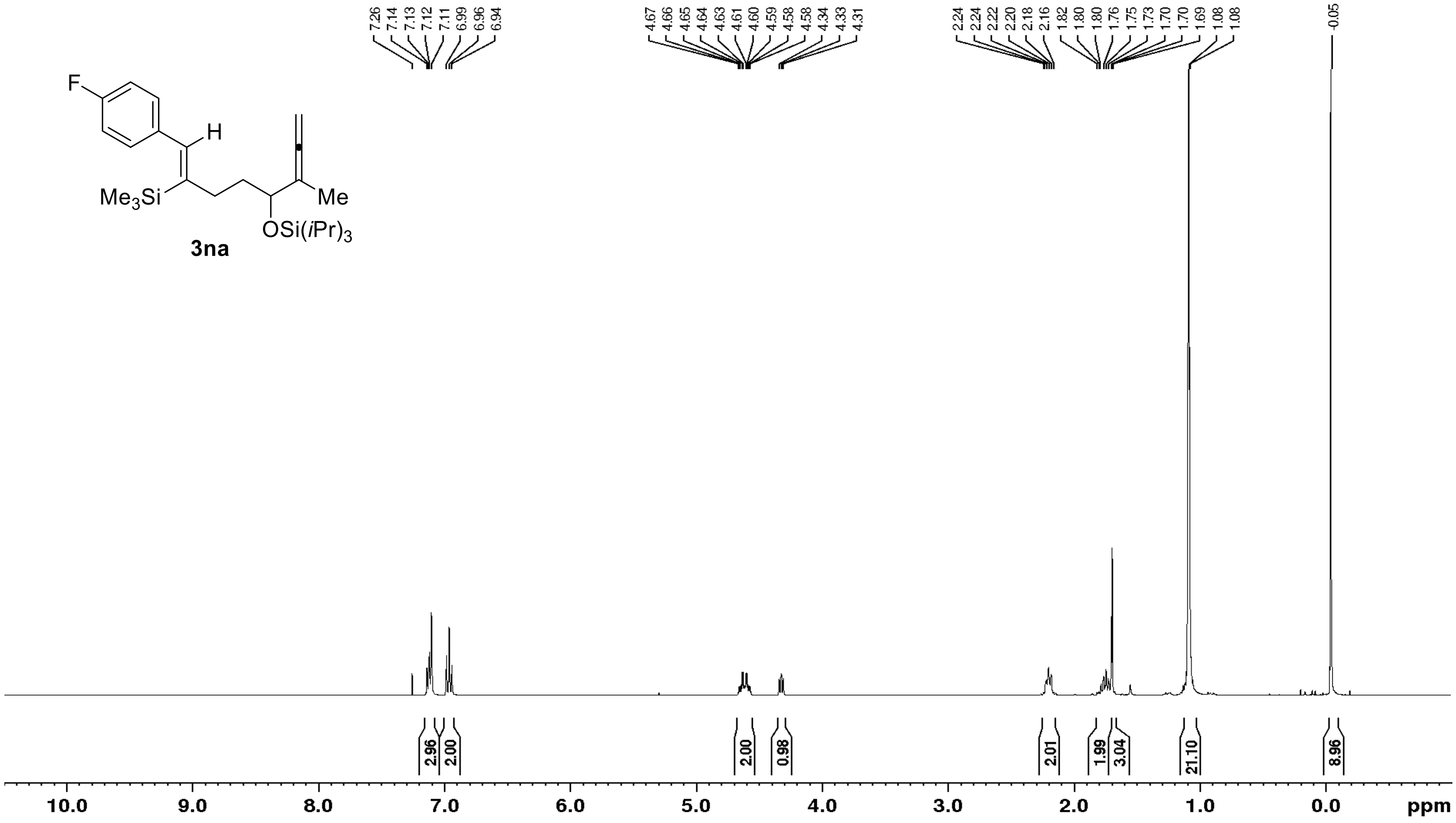

Figure S123. $^{13}C\{^1H\}$ NMR spectrum (101 MHz, $CDCl_3$, 298 K) of **3na**

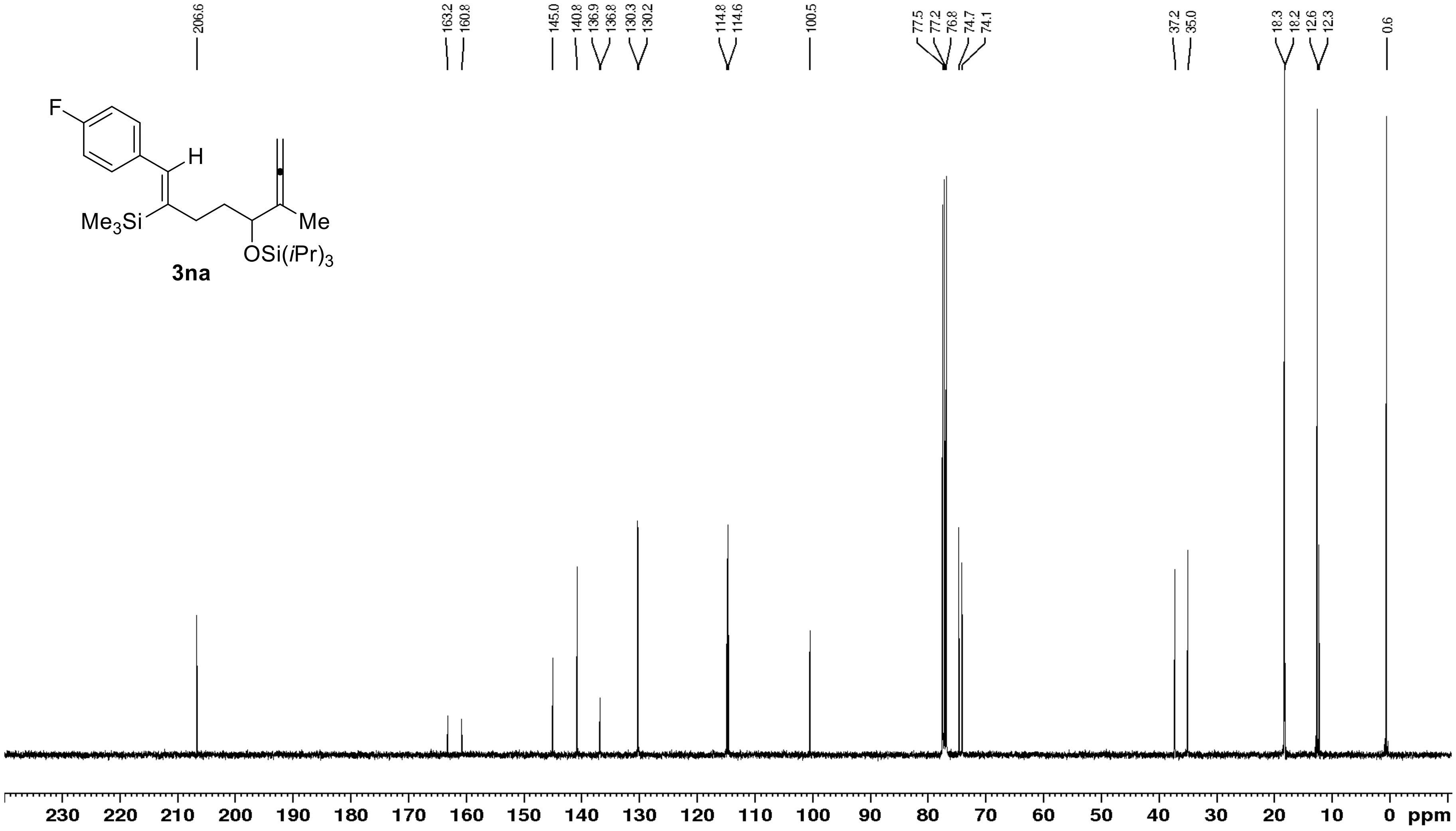

Figure S124. ^{19}F NMR spectrum (471MHz, $CDCl_3$, 298 K) of **3na**

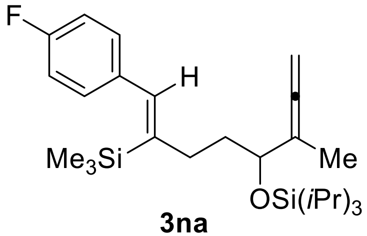

3na

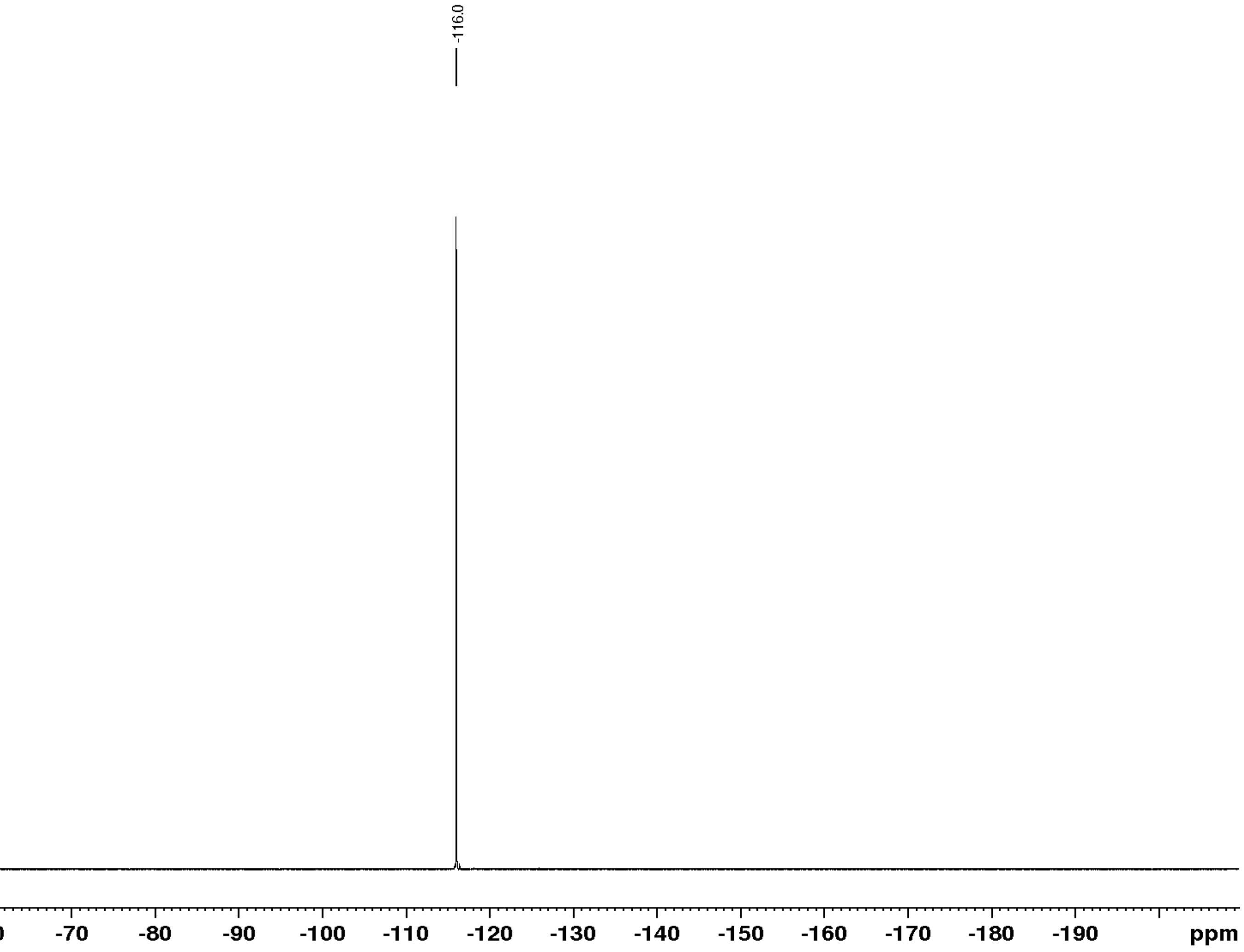

Figure S125. ^{29}Si DEPT NMR spectrum (79 MHz, $CDCl_3$, 298 K, optimized for J = 15.0 Hz) of **3na**

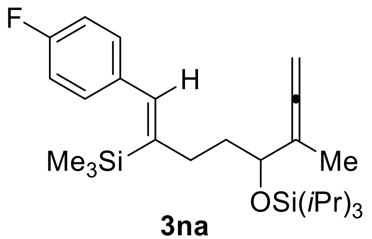

3na

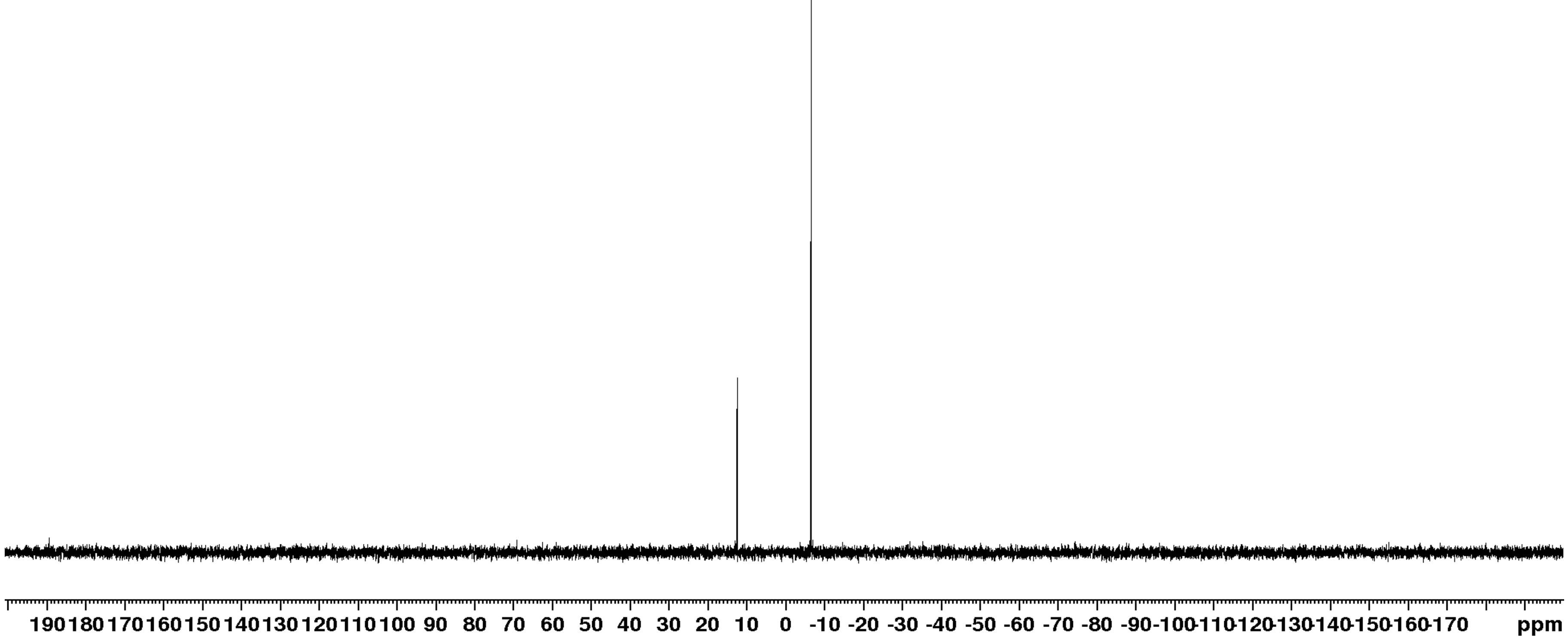

Figure S126. ^{1}H NMR spectrum (400 MHz, $CDCl_3$, 298 K) of **3oa**

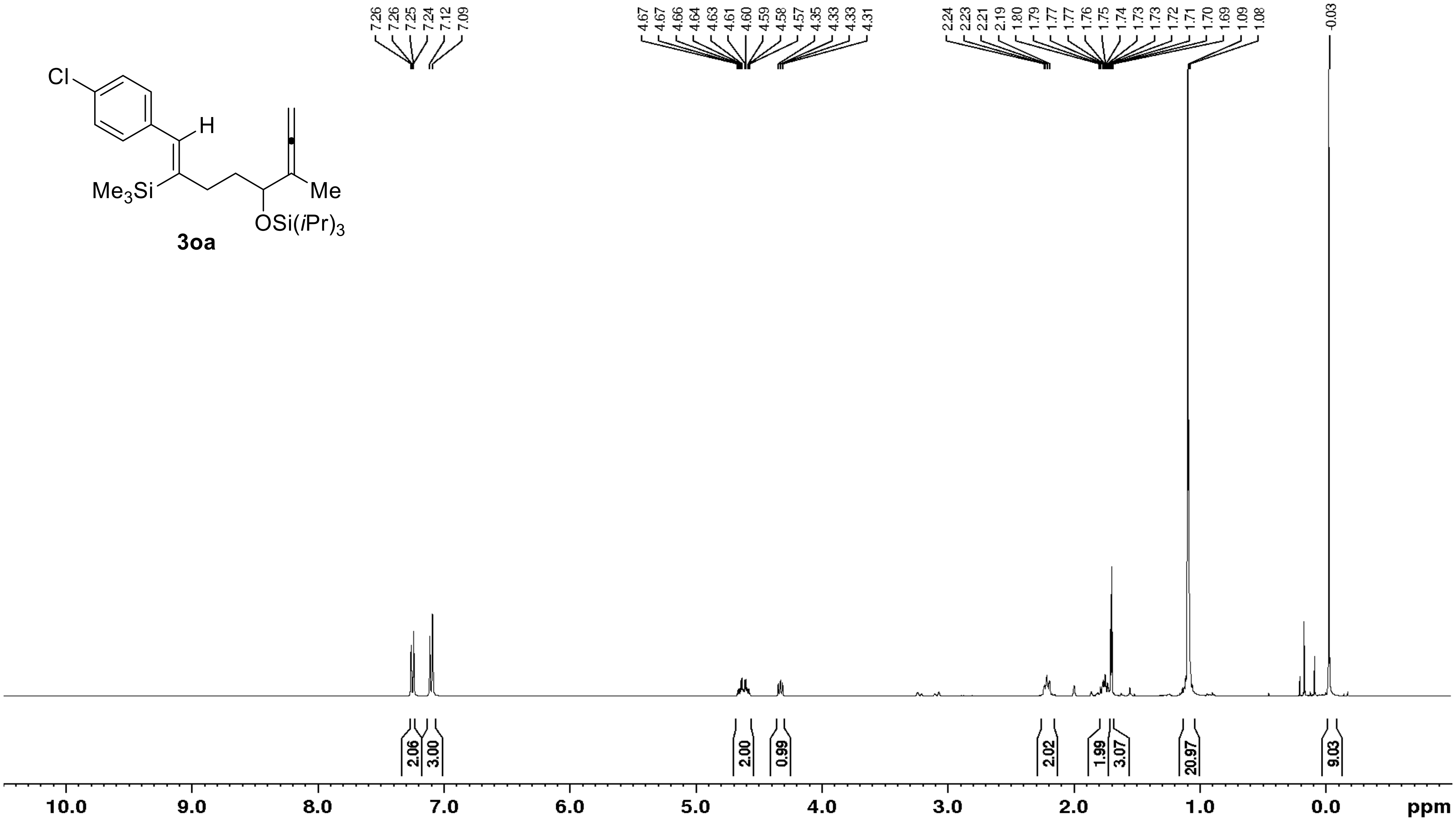

Figure S127. $^{13}C\{^{1}H\}$ NMR spectrum (101 MHz, $CDCl_3$, 298 K) of **3oa**

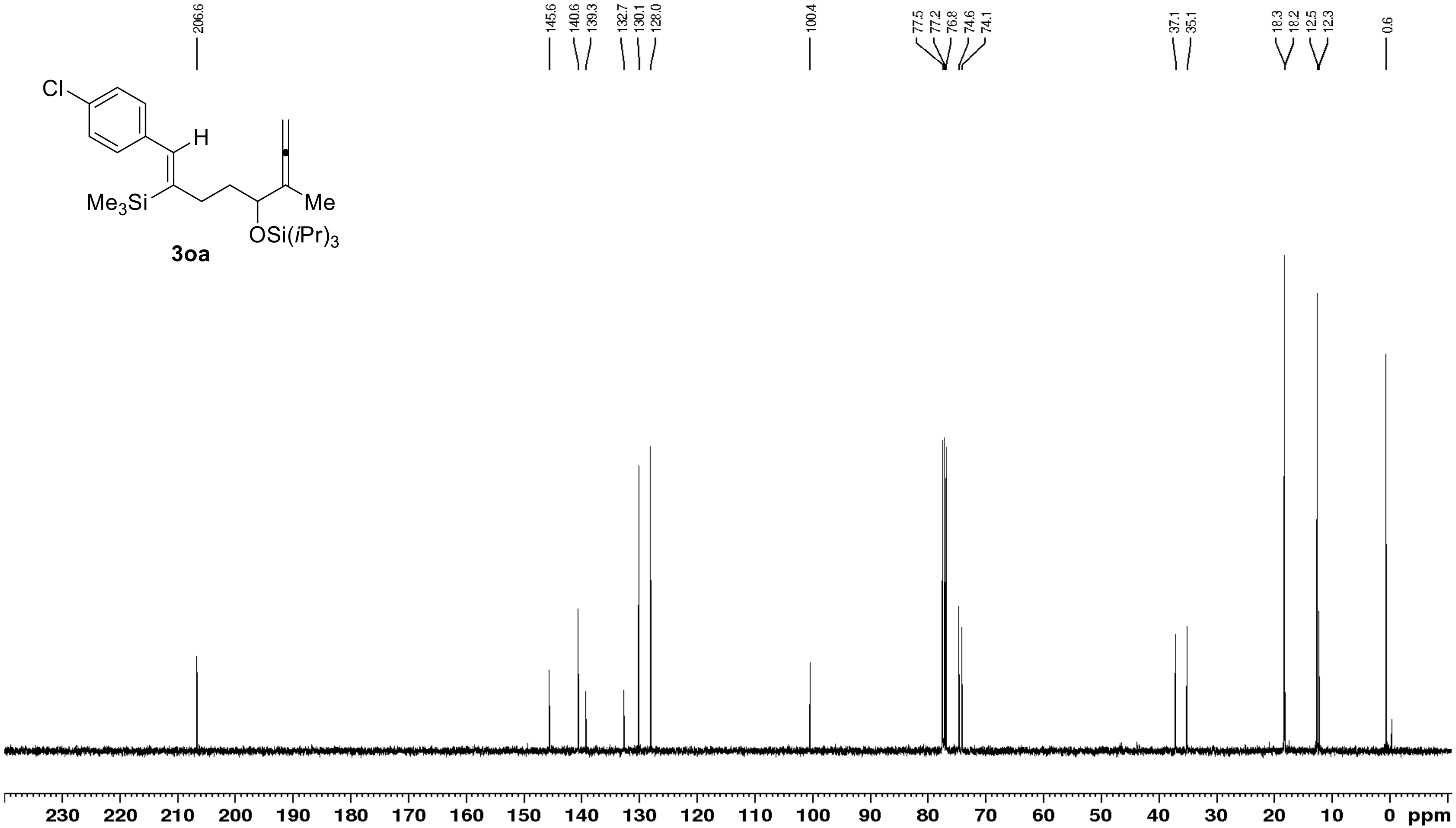

Figure S128. ^{29}Si DEPT NMR spectrum (79 MHz, $CDCl_3$, 298 K, optimized for *J* = 15.0 Hz) of **3oa**

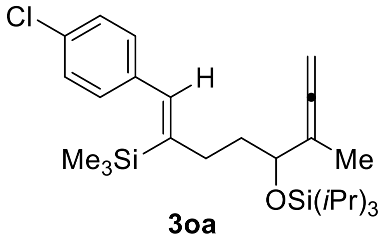

3oa

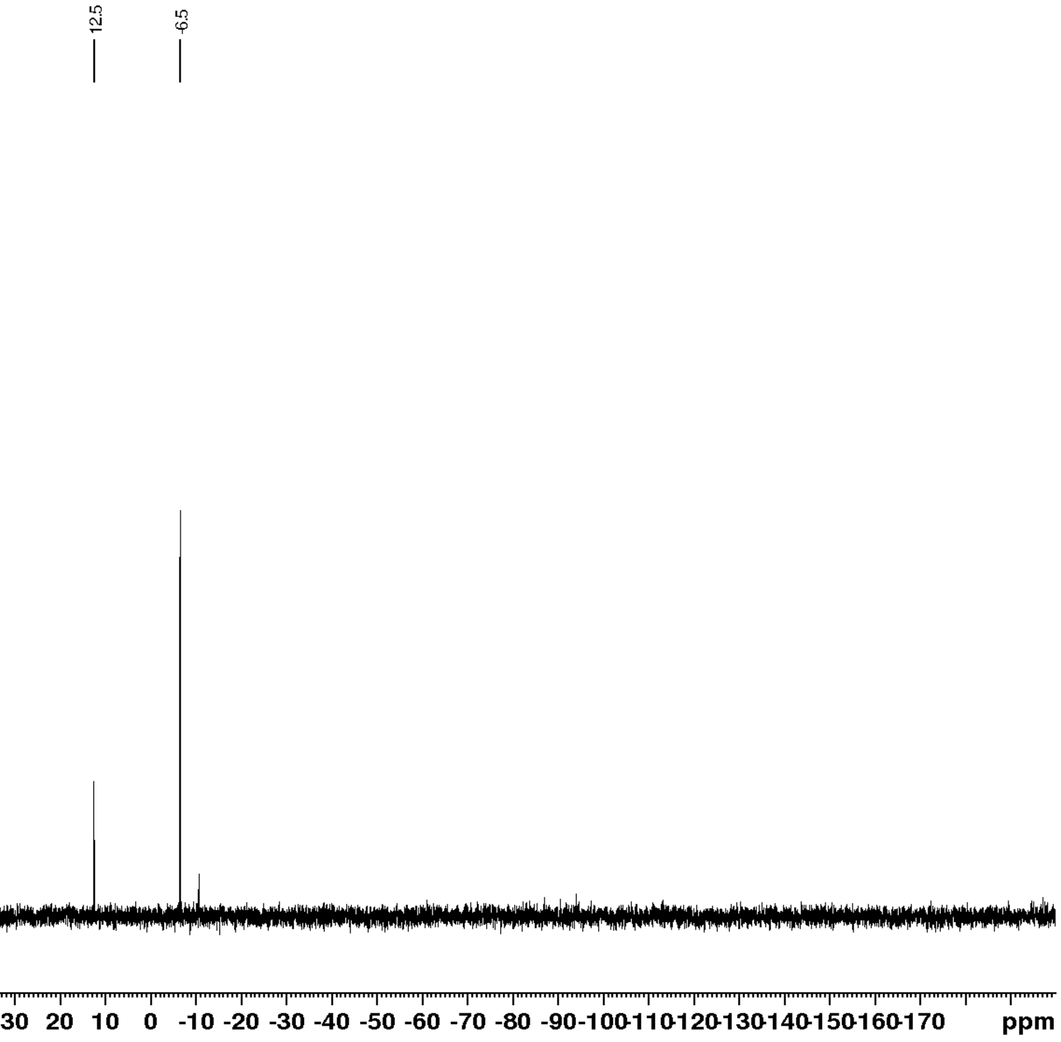

Figure S129. ^{1}H NMR spectrum (400 MHz, $CDCl_3$, 298 K) of **3pa**

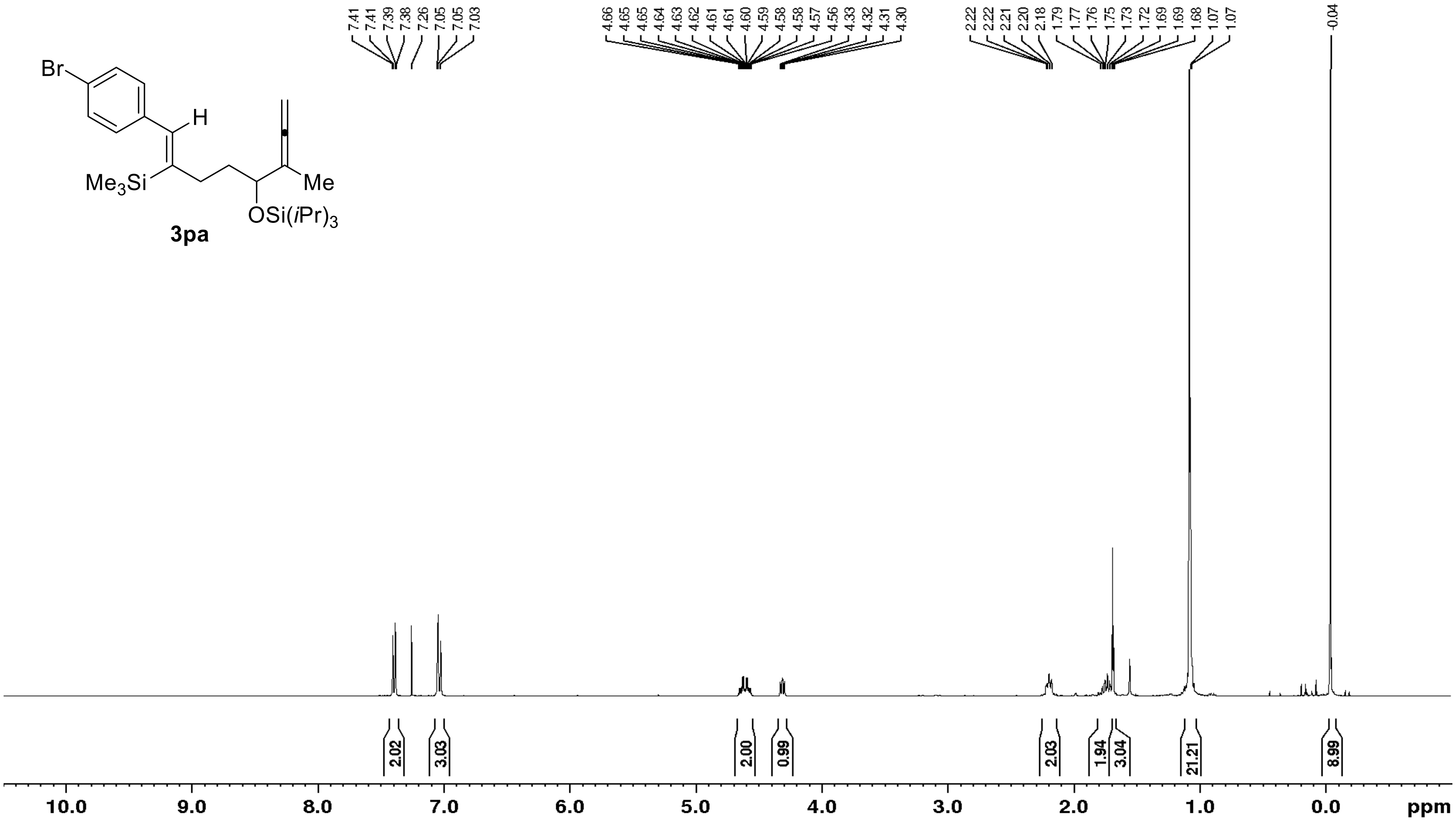

Figure S130. $^{13}C\{^1H\}$ NMR spectrum (101 MHz, $CDCl_3$, 298 K) of **3pa**

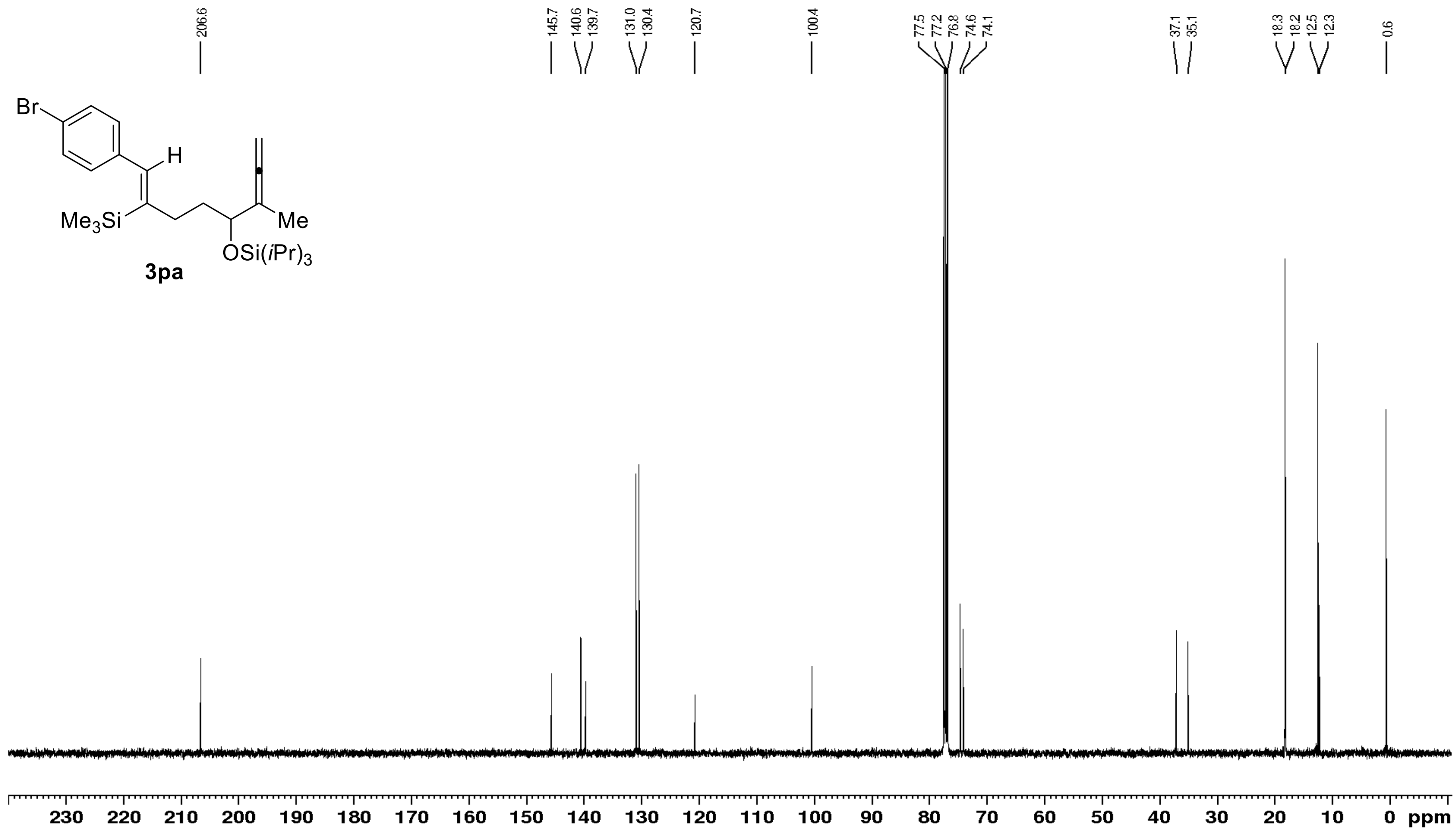

Figure S131. ^{29}Si DEPT NMR spectrum (79 MHz, $CDCl_3$, 298 K, optimized for J = 15.0 Hz) of **3pa**

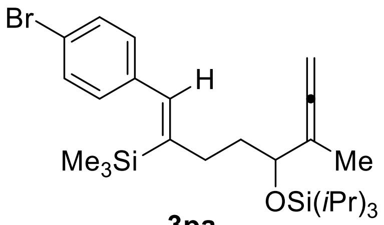

3pa

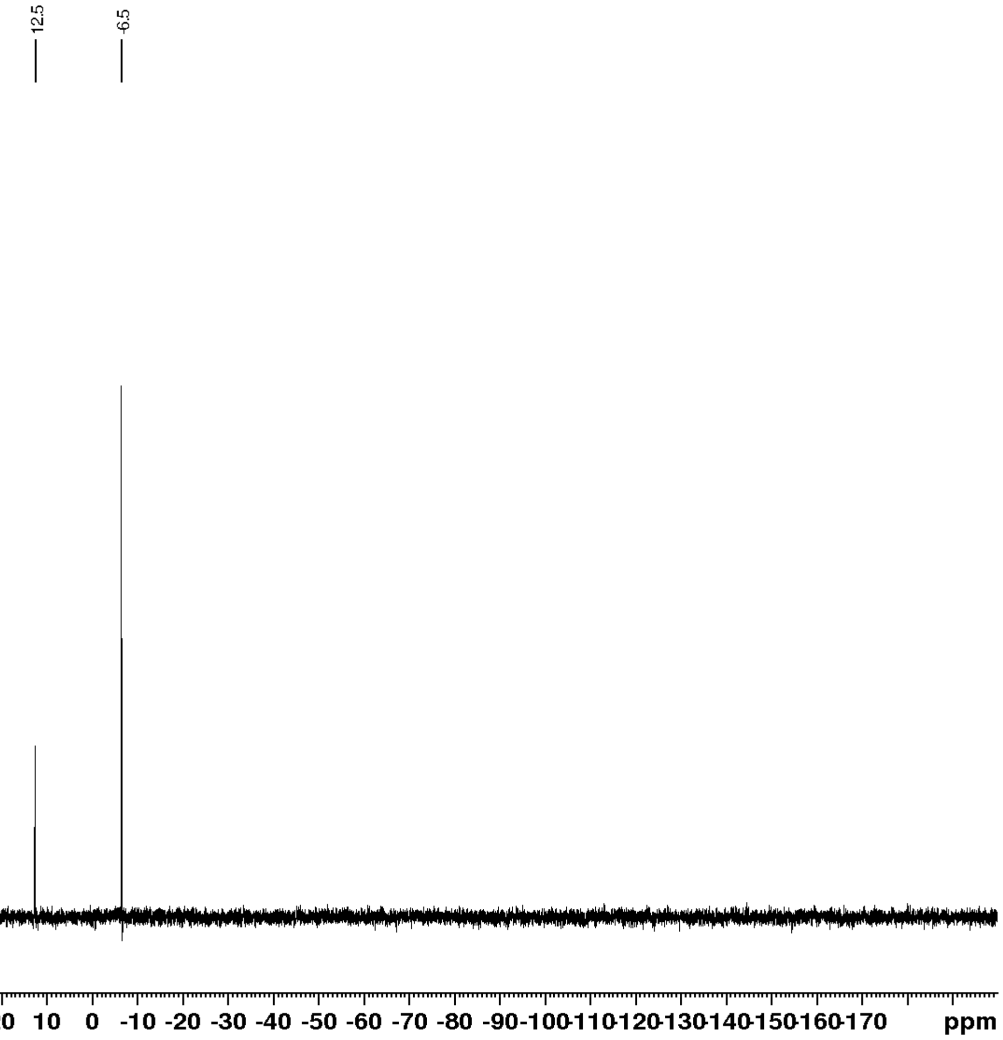

Figure S132. ^{1}H NMR spectrum (400 MHz, $CDCl_3$, 298 K) of **3qa**

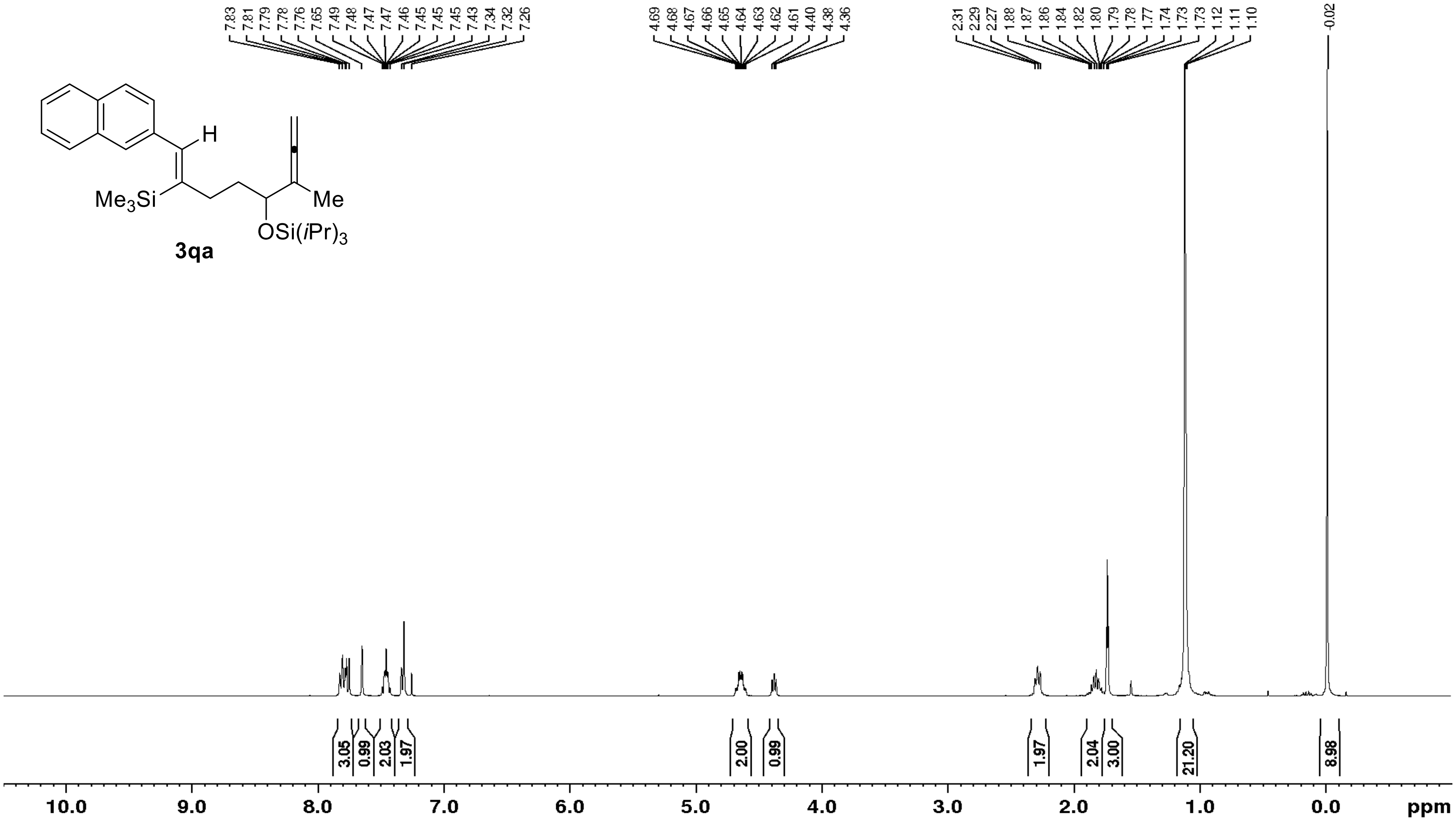

Figure S133. $^{13}C\{^{1}H\}$ NMR spectrum (101 MHz, $CDCl_3$, 298 K) of **3qa**

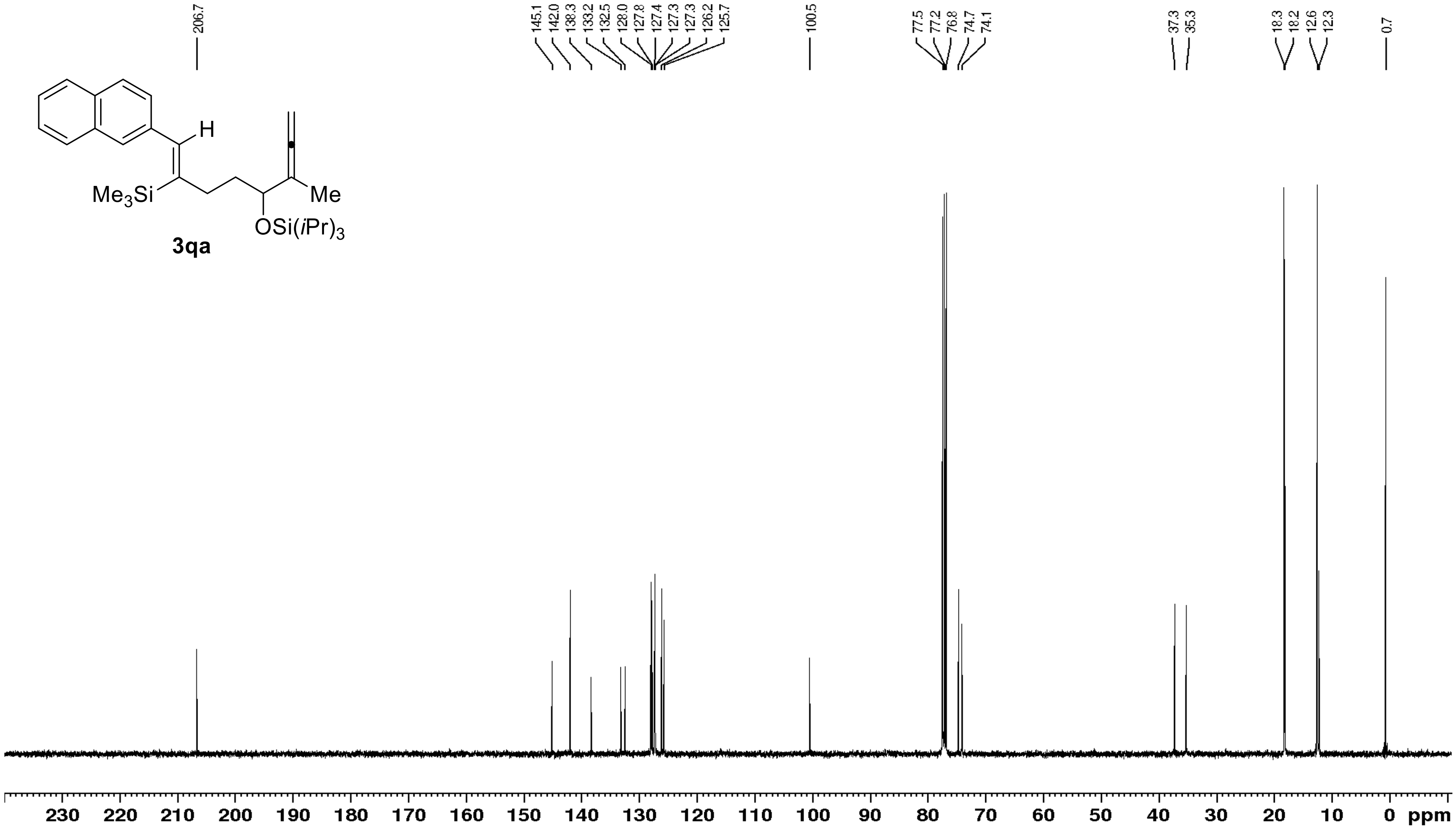

Figure S134. ^{29}Si DEPT NMR spectrum (79 MHz, $CDCl_3$, 298 K, optimized for *J* = 15.0 Hz) of **3qa**

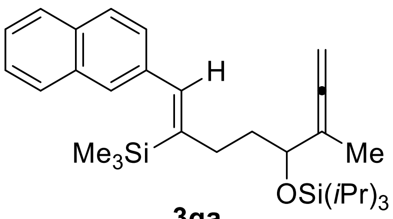

3qa

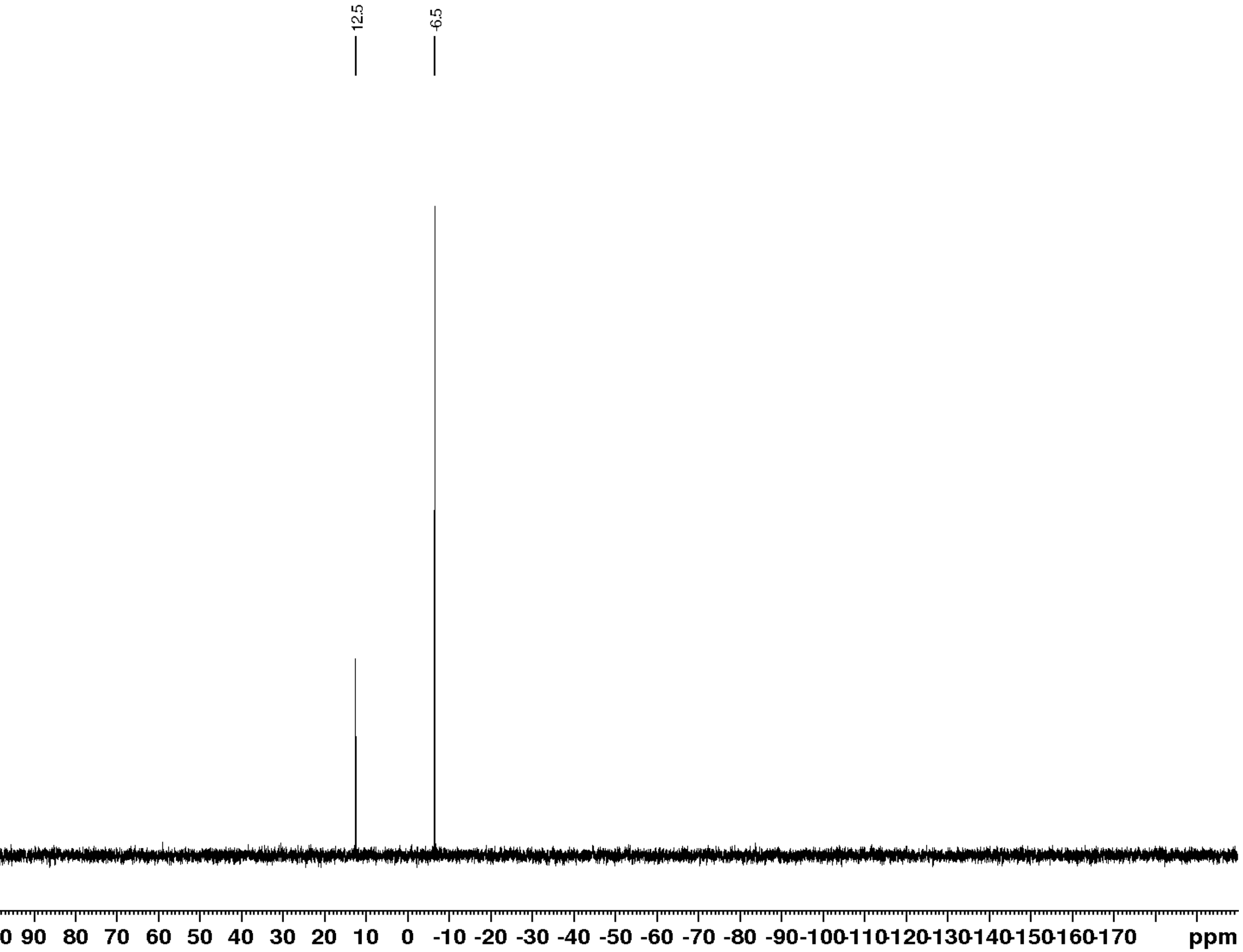

Figure S135. ^{1}H NMR spectrum (400 MHz, $CDCl_3$, 298 K) of **3ra**

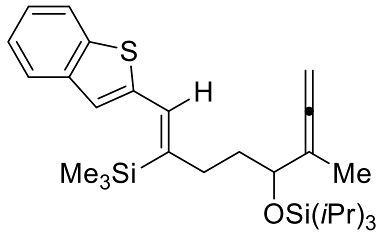

3ra

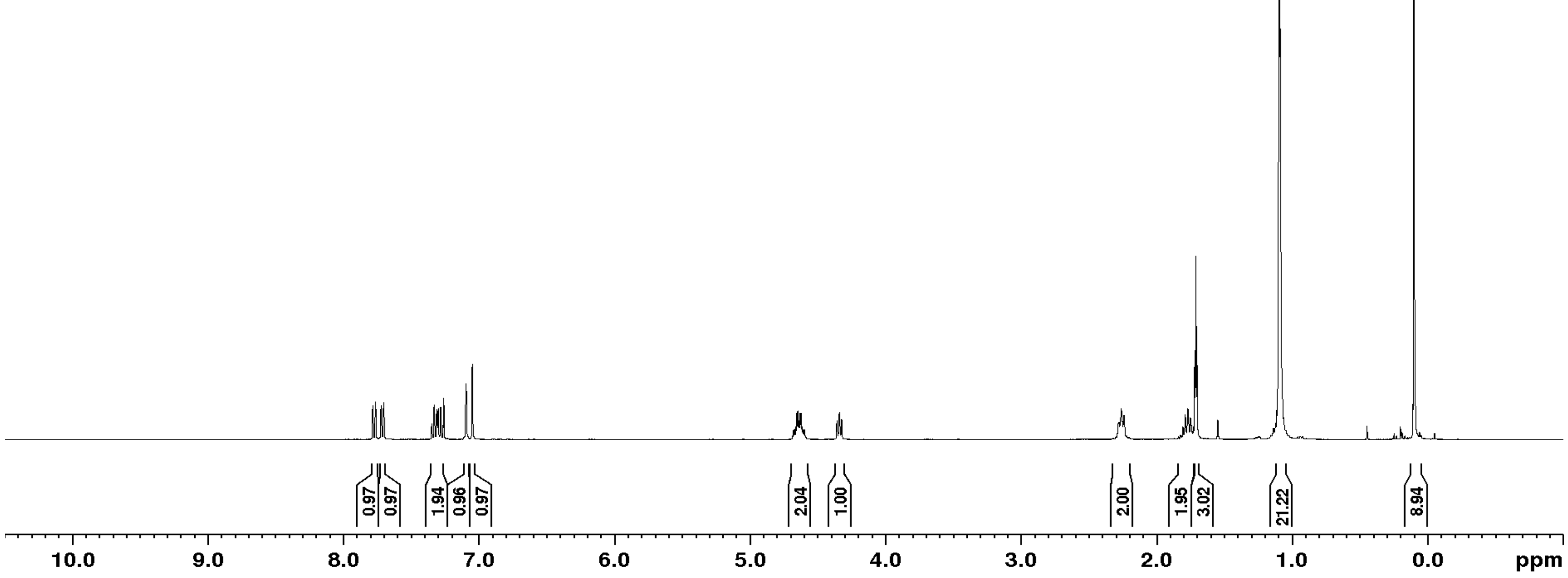

Figure S136. $^{13}C\{^1H\}$ NMR spectrum (101 MHz, $CDCl_3$, 298 K) of **3ra**

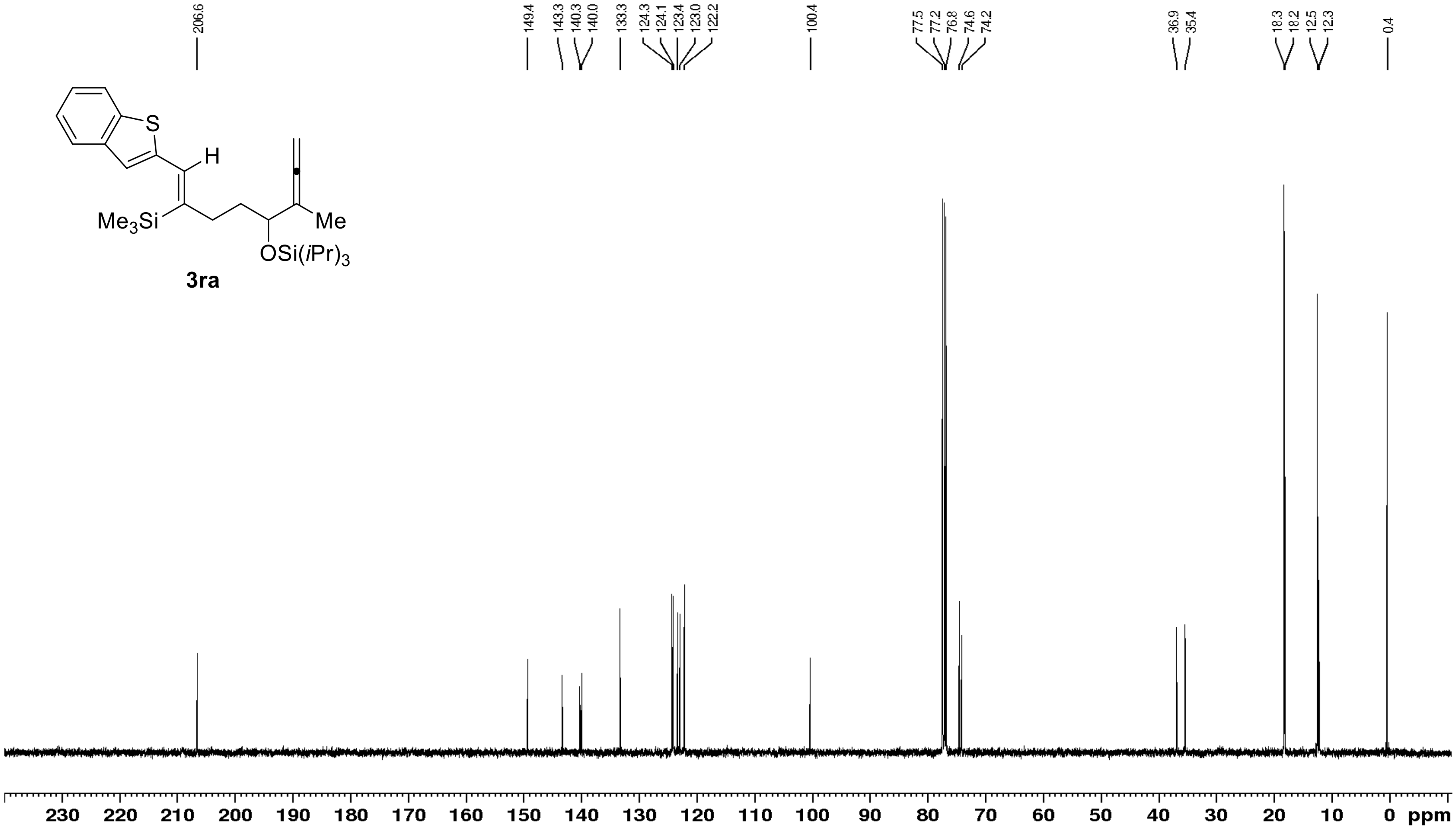

Figure S137. ^{29}Si DEPT NMR spectrum (79 MHz, $CDCl_3$, 298 K, optimized for *J* = 15.0 Hz) of **3ra**

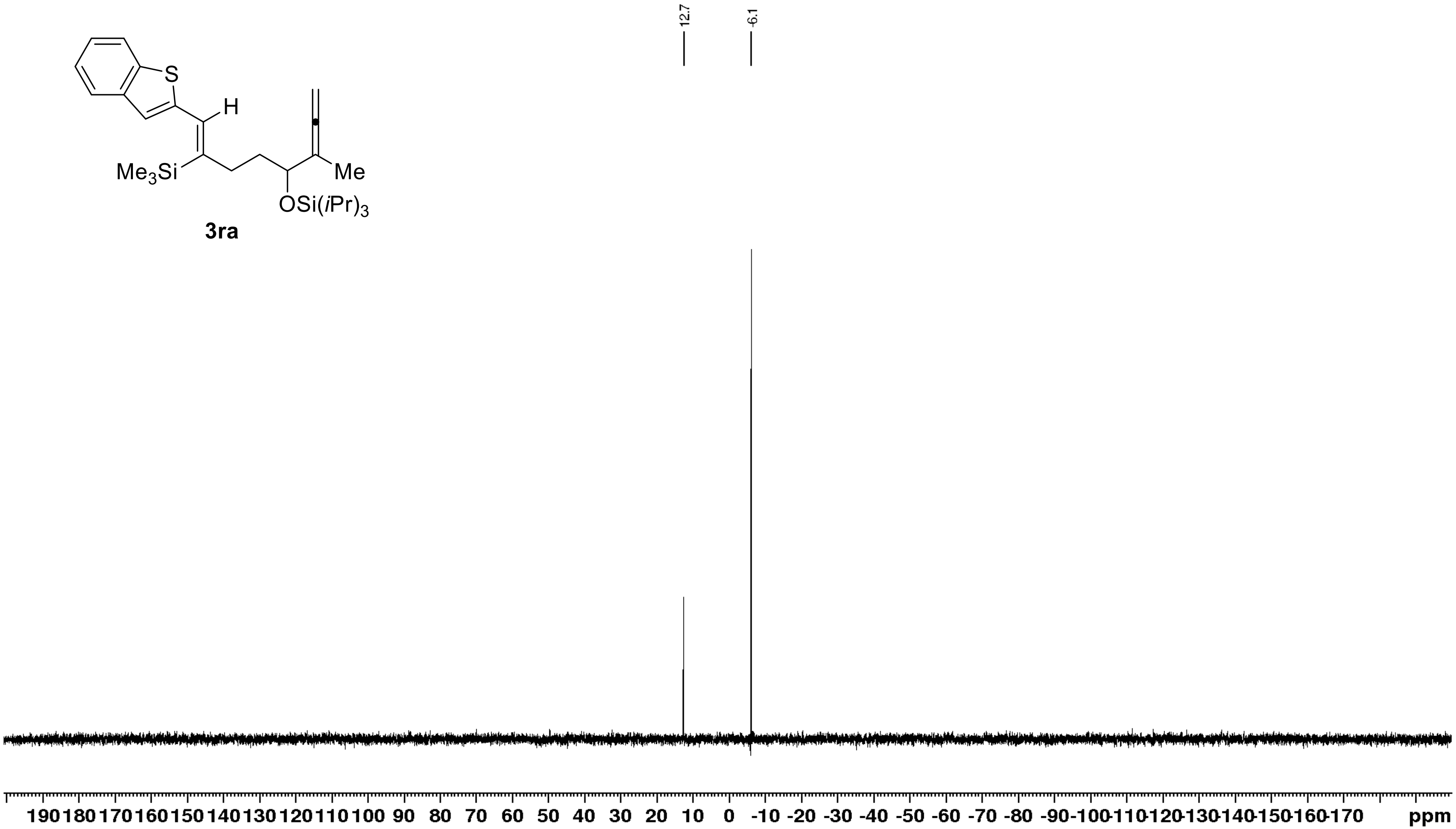

Figure S138. ^{1}H NMR spectrum (400 MHz, $CDCl_3$, 298 K) of **3sa**

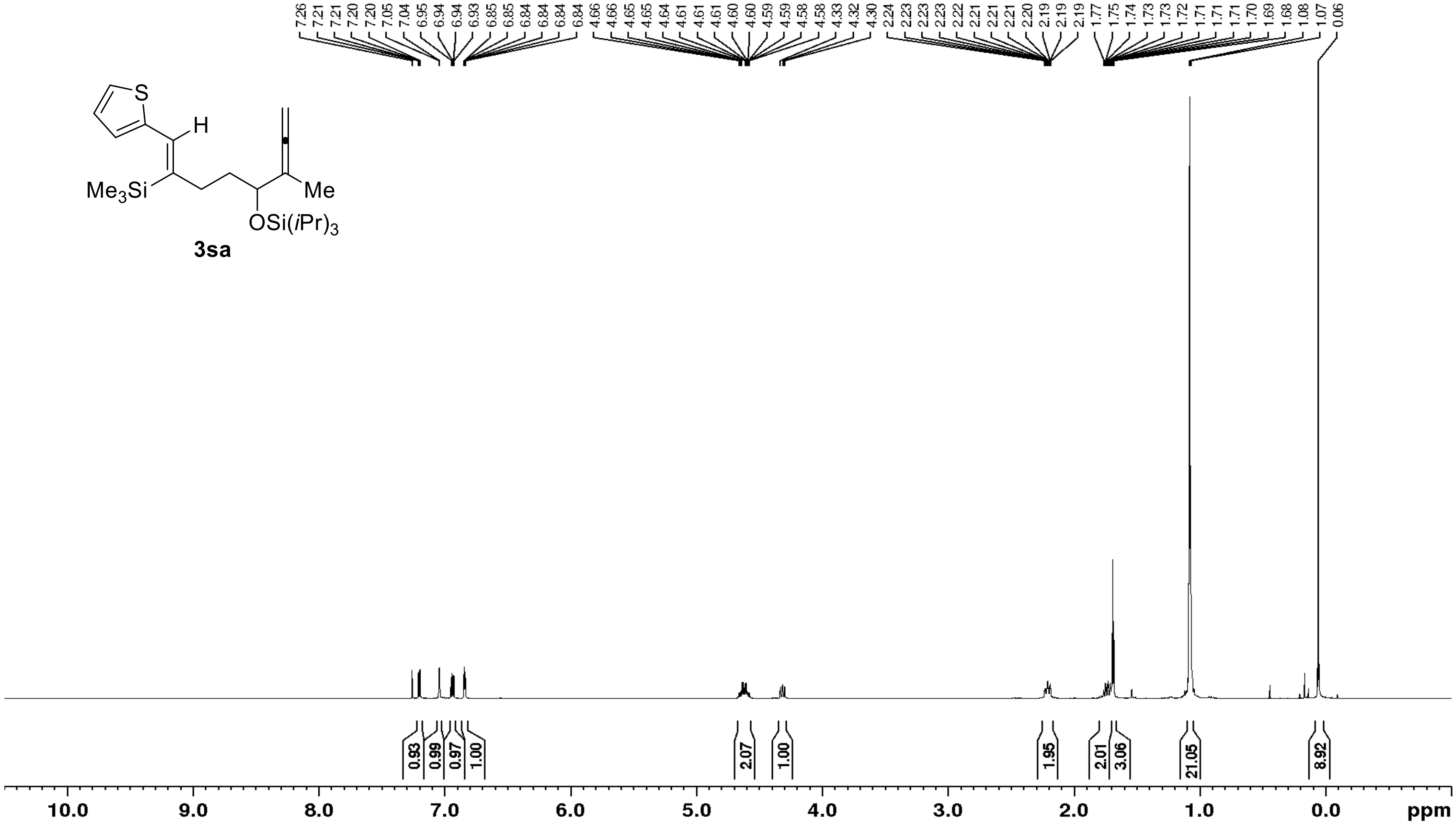

Figure S139. $^{13}C\{^1H\}$ NMR spectrum (101 MHz, $CDCl_3$, 298 K) of **3sa**

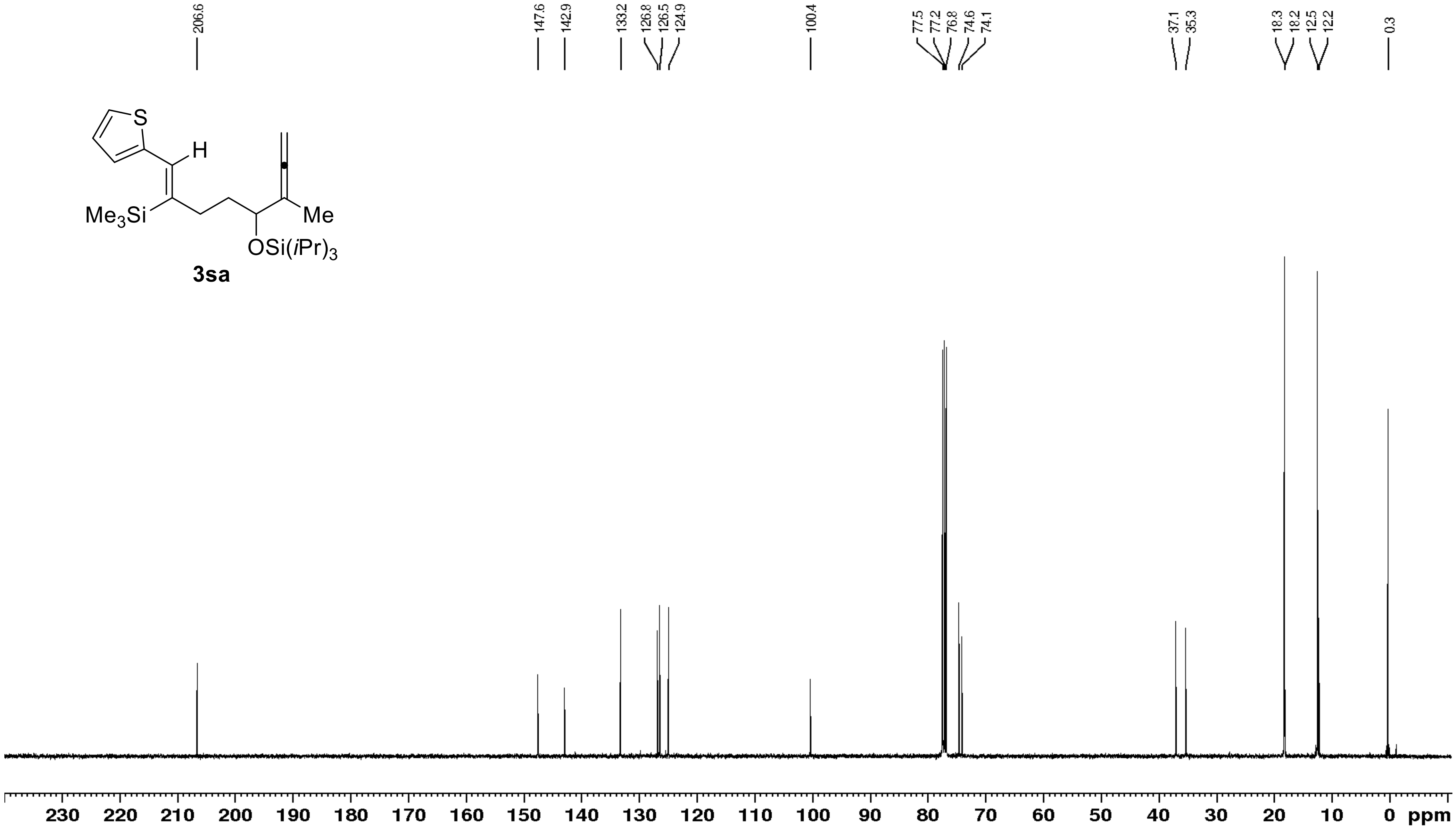

3sa

Figure S140. ^{29}Si DEPT NMR spectrum (79 MHz, $CDCl_3$, 298 K, optimized for J = 15.0 Hz) of **3sa**

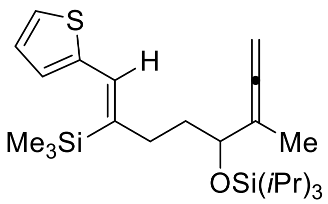

3sa

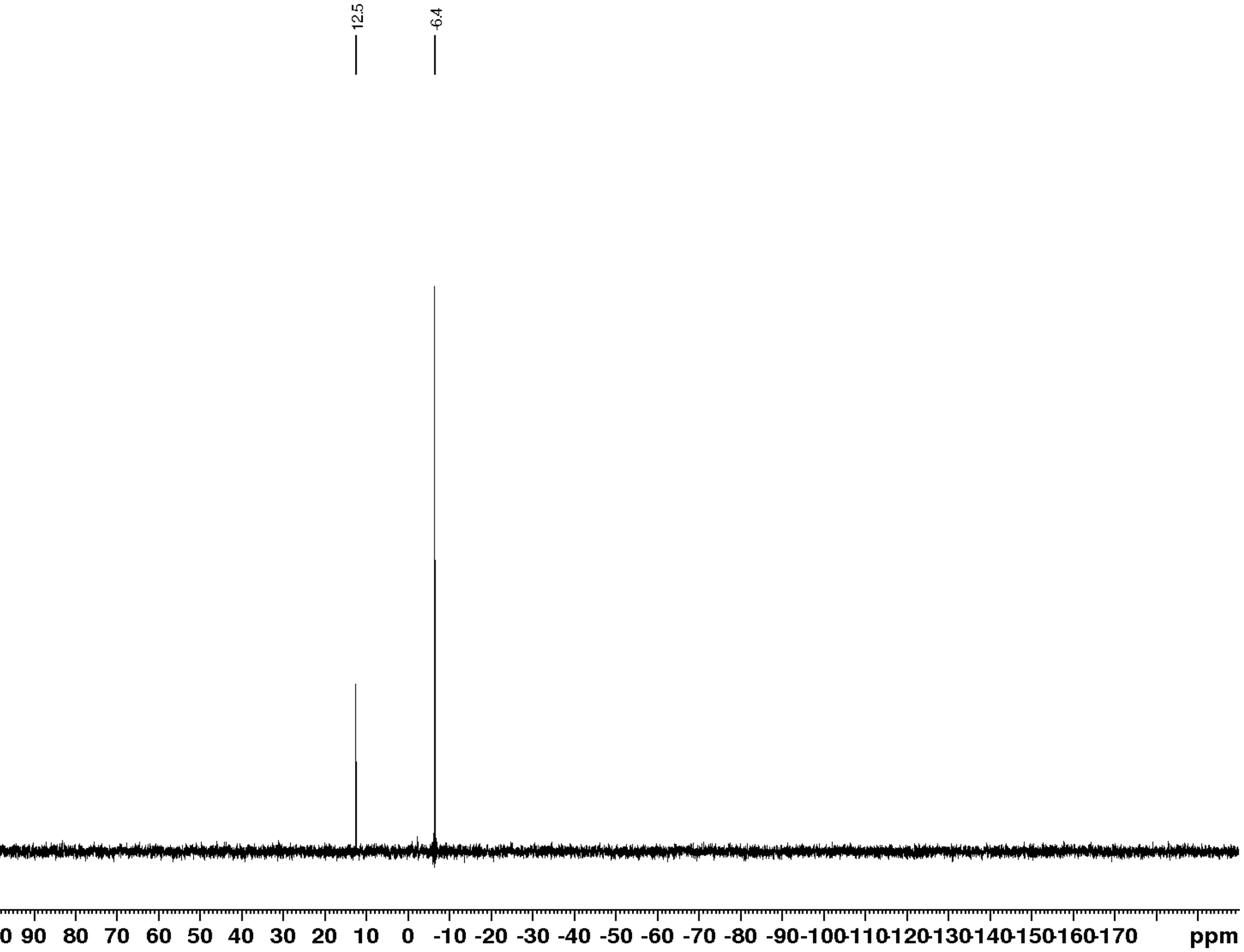

Figure S141. ^{1}H NMR spectrum (400 MHz, $CDCl_3$, 298 K) of **3ua**

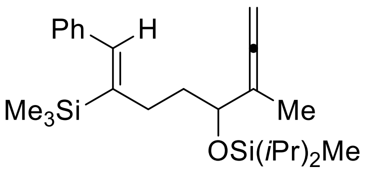

3ua

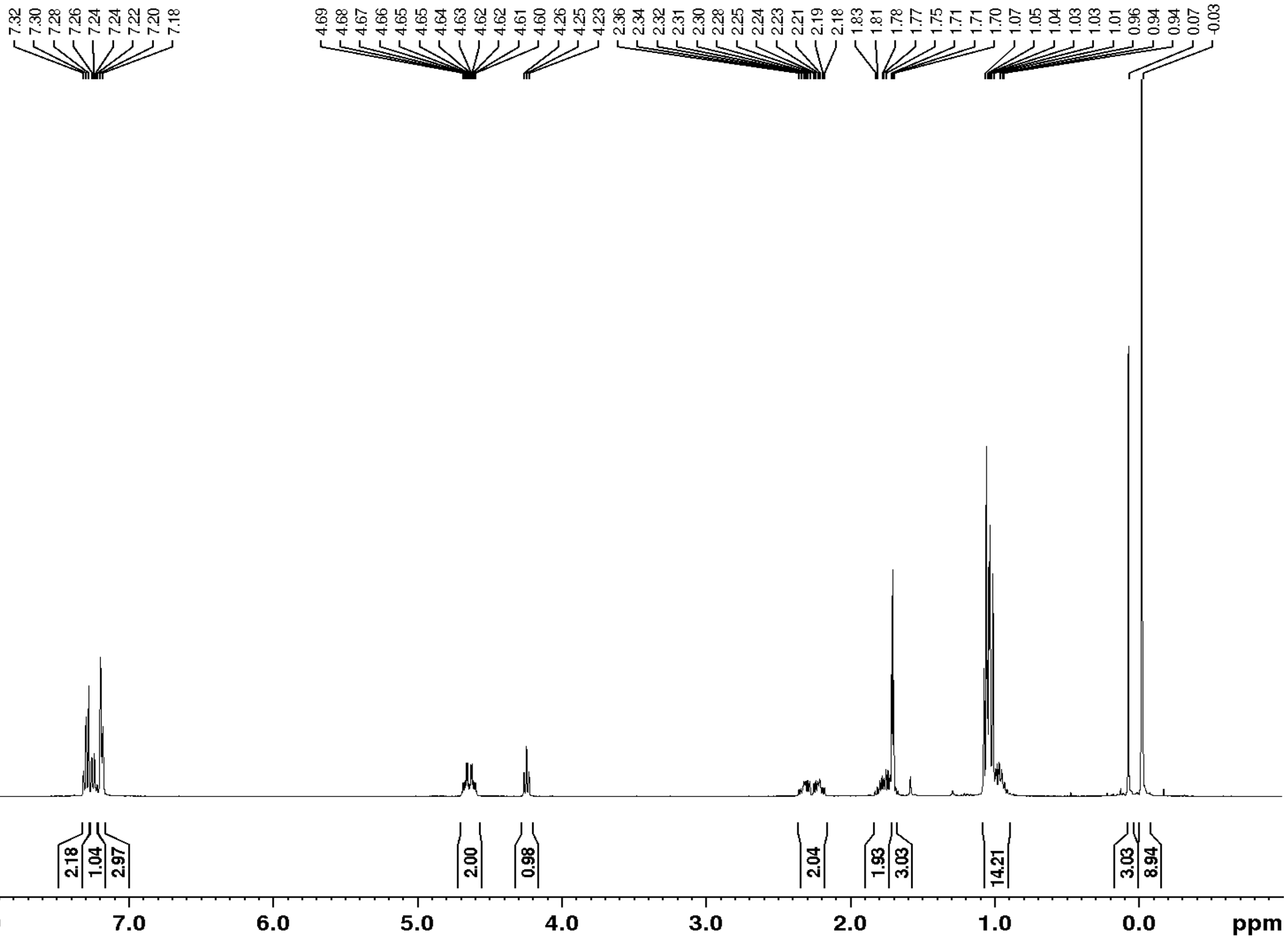

Figure S142. $^{13}C\{^{1}H\}$ NMR spectrum (101 MHz, $CDCl_3$, 298 K) of **3ua**

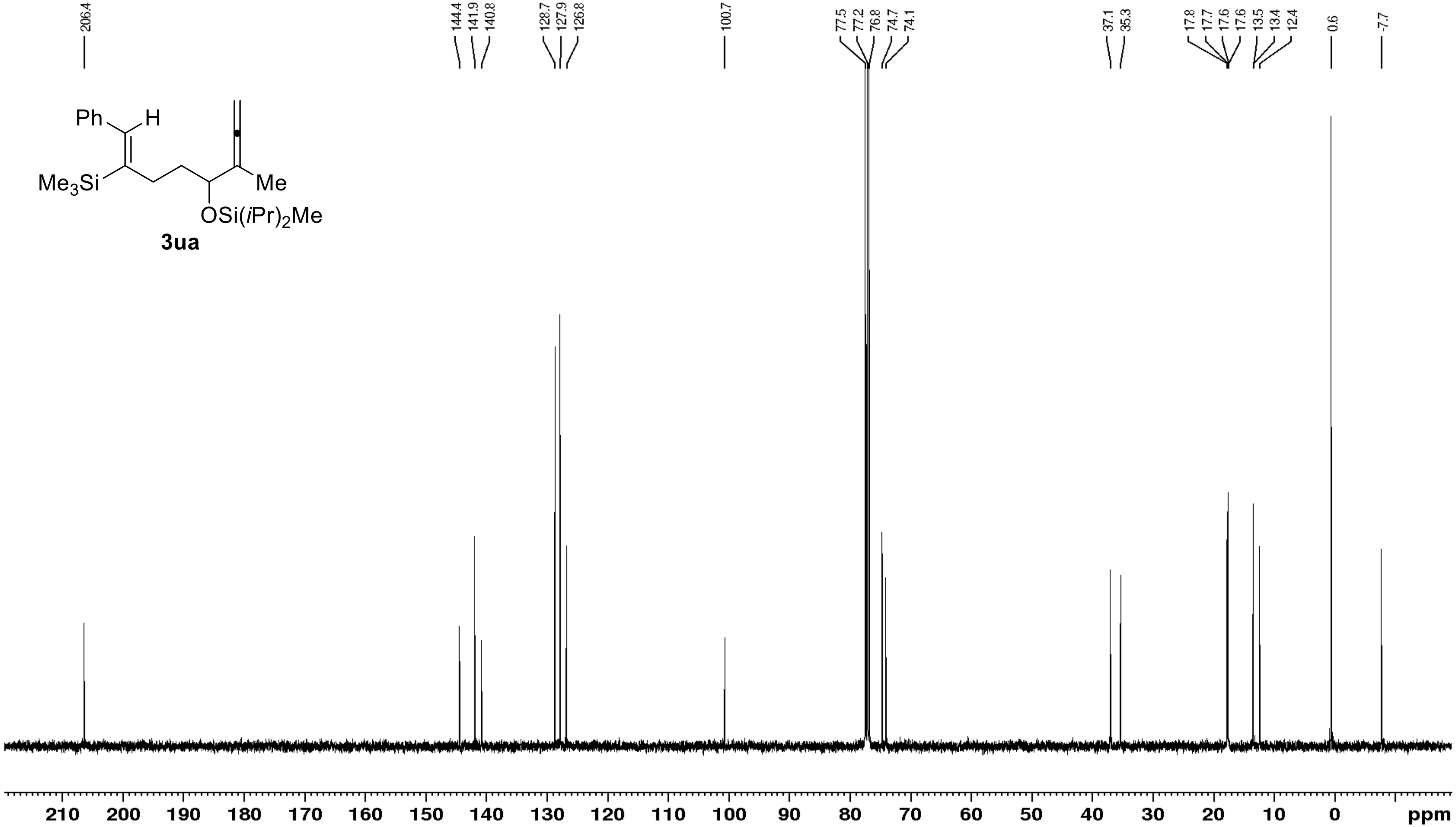

Figure S143. ^{29}Si DEPT NMR spectrum (79 MHz, $CDCl_3$, 298 K, optimized for J = 15.0 Hz) of **3ua**

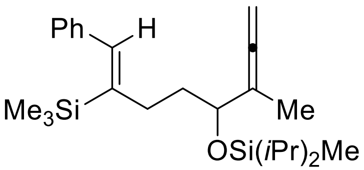

3ua

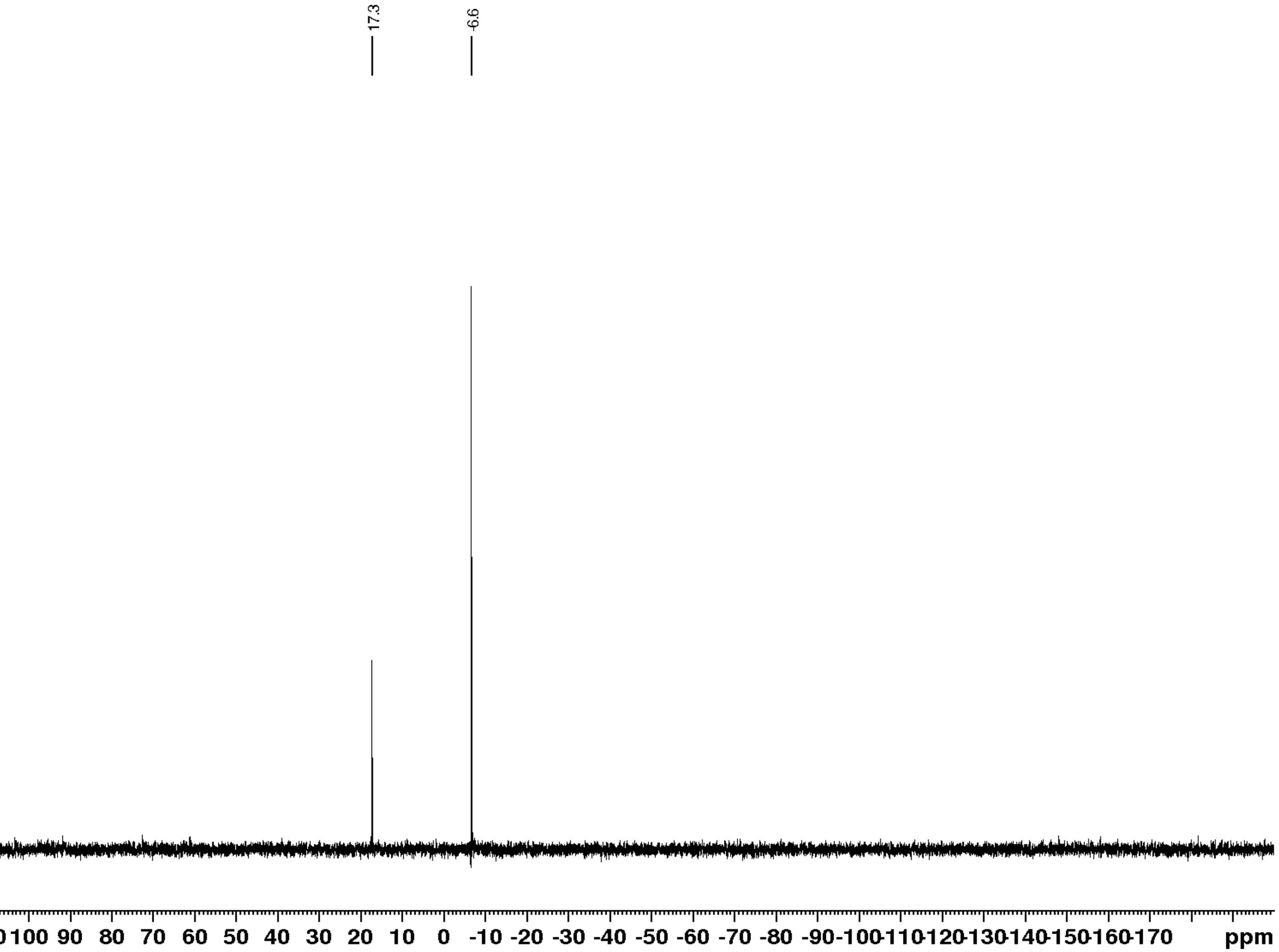

Figure S144. ^{1}H NMR spectrum (500 MHz, $CDCl_3$, 298 K) of **3va**

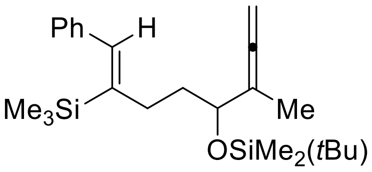

3va

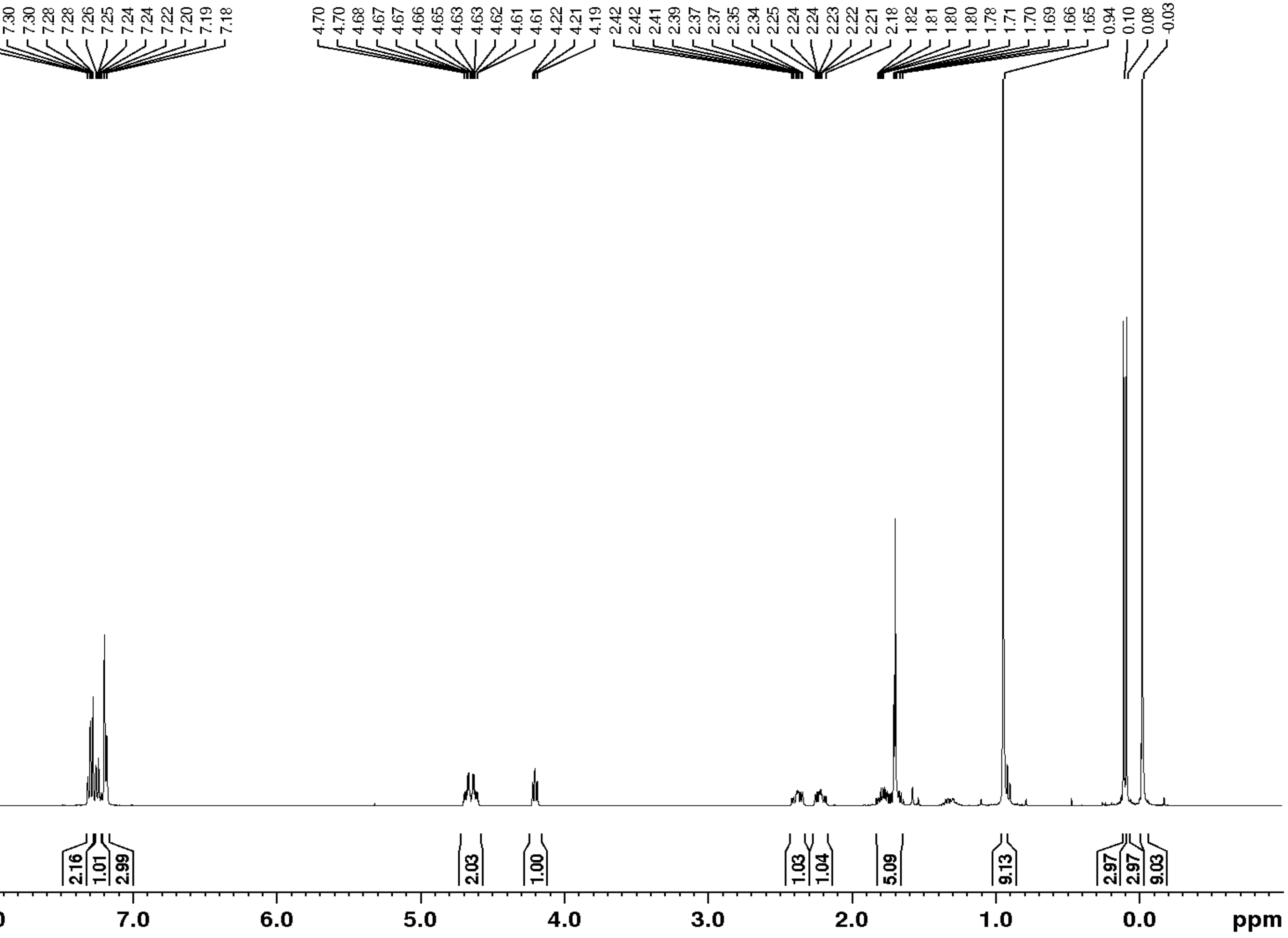

Figure S145. $^{13}C\{^1H\}$ NMR spectrum (126 MHz, $CDCl_3$, 298 K) of **3va**

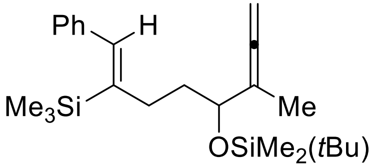

3va

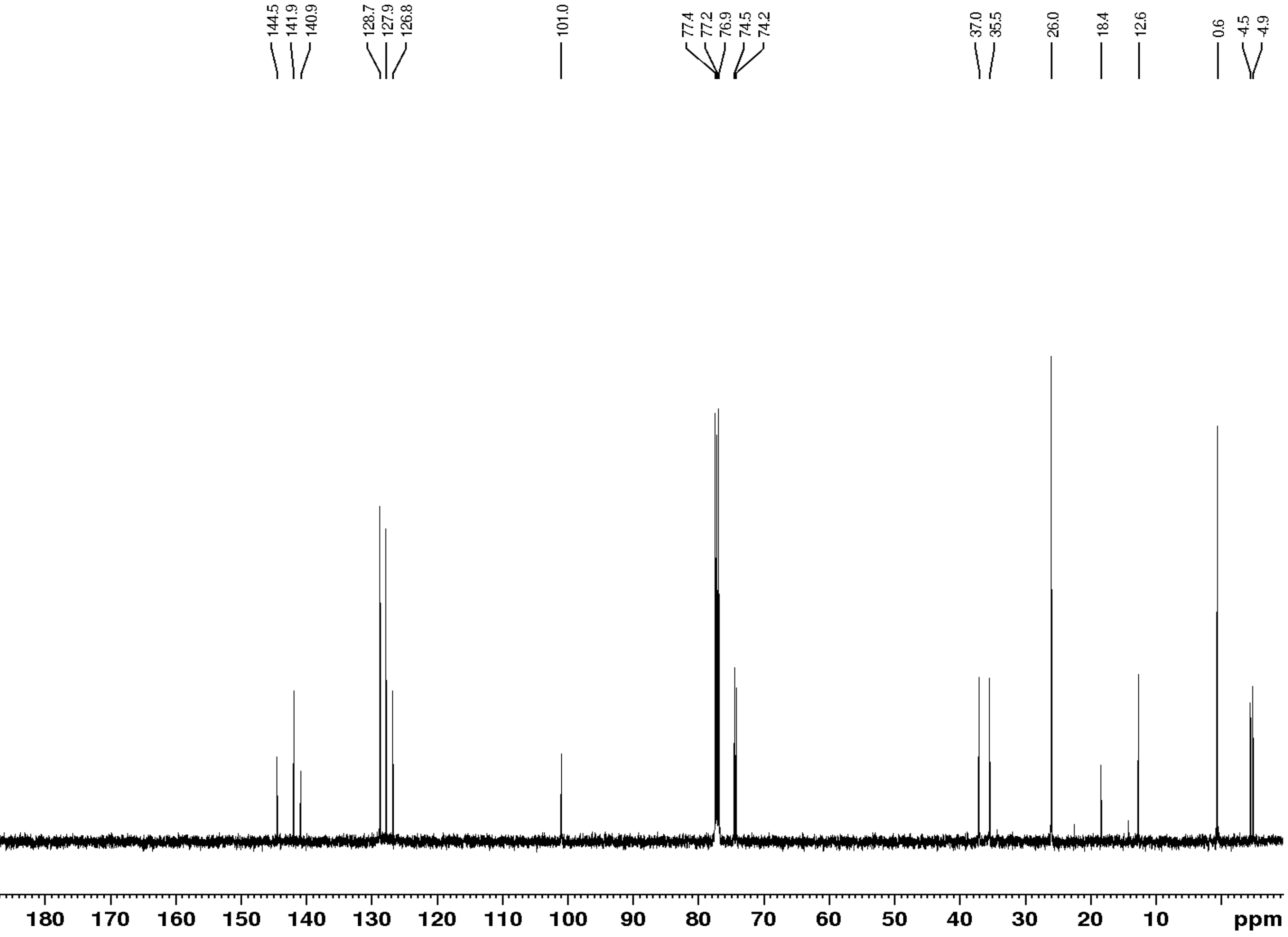

Figure S146. ^{29}Si DEPT NMR spectrum (99 MHz, $CDCl_3$, 298 K, optimized for *J* = 15.0 Hz) of **3va**

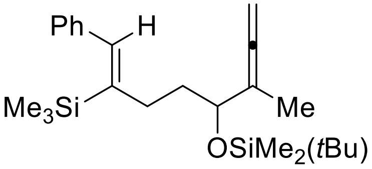

3va

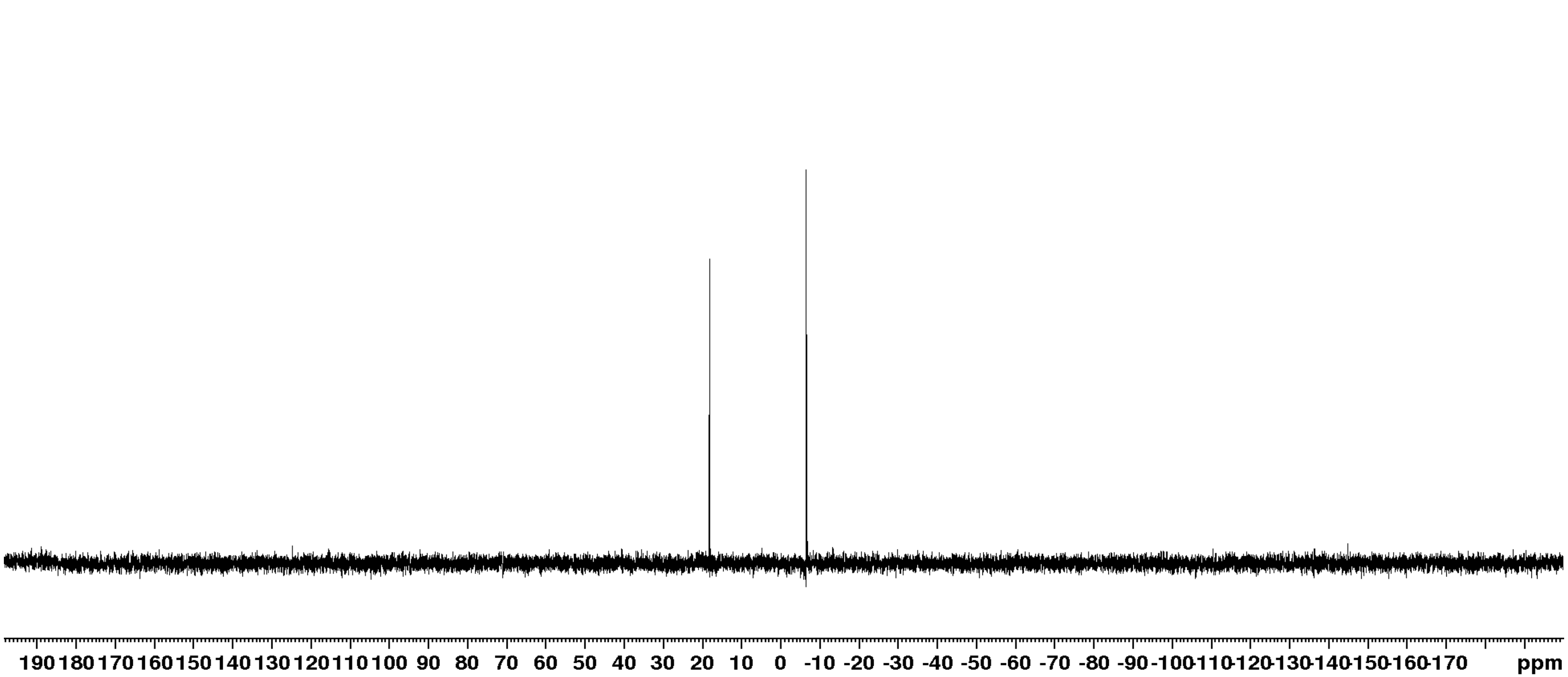

Figure S147. ^{1}H NMR spectrum (500 MHz, $CDCl_3$, 298 K) of **3wa**

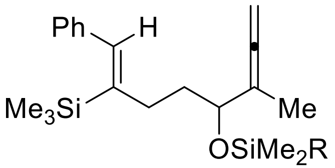

R = 2,3-dimethyl-2-butyl
3wa

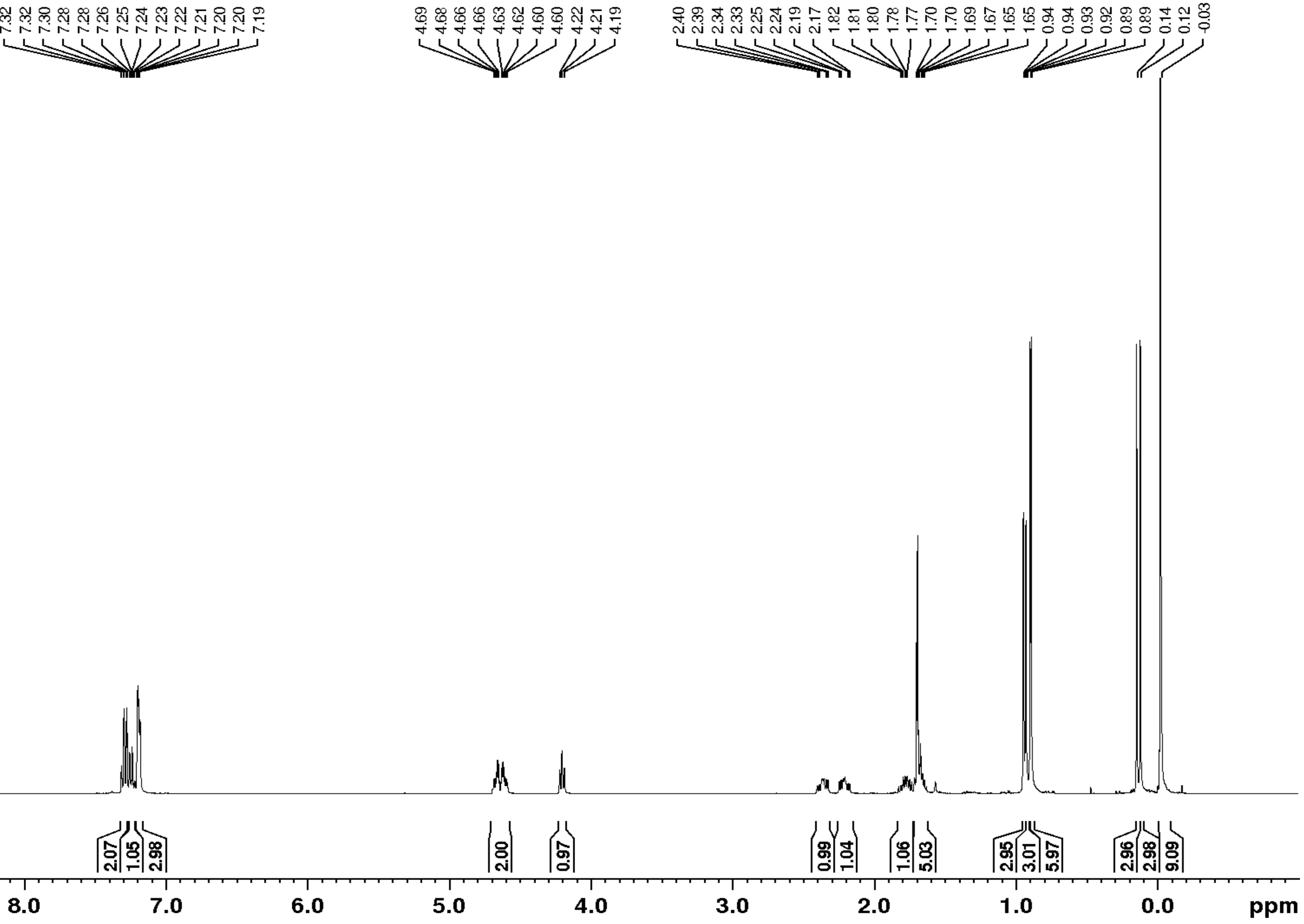

Figure S148. $^{13}C\{^{1}H\}$ NMR spectrum (126 MHz, $CDCl_3$, 298 K) of **3wa**

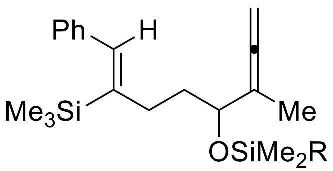

R = 2,3-dimethyl-2-butyl
3wa

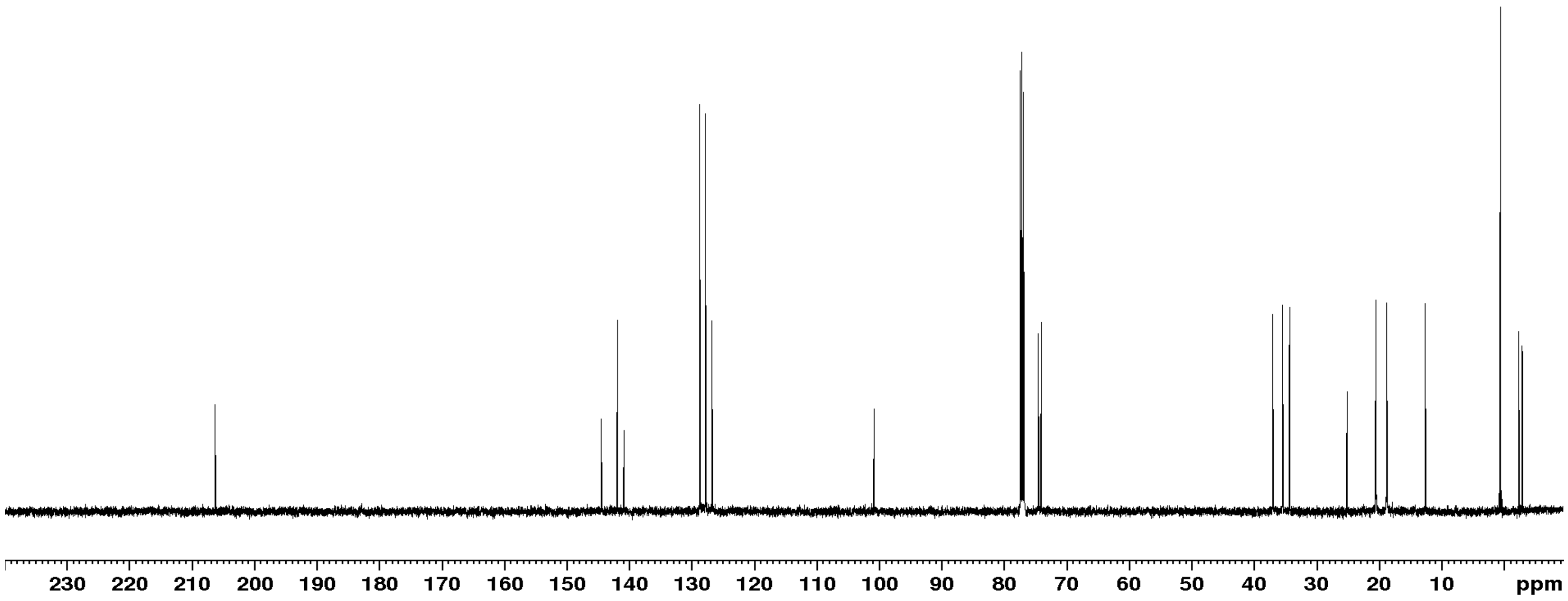

Figure S149. ^{29}Si DEPT NMR spectrum (99 MHz, $CDCl_3$, 298 K, optimized for J = 15.0 Hz) of **3wa**

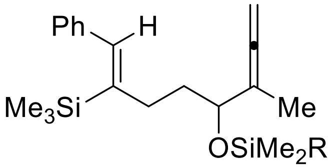

R = 2,3-dimethyl-2-butyl
3wa

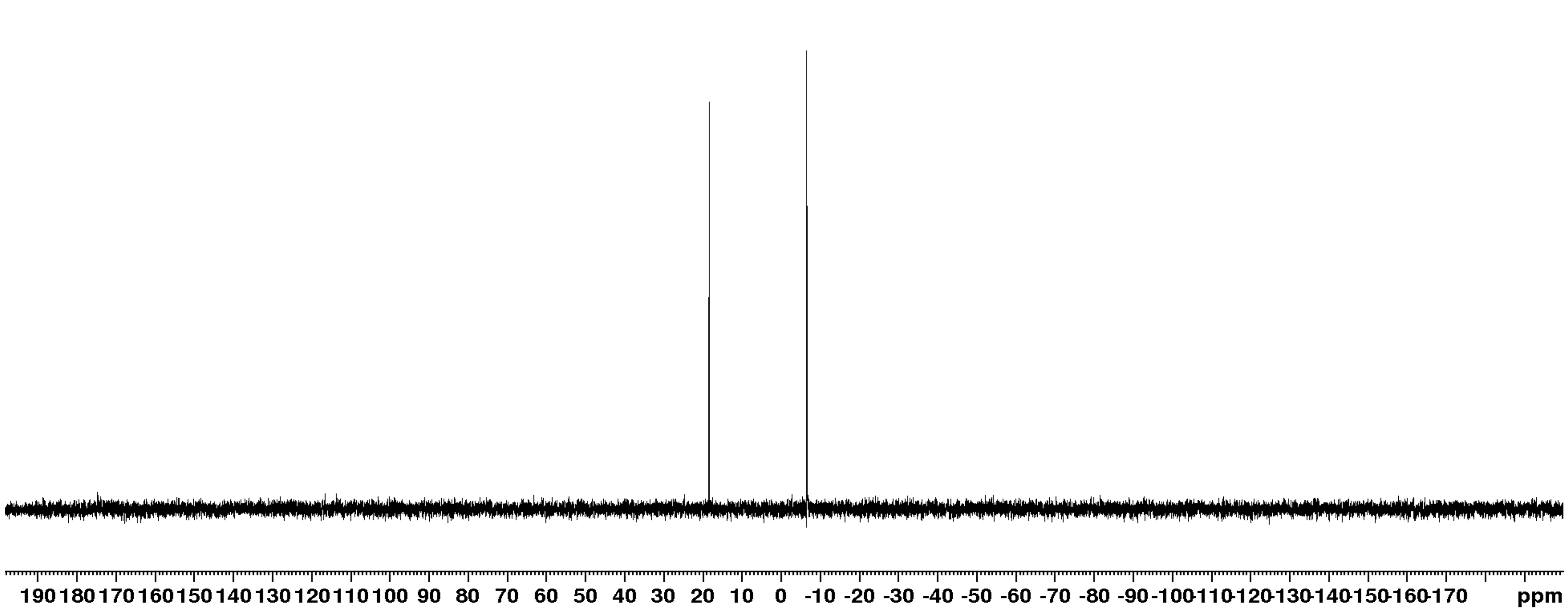

Figure S150. ^{1}H NMR spectrum (400 MHz, $CDCl_3$, 298 K) of **3ab**

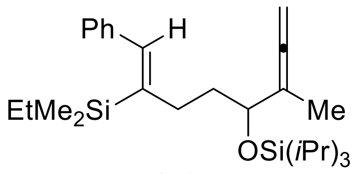

3ab

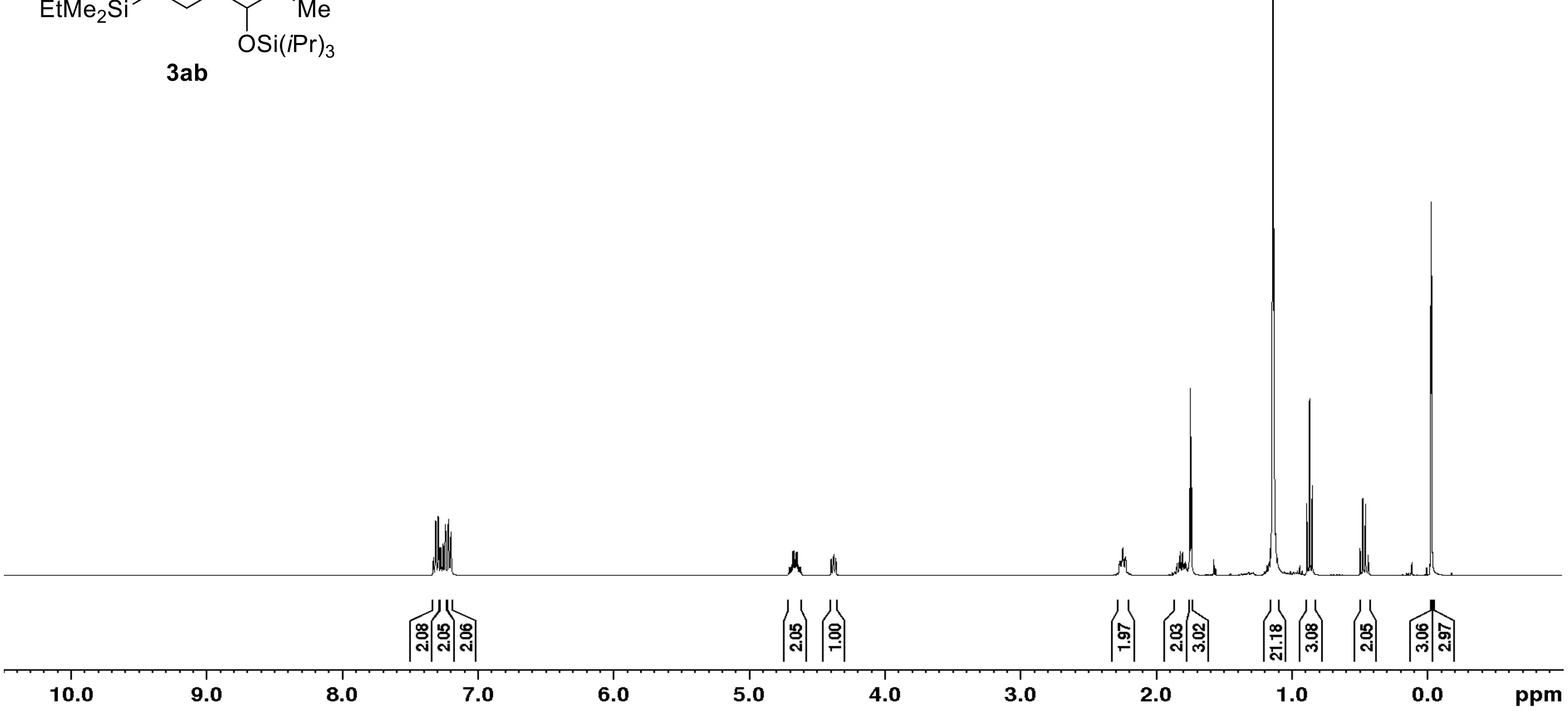

Figure S151. $^{13}C\{^1H\}$ NMR spectrum (101 MHz, $CDCl_3$, 298 K) of **3ab**

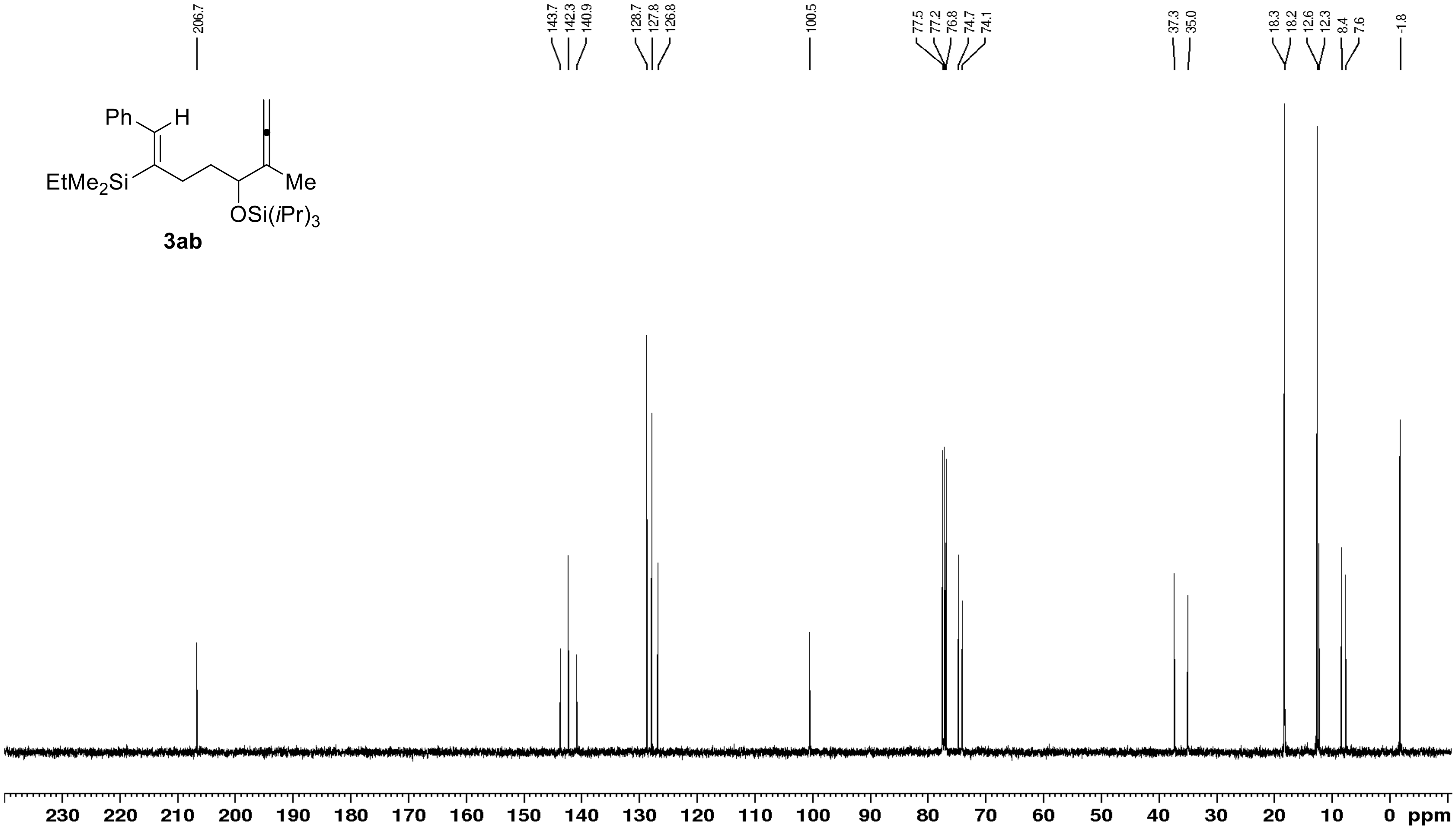

Figure S152. ^{29}Si DEPT NMR spectrum (79 MHz, $CDCl_3$, 298 K, optimized for *J* = 15.0 Hz) of **3ab**

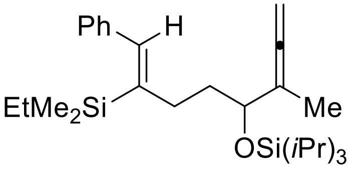

3ab

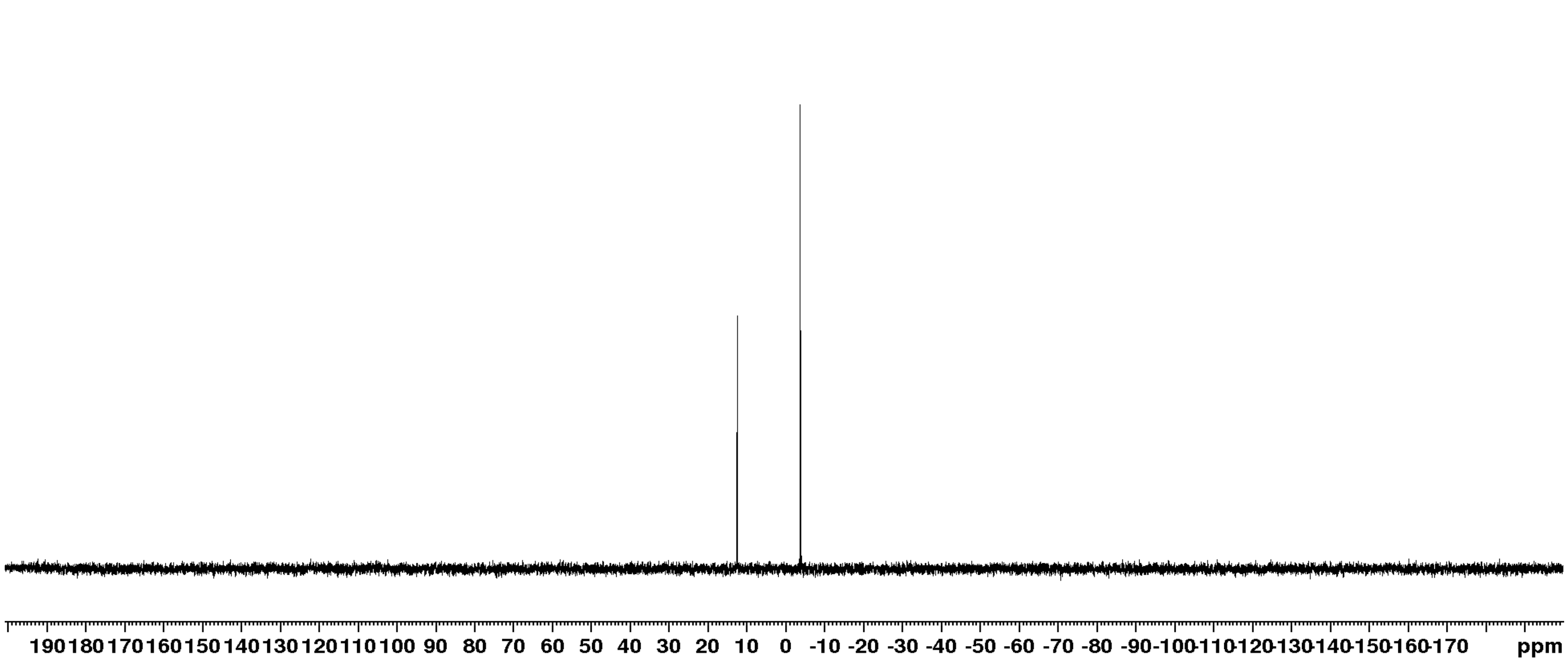

Figure S153. ^{1}H NMR spectrum (400 MHz, $CDCl_3$, 298 K) of **3ac**

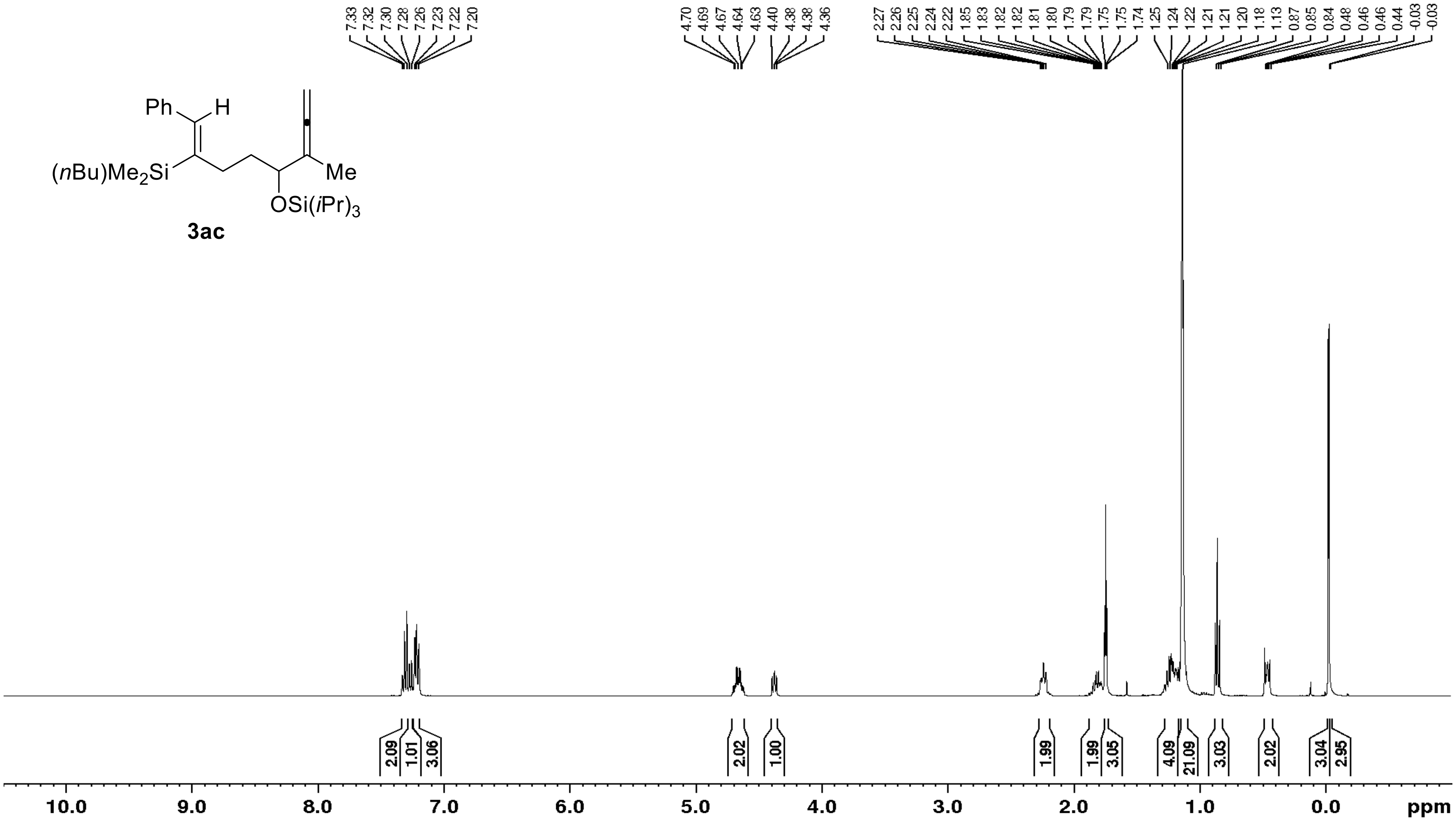

Figure S154. $^{13}C\{^1H\}$ NMR spectrum (101 MHz, $CDCl_3$, 298 K) of **3ac**

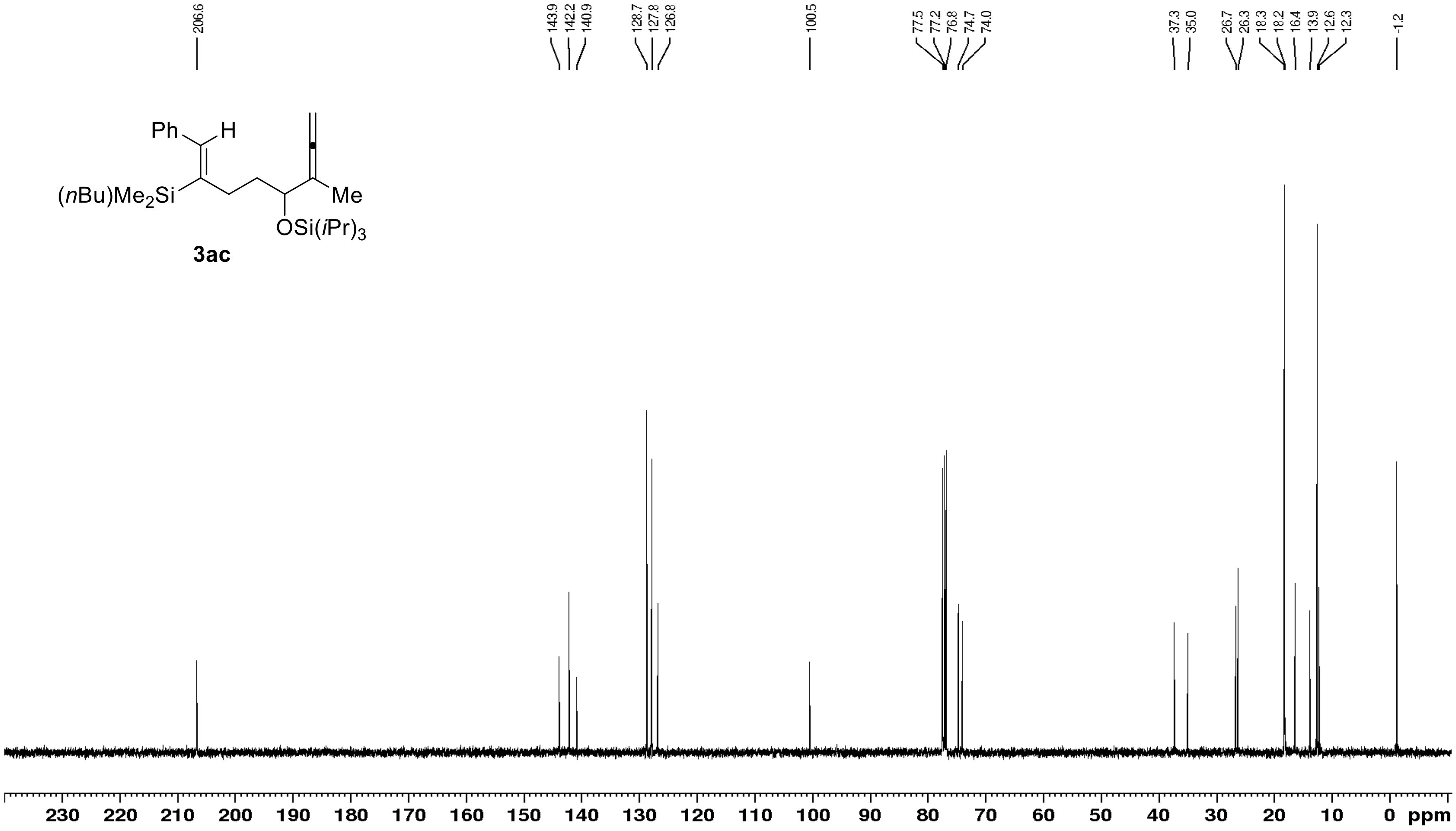

Figure S155. ^{29}Si DEPT NMR spectrum (79 MHz, $CDCl_3$, 298 K, optimized for J = 15.0 Hz) of **3ac**

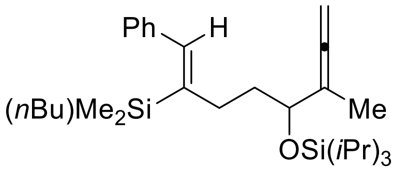

3ac

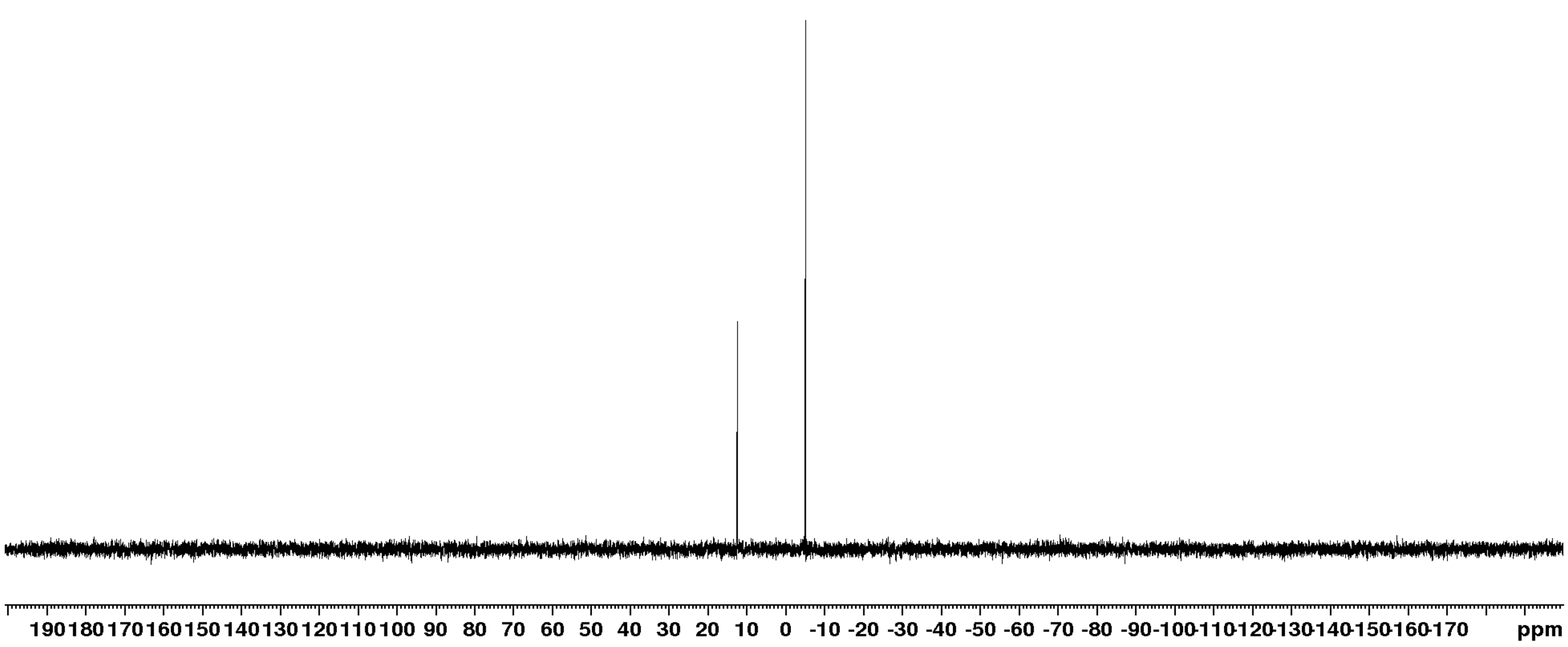

Figure S156. ^{1}H NMR spectrum (400 MHz, $CDCl_3$, 298 K) of **3ad**

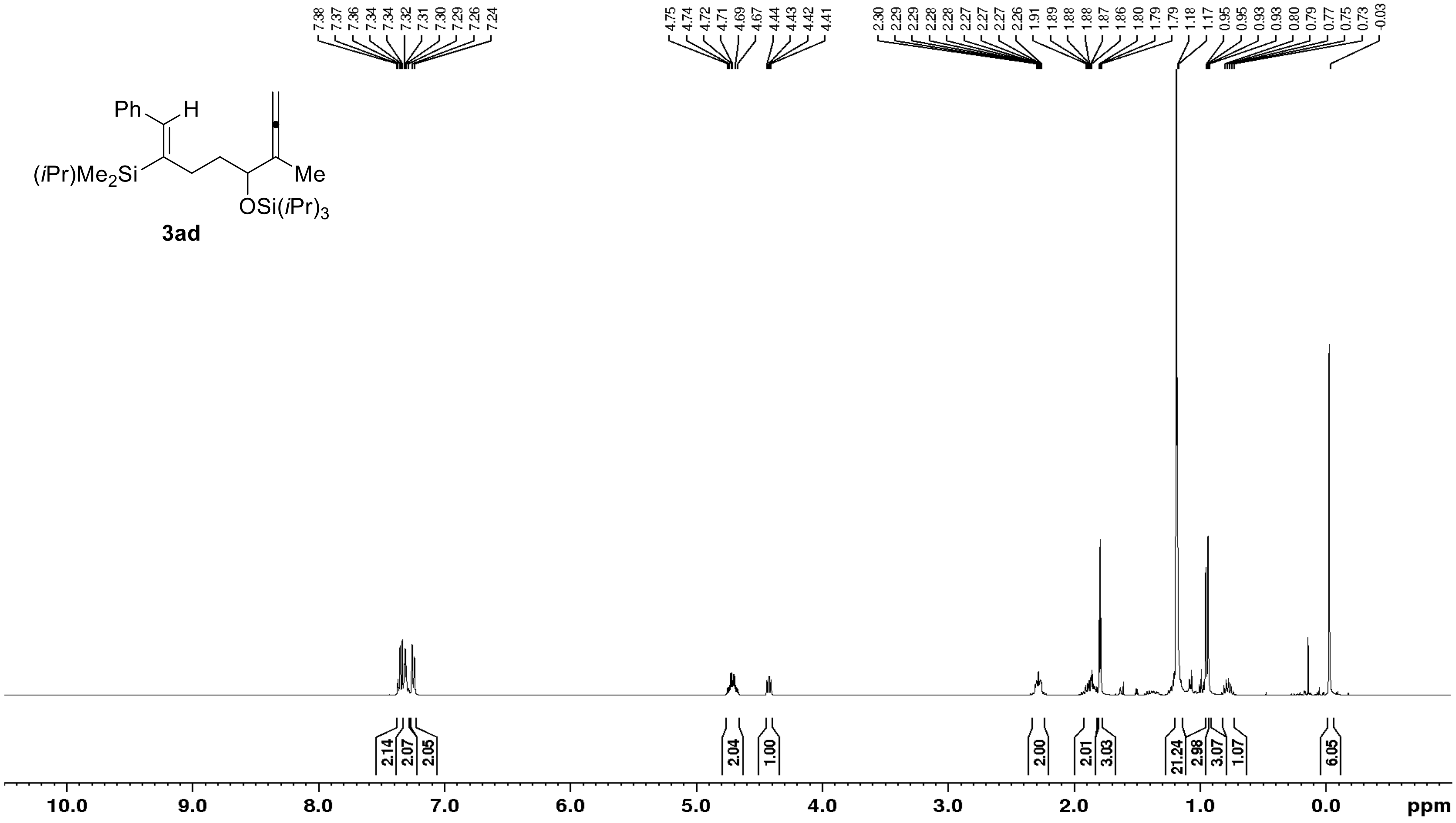

3ad

Figure S157. $^{13}C\{^{1}H\}$ NMR spectrum (101 MHz, $CDCl_3$, 298 K) of **3ad**

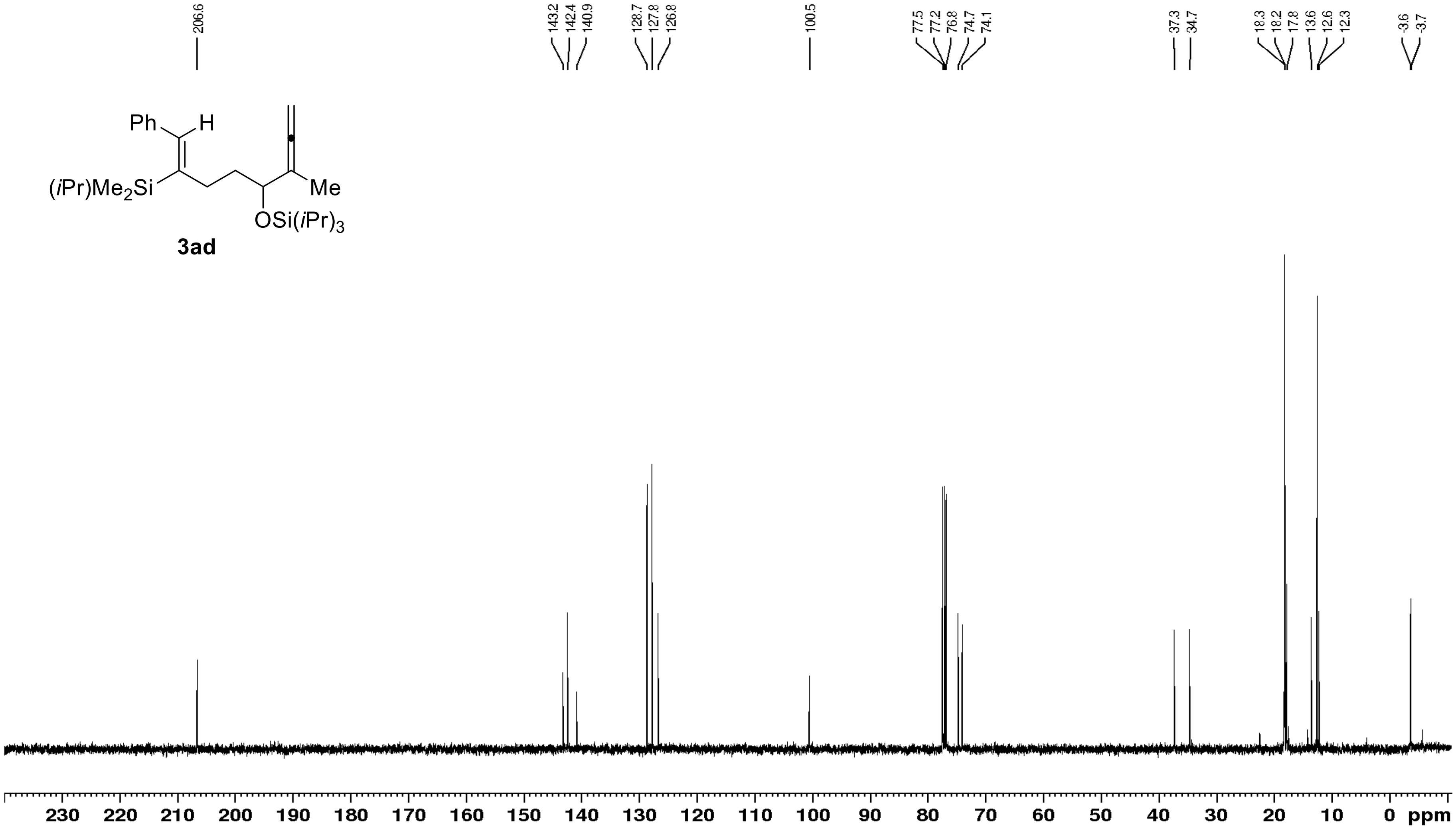

Figure S158. ^{29}Si DEPT NMR spectrum (79 MHz, $CDCl_3$, 298 K, optimized for *J* = 15.0 Hz) of **3ad**

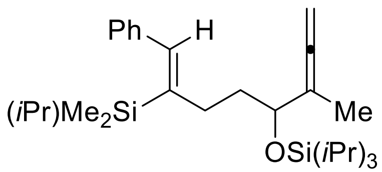

3ad

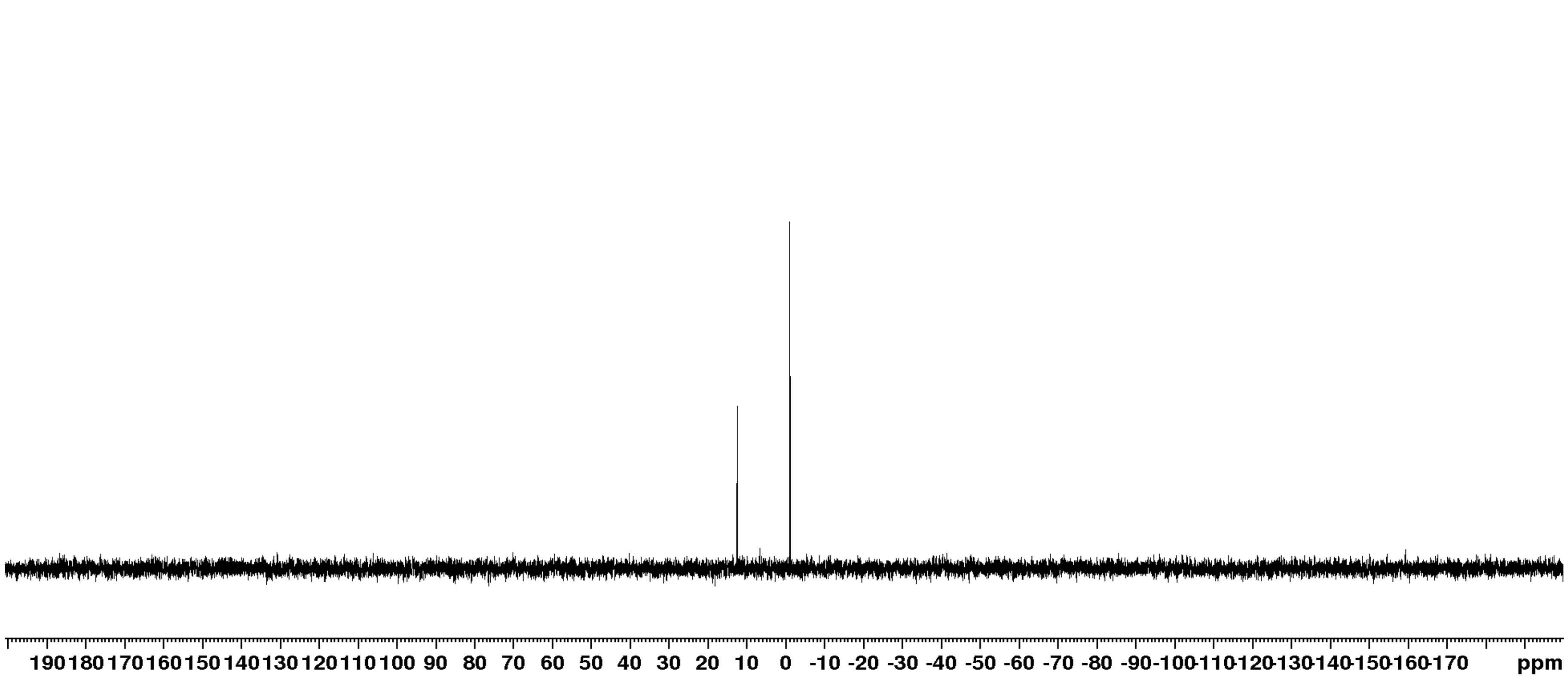

Figure S159. ^{1}H NMR spectrum (400 MHz, $CDCl_3$, 298 K) of **3ag**

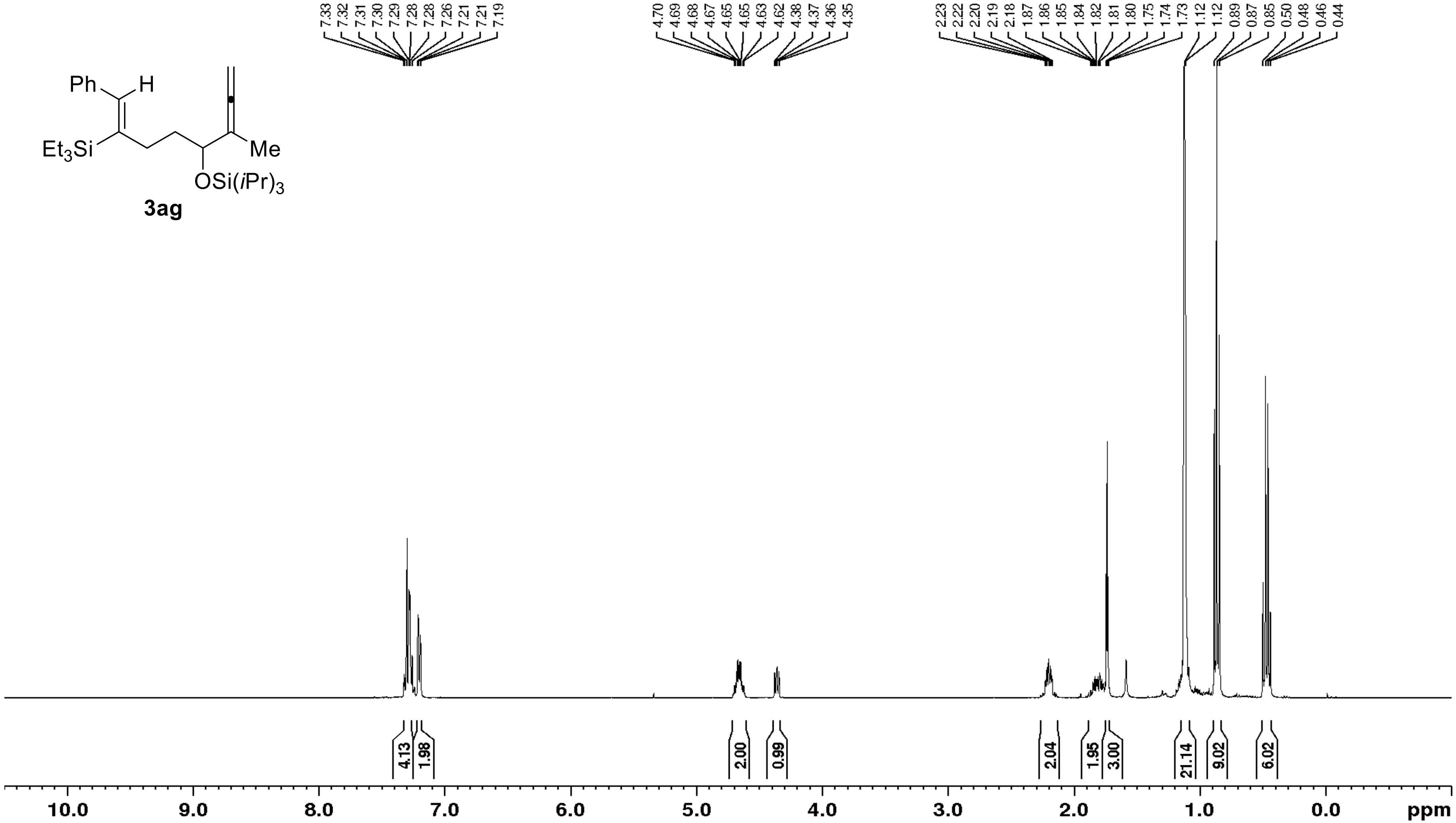

Figure S160. $^{13}C\{^1H\}$ NMR spectrum (101 MHz, $CDCl_3$, 298 K) of **3ag**

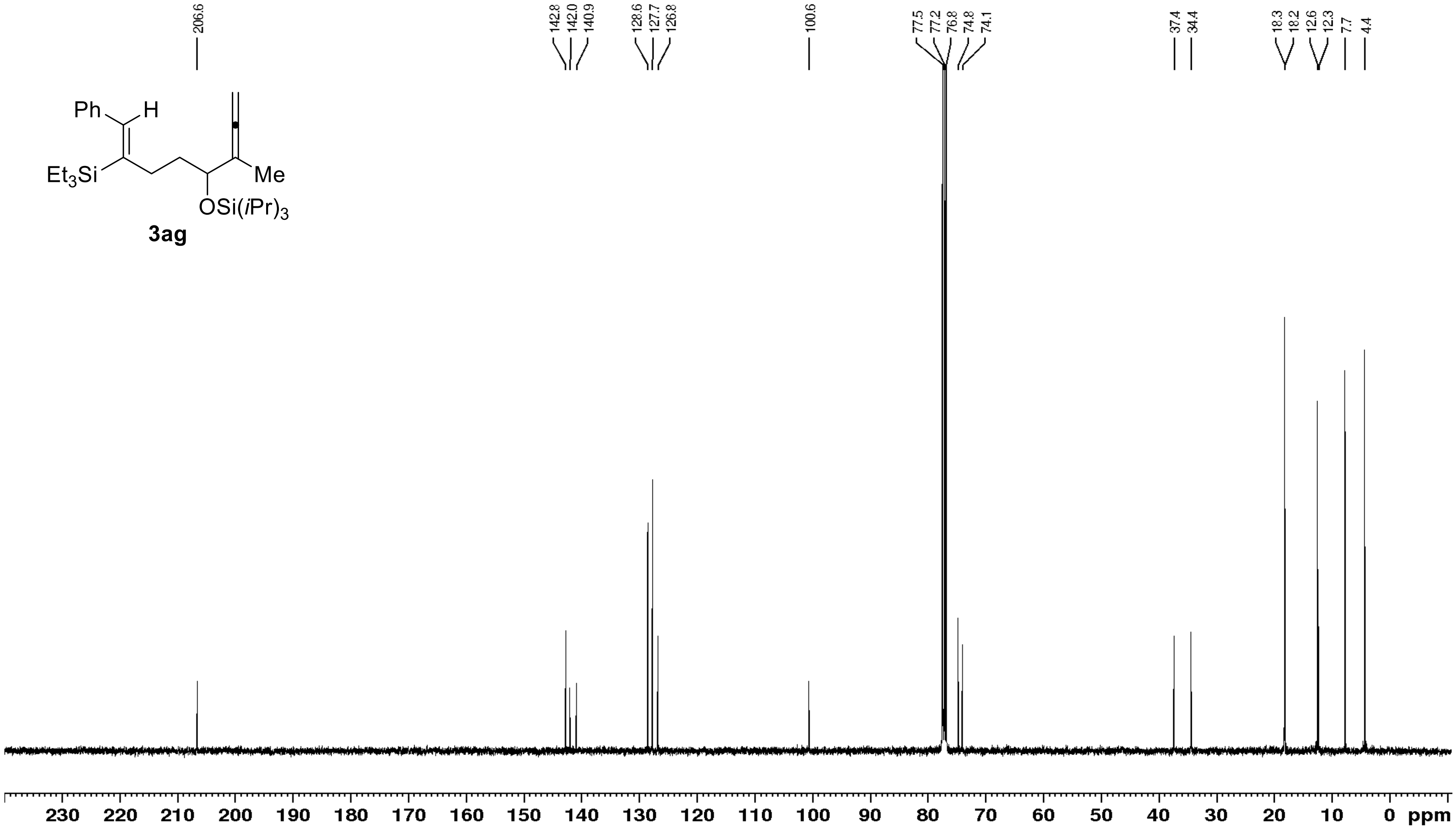

Figure S161. ^{29}Si DEPT NMR spectrum (79 MHz, $CDCl_3$, 298 K, optimized for J = 15.0 Hz) of **3ag**

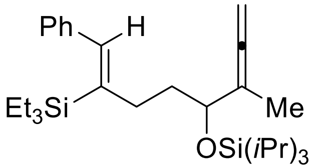

3ag

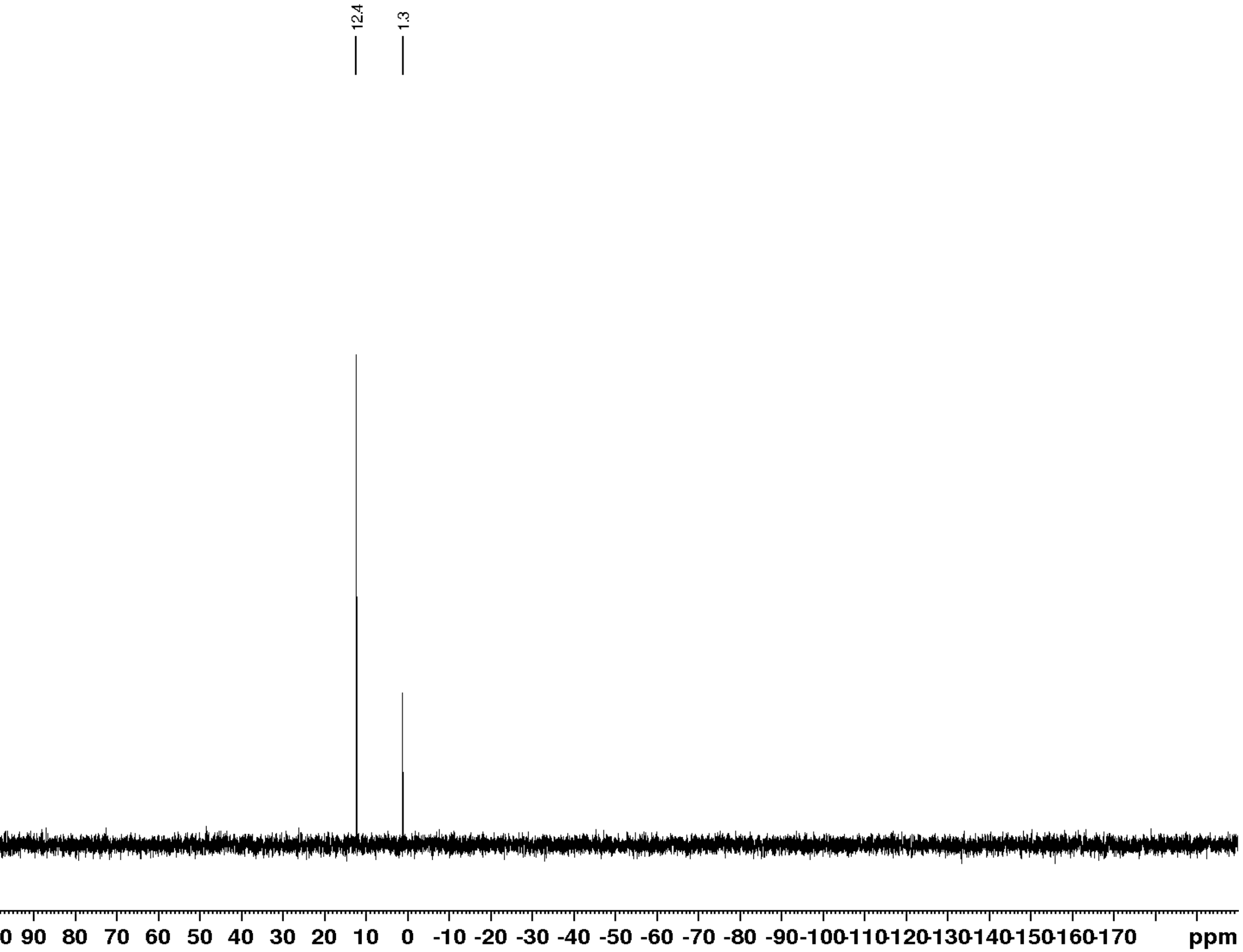

Figure S162. ^{1}H NMR spectrum (400 MHz, $CDCl_3$, 298 K) of **3ah**

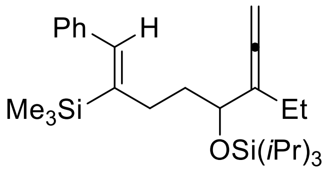

3ah

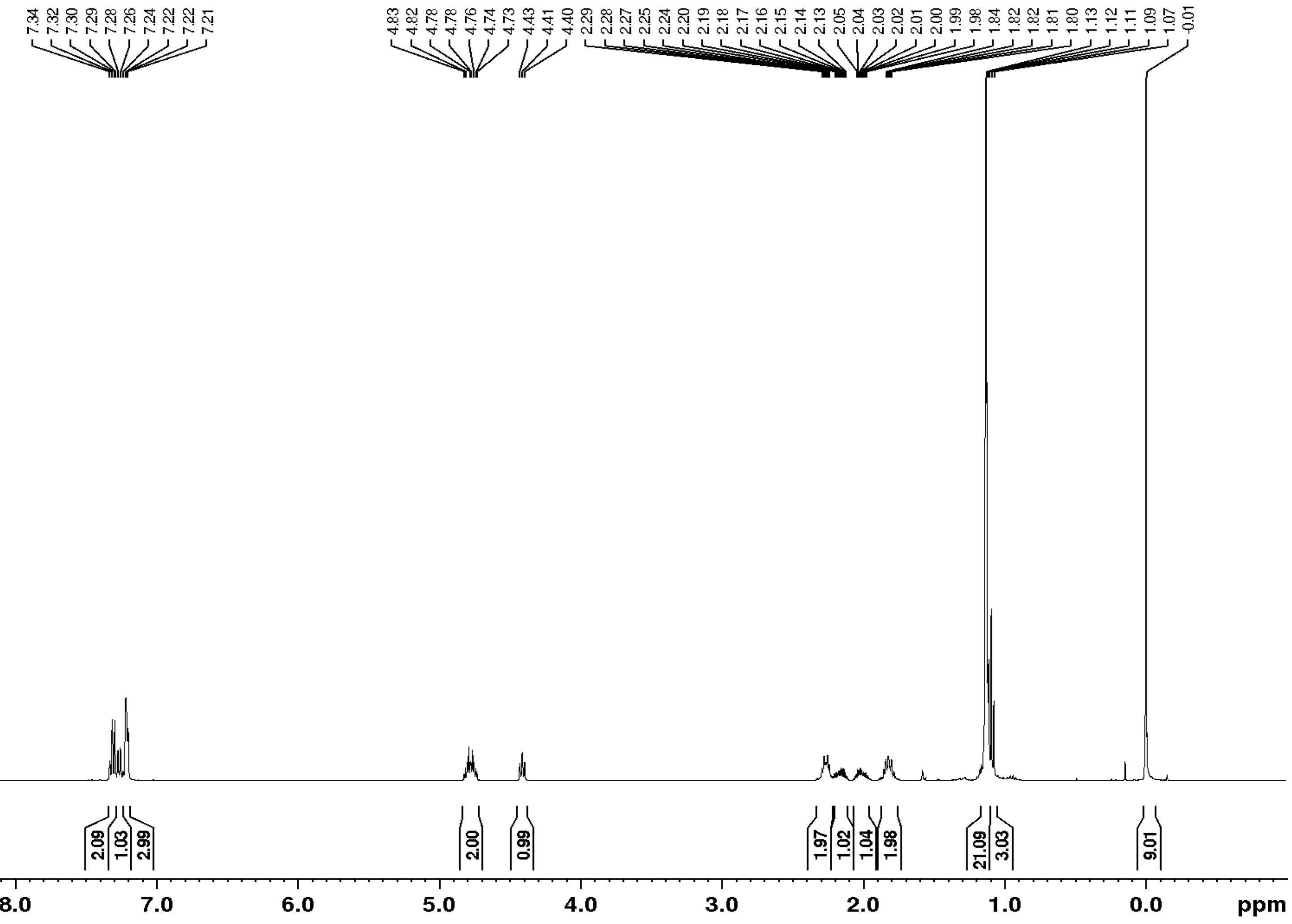

Figure S163. $^{13}C\{^{1}H\}$ NMR spectrum (101 MHz, $CDCl_3$, 298 K) of **3ah**

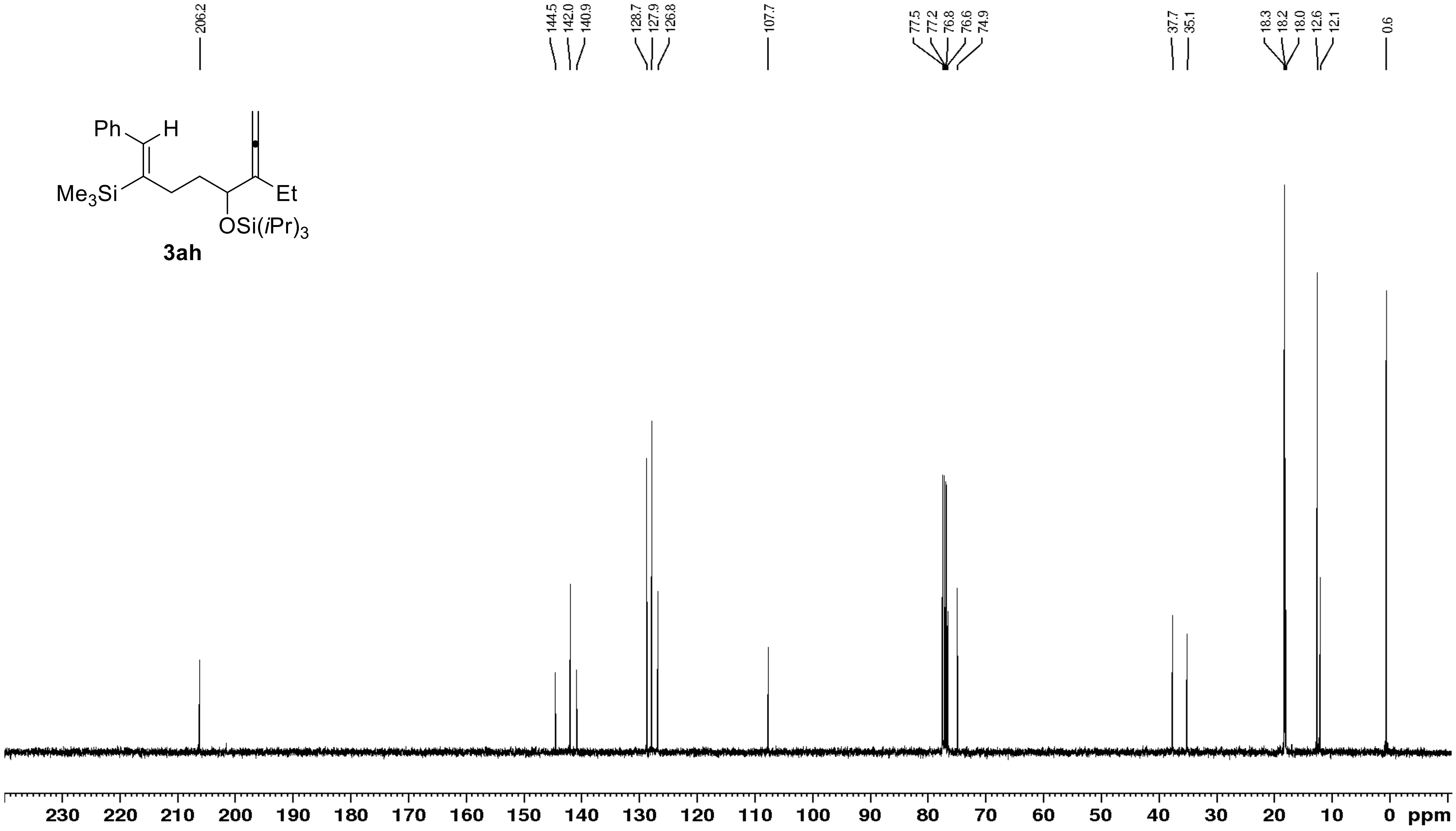

Figure S164. ^{29}Si DEPT NMR spectrum (79 MHz, $CDCl_3$, 298 K, optimized for J = 15.0 Hz) of **3ah**

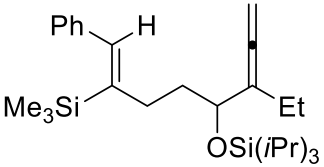

3ah

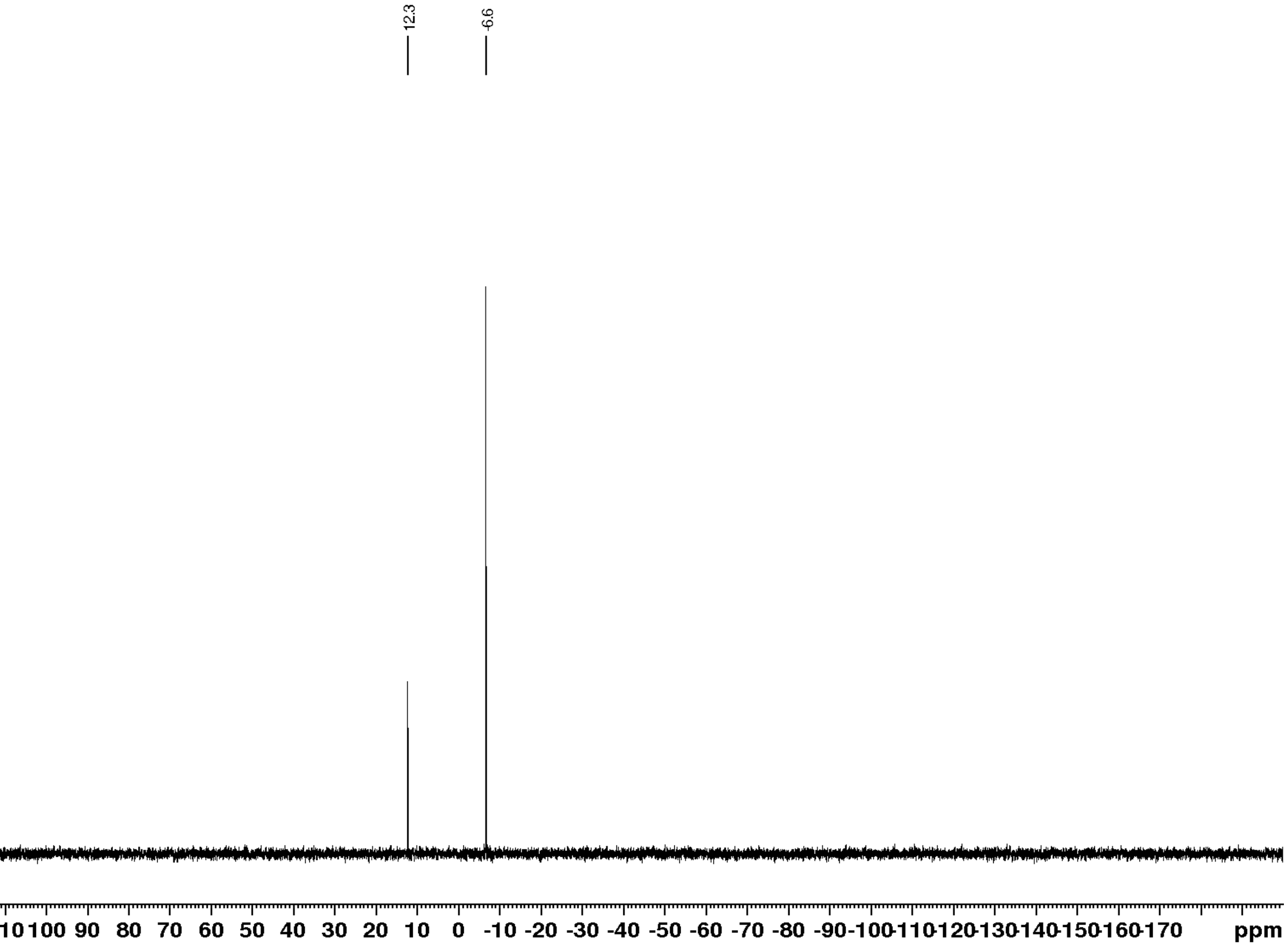

Figure S165. ^{1}H NMR spectrum (400 MHz, $CDCl_3$, 298 K) of **3ai**

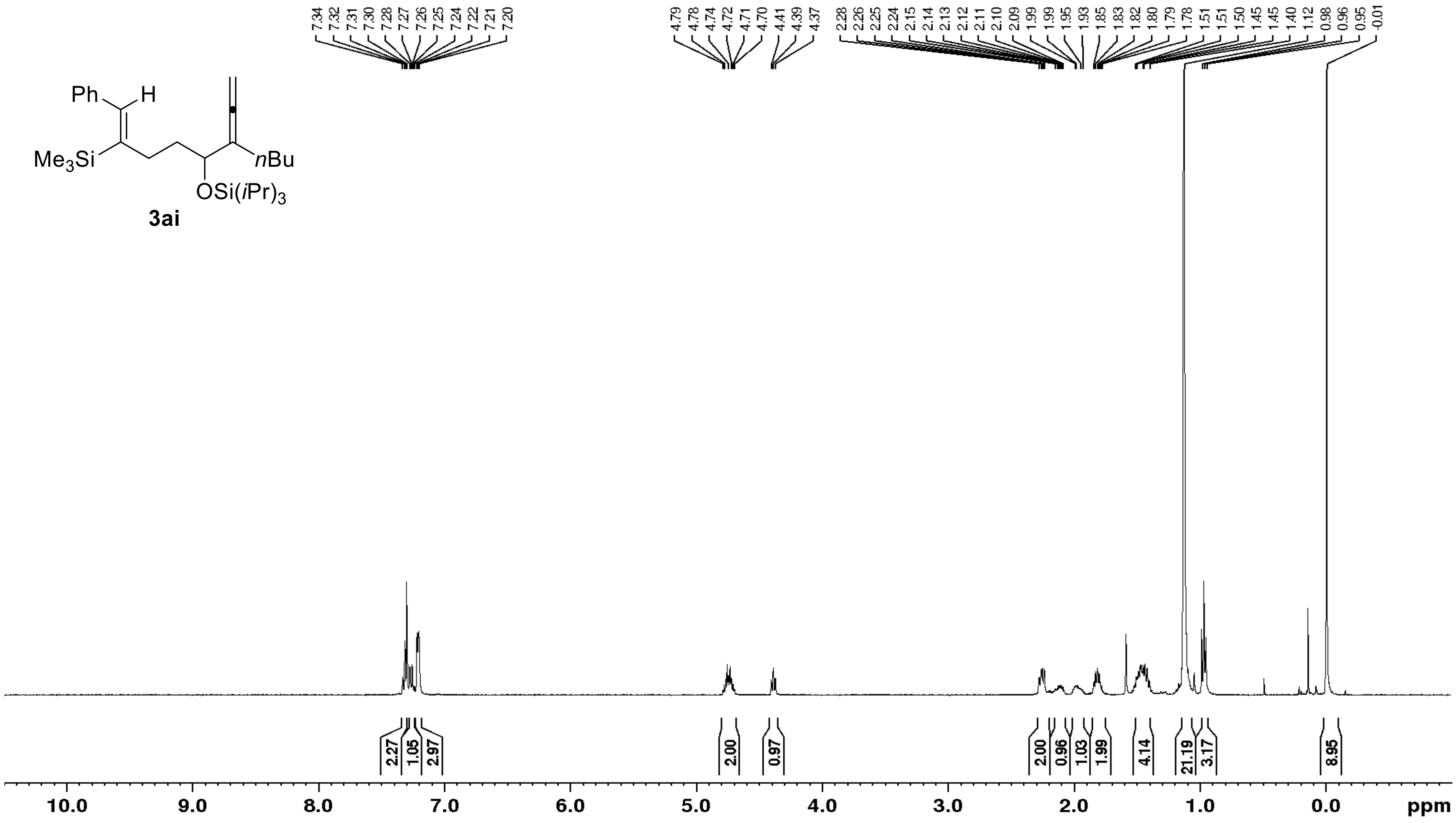

Figure S166. $^{13}C\{^1H\}$ NMR spectrum (101 MHz, $CDCl_3$, 298 K) of **3ai**

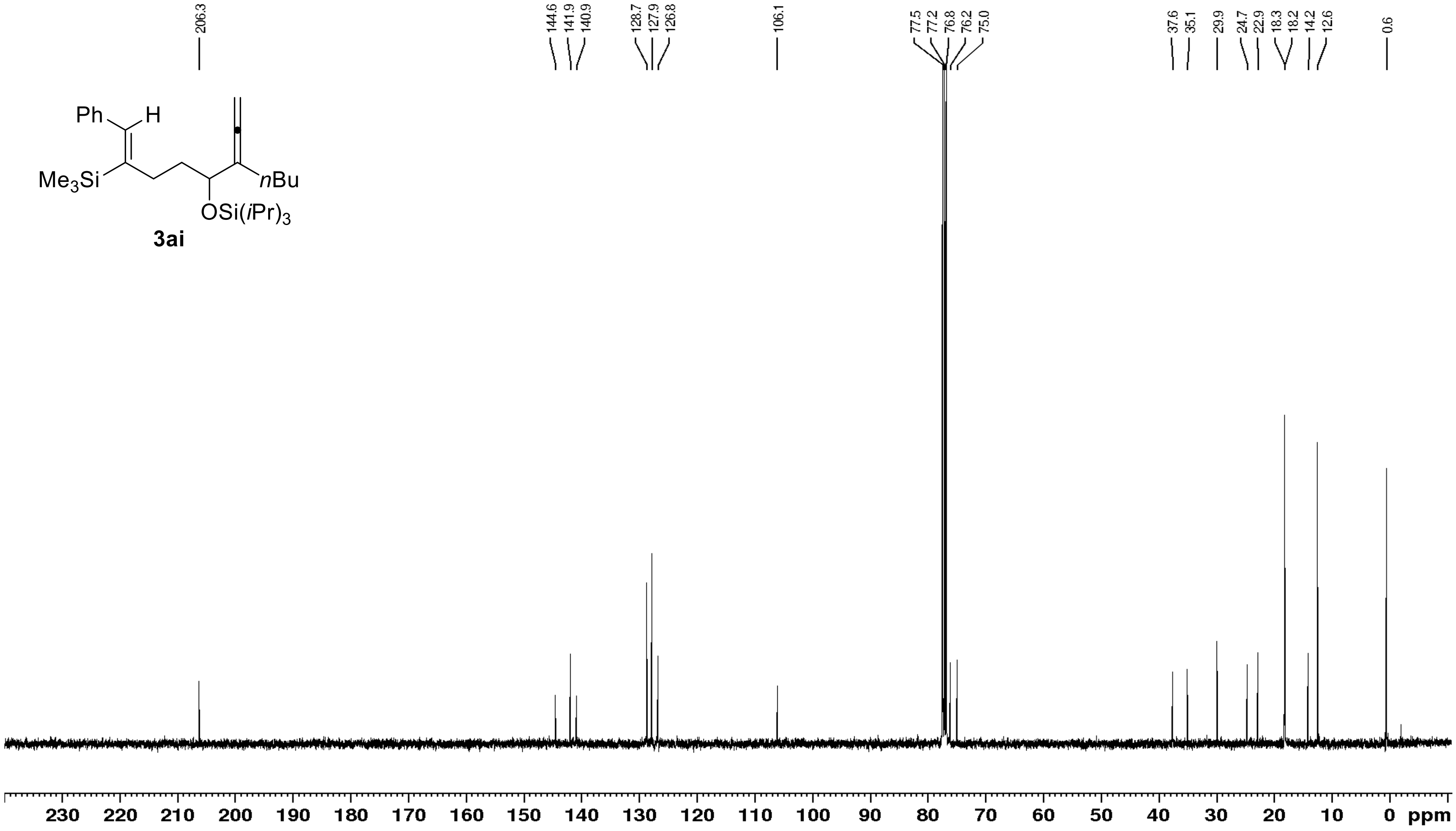

Figure S167. ^{29}Si DEPT NMR spectrum (79 MHz, $CDCl_3$, 298 K, optimized for J = 15.0 Hz) of **3ai**

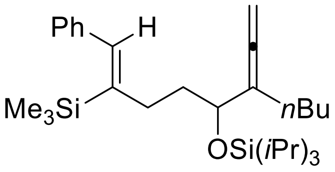

3ai

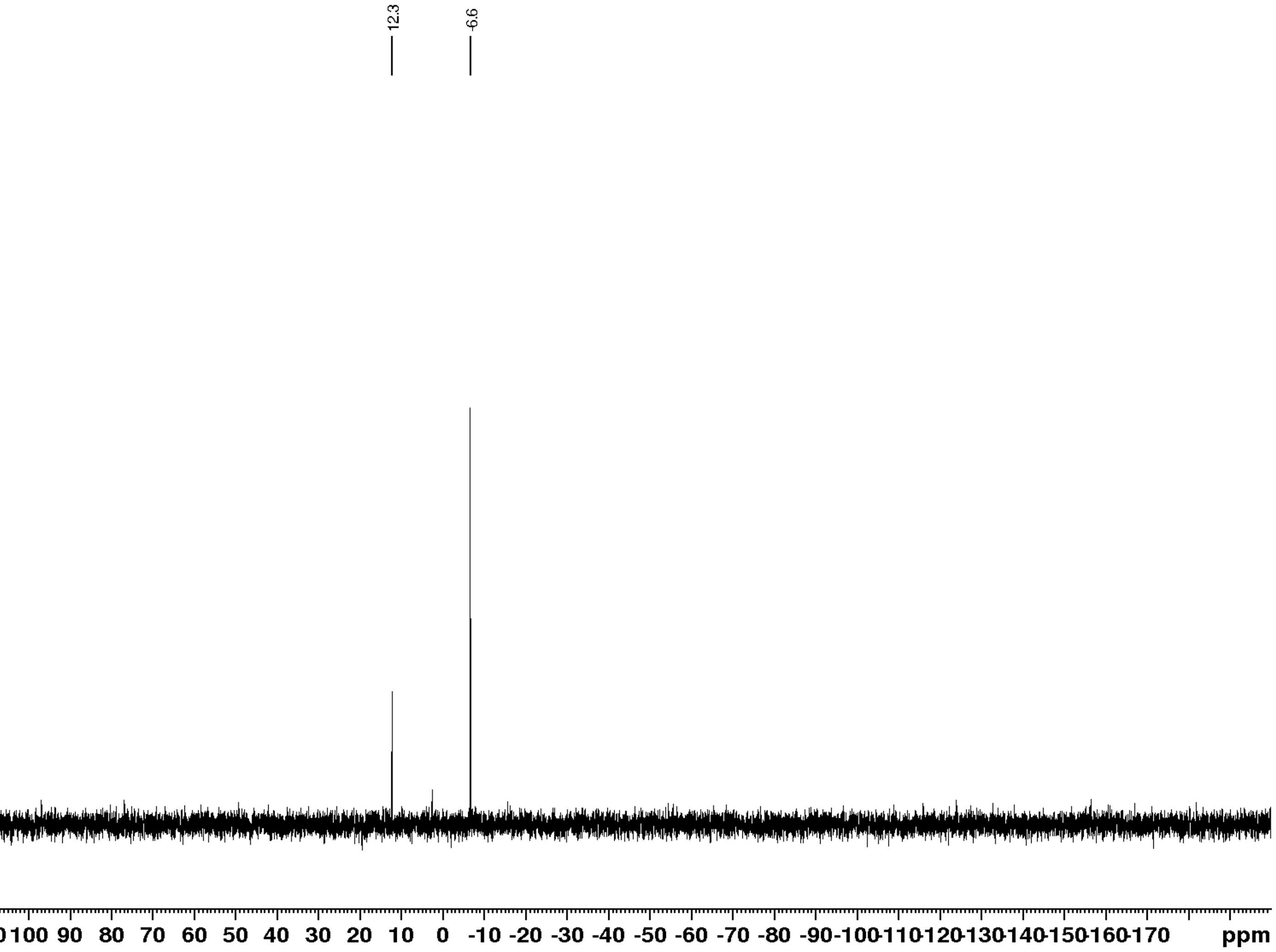

Figure S168. ^{1}H NMR spectrum (400 MHz, $CDCl_3$, 298 K) of **3aj**

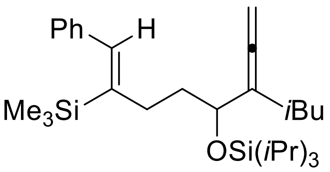

3aj

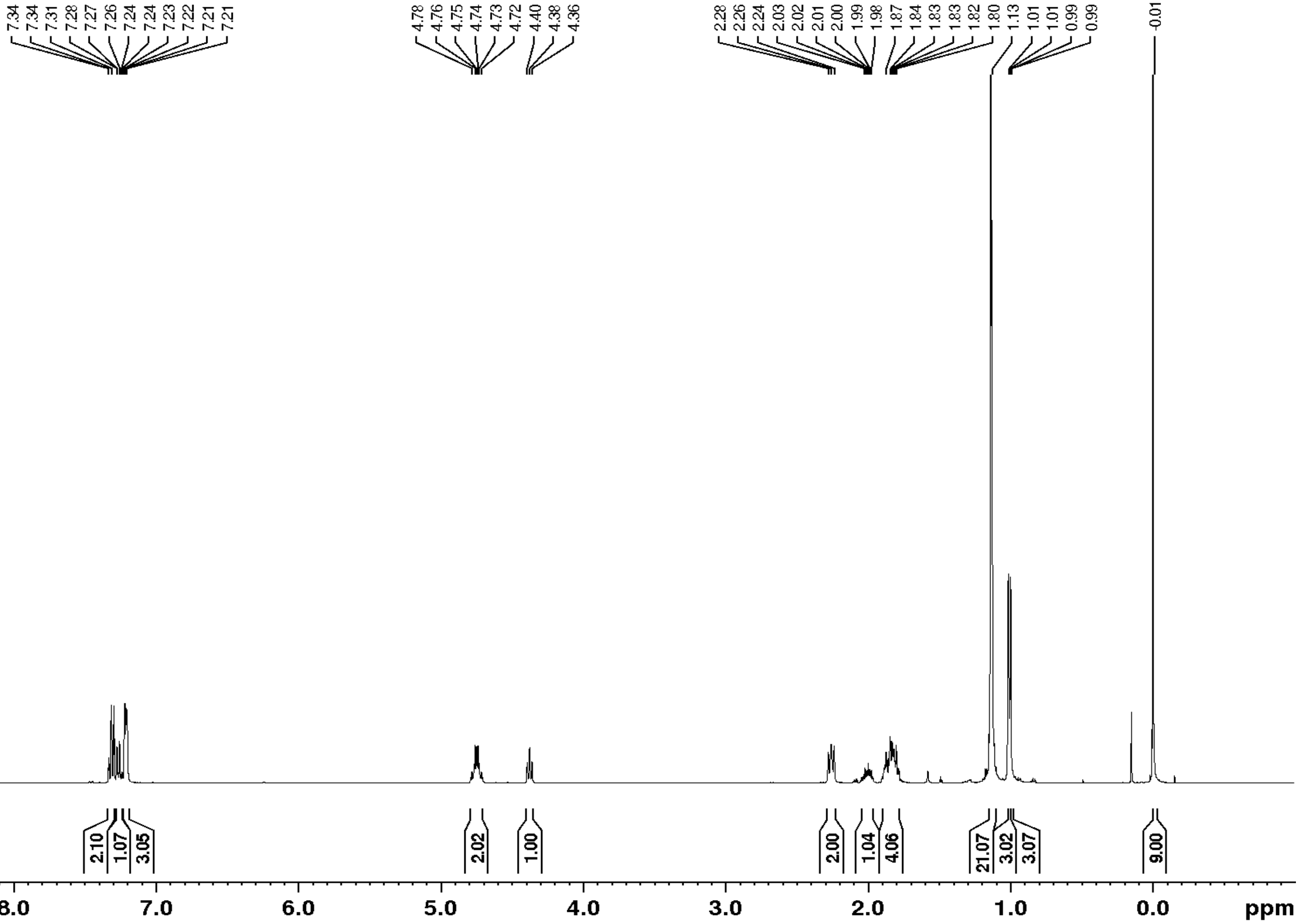

Figure S169. $^{13}C\{^1H\}$ NMR spectrum (101 MHz, $CDCl_3$, 298 K) of **3aj**

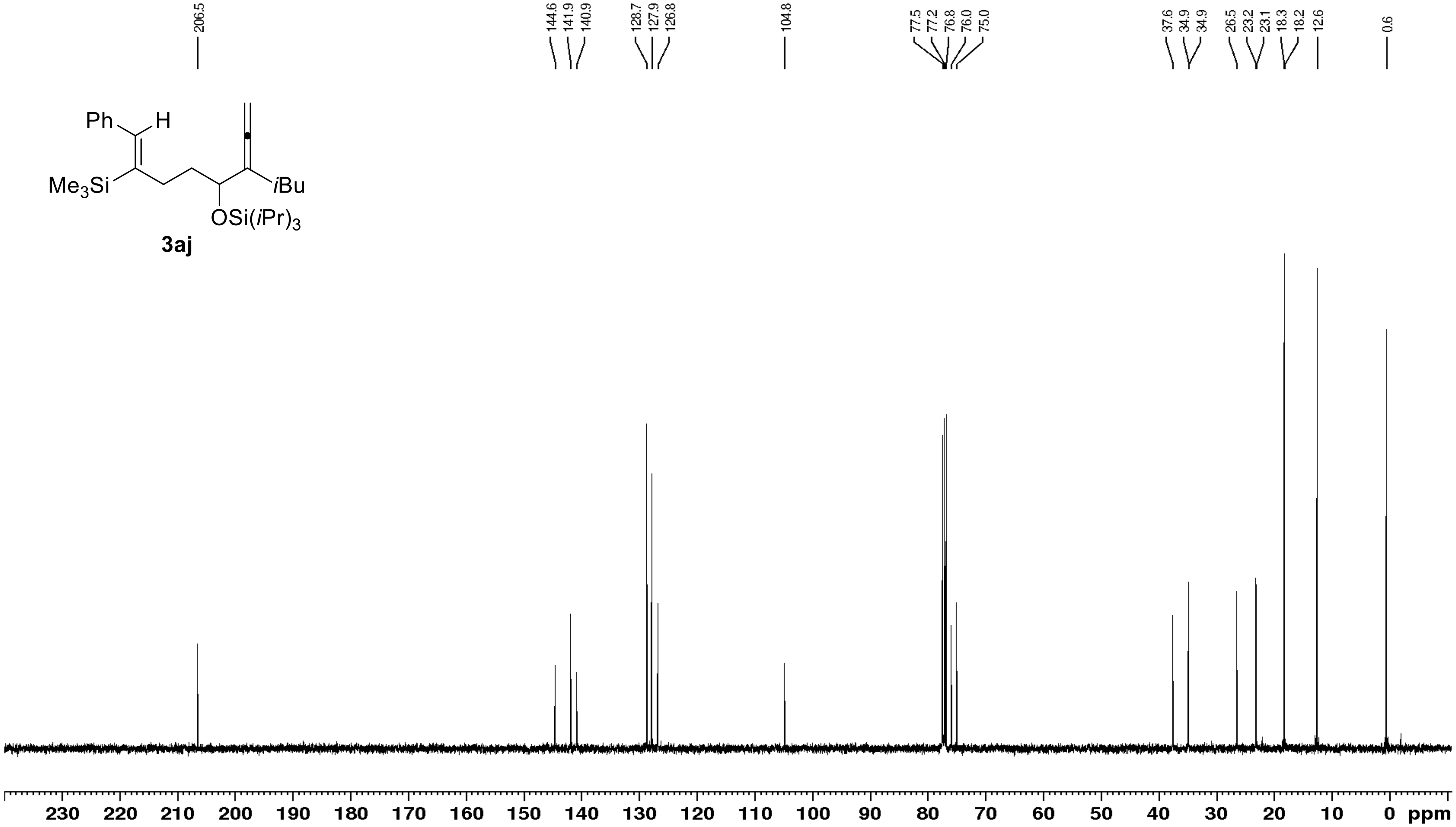

Figure S170. ^{29}Si DEPT NMR spectrum (79 MHz, $CDCl_3$, 298 K, optimized for J = 15.0 Hz) of **3aj**

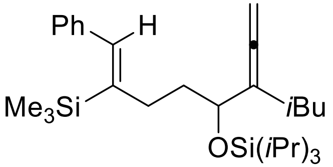

3aj

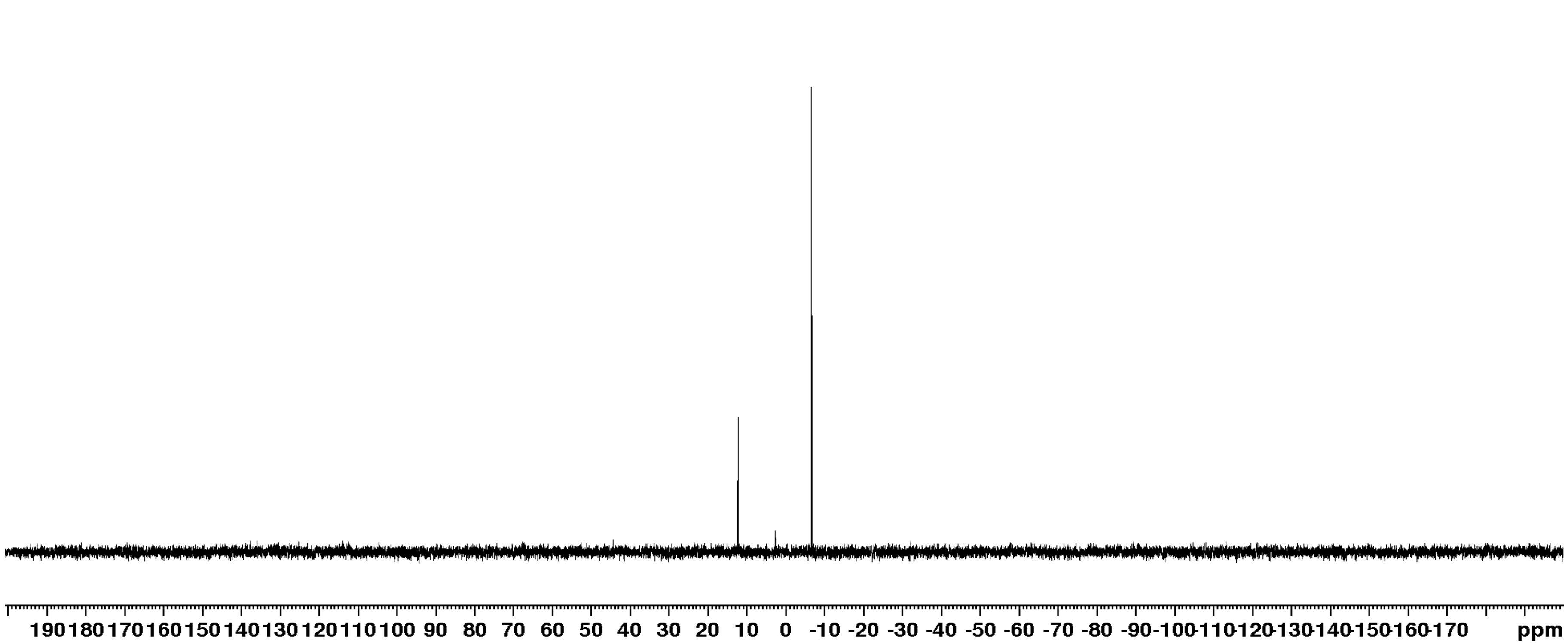

Figure S171. ^{1}H NMR spectrum (400 MHz, $CDCl_3$, 298 K) of **3ak**

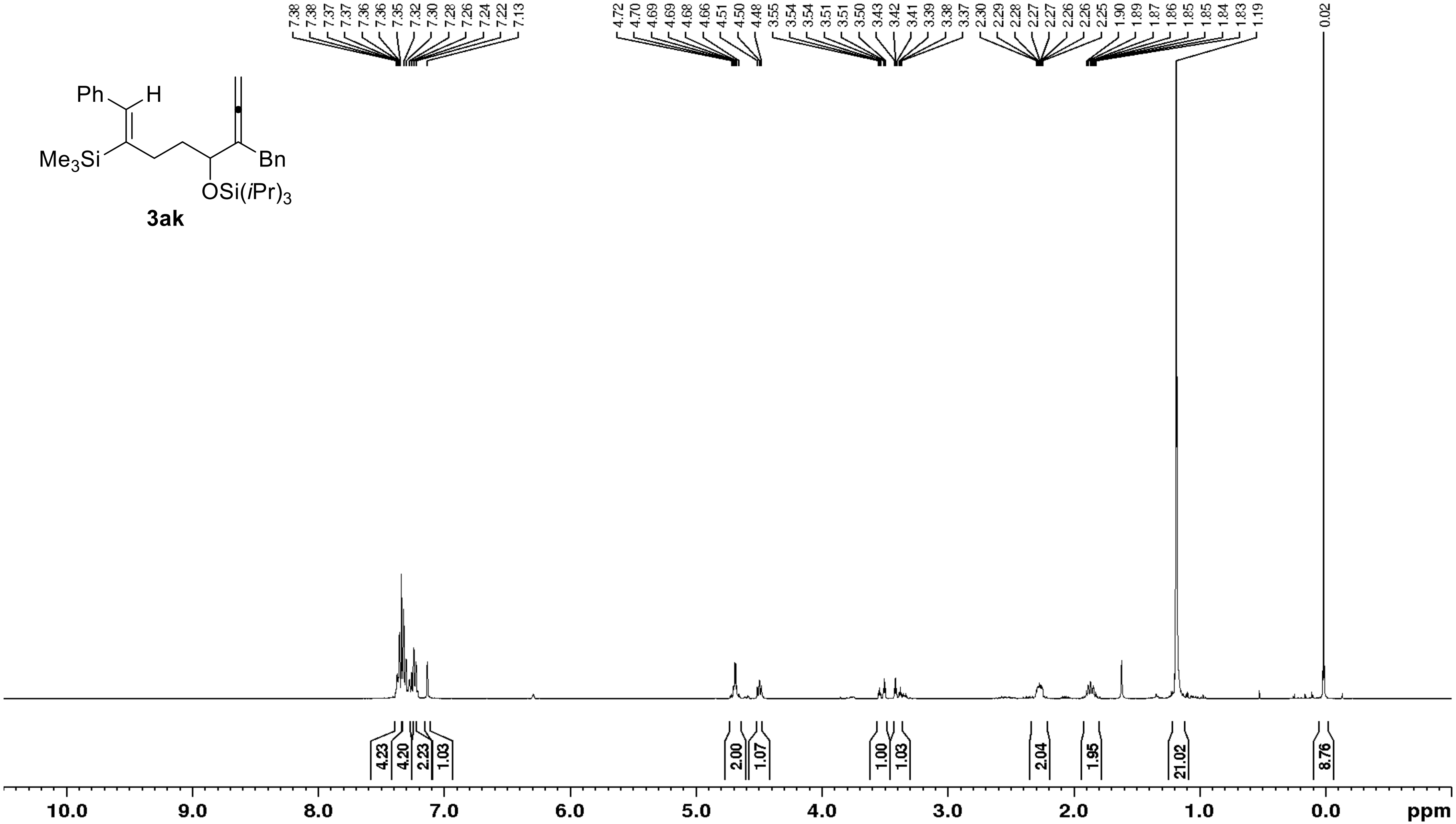

Figure S172. $^{13}C\{^{1}H\}$ NMR spectrum (101 MHz, $CDCl_3$, 298 K) of **3ak**

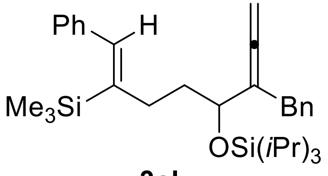

3ak

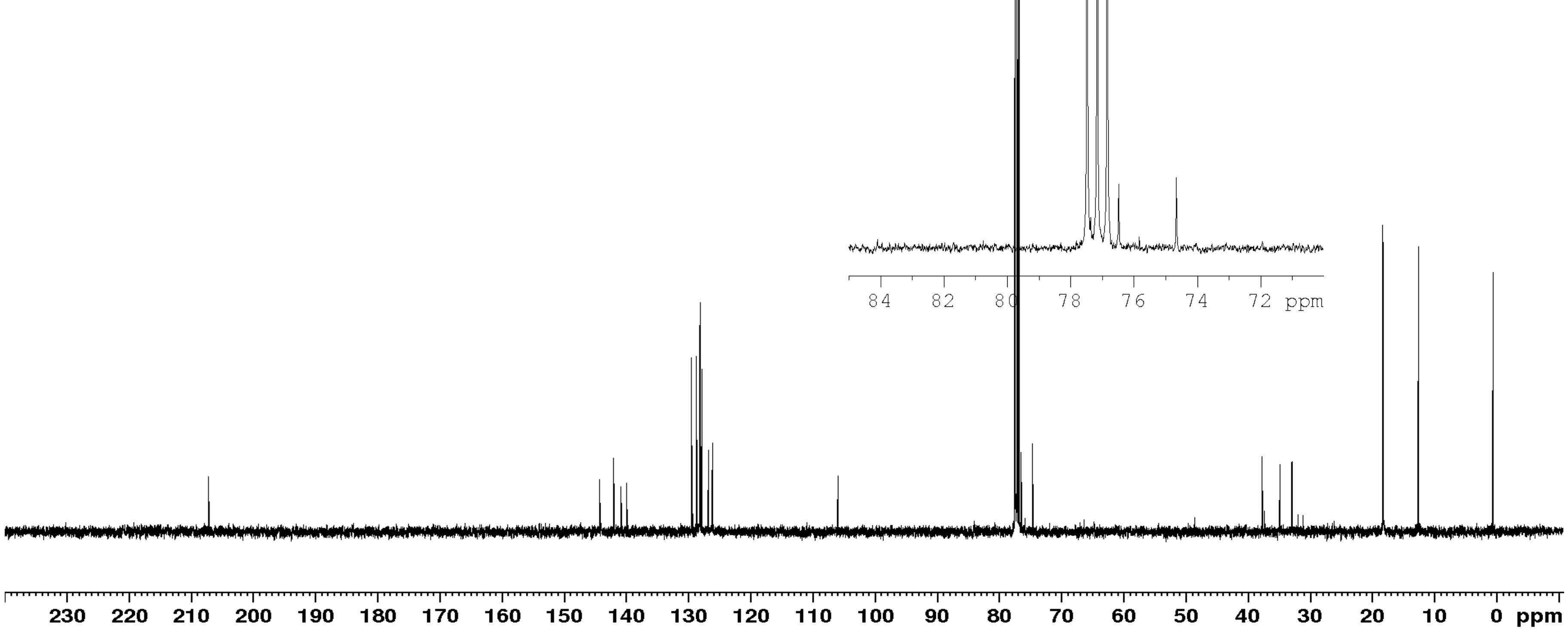

Figure S173. ^{29}Si DEPT NMR spectrum (79 MHz, $CDCl_3$, 298 K, optimized for J = 15.0 Hz) of **3ak**

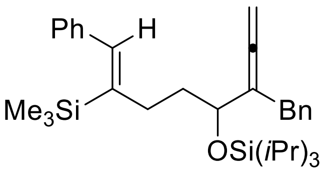

3ak

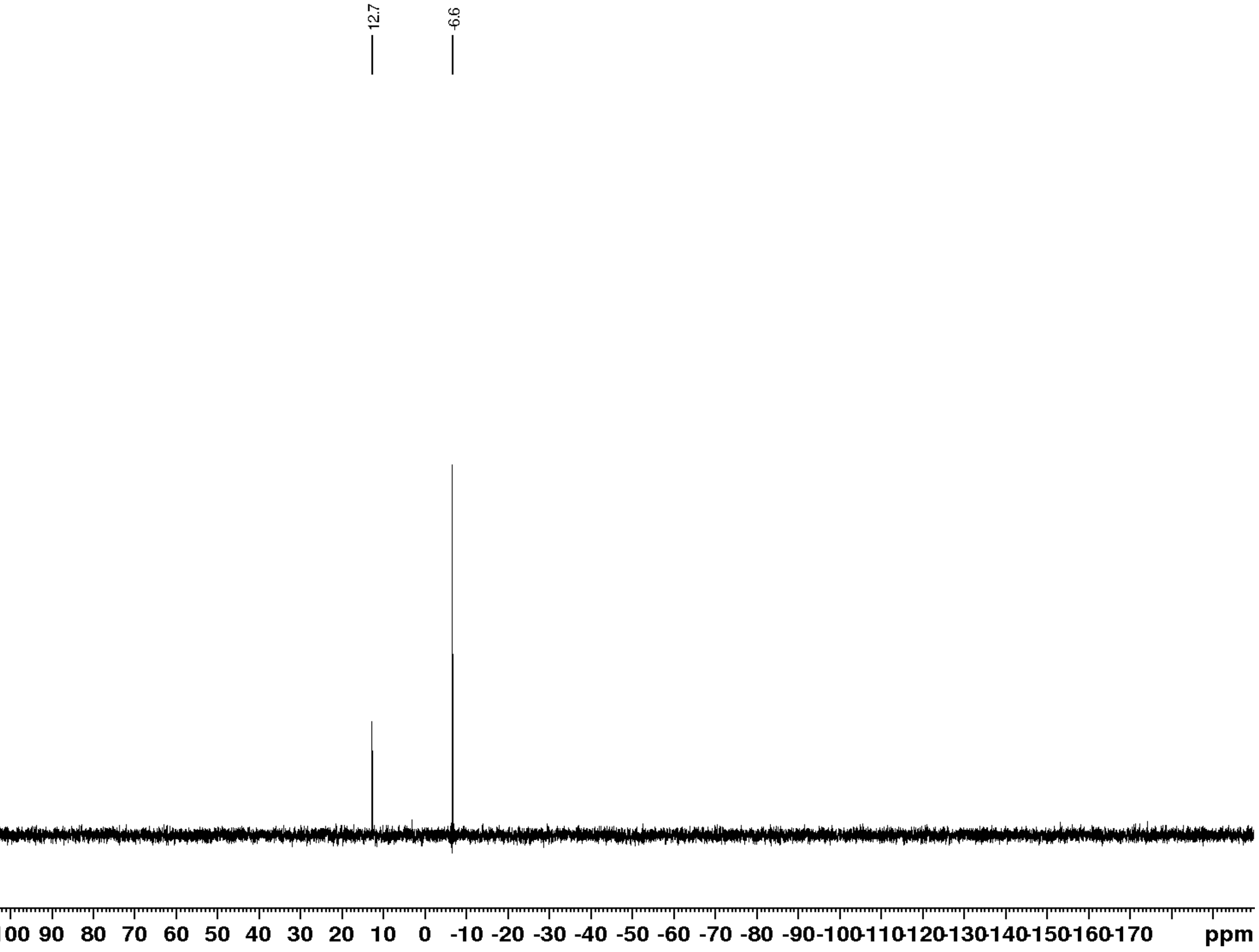

Figure S174. ^{1}H NMR spectrum (400 MHz, $CDCl_3$, 298 K) of **3al**

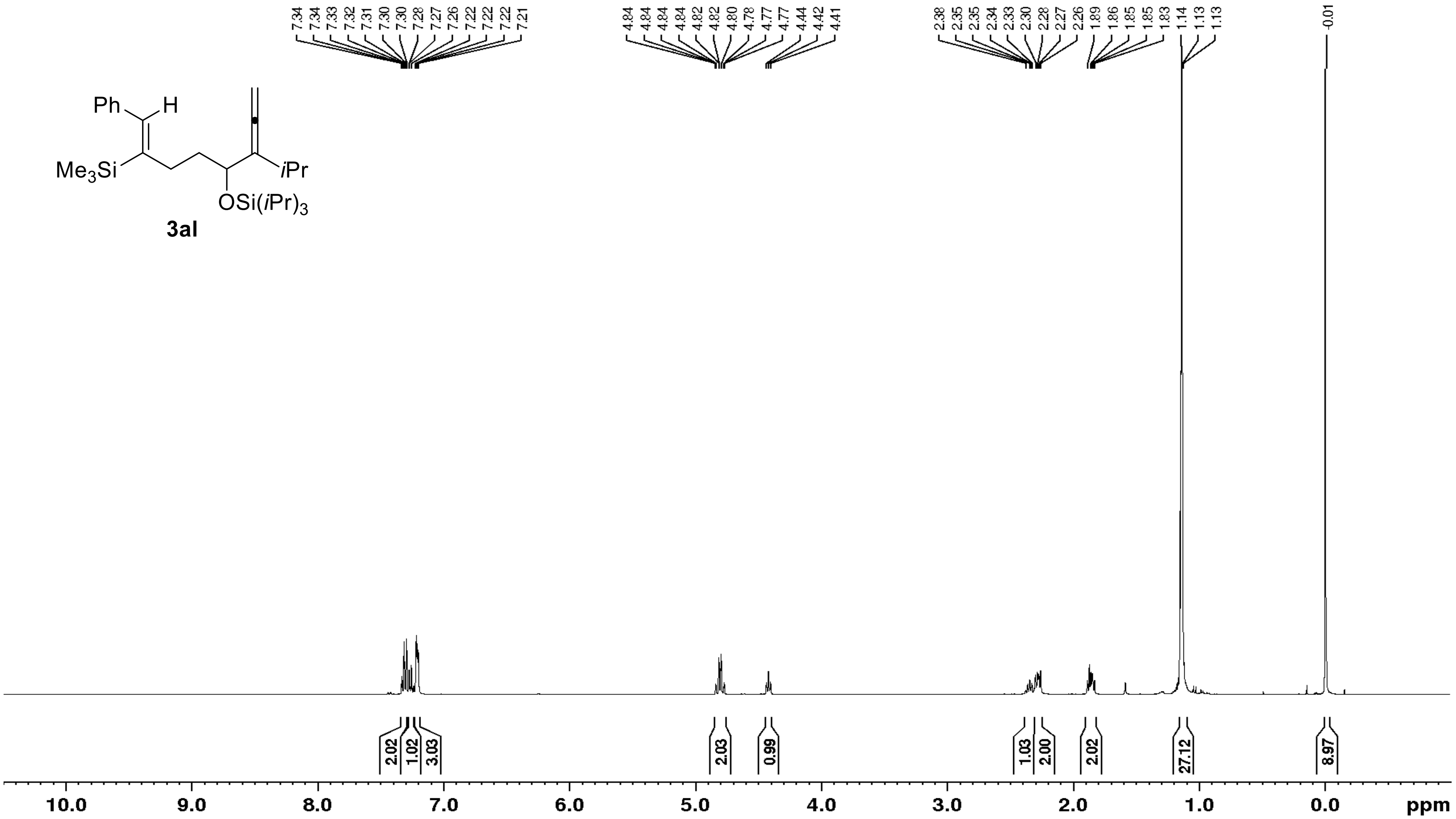

Figure S175. $^{13}C\{^{1}H\}$ NMR spectrum (101 MHz, $CDCl_3$, 298 K) of **3al**

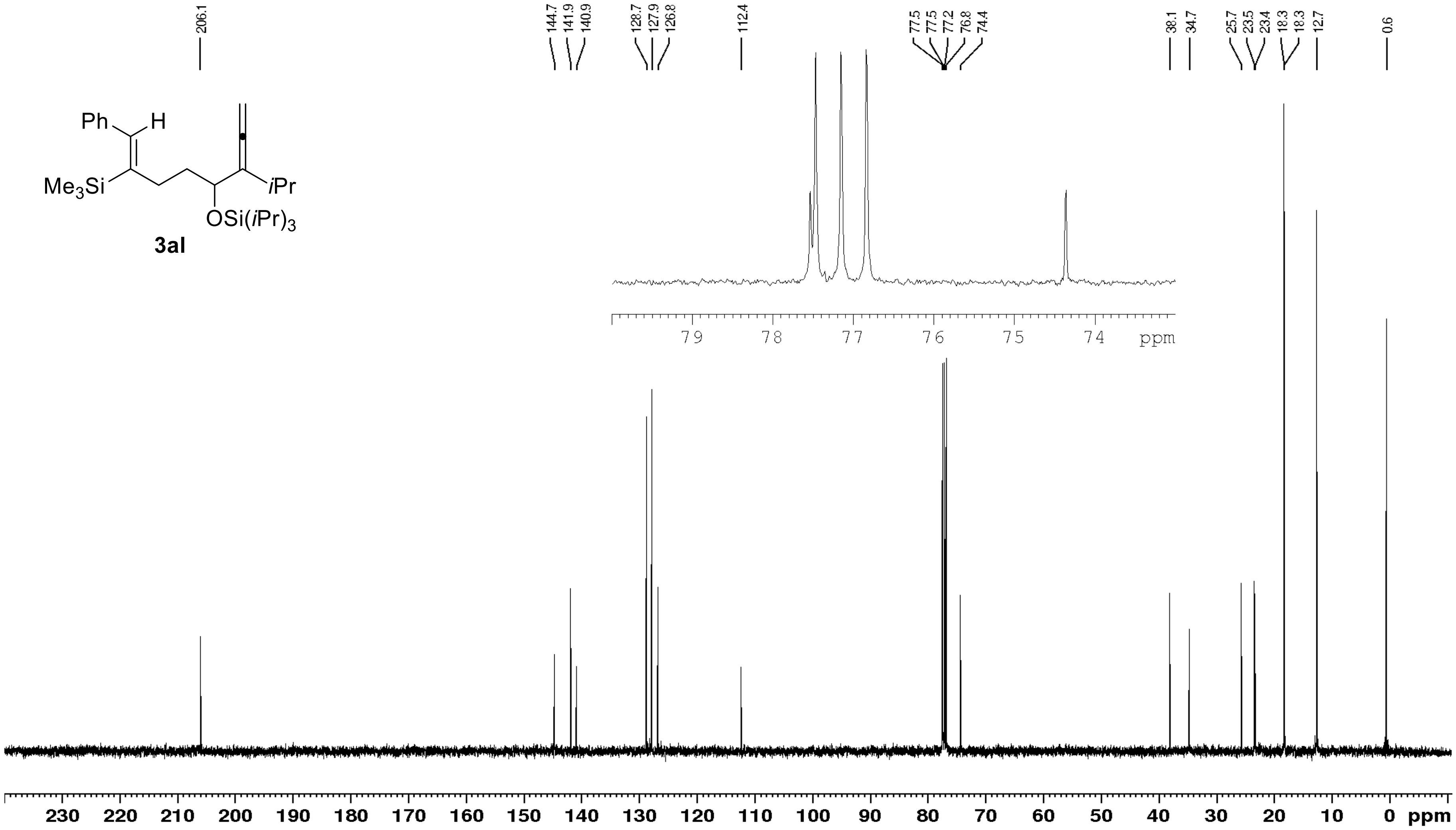

Figure S176. ^{29}Si DEPT NMR spectrum (79 MHz, $CDCl_3$, 298 K, optimized for J = 15.0 Hz) of **3al**

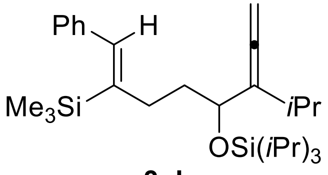

3al

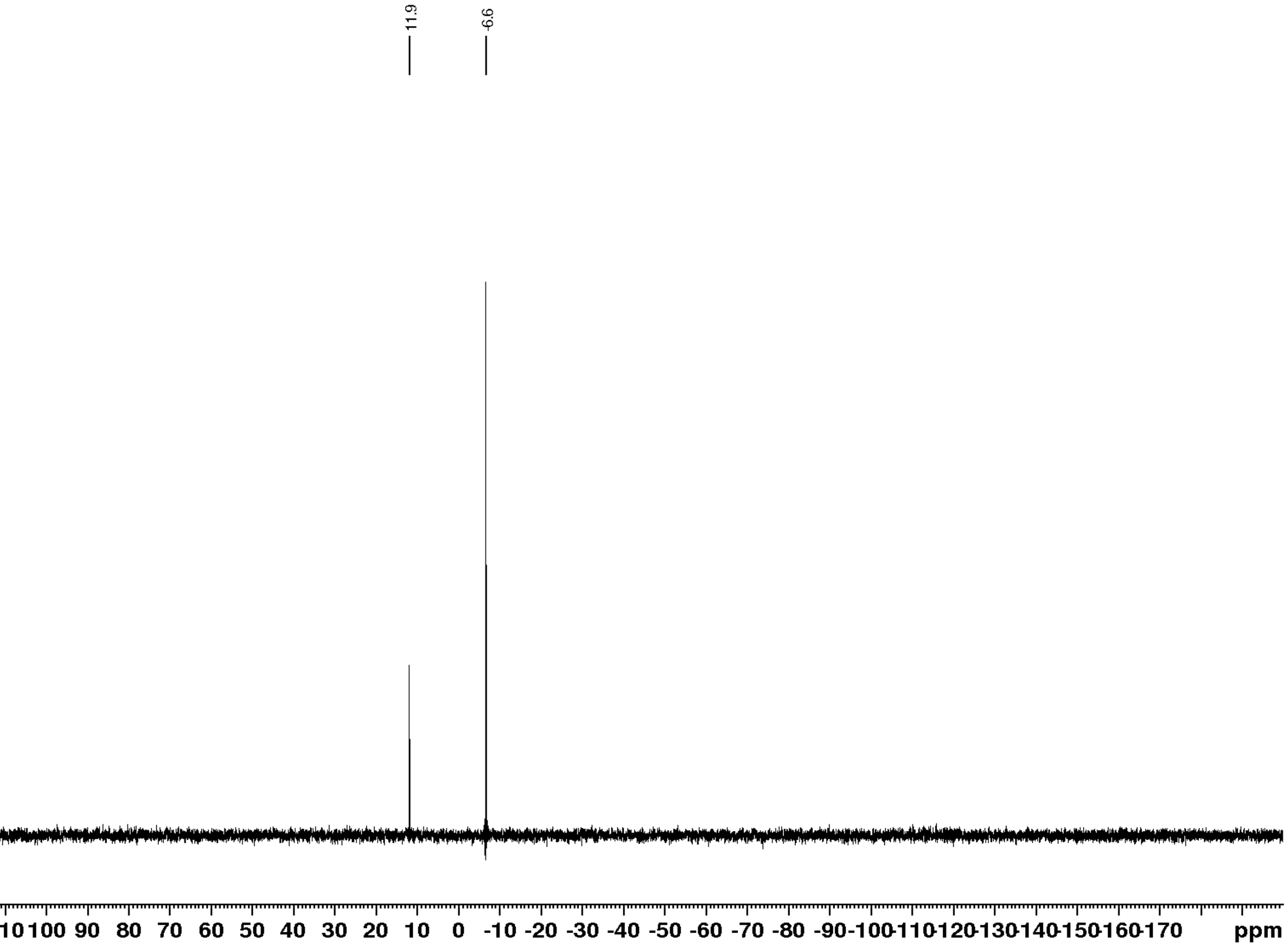

Figure S177. ^{1}H NMR spectrum (400 MHz, $CDCl_3$, 298 K) of **3am**

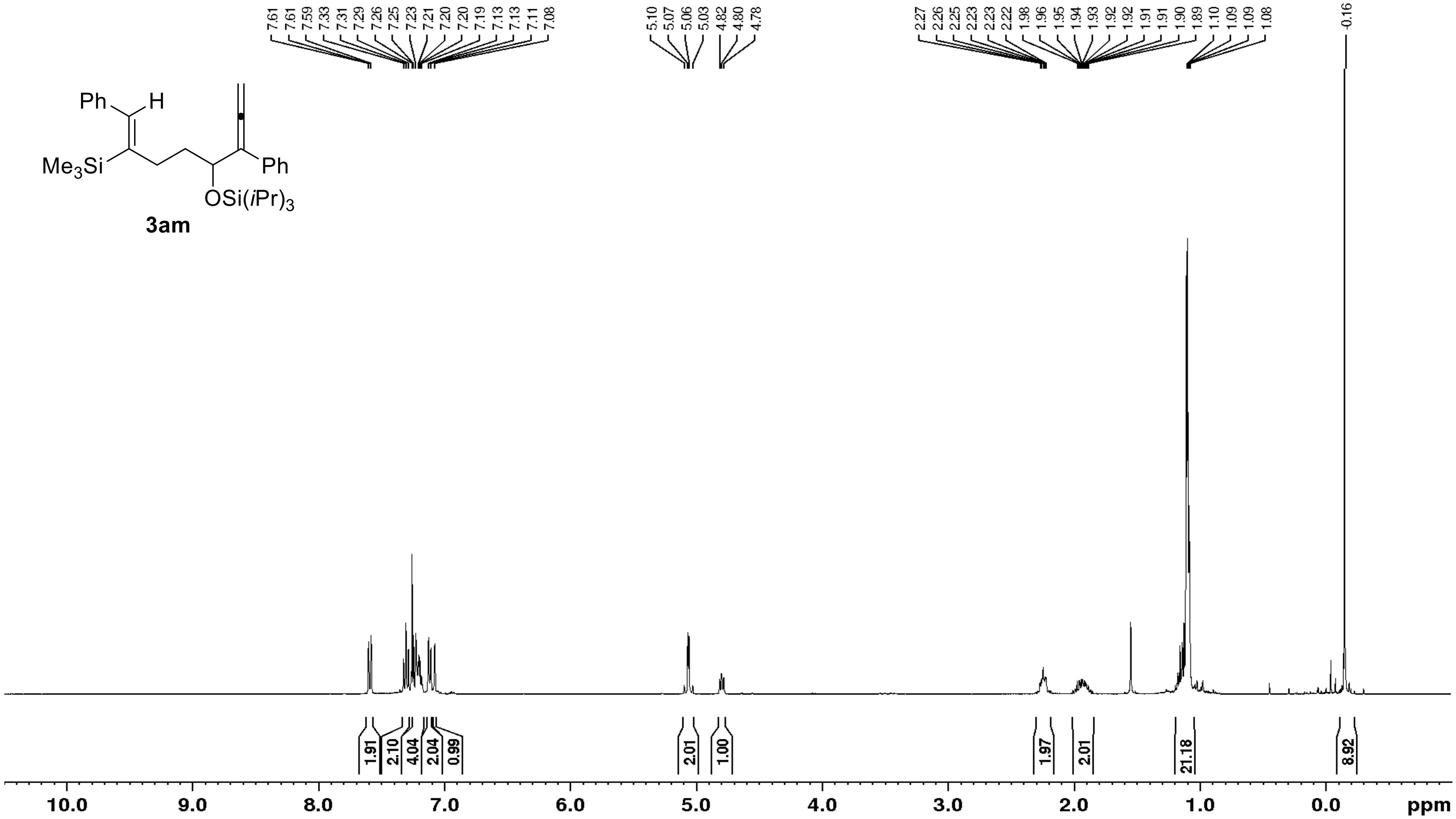

Figure S178. $^{13}C\{^{1}H\}$ NMR spectrum (101 MHz, $CDCl_3$, 298 K) of **3am**

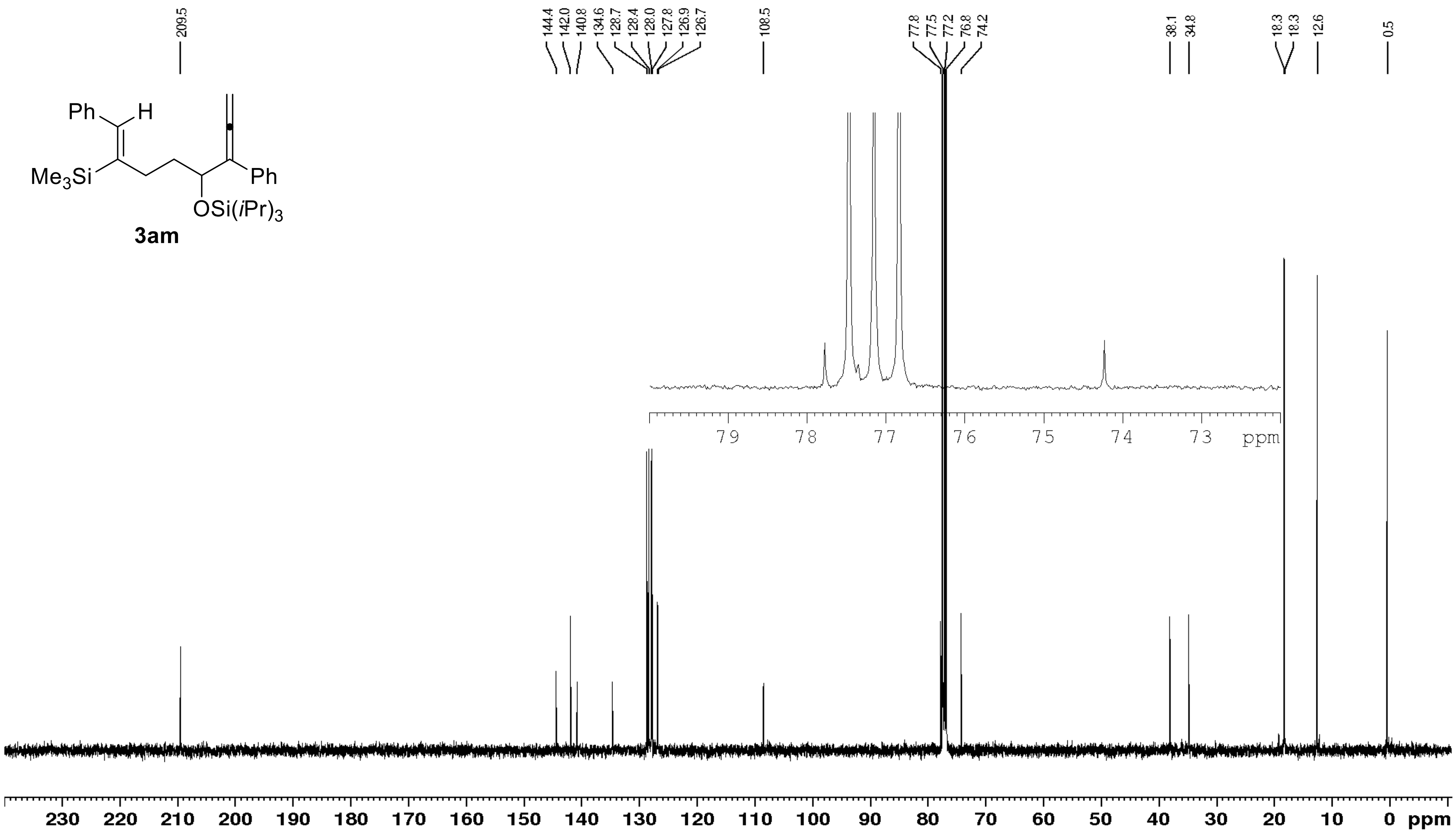

Figure S179. ^{29}Si DEPT NMR spectrum (79 MHz, $CDCl_3$, 298 K, optimized for J = 15.0 Hz) of **3am**

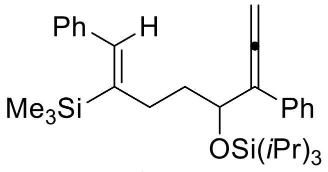

3am

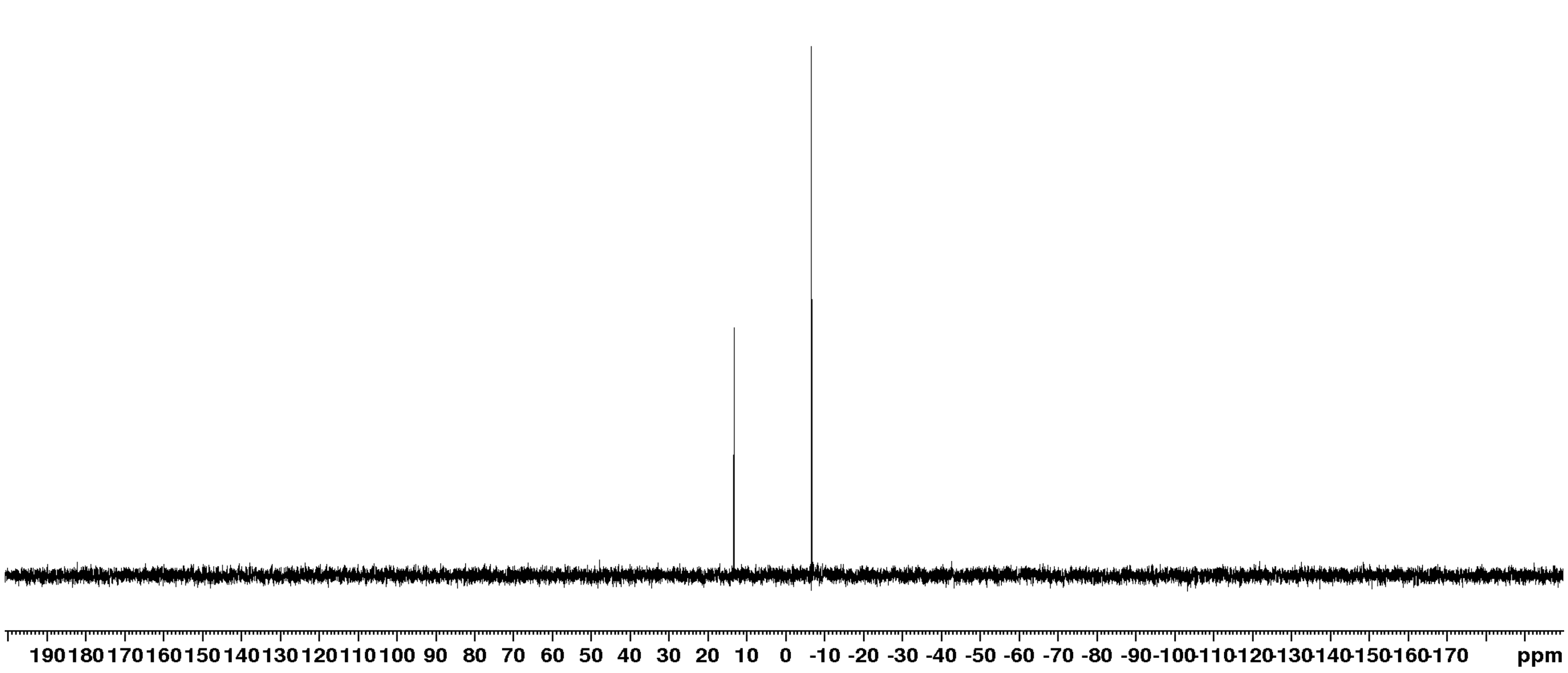

Figure S180. ^{1}H NMR spectrum (400 MHz, $CDCl_3$, 298 K) of **6aa**

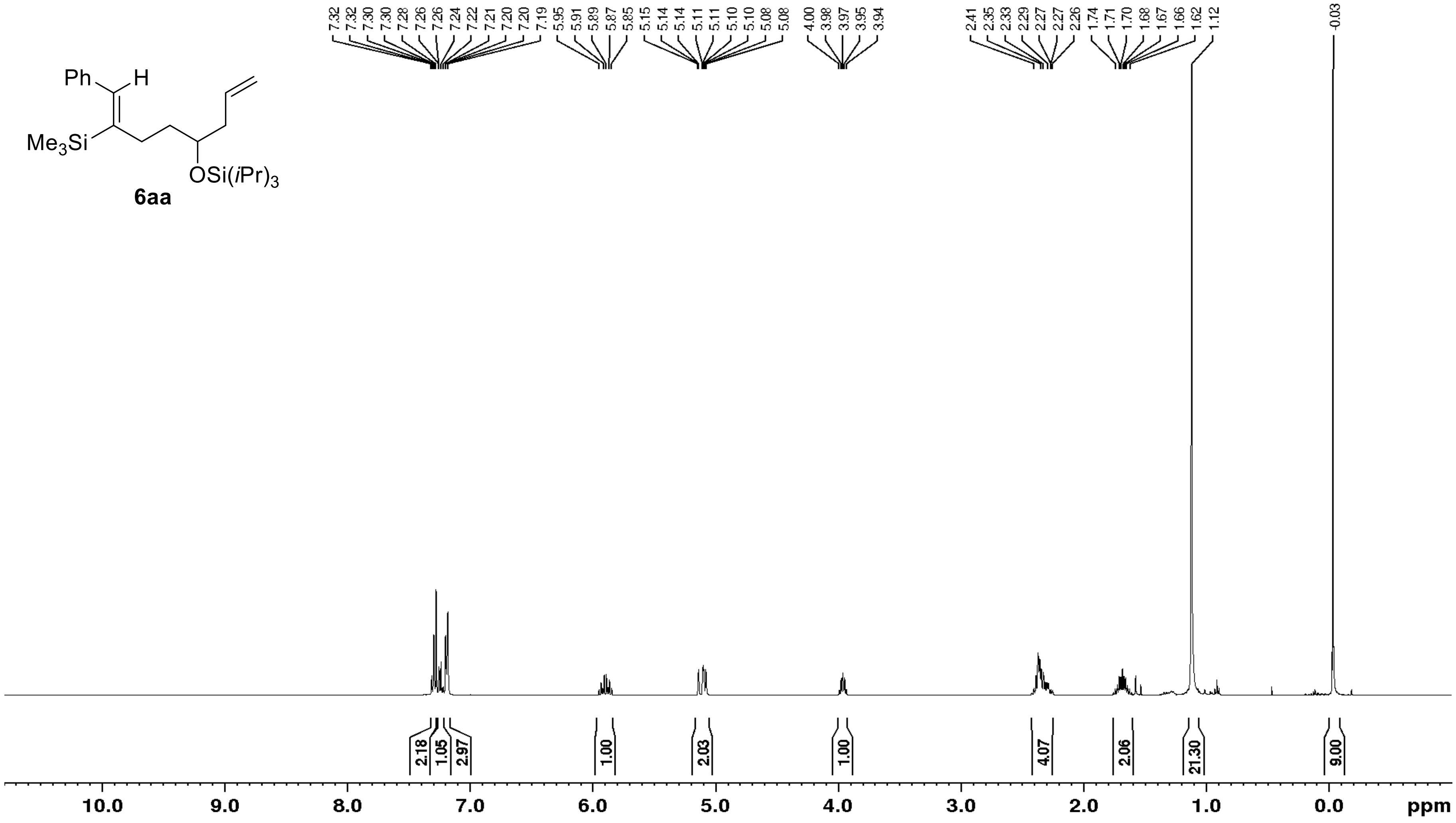

Figure S181. $^{13}C\{^{1}H\}$ NMR spectrum (101 MHz, $CDCl_3$, 298 K) of **6aa**

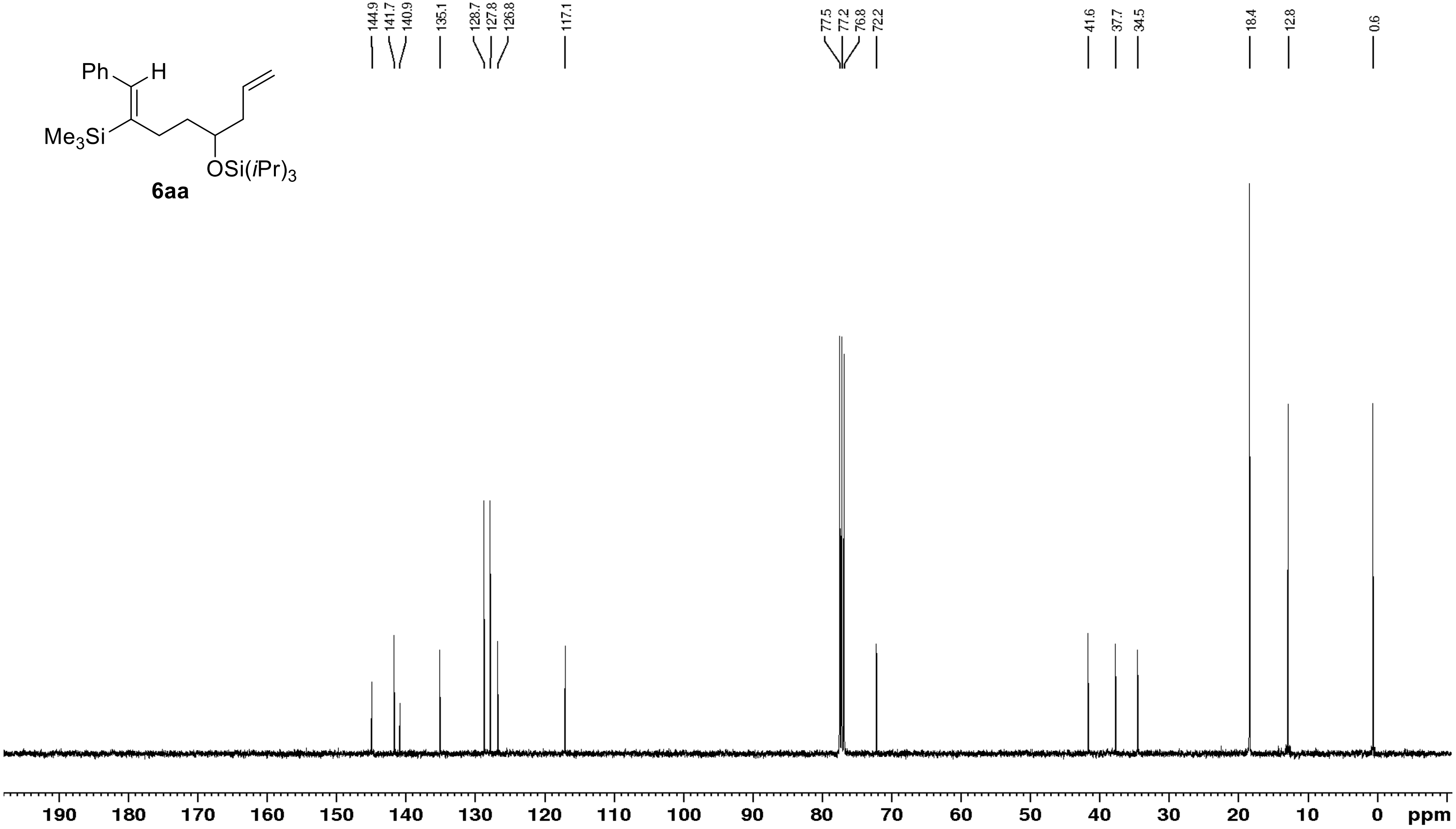

Figure S182. ^{29}Si DEPT NMR spectrum (79 MHz, $CDCl_3$, 298 K, optimized for J = 15.0 Hz) of **6aa**

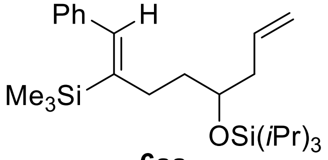

6aa

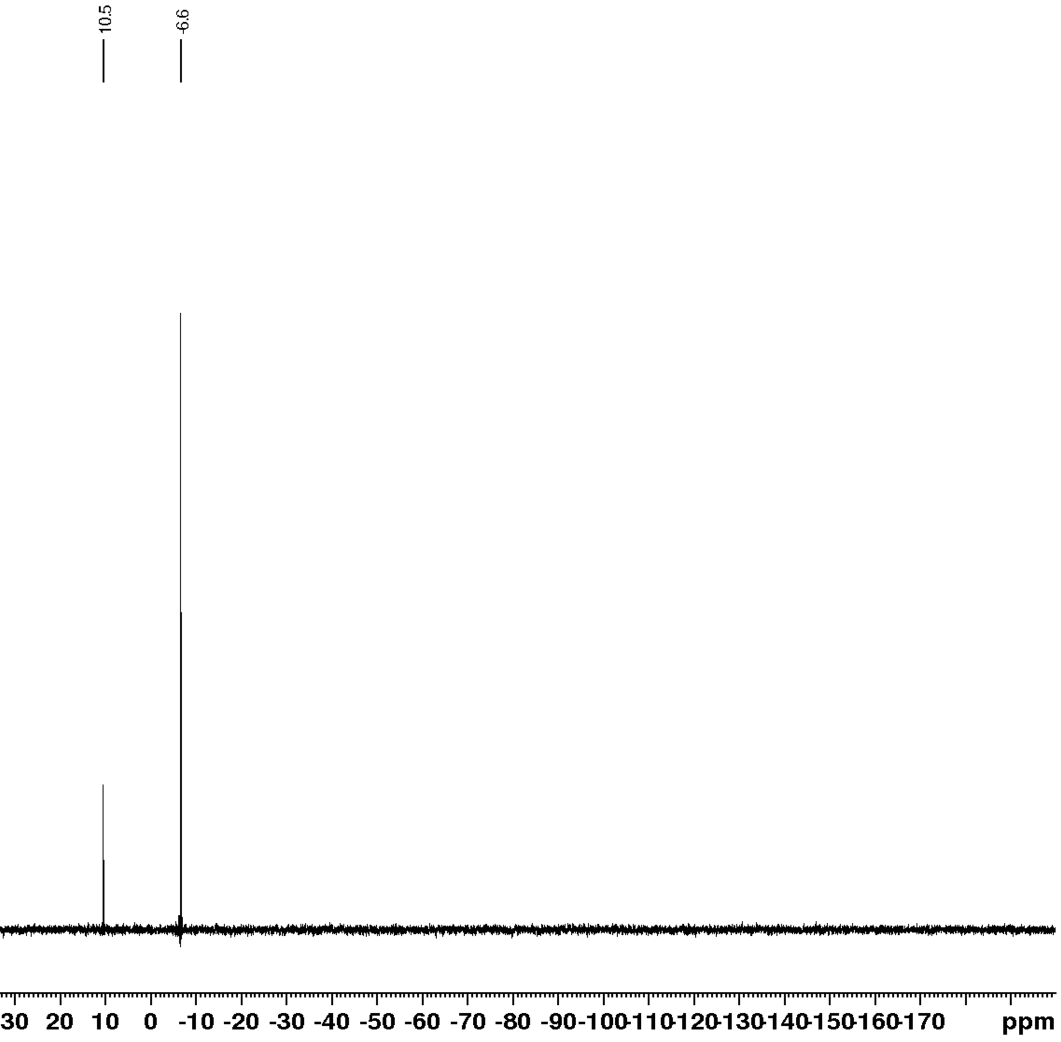

Figure S183. ^{1}H NMR spectrum (400 MHz, $CDCl_3$, 298 K) of **1a**-d_2

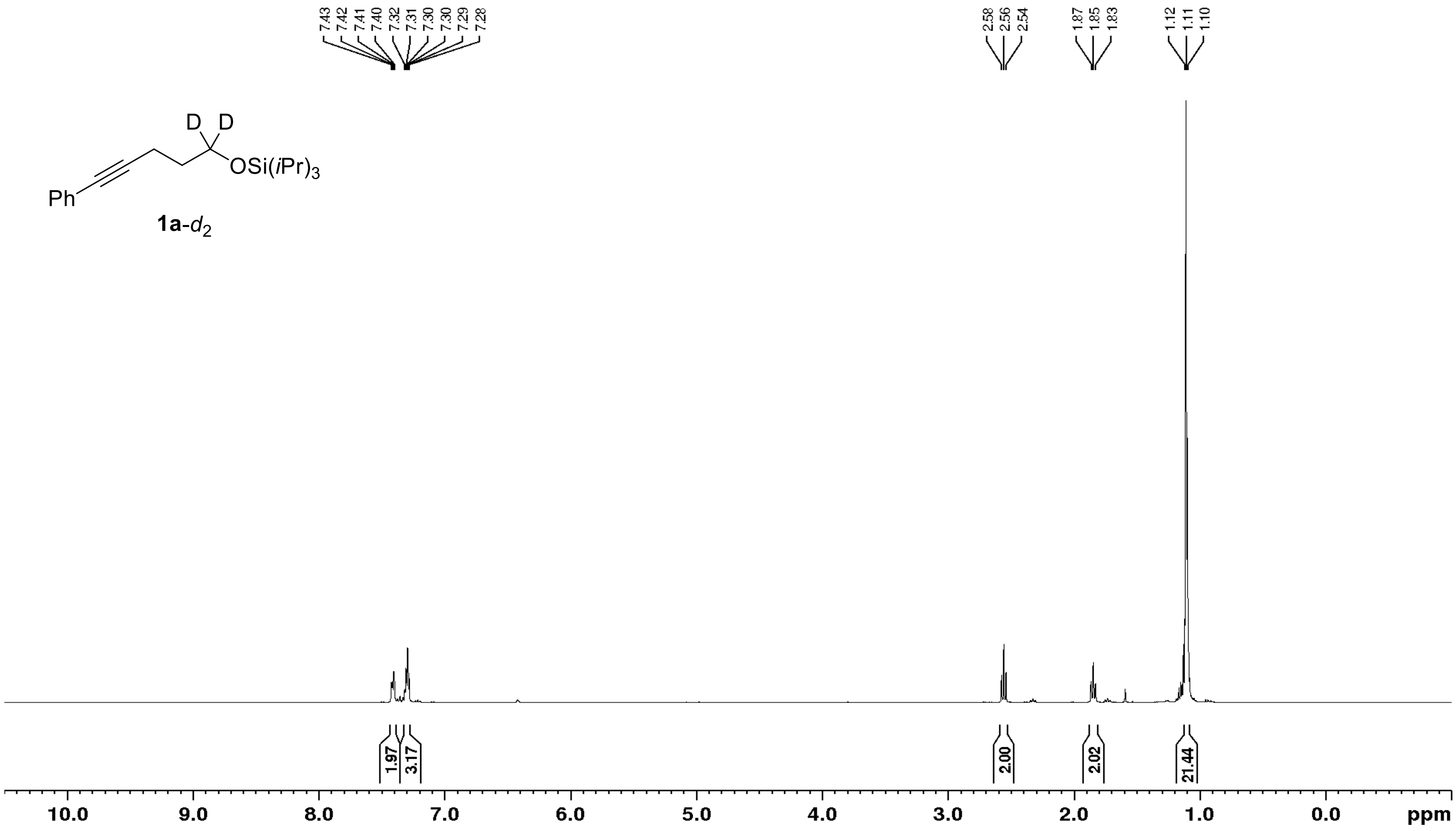

Figure S184. $^{13}C\{^{1}H\}$ NMR spectrum (101 MHz, $CDCl_3$, 298 K) of **1a**-d_2

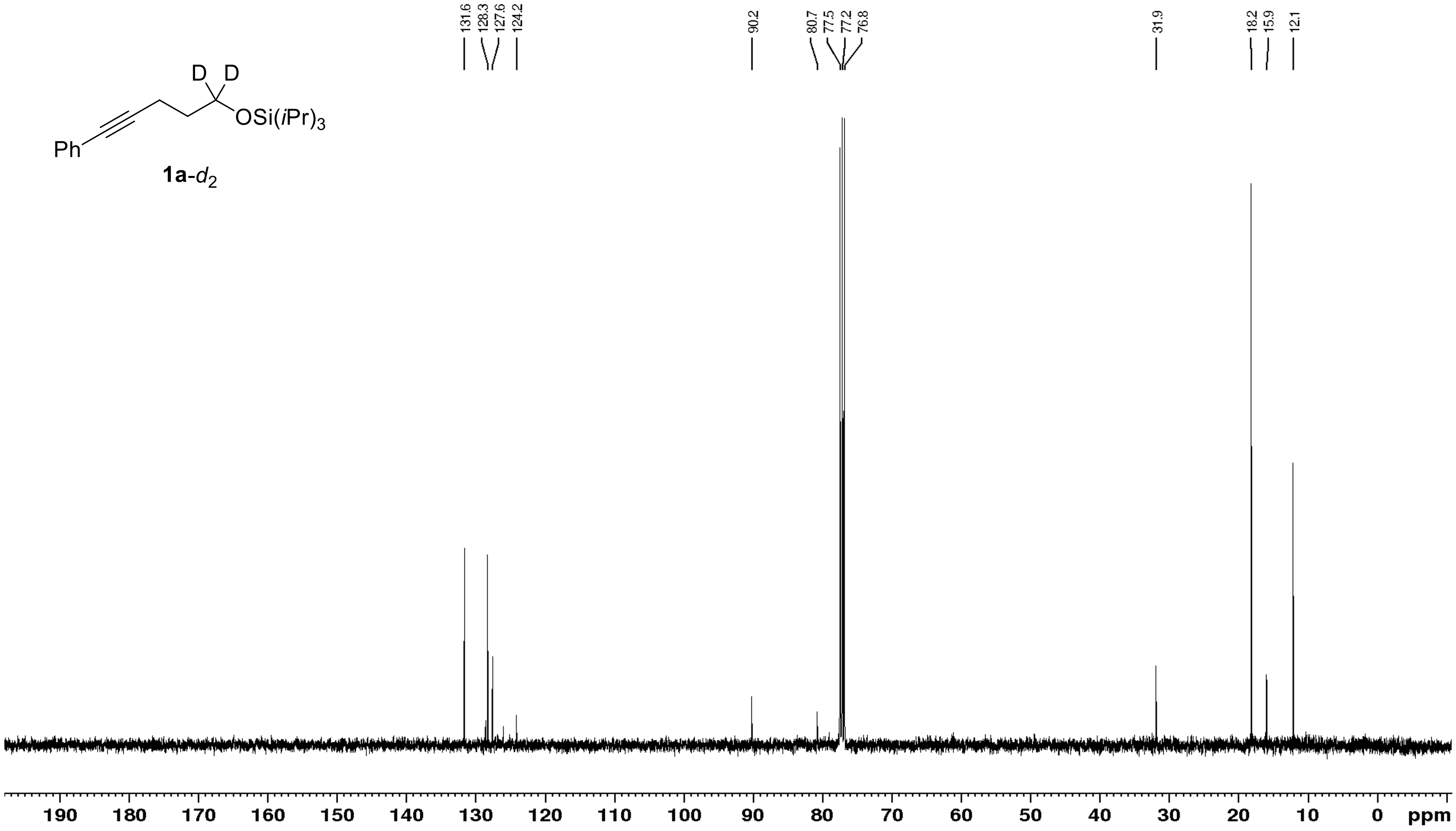

Figure S185. ^{29}Si DEPT NMR spectrum (99 MHz, $CDCl_3$, 298 K, optimized for J = 15.0 Hz) of **1a**-d_2

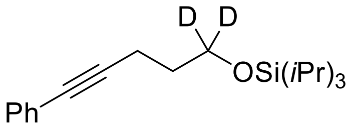

1a-d_2

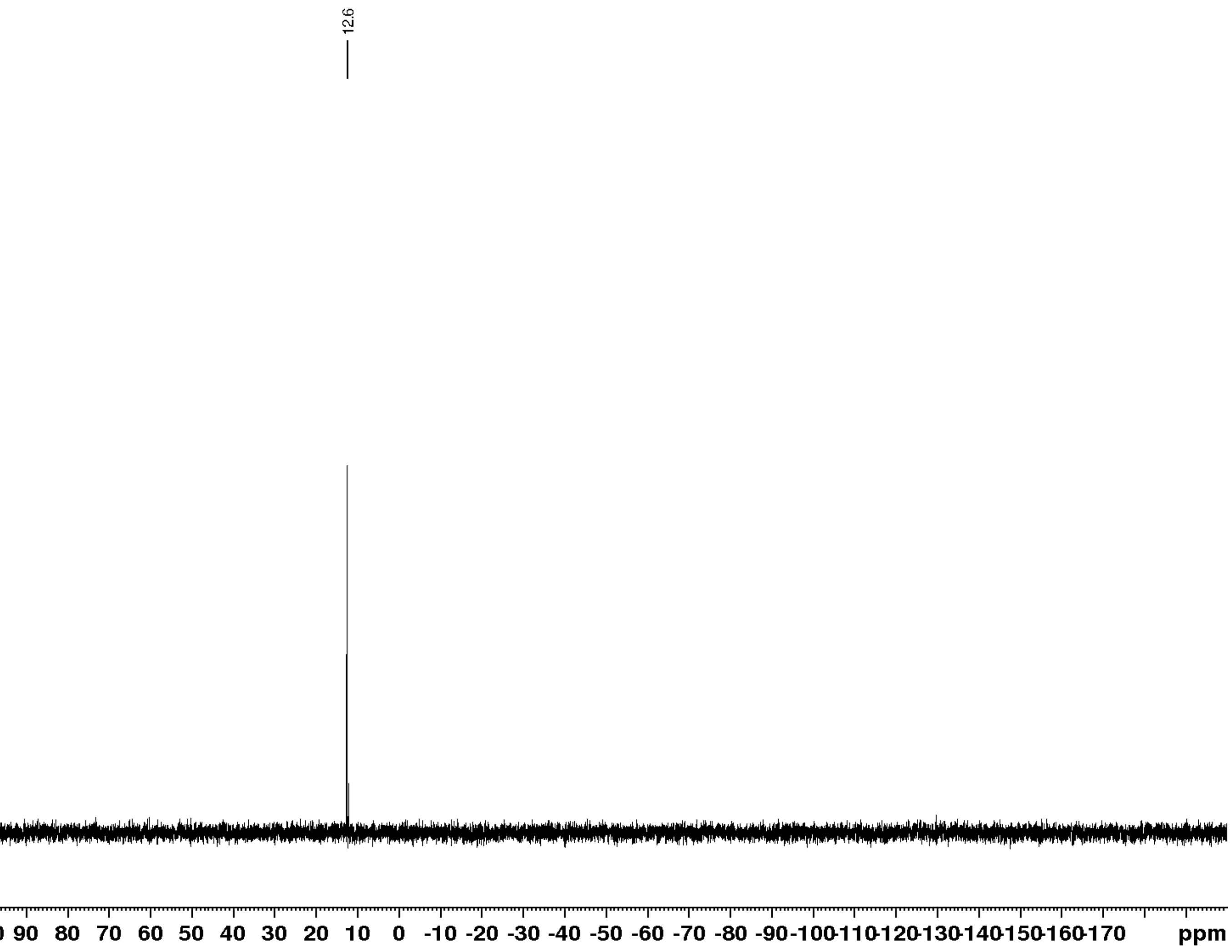

Figure S186. ^{1}H NMR spectrum (400 MHz, $CDCl_3$, 298 K) of **3aa**-d_2

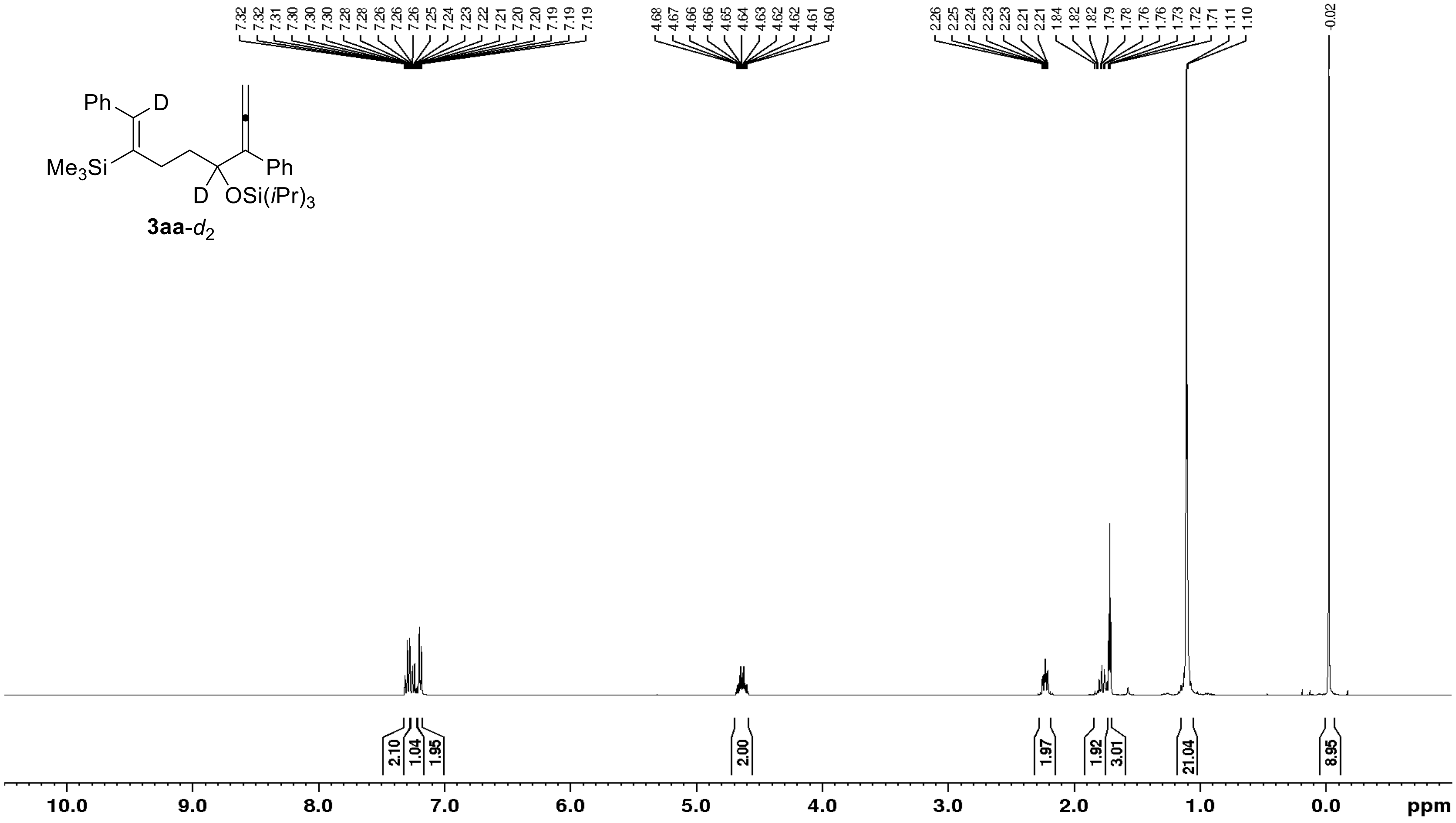

Figure S187. $^{13}C\{^1H\}$ NMR spectrum (101 MHz, $CDCl_3$, 298 K) of **3aa**-d_2

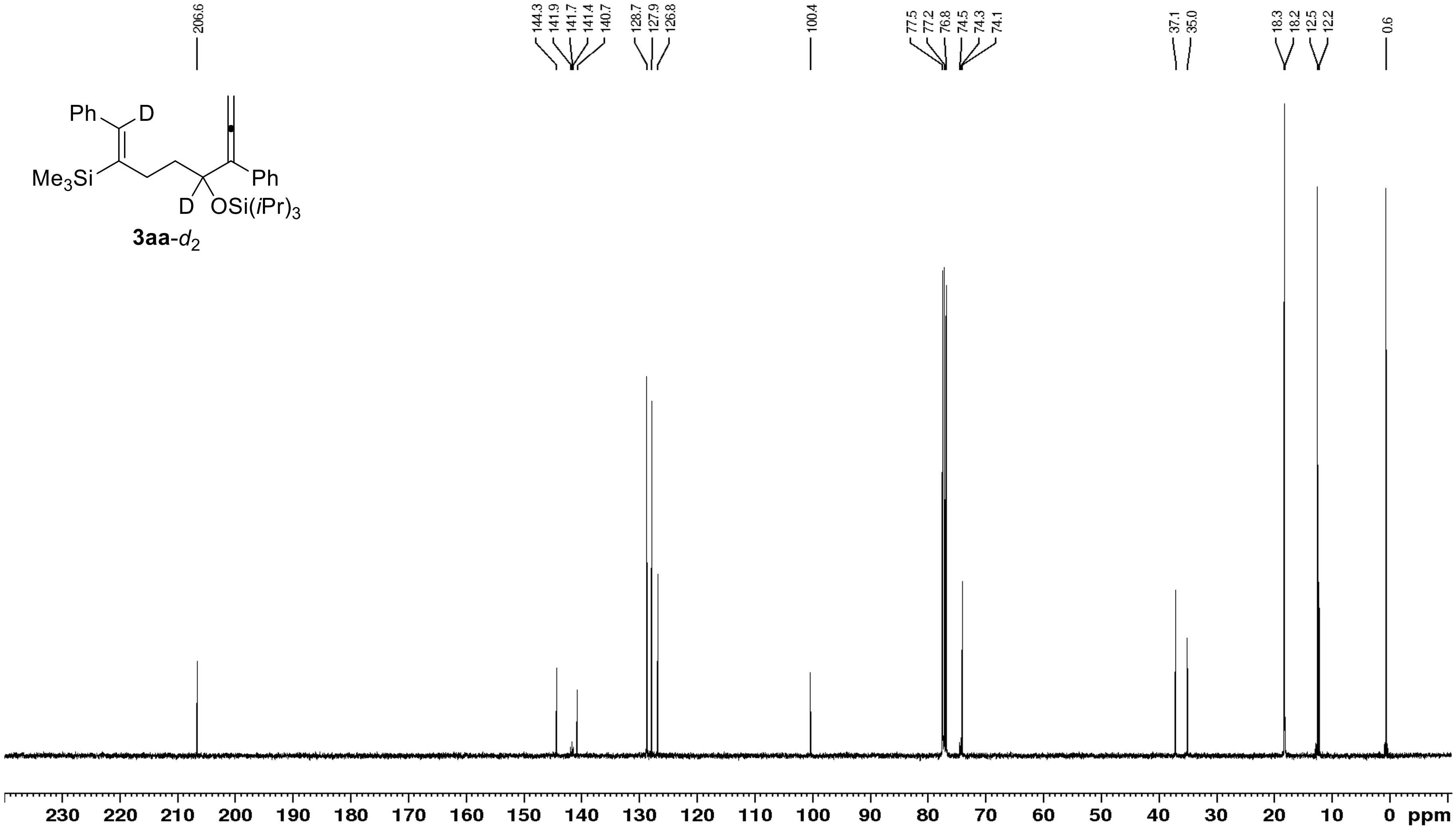

Figure S188. ^{29}Si DEPT NMR spectrum (79 MHz, $CDCl_3$, 298 K, optimized for J = 15.0 Hz) of **3aa**-d_2

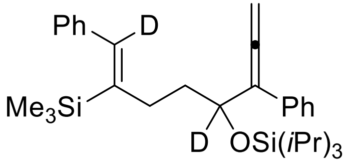

3aa-d_2

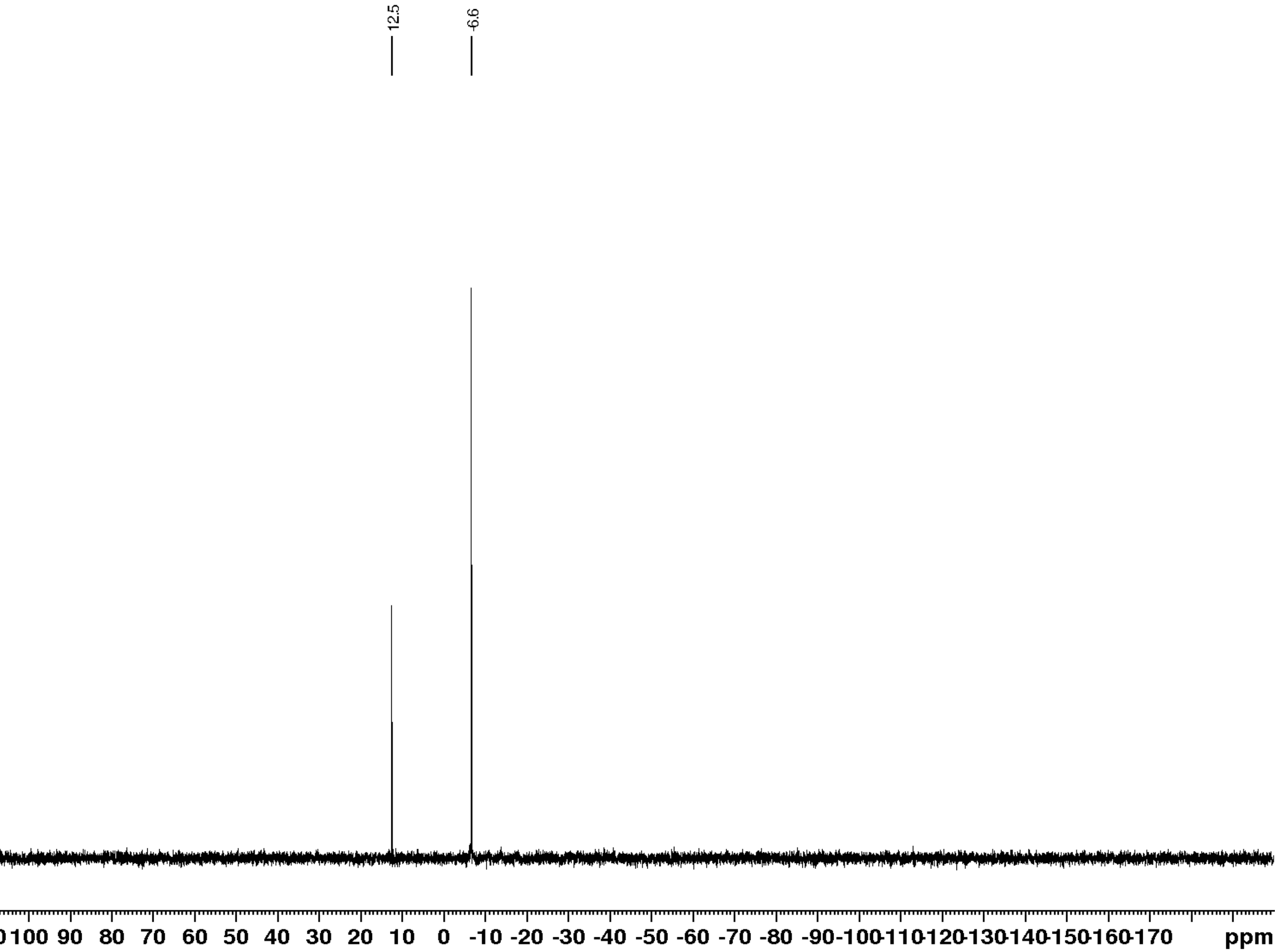

Figure S189. ^{1}H NMR spectrum (400 MHz, $CDCl_3$, 298 K) of **7a**

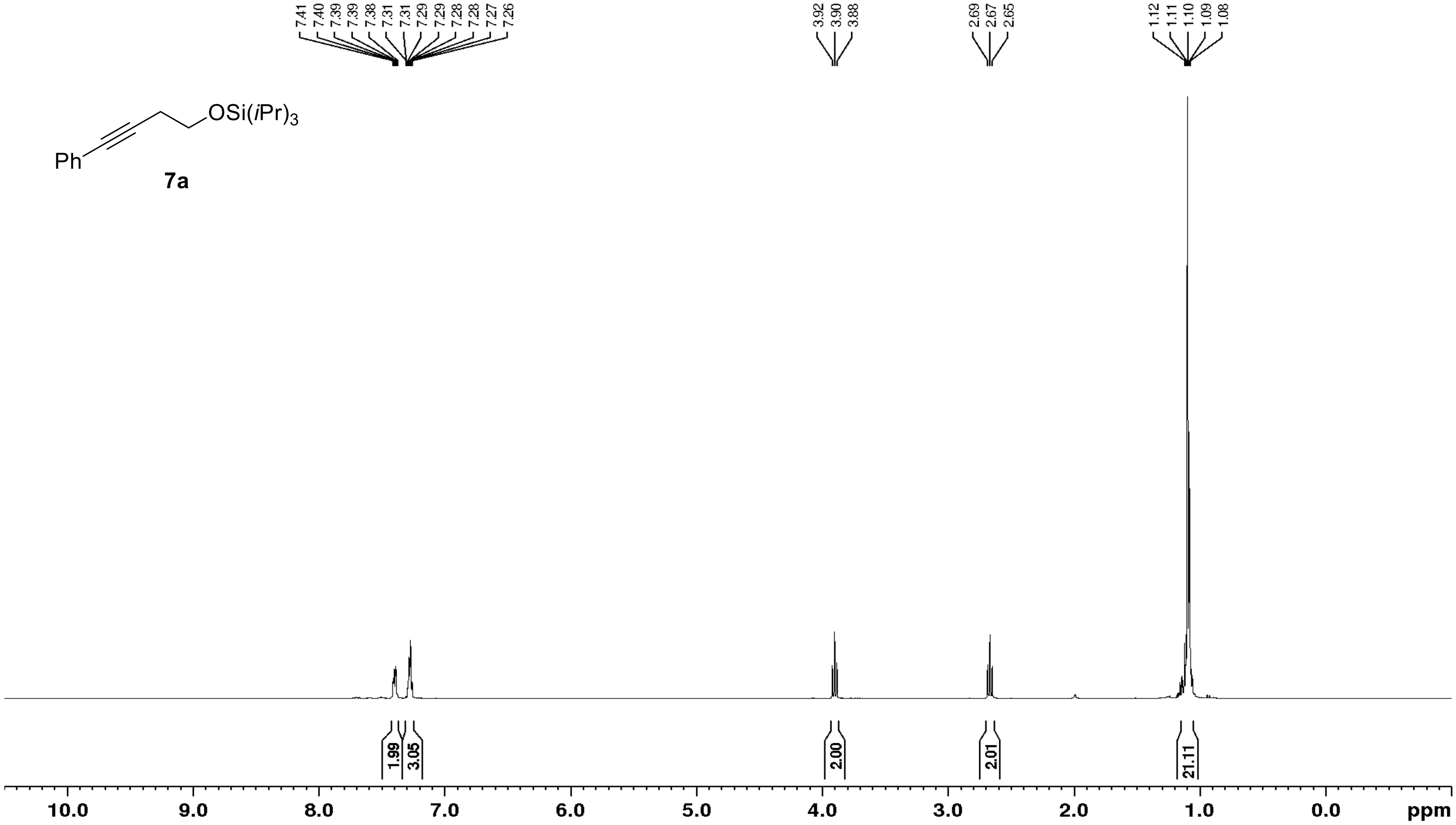

Figure S190. $^{13}C\{^1H\}$ NMR spectrum (101 MHz, $CDCl_3$, 298 K) of **7a**

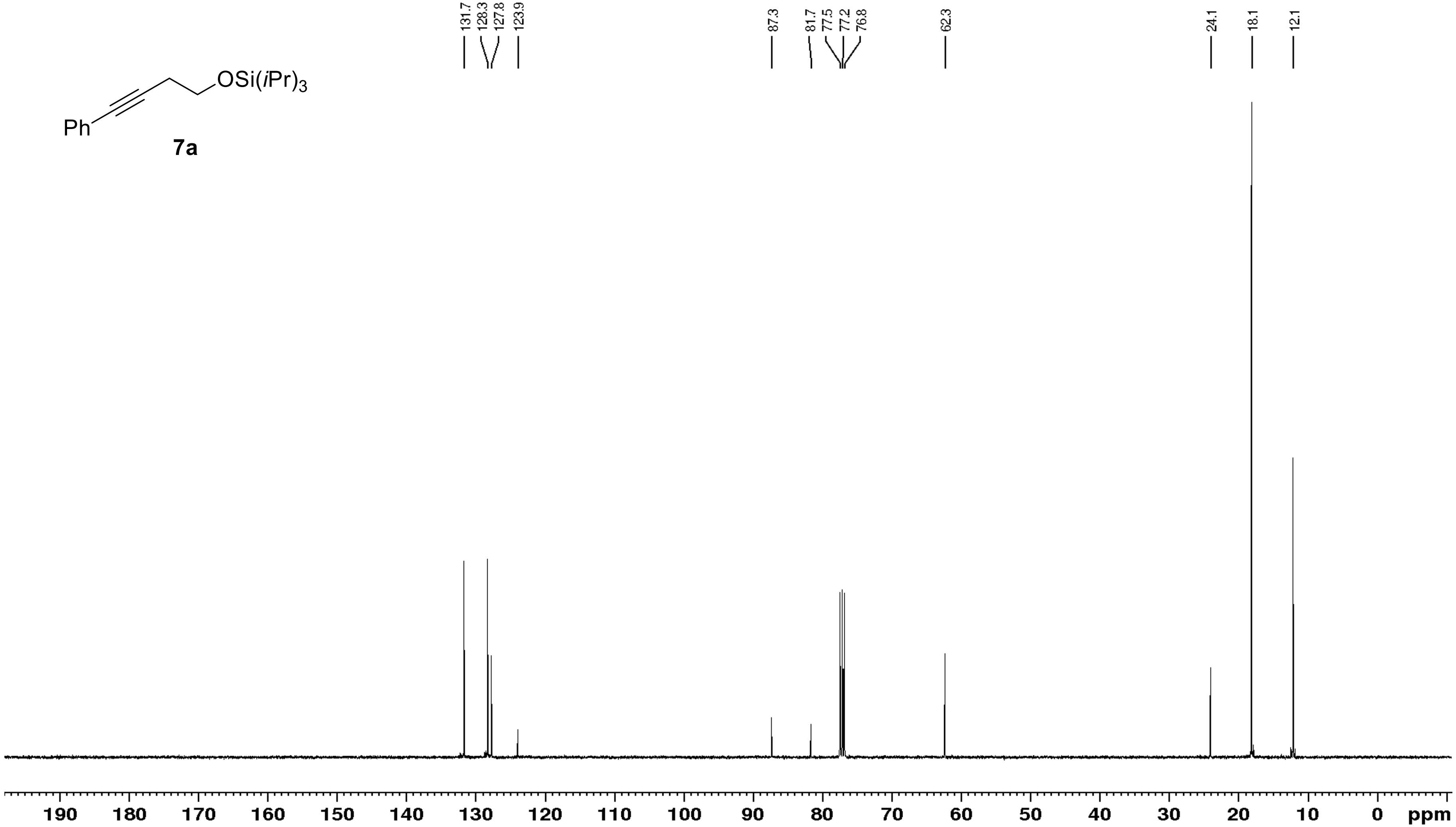

Figure S191. ^{29}Si DEPT NMR spectrum (79 MHz, $CDCl_3$, 298 K, optimized for *J* = 15.0 Hz) of **7a**

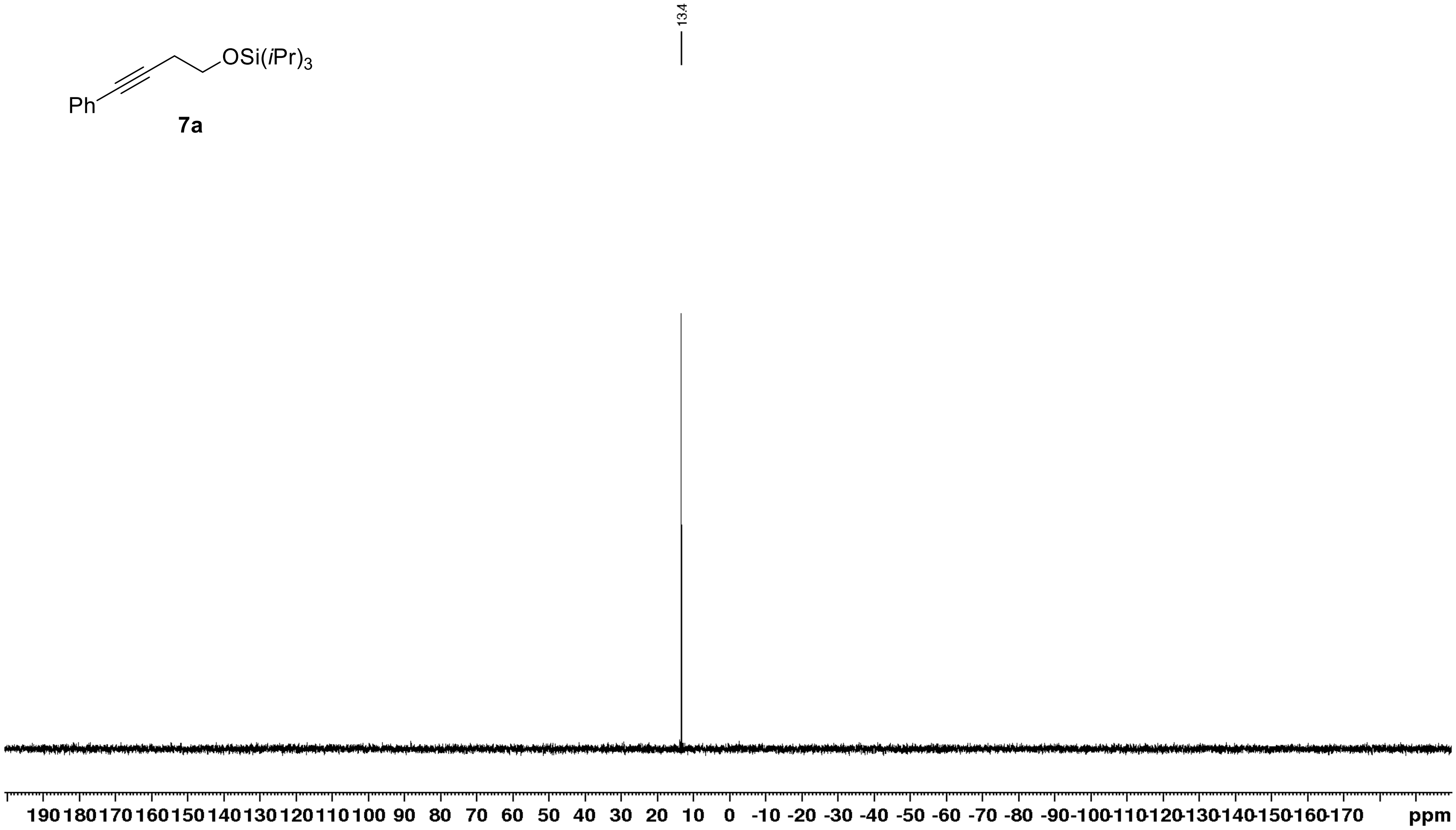

Figure S192. ^{1}H NMR spectrum (400 MHz, $CDCl_3$, 298 K) of **8a**

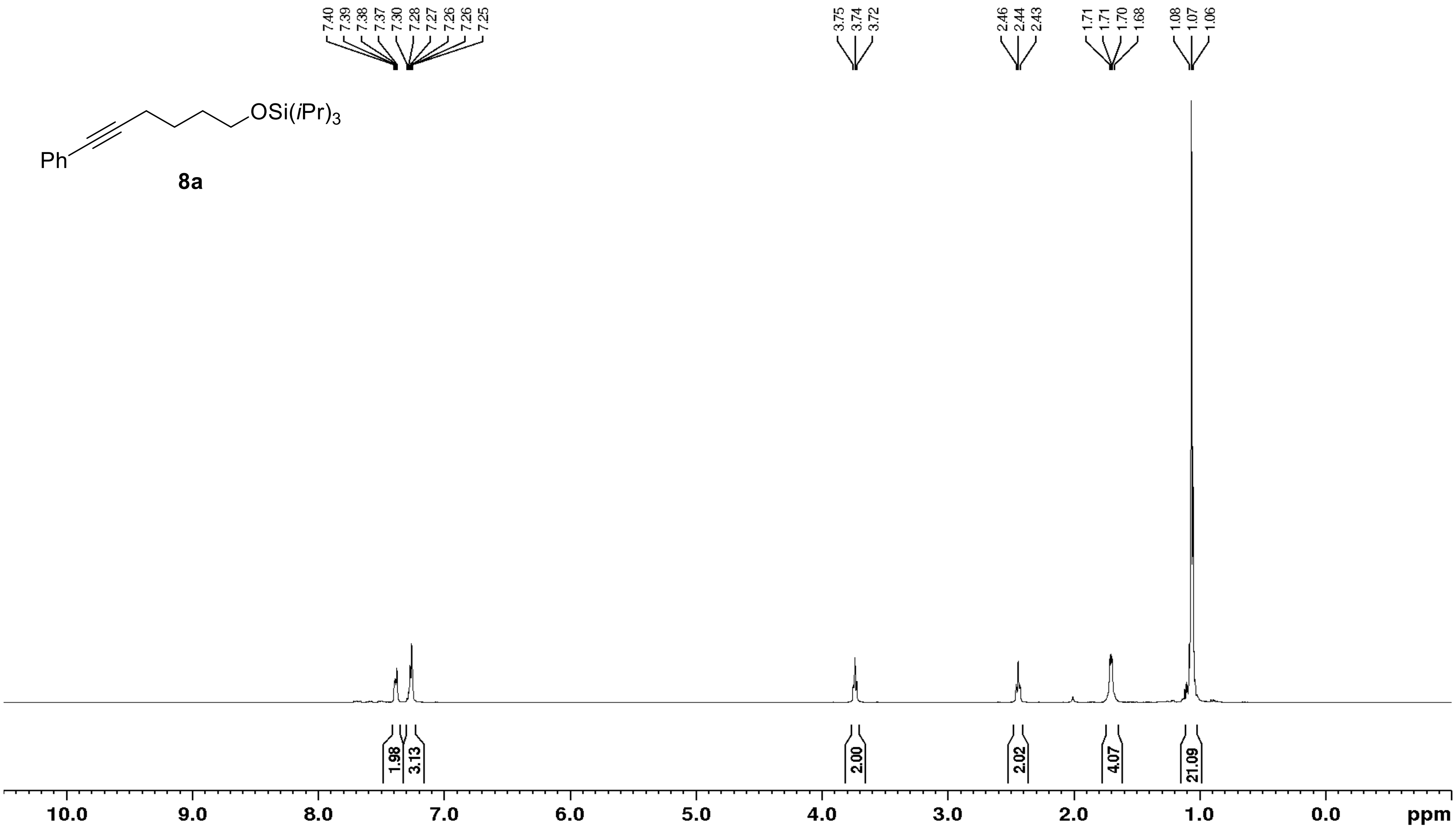

Figure S193. $^{13}C\{^{1}H\}$ NMR spectrum (101 MHz, $CDCl_3$, 298 K) of **8a**

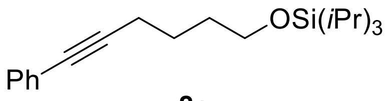

8a

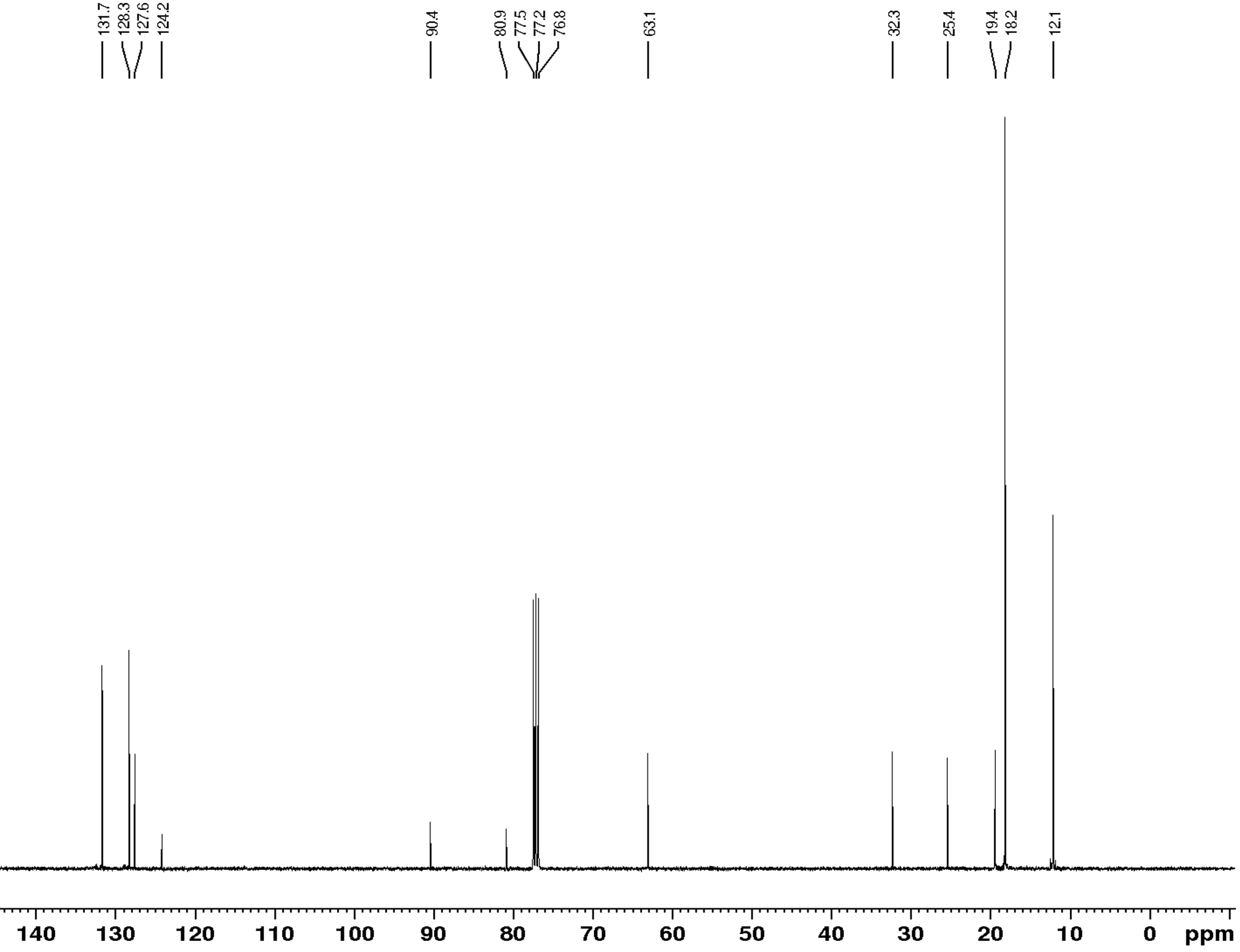

Figure S194. ^{29}Si DEPT NMR spectrum (79 MHz, $CDCl_3$, 298 K, optimized for J = 15.0 Hz) of **8a**

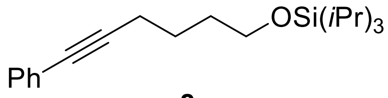

8a

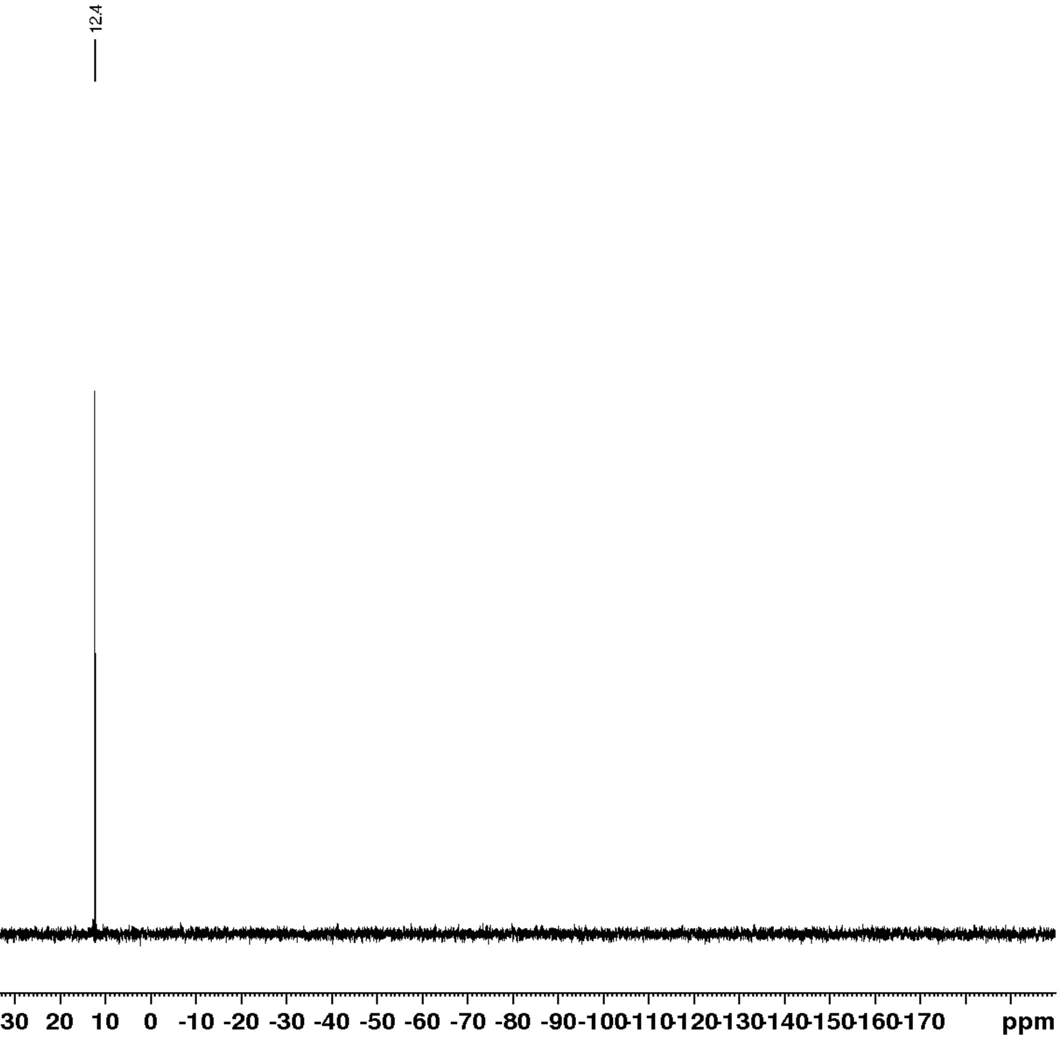

Figure S195. ^{1}H NMR spectrum (400 MHz, $CDCl_3$, 298 K) of **13**

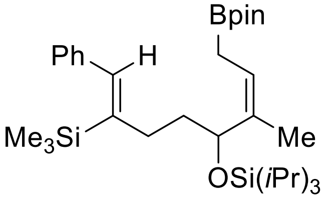

13

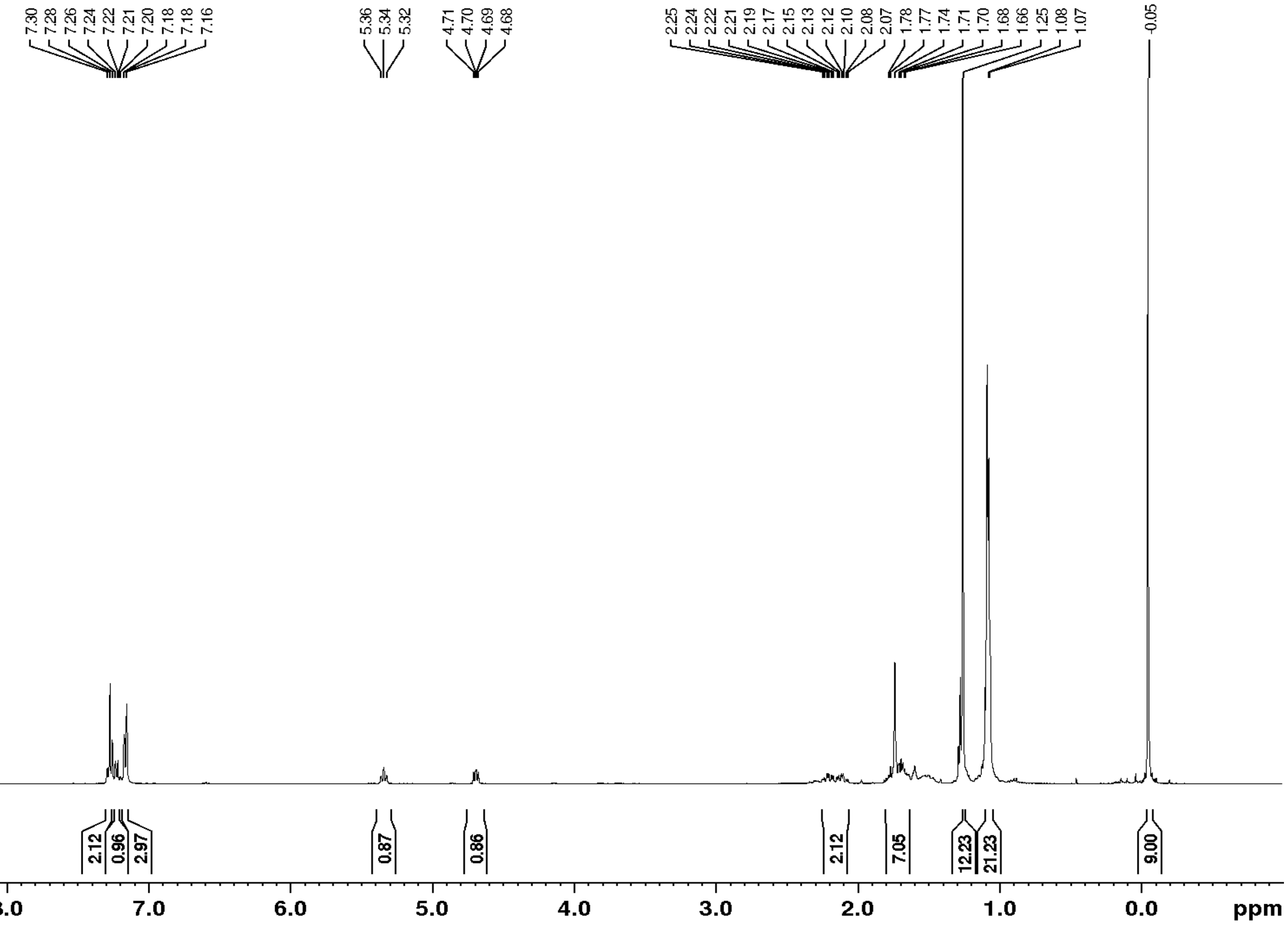

Figure S196. $^{13}C\{^{1}H\}$ NMR spectrum (101 MHz, $CDCl_3$, 298 K) of **13**

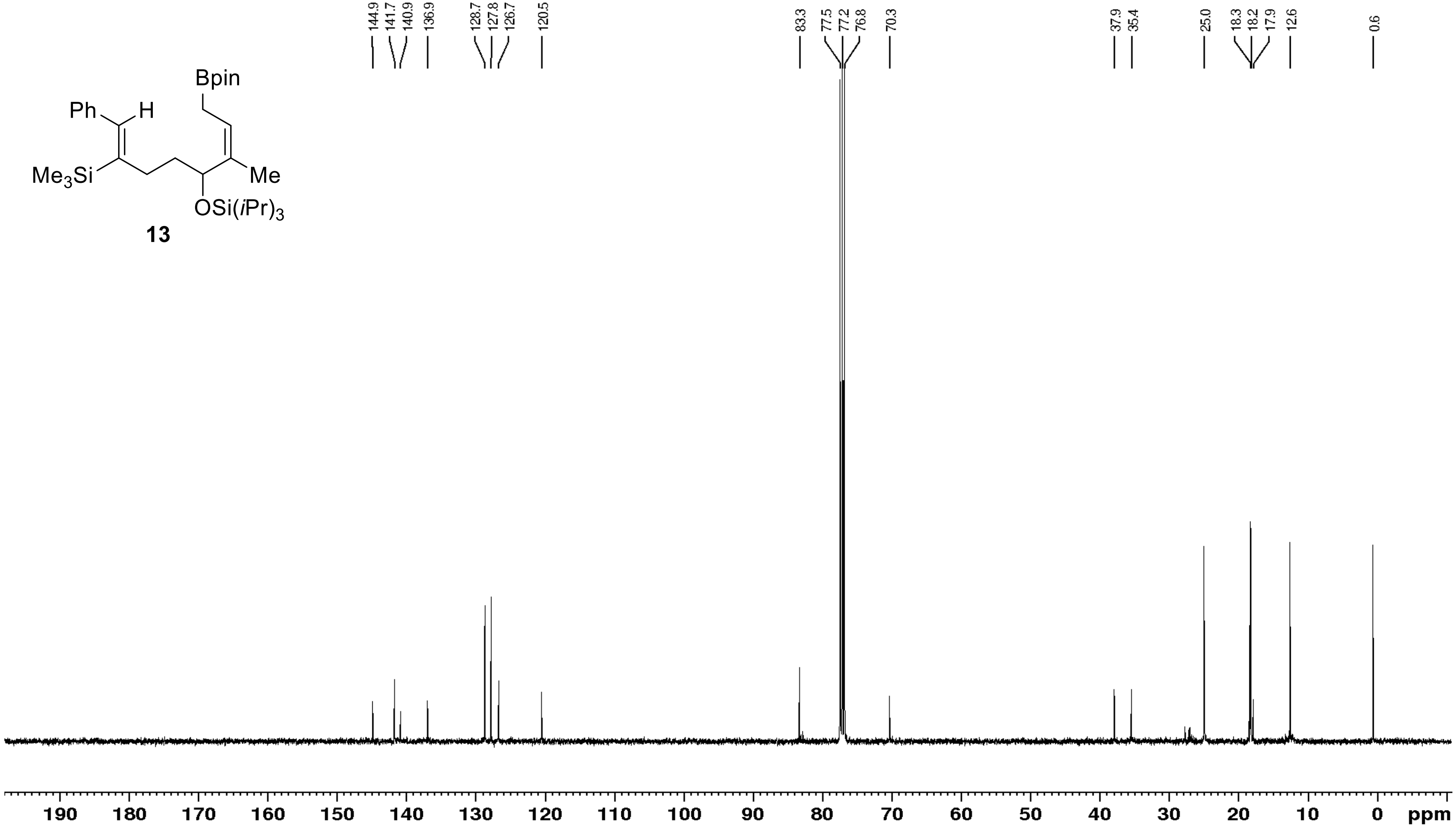

Figure S197. ^{29}Si DEPT NMR spectrum (79 MHz, $CDCl_3$, 298 K, optimized for J = 15.0 Hz) of **13**

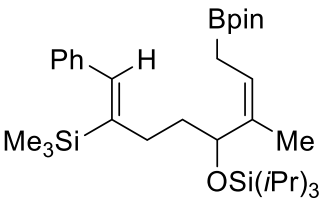

13

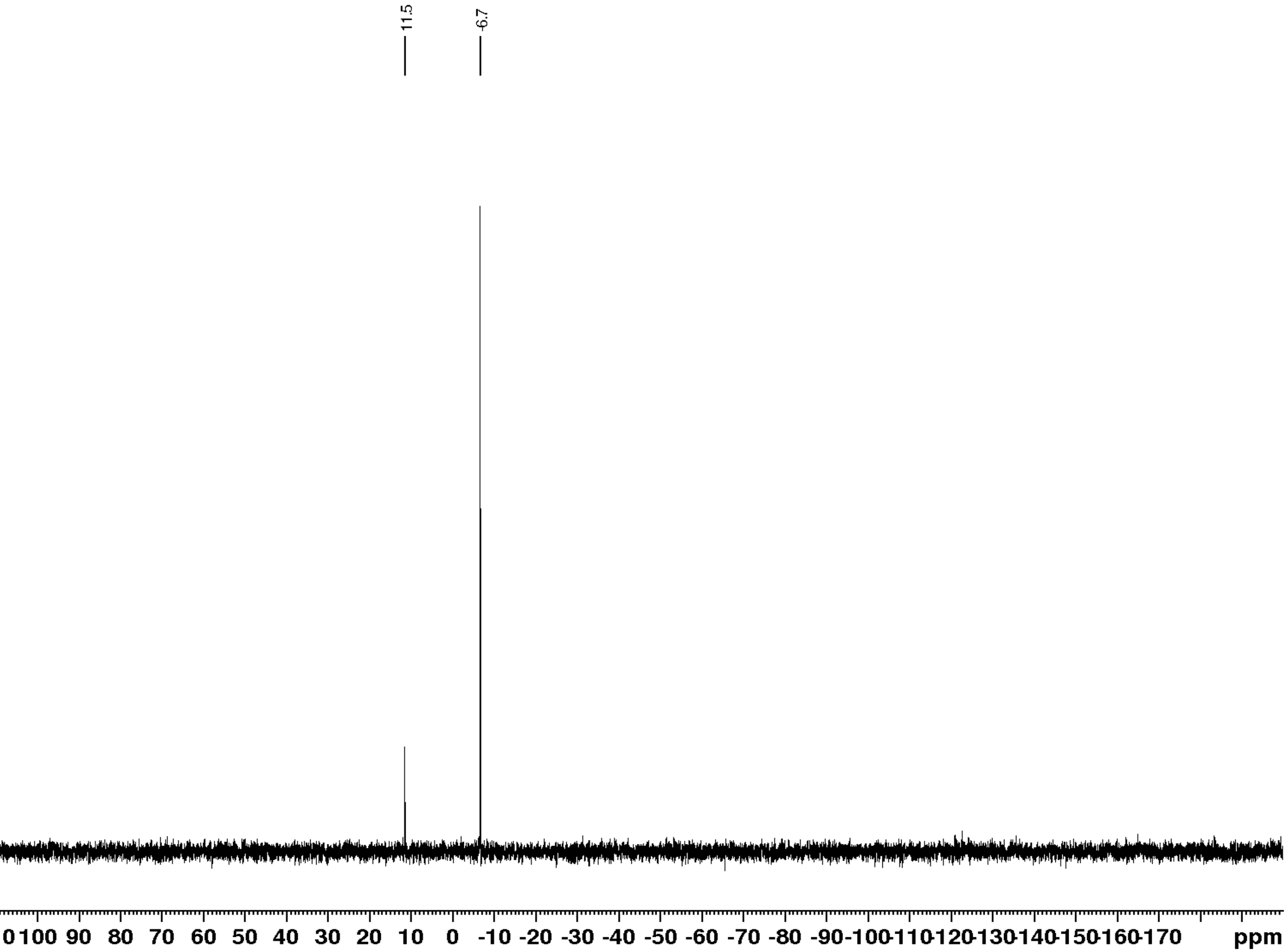

Figure S198. ^{11}B NMR spectrum (128 MHz, $CDCl_3$, 298 K) of **13**

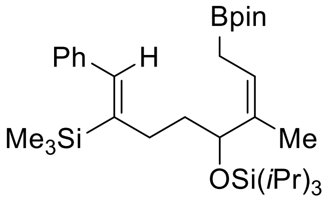

13

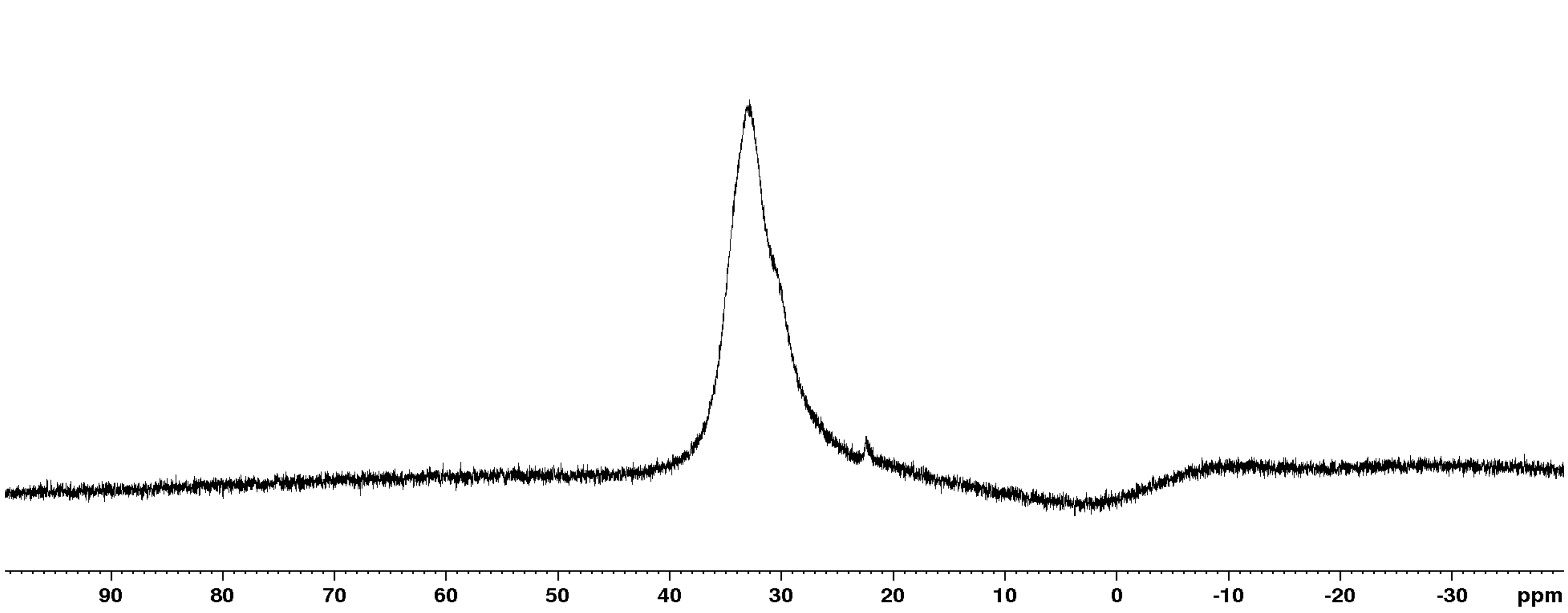

Figure S199. ^{1}H NMR spectrum (400 MHz, $CDCl_3$, 298 K) of **14**

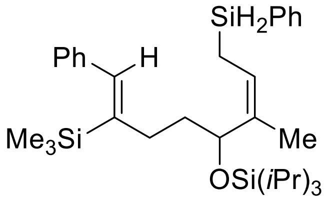

14

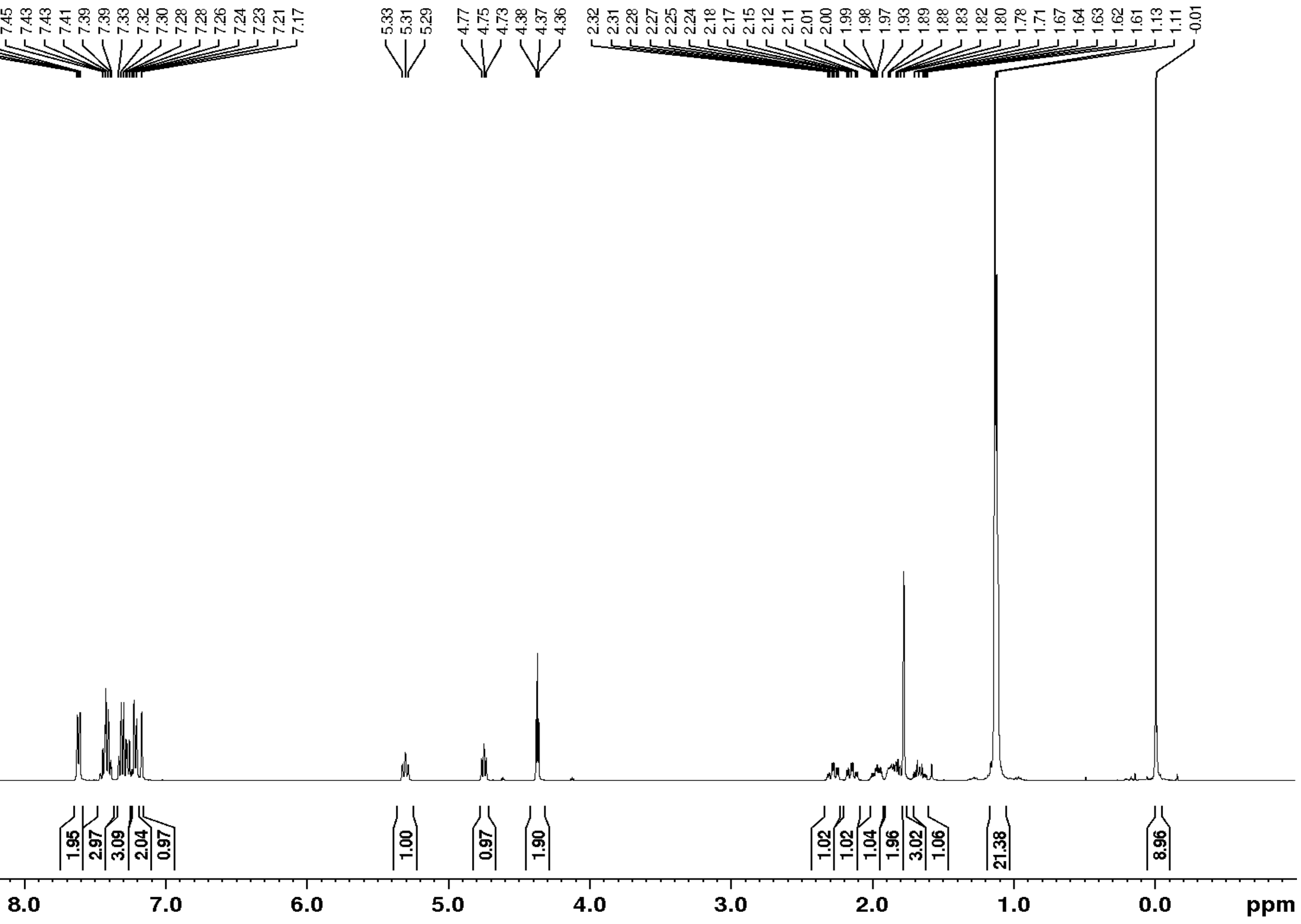

Figure S200. $^{13}C\{^1H\}$ NMR spectrum (101 MHz, $CDCl_3$, 298 K) of **14**

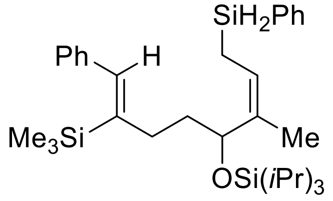

14

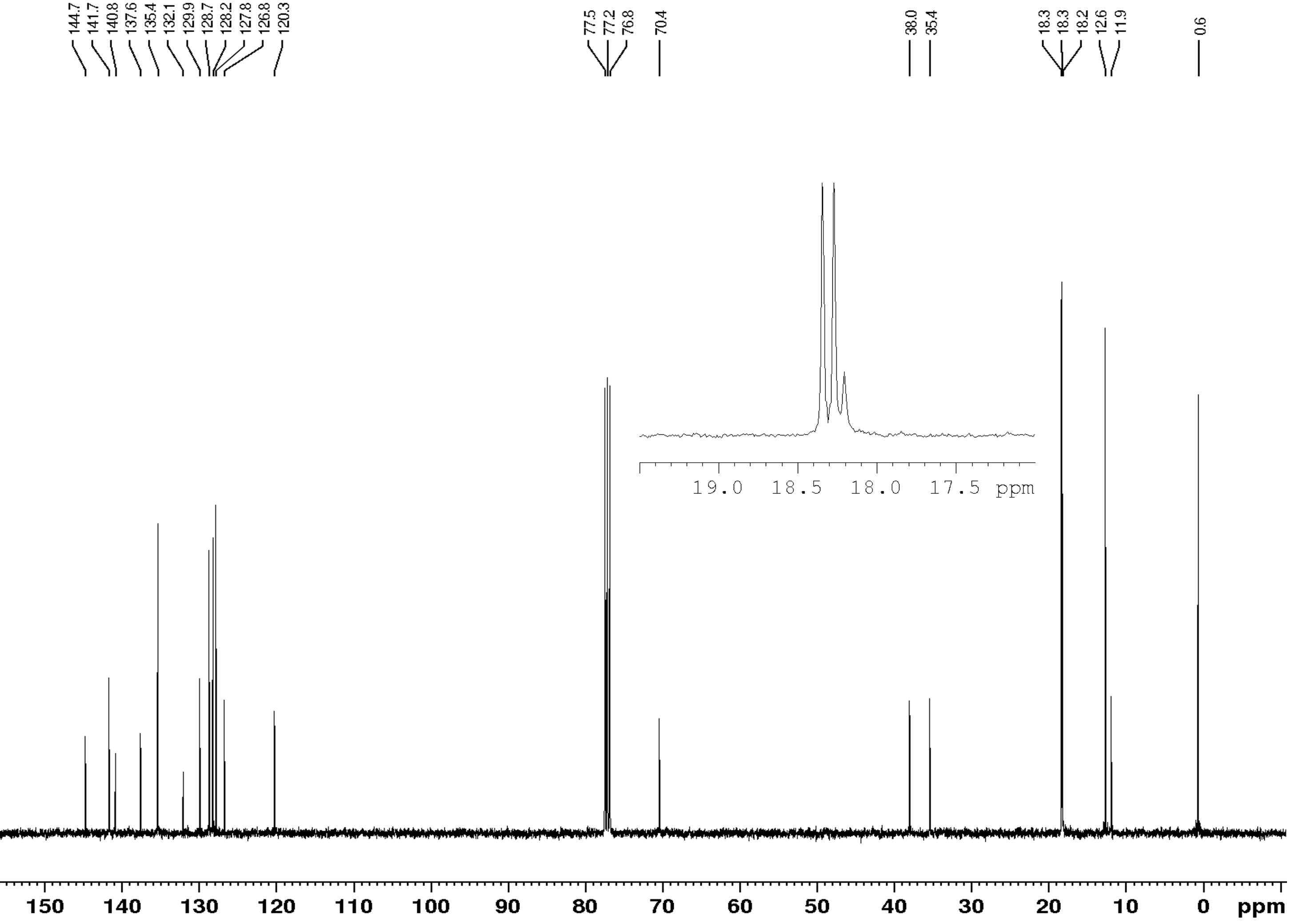

Figure S201. ^{29}Si DEPT NMR spectrum (79 MHz, $CDCl_3$, 298 K, optimized for J = 7.0 Hz) of **14**

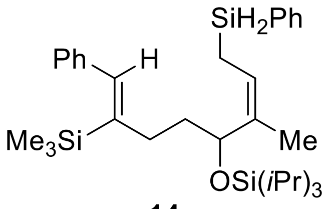

14

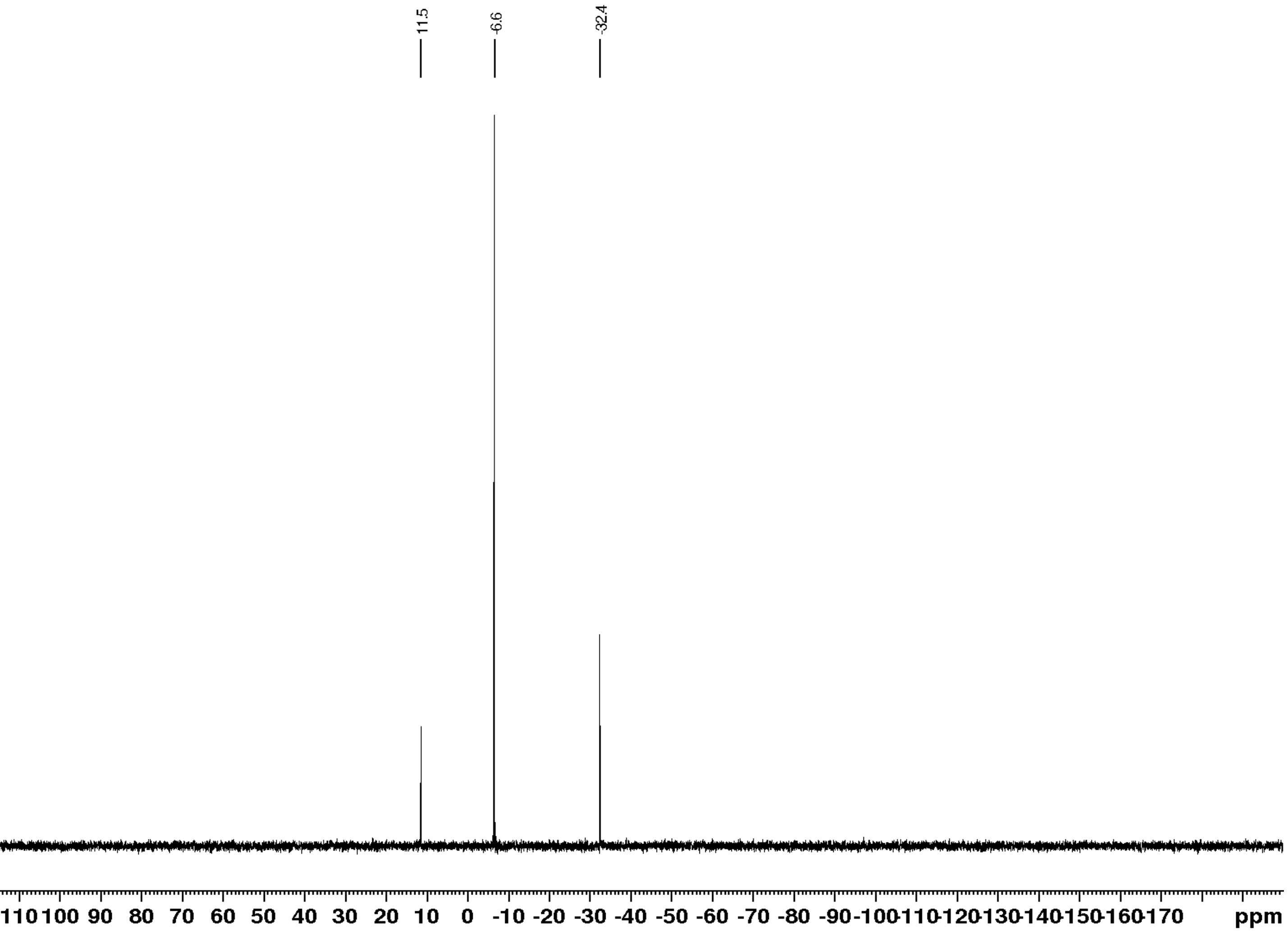

Figure S202. ^{1}H NMR spectrum (400 MHz, $CDCl_3$, 298 K) of **15**

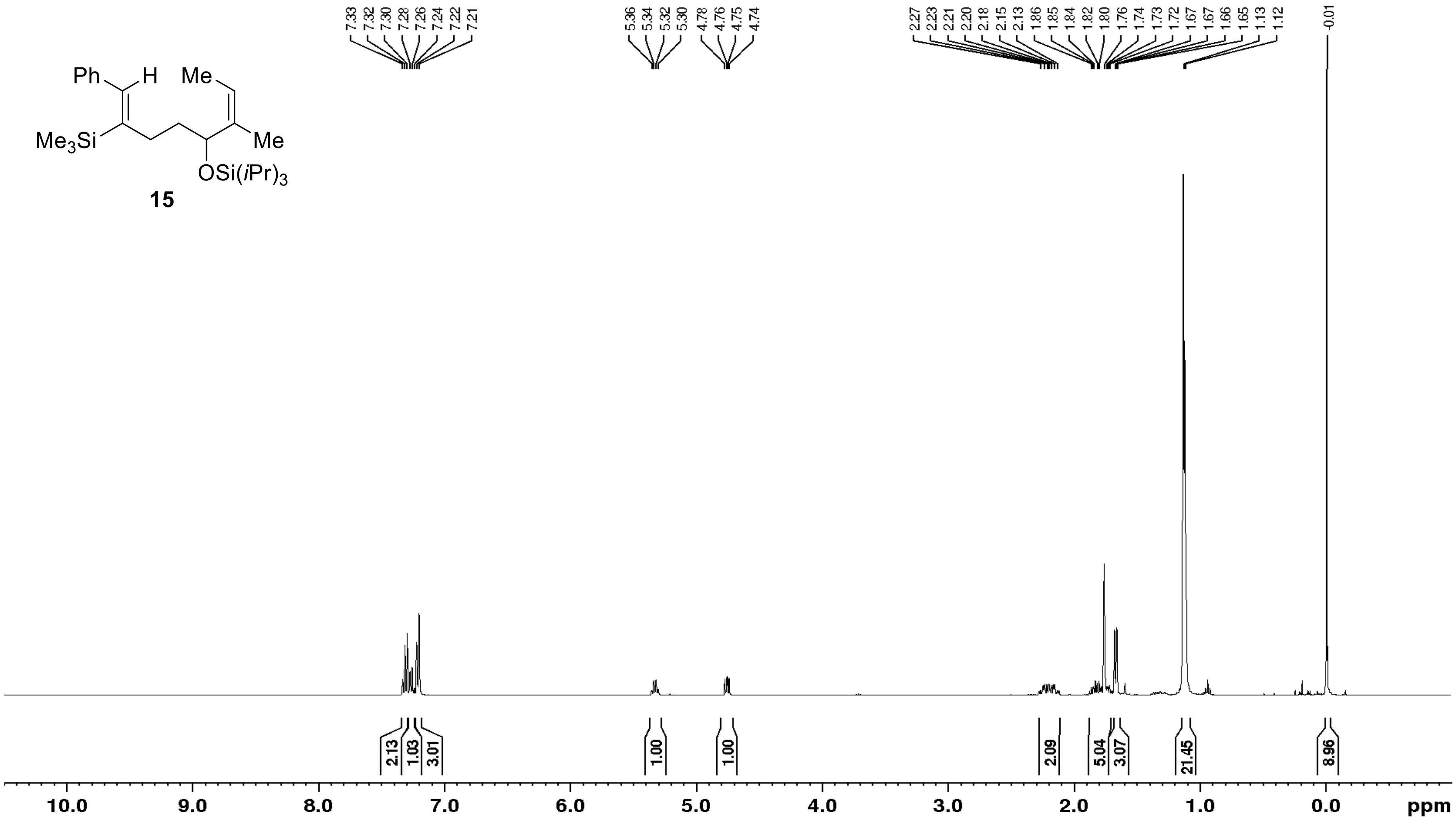

Figure S203. $^{13}C\{^{1}H\}$ NMR spectrum (101 MHz, $CDCl_3$, 298 K) of **15**

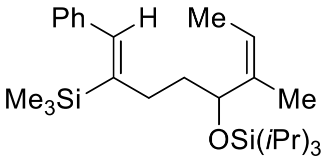

15

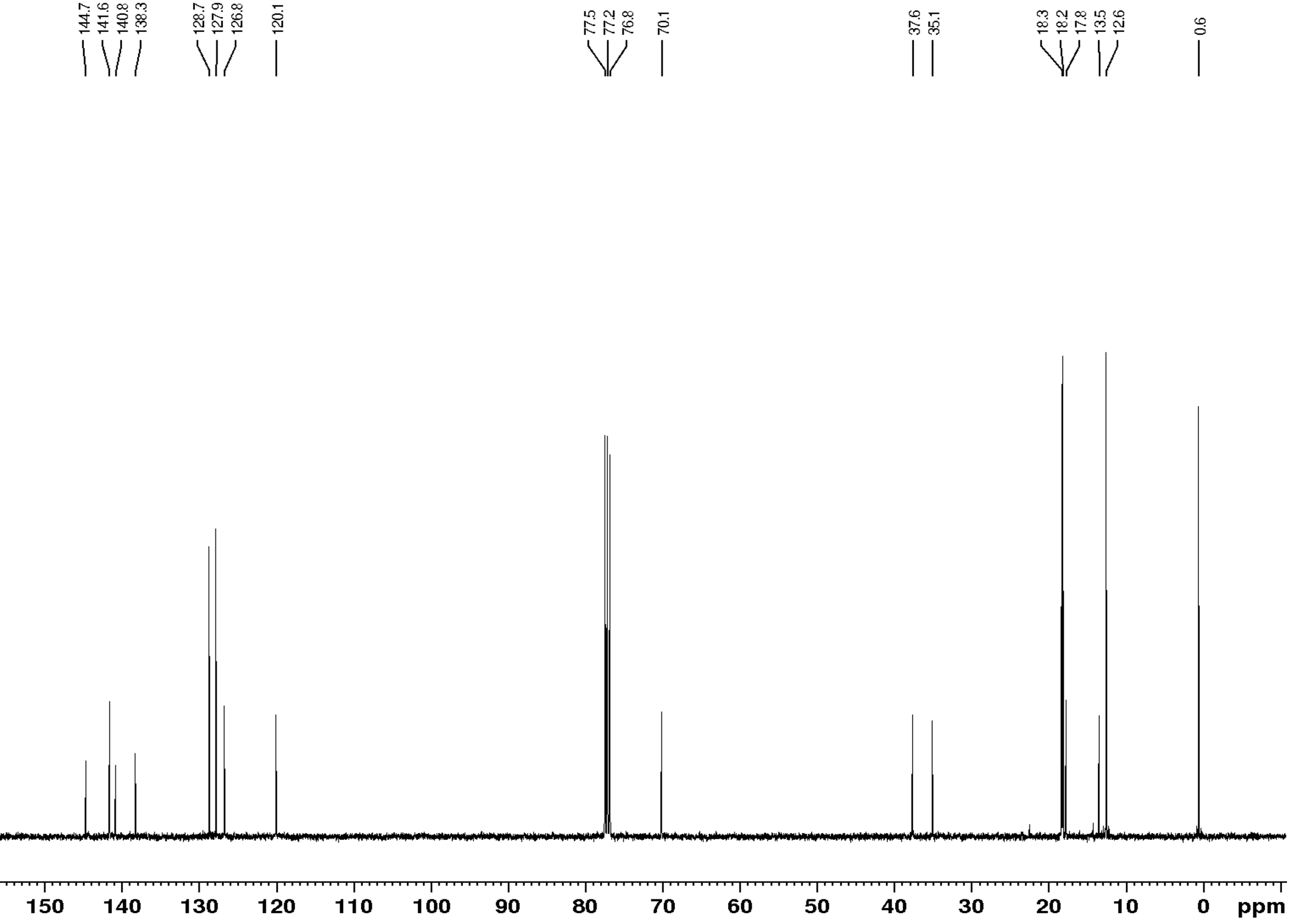

Figure S204. ^{29}Si DEPT NMR spectrum (79 MHz, $CDCl_3$, 298 K, optimized for J = 15.0 Hz) of **15**

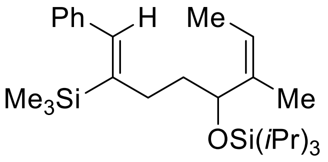

15

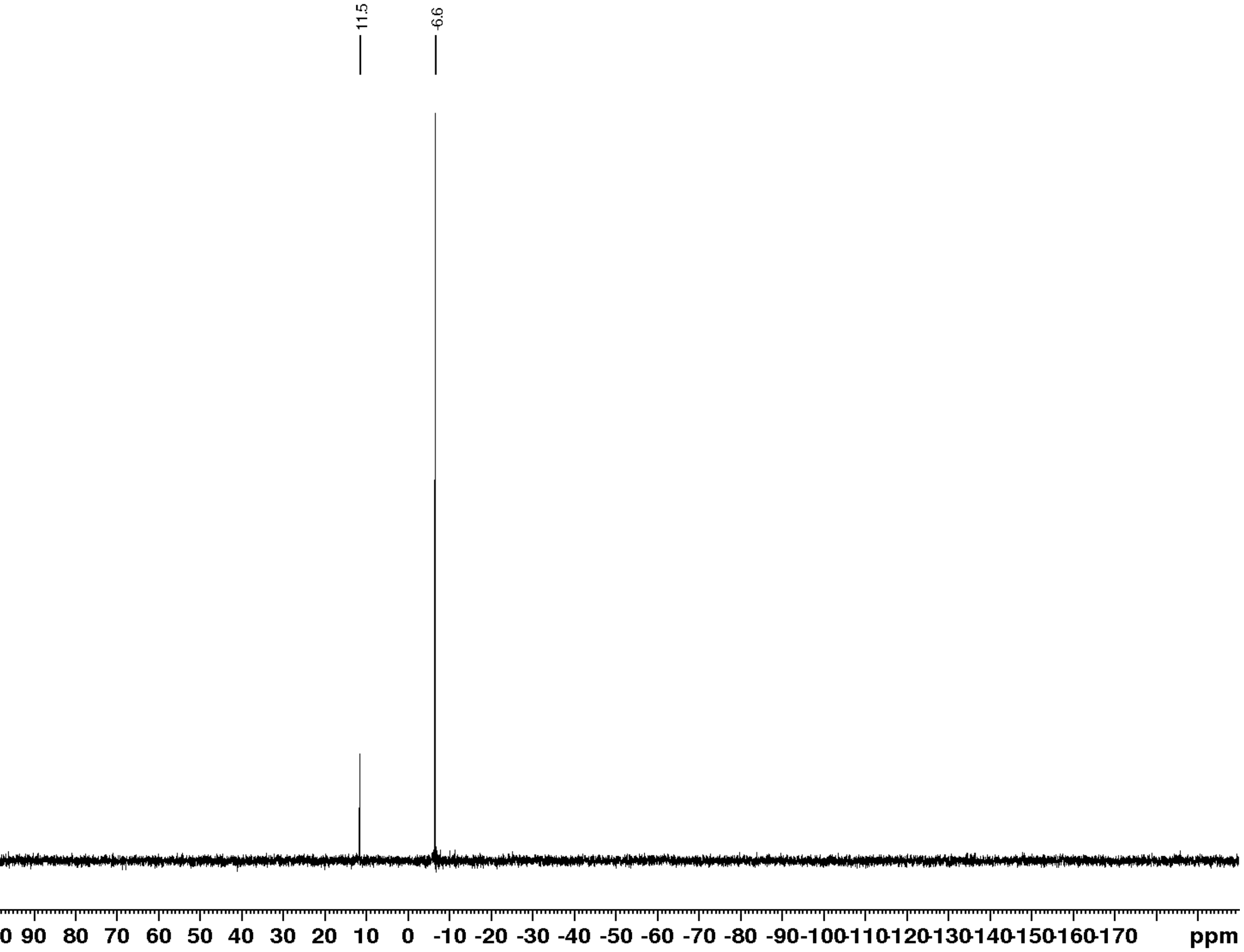

Figure S205. ^{1}H NMR spectrum (500 MHz, $CDCl_3$, 298 K) of **16**

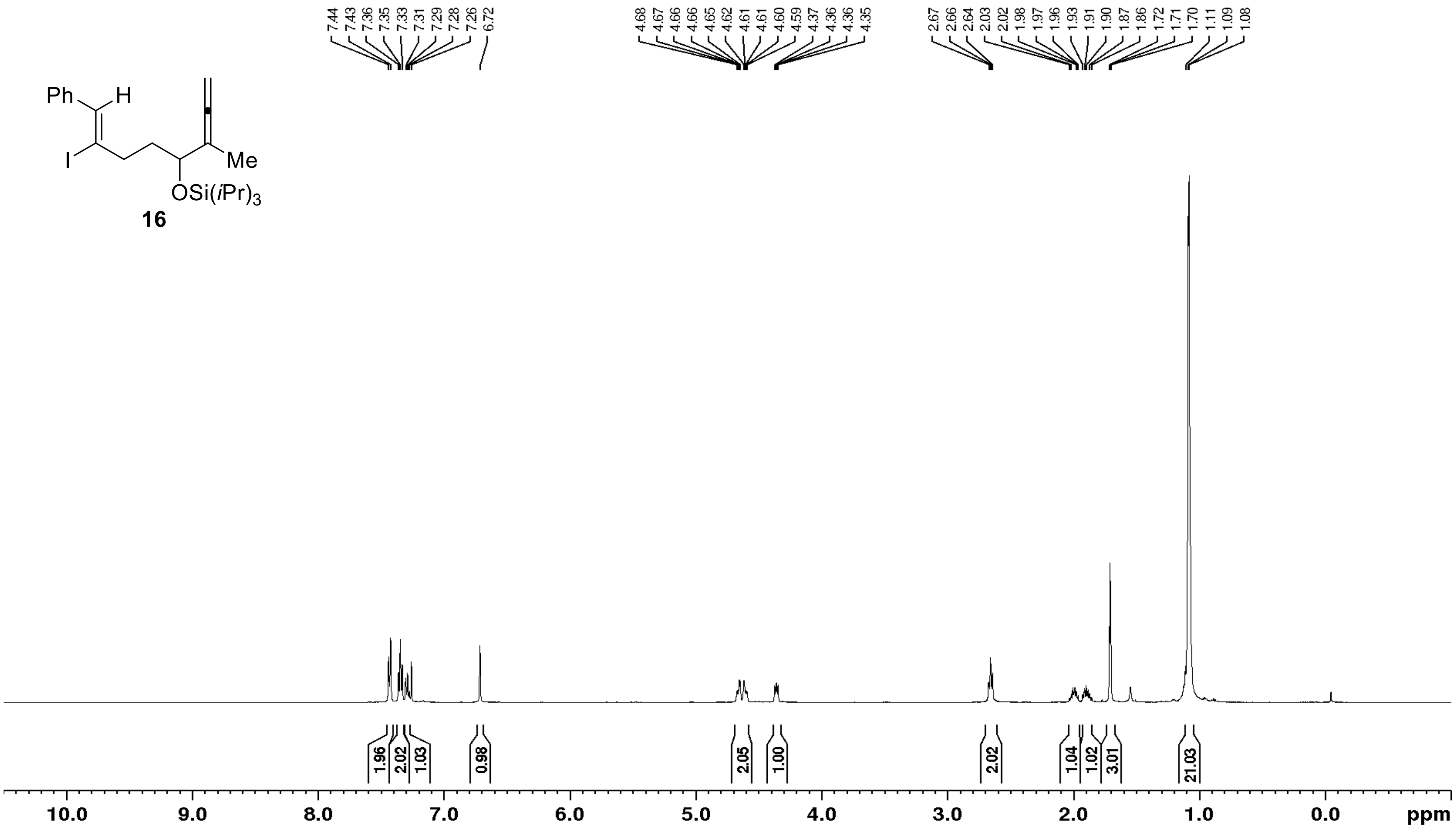

Figure S206. $^{13}C\{^{1}H\}$ NMR spectrum (126 MHz, $CDCl_3$, 298 K) of **16**

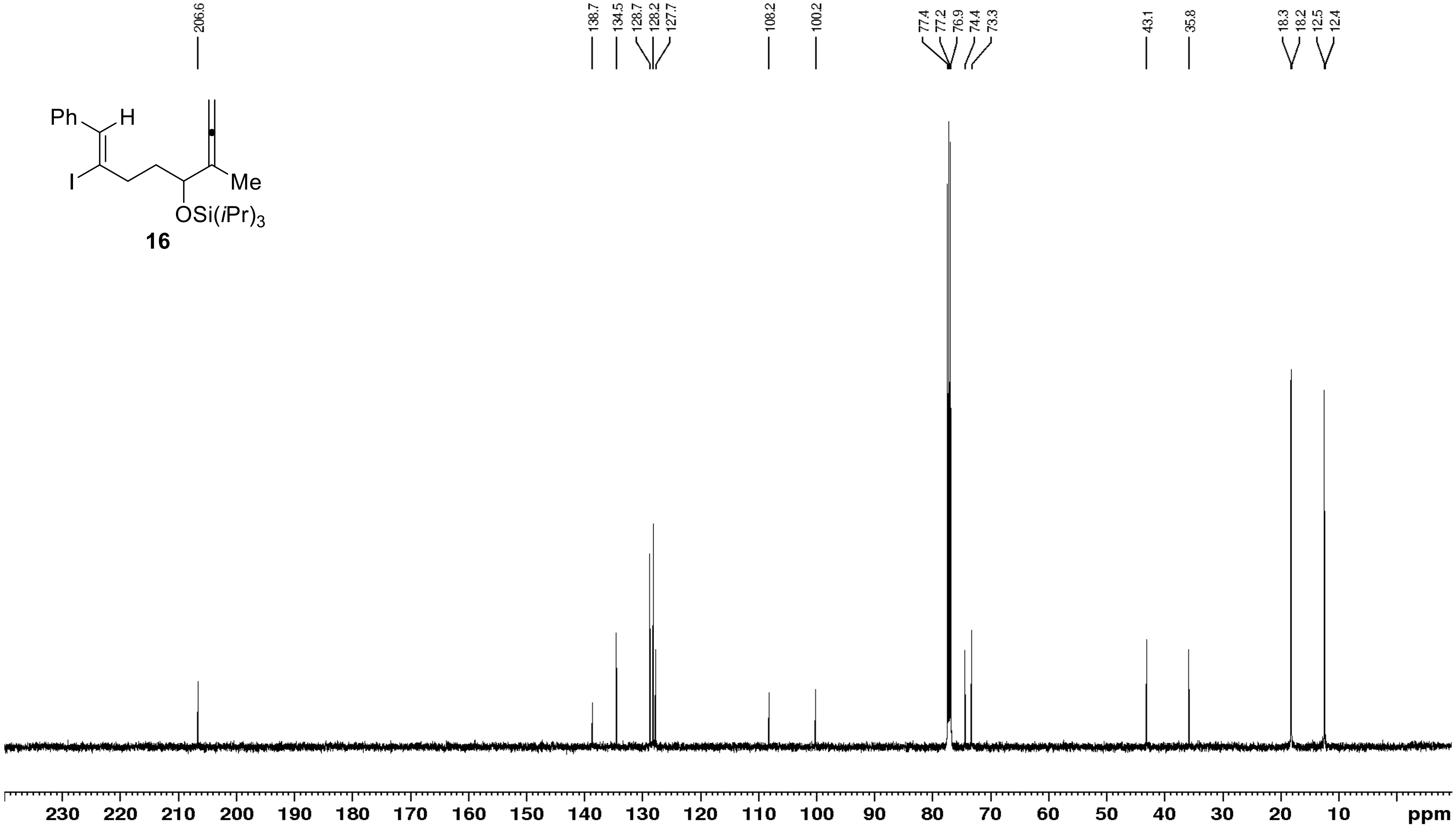

Figure S207. ^{29}Si DEPT NMR spectrum (99 MHz, $CDCl_3$, 298 K, optimized for J = 15.0 Hz) of **16**

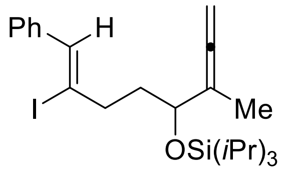

16

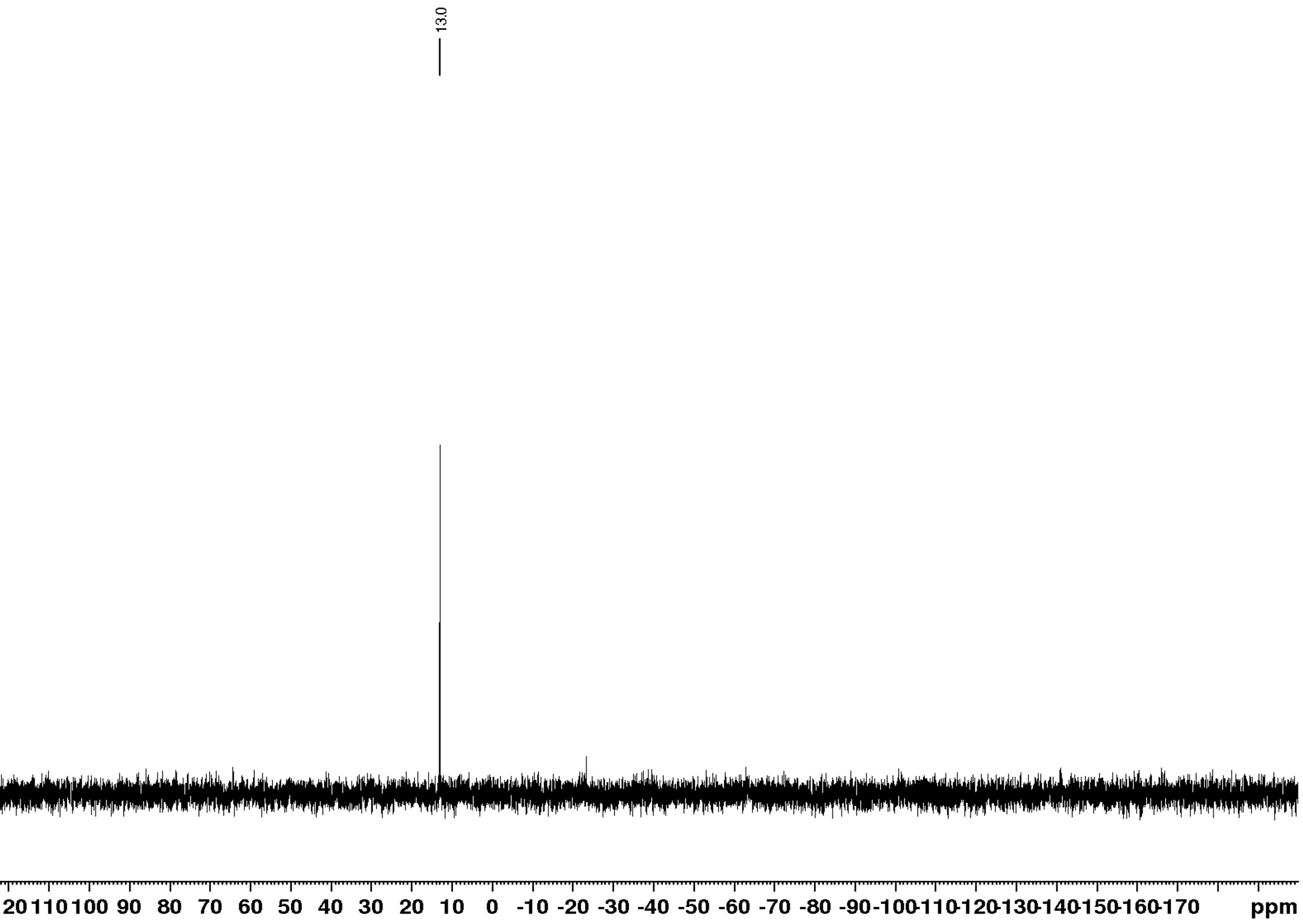

Figure S208. ^{1}H NMR spectrum (400 MHz, $CDCl_3$, 298 K) of **17**

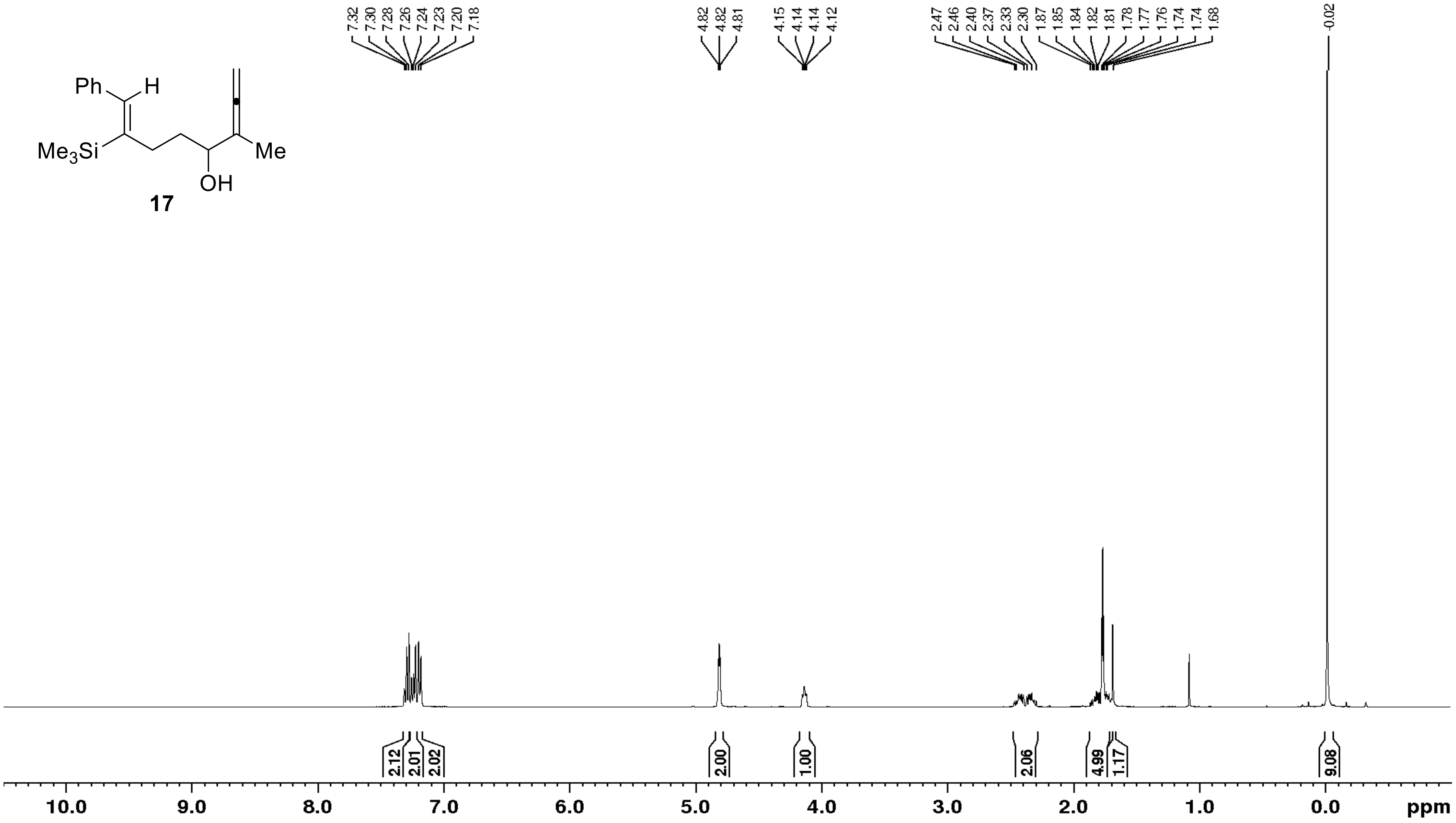

Figure S209. $^{13}C\{^1H\}$ NMR spectrum (101 MHz, $CDCl_3$, 298 K) of **17**

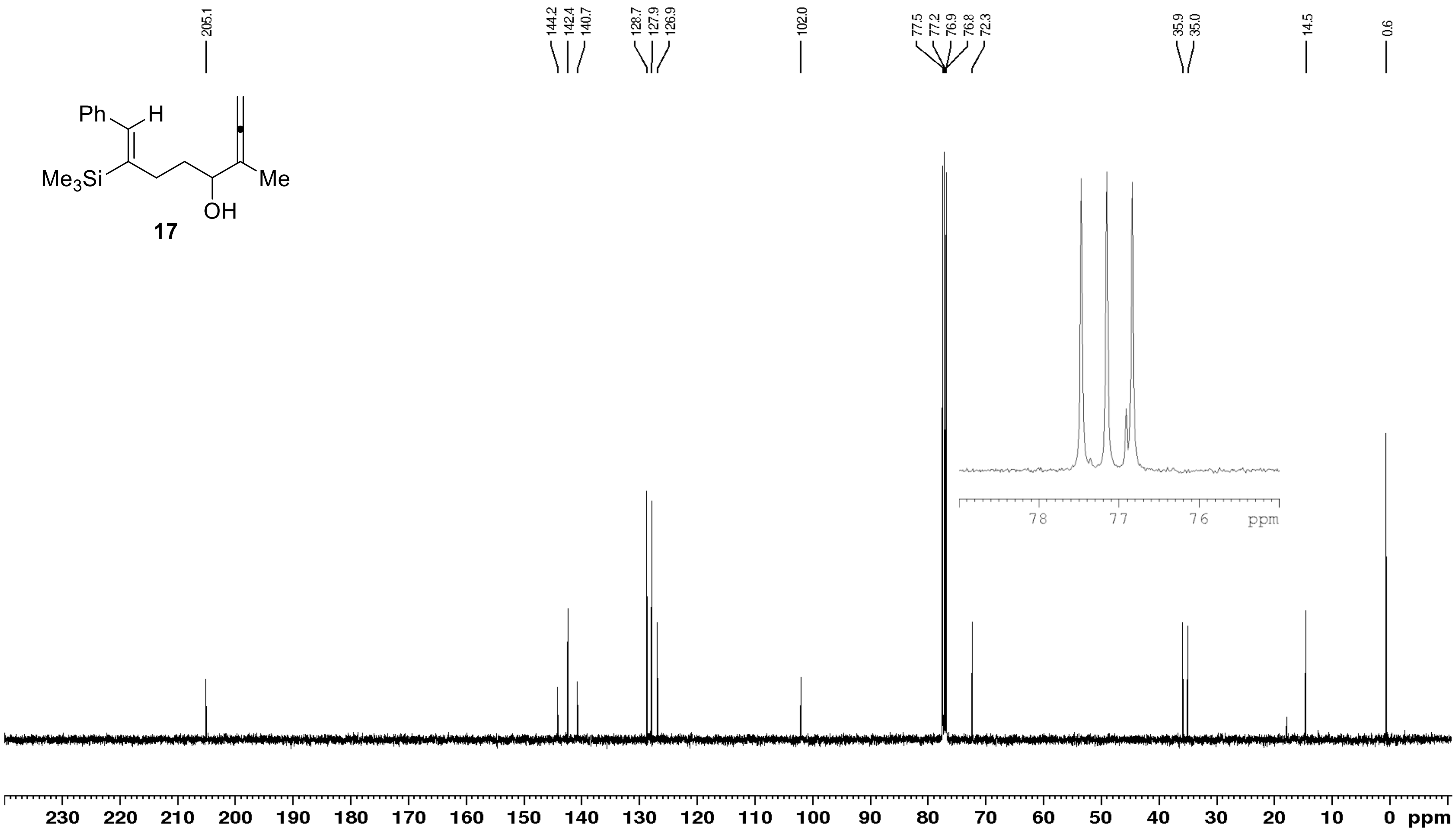

Figure S210. ^{29}Si DEPT NMR spectrum (79 MHz, $CDCl_3$, 298 K, optimized for J = 15.0 Hz) of **17**

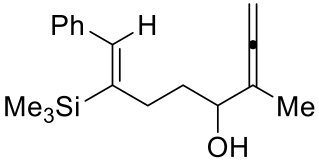

17

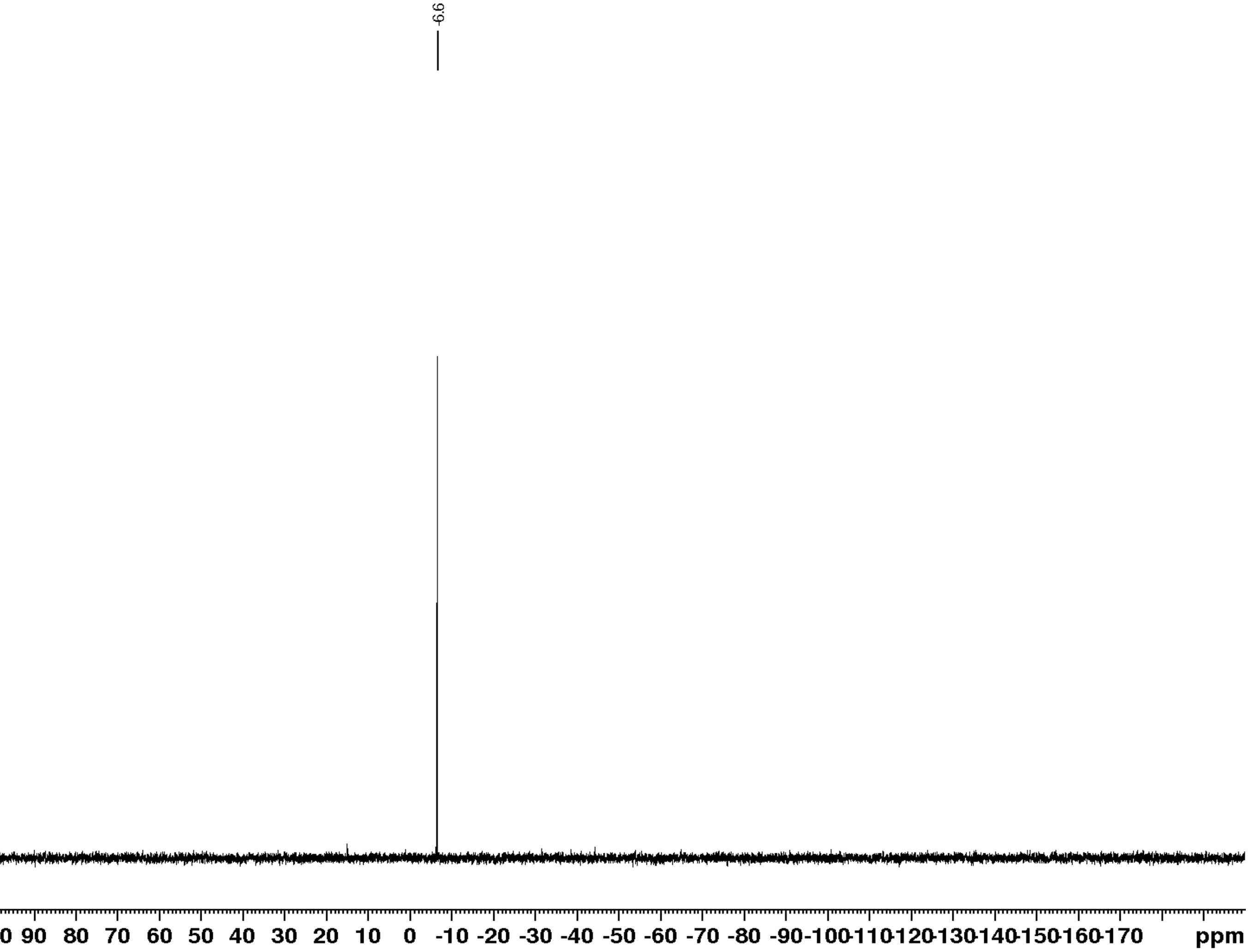

Figure S211. ^{1}H NMR spectrum (400 MHz, $CDCl_3$, 298 K) of **18**

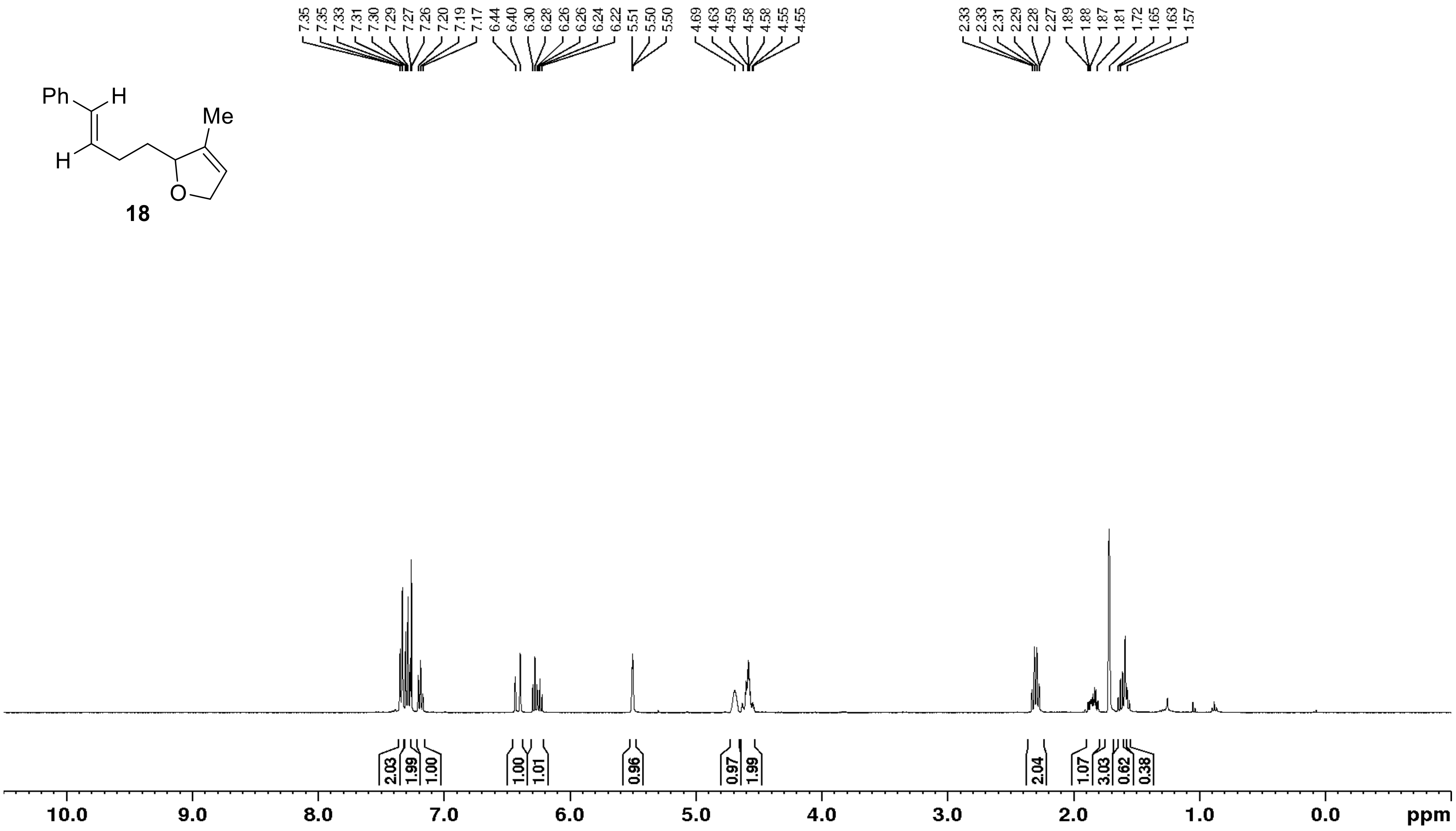

18

Figure S212. $^{13}C\{^{1}H\}$ NMR spectrum (101 MHz, $CDCl_3$, 298 K) of **18**

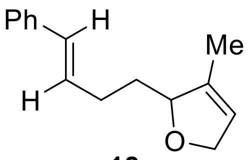

18

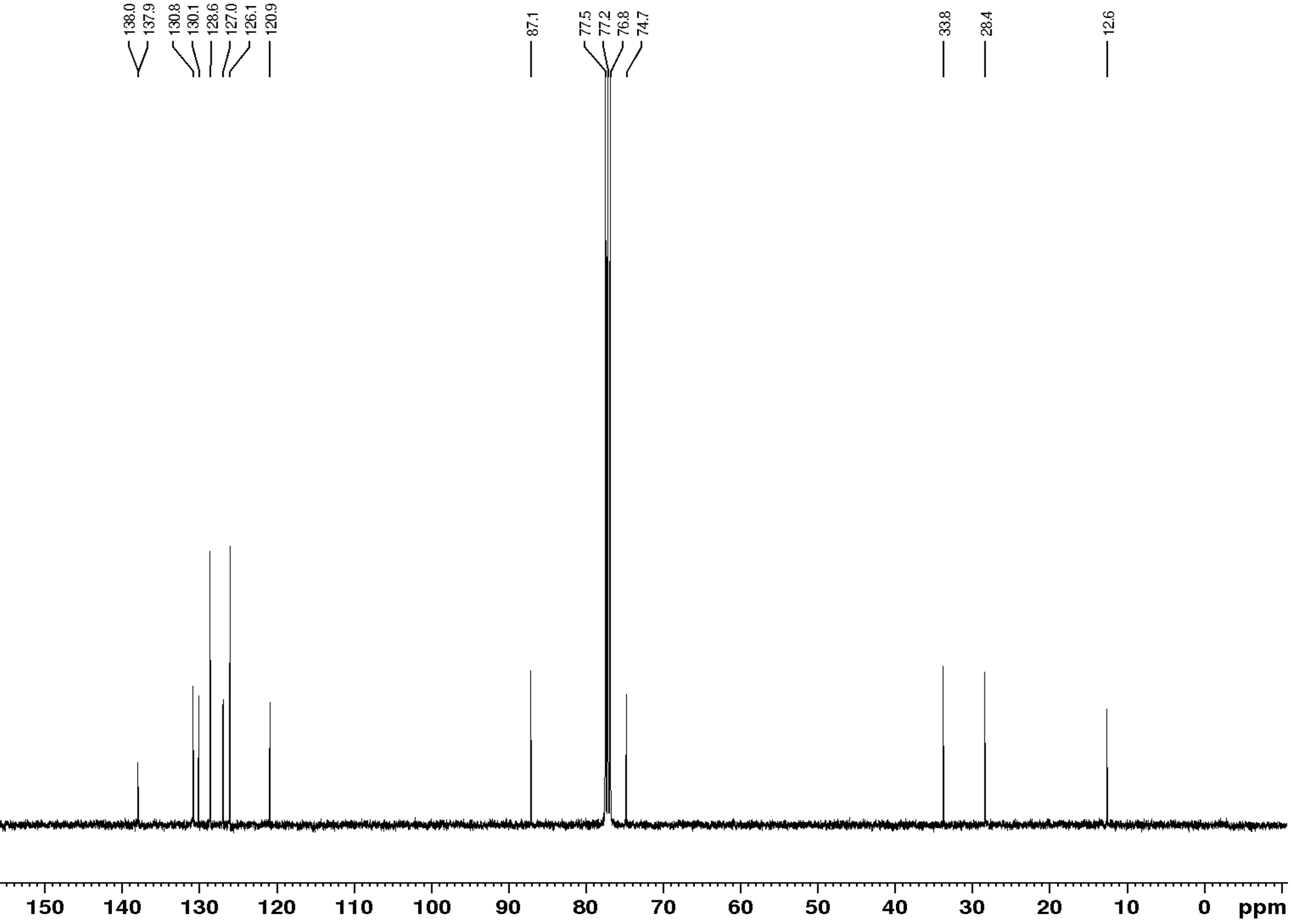

Figure S213. ^{1}H NMR spectrum (400 MHz, $CDCl_3$, 298 K) of **19**

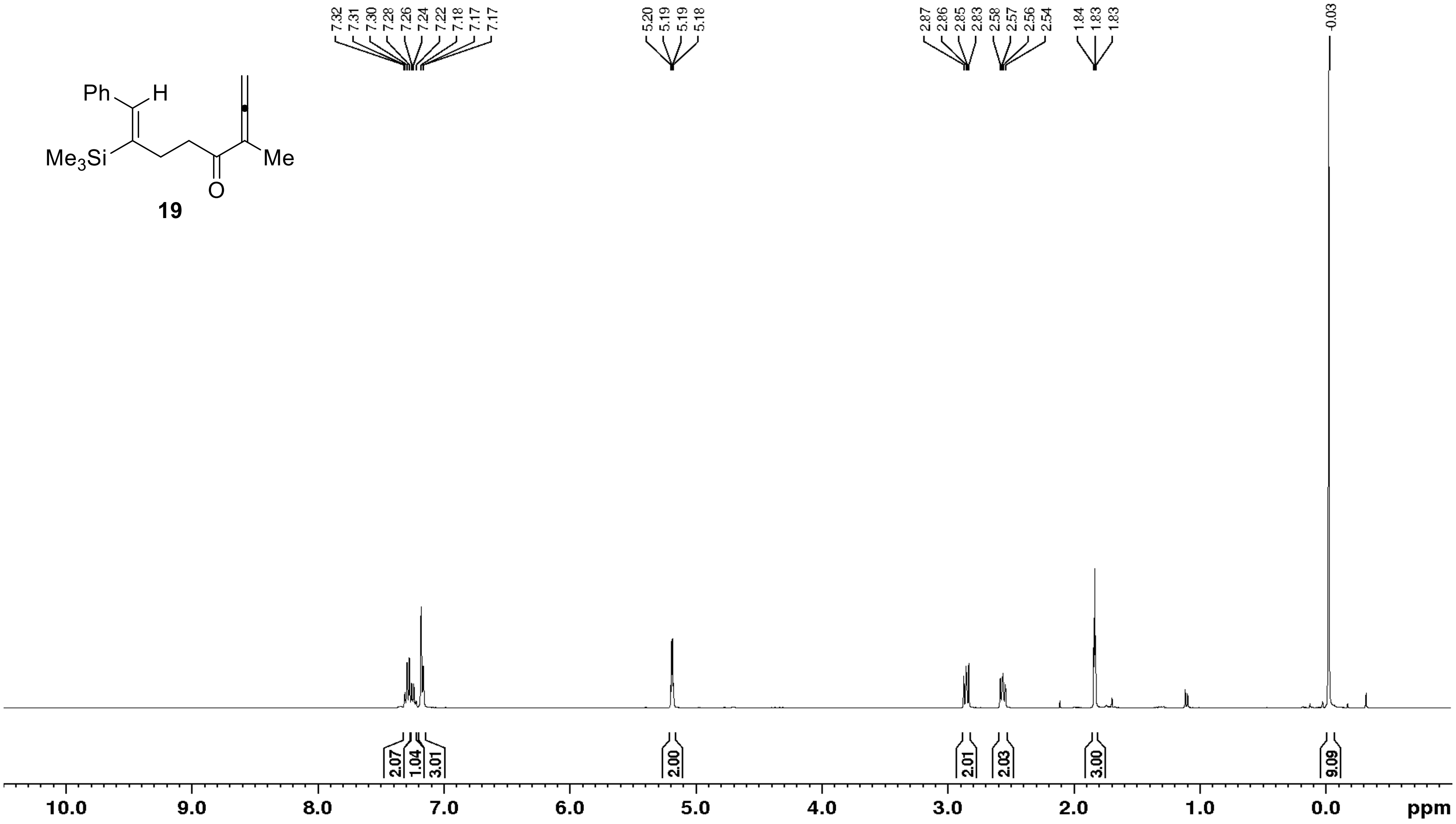

Figure S214. $^{13}C\{^{1}H\}$ NMR spectrum (101 MHz, $CDCl_3$, 298 K) of **19**

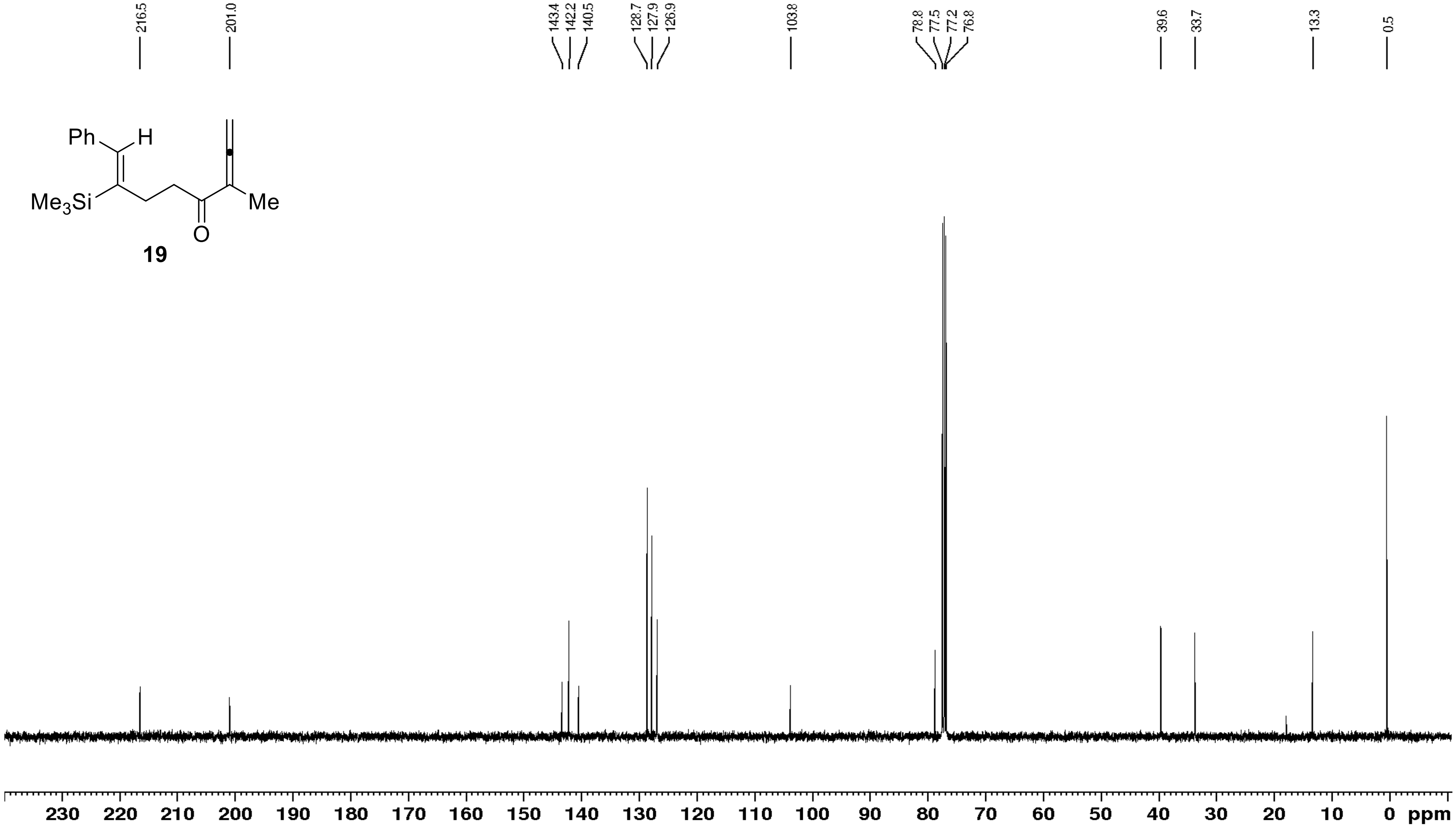

Figure S215. ^{29}Si DEPT NMR spectrum (79 MHz, $CDCl_3$, 298 K, optimized for J = 15.0 Hz) of **19**

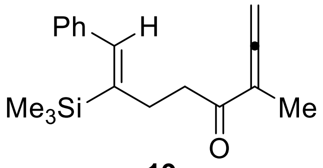

19

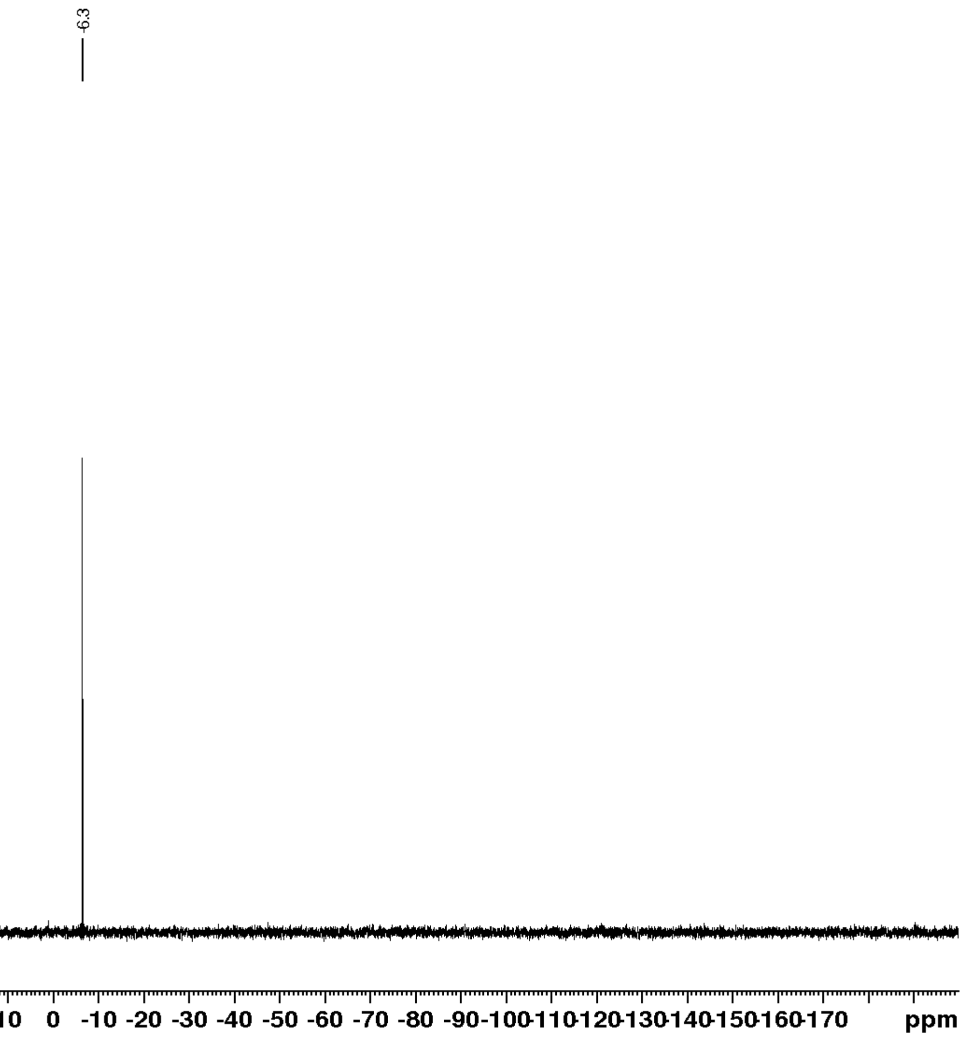

10 References

[S1] Wu, Q.; Roy, A.; Irran, E.; Qu, Z.-W.; Grimme, S.; Klare, H. F. T.; Oestreich, M. Catalytic Difunctionalization of Unactivated Alkenes with Unreactive Hexamethyldisilane through Regeneration of Silylium Ions. *Angew. Chem., Int. Ed.* **2019**, *58*, 17307–17311.

[S2] (a) Reed, C. A. H^+, CH_3^+, and R_3Si^+ Carborane Reagents: When Triflates Fail. *Acc. Chem. Res.* **2010**, *43*, 121–128. (b) He, T.; Wang, G.; Long, P.-W.; Kemper, S.; Irran, E.; Klare, H. F. T.; Oestreich, M. Intramolecular Friedel–Crafts alkylation with a silylium-ion-activated cyclopropyl group: formation of tricyclic ring systems from benzylsubstituted vinylcyclopropanes and hydrosilanes. *Chem. Sci.* **2021**, *12*, 569–575.

[S3] Reed, C. A.; Xie, Z.; Bau, R.; Benesi, A. Closely Approaching the Silylium Ion (R_3Si^+). *Science* **1993**, *262*, 402–404.

[S4] Wu, Q.; Qu, Z.-W.; Omann, L.; Irran, E.; Klare, H. F. T.; Oestreich, M. Cleavage of Unactivated Si−C(sp^3) Bonds with Reed's Carborane Acids: Formation of Known and Unknown Silylium Ions. *Angew. Chem., Int. Ed.* **2018**, *57*, 9176–9179.

[S5] Harris, R. K.; Becker, E. D.; Cabral de Menezes, S. M.; Goodfellow, R.; Granger, P. NMR nomenclature. Nuclear spin properties and conventions for chemical shifts (IUPAC Recommendations 2001). *Pure Appl. Chem*. **2001**, *73*, 1795–1818.

[S6] Grafton, M. W.; Johnson, S. A.; Farrugia, L. J.; Sutherland, A. Diastereoselective synthesis of highly substituted polycyclic scaffolds via a one-pot four-step tandem catalytic process. *Tetrahedron* **2014**, *70*, 7133–7141.

[S7] Holmbo, S. D.; Godfrey, N. A.; Hirner, J. J.; Pronin, S. V. A Catalytic Intermolecular Formal Ene Reaction between Ketone-Derived Silyl Enol Ethers and Alkynes. *J. Am. Chem. Soc.* **2016**, *138*, 12316–12319.

[S8] Zuo, H.; Qu, Z.-W.; Kemper, S.; Klare, H. F. T.; Grimme, S.; Oestreich, M. Silylium-Ion-Promoted (3+2) Annulation of Allenylsilanes with Internal Alkynes Involving a Pentadienyl-to-Allyl Cation. *J. Am. Chem. Soc.* **2024**, *146*, 31377–31383.

[S9] (a) Danheiser, R. L.; Carini, D. J.; Fink, D. M.; Basak, A. Scope and stereochemical course of the (trimethylsilyl)cyclopentene annulation. *Tetrahedron* **1983**, *39*, 935–947. (b) Danheiser, R. L.; Tsai, Y.-M.; Fink, D. M. A General Method for the Synthesis of Allenylsilanes: 1-Methyl-1-(trimethylsilyl)allene. *Org. Synth*. **1988**, *66*, 1.

[S10] Li, J.; Sun, C.; Demerzhan, S.; Lee, D. Metal-Catalyzed Rearrangement of Cyclopropenes to Allenes. *J. Am. Chem. Soc.* **2011**, *133*, 12964–12967.

[S11] Hamasala, G.; Uozumi, Y. Cyclization of alkynoic acids in water in the presence of a vesicular self-assembled amphiphilic pincer palladium complex catalyst. *Chem. Commun.* **2014**, *50*, 14516–14518.

[S12] Peng, X.-X.; Wei, D.; Han, W.-J.; Chen, F.; Yu, W.; Han, B. Dioxygen Activation via Cu-Catalyzed Cascade Radical Reaction: An Approach to Isoxazoline/Cyclic Nitrone-Featured α-Ketols. *ACS Catal.* **2017**, *7*, 7830–7834.
